# Mapping heterogeneity in patient-derived melanoma cultures by single-cell RNA-seq

**DOI:** 10.18632/oncotarget.13666

**Published:** 2016-11-26

**Authors:** Tobias Gerber, Edith Willscher, Henry Loeffler-Wirth, Lydia Hopp, Dirk Schadendorf, Manfred Schartl, Ulf Anderegg, Gray Camp, Barbara Treutlein, Hans Binder, Manfred Kunz

**Affiliations:** ^1^ Department of Evolutionary Genetics, Max Planck Institute for Evolutionary Anthropology Leipzig, 04103 Leipzig, Germany; ^2^ Interdisciplinary Center for Bioinformatics, University of Leipzig, 04107 Leipzig, Germany; ^3^ Department of Dermatology, Venereology and Allergology, University Hospital Essen, 45147 Essen, Germany; ^4^ Department of Physiological Chemistry, University of Würzburg, Biozentrum, Am Hubland, 97074 Würzburg, Germany; ^5^ Comprehensive Cancer Center Mainfranken, University Clinic Würzburg, 97080 Würzburg, Germany; ^6^ Institute for Advanced Study, 3572 Texas A&M University, College Station, Texas 77843-3572, USA; ^7^ Department of Dermatology, Venereology and Allergology, University of Leipzig, 04103 Leipzig, Germany

**Keywords:** melanoma, single cell transcriptome sequencing, stem cells

## Abstract

Recent technological advances in single-cell genomics make it possible to analyze cellular heterogeneity of tumor samples. Here, we applied single-cell RNA-seq to measure the transcriptomes of 307 single cells cultured from three biopsies of three different patients with a *BRAF/NRAS* wild type, *BRAF* mutant/*NRAS* wild type and *BRAF* wild type/*NRAS* mutant melanoma metastasis, respectively. Analysis based on self-organizing maps identified sub-populations defined by multiple gene expression modules involved in proliferation, oxidative phosphorylation, pigmentation and cellular stroma. Gene expression modules had prognostic relevance when compared with gene expression data from published melanoma samples and patient survival data. We surveyed kinome expression patterns across sub-populations of the *BRAF/NRAS* wild type sample and found that CDK4 and CDK2 were consistently highly expressed in the majority of cells, suggesting that these kinases might be involved in melanoma progression. Treatment of cells with the CDK4 inhibitor palbociclib restricted cell proliferation to a similar, and in some cases greater, extent than MAPK inhibitors. Finally, we identified a low abundant sub-population in this sample that highly expressed a module containing ABC transporter ABCB5, surface markers CD271 and CD133, and multiple aldehyde dehydrogenases (ALDHs). Patient-derived cultures of the *BRAF* mutant/*NRAS* wild type and *BRAF* wild type/*NRAS* mutant metastases showed more homogeneous single-cell gene expression patterns with gene expression modules for proliferation and ABC transporters. Taken together, our results describe an intertumor and intratumor heterogeneity in melanoma short-term cultures which might be relevant for patient survival, and suggest promising targets for new treatment approaches in melanoma therapy.

## INTRODUCTION

Large-scale mutation analyses of malignant melanoma in the past identified mutations in the BRAF oncogene in 50% of melanoma samples [1, 2]. The most prevalent *BRAF* V600E missense mutation leads to an activation of the classical mitogen-activated protein kinase (MAPK) pathway. Targeted treatment of metastatic melanoma patients using small molecule inhibitors such as vemurafenib, dabrafenib and encorafenib directed against activated (mutated) BRAF kinase has shown promising results in recent years, significantly improving overall survival of affected patients [3]. However, a significant number of patients show primary resistance, and recurrences under inhibitor treatment occur as secondary resistance in the vast majority of cases. Recent studies have shown that combination treatments of BRAF and MEK1/2 inhibitors are significantly more effective than BRAF-inhibitor treatment alone [4]. However, 50% of patients develop a secondary resistance after 6–9 months [5].

There are a series of mechanisms described that underlie the secondary resistance of BRAF-mutant melanomas that occur after BRAF inhibitor treatment, including *NRAS* mutations, aberrant *BRAF* splicing, *BRAF* amplifications, *MAP2K1* (MEK1) mutations, *PTEN* and *PIK3CA* mutations, and *COT1* overexpression [6, 7]. In addition, mechanisms of primary treatment resistance of BRAF-mutant melanoma cells may be due to a MITF low/NF-κB high phenotype, which could be linked to a specific gene expression profile [8]. These results suggest that primary and secondary resistance mechanisms may be either due to genetic changes (mutations, amplifications) or changes in gene expression of specific pathways.

It has been suggested that recurrences and treatment failures may derive from intratumor heterogeneity [9]. That is, multiple subclonal mutations, gene expression patterns or epigenetic mechanisms may be present in tumor lesions and create a genetically heterogeneous population of tumor cells. Here, we analyzed the intratumoral heterogeneity in three short-term cultures derived from three different patients with metastatic malignant melanoma using single-cell RNA-seq. We used a comprehensive analysis and visualization strategy based on self-organizing maps (SOM) machine learning which is called ‘high-dimensional data portrayal’ because it visualizes the gene expression landscape of each individual cell. As a clustering method, SOMs offer several advantages compared with alternative methods such as non-negative matrix factorization, K-means, hierarchical clustering or correlation clustering [10]. By this means we identified gene expression patterns that may be useful for designing new treatments targeting tumor sub-populations.

## RESULTS

### Gene expression portraits of single-cell transcriptome heterogeneity in a *BRAF/NRAS* wild type melanoma sample

We applied microfluidic single-cell RNA-seq to measure the transcriptome of 92 single cells obtained from a *BRAF*/*NRAS* wild type melanoma short-term culture (Ma-Mel-123). In order to rule out intermixture of benign non-melanoma cells, we inferred largescale copy number variations (CNVs) from expression profiles by averaging gene expression over stretches of 50 genes on their respective chromosomes (Supplementary Figure S1). Data are shown as heatmap and revealed extensive copy number variations as a typical feature of cancer cells, basically ruling out an intermixture of benign cells such as fibroblasts.

For analysis of subpopulations, we used self-organizing map (SOM) machine learning which bundles a series of sophisticated downstream analysis tasks such as gene module selection, sample diversity clustering and functional knowledge discovery [11]. Its performance was previously demonstrated in different studies on cancer heterogeneity [12, 13].

**Figure 1:**
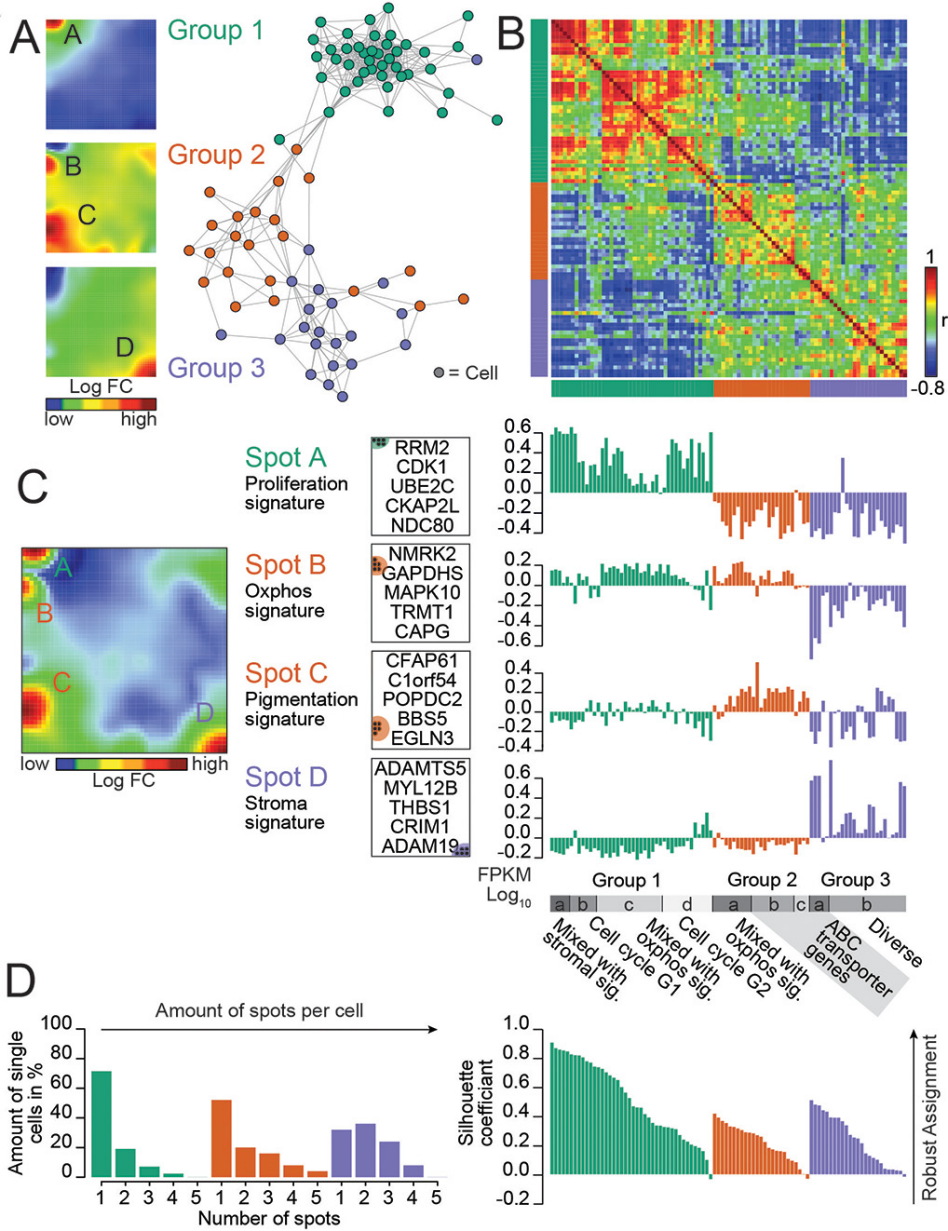
Dissecting heterogeneity in a *BRAF/NRAS* wild type patient-derived melanoma culture revealed by single-cell RNA-seq (**A**) A similarity network illustrates the diversity of single-cell expression landscapes. Three major groups of cells color-coded in green, red and purple are identified. Expression landscapes are visualized as mean SOM expression portraits of each group. Group-specific modules (A–D) of co-regulated genes are identified. (**B**) Pairwise correlation heatmap quantifies the mutual similarity of single-cell expression landscapes as Pearson's correlation coefficient (r). An anti-correlation for group 1 cells compared to cells of group 2 and 3 is discovered. (**C**) Mean SOM expression portrait of all cells illustrates the distribution of main expression spots across the SOM landscape. Spot-specific genes which could be assigned to different functional signatures (Proliferation, Oxphos, Pigmentation and Stroma) are identified. Top 5 genes of each spot defining the assignment to a functional group are shown for each spot. A refined analysis discovered subgroups (a–d) which are associated with different functions, e.g., different phases of cell cycle and ABC-membrane transporters. (**D**) Number of over-expression spots per cell increases from group 1 to group 3 revealed by the spot number distribution. Quality of cluster assignment is demonstrated by a silhouette plot. Genbank database accession number: GSE81383.

SOM classified the cells into three major groups as proliferation, pigmentation and stromal type (Figure 1A; Supplementary Figure S2) according to the major gene categories represented in each group. The majority of the 92 cells (*n* = 42) were defined by genes involved in processes of cellular proliferation such as DNA replication, DNA repair, chromosome segregation and mitosis [14]. The pairwise correlation map shows that the expression landscapes of group 1 virtually anti-correlates with those of groups 2 and 3 (Figure 1B). We identified four main clusters of co-expressed genes which were called spot-modules A–D (Figure 1C, 1D; Table 1; Supplementary Table S1).

**Table 1:** Overview of gene sets identified in different groups of cells as defined by SOM analysis of single-cell sequencing data

Spot module	Group^a)^			Name	Enriched gene sets^b)^	Top 10 genes^c)^	Kinome^d)^	MMIC^d)^	MKG^d)^
	1	2	3						
**A**	+	−	−	Proliferation	Cell cycle, cell division, DNA repair, G1/S transition of mitotic cell cycle, telomere maintenance (BP), Nuytten_EZH2-targets_DN, E2F targets (HM)	TOP2A, ASF1B, RRM2, HMGB2, SPC24, UBE2T, KIAA0101, CDK1, RAD51AP1, PRC1	CDK1, NEK2, PLK1, AUKB1, PBK		EZH2
**B**	+	+	−	Oxphos	Mitochondrion (CC), Oxidative phosphorylation (HM), NAD-binding (MF), DAIRKEE_TERT_TARGETS_UP	ARHGAP8, TRPM1, CDK2, MLANA, CHCHD6, FAM207A, NMRK2, ASAH1, TYR, SVIP	CLK3	ABCB5, (CD279)	MITF, IDH1, TP53
**C**	−	+	−	Pigmentation	Melanocyte-differentiation (BP), melanocome (CC), pigmentation (BP), cell differentiation (BP)	CCDC171, GPR143, RAB27A, TBC1D16, SOCS6, DSTYK, VEPH1, ATP5B, CDH3, SNAI2	MET, MOK, KDR, ACTR2	CD133	
**D**	−	−	+	Stroma	Epithelial-mesenchymal-transition (HM), Lenz_stromal-signature, Pasini_Suz12_targets_DN, Nuytten_EZH2-targets_UP, focal adhesion (CC), Wong_adult-tissue-stem-module, Lim_mammary-stem-cell_UP, Wu_cell-migration, Naba_matrisome, Wang_Smarce1_targets_up	CRIM1, ADAMTS5, MYL12B, THBS1, ANXA1, NAV3, PZP, NTN4, FRMD6, ITGA1	SRPK2, RAF1, TDFBR1		HRAS, RAF1
**E**^e)^	about 10% of all cells			ABC-trans-porters	ABC-transporters (KEGG), ATPase activity coupled to transmembrane transport (MF), adenylate cyclase activating G-protein receptor signaling (MF), G alpha signaling events (reactome), bile acid metabolism (HM), aldo-keto reductase (NAD), protein targeting to GOLGI (CC), PID_endothelin_pathway, signaling by EGFR in cancer (reactome)	**ABC-transporters:** ABC-A9, B4, C4, D1, C1, A2, G5, C9, A1, A6, G1, A13, C11, A3, C6, C3, Aldehyde **dehydrogenases:** ALDH-5A1, 16A1, 3A1, 1L2, 8A1, 2, 1A3, 4A1	KIT	ABCG2, ABCG4	BRAF, NRAS, CDKN2A

a) up(+)/down(−) regulated in group.

b) Gene sets refer to the categories assigned in the methodical part. Only sets with *p* values < 10–7 (Fishers exact test) were taken into account. Literature sets were taken from Ref. 31,62–68.

c) Genes are ranked with decreasing correlation coefficient with respect to mean spot expression profile; full gene lists for each spot are provided as Supplementary Tables (Supplementary Tables S1, S2). ABC transporter and aldehyde dehydrgenases were grouped together.

d) Kinases are selected using the criteria‚ high and almost invariant expression as described in the Supplementary material. MMIC markers were taken from independent studies (summarized in [23]). Melanoma key genes (MKG) collect genes directly related to melanoma.

e) Cells expressing the ABC-transporter signature are observed in all three groups.Abbreviations: BP, Ben-Porath et al., 2008.

Spot A, upregulated in cells of group 1, is enriched with genes related to cell cycle and cellular proliferation (Figure 1C). Spot B is enriched with genes involved in energy metabolism and oxidative phosphorylation (Oxphos) which are concertedly upregulated in cells of group 1 and group 2. Spot C is enriched with genes related to pigmentation and melanocyte differentiation. Spot D is enriched with genes related to tumor stroma. For further details, see also Supplementary Figures S2, S3. A closer inspection of the single-cell expression portraits in the different groups revealed a fine structure (Figure 1C, Supplementary Figure S4). E.g., expression portraits of group 1 can be separated into cells showing characteristic patterns of the G2 phase of cell cycle (subgroup SG1a, 13 cells) and G1S-phase (SG1c, 7 cells).

To obtain a more detailed view on the biological context we mapped selected gene sets of different categories into the SOM and characterized them in terms of gene set-maps and GSZ-profiles (Supplementary Figure S5). Interestingly, genes from G1 and G2 phase of the cell cycle accumulate in close proximity (Supplementary Figure S5A). The gene sets oxydative phosphorylation (Oxphos) and melanocyte differentiation accumulate in spots B and C (Supplementary Figure S5B). In addition to the stromal signature in group 3 cells, we identified a cell population with enriched ABC-cassette membrane transporters which is described in detail below (Supplementary Figure S5C).

### Marker genes of cellular subpopulations show similar expression patterns in the original *NRAS/BRAF* wild type tumor tissue

In order to demonstrate that different cellular subpopulations identified in the single-cell analysis are also present in the original tumor sample, immunofluorescence stainings were performed of the original frozen tumor material (Ma-Mel-123 tissue). Antibodies used were directed against proliferation markers TOP2A and Ki-67 (spot module Proliferation; Table 1 and Supplementary Table S1), ITGA1 (spot module Stroma; Table 1; Supplementary Table S1), and CDK4 (highly expressed in all cells). As shown in Supplementary Figure S6, the mentioned proliferation markers showed disseminated staining of individual cells within the tumor tissue with 30–40% of positively staining tumor cells. Staining of ITGA1 showed a lower percentage of staining of 15–20% of individual cells but also stained endothelial cells lining microvasculature. CDK4 expression was found in the cytoplasm and nucleus and was homogeneously distributed over the whole sample (Supplementary Figure S6). These findings are in agreement with the percentages of cell subpopulations of the single-cell analysis (45% of cells with the proliferation signature; 27% of cells with the stromal signature). Thus, by analysis of a selected set of marker proteins we could validate the single-cell data *in vivo*, which argues for a preservation of cell subpopulations in tumor tissues in tumor-derived short-term cultures.

### An independent single-cell RNA-seq study on different melanoma tissues confirms cellular subpopulations found in a melanoma short-term culture

Gene sets as determined in a recent single-cell transcriptome analysis on 19 melanoma tissues were mapped into our single-cell RNAseq data to study whether expression characteristics of these experiments may be identified in our data sets [15]. As shown in Supplementary Figure S7, the heatmap of the GSZ score of the different melanoma gene sets of the mentioned study revealed strong correlations with our data in terms of spot enrichment, expression profiles and group assignments. The group of proliferative cells (group 1 of our study) showed high expression of cell cycle signature genes (principal component 2 (PC2)) from the study of Tirosh and co-workers [15] (Supplementary Figure S7A). Group 2 cells of our study show high expression of MITF- and melanoma-signature genes (PC4 of the study of Tirosh and co-workers) [15]. AXL signature genes were highly expressed in our group 3 (stromal genes). The right part of the Supplementary Figure S7A shows selected GSZ-profiles and gene set maps which illustrate accumulation of the genes in spots A–D in a set-specific manner showing that the melanoma cells studied by us and by Tirosh and co-workers show similar activation patterns of different cellular programs. Moreover, cells with an activated KDM5B (JARID1B) signature refer to group 2 or 3 of our study and thus to low cycling cells in agreement with their study [15]. AXL high expression co-regulates with our stromal signature (Supplementary Figure S7A). Slow-cycling cells of our study divided into two populations according to the activity of MITF- and AXL-programs (Supplementary Figure S7C, S7D). A similar division was also shown for individual samples in the study of Tirosh and co-workers [15]. Overall, gene sets referring to PC1 to PC5 of the study by Tirosh and co-workers agree with our spot and group characteristics and thus support our classification scheme (Supplementary Figure S7B, S7C). Taken together, the cellular subpopulations in the present study show large overlaps with the mentioned study on gene signatures active *in vivo*.

### Single-cell transcriptome data reflect prognostic expression signatures of melanomas *in situ*

**Figure 2:**
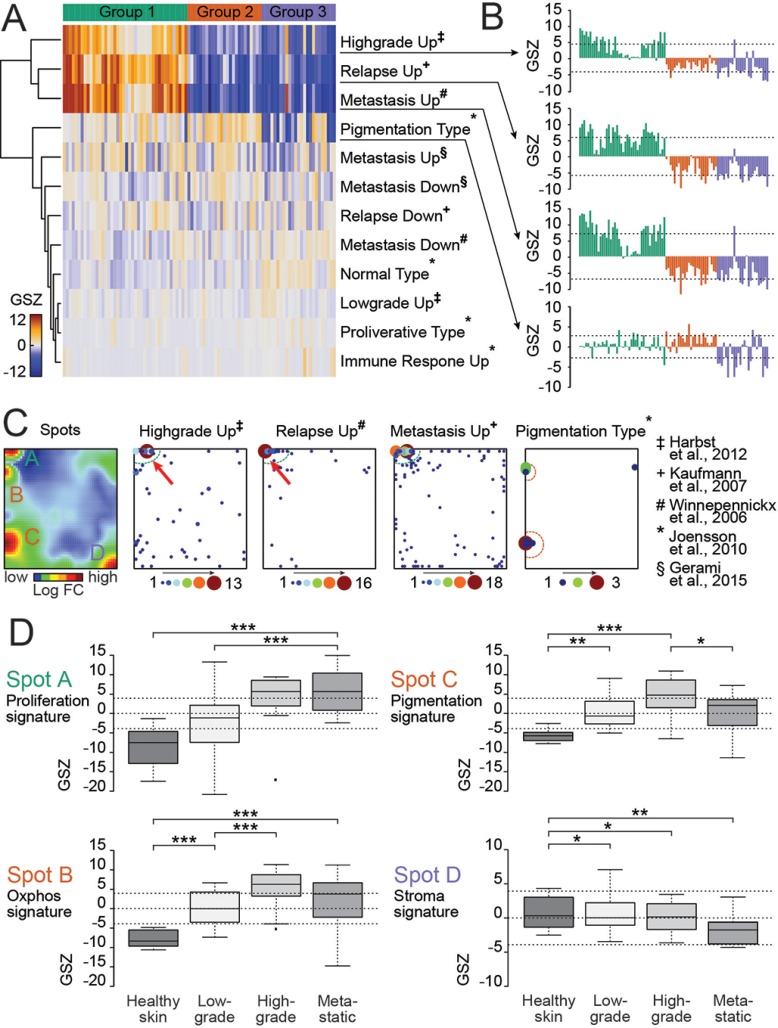
Patient-derived *BRAF/NRAS* wild type melanoma culture resembles partially primary and metastatic melanoma gene expression signatures (**A**) Heatmap showing correlation of independent studies [16–20] and our gene expression modules detected by single-cell RNAseq. Four of the tested gene expression data sets (Highgrade Up, Relapse Up, Metastasis Up and Pigmentation Type) show upregulation in cells of either group 1 or 2. Each column represents a single cell. (**B**) GSZ-profiles of identified gene sets illustrate the proportion of their similarity with single cells of our study. Each bar represents a single cell. (**C**) Selected gene sets are mapped on the SOM portrait to identify additional substructures. A distinct expression of genes between the Highgrade Up and the Relapse Up type can be recognized within spot A (cell cycle signature) indicated by red arrows. (**D**) Comparison of single-cell expression data from the patient-derived melanoma culture with melanoma expression data of an independent study by mapping top 100 genes of each expression spot (A–D) against melanoma data by Raskin and co-workers [21]. Cell cycle genes (spot A) are upregulated in metastatic melanoma and in high-grade melanomas.

Gene expression patterns in the spot modules of the present study were compared with prognostic gene patterns observed in melanoma samples of independent studies [16–20] (Figure 2). In the study of Jönsson and co-workers on metastatic lesions, the proliferative subtype had the shortest overall survival compared to all other groups [16]. In the study of Harbst and co-workers on primary melanomas the gene signatures of proliferation and pigmentation combined defined a high-grade class of melanomas with poorer overall survival compared to other subgroups [17]. The study of Kaufmann and co-workers and Winnepenninckx and co-workers found different gene signatures in patient material of patients with metastasis (bad prognosis) or no metastasis in a 4-year follow-up period [18, 19]. The study by Gerami and co-workers provided a meta-analysis of the mentioned studies [20]. Each of the signature gene sets associated with poor prognosis were strongly upregulated in cells of either group 1 or 2 (Figure 2A, 2B). Group 1 genes are upregulated in early relapse melanomas, highly metastatic and high-grade melanomas. Interestingly, the gene set maps reveal that the signature sets shift with respect to their cell cycle characteristics from the G2 to the G1S pattern between the different prognostic melanoma types (Figure 2C). These findings show that single-cell gene expression patterns from an *in vitro* analysis may identify cell subsets with impact on the patients' prognosis.

Expression of top 100 genes from each spot-cluster (A–D) was analyzed in a further independent data set [21] (Figure 2D). Our cell signatures of spot B (Oxphos) and C (Pigmentation) show maximum expression in high grade primary melanomas whereas spot signature A (Proliferation) shows strongest expression in high-grade and metastatic melanomas (Figure 2D). Spot D (Stroma) shows downregulation during tumor progression [21]. Hence, high similarities of gene activity of our proliferation signature were found in high grade primary and metastatic tumors.

### Mapping of the kinome identifies signaling kinases as potential targets for targeted treatment of the *BRAF/NRAS* wild type melanoma

**Figure 3:**
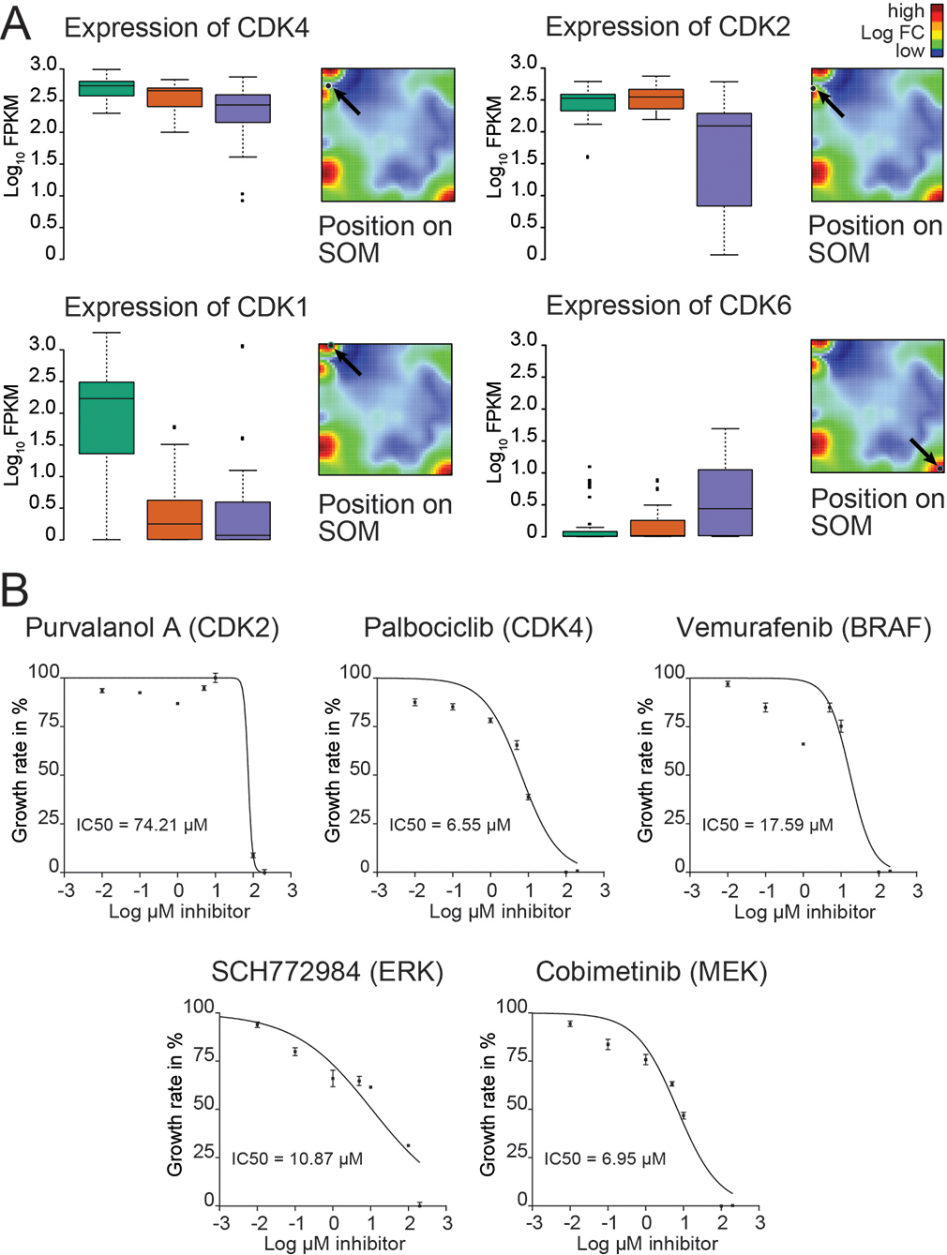
Kinome analysis of the *BRAF/NRAS* wild type culture reveals potential targets for clinical treatments (**A**) Gene expression levels of 4 cyclin-dependent kinases (CDK1, CDK2, CDK4 and CDK6) are analyzed for each of the 3 identified main groups. Data are given as standard box plots. CDK2 and CDK4 are consistently highly expressed in all groups. Positions of each CDK gene are shown on the mean SOM expression landscape indicated by arrows. (**B**) Growth inhibition curves are shown for treatments of a patient-derived melanoma short-term culture using inhibitors directed against CDK2 (purvanolol), CDK4 palbociclib, activated BRAF (vemurafenib), ERK1/2 (SCH772984), and MEK1/2 (cobimetinib).

We next explored the cellular kinome heterogeneity across the cell groups [22]. Genes of different kinase families were mapped to the different spot-modules (Supplementary Figure S8). Most of the candidate genes identified were co-expressed with group-specific spot signatures/modules (Table 1). Interestingly, the cell cycle-dependent kinases CDK4 and CDK2 were consistently highly expressed in the vast majority of cells analyzed with CDK4 showing the most prominent expression in the *BRAF/NRAS* wild type culture (Figure 3A; Supplementary Figure S8). In addition, KIT was found to be among the top ranked kinases (Supplementary Figure S8). CDK4 was also part of the PC1, and CDK2 was part of the MITF and melanoma signature in the study of Tirosh and co-workers which is suggestive for a role of both kinases in this study [15].

### CDK4 inhibitor palbociclib reduces melanoma cell proliferation

To test the impact of CDK4 expression on treatment response of the *BRAF/NRAS* wild type melanoma cells, different small molecule inhibitors were used targeting MAPK, CDK2 and CDK4 pathways. After 72 h of exposure, CDK4 inhibitor palbociclib reduced melanoma cell proliferation of Ma-Mel-123 cells with significantly higher efficiency than BRAF inhibitor vemurafenib (*p* = 0.0017) (Figure 3B). The activity of MEK1/2 inhibitor cobimetinib was comparable to that of palbociclib (*p* = 0.80). Significant higher activity was also obtained for palbociclib in comparison to CDK2 inhibitor purvanolol but not against ERK1/2 inhibitor SCH772984 (*p* = 0.14).

In addition, the melanoma short-term culture carrying an activating *NRAS* mutation (*NRAS* G13R) (Ma-Mel-93) was used (data not shown). No higher activity was found for palbociclib compared to vemurafenib (IC50=10.19 vs IC50=15.93, *p* = 0.08) and ERK1/2 inhibitor SCH772984 (IC50 = 15.66; *p* = 0.06). MEK1/2 inhibitor cobimetinib showed significantly higher activity than palbociclib (IC50 = 7.30 μM; *p <* 0.0001).

Control experiments were carried out with benign fibroblasts (48 h). The IC50 values of palbociclib for Ma-Mel-123 cells after 48 h were 10.05 μM and for fibroblasts 17.57 μM (*p* = 0.49). In summary, CDK4 inhibitor palbociclib may be a strong inhibitor of melanoma cell proliferation in the absence of *BRAF* and *NRAS* mutations in melanomas with homogeneous CDK4 expression.

### MMIC signatures associate with melanoma single-cell expression

**Figure 4:**
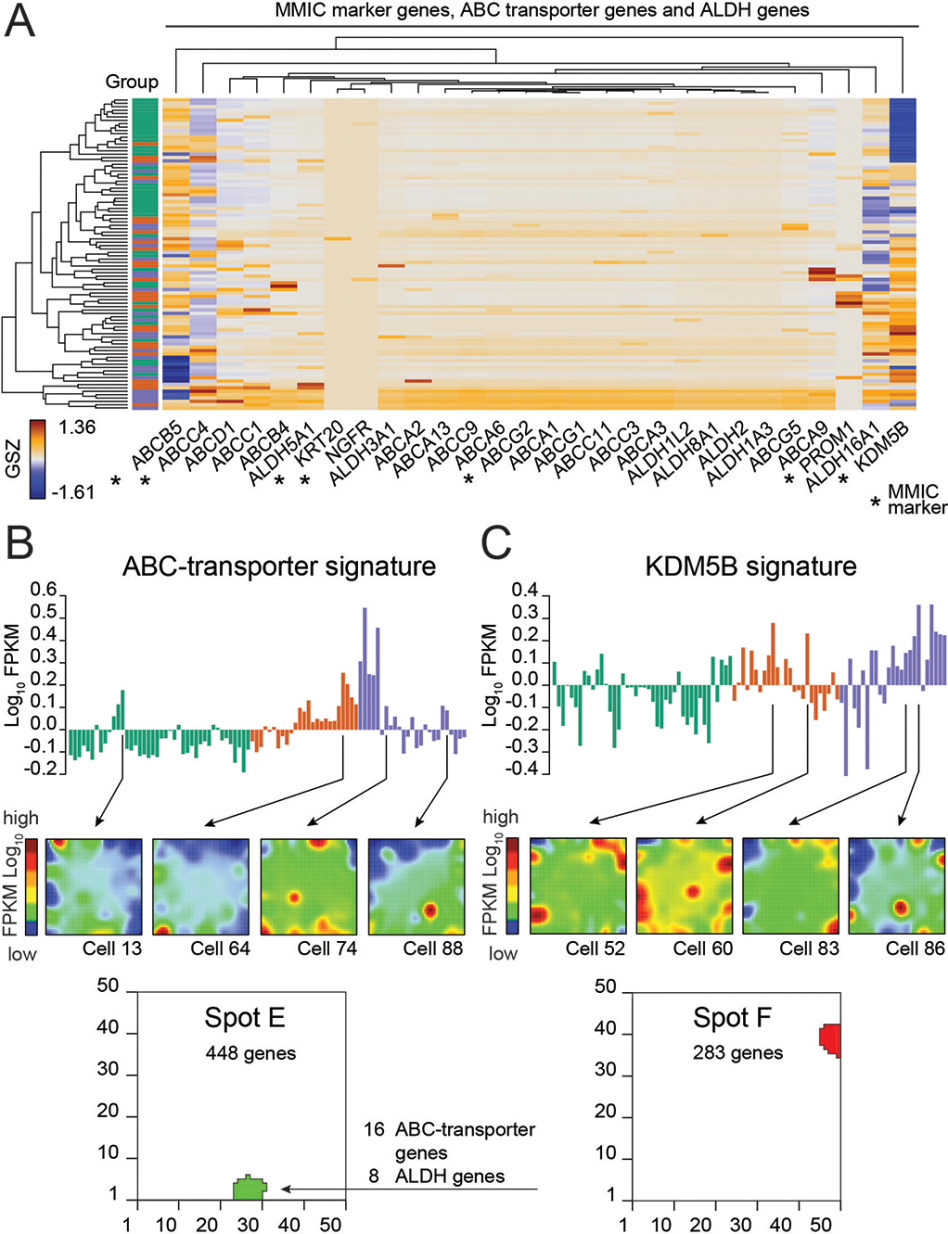
Expression of marker genes of melanoma stem or initiating cells (MMIC) (**A**) Heatmap showing scaled expression of MMIC marker genes [23] (e.g., ABCB5, KDM5B and ABCC4), various ABC-transporters and ALDH genes. Additional ABC-transporters and ALDH genes shown are all co-expressed in spot E (Supplementary Figure S5). Rows represent single cells. (**B**) Expression profile of spot E comprising 16 ABC-transporter and 8 ALDH genes. Selected expression portraits of cells expressing these genes are shown. (**C**) Expression profile of spot F where *KDM5B* (JARID1B) is heterogeneously expressed across single cells analyzed. Selected expression portraits of cells expressing *KDM5B* (JARID1B) are shown.

Next we searched for a subpopulation of malignant melanoma stem and initiating cells (MMIC) using a set of MMIC marker genes [23] (Figure 4A 4B; Table 1; Supplementary Figure S9A). MMIC markers of the ATP-binding cassette (ABC) transporter family are expressed in a large number of cells (Figure 4A). Interestingly, two of these genes (*ABCG2* and *ABCG4*) co-express together with 16 other members of the ABC transporter family and with 8 aldehyde dehydrogenases (ALDHs) in about 10% of all cells of the *BRAF*/*NRAS* wild type cells (Figure 4B, additional Spot E; Table 1; Supplementary Table S2). The co-expression of ABC-transporters with ALDHs supports their possible role in resistance against chemotherapy and tumor stem cell activity [23–25]. Mapping of this signature into the melanoma data set of Raskin and co-workers shows activation in early stages of melanoma progression (Supplementary Figure S9B) [21].

Another MMIC-marker is *KDM5B* (*JARID1B*), a demethylase of H3K4me3. *KDM5B* (*JARID1B*) is often found in association with weakly transcribed genes which demethylate their gene bodies during embryonic stem cell self-renewal [26–28]. In melanoma, *KDM5B* (*JARID1B*) is regarded as a marker of slow-cycling melanoma cells which are essential for long-term tumor growth [29] Our *KDM5B* (*JARID1B*) signature clearly reflects decreased gene activity in group 1 cells (Proliferation) (Figure 4C). *KDM5B (JARID1B)*-positive cells are assumed to form a side population that displays characteristics of enriched chemoresistance and tumorigenesis [30]. About 7% of our cells mainly of group 3 overexpress this *KDM5B*-signature (Figure 4C).

Taken together, cells which could possibly be candidates for MMICs are those with the *ABC* transporter and *ALDH* gene expression signatures and those with *KDM5B* expression.

### Modes of epigenetic regulation

Next, we analyzed a series of embryonic stem cell (ESC) and neural progenitor cell gene expression signatures characterizing poorly differentiated tumors [31–33] and signatures of 15 chromatin states in fibroblasts, melanocytes (MCs) and neural progenitor cells (NPCs) [34] (Supplementary Figure S10). So-called poised and repressed genes are regulators for fate decisions of embryonic stem cells ensuring their pluripotency. We find that the expression signatures of poised and repressed genes in ESCs, MCs, NPCs and partly also in fibroblasts show similar profiles as the ABC- and the KDM5B-signatures identified above (Figure 4, Supplementary Figures S10, S11).

Deregulation of these states due to epigenetic mechanisms is associated with cellular reprogramming and cancer stemness [35, 36]. Changes of expression-inactive chromatin states (poised, repressed and silent chromatin) were especially observed for the ABC-transporter and *KDM5B*-positive cells.

Finally, we systematically studied the gene expression levels of more than fifty chromatin modifying enzymes in the single cell data (Supplementary Figure S12). Cells of the stromal type (group 3) show a high activity level of enzymes that remove methylation marks from DNA and histone lysine side chains. Among them is a high fraction of demethylases of H3K9me3. This result together with the finding that the DNA demethylases TET1 and TET3 are upregulated in ABC-transporter type cells suggests a deregulation of the DNA-methylation machinery with consequences for DNA-methylation. In summary, we find that epigenetic modes of transcriptional regulation such as chromatin remodeling, DNA methylation changes and modulation of the expression of chromatin modifying enzymes are potential regulators of different functional states in melanoma.

### Single-cell transcriptome patterns of *BRAF* mutant/*NRAS* wild type and *BRAF* wild type/*NRAS* mutant short-term cultures

**Figure 5:**
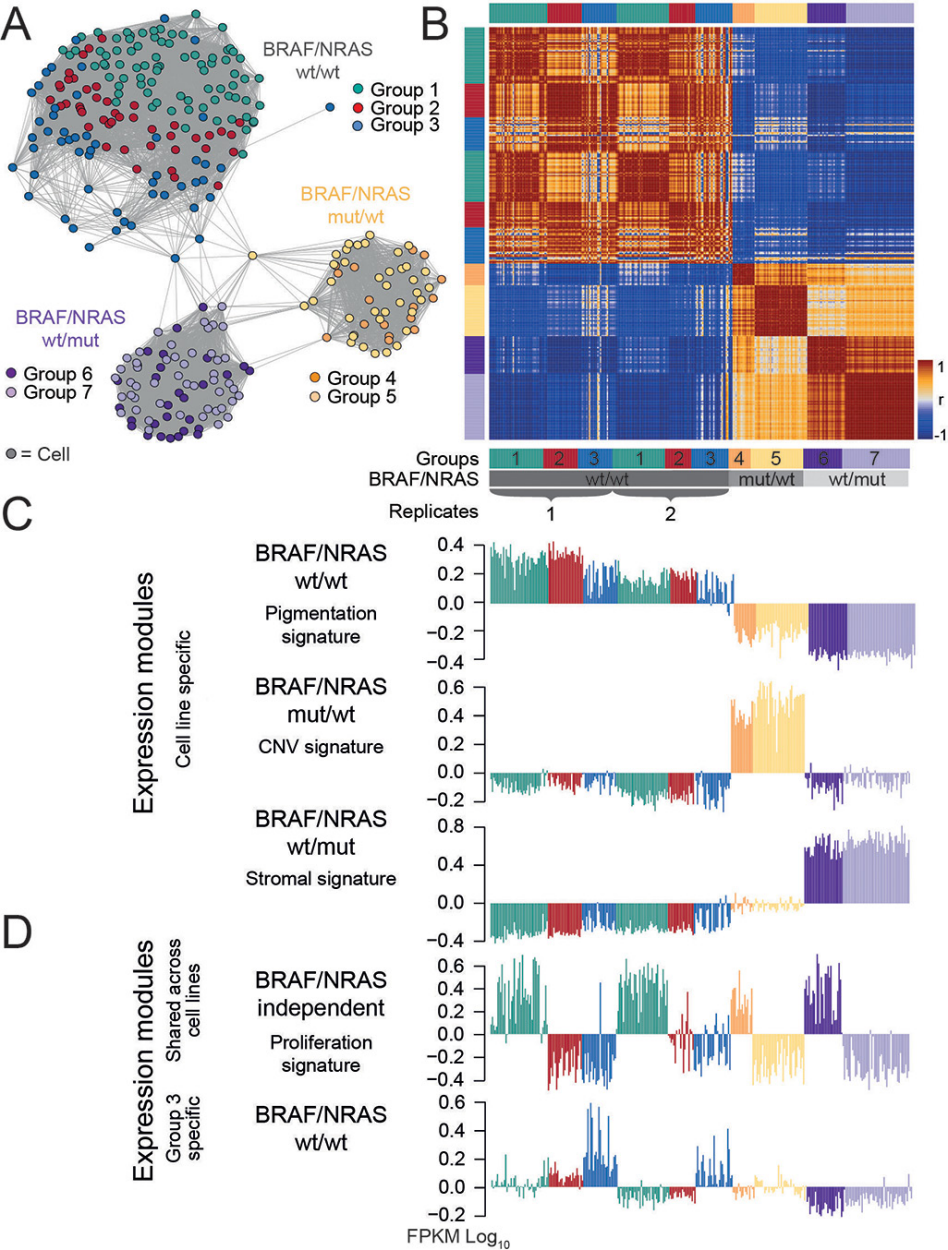
Comparative analysis reveals intertumor heterogeneity (**A**) A similarity network was calculated and reveals separate clusters of *BRAF/NRAS* wild type (wt/wt), *BRAF* mutant/*NRAS* wild type (mut/wt) and *BRAF* wild type/*NRAS* mutant (wt/mut) cells, respectively. A replicate analysis of wt/wt cells was performed and fits into the respective wt/wt cluster. Cells in a highly proliferative state were highlighted in each cluster. (**B**) Pairwise correlation heatmap quantifies the mutual similarity of single-cell expression landscapes as Pearson's correlation coefficient (r). (**C**) Comparative SOM analysis between the different cultures generated five main expression signatures. Three expression signatures characterize each individual cell culture which represents intertumor heterogeneity. (**D**) Integrated proliferation signature which is shared between all cell cultures analyzed and group-specific expression signature for group 3 cells of the wild type short-term culture. Genbank database accession number: GSE81383.

In order to further substantiate our findings, a SOM analysis of an experimental replicate of the *BRAF/NRAS* wild type culture was performed, together with a t-distributed stochastic neighbour embedding (tSNE) analysis. As shown in Figure 5A, 5B and Supplementary Figure S13A, the cells of the replicate analysis clustered together with those of the first analysis revealing no major transcriptional differences between independent experiments of the same short-term-culture after an additional passaging. The cells showed similar percentages of cells within each group (group 1: 46% vs 45%; group 2: 27% vs 23%; group 3: 27% vs 32%) (Figure 5B; Supplementary Figure S13; Table 2; Supplementary Table S3).

**Table 2:** Comparison on single-cell gene expression signatures between different cell cultures

Upregulated in culture^a)^	Groups	Enriched gene sets	Signature^a)^	Top 10 genes
BRAF/NRAS wt	1–3	Oxphos (HM), mitochondrion (CC), melanocyte differentiation (BP), melanosome (CC), Tirosh_MITF-signature, Joensson_pigmentation-subtype	Oxphos (spot B) and pigmentation (C)	PMEL, GSTP1, GPR143, MBP, SLC45A2, TRPM1, CDK2, NARS2, GPR161, KDR, TYRP1
BRAF/NRAS wt	3	DaCosta_UV-response-via-ECCR3_DN, WANG_SMARCE1-targets_UP, Reactome_PolII-promoter-opening	Stroma (spot D)^b)^	VTN, PZP, HIST2H4B, POTEI, HIST2H4A, HIST2H2AA4, SPTA1, A2M, LOC102724334, PCDH20
BRAF mut/NRAS wt	4, 5	Chr. 11, Chr. 8, Chr. 5, Chr. 19, DaCosta_UV-response-via-ECCR3_UP, mitochondrial-inner-membrane (CC), extracellular exosome (CC), Liang_hematopoesis, TSSA_melanocytes (active chromosomal states)	Aberrant copy number	CD36, CBR1, NELL1, SNX10, HTR2B, PLCD1, PRDX2, CFI, BCAS3, MOXD1
BRAF wt/NRAS mut	6, 7	Epithelial-mesenchymal transition (HM), Wu_cell-migration, Pasini_SUZ12-targets_DN, Lenz_Stromal-signature, extracellular-matrix-organization (BP); Tirosh_AXL-signature	Stroma (spot D)^b)^	AXL, CDH13, CD74, HLA-DRA, CSAG1, SERPINB2, TGFBI, THBS1, GAGE1, HLA-DRB1
All cell cultures: Gr. 1 (45%), Gr. 4 (30%), Gr. 6 (36%)	1, 4, 6	BenPorath_cycling-genes, cell-division (BP), DNA-replication (BP); Tirosh_core-cycling-genes	Proliferation (spot A)	HMGB2, CDK1, TOP2A, NUSAP1, MAD2L1, NCAPG, RRM2, UBE2C, PRC1, SKA1

a) Enriched gene sets and gene signatures refer to comparisons between different cell cultures. The single-cell gene expression signatures of the indicated culture are compared to those of the two other cell cultures. Literature sets were taken from Ref. 15, 31 64, 68–71.

b) The stromal (spot D) signature identified in wt/wt cells splits into two groups of genes, one being specifically activated in group 3 cells of the wt/wt-cell culture only, and one common for the wt/wt and wt/mut cell culture.

Furthermore, two short-term cultures of two additional patients were analyzed (Figure 5). These two cultures consisted of *BRAF* mutant/*NRAS* wild type and *BRAF* wild type/*NRAS* mutant cells, respectively. As shown in Figure 5A, 5B and Supplementary Figure S13, the *BRAF* and *NRAS* mutant cells produced clusters of cells which well separated from the *BRAF*/*NRAS* wild type cells due to pronounced changes of their gene expression patterns (Figure 5; Supplementary Figure S13). Joint SOM analysis of all three different cell cultures provided signatures of genes which were specifically up-regulated in each of the cell cultures and thus separated the three different cultures (Figure 5C; Table 2). *BRAF/NRAS* wild type cells were characterized by a gene signature which combines the oxphos and pigmentation signatures. A subset of signature genes of the stromal cells found in the wild type short term culture (Group 3) was also uniquely expressed in these cells (Figure 5D). In comparison to both others, the expression signature of *BRAF* mutant/*NRAS* wild type cells was governed by large-scale copy number variations evident in chromosomal mapping of the expression data (Figure 5C, Table 2; Supplementary Figure S1). The *BRAF* wild type/*NRAS* mutant cells predominantly up-regulated genes associated with the stromal signature in a functional context different to the one found in the wildtype short-term culture (Table 2; Supplementary Figure S13). An overlapping gene expression signature between different cultures was found for genes involved in cellular proliferation. A fraction of 30% to 45% (45% in *BRAF* wt/*NRAS* wt, 30% in *BRAF* mut/*NRAS* wt and 36% in *BRAF* wt/NRAS mut) of cells from each culture was highly proliferative and showed a common proliferation signature independent of their culture-specific expression patterns (Figure 5D). This common proliferative signature was almost identical between the mutant cells and double wild type cells and also with the cell cycle signatures described by Tirosh and co-workers [15]. Additionally, it provided expression profiles that closely resembled those of metastatic, relapsed and high-grade melanomas (Figure 2A, Supplementary Figure S13). Other subpopulations found in the primary analysis of the *BRAF/NRAS* wild type cells could also be recapitulated in the mutant cell lines. About 20–40% of cells in the mutant cell cultures co-expressed sets of ABC transporters together with aldehyde dehydrogenases (ALDHs) (Supplementary Figure S13; Supplementary Table S3).

The single-cell study of Tirosh and co-workers detected an antagonism of MITF- and AXL-related transcriptional programs. In the present analysis, *NRAS*-mutant cells activated the AXL program and *BRAF*/*NRAS* wild type cells the MITF program which also resembles the melanoma pigmentation subtype described earlier [16] (Supplementary Figure S7D; Supplementary Figure S13). Detailed analysis of the *BRAF*/*NRAS* wild type data revealed differential activation of the MITF- and AXL-programs in groups 2 and 3 of our data (Supplementary Figure S7).

Taken together, we could show that the same expression profiles were identified when performing replicate experiments on the same short-term culture. However, cell cultures of different patients showed distinct gene expression patterns.

## DISCUSSION

Intratumor heterogeneity based on a complex subclonal molecular structure in malignant tumors has been regarded as a major factor that influences therapeutic response and secondary treatment resistance [9, 37]. In order to further estimate intratumor heterogeneity and the subclonal structure of malignant tumors, single-cell sequencing has been used for the analysis of a series of tumor cell lines, solid tumors and haematological malignancies in recent years [37–39]. In the present study, three different patient-derived melanoma cell cultures were analyzed. Intercellular (intratumor) heterogeneity was largely driven by genes involved in proliferation, stroma and MITF/AXL programs. The proliferation signature showed strong overlaps between the different cultures.

It is known that cell cycle control, cellular proliferation and oncogenic signaling are largely dependent on intracellular mechanisms such as kinase-directed signaling. We therefore more closely investigated the differential expression of the cellular kinome in the *BRAF/NRAS* wild type culture [22]. Among the most differentially expressed kinases and kinase-related genes are CDK1, NEK2, CLK3, MET, MOK, KDR, RAF1 and KIT. Interestingly, CDK2 and CDK4 were the only kinases that were almost homogeneously expressed in the vast majority of single cells, with CDK4 being more prominent than CDK2. Similar findings regarding kinases and kinase-related genes have been reported by several other studies [38–42].

The identified gene expression pattern was highly suggestive for a targeted treatment approach directed against CDK4 and potentially also CDK2, which were the only signaling kinases expressed in the vast majority of cells. Interestingly, in a recent study, the cyclinD-CDK4/6 axis has been emphasized as an interesting target for tumor treatment [43]. Indeed, CDK4 inhibitor palbociclib was more effective than BRAF inhibitor vemurafenib for treatment of the bulk short-term culture. This finding is not surprising as the short-term culture is derived from a BRAF wild type tumor, which is known to not respond to BRAF inhibition [3]. However palbociclib activity equaled the activity of MEK1/2 and ERK1/2 inhibitors in this culture. MEK1/2 inhibitors such as trametinib have been shown to be active in BRAF/NRAS wild type melanomas and induced at least a partial response in 10% of melanoma patients [44]. Although we observed some effects of palbociclib on fibroblasts which might argue against its widespread clinical use, palbociclib is well tolerated in treatment of other tumor entities [45].

Despite the dominating proliferation signature found in all three cultures, also other functional groups such as a pigmentation and a stromal signature were identified in the double wild type melanoma short-term culture. This raised the question if the heterogeneity of patient-derived short-term cultures revealed by single-cell RNA-seq might reflect in part different gene expression patterns found in primary or metastatic melanomas. In a direct comparison of the single-cell data and an immunofluorescence analysis of the original tumor of the *BRAF/NRAS* wild type tumor sample, we provided strong evidence that the cell composition revealed by single-cell RNA-seq reflects the actual situation in the original primary tissue. Moreover, some gene expression signatures found in our single-cell data overlapped with patterns of prognostic relevance in a series of independent studies on melanoma patients [16–19]. Genes associated with high proliferative activity and genes of the pigmentation signature were found in the gene signatures related to negative patients' prognosis [16, 17, 19]. Moreover, the proliferation signature of our study was also highly expressed in high-grade and metastatic melanomas in another independent study [21]. Notably, a recent single-cell analysis of 114 selected genes of invasive and proliferative signatures in two melanoma cell lines with different metastatic capacity is in line with our results as the single-cell gene expression differed significantly between both cell lines [46]. Thus, *in vitro* gene signatures may at least in part predict *in vivo* behavior of melanoma cells.

In a more recent study, and in accordance with our data, a significant intertumor heterogeneity was observed in a single-cell RNA-seq analysis of 19 melanoma tissues [15]. Major subgroups of transcriptional heterogeneity were associated with cell cycle, spatial context of cells, and a drug-resistance program (MITF low/AXL high signature). The majority of the different gene signatures of tumor cells of the study of Tirosh and co-workers could be mapped to our cell subpopulations, arguing for a conservation of *in vivo* gene signatures in *in vitro* cultures at least in those with low passage numbers [15]. Among these overlapping gene signatures were the proliferation signatures and the MITF/AXL signatures. Furthermore, the *KDM5B* (*JARID1B*) signature in this study showed a strong association with non-cycling cells in our study. It is noteworthy that *KDM5B* (*JARID1B*) is a known marker of slow-cycling cells [29]. Together, these findings supported the notion that *in vivo* gene signatures are conserved in short-term cultures.

Recently, single-cell analyses have been used to identify candidates for tumor stem cells [38, 40]. Similar to these studies, we analyzed a set of genes recently described as markers for malignant melanoma stem or initiating cells (MMIC) [23]. No single stem-like subpopulation could be defined by the presence of all these genes. However, a subpopulation of about 10% of cells was defined by an increased expression of ABC transporter and *ALDH* genes in all three cultures. Overexpression of ABC transporters is found in cancer stem cells of different tumors and, similarly, *ALDH* genes have been described as a characteristic feature of cancer stem cells [47]. Thus, the ABC/ALDH subgroup presents interesting gene candidates for MMICs in our study [23–25]. In addition, our analyses of epigenetic marks of gene expression further favor the presence of a small subpopulation of cells with stemness character. Genes poised by histone marks and by polycomb genes, both hallmarks of stemness, were found in our ABC transporter and ALDH subpopulation.

Interestingly, another small group of cells concordantly expressed *KDM5B* (*JARID1B*), a gene known to be involved in epigenetic regulation of gene expression. As mentioned above, a *KDM5B* (*JARID1B*) signature has also been described in the study of Tirosh and co-workers [15]. In accordance with this latter study, we could show that *KDM5B* is associated with non-proliferating cells, which is consistent with other previous studies where it was shown to be a marker for slow-cycling melanoma cells as well as involved in long-term melanoma growth [15, 24]. Strikingly, *KDM5B* was also identified by Patel and co-workers to be significantly higher expressed in non-cycling glioblastoma cells [38]. Since it is known that stem-like cells divide with lowest rates, cells of the identified KDM5B subgroup are interesting candidates for stem-like melanoma cells [29, 48].

Taken together, we identified marked intertumor and intratumor (intercellular) heterogeneity in three different patient-derived melanoma cultures including a small subpopulation of stem-like cells. Specifically targeting these stem-like cells or cells with negative impact on the patients' prognosis may in the future provide promising treatment strategies.

## MATERIALS AND METHODS

### Melanoma cell culture

Three melanoma short-term cultures were used: Ma-Mel-123 (23 passages), Ma-Mel-108 (29 passages), and Ma-Mel-93 (15 passages). All three are derived from subcutaneous metastases. Ma-Mel-123 is *BRAF* and *NRAS* wild type, Ma-Mel-108 is *BRAF* V600E mutant and *NRAS* wild type, Ma-Mel-93 is *BRAF* wild type and *NRAS* G13R mutant [49]. Cell cultures were authenticated immediately before the experiments. Clinical information of Ma-Mel-123, Ma-Mel-108, Ma-Mel-93: age at biopsy in years (67, 35, 75), gender (female, female, male), localization of primary tumor (mucosa, occult, occult), type of primary tumor (mucosal, occult, occult), stage at biopsy (IV, IV, IV), biopsy origin (all cutaneous metastases), and survival in months after biopsy (6.3+, 8.8, 9.2). Melanoma cells were cultured under standard culture conditions [49]. All clinical investigations have been conducted according to the principles expressed in the Declaration of Helsinki and have been approved by the Ethics committee of the Medical Faculty of the University of Leipzig (Az 023-16-01022016).

### Single-cell RNA-seq

Cell suspensions was generated by washing melanoma cells from different cultures with PBS followed by trypsinizing with TripLE Xpress (Invitrogen, Darmstadt, Germany) for 5 minutes. The reaction was stopped by adding media containing FCS. Single melanoma cells from short-term cultures were captured on an integrated fluidic circuit RNA-seq chip (Fluidigm, Hamburg, Germany) using the Fluidigm C1 system. Cells were loaded onto the chip at a concentration of 200 cells/μL and imaged by phase-contrast. Cell capture, cell lysis, reverse transcription, and cDNA amplification were performed on the chip as described [50]. Illumina libraries were constructed using the Illumina Nextera XT DNA Sample Preparation kit using the protocol supplied by Fluidigm. Sequencing libraries were pooled (3 μL each) and purified with 18% SPRI beads. Library concentration and size distribution were assessed on an Agilent Bioanalyzer and with Qubit dsDNA HS Assay kits and a Qubit 2.0 Fluorometer (Thermo Fisher Scientific, Invitrogen, Darmstadt). Each cell was paired-end sequenced (100 base reads) on an Illumina HiSeq 2500 to a depth of 2–5 million reads and base-calling, adaptor trimming, and de-multiplexing were performed as described [51, 52].

### Analysis of single-cell data by SOM portrayals and tSNE

Raw reads were processed using a custom script and aligned to a Bowtie2 [53] indexed human genome (grch38 sourced from ENSEMBL) using TopHat [54] with default settings. Transcript levels were quantified as fragments per kilobase of mapped reads (FPKM) generated by Cufflinks [55] using gencode protein coding genes (grch38 v22 Havana). We excluded cells that did express neither of two housekeeping genes ACTB and GAPDH. Ninety-two single cells remained for the transcriptome analysis of the *BRAF/NRAS* wt/wt culture, 84 cells for the replicate analysis of this culture, 54 cells for the *BRAF* mutant/*NRAS* wild type, and 77 cells for *BRAF* wild type/*NRAS* mutant culture, respectively. The mean number of genes detected was 6014 in the *BRAF*/*NRAS* wild type culture (4078 in the replicate) and 4655 in the mutant cell cultures. The higher gene detection rate in the first analysis of the short-term culture Ma-Mel-123 compared to the replicate might explain minor batch effects in the subsequent analysis.

Gene-centric expression data were quantile normalized, then centralized and clustered using self-organizing map (SOM) machine learning. The SOM method translates the approximately 15,000 gene expression profiles into 2,500 metagene expression profiles and visualizes their expression levels in each sample using a two-dimensional quadratic 50×50 grid. SOM size and topology were chosen to allow robust identification of expression modules in terms of so-called spots [10, 56]. For the functional interpretation we applied gene set analysis using Fisher's exact test and the gene set Z-score (GSZ) [57]. We considered gene sets related to biological processes (BP), cellular components (CC) or molecular function (MF) of the gene ontology (GO) classification, and standard literature sets [56, 58] and literature sets curated by our group. Sample diversity analysis, class discovery and presentation were performed as described previously [13]. For estimation of the quality of sample clustering we made use of the silhouette-plot [13]. All downstream methods have been described previously [10, 56] and are implemented in the R-package ‘oposSOM’ [11].

In order to demonstrate the similarity of replicate samples, a t-distributed stochastic neighbour embedding analysis (tSNE) [59]) was performed. The R-package ‘Seurat’ was used for running tSNE analysis with default settings [60]. Only small but systematic differences between the two replicates of wild type/wild type cells were interpreted as batch effects and were not considered in downstream analyses. Largescale copy number variations (CNVs) for the three short-term cultures were inferred from expression profiles by averaging gene expression over stretches of 50 genes on their respective chromosomes, as described in a recent study [38].

### Immunofluorescence staining

The original melanoma tissue corresponding to the Ma-Mel-123 culture was used for immunofluorescence staining using a standard protocol as described earlier [61]. Counterstaining was performed with 4′,6-Diamidin-2-phenylindol (DAPI) solution (Merck, Darmstadt, Germany). The following antibodies were used: TOP2A monoclonal antibody (Ki-S1, Thermo Fisher Scientific), CDK4 monoclonal antibody (H-22, Santa Cruz Biotechnology), ITGA1/CD49a monoclonal antibody (MAB1973Z, Chemicon International, Temecula, CA, U.S.A.), and Ki-67 monoclonal antibody (MIB-1, Dako, Glostrup, Denmark). Control staining of the sample was done with Mayer's Hemalaun solution (Dr. K. Hollborn, Leipzig, Germany). Pictures were taken with a Biorevo BZ-9000 microscope (Keyence, Neu-Isenburg, Germany).

### Inhibitors and IC50 determination

The following small molecule inhibitors were used: CDK4 inhibitor palbociclib (PD-0332991, SEL-S1116-5MG, Biozol, Eching, Germany), CDK2 inhibitor purvanolol (SEL-S7793-5MG, Biozol), BRAF inhibitor vemurafenib (PLX4032, SEL-S1267-10MG, Biozol), MEK1/2 inhibitor cobimetinib (RG7420, SEL-S8041-5MG, Biozol), and ERK1/2 inhibitor SCH772984 (SEL-S7101-5MG, Biozol). For determining the half-maximal growth inhibitory concentration (IC50), melanoma cells were seeded in a 96-well format. Cells were then drugged with serial dilutions of indicated inhibitors. Cellular viability was assessed using CellTiter-Glo (Promega, Mannheim, Germany). IC50 calculations were performed in GraphPad Prism^®^. For comparison of the response curve a nonlinear regression analysis was performed using Prism 6 software (GraphPad Software, La Jolla, USA). *P* values ≤ 0.05 were regarded as statistically significant.

## SUPPLEMENTARY MATERIALS








