# Supplementary material for: Mapping heterogeneity in patient-derived melanoma cultures by single-cell RNA-seq
Source: Oncotarget. 2016 Nov 26;8(1):846–62. doi: 10.18632/oncotarget.13666 (PMC5352202; doi:10.18632/oncotarget.13666)
Supplement: Supplementary file 1 [file oncotarget-08-846-s001.pdf]

Mapping heterogeneity in patient-derived melanoma cultures by single-cell RNA-seq

Supplementary Materials

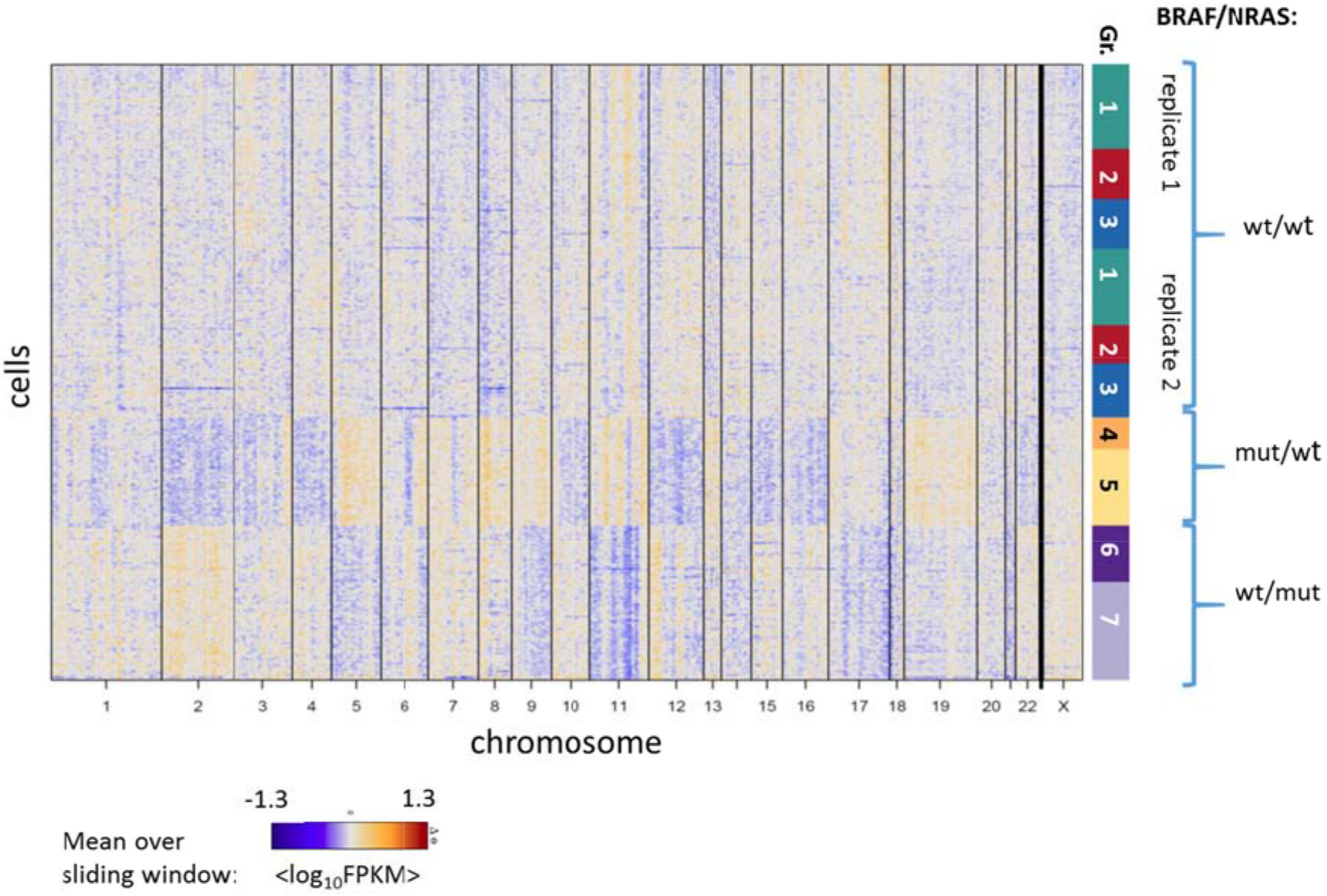

Supplementary Figure S1: Chromosomal expression landscape of patient-derived melanoma cell cultures. The different melanoma cell cultures are shown with individual cells (y axis) and chromosomal regions (x axis). The chromosomal landscape of inferred large-scale copy number variations distinguishes malignant from nonmalignant cells as described by Patel and co-workers [38]. Amplifications (red) and deletions (blue) were inferred by averaging expression over 50-gene stretches on the respective chromosomes. Each of the cultures shows a cell-culture specific expression pattern of inferred over- (red) and under- (blue) represented regions along the chromosomes. Wt/wt, *BRAF/NRAS* wild type; mut/wt, *BRAF* mut/*NRAS* wild type; wt/mut, *BRAF* wild type, *NRAS* mut.

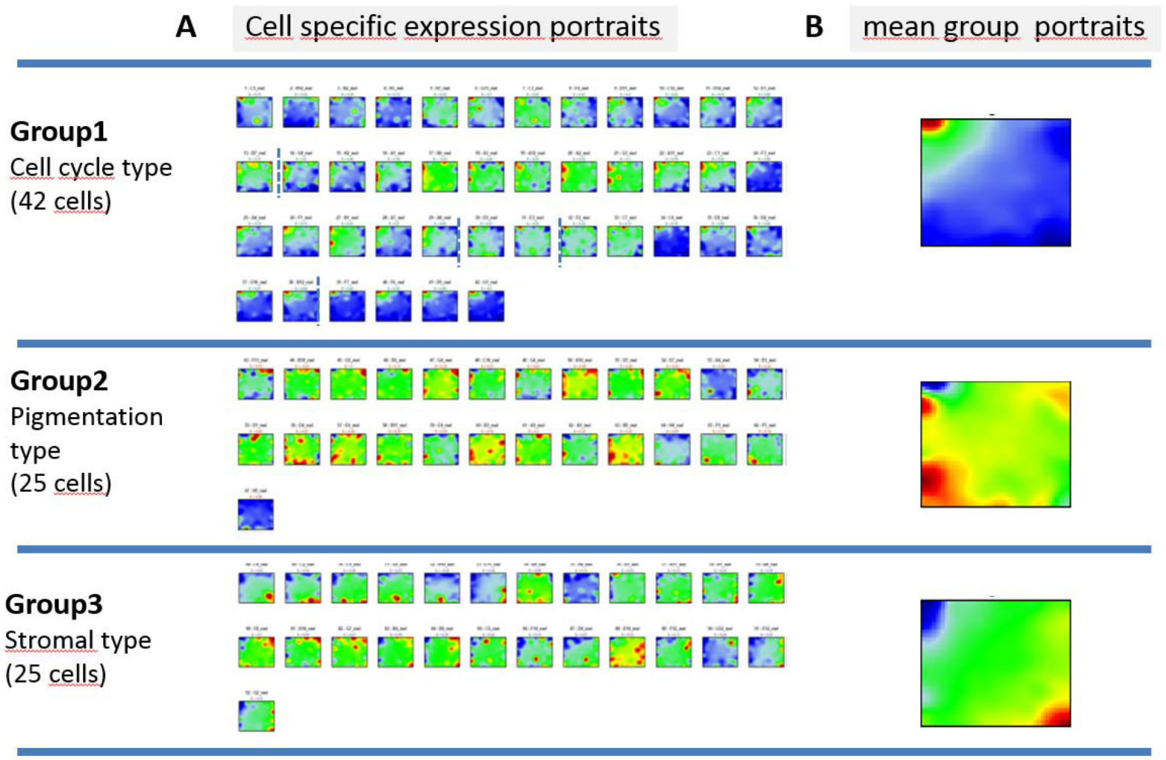

Supplementary Figure S2: SOM portrayal of single-cell expression data of *BRAF/NRAS* wild type cells. (**A**) Gallery of the SOM expression portraits of all 92 single cells analyzed. Red spot-like regions in the portraits refer to highly expressed genes whereas blue regions indicate low expression. A resolution of 50x50 pixels in the SOM portraits was used. The portraits were classified into three major groups. (**B**) Mean SOM expression portraits of each group. The mean portraits were calculated as log-mean expression in each of the pixels averaged over all single portraits in each group.

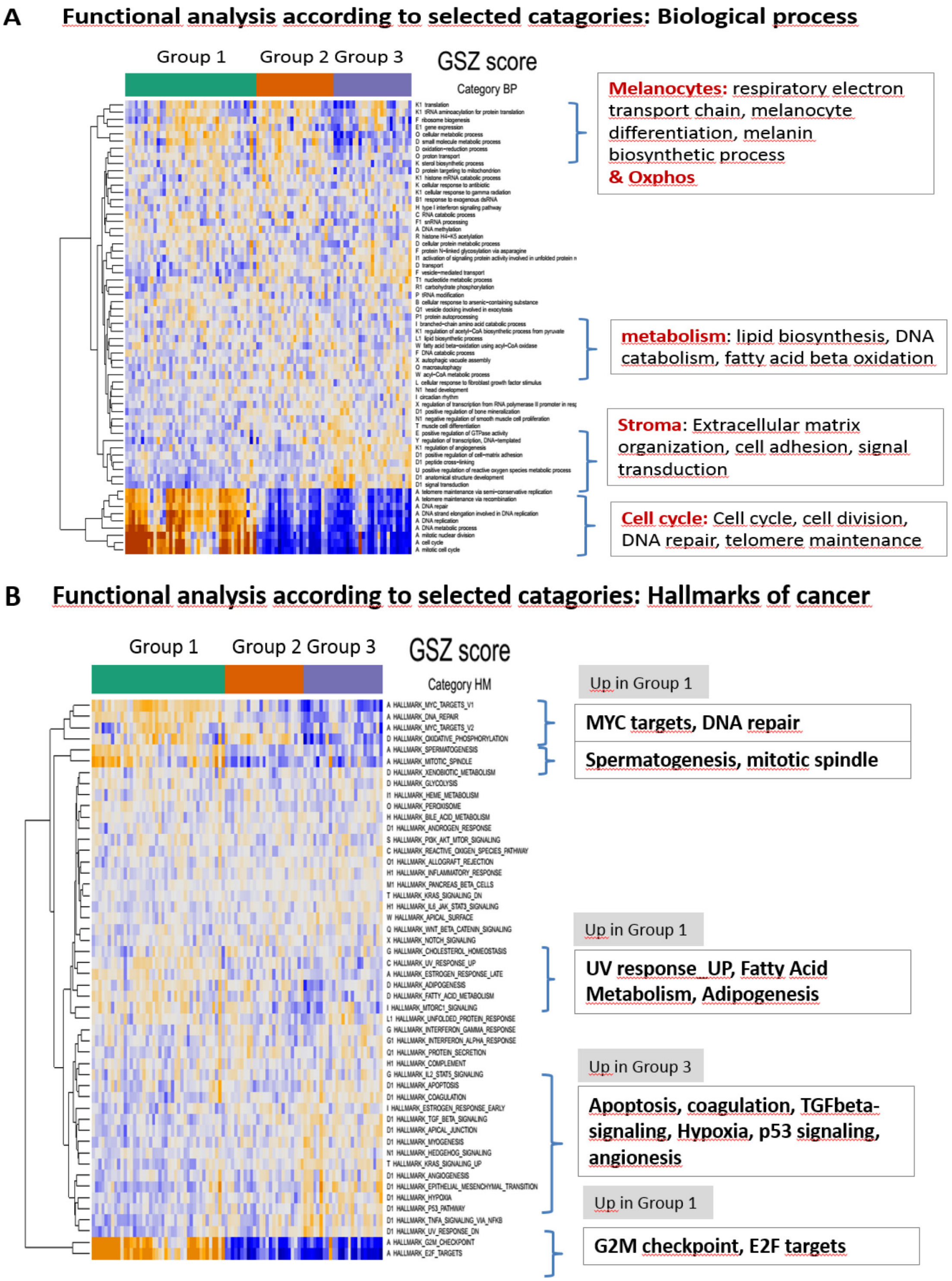

Supplementary Figure S3: Functional analysis of the single cell expression data of *BRAF/NRAS* wild type cells. The heatmaps visualize GSZ-overexpression profiles of functional gene sets taken from the categories biological process (**A**) and hallmarks of cancer (**B**) (Liberzon et al., 2015). The cells are sorted according to their group membership extracted from Supplementary Figure S4 below.

REFERENCES

1. Liberzon A, Birger C, Thorvaldsdóttir H, Ghandi M, Mesirov JP, Tamayo P. The Molecular Signatures Database (MSigDB) hallmark gene set collection. Cell Syst. 2015; 1:417–425.

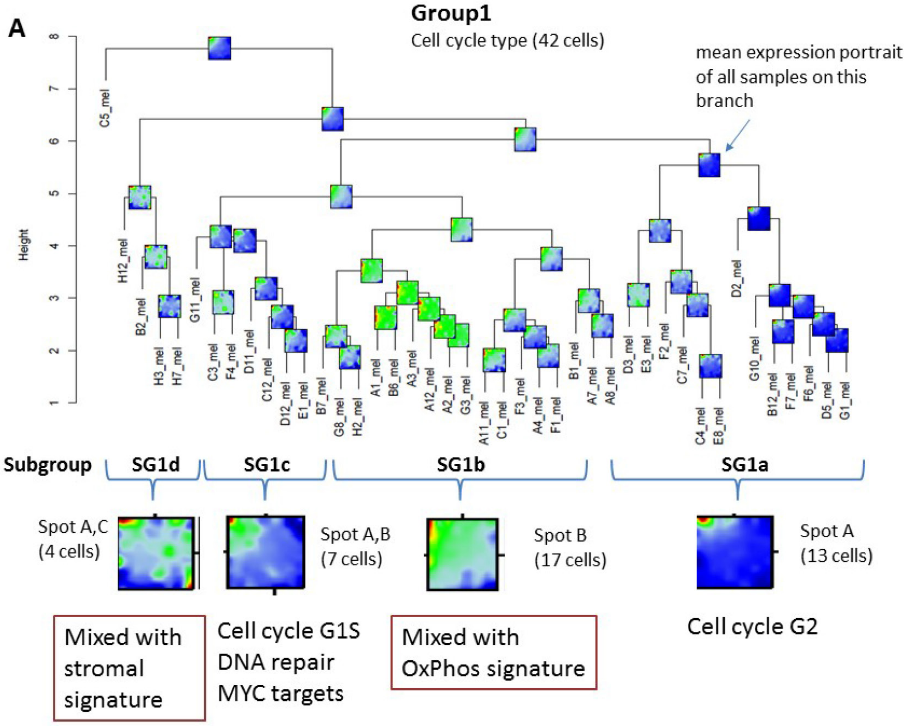
A
Group1
Cell cycle type (42 cells)
mean expression portrait of all samples on this branch
Height
Subgroup
SG1d
SG1c
SG1b
SG1a
Spot A,C (4 cells)
Spot A,B (7 cells)
Spot B (17 cells)
Spot A (13 cells)
Mixed with stromal signature
Cell cycle G1S DNA repair MYC targets
Mixed with OxPhos signature
Cell cycle G2

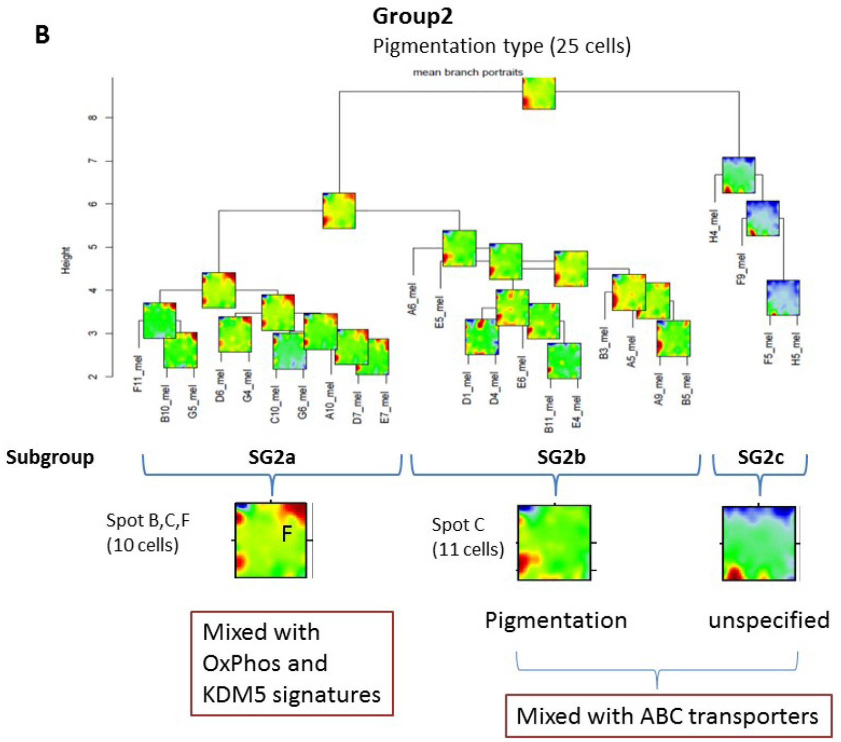
B
Group2
Pigmentation type (25 cells)
mean branch portraits
Height
Subgroup
SG2a
SG2b
SG2c
Spot B,C,F (10 cells)
F
Spot C (11 cells)
Mixed with OxPhos and KDM5 signatures
Pigmentation
unspecified
Mixed with ABC transporters

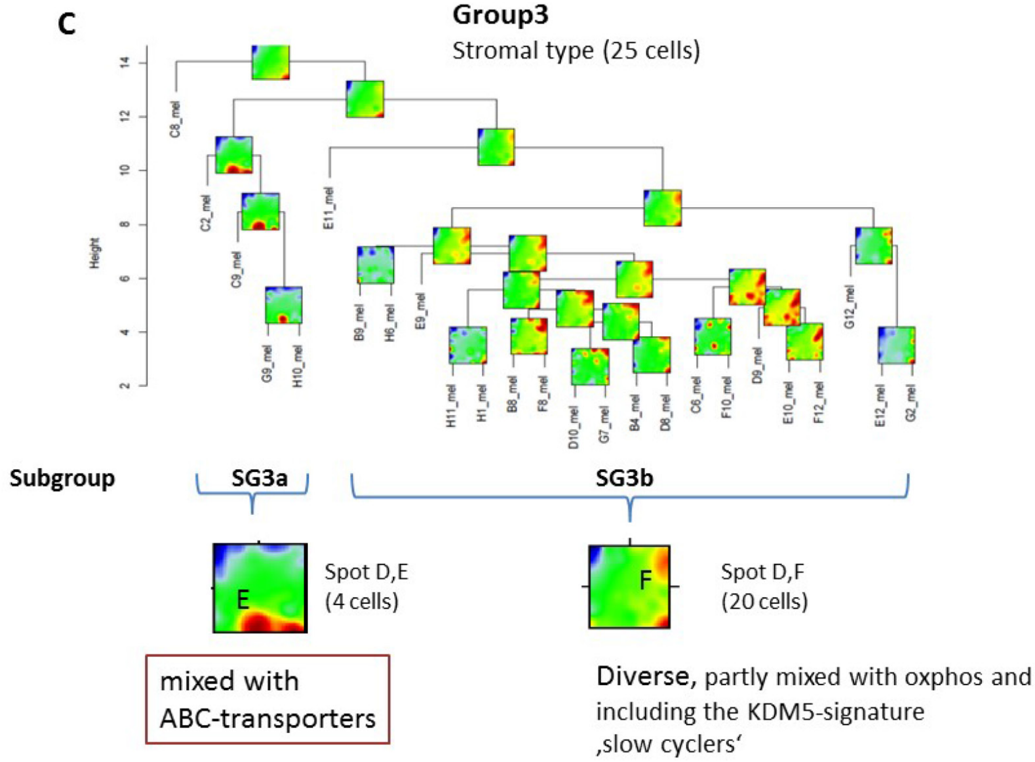

Supplementary Figure S4: Disentanglement of heterogeneity of group 1 to group 3 of *BRAF/NRAS* wild type cells. Hierarchical clustering of the SOM-portraits of the individual cell stratifies each of the groups into subgroups with a different functional context as indicated in the figure.

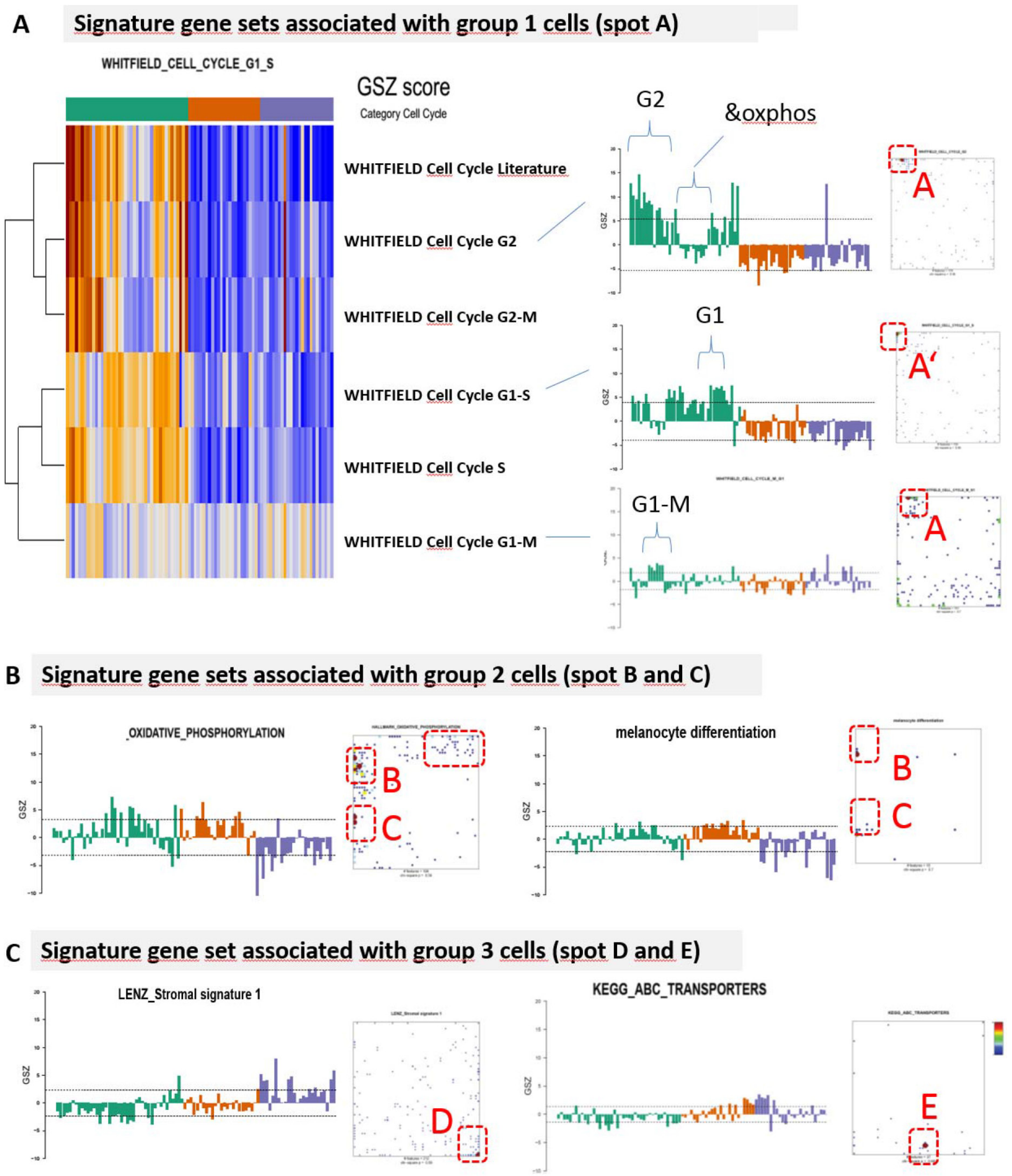

Supplementary Figure S5: Mapping of selected gene sets associated with group 1 to 3 of *BRAF/NRAS* wild type cells. Gene sets were taken from different independent studies on either cell cycle genes, oxidative phosphorylation, melanocyte differentiation, stroma and ABC transporters and mapped to the single-cell expression data (Whitfield et al., 2002; Lenz et al., 2008), and the respective gene ontology and KEGG categories. The samples are ranked according to their subgroup-assignment in Figure 1C.

REFERENCES

1. Lenz G, Wright G, Dave SS, Xiao W, Powell J, Zhao H, et al. Lymphoma/Leukemia Molecular Profiling Project. Stromal gene signatures in large-B-cell lymphomas. N Engl J Med. 2008; 359:2313–23.

2. Whitfield ML, Sherlock G, Saldanha AJ, Murray JI, Ball CA, Alexander KE, et al. Identification of genes periodically expressed in the human cell cycle and their expression in tumors. Mol Biol Cell. 2002; 13:1977–2000

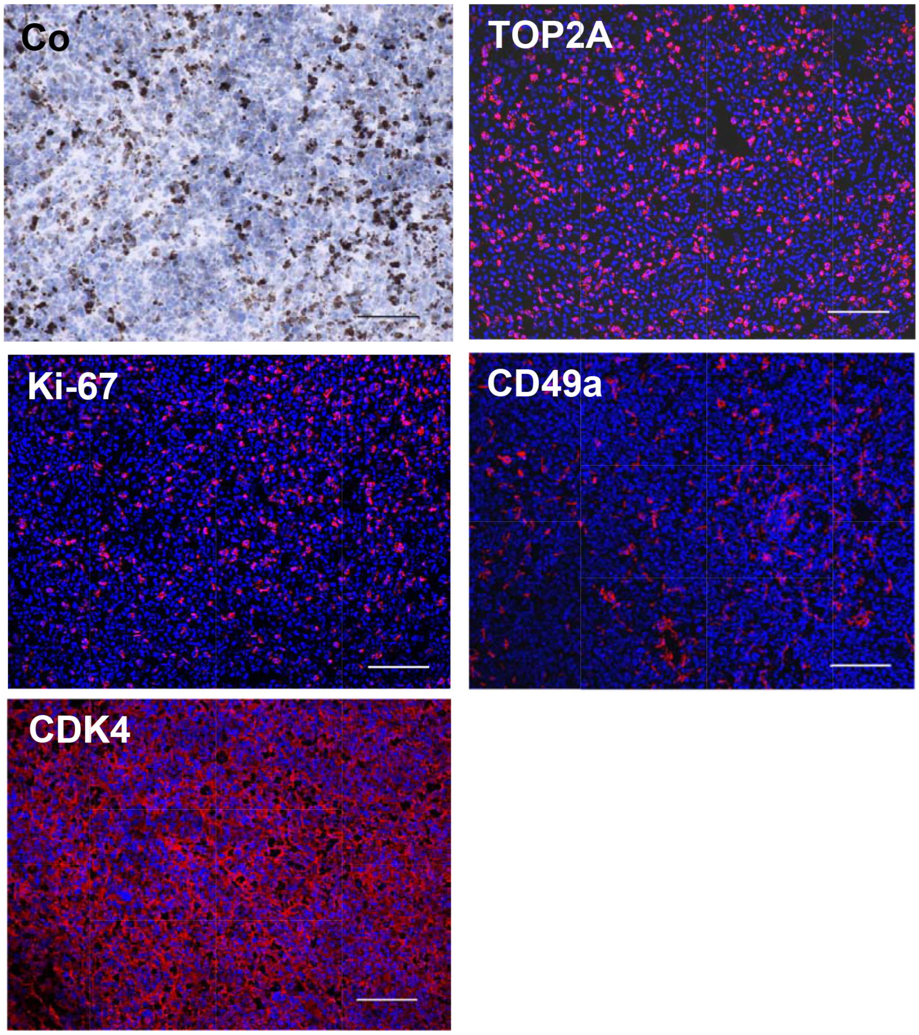

Supplementary Figure S6: Immunofluorescence staining of selected marker proteins of different subgroups of cells from the single-cell analysis of the *BRAF/NRAS* wild type sample. Cryosections of the original tumor sample corresponding to the Ma-Mel-123 culture were generated and stained with immunofluorescence staining using antibodies directed against TOP2A and MKI67 (Ki-76) (both group 1 genes), ITGA1/CD49a (group 3 gene), and CDK4 (present in all subgroups), respectively. Nuclear staining was performed with DAPI. Original magnifications x 20. Scale bars indicate 100 µM. Co, staining with Mayer's hemalaun.

A. Expression characteristics of gene sets taken from Tirosh et al. (2016)

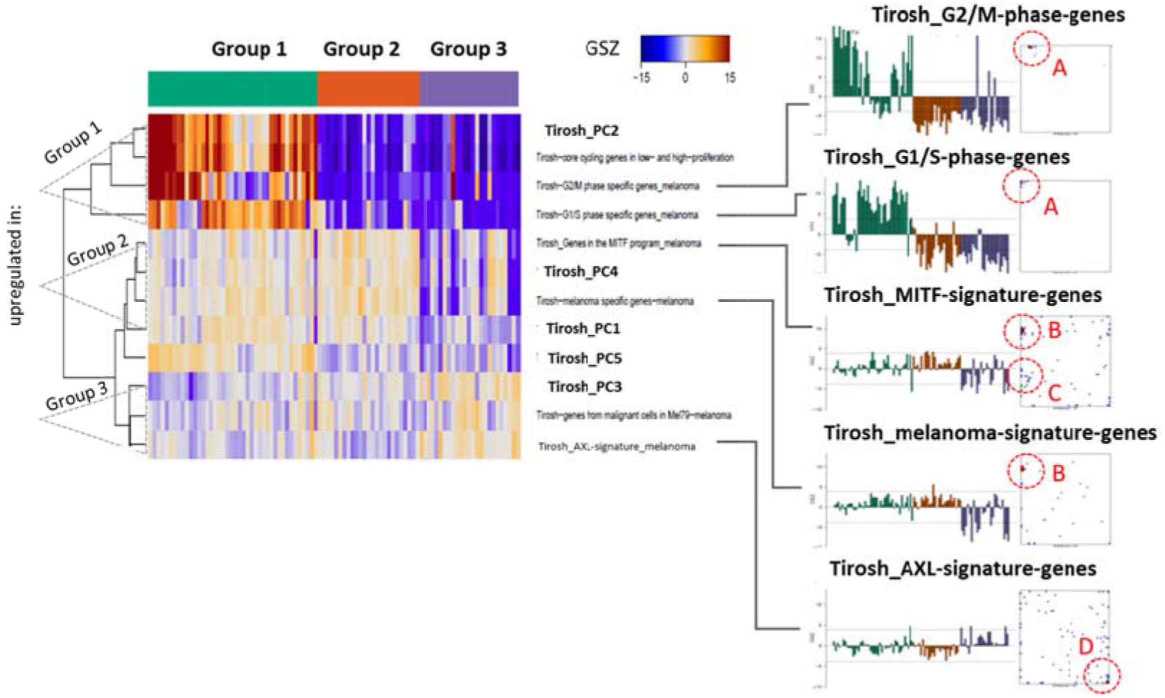

B. Profiles and maps of gene sets characterizing principal components 1 – 5 of melanoma single cell gene expression data published by Tirosh et al. (2016)

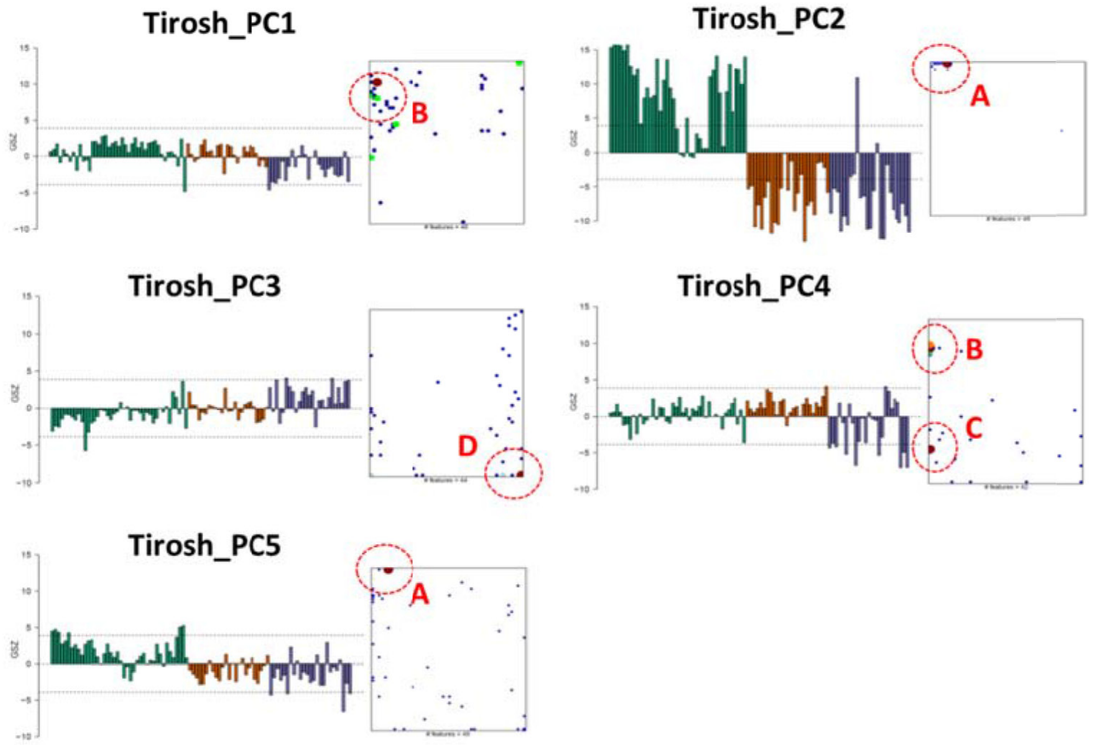

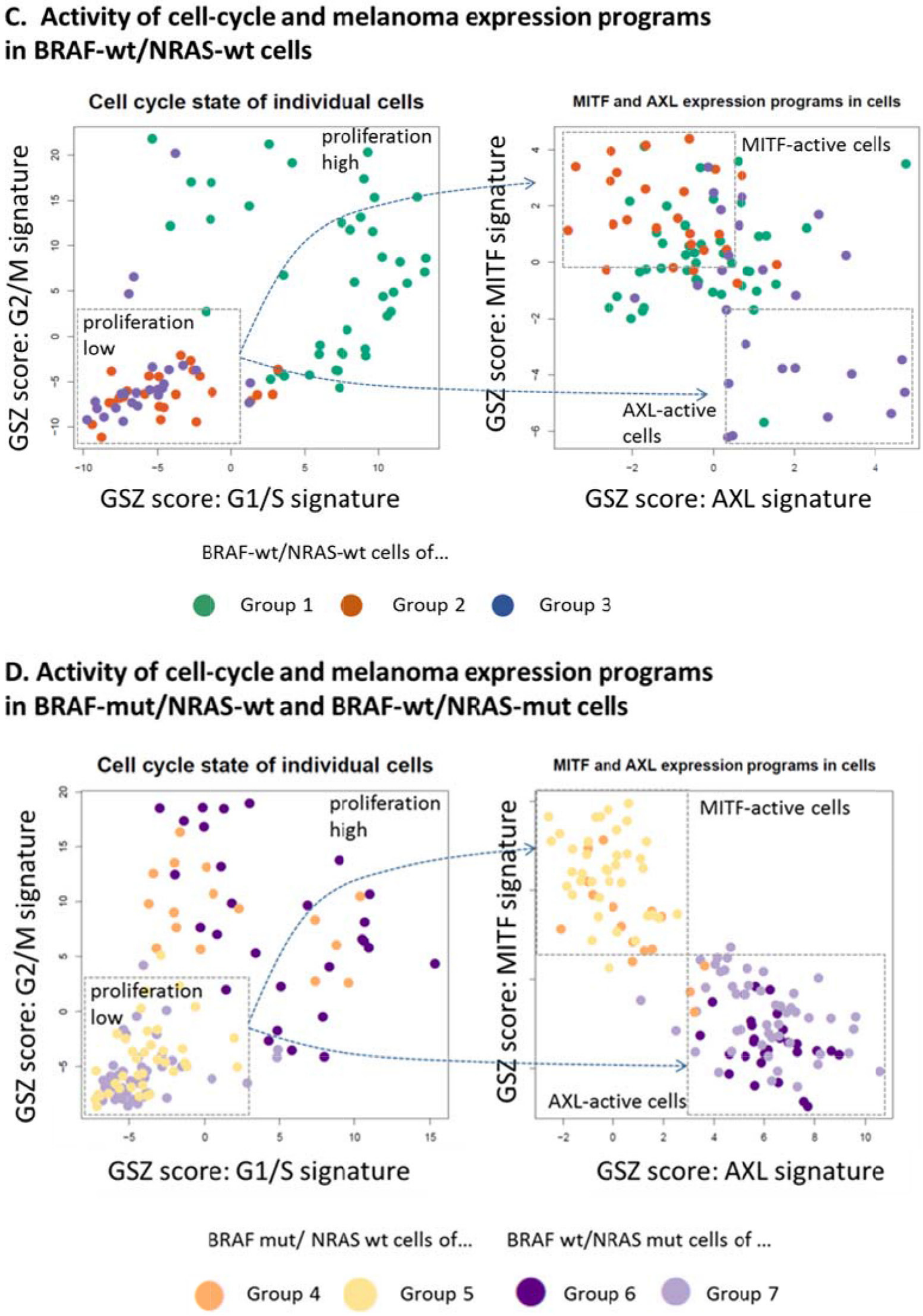

Supplementary Figure S7: Mapping of signature genes of an independent study. Signature genes of the single-cell RNA-seq analysis of melanoma samples from Tirosh and co-workers [15] were mapped onto the gene expression data of the present study. (**A**) Heatmap of the GSZ score of different gene sets of Tirosh and co-workers [15] in the present data. The group of proliferative cells (group 1) shows high GSZ scores of cell cycle signature genes. Group 2 cells show high expression of MITF- and melanoma-signature genes. AXL signature genes are highly expressed in stromal group 3. The right part of the figure shows selected GSZ-profiles and gene set maps which illustrate accumulation of the genes in spots (A–D). (**B**) Gene sets referring to principal components 1–5 (PC1 – PC5) of the study of Tirosh and co-workers [15]. The PC-sets agree with the spot and group characteristics of the present study. (**C**) Correlation plots between cell cycle (left plot) and melanoma expression programs (right plot) of group 1–3 cells. Cells of group 2 and group 3 with low cell cycling activity split into two populations according to the activity of MITF- and AXL programs. (**D**) The same analysis as in (C) for cells of groups 4 and 5 (*BRAF* mutant/*NRAS* wild type) and of groups 6 and 7 (*BRAF* wild type/*NRAS* mutant) of the present analysis. The BRAF mutation associates with higher activity of the MTIF-program and lower activity of the AXL program whereas the NRAS mutation associates with higher activity of the AXL program and lower activity of the MTIF program.

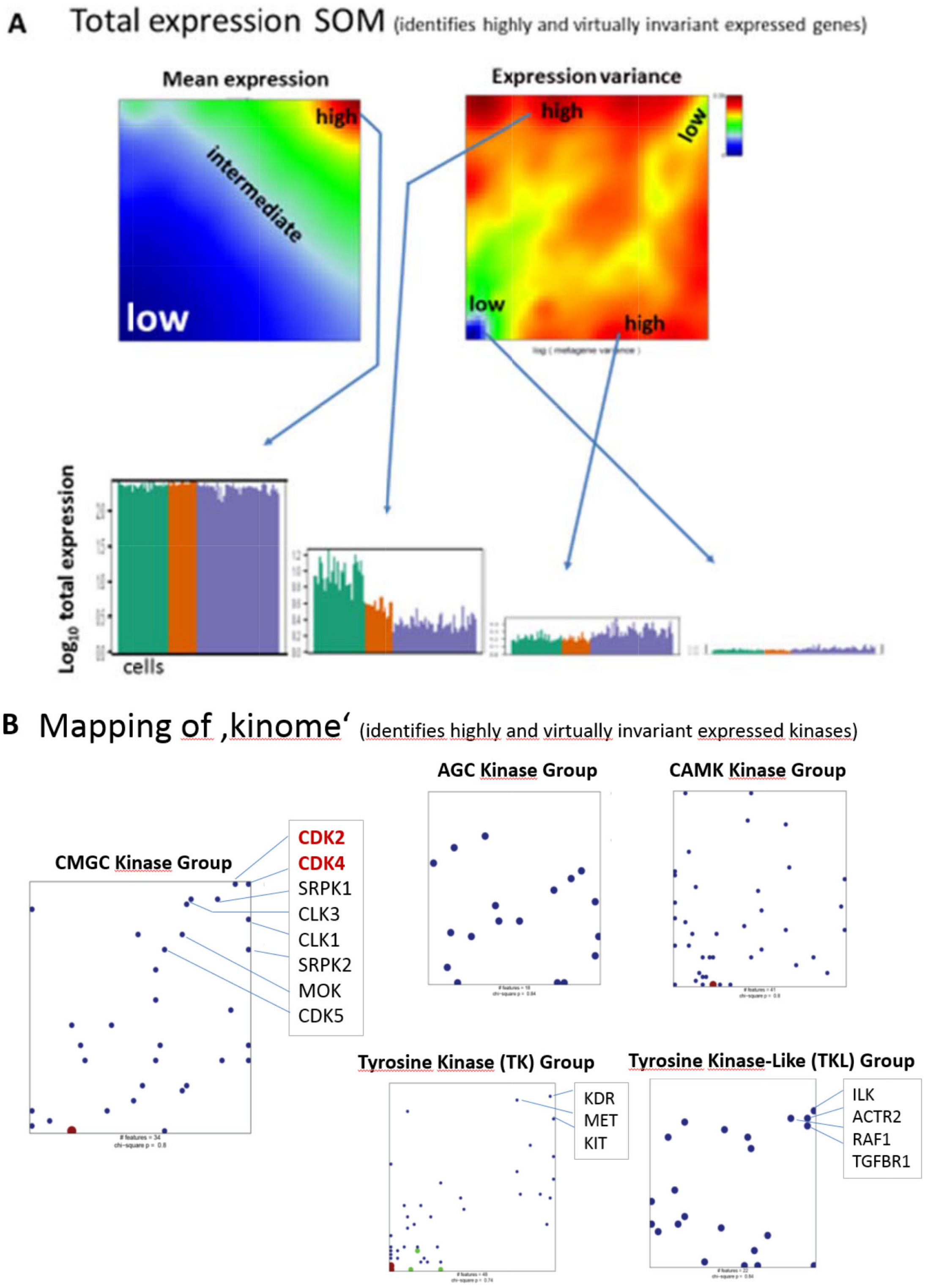
A
Total expression SOM (identifies highly and virtually invariant expressed genes)
Mean expression
high
intermediate
low
Expression variance
high
low
low
high
Log10 total expression
cells
B
Mapping of ‚kinome' (identifies highly and virtually invariant expressed kinases)
CMGC Kinase Group
CDK2
CDK4
SRPK1
CLK3
CLK1
SRPK2
MOK
CDK5
AGC Kinase Group
CAMK Kinase Group
Tyrosine Kinase (TK) Group
KDR
MET
KIT
Tyrosine Kinase-Like (TKL) Group
ILK
ACTR2
RAF1
TGFBR1

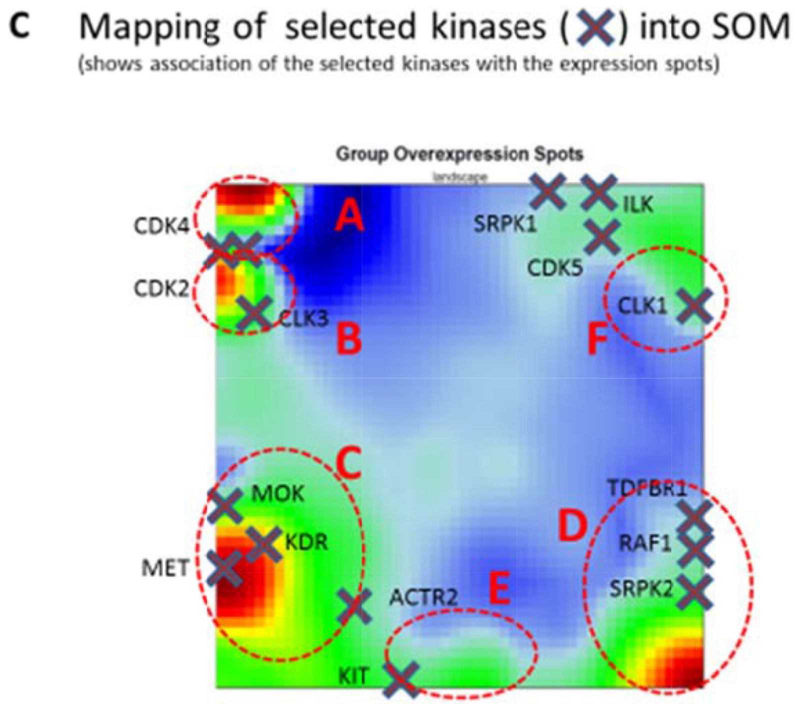

Supplementary Figure S8: Kinome analysis of single cells of *BRAF/NRAS* wild type culture. (**A**) A SOM was trained using total (i.e., not centralized) expression values. It enabled to identify highly and virtually invariantly expressed genes. Highly expressed and virtually invariant genes accumulate in the right upper corner of the map. The expression profiles in the lower part of panel in (A) were taken from different regions of the map (see arrows). They clearly illustrate the changing expression level and its variance. (**B**) Genes were mapped referring to different categories of the kinome into the SOM and selected genes from the region in the right upper corner (high and almost invariant expression). CDK4 and CDK2 best met the criteria of high expression and small expression variance. (**C**) Genes selected were mapped into the original SOM that was trained using centralized expression data. The genes selected accumulate near spots A to E.

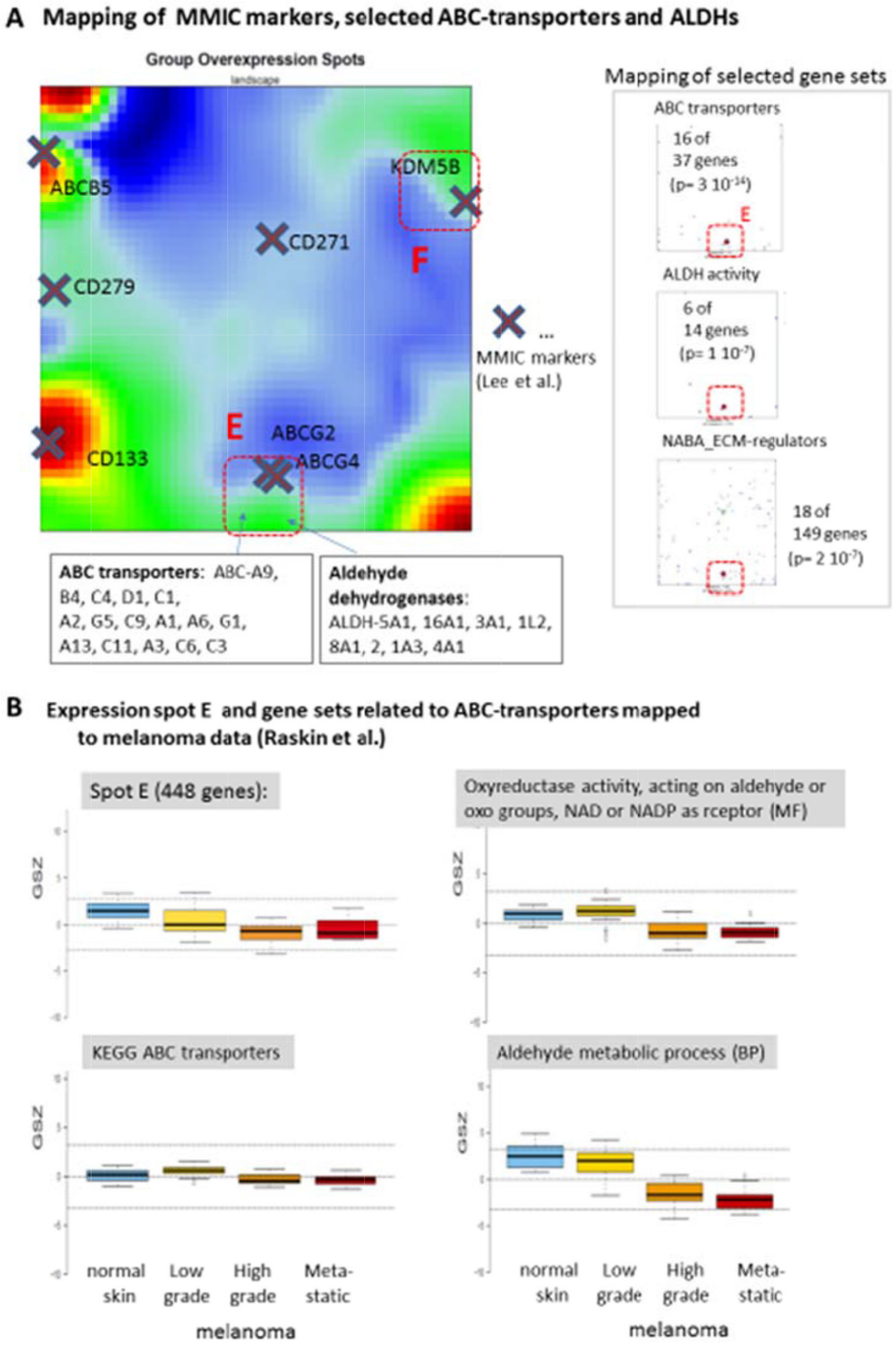

Supplementary Figure S9: Mapping of malignant melanoma stem and initiating cell (MMIC) marker genes into the single-cell expression landscape of the *BRAF/NRAS* wild type culture. (**A**) Spot E accumulates a series of *ABC* transporter and *ALDH* genes and related gene sets as illustrated by the gene set maps in the right part of the figure. MMIC marker genes were taken from different independent studies [23]. (**B**) Mapping of *ABC* transporter and *ALDH* signatures of spot E into the melanoma data of an independent study on gene expression profiles of normal skin, low-grade, high-grade and metastatic melanomas [21]. Note that the ABC- and ALDH-signatures are activated at early tumor stages.

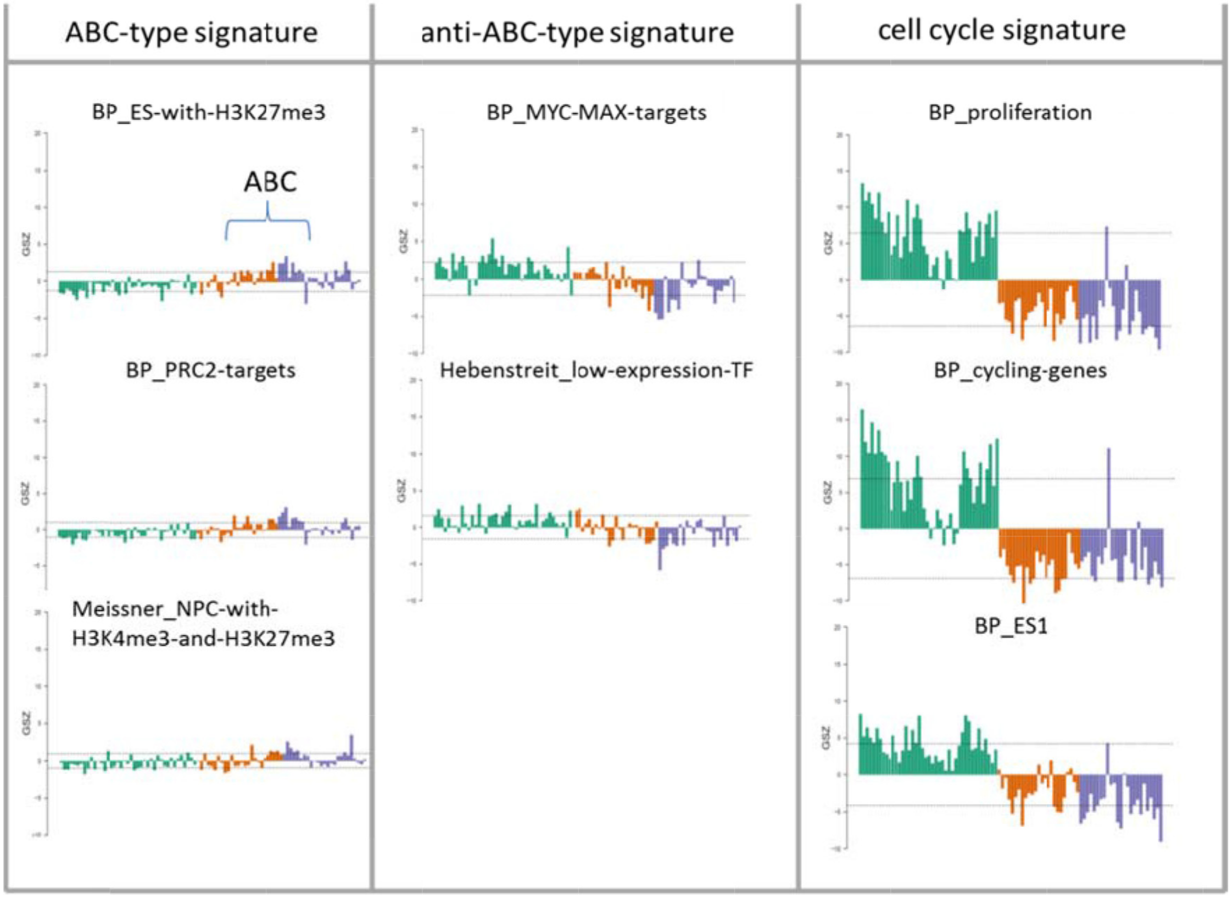

Supplementary Figure S10: Overexpression profiles of the ABC- and cell cycle signatures of selected gene sets related to embryonic stem cells. Gene expression signatures of different independent studies were mapped on the gene expression groups of the present study. Gene expression patterns referred to stem-cell like gene expression [31], poised promoters in neuronal progenitor cells (NPC) [32] and expression classes of different transcription factors [33]. BP, Ben-Porath [31]; TF, transcription factors; ES, embryonic stem cells.

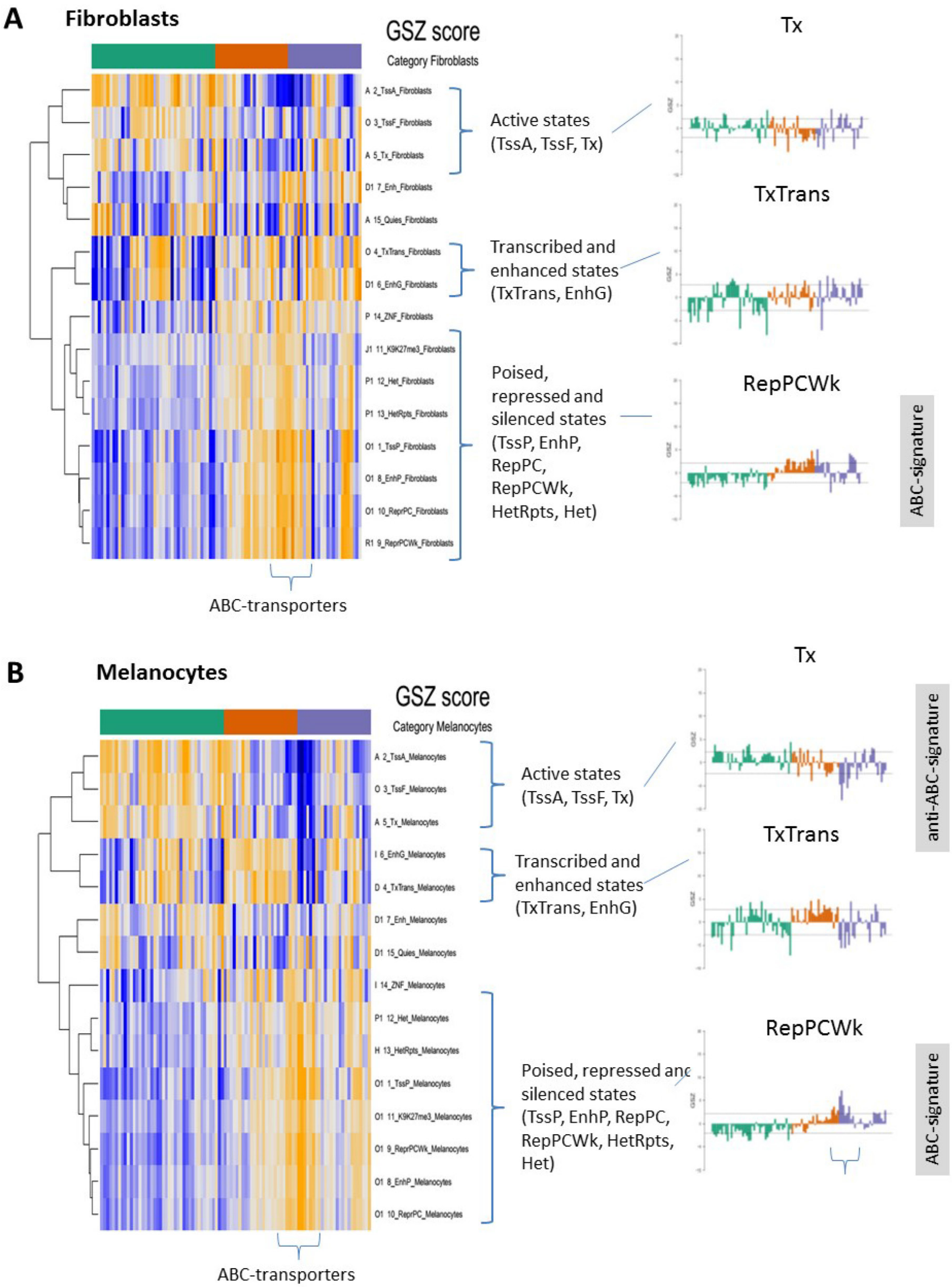
A
Fibroblasts
GSZ score
Category Fibroblasts
A 2_TssA_Fibroblasts
O 3_TssF_Fibroblasts
A 5_Tx_Fibroblasts
D1 7_Enh_Fibroblasts
A 15_Quies_Fibroblasts
O 4_TxTrans_Fibroblasts
D1 6_EnhG_Fibroblasts
P 14_ZNF_Fibroblasts
J1 11_K9K27me3_Fibroblasts
P1 12_Het_Fibroblasts
P1 13_HetRpts_Fibroblasts
O1 1_TssP_Fibroblasts
O1 8_EnhP_Fibroblasts
O1 10_ReprPC_Fibroblasts
R1 9_ReprPCWk_Fibroblasts
Active states (TssA, TssF, Tx)
Transcribed and enhanced states (TxTrans, EnhG)
Poised, repressed and silenced states (TssP, EnhP, RepPC, RepPCWk, HetRpts, Het)
Tx
TxTrans
RepPCWk
ABC-signature
ABC-transporters
B
Melanocytes
GSZ score
Category Melanocytes
A 2_TssA_Melanocytes
O 3_TssF_Melanocytes
A 5_Tx_Melanocytes
I 6_EnhG_Melanocytes
D 4_TxTrans_Melanocytes
D1 7_Enh_Melanocytes
D1 15_Quies_Melanocytes
I 14_ZNF_Melanocytes
P1 12_Het_Melanocytes
H 13_HetRpts_Melanocytes
O1 1_TssP_Melanocytes
O1 11_K9K27me3_Melanocytes
O1 9_ReprPCWk_Melanocytes
O1 8_EnhP_Melanocytes
O1 10_ReprPC_Melanocytes
Active states (TssA, TssF, Tx)
Transcribed and enhanced states (TxTrans, EnhG)
Poised, repressed and silenced states (TssP, EnhP, RepPC, RepPCWk, HetRpts, Het)
Tx
anti-ABC-signature
TxTrans
RepPCWk
ABC-signature
ABC-transporters

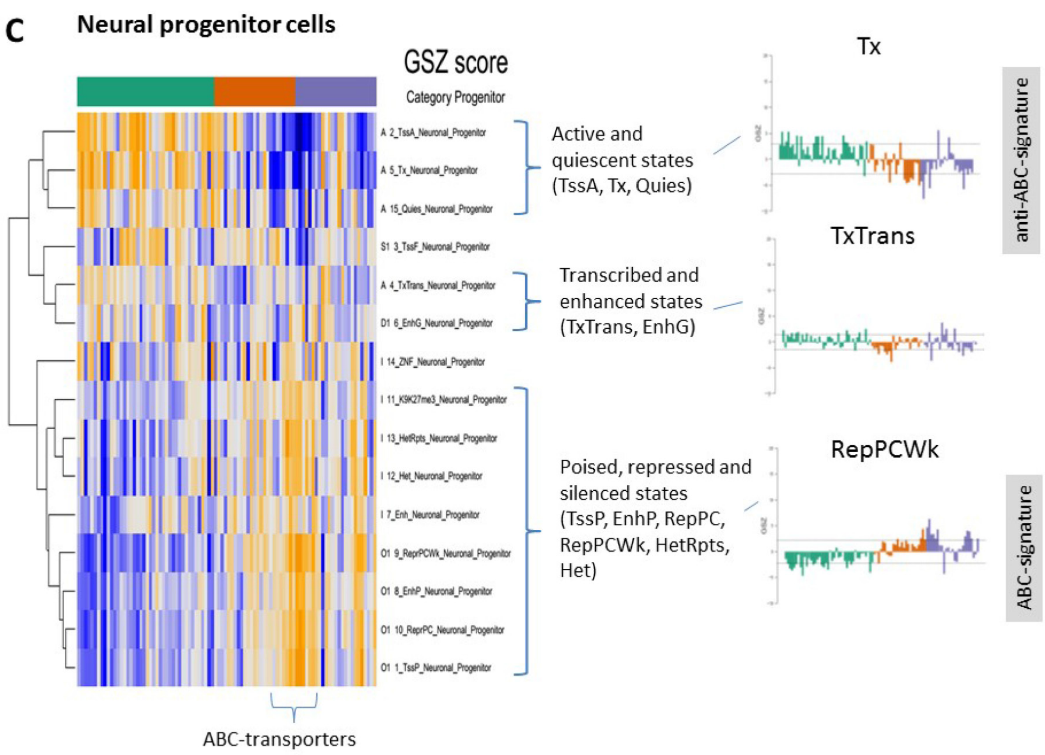

Supplementary Figure S11: GSZ-expression heatmaps of genes referring to 15 different chromatin states in fibroblasts, melanocytes and neural progenitor cells. Genes upregulated in group 1 cells are commonly in transcriptional active states whereas poised, repressed and silenced states are on low expression level on the average. Interestingly, this relation reverses for cells showing the ABC-transporter signature meaning that, poised and repressed states show relatively high gene expression which suggests aberrant chromatin remodeling in melanoma cells showing the ABC-transporter signature. Note that melanocytes specifically express selected active states in group2 cells. The right part shows selected profiles as bar plots.

The reference data and chromatin state assignments were downloaded from http://egg2.wustl.edu/roadmap/web_portal/chr_state_learning.html.

The states were defined as follows:

1	TssP	TSS_poised
2	TssF	TSS_flanking_more_upstream
3	TssA	TSS_active
4	Tx	Transcription
5	EnhG	Transcription Enhancer-like
6	Enh	Enhancer_active_with_weakK4me1_strong_K27ac
7	EnhP	Enhancer_poised
8	ReprPCWk	Repressed_polycomb_weak
9	ReprPC	Repressed_polycomb
10	K9K27me3	H3K9me3_K27me3
11	ZNF	Zinc_finger_genes_H3K36me3_K9me3
12	HetRpts	Heterochromatin_at_repeats
13	Het	Heterochromatin
14	Quies	Quiescent
15	K9acLow	low H3K9ac

REFERENCES

1. Ernst J, Kellis M. Discovery and characterization of chromatin states for systematic annotation of the human genome. Nat Biotech. 2010; 28:817–25.

2. Ernst J, Kheradpour P, Mikkelsen TS, Shoresh N, Ward LD, Epstein CB, et al. Mapping and analysis of chromatin state dynamics in nine human cell types. Nature. 2011; 473:43–9.

3. Roadmap Epigenomics Consortium, Kundaje A, Meuleman W, Ernst J, Bilenky M, Yen A, Heravi-Moussavi A, Kheradpour P, Zhang Z, Wang J, Ziller MJ, Amin V, Whitaker JW, et al. Integrative analysis of 111 reference human epigenomes. Nature. 2015; 518:317–30.

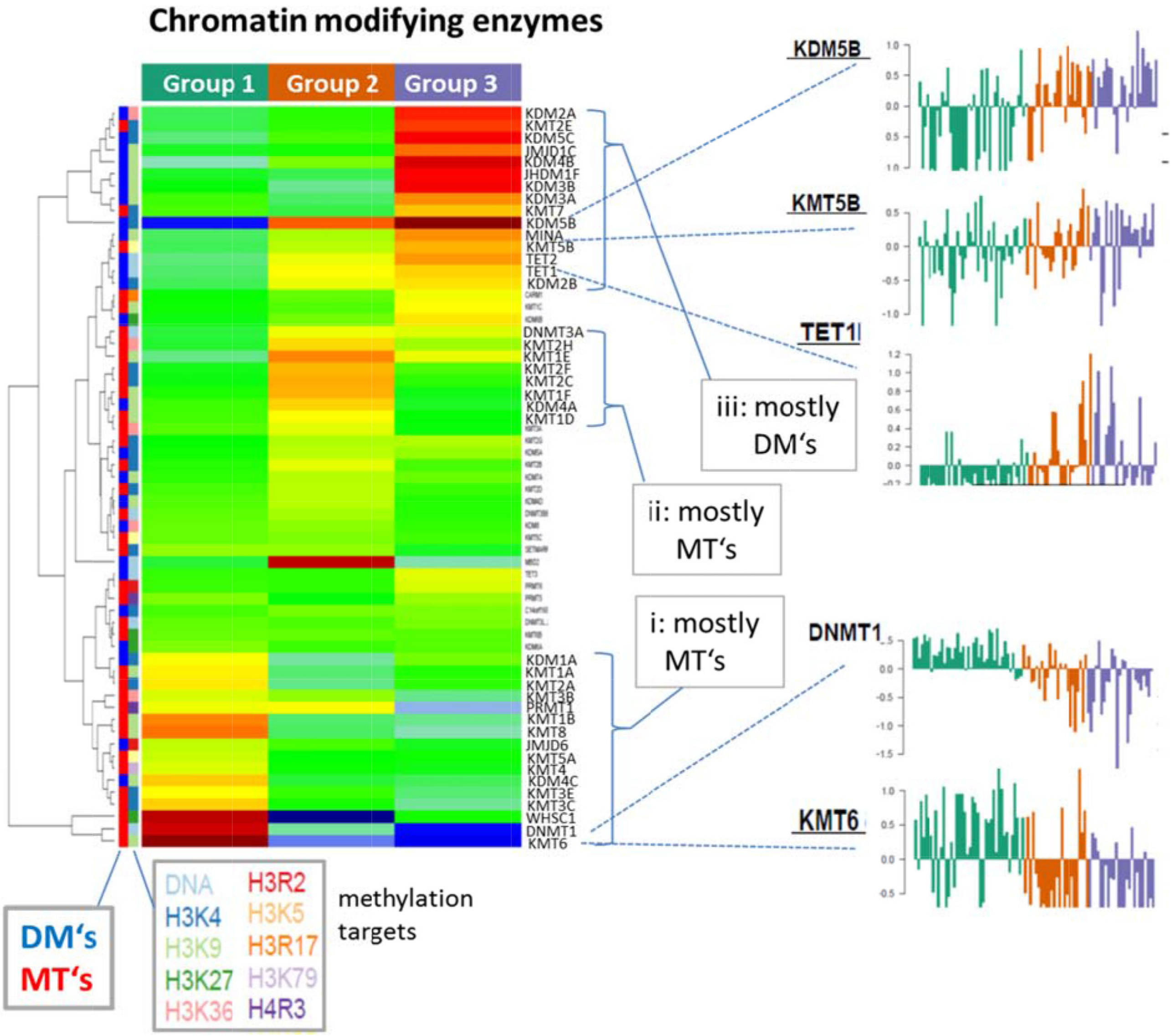

Supplementary Figure S12: Expression heatmap of chromatin modifying enzymes in the three expression groups of single cells of the *BRAF/NRAS* wild type culture. The gene expression data of methyltransferases (MTs) and demethylases (DMs) of DNA cytosines, histone lysine and arginine side chains split into three main clusters (i-iii) of different expression profiles upregulated typically in one of the three groups of single cells. The type of enzyme (MT or DM) and the methylation targets (DNA, histone lysine or arginine side chains mostly at H3) are color labeled in the color bars as indicated. Cluster i, upregulated in group 1 cells collects mainly MTs such as the DNMT1 and KMT6 (alias EZH2) methylating DNA and H3K27me3, respectively. Cluster ii upregulated in group 2 cells also contains mainly MTs whereas cluster iii, upregulated mainly in group 3 cells includes a high fraction of DMs such as the MMIC gene *KDM5B* and the demethylases of DNA TET2 and TET3. The asymmetrical distribution of MTs and DMs especially between clusters i and iii seems reasonable, because highly proliferation cells of group 1 need high activities of MTs for maintenance of the methylation levels at DNA and the histones.

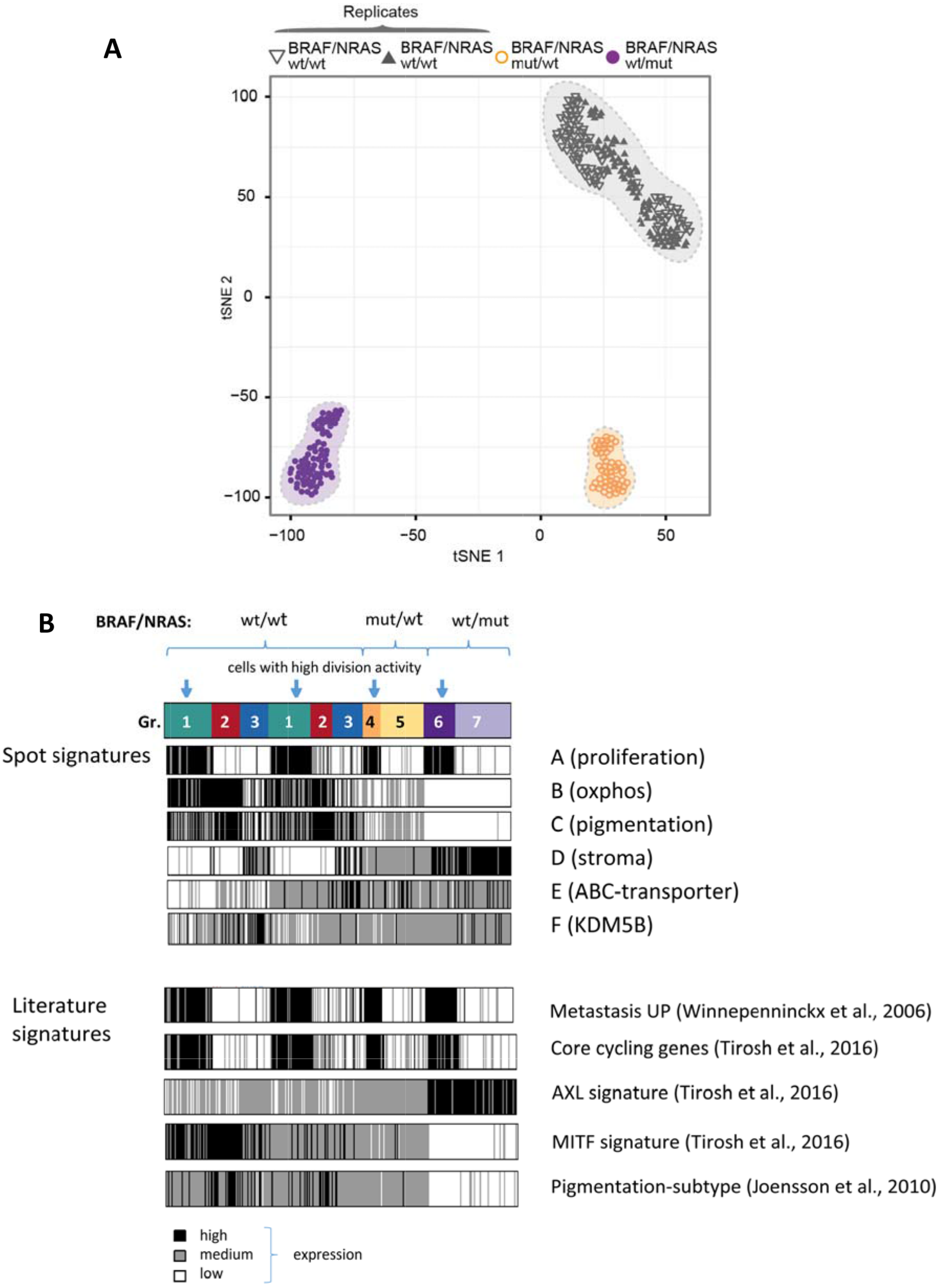

Supplementary Figure S13: Characterization of single-cell expression of the three different patient-derived short-term cultures. (**A**) PCA and unbiased clustering using tSNE reveals three major cell clusters which are separated based on the different short-term cultures used. Cells of replicate experiments intermix within one cluster. (**B**) Gene signatures (groups 1–7) from the present study were used for comparison of the three different cell cultures and for comparison with different published data sets [15, 16, 18]. All three cell cultures of the present study contain a significant fraction of cells in the proliferative state (30% to 45% of cells). The spot-signatures B (oxphos) and C (pigmentation) were activated in wt/wt cells and deactivated in NRAS mutant cells. The latter cells instead show an activated stromal signature (spot D) with similar regulation as the AXL signature defined by Tirosh and co-workers [15]. The MITF signature resembles the combined oxphos and pigmentation signatures of the present study and that of the melanoma pigmentation subtype [16].

Supplementary Table S1: Complete list of genes of spot A, B, C and D: Gene name, statistics, position in SOM, and description. See Supplementary_Table_S1

Supplementary Table S2: Complete list of genes of spot E: Gene name, statistics, position in SOM, and description. See Supplementary_Table_S2

Supplementary Table S3: List of differentially expressed genes between different cell cultures.
See Supplementary_Table_S3
