# Supplementary material for: Mapping heterogeneity in patient-derived melanoma cultures by single-cell RNA-seq
Source: Oncotarget. 2016 Nov 26;8(1):846–62. doi: 10.18632/oncotarget.13666 (PMC5352202; doi:10.18632/oncotarget.13666)
Supplement: Supplementary file 2 [file oncotarget-08-846-s002.docx]

**Supplementary Table 1. Complete list of genes of spot A,B,C and D: Gene name, statistics, position in SOM, and description.**

| **Genes of spot A** | | | | | | | | | | |
| --- | --- | --- | --- | --- | --- | --- | --- | --- | --- | --- |
| **Symbol** | **Correlation ^1^** | | **->t.score** | | **->p.value** | | **Metagene ^2^** | | **Chromosome** | **Description** |
| **ASF1B** | 0.88 | 17.58 | | 0.E+00 | | 4 x 50 | | 19 p13 | | anti-silencing function 1B histone chaperone [Source:HGNC Symbol;Acc:HGNC:20996] |
| **RRM2** | 0.86 | 15.86 | | 0.E+00 | | 5 x 50 | | 2 p25 | | ribonucleotide reductase M2 [Source:HGNC Symbol;Acc:HGNC:10452] |
| **HMGB2** | 0.85 | 15.33 | | 0.E+00 | | 5 x 50 | | 4 q34 | | high mobility group box 2 [Source:HGNC Symbol;Acc:HGNC:5000] |
| **SPC24** | 0.84 | 14.64 | | 0.E+00 | | 5 x 50 | | 19 p13 | | SPC24, NDC80 kinetochore complex component [Source:HGNC Symbol;Acc:HGNC:26913] |
| **UBE2T** | 0.84 | 14.49 | | 0.E+00 | | 4 x 50 | | 1 q32 | | ubiquitin-conjugating enzyme E2T [Source:HGNC Symbol;Acc:HGNC:25009] |
| **KIAA0101** | 0.84 | 14.40 | | 0.E+00 | | 3 x 50 | | 15 q22 | | KIAA0101 [Source:HGNC Symbol;Acc:HGNC:28961] |
| **CDK1** | 0.83 | 14.28 | | 0.E+00 | | 5 x 50 | | 10 q21 | | cyclin-dependent kinase 1 [Source:HGNC Symbol;Acc:HGNC:1722] |
| **TOP2A** | 0.83 | 14.25 | | 0.E+00 | | 6 x 50 | | 17 q21 | | topoisomerase (DNA) II alpha 170kDa [Source:HGNC Symbol;Acc:HGNC:11989] |
| **RAD51AP1** | 0.82 | 13.76 | | 0.E+00 | | 4 x 50 | | 12 p13 | | RAD51 associated protein 1 [Source:HGNC Symbol;Acc:HGNC:16956] |
| **PRC1** | 0.81 | 12.98 | | 0.E+00 | | 5 x 50 | | 15 q26 | | protein regulator of cytokinesis 1 [Source:HGNC Symbol;Acc:HGNC:9341] |
| **UBE2C** | 0.81 | 12.91 | | 0.E+00 | | 6 x 50 | | 20 q13 | | ubiquitin-conjugating enzyme E2C [Source:HGNC Symbol;Acc:HGNC:15937] |
| **MAD2L1** | 0.80 | 12.79 | | 0.E+00 | | 5 x 50 | | 4 q27 | | MAD2 mitotic arrest deficient-like 1 (yeast) [Source:HGNC Symbol;Acc:HGNC:6763] |
| **HIST1H4C** | 0.80 | 12.69 | | 0.E+00 | | 5 x 49 | | 6 p22 | | histone cluster 1, H4c [Source:HGNC Symbol;Acc:HGNC:4787] |
| **NUSAP1** | 0.80 | 12.50 | | 0.E+00 | | 6 x 50 | | 15 q15 | | nucleolar and spindle associated protein 1 [Source:HGNC Symbol;Acc:HGNC:18538] |
| **NCAPG** | 0.79 | 12.35 | | 0.E+00 | | 6 x 50 | | 4 p15 | | non-SMC condensin I complex, subunit G [Source:HGNC Symbol;Acc:HGNC:24304] |
| **CDCA5** | 0.78 | 11.95 | | 0.E+00 | | 4 x 50 | | 11 q13 | | cell division cycle associated 5 [Source:HGNC Symbol;Acc:HGNC:14626] |
| **TUBA1B** | 0.78 | 11.86 | | 0.E+00 | | 6 x 48 | | 12 q13 | | tubulin, alpha 1b [Source:HGNC Symbol;Acc:HGNC:18809] |
| **TYMS** | 0.78 | 11.85 | | 0.E+00 | | 3 x 50 | | 18 p11 | | thymidylate synthetase [Source:HGNC Symbol;Acc:HGNC:12441] |
| **NDC80** | 0.77 | 11.63 | | 0.E+00 | | 6 x 50 | | 18 p11 | | NDC80 kinetochore complex component [Source:HGNC Symbol;Acc:HGNC:16909] |
| **TRIP13** | 0.77 | 11.63 | | 0.E+00 | | 5 x 50 | | 5 p15 | | thyroid hormone receptor interactor 13 [Source:HGNC Symbol;Acc:HGNC:12307] |
| **SKA3** | 0.77 | 11.52 | | 0.E+00 | | 5 x 50 | | 13 q12 | | spindle and kinetochore associated complex subunit 3 [Source:HGNC Symbol;Acc:HGNC:20262] |
| **CKS1B** | 0.77 | 11.48 | | 0.E+00 | | 5 x 50 | | 1 q21 | | CDC28 protein kinase regulatory subunit 1B [Source:HGNC Symbol;Acc:HGNC:19083] |
| **CKAP2L** | 0.77 | 11.48 | | 0.E+00 | | 6 x 50 | | 2 q14 | | cytoskeleton associated protein 2-like [Source:HGNC Symbol;Acc:HGNC:26877] |
| **ANLN** | 0.76 | 11.25 | | 0.E+00 | | 5 x 50 | | 7 p14 | | anillin, actin binding protein [Source:HGNC Symbol;Acc:HGNC:14082] |
| **CENPF** | 0.76 | 11.18 | | 0.E+00 | | 6 x 50 | | 1 q41 | | centromere protein F, 350/400kDa [Source:HGNC Symbol;Acc:HGNC:1857] |
| **NUF2** | 0.76 | 11.18 | | 0.E+00 | | 6 x 50 | | 1 q23 | | NUF2, NDC80 kinetochore complex component [Source:HGNC Symbol;Acc:HGNC:14621] |
| **ATAD2** | 0.76 | 11.16 | | 0.E+00 | | 2 x 50 | | 8 q24 | | ATPase family, AAA domain containing 2 [Source:HGNC Symbol;Acc:HGNC:30123] |
| **SPC25** | 0.76 | 11.08 | | 0.E+00 | | 6 x 50 | | NA | | SPC25, NDC80 kinetochore complex component [Source:HGNC Symbol;Acc:HGNC:24031] |
| **BUB1** | 0.76 | 10.98 | | 0.E+00 | | 6 x 50 | | 2 q13 | | BUB1 mitotic checkpoint serine/threonine kinase [Source:HGNC Symbol;Acc:HGNC:1148] |
| **TK1** | 0.75 | 10.86 | | 0.E+00 | | 4 x 50 | | 17 q25 | | thymidine kinase 1, soluble [Source:HGNC Symbol;Acc:HGNC:11830] |
| **WDR76** | 0.75 | 10.83 | | 0.E+00 | | 1 x 50 | | 15 q15 | | WD repeat domain 76 [Source:HGNC Symbol;Acc:HGNC:25773] |
| **KIF18B** | 0.75 | 10.70 | | 0.E+00 | | 6 x 49 | | 17 q21 | | kinesin family member 18B [Source:HGNC Symbol;Acc:HGNC:27102] |
| **FANCD2** | 0.74 | 10.54 | | 0.E+00 | | 4 x 50 | | 3 p25 | | Fanconi anemia, complementation group D2 [Source:HGNC Symbol;Acc:HGNC:3585] |
| **CENPU** | 0.74 | 10.52 | | 0.E+00 | | 2 x 50 | | 4 q35 | | centromere protein U [Source:HGNC Symbol;Acc:HGNC:21348] |
| **FANCI** | 0.74 | 10.50 | | 0.E+00 | | 3 x 50 | | 15 q26 | | Fanconi anemia, complementation group I [Source:HGNC Symbol;Acc:HGNC:25568] |
| **NCAPH** | 0.74 | 10.40 | | 0.E+00 | | 5 x 50 | | 2 q11 | | non-SMC condensin I complex, subunit H [Source:HGNC Symbol;Acc:HGNC:1112] |
| **SHCBP1** | 0.74 | 10.40 | | 0.E+00 | | 5 x 50 | | 16 q11 | | SHC SH2-domain binding protein 1 [Source:HGNC Symbol;Acc:HGNC:29547] |
| **ESCO2** | 0.74 | 10.32 | | 0.E+00 | | 4 x 50 | | 8 p21 | | establishment of sister chromatid cohesion N-acetyltransferase 2 [Source:HGNC Symbol;Acc:HGNC:27230] |
| **SKA1** | 0.73 | 10.27 | | 0.E+00 | | 6 x 50 | | 18 q21 | | spindle and kinetochore associated complex subunit 1 [Source:HGNC Symbol;Acc:HGNC:28109] |
| **PBK** | 0.73 | 10.27 | | 0.E+00 | | 6 x 50 | | 8 p21 | | PDZ binding kinase [Source:HGNC Symbol;Acc:HGNC:18282] |
| **AURKB** | 0.73 | 10.23 | | 0.E+00 | | 6 x 50 | | 17 p13 | | aurora kinase B [Source:HGNC Symbol;Acc:HGNC:11390] |
| **ASPM** | 0.73 | 10.21 | | 0.E+00 | | 6 x 50 | | 1 q31 | | asp (abnormal spindle) homolog, microcephaly associated (Drosophila) [Source:HGNC Symbol;Acc:HGNC:19048] |
| **EXO1** | 0.73 | 10.11 | | 1.E-16 | | 1 x 50 | | 1 q43 | | exonuclease 1 [Source:HGNC Symbol;Acc:HGNC:3511] |
| **MKI67** | 0.72 | 9.88 | | 2.E-16 | | 6 x 50 | | 10 q26 | | marker of proliferation Ki-67 [Source:HGNC Symbol;Acc:HGNC:7107] |
| **KIF2C** | 0.72 | 9.88 | | 2.E-16 | | 6 x 50 | | 1 p34 | | kinesin family member 2C [Source:HGNC Symbol;Acc:HGNC:6393] |
| **MELK** | 0.72 | 9.86 | | 2.E-16 | | 5 x 50 | | 9 p13 | | maternal embryonic leucine zipper kinase [Source:HGNC Symbol;Acc:HGNC:16870] |
| **SPAG5** | 0.72 | 9.84 | | 3.E-16 | | 6 x 50 | | 17 q11 | | sperm associated antigen 5 [Source:HGNC Symbol;Acc:HGNC:13452] |
| **HJURP** | 0.72 | 9.83 | | 3.E-16 | | 6 x 50 | | 2 q37 | | Holliday junction recognition protein [Source:HGNC Symbol;Acc:HGNC:25444] |
| **FEN1** | 0.72 | 9.78 | | 4.E-16 | | 1 x 50 | | 11 q12 | | flap structure-specific endonuclease 1 [Source:HGNC Symbol;Acc:HGNC:3650] |
| **BIRC5** | 0.72 | 9.76 | | 4.E-16 | | 6 x 50 | | 17 q25 | | baculoviral IAP repeat containing 5 [Source:HGNC Symbol;Acc:HGNC:593] |
| **SGOL1** | 0.71 | 9.67 | | 7.E-16 | | 5 x 50 | | 3 p24 | | shugoshin-like 1 (S. pombe) [Source:HGNC Symbol;Acc:HGNC:25088] |
| **ZWINT** | 0.71 | 9.66 | | 7.E-16 | | 5 x 50 | | 10 q21 | | ZW10 interacting kinetochore protein [Source:HGNC Symbol;Acc:HGNC:13195] |
| **MCM7** | 0.71 | 9.55 | | 1.E-15 | | 2 x 50 | | 7 q22 | | minichromosome maintenance complex component 7 [Source:HGNC Symbol;Acc:HGNC:6950] |
| **KIF15** | 0.71 | 9.51 | | 2.E-15 | | 5 x 50 | | 3 p21 | | kinesin family member 15 [Source:HGNC Symbol;Acc:HGNC:17273] |
| **KIF11** | 0.70 | 9.43 | | 2.E-15 | | 6 x 50 | | 10 q23 | | kinesin family member 11 [Source:HGNC Symbol;Acc:HGNC:6388] |
| **BUB1B** | 0.70 | 9.39 | | 3.E-15 | | 6 x 50 | | 15 q15 | | BUB1 mitotic checkpoint serine/threonine kinase B [Source:HGNC Symbol;Acc:HGNC:1149] |
| **CENPN** | 0.70 | 9.39 | | 3.E-15 | | 4 x 50 | | 16 q23 | | centromere protein N [Source:HGNC Symbol;Acc:HGNC:30873] |
| **DTL** | 0.70 | 9.37 | | 3.E-15 | | 1 x 50 | | 1 q32 | | denticleless E3 ubiquitin protein ligase homolog (Drosophila) [Source:HGNC Symbol;Acc:HGNC:30288] |
| **RFC5** | 0.70 | 9.35 | | 3.E-15 | | 2 x 50 | | 12 q24 | | replication factor C (activator 1) 5, 36.5kDa [Source:HGNC Symbol;Acc:HGNC:9973] |
| **BARD1** | 0.70 | 9.34 | | 3.E-15 | | 2 x 50 | | 2 q35 | | BRCA1 associated RING domain 1 [Source:HGNC Symbol;Acc:HGNC:952] |
| **CENPK** | 0.70 | 9.31 | | 4.E-15 | | 3 x 50 | | 5 q12 | | centromere protein K [Source:HGNC Symbol;Acc:HGNC:29479] |
| **CLSPN** | 0.70 | 9.30 | | 4.E-15 | | 1 x 50 | | 1 p34 | | claspin [Source:HGNC Symbol;Acc:HGNC:19715] |
| **TPX2** | 0.70 | 9.26 | | 5.E-15 | | 6 x 50 | | 20 q11 | | TPX2, microtubule-associated [Source:HGNC Symbol;Acc:HGNC:1249] |
| **CDCA3** | 0.70 | 9.25 | | 5.E-15 | | 6 x 50 | | 12 p13 | | cell division cycle associated 3 [Source:HGNC Symbol;Acc:HGNC:14624] |
| **DIAPH3** | 0.70 | 9.23 | | 6.E-15 | | 6 x 50 | | 13 q21 | | diaphanous-related formin 3 [Source:HGNC Symbol;Acc:HGNC:15480] |
| **SMC4** | 0.70 | 9.21 | | 6.E-15 | | 5 x 50 | | 3 q25 | | structural maintenance of chromosomes 4 [Source:HGNC Symbol;Acc:HGNC:14013] |
| **CCNB2** | 0.70 | 9.20 | | 7.E-15 | | 6 x 50 | | 15 q22 | | cyclin B2 [Source:HGNC Symbol;Acc:HGNC:1580] |
| **UHRF1** | 0.69 | 9.16 | | 8.E-15 | | 1 x 50 | | 19 p13 | | ubiquitin-like with PHD and ring finger domains 1 [Source:HGNC Symbol;Acc:HGNC:12556] |
| **GINS2** | 0.69 | 9.15 | | 8.E-15 | | 1 x 50 | | 16 q24 | | GINS complex subunit 2 (Psf2 homolog) [Source:HGNC Symbol;Acc:HGNC:24575] |
| **GMNN** | 0.69 | 9.15 | | 8.E-15 | | 2 x 50 | | 6 p22 | | geminin, DNA replication inhibitor [Source:HGNC Symbol;Acc:HGNC:17493] |
| **CDCA8** | 0.69 | 9.10 | | 1.E-14 | | 6 x 50 | | 1 p34 | | cell division cycle associated 8 [Source:HGNC Symbol;Acc:HGNC:14629] |
| **RFC3** | 0.69 | 9.09 | | 1.E-14 | | 2 x 50 | | 13 q13 | | replication factor C (activator 1) 3, 38kDa [Source:HGNC Symbol;Acc:HGNC:9971] |
| **HELLS** | 0.69 | 9.06 | | 1.E-14 | | 1 x 50 | | 10 q23 | | helicase, lymphoid-specific [Source:HGNC Symbol;Acc:HGNC:4861] |
| **TMPO** | 0.69 | 8.98 | | 2.E-14 | | 5 x 50 | | 12 q23 | | thymopoietin [Source:HGNC Symbol;Acc:HGNC:11875] |
| **PCNA** | 0.69 | 8.96 | | 2.E-14 | | 2 x 50 | | 20 p12 | | proliferating cell nuclear antigen [Source:HGNC Symbol;Acc:HGNC:8729] |
| **POLQ** | 0.68 | 8.77 | | 5.E-14 | | 3 x 50 | | 3 q13 | | polymerase (DNA directed), theta [Source:HGNC Symbol;Acc:HGNC:9186] |
| **CDC6** | 0.68 | 8.74 | | 6.E-14 | | 1 x 50 | | 17 q21 | | cell division cycle 6 [Source:HGNC Symbol;Acc:HGNC:1744] |
| **FBXO5** | 0.68 | 8.72 | | 7.E-14 | | 4 x 50 | | 6 q25 | | F-box protein 5 [Source:HGNC Symbol;Acc:HGNC:13584] |
| **CCNA2** | 0.68 | 8.71 | | 7.E-14 | | 6 x 50 | | 4 q27 | | cyclin A2 [Source:HGNC Symbol;Acc:HGNC:1578] |
| **STMN1** | 0.67 | 8.60 | | 1.E-13 | | 5 x 50 | | 1 p36 | | stathmin 1 [Source:HGNC Symbol;Acc:HGNC:6510] |
| **H2AFZ** | 0.67 | 8.57 | | 1.E-13 | | 7 x 48 | | 4 q23 | | H2A histone family, member Z [Source:HGNC Symbol;Acc:HGNC:4741] |
| **TACC3** | 0.67 | 8.50 | | 2.E-13 | | 6 x 50 | | 4 p16 | | transforming, acidic coiled-coil containing protein 3 [Source:HGNC Symbol;Acc:HGNC:11524] |
| **CCNE2** | 0.66 | 8.42 | | 3.E-13 | | 2 x 50 | | 8 q22 | | cyclin E2 [Source:HGNC Symbol;Acc:HGNC:1590] |
| **DEK** | 0.66 | 8.33 | | 4.E-13 | | 2 x 50 | | 6 p22 | | DEK proto-oncogene [Source:HGNC Symbol;Acc:HGNC:2768] |
| **MMS22L** | 0.66 | 8.30 | | 5.E-13 | | 2 x 50 | | 6 q16 | | MMS22-like, DNA repair protein [Source:HGNC Symbol;Acc:HGNC:21475] |
| **ANP32E** | 0.66 | 8.30 | | 5.E-13 | | 8 x 50 | | 1 q21 | | acidic (leucine-rich) nuclear phosphoprotein 32 family, member E [Source:HGNC Symbol;Acc:HGNC:16673] |
| **PLK4** | 0.66 | 8.26 | | 6.E-13 | | 5 x 50 | | 4 q28 | | polo-like kinase 4 [Source:HGNC Symbol;Acc:HGNC:11397] |
| **NEIL3** | 0.65 | 8.19 | | 8.E-13 | | 7 x 50 | | 4 q34 | | nei endonuclease VIII-like 3 (E. coli) [Source:HGNC Symbol;Acc:HGNC:24573] |
| **KIAA1524** | 0.65 | 8.17 | | 9.E-13 | | 6 x 50 | | 3 q13 | | KIAA1524 [Source:HGNC Symbol;Acc:HGNC:29302] |
| **DEPDC1** | 0.65 | 8.12 | | 1.E-12 | | 6 x 50 | | 1 p31 | | DEP domain containing 1 [Source:HGNC Symbol;Acc:HGNC:22949] |
| **CDC45** | 0.65 | 8.08 | | 1.E-12 | | 1 x 50 | | 22 q11 | | cell division cycle 45 [Source:HGNC Symbol;Acc:HGNC:1739] |
| **CDKN3** | 0.65 | 8.07 | | 1.E-12 | | 6 x 50 | | 14 q22 | | cyclin-dependent kinase inhibitor 3 [Source:HGNC Symbol;Acc:HGNC:1791] |
| **RRM1** | 0.65 | 8.06 | | 2.E-12 | | 2 x 50 | | 11 p15 | | ribonucleotide reductase M1 [Source:HGNC Symbol;Acc:HGNC:10451] |
| **CENPI** | 0.64 | 8.00 | | 2.E-12 | | 6 x 50 | | X q22 | | centromere protein I [Source:HGNC Symbol;Acc:HGNC:3968] |
| **BUB3** | 0.64 | 7.99 | | 2.E-12 | | 7 x 48 | | 10 q26 | | BUB3 mitotic checkpoint protein [Source:HGNC Symbol;Acc:HGNC:1151] |
| **FANCB** | 0.64 | 7.97 | | 2.E-12 | | 2 x 50 | | X p22 | | Fanconi anemia, complementation group B [Source:HGNC Symbol;Acc:HGNC:3583] |
| **CENPQ** | 0.64 | 7.95 | | 3.E-12 | | 2 x 50 | | 6 p12 | | centromere protein Q [Source:HGNC Symbol;Acc:HGNC:21347] |
| **CASC5** | 0.64 | 7.94 | | 3.E-12 | | 6 x 50 | | 15 q15 | | cancer susceptibility candidate 5 [Source:HGNC Symbol;Acc:HGNC:24054] |
| **ATAD5** | 0.64 | 7.90 | | 3.E-12 | | 1 x 50 | | 17 q11 | | ATPase family, AAA domain containing 5 [Source:HGNC Symbol;Acc:HGNC:25752] |
| **CENPW** | 0.64 | 7.90 | | 3.E-12 | | 6 x 50 | | 6 q22 | | centromere protein W [Source:HGNC Symbol;Acc:HGNC:21488] |
| **MCM10** | 0.64 | 7.85 | | 4.E-12 | | 1 x 50 | | 10 p13 | | minichromosome maintenance complex component 10 [Source:HGNC Symbol;Acc:HGNC:18043] |
| **POLD3** | 0.64 | 7.81 | | 5.E-12 | | 2 x 50 | | 11 q13 | | polymerase (DNA-directed), delta 3, accessory subunit [Source:HGNC Symbol;Acc:HGNC:20932] |
| **PARPBP** | 0.63 | 7.78 | | 6.E-12 | | 6 x 50 | | 12 q23 | | PARP1 binding protein [Source:HGNC Symbol;Acc:HGNC:26074] |
| **VRK1** | 0.63 | 7.78 | | 6.E-12 | | 3 x 50 | | 14 q32 | | vaccinia related kinase 1 [Source:HGNC Symbol;Acc:HGNC:12718] |
| **ECT2** | 0.63 | 7.78 | | 6.E-12 | | 6 x 50 | | 3 q26 | | epithelial cell transforming 2 [Source:HGNC Symbol;Acc:HGNC:3155] |
| **HIST1H1A** | 0.63 | 7.72 | | 8.E-12 | | 5 x 49 | | 6 p22 | | histone cluster 1, H1a [Source:HGNC Symbol;Acc:HGNC:4715] |
| **PKMYT1** | 0.63 | 7.62 | | 1.E-11 | | 1 x 50 | | 16 p13 | | protein kinase, membrane associated tyrosine/threonine 1 [Source:HGNC Symbol;Acc:HGNC:29650] |
| **CEP152** | 0.63 | 7.61 | | 1.E-11 | | 4 x 50 | | 15 q21 | | centrosomal protein 152kDa [Source:HGNC Symbol;Acc:HGNC:29298] |
| **HIST1H3B** | 0.63 | 7.61 | | 1.E-11 | | 7 x 48 | | 6 p22 | | histone cluster 1, H3b [Source:HGNC Symbol;Acc:HGNC:4776] |
| **CKS2** | 0.62 | 7.58 | | 1.E-11 | | 5 x 50 | | 9 q22 | | CDC28 protein kinase regulatory subunit 2 [Source:HGNC Symbol;Acc:HGNC:2000] |
| **TCF19** | 0.62 | 7.57 | | 2.E-11 | | 4 x 47 | | NA | | transcription factor 19 [Source:HGNC Symbol;Acc:HGNC:11629] |
| **KIF23** | 0.62 | 7.50 | | 2.E-11 | | 6 x 50 | | 15 q23 | | kinesin family member 23 [Source:HGNC Symbol;Acc:HGNC:6392] |
| **CEP55** | 0.62 | 7.49 | | 2.E-11 | | 6 x 50 | | 10 q23 | | centrosomal protein 55kDa [Source:HGNC Symbol;Acc:HGNC:1161] |
| **USP1** | 0.62 | 7.42 | | 3.E-11 | | 2 x 50 | | 1 p31 | | ubiquitin specific peptidase 1 [Source:HGNC Symbol;Acc:HGNC:12607] |
| **SMC2** | 0.62 | 7.42 | | 3.E-11 | | 5 x 50 | | 9 q31 | | structural maintenance of chromosomes 2 [Source:HGNC Symbol;Acc:HGNC:14011] |
| **GTSE1** | 0.61 | 7.40 | | 4.E-11 | | 7 x 50 | | 22 q13 | | G-2 and S-phase expressed 1 [Source:HGNC Symbol;Acc:HGNC:13698] |
| **DSN1** | 0.61 | 7.38 | | 4.E-11 | | 2 x 50 | | 20 q11 | | DSN1, MIS12 kinetochore complex component [Source:HGNC Symbol;Acc:HGNC:16165] |
| **BRCA2** | 0.61 | 7.37 | | 4.E-11 | | 2 x 50 | | 13 q13 | | breast cancer 2, early onset [Source:HGNC Symbol;Acc:HGNC:1101] |
| **CCDC150** | 0.61 | 7.37 | | 4.E-11 | | 5 x 50 | | 2 q33 | | coiled-coil domain containing 150 [Source:HGNC Symbol;Acc:HGNC:26834] |
| **E2F7** | 0.61 | 7.37 | | 4.E-11 | | 2 x 50 | | 12 q21 | | E2F transcription factor 7 [Source:HGNC Symbol;Acc:HGNC:23820] |
| **E2F8** | 0.61 | 7.29 | | 6.E-11 | | 3 x 49 | | 11 p15 | | E2F transcription factor 8 [Source:HGNC Symbol;Acc:HGNC:24727] |
| **MYBL2** | 0.61 | 7.28 | | 6.E-11 | | 4 x 50 | | 20 q13 | | v-myb avian myeloblastosis viral oncogene homolog-like 2 [Source:HGNC Symbol;Acc:HGNC:7548] |
| **CSE1L** | 0.61 | 7.26 | | 7.E-11 | | 3 x 49 | | 20 q13 | | CSE1 chromosome segregation 1-like (yeast) [Source:HGNC Symbol;Acc:HGNC:2431] |
| **HIST1H3C** | 0.61 | 7.26 | | 7.E-11 | | 6 x 48 | | 6 p22 | | histone cluster 1, H3c [Source:HGNC Symbol;Acc:HGNC:4768] |
| **ESPL1** | 0.61 | 7.25 | | 7.E-11 | | 7 x 49 | | 12 q13 | | extra spindle pole bodies homolog 1 (S. cerevisiae) [Source:HGNC Symbol;Acc:HGNC:16856] |
| **TROAP** | 0.61 | 7.25 | | 7.E-11 | | 6 x 50 | | 12 q13 | | trophinin associated protein [Source:HGNC Symbol;Acc:HGNC:12327] |
| **DNMT1** | 0.61 | 7.22 | | 8.E-11 | | 2 x 48 | | 19 p13 | | DNA (cytosine-5-)-methyltransferase 1 [Source:HGNC Symbol;Acc:HGNC:2976] |
| **KPNA2** | 0.60 | 7.09 | | 1.E-10 | | 7 x 50 | | 17 q24 | | karyopherin alpha 2 (RAG cohort 1, importin alpha 1) [Source:HGNC Symbol;Acc:HGNC:6395] |
| **CKAP2** | 0.60 | 7.06 | | 2.E-10 | | 6 x 50 | | 13 q14 | | cytoskeleton associated protein 2 [Source:HGNC Symbol;Acc:HGNC:1990] |
| **LMNB1** | 0.60 | 7.06 | | 2.E-10 | | 1 x 50 | | 5 q23 | | lamin B1 [Source:HGNC Symbol;Acc:HGNC:6637] |
| **RHNO1** | 0.60 | 7.03 | | 2.E-10 | | 5 x 50 | | 12 p13 | | RAD9-HUS1-RAD1 interacting nuclear orphan 1 [Source:HGNC Symbol;Acc:HGNC:28206] |
| **DNAJC9** | 0.59 | 6.97 | | 3.E-10 | | 1 x 50 | | 10 q22 | | DnaJ (Hsp40) homolog, subfamily C, member 9 [Source:HGNC Symbol;Acc:HGNC:19123] |
| **IQGAP3** | 0.59 | 6.93 | | 3.E-10 | | 8 x 50 | | 1 q22 | | IQ motif containing GTPase activating protein 3 [Source:HGNC Symbol;Acc:HGNC:20669] |
| **CDC20** | 0.59 | 6.93 | | 3.E-10 | | 6 x 50 | | 1 p34 | | cell division cycle 20 [Source:HGNC Symbol;Acc:HGNC:1723] |
| **TTK** | 0.59 | 6.86 | | 4.E-10 | | 6 x 50 | | 6 q14 | | TTK protein kinase [Source:HGNC Symbol;Acc:HGNC:12401] |
| **CENPJ** | 0.58 | 6.81 | | 5.E-10 | | 2 x 50 | | 13 q12 | | centromere protein J [Source:HGNC Symbol;Acc:HGNC:17272] |
| **FOXM1** | 0.58 | 6.81 | | 5.E-10 | | 7 x 50 | | 12 p13 | | forkhead box M1 [Source:HGNC Symbol;Acc:HGNC:3818] |
| **BLM** | 0.58 | 6.78 | | 6.E-10 | | 4 x 50 | | 15 q26 | | Bloom syndrome, RecQ helicase-like [Source:HGNC Symbol;Acc:HGNC:1058] |
| **KIF20B** | 0.58 | 6.69 | | 9.E-10 | | 6 x 50 | | 10 q23 | | kinesin family member 20B [Source:HGNC Symbol;Acc:HGNC:7212] |
| **MASTL** | 0.58 | 6.68 | | 1.E-09 | | 5 x 48 | | 10 p12 | | microtubule associated serine/threonine kinase-like [Source:HGNC Symbol;Acc:HGNC:19042] |
| **CHAF1A** | 0.57 | 6.65 | | 1.E-09 | | 1 x 50 | | 19 p13 | | chromatin assembly factor 1, subunit A (p150) [Source:HGNC Symbol;Acc:HGNC:1910] |
| **MCM3** | 0.57 | 6.65 | | 1.E-09 | | 1 x 50 | | 6 p12 | | minichromosome maintenance complex component 3 [Source:HGNC Symbol;Acc:HGNC:6945] |
| **CIT** | 0.57 | 6.62 | | 1.E-09 | | 7 x 50 | | 12 q24 | | citron rho-interacting serine/threonine kinase [Source:HGNC Symbol;Acc:HGNC:1985] |
| **TRAIP** | 0.57 | 6.62 | | 1.E-09 | | 5 x 50 | | 3 p21 | | TRAF interacting protein [Source:HGNC Symbol;Acc:HGNC:30764] |
| **HMMR** | 0.57 | 6.61 | | 1.E-09 | | 7 x 50 | | 5 q34 | | hyaluronan-mediated motility receptor (RHAMM) [Source:HGNC Symbol;Acc:HGNC:5012] |
| **C1orf112** | 0.57 | 6.61 | | 1.E-09 | | 6 x 50 | | 1 q24 | | chromosome 1 open reading frame 112 [Source:HGNC Symbol;Acc:HGNC:25565] |
| **DLGAP5** | 0.57 | 6.60 | | 1.E-09 | | 7 x 50 | | 14 q22 | | discs, large (Drosophila) homolog-associated protein 5 [Source:HGNC Symbol;Acc:HGNC:16864] |
| **LIG1** | 0.57 | 6.57 | | 2.E-09 | | 2 x 50 | | 19 q13 | | ligase I, DNA, ATP-dependent [Source:HGNC Symbol;Acc:HGNC:6598] |
| **EZH2** | 0.57 | 6.57 | | 2.E-09 | | 2 x 50 | | 7 q36 | | enhancer of zeste 2 polycomb repressive complex 2 subunit [Source:HGNC Symbol;Acc:HGNC:3527] |
| **HAUS8** | 0.57 | 6.57 | | 2.E-09 | | 3 x 50 | | 19 p13 | | HAUS augmin-like complex, subunit 8 [Source:HGNC Symbol;Acc:HGNC:30532] |
| **FAM111A** | 0.57 | 6.56 | | 2.E-09 | | 4 x 50 | | 11 q12 | | family with sequence similarity 111, member A [Source:HGNC Symbol;Acc:HGNC:24725] |
| **FAM64A** | 0.57 | 6.53 | | 2.E-09 | | 7 x 50 | | 17 p13 | | family with sequence similarity 64, member A [Source:HGNC Symbol;Acc:HGNC:25483] |
| **GINS1** | 0.56 | 6.48 | | 2.E-09 | | 2 x 49 | | 20 p11 | | GINS complex subunit 1 (Psf1 homolog) [Source:HGNC Symbol;Acc:HGNC:28980] |
| **RNASEH2A** | 0.56 | 6.47 | | 2.E-09 | | 4 x 50 | | 19 p13 | | ribonuclease H2, subunit A [Source:HGNC Symbol;Acc:HGNC:18518] |
| **KIF18A** | 0.56 | 6.43 | | 3.E-09 | | 7 x 50 | | 11 p14 | | kinesin family member 18A [Source:HGNC Symbol;Acc:HGNC:29441] |
| **PHF19** | 0.56 | 6.43 | | 3.E-09 | | 5 x 50 | | 9 q33 | | PHD finger protein 19 [Source:HGNC Symbol;Acc:HGNC:24566] |
| **KIF14** | 0.56 | 6.40 | | 3.E-09 | | 7 x 50 | | 1 q32 | | kinesin family member 14 [Source:HGNC Symbol;Acc:HGNC:19181] |
| **SGOL2** | 0.56 | 6.38 | | 4.E-09 | | 7 x 50 | | 2 q33 | | shugoshin-like 2 (S. pombe) [Source:HGNC Symbol;Acc:HGNC:30812] |
| **MCM4** | 0.56 | 6.37 | | 4.E-09 | | 1 x 50 | | 8 q11 | | minichromosome maintenance complex component 4 [Source:HGNC Symbol;Acc:HGNC:6947] |
| **RAD54L** | 0.56 | 6.35 | | 4.E-09 | | 4 x 50 | | 1 p34 | | RAD54-like (S. cerevisiae) [Source:HGNC Symbol;Acc:HGNC:9826] |
| **HMGN2** | 0.55 | 6.33 | | 5.E-09 | | 8 x 50 | | 1 p36 | | high mobility group nucleosomal binding domain 2 [Source:HGNC Symbol;Acc:HGNC:4986] |
| **ORC6** | 0.55 | 6.31 | | 5.E-09 | | 1 x 50 | | 16 q11 | | origin recognition complex, subunit 6 [Source:HGNC Symbol;Acc:HGNC:17151] |
| **GGCT** | 0.55 | 6.30 | | 5.E-09 | | 1 x 47 | | 7 p14 | | gamma-glutamylcyclotransferase [Source:HGNC Symbol;Acc:HGNC:21705] |
| **CDCA2** | 0.55 | 6.29 | | 6.E-09 | | 7 x 50 | | 8 p21 | | cell division cycle associated 2 [Source:HGNC Symbol;Acc:HGNC:14623] |
| **TUBG1** | 0.55 | 6.28 | | 6.E-09 | | 2 x 49 | | 17 q21 | | tubulin, gamma 1 [Source:HGNC Symbol;Acc:HGNC:12417] |
| **KIF22** | 0.55 | 6.27 | | 6.E-09 | | 6 x 50 | | 16 p11 | | kinesin family member 22 [Source:HGNC Symbol;Acc:HGNC:6391] |
| **CENPE** | 0.55 | 6.25 | | 7.E-09 | | 7 x 50 | | 4 q24 | | centromere protein E, 312kDa [Source:HGNC Symbol;Acc:HGNC:1856] |
| **SMC1A** | 0.55 | 6.23 | | 7.E-09 | | 3 x 48 | | X p11 | | structural maintenance of chromosomes 1A [Source:HGNC Symbol;Acc:HGNC:11111] |
| **HSPB11** | 0.55 | 6.21 | | 8.E-09 | | 1 x 48 | | 1 p32 | | heat shock protein family B (small), member 11 [Source:HGNC Symbol;Acc:HGNC:25019] |
| **SAE1** | 0.55 | 6.20 | | 8.E-09 | | 4 x 47 | | 19 q13 | | SUMO1 activating enzyme subunit 1 [Source:HGNC Symbol;Acc:HGNC:30660] |
| **GEN1** | 0.55 | 6.19 | | 9.E-09 | | 5 x 50 | | 2 p24 | | GEN1 Holliday junction 5' flap endonuclease [Source:HGNC Symbol;Acc:HGNC:26881] |
| **PRIM1** | 0.55 | 6.18 | | 9.E-09 | | 1 x 50 | | 12 q13 | | primase, DNA, polypeptide 1 (49kDa) [Source:HGNC Symbol;Acc:HGNC:9369] |
| **PSMC3IP** | 0.55 | 6.18 | | 9.E-09 | | 1 x 50 | | 17 q21 | | PSMC3 interacting protein [Source:HGNC Symbol;Acc:HGNC:17928] |
| **C5orf34** | 0.55 | 6.18 | | 9.E-09 | | 6 x 50 | | 5 p12 | | chromosome 5 open reading frame 34 [Source:HGNC Symbol;Acc:HGNC:24738] |
| **MNS1** | 0.54 | 6.15 | | 1.E-08 | | 7 x 49 | | 15 q21 | | meiosis-specific nuclear structural 1 [Source:HGNC Symbol;Acc:HGNC:29636] |
| **WEE1** | 0.54 | 6.13 | | 1.E-08 | | 2 x 50 | | 11 p15 | | WEE1 G2 checkpoint kinase [Source:HGNC Symbol;Acc:HGNC:12761] |
| **CENPM** | 0.54 | 6.12 | | 1.E-08 | | 3 x 50 | | 22 q13 | | centromere protein M [Source:HGNC Symbol;Acc:HGNC:18352] |
| **ZNF367** | 0.54 | 6.09 | | 1.E-08 | | 1 x 49 | | 9 q22 | | zinc finger protein 367 [Source:HGNC Symbol;Acc:HGNC:18320] |
| **E2F2** | 0.54 | 6.09 | | 1.E-08 | | 1 x 48 | | 1 p36 | | E2F transcription factor 2 [Source:HGNC Symbol;Acc:HGNC:3114] |
| **BRIP1** | 0.54 | 6.06 | | 2.E-08 | | 1 x 50 | | 17 q23 | | BRCA1 interacting protein C-terminal helicase 1 [Source:HGNC Symbol;Acc:HGNC:20473] |
| **RFC4** | 0.54 | 6.04 | | 2.E-08 | | 1 x 50 | | 3 q27 | | replication factor C (activator 1) 4, 37kDa [Source:HGNC Symbol;Acc:HGNC:9972] |
| **PRR11** | 0.53 | 5.98 | | 2.E-08 | | 7 x 50 | | 17 q22 | | proline rich 11 [Source:HGNC Symbol;Acc:HGNC:25619] |
| **CHAF1B** | 0.53 | 5.97 | | 2.E-08 | | 1 x 50 | | 21 q22 | | chromatin assembly factor 1, subunit B (p60) [Source:HGNC Symbol;Acc:HGNC:1911] |
| **KNTC1** | 0.53 | 5.96 | | 2.E-08 | | 1 x 50 | | 12 q24 | | kinetochore associated 1 [Source:HGNC Symbol;Acc:HGNC:17255] |
| **WHSC1** | 0.53 | 5.94 | | 3.E-08 | | 4 x 50 | | 4 p16 | | Wolf-Hirschhorn syndrome candidate 1 [Source:HGNC Symbol;Acc:HGNC:12766] |
| **DNA2** | 0.53 | 5.93 | | 3.E-08 | | 2 x 50 | | 10 q21 | | DNA replication helicase/nuclease 2 [Source:HGNC Symbol;Acc:HGNC:2939] |
| **CCP110** | 0.53 | 5.89 | | 3.E-08 | | 3 x 50 | | 16 p12 | | centriolar coiled coil protein 110kDa [Source:HGNC Symbol;Acc:HGNC:24342] |
| **DSCC1** | 0.53 | 5.86 | | 4.E-08 | | 1 x 50 | | 8 q24 | | DNA replication and sister chromatid cohesion 1 [Source:HGNC Symbol;Acc:HGNC:24453] |
| **RFC2** | 0.53 | 5.86 | | 4.E-08 | | 1 x 50 | | 7 q11 | | replication factor C (activator 1) 2, 40kDa [Source:HGNC Symbol;Acc:HGNC:9970] |
| **MND1** | 0.52 | 5.82 | | 4.E-08 | | 1 x 50 | | 4 q31 | | meiotic nuclear divisions 1 homolog (S. cerevisiae) [Source:HGNC Symbol;Acc:HGNC:24839] |
| **OIP5** | 0.52 | 5.75 | | 6.E-08 | | 7 x 50 | | 15 q15 | | Opa interacting protein 5 [Source:HGNC Symbol;Acc:HGNC:20300] |
| **FANCA** | 0.52 | 5.73 | | 7.E-08 | | 1 x 50 | | 16 q24 | | Fanconi anemia, complementation group A [Source:HGNC Symbol;Acc:HGNC:3582] |
| **SKP2** | 0.52 | 5.72 | | 7.E-08 | | 3 x 50 | | 5 p13 | | S-phase kinase-associated protein 2, E3 ubiquitin protein ligase [Source:HGNC Symbol;Acc:HGNC:10901] |
| **RAD51** | 0.52 | 5.71 | | 7.E-08 | | 1 x 50 | | 15 q15 | | RAD51 recombinase [Source:HGNC Symbol;Acc:HGNC:9817] |
| **RBL1** | 0.52 | 5.71 | | 7.E-08 | | 1 x 50 | | 20 q11 | | retinoblastoma-like 1 [Source:HGNC Symbol;Acc:HGNC:9893] |
| **MTHFD2** | 0.51 | 5.69 | | 8.E-08 | | 3 x 47 | | 2 p13 | | methylenetetrahydrofolate dehydrogenase (NADP+ dependent) 2, methenyltetrahydrofolate cyclohydrolase [Source:HGNC Symbol;Acc:HGNC:7434] |
| **FAM83D** | 0.51 | 5.69 | | 8.E-08 | | 7 x 50 | | 20 q11 | | family with sequence similarity 83, member D [Source:HGNC Symbol;Acc:HGNC:16122] |
| **MIS18A** | 0.51 | 5.67 | | 9.E-08 | | 3 x 49 | | 21 q22 | | MIS18 kinetochore protein A [Source:HGNC Symbol;Acc:HGNC:1286] |
| **KDELC2** | 0.51 | 5.66 | | 9.E-08 | | 3 x 49 | | 11 q22 | | KDEL (Lys-Asp-Glu-Leu) containing 2 [Source:HGNC Symbol;Acc:HGNC:28496] |
| **NUP50** | 0.51 | 5.66 | | 9.E-08 | | 5 x 47 | | 22 q13 | | nucleoporin 50kDa [Source:HGNC Symbol;Acc:HGNC:8065] |
| **ORC1** | 0.51 | 5.64 | | 1.E-07 | | 1 x 49 | | 1 p32 | | origin recognition complex, subunit 1 [Source:HGNC Symbol;Acc:HGNC:8487] |
| **DCK** | 0.51 | 5.64 | | 1.E-07 | | 2 x 50 | | 4 q13 | | deoxycytidine kinase [Source:HGNC Symbol;Acc:HGNC:2704] |
| **PTTG1** | 0.51 | 5.64 | | 1.E-07 | | 7 x 50 | | 5 q33 | | pituitary tumor-transforming 1 [Source:HGNC Symbol;Acc:HGNC:9690] |
| **LBR** | 0.51 | 5.63 | | 1.E-07 | | 7 x 50 | | 1 q42 | | lamin B receptor [Source:HGNC Symbol;Acc:HGNC:6518] |
| **APOBEC3B** | 0.51 | 5.62 | | 1.E-07 | | 6 x 50 | | 22 q13 | | apolipoprotein B mRNA editing enzyme, catalytic polypeptide-like 3B [Source:HGNC Symbol;Acc:HGNC:17352] |
| **CD83** | 0.51 | 5.61 | | 1.E-07 | | 1 x 50 | | 6 p23 | | CD83 molecule [Source:HGNC Symbol;Acc:HGNC:1703] |
| **LRR1** | 0.51 | 5.60 | | 1.E-07 | | 4 x 49 | | 14 q21 | | leucine rich repeat protein 1 [Source:HGNC Symbol;Acc:HGNC:19742] |
| **TMEM97** | 0.51 | 5.59 | | 1.E-07 | | 1 x 48 | | 17 q11 | | transmembrane protein 97 [Source:HGNC Symbol;Acc:HGNC:28106] |
| **RACGAP1** | 0.51 | 5.58 | | 1.E-07 | | 7 x 50 | | 12 q13 | | Rac GTPase activating protein 1 [Source:HGNC Symbol;Acc:HGNC:9804] |
| **MSH2** | 0.50 | 5.55 | | 1.E-07 | | 1 x 49 | | 2 p21 | | mutS homolog 2 [Source:HGNC Symbol;Acc:HGNC:7325] |
| **NFYB** | 0.50 | 5.55 | | 1.E-07 | | 5 x 50 | | 12 q23 | | nuclear transcription factor Y, beta [Source:HGNC Symbol;Acc:HGNC:7805] |
| **FKBP5** | 0.50 | 5.54 | | 1.E-07 | | 4 x 47 | | 6 p21 | | FK506 binding protein 5 [Source:HGNC Symbol;Acc:HGNC:3721] |
| **CENPL** | 0.50 | 5.54 | | 1.E-07 | | 6 x 50 | | 1 q25 | | centromere protein L [Source:HGNC Symbol;Acc:HGNC:17879] |
| **CEP192** | 0.50 | 5.51 | | 2.E-07 | | 5 x 49 | | 18 p11 | | centrosomal protein 192kDa [Source:HGNC Symbol;Acc:HGNC:25515] |
| **CTNNAL1** | 0.50 | 5.47 | | 2.E-07 | | 1 x 50 | | 9 q31 | | catenin (cadherin-associated protein), alpha-like 1 [Source:HGNC Symbol;Acc:HGNC:2512] |
| **DUT** | 0.50 | 5.45 | | 2.E-07 | | 1 x 48 | | 15 q21 | | deoxyuridine triphosphatase [Source:HGNC Symbol;Acc:HGNC:3078] |
| **KIF4A** | 0.50 | 5.42 | | 2.E-07 | | 7 x 50 | | X q13 | | kinesin family member 4A [Source:HGNC Symbol;Acc:HGNC:13339] |
| **CKLF** | 0.50 | 5.42 | | 2.E-07 | | 3 x 49 | | 16 q21 | | chemokine-like factor [Source:HGNC Symbol;Acc:HGNC:13253] |
| **CCNB1** | 0.50 | 5.41 | | 3.E-07 | | 7 x 50 | | 5 q13 | | cyclin B1 [Source:HGNC Symbol;Acc:HGNC:1579] |
| **TUBB4B** | 0.50 | 5.41 | | 3.E-07 | | 7 x 50 | | 9 q34 | | tubulin, beta 4B class IVb [Source:HGNC Symbol;Acc:HGNC:20771] |
| **HIRIP3** | 0.49 | 5.39 | | 3.E-07 | | 2 x 50 | | 16 p11 | | HIRA interacting protein 3 [Source:HGNC Symbol;Acc:HGNC:4917] |
| **MCM6** | 0.49 | 5.38 | | 3.E-07 | | 1 x 50 | | 2 q21 | | minichromosome maintenance complex component 6 [Source:HGNC Symbol;Acc:HGNC:6949] |
| **KIF20A** | 0.49 | 5.36 | | 3.E-07 | | 7 x 50 | | 5 q31 | | kinesin family member 20A [Source:HGNC Symbol;Acc:HGNC:9787] |
| **GPD2** | 0.49 | 5.36 | | 3.E-07 | | 1 x 50 | | 2 q24 | | glycerol-3-phosphate dehydrogenase 2 (mitochondrial) [Source:HGNC Symbol;Acc:HGNC:4456] |
| **CEP128** | 0.49 | 5.31 | | 4.E-07 | | 3 x 48 | | 14 q31 | | centrosomal protein 128kDa [Source:HGNC Symbol;Acc:HGNC:20359] |
| **MCM5** | 0.49 | 5.30 | | 4.E-07 | | 1 x 49 | | 22 q12 | | minichromosome maintenance complex component 5 [Source:HGNC Symbol;Acc:HGNC:6948] |
| **TOPBP1** | 0.49 | 5.29 | | 4.E-07 | | 2 x 50 | | 3 q22 | | topoisomerase (DNA) II binding protein 1 [Source:HGNC Symbol;Acc:HGNC:17008] |
| **TMEM194A** | 0.49 | 5.27 | | 5.E-07 | | 4 x 48 | | 12 q13 | | transmembrane protein 194A [Source:HGNC Symbol;Acc:HGNC:29001] |
| **CHEK1** | 0.48 | 5.25 | | 5.E-07 | | 1 x 50 | | 11 q24 | | checkpoint kinase 1 [Source:HGNC Symbol;Acc:HGNC:1925] |
| **NCAPG2** | 0.48 | 5.24 | | 5.E-07 | | 6 x 48 | | 7 q36 | | non-SMC condensin II complex, subunit G2 [Source:HGNC Symbol;Acc:HGNC:21904] |
| **TIFA** | 0.48 | 5.23 | | 5.E-07 | | 3 x 47 | | 4 q25 | | TRAF-interacting protein with forkhead-associated domain [Source:HGNC Symbol;Acc:HGNC:19075] |
| **MIS18BP1** | 0.48 | 5.23 | | 6.E-07 | | 6 x 50 | | 14 q21 | | MIS18 binding protein 1 [Source:HGNC Symbol;Acc:HGNC:20190] |
| **CDCA7** | 0.48 | 5.21 | | 6.E-07 | | 1 x 49 | | 2 q31 | | cell division cycle associated 7 [Source:HGNC Symbol;Acc:HGNC:14628] |
| **FIGNL1** | 0.48 | 5.20 | | 6.E-07 | | 2 x 50 | | 7 p12 | | fidgetin-like 1 [Source:HGNC Symbol;Acc:HGNC:13286] |
| **DDIAS** | 0.48 | 5.19 | | 6.E-07 | | 4 x 48 | | 11 q14 | | DNA damage-induced apoptosis suppressor [Source:HGNC Symbol;Acc:HGNC:26351] |
| **NUP107** | 0.48 | 5.18 | | 7.E-07 | | 2 x 48 | | 12 q15 | | nucleoporin 107kDa [Source:HGNC Symbol;Acc:HGNC:29914] |
| **TMSB15A** | 0.48 | 5.13 | | 8.E-07 | | 6 x 50 | | X q22 | | thymosin beta 15a [Source:HGNC Symbol;Acc:HGNC:30744] |
| **MTFR2** | 0.47 | 5.11 | | 9.E-07 | | 5 x 48 | | 6 q23 | | mitochondrial fission regulator 2 [Source:HGNC Symbol;Acc:HGNC:21115] |
| **NUPL1** | 0.47 | 5.11 | | 9.E-07 | | 1 x 48 | | 13 q12 | | nucleoporin like 1 [Source:HGNC Symbol;Acc:HGNC:20261] |
| **HLTF** | 0.47 | 5.11 | | 9.E-07 | | 3 x 48 | | 3 q24 | | helicase-like transcription factor [Source:HGNC Symbol;Acc:HGNC:11099] |
| **H2AFV** | 0.47 | 5.10 | | 9.E-07 | | 8 x 50 | | 7 p13 | | H2A histone family, member V [Source:HGNC Symbol;Acc:HGNC:20664] |
| **HADH** | 0.47 | 5.09 | | 1.E-06 | | 1 x 48 | | 4 q25 | | hydroxyacyl-CoA dehydrogenase [Source:HGNC Symbol;Acc:HGNC:4799] |
| **PDS5B** | 0.47 | 5.08 | | 1.E-06 | | 1 x 50 | | 13 q13 | | PDS5 cohesin associated factor B [Source:HGNC Symbol;Acc:HGNC:20418] |
| **SPDL1** | 0.47 | 5.07 | | 1.E-06 | | 6 x 49 | | 5 q35 | | spindle apparatus coiled-coil protein 1 [Source:HGNC Symbol;Acc:HGNC:26010] |
| **XRCC2** | 0.47 | 5.06 | | 1.E-06 | | 1 x 49 | | 7 q36 | | X-ray repair complementing defective repair in Chinese hamster cells 2 [Source:HGNC Symbol;Acc:HGNC:12829] |
| **NUDT1** | 0.47 | 5.05 | | 1.E-06 | | 5 x 48 | | 7 p22 | | nudix (nucleoside diphosphate linked moiety X)-type motif 1 [Source:HGNC Symbol;Acc:HGNC:8048] |
| **ARHGEF39** | 0.47 | 5.04 | | 1.E-06 | | 6 x 48 | | 9 p13 | | Rho guanine nucleotide exchange factor (GEF) 39 [Source:HGNC Symbol;Acc:HGNC:25909] |
| **ZNF143** | 0.47 | 5.03 | | 1.E-06 | | 5 x 50 | | 11 p15 | | zinc finger protein 143 [Source:HGNC Symbol;Acc:HGNC:12928] |
| **CENPO** | 0.47 | 5.02 | | 1.E-06 | | 5 x 48 | | 2 p23 | | centromere protein O [Source:HGNC Symbol;Acc:HGNC:28152] |
| **CDC25C** | 0.47 | 5.01 | | 1.E-06 | | 8 x 50 | | 5 q31 | | cell division cycle 25C [Source:HGNC Symbol;Acc:HGNC:1727] |
| **LIN9** | 0.47 | 4.99 | | 1.E-06 | | 1 x 50 | | 1 q42 | | lin-9 DREAM MuvB core complex component [Source:HGNC Symbol;Acc:HGNC:30830] |
| **HYLS1** | 0.46 | 4.98 | | 2.E-06 | | 8 x 50 | | 11 q24 | | hydrolethalus syndrome 1 [Source:HGNC Symbol;Acc:HGNC:26558] |
| **RBBP8** | 0.46 | 4.96 | | 2.E-06 | | 1 x 49 | | 18 q11 | | retinoblastoma binding protein 8 [Source:HGNC Symbol;Acc:HGNC:9891] |
| **NUDT15** | 0.46 | 4.93 | | 2.E-06 | | 3 x 49 | | 13 q14 | | nudix (nucleoside diphosphate linked moiety X)-type motif 15 [Source:HGNC Symbol;Acc:HGNC:23063] |
| **CHEK2** | 0.46 | 4.89 | | 2.E-06 | | 3 x 50 | | 22 q12 | | checkpoint kinase 2 [Source:HGNC Symbol;Acc:HGNC:16627] |
| **DHFR** | 0.45 | 4.83 | | 3.E-06 | | 1 x 50 | | 5 q14 | | dihydrofolate reductase [Source:HGNC Symbol;Acc:HGNC:2861] |
| **ANKRD32** | 0.45 | 4.83 | | 3.E-06 | | 1 x 50 | | 5 q15 | | ankyrin repeat domain 32 [Source:HGNC Symbol;Acc:HGNC:25408] |
| **ANP32B** | 0.45 | 4.83 | | 3.E-06 | | 5 x 47 | | 9 q22 | | acidic (leucine-rich) nuclear phosphoprotein 32 family, member B [Source:HGNC Symbol;Acc:HGNC:16677] |
| **NCAPD2** | 0.45 | 4.81 | | 3.E-06 | | 7 x 50 | | 12 p13 | | non-SMC condensin I complex, subunit D2 [Source:HGNC Symbol;Acc:HGNC:24305] |
| **ARL6IP6** | 0.45 | 4.79 | | 3.E-06 | | 3 x 48 | | 2 q23 | | ADP-ribosylation factor-like 6 interacting protein 6 [Source:HGNC Symbol;Acc:HGNC:24048] |
| **POC1A** | 0.45 | 4.78 | | 3.E-06 | | 4 x 48 | | 3 p21 | | POC1 centriolar protein A [Source:HGNC Symbol;Acc:HGNC:24488] |
| **NASP** | 0.45 | 4.76 | | 4.E-06 | | 1 x 48 | | 1 p34 | | nuclear autoantigenic sperm protein (histone-binding) [Source:HGNC Symbol;Acc:HGNC:7644] |
| **EXOSC8** | 0.45 | 4.74 | | 4.E-06 | | 1 x 50 | | 13 q13 | | exosome component 8 [Source:HGNC Symbol;Acc:HGNC:17035] |
| **HMGB3** | 0.45 | 4.73 | | 4.E-06 | | 7 x 50 | | X q28 | | high mobility group box 3 [Source:HGNC Symbol;Acc:HGNC:5004] |
| **RANBP1** | 0.45 | 4.72 | | 4.E-06 | | 1 x 48 | | 22 q11 | | RAN binding protein 1 [Source:HGNC Symbol;Acc:HGNC:9847] |
| **CEP250** | 0.44 | 4.71 | | 4.E-06 | | 4 x 48 | | 20 q11 | | centrosomal protein 250kDa [Source:HGNC Symbol;Acc:HGNC:1859] |
| **UBR7** | 0.44 | 4.70 | | 5.E-06 | | 1 x 47 | | NA | | ubiquitin protein ligase E3 component n-recognin 7 (putative) [Source:HGNC Symbol;Acc:HGNC:20344] |
| **HAUS1** | 0.44 | 4.70 | | 5.E-06 | | 1 x 50 | | 18 q21 | | HAUS augmin-like complex, subunit 1 [Source:HGNC Symbol;Acc:HGNC:25174] |
| **PRPS2** | 0.44 | 4.68 | | 5.E-06 | | 2 x 48 | | X p22 | | phosphoribosyl pyrophosphate synthetase 2 [Source:HGNC Symbol;Acc:HGNC:9465] |
| **DCLRE1C** | 0.44 | 4.67 | | 5.E-06 | | 3 x 49 | | 10 p13 | | DNA cross-link repair 1C [Source:HGNC Symbol;Acc:HGNC:17642] |
| **POLE** | 0.44 | 4.66 | | 5.E-06 | | 1 x 48 | | 12 q24 | | polymerase (DNA directed), epsilon, catalytic subunit [Source:HGNC Symbol;Acc:HGNC:9177] |
| **ACTL6A** | 0.44 | 4.60 | | 7.E-06 | | 4 x 50 | | 3 q26 | | actin-like 6A [Source:HGNC Symbol;Acc:HGNC:24124] |
| **C19orf48** | 0.43 | 4.55 | | 8.E-06 | | 1 x 50 | | 19 q13 | | chromosome 19 open reading frame 48 [Source:HGNC Symbol;Acc:HGNC:29667] |
| **CENPH** | 0.43 | 4.54 | | 9.E-06 | | 1 x 47 | | 5 q13 | | centromere protein H [Source:HGNC Symbol;Acc:HGNC:17268] |
| **KLF11** | 0.43 | 4.53 | | 9.E-06 | | 5 x 47 | | 2 p25 | | Kruppel-like factor 11 [Source:HGNC Symbol;Acc:HGNC:11811] |
| **FAM178A** | 0.43 | 4.53 | | 9.E-06 | | 5 x 47 | | 10 q24 | | family with sequence similarity 178, member A [Source:HGNC Symbol;Acc:HGNC:17814] |
| **MCM8** | 0.43 | 4.53 | | 9.E-06 | | 1 x 48 | | 20 p12 | | minichromosome maintenance complex component 8 [Source:HGNC Symbol;Acc:HGNC:16147] |
| **CBR3** | 0.43 | 4.52 | | 9.E-06 | | 3 x 49 | | 21 q22 | | carbonyl reductase 3 [Source:HGNC Symbol;Acc:HGNC:1549] |
| **OMD** | 0.43 | 4.51 | | 1.E-05 | | 1 x 49 | | 9 q22 | | osteomodulin [Source:HGNC Symbol;Acc:HGNC:8134] |
| **NCAPD3** | 0.43 | 4.51 | | 1.E-05 | | 4 x 48 | | 11 q25 | | non-SMC condensin II complex, subunit D3 [Source:HGNC Symbol;Acc:HGNC:28952] |
| **PCNT** | 0.43 | 4.49 | | 1.E-05 | | 4 x 48 | | 21 q22 | | pericentrin [Source:HGNC Symbol;Acc:HGNC:16068] |
| **C4orf46** | 0.43 | 4.48 | | 1.E-05 | | 1 x 48 | | 4 q32 | | chromosome 4 open reading frame 46 [Source:HGNC Symbol;Acc:HGNC:27320] |
| **HMCES** | 0.43 | 4.48 | | 1.E-05 | | 3 x 47 | | 3 q21 | | 5-hydroxymethylcytosine (hmC) binding, ES cell-specific [Source:HGNC Symbol;Acc:HGNC:24446] |
| **WDR34** | 0.43 | 4.46 | | 1.E-05 | | 5 x 50 | | 9 q34 | | WD repeat domain 34 [Source:HGNC Symbol;Acc:HGNC:28296] |
| **BRCA1** | 0.42 | 4.42 | | 1.E-05 | | 1 x 50 | | 17 q21 | | breast cancer 1, early onset [Source:HGNC Symbol;Acc:HGNC:1100] |
| **ZGRF1** | 0.42 | 4.42 | | 1.E-05 | | 1 x 50 | | 4 q25 | | zinc finger, GRF-type containing 1 [Source:HGNC Symbol;Acc:HGNC:25654] |
| **GSTCD** | 0.42 | 4.40 | | 1.E-05 | | 1 x 49 | | 4 q24 | | glutathione S-transferase, C-terminal domain containing [Source:HGNC Symbol;Acc:HGNC:25806] |
| **HAT1** | 0.42 | 4.39 | | 2.E-05 | | 1 x 50 | | 2 q31 | | histone acetyltransferase 1 [Source:HGNC Symbol;Acc:HGNC:4821] |
| **CDCA7L** | 0.42 | 4.38 | | 2.E-05 | | 1 x 50 | | 7 p15 | | cell division cycle associated 7-like [Source:HGNC Symbol;Acc:HGNC:30777] |
| **STIL** | 0.42 | 4.36 | | 2.E-05 | | 5 x 47 | | 1 p33 | | SCL/TAL1 interrupting locus [Source:HGNC Symbol;Acc:HGNC:10879] |
| **CDCA4** | 0.42 | 4.35 | | 2.E-05 | | 1 x 47 | | 14 q32 | | cell division cycle associated 4 [Source:HGNC Symbol;Acc:HGNC:14625] |
| **EZR** | 0.42 | 4.35 | | 2.E-05 | | 3 x 47 | | 6 q25 | | ezrin [Source:HGNC Symbol;Acc:HGNC:12691] |
| **NDC1** | 0.42 | 4.33 | | 2.E-05 | | 7 x 48 | | 1 p32 | | NDC1 transmembrane nucleoporin [Source:HGNC Symbol;Acc:HGNC:25525] |
| **ASRGL1** | 0.42 | 4.33 | | 2.E-05 | | 1 x 50 | | 11 q12 | | asparaginase like 1 [Source:HGNC Symbol;Acc:HGNC:16448] |
| **SLC25A40** | 0.42 | 4.33 | | 2.E-05 | | 4 x 48 | | 7 q21 | | solute carrier family 25, member 40 [Source:HGNC Symbol;Acc:HGNC:29680] |
| **DONSON** | 0.42 | 4.33 | | 2.E-05 | | 1 x 49 | | 21 q22 | | downstream neighbor of SON [Source:HGNC Symbol;Acc:HGNC:2993] |
| **CCDC18** | 0.41 | 4.32 | | 2.E-05 | | 7 x 48 | | 1 p22 | | coiled-coil domain containing 18 [Source:HGNC Symbol;Acc:HGNC:30370] |
| **RNF168** | 0.41 | 4.30 | | 2.E-05 | | 4 x 47 | | 3 q29 | | ring finger protein 168, E3 ubiquitin protein ligase [Source:HGNC Symbol;Acc:HGNC:26661] |
| **NEK2** | 0.41 | 4.29 | | 2.E-05 | | 8 x 50 | | 1 q32 | | NIMA-related kinase 2 [Source:HGNC Symbol;Acc:HGNC:7745] |
| **GINS4** | 0.41 | 4.28 | | 2.E-05 | | 5 x 47 | | 8 p11 | | GINS complex subunit 4 (Sld5 homolog) [Source:HGNC Symbol;Acc:HGNC:28226] |
| **EXOSC9** | 0.41 | 4.26 | | 3.E-05 | | 1 x 50 | | 4 q27 | | exosome component 9 [Source:HGNC Symbol;Acc:HGNC:9137] |
| **YEATS4** | 0.41 | 4.26 | | 3.E-05 | | 1 x 47 | | 12 q15 | | YEATS domain containing 4 [Source:HGNC Symbol;Acc:HGNC:24859] |
| **HSD17B11** | 0.41 | 4.25 | | 3.E-05 | | 8 x 50 | | 4 q22 | | hydroxysteroid (17-beta) dehydrogenase 11 [Source:HGNC Symbol;Acc:HGNC:22960] |
| **TMEM106C** | 0.40 | 4.20 | | 3.E-05 | | 5 x 48 | | 12 q13 | | transmembrane protein 106C [Source:HGNC Symbol;Acc:HGNC:28775] |
| **RNASEH2B** | 0.40 | 4.19 | | 3.E-05 | | 1 x 50 | | 13 q14 | | ribonuclease H2, subunit B [Source:HGNC Symbol;Acc:HGNC:25671] |
| **ZNF519** | 0.40 | 4.18 | | 3.E-05 | | 3 x 48 | | 18 p11 | | zinc finger protein 519 [Source:HGNC Symbol;Acc:HGNC:30574] |
| **POLA2** | 0.40 | 4.16 | | 4.E-05 | | 1 x 48 | | 11 q13 | | polymerase (DNA directed), alpha 2, accessory subunit [Source:HGNC Symbol;Acc:HGNC:30073] |
| **CDC7** | 0.40 | 4.16 | | 4.E-05 | | 1 x 50 | | 1 p22 | | cell division cycle 7 [Source:HGNC Symbol;Acc:HGNC:1745] |
| **RMI1** | 0.40 | 4.15 | | 4.E-05 | | 1 x 50 | | 9 q21 | | RecQ mediated genome instability 1 [Source:HGNC Symbol;Acc:HGNC:25764] |
| **NUP54** | 0.40 | 4.14 | | 4.E-05 | | 1 x 50 | | 4 q21 | | nucleoporin 54kDa [Source:HGNC Symbol;Acc:HGNC:17359] |
| **CKB** | 0.40 | 4.13 | | 4.E-05 | | 2 x 47 | | 14 q32 | | creatine kinase, brain [Source:HGNC Symbol;Acc:HGNC:1991] |
| **CCND3** | 0.40 | 4.13 | | 4.E-05 | | 1 x 47 | | 6 p21 | | cyclin D3 [Source:HGNC Symbol;Acc:HGNC:1585] |
| **MAD2L2** | 0.40 | 4.12 | | 4.E-05 | | 2 x 50 | | 1 p36 | | MAD2 mitotic arrest deficient-like 2 (yeast) [Source:HGNC Symbol;Acc:HGNC:6764] |
| **WDHD1** | 0.40 | 4.12 | | 4.E-05 | | 1 x 49 | | 14 q22 | | WD repeat and HMG-box DNA binding protein 1 [Source:HGNC Symbol;Acc:HGNC:23170] |
| **GGH** | 0.40 | 4.11 | | 4.E-05 | | 4 x 47 | | 8 q12 | | gamma-glutamyl hydrolase (conjugase, folylpolygammaglutamyl hydrolase) [Source:HGNC Symbol;Acc:HGNC:4248] |
| **EMP2** | 0.40 | 4.10 | | 4.E-05 | | 2 x 50 | | 16 p13 | | epithelial membrane protein 2 [Source:HGNC Symbol;Acc:HGNC:3334] |
| **CASP8AP2** | 0.40 | 4.10 | | 5.E-05 | | 1 x 50 | | 6 q15 | | caspase 8 associated protein 2 [Source:HGNC Symbol;Acc:HGNC:1510] |
| **TACC1** | 0.40 | 4.10 | | 5.E-05 | | 5 x 47 | | 8 p11 | | transforming, acidic coiled-coil containing protein 1 [Source:HGNC Symbol;Acc:HGNC:11522] |
| **PRIM2** | 0.40 | 4.09 | | 5.E-05 | | 5 x 48 | | 6 p11 | | primase, DNA, polypeptide 2 (58kDa) [Source:HGNC Symbol;Acc:HGNC:9370] |
| **DEPDC1B** | 0.39 | 4.04 | | 6.E-05 | | 7 x 50 | | 5 q12 | | DEP domain containing 1B [Source:HGNC Symbol;Acc:HGNC:24902] |
| **ZDHHC6** | 0.39 | 4.02 | | 6.E-05 | | 1 x 49 | | 10 q25 | | zinc finger, DHHC-type containing 6 [Source:HGNC Symbol;Acc:HGNC:19160] |
| **FRMD5** | 0.39 | 3.97 | | 7.E-05 | | 1 x 49 | | 15 q15 | | FERM domain containing 5 [Source:HGNC Symbol;Acc:HGNC:28214] |
| **PSRC1** | 0.38 | 3.94 | | 8.E-05 | | 8 x 50 | | 1 p13 | | proline/serine-rich coiled-coil 1 [Source:HGNC Symbol;Acc:HGNC:24472] |
| **ZWILCH** | 0.38 | 3.90 | | 9.E-05 | | 3 x 47 | | 15 q22 | | zwilch kinetochore protein [Source:HGNC Symbol;Acc:HGNC:25468] |
| **NCAPH2** | 0.38 | 3.87 | | 1.E-04 | | 1 x 50 | | 22 q13 | | non-SMC condensin II complex, subunit H2 [Source:HGNC Symbol;Acc:HGNC:25071] |
| **FANCL** | 0.38 | 3.87 | | 1.E-04 | | 1 x 48 | | 2 p16 | | Fanconi anemia, complementation group L [Source:HGNC Symbol;Acc:HGNC:20748] |
| **MAGOHB** | 0.37 | 3.83 | | 1.E-04 | | 1 x 47 | | 12 p13 | | mago-nashi homolog B (Drosophila) [Source:HGNC Symbol;Acc:HGNC:25504] |
| **INCENP** | 0.37 | 3.83 | | 1.E-04 | | 8 x 50 | | 11 q12 | | inner centromere protein antigens 135/155kDa [Source:HGNC Symbol;Acc:HGNC:6058] |
| **Sept10** | 0.37 | 3.81 | | 1.E-04 | | 7 x 48 | | 2 q13 | | septin 10 [Source:HGNC Symbol;Acc:HGNC:14349] |
| **PPIL3** | 0.37 | 3.80 | | 1.E-04 | | 7 x 49 | | 2 q33 | | peptidylprolyl isomerase (cyclophilin)-like 3 [Source:HGNC Symbol;Acc:HGNC:9262] |
| **KNSTRN** | 0.37 | 3.77 | | 1.E-04 | | 8 x 50 | | 15 q15 | | kinetochore-localized astrin/SPAG5 binding protein [Source:HGNC Symbol;Acc:HGNC:30767] |
| **NUP85** | 0.37 | 3.76 | | 2.E-04 | | 1 x 50 | | 17 q25 | | nucleoporin 85kDa [Source:HGNC Symbol;Acc:HGNC:8734] |
| **SAAL1** | 0.37 | 3.75 | | 2.E-04 | | 1 x 47 | | 11 p15 | | serum amyloid A-like 1 [Source:HGNC Symbol;Acc:HGNC:25158] |
| **CCDC138** | 0.36 | 3.72 | | 2.E-04 | | 7 x 48 | | 2 q13 | | coiled-coil domain containing 138 [Source:HGNC Symbol;Acc:HGNC:26531] |
| **RELT** | 0.36 | 3.71 | | 2.E-04 | | 1 x 47 | | 11 q13 | | RELT tumor necrosis factor receptor [Source:HGNC Symbol;Acc:HGNC:13764] |
| **EBP** | 0.36 | 3.71 | | 2.E-04 | | 1 x 48 | | X p11 | | emopamil binding protein (sterol isomerase) [Source:HGNC Symbol;Acc:HGNC:3133] |
| **THYN1** | 0.36 | 3.68 | | 2.E-04 | | 6 x 48 | | 11 q25 | | thymocyte nuclear protein 1 [Source:HGNC Symbol;Acc:HGNC:29560] |
| **ING3** | 0.36 | 3.67 | | 2.E-04 | | 5 x 47 | | 7 q31 | | inhibitor of growth family, member 3 [Source:HGNC Symbol;Acc:HGNC:14587] |
| **PMM1** | 0.36 | 3.67 | | 2.E-04 | | 6 x 48 | | 22 q13 | | phosphomannomutase 1 [Source:HGNC Symbol;Acc:HGNC:9114] |
| **RFWD3** | 0.36 | 3.66 | | 2.E-04 | | 1 x 49 | | 16 q23 | | ring finger and WD repeat domain 3 [Source:HGNC Symbol;Acc:HGNC:25539] |
| **FAIM** | 0.36 | 3.63 | | 2.E-04 | | 4 x 48 | | 3 q22 | | Fas apoptotic inhibitory molecule [Source:HGNC Symbol;Acc:HGNC:18703] |
| **PLSCR1** | 0.35 | 3.59 | | 3.E-04 | | 1 x 47 | | 3 q24 | | phospholipid scramblase 1 [Source:HGNC Symbol;Acc:HGNC:9092] |
| **MED14** | 0.35 | 3.55 | | 3.E-04 | | 3 x 48 | | X p11 | | mediator complex subunit 14 [Source:HGNC Symbol;Acc:HGNC:2370] |
| **HNRNPAB** | 0.35 | 3.52 | | 3.E-04 | | 1 x 48 | | 5 q35 | | heterogeneous nuclear ribonucleoprotein A/B [Source:HGNC Symbol;Acc:HGNC:5034] |
| **PLK1** | 0.35 | 3.51 | | 4.E-04 | | 8 x 50 | | 16 p12 | | polo-like kinase 1 [Source:HGNC Symbol;Acc:HGNC:9077] |
| **MPHOSPH9** | 0.35 | 3.49 | | 4.E-04 | | 7 x 49 | | 12 q24 | | M-phase phosphoprotein 9 [Source:HGNC Symbol;Acc:HGNC:7215] |
| **PHGDH** | 0.34 | 3.47 | | 4.E-04 | | 4 x 47 | | 1 p12 | | phosphoglycerate dehydrogenase [Source:HGNC Symbol;Acc:HGNC:8923] |
| **THAP7** | 0.34 | 3.45 | | 4.E-04 | | 1 x 48 | | 22 q11 | | THAP domain containing 7 [Source:HGNC Symbol;Acc:HGNC:23190] |
| **HIST1H4E** | 0.34 | 3.43 | | 5.E-04 | | 7 x 50 | | 6 p22 | | histone cluster 1, H4e [Source:HGNC Symbol;Acc:HGNC:4790] |
| **SLBP** | 0.34 | 3.43 | | 5.E-04 | | 1 x 49 | | 4 p16 | | stem-loop binding protein [Source:HGNC Symbol;Acc:HGNC:10904] |
| **AURKA** | 0.34 | 3.43 | | 5.E-04 | | 8 x 50 | | 20 q13 | | aurora kinase A [Source:HGNC Symbol;Acc:HGNC:11393] |
| **GMPS** | 0.34 | 3.42 | | 5.E-04 | | 1 x 48 | | 3 q25 | | guanine monphosphate synthase [Source:HGNC Symbol;Acc:HGNC:4378] |
| **SIN3A** | 0.34 | 3.41 | | 5.E-04 | | 1 x 49 | | 15 q24 | | SIN3 transcription regulator family member A [Source:HGNC Symbol;Acc:HGNC:19353] |
| **DDX11** | 0.34 | 3.41 | | 5.E-04 | | 1 x 47 | | 12 p11 | | DEAD/H (Asp-Glu-Ala-Asp/His) box helicase 11 [Source:HGNC Symbol;Acc:HGNC:2736] |
| **TUBGCP3** | 0.34 | 3.39 | | 0.001 | | 1 x 48 | | 13 q34 | | tubulin, gamma complex associated protein 3 [Source:HGNC Symbol;Acc:HGNC:18598] |
| **MTBP** | 0.34 | 3.37 | | 0.001 | | 4 x 47 | | 8 q24 | | MDM2 binding protein [Source:HGNC Symbol;Acc:HGNC:7417] |
| **POLA1** | 0.33 | 3.33 | | 0.001 | | 1 x 47 | | X p22 | | polymerase (DNA directed), alpha 1, catalytic subunit [Source:HGNC Symbol;Acc:HGNC:9173] |
| **RAD1** | 0.33 | 3.32 | | 0.001 | | 1 x 49 | | 5 p13 | | RAD1 checkpoint DNA exonuclease [Source:HGNC Symbol;Acc:HGNC:9806] |
| **ISOC1** | 0.33 | 3.32 | | 0.001 | | 3 x 47 | | 5 q23 | | isochorismatase domain containing 1 [Source:HGNC Symbol;Acc:HGNC:24254] |
| **TFDP1** | 0.33 | 3.31 | | 0.001 | | 1 x 47 | | 13 q34 | | transcription factor Dp-1 [Source:HGNC Symbol;Acc:HGNC:11749] |
| **GUCD1** | 0.32 | 3.24 | | 0.001 | | 1 x 47 | | 22 q11 | | guanylyl cyclase domain containing 1 [Source:HGNC Symbol;Acc:HGNC:14237] |
| **NOL12** | 0.32 | 3.24 | | 0.001 | | 1 x 47 | | 22 q13 | | nucleolar protein 12 [Source:HGNC Symbol;Acc:HGNC:28585] |
| **ZDHHC13** | 0.32 | 3.21 | | 0.001 | | 1 x 50 | | 11 p15 | | zinc finger, DHHC-type containing 13 [Source:HGNC Symbol;Acc:HGNC:18413] |
| **ZFYVE19** | 0.32 | 3.19 | | 0.001 | | 1 x 47 | | 15 q15 | | zinc finger, FYVE domain containing 19 [Source:HGNC Symbol;Acc:HGNC:20758] |
| **PCGF6** | 0.32 | 3.19 | | 0.001 | | 1 x 47 | | 10 q24 | | polycomb group ring finger 6 [Source:HGNC Symbol;Acc:HGNC:21156] |
| **TMEM237** | 0.32 | 3.16 | | 0.001 | | 4 x 48 | | 2 q33 | | transmembrane protein 237 [Source:HGNC Symbol;Acc:HGNC:14432] |
| **TTF2** | 0.32 | 3.15 | | 0.001 | | 8 x 50 | | 1 p13 | | transcription termination factor, RNA polymerase II [Source:HGNC Symbol;Acc:HGNC:12398] |
| **TRIM24** | 0.31 | 3.12 | | 0.001 | | 1 x 47 | | 7 q33 | | tripartite motif containing 24 [Source:HGNC Symbol;Acc:HGNC:11812] |
| **RANBP17** | 0.31 | 3.12 | | 0.001 | | 1 x 50 | | 5 q35 | | RAN binding protein 17 [Source:HGNC Symbol;Acc:HGNC:14428] |
| **INTS7** | 0.31 | 3.08 | | 0.001 | | 1 x 48 | | 1 q32 | | integrator complex subunit 7 [Source:HGNC Symbol;Acc:HGNC:24484] |
| **GNB4** | 0.31 | 3.08 | | 0.001 | | 1 x 48 | | 3 q26 | | guanine nucleotide binding protein (G protein), beta polypeptide 4 [Source:HGNC Symbol;Acc:HGNC:20731] |
| **LSM6** | 0.31 | 3.05 | | 0.002 | | 1 x 47 | | 4 q31 | | LSM6 homolog, U6 small nuclear RNA associated (S. cerevisiae) [Source:HGNC Symbol;Acc:HGNC:17017] |
| **TAF1A** | 0.30 | 3.04 | | 0.002 | | 1 x 48 | | 1 q41 | | TATA box binding protein (TBP)-associated factor, RNA polymerase I, A, 48kDa [Source:HGNC Symbol;Acc:HGNC:11532] |
| **FTSJ2** | 0.30 | 3.02 | | 0.002 | | 1 x 48 | | 7 p22 | | FtsJ RNA methyltransferase homolog 2 (E. coli) [Source:HGNC Symbol;Acc:HGNC:16352] |
| **EPB41L2** | 0.30 | 3.02 | | 0.002 | | 4 x 47 | | 6 q23 | | erythrocyte membrane protein band 4.1-like 2 [Source:HGNC Symbol;Acc:HGNC:3379] |
| **FN3KRP** | 0.30 | 3.01 | | 0.002 | | 1 x 48 | | 17 q25 | | fructosamine 3 kinase related protein [Source:HGNC Symbol;Acc:HGNC:25700] |
| **ARL13B** | 0.30 | 3.00 | | 0.002 | | 1 x 49 | | 3 q11 | | ADP-ribosylation factor-like 13B [Source:HGNC Symbol;Acc:HGNC:25419] |
| **NUP62** | 0.30 | 3.00 | | 0.002 | | 1 x 47 | | 19 q13 | | nucleoporin 62kDa [Source:HGNC Symbol;Acc:HGNC:8066] |
| **GAS2L3** | 0.30 | 2.97 | | 0.002 | | 8 x 50 | | 12 q23 | | growth arrest-specific 2 like 3 [Source:HGNC Symbol;Acc:HGNC:27475] |
| **PMAIP1** | 0.30 | 2.93 | | 0.002 | | 1 x 49 | | 18 q21 | | phorbol-12-myristate-13-acetate-induced protein 1 [Source:HGNC Symbol;Acc:HGNC:9108] |
| **TIMM9** | 0.29 | 2.92 | | 0.002 | | 1 x 47 | | 14 q23 | | translocase of inner mitochondrial membrane 9 homolog (yeast) [Source:HGNC Symbol;Acc:HGNC:11819] |
| **ENOSF1** | 0.29 | 2.92 | | 0.002 | | 1 x 48 | | 18 p11 | | enolase superfamily member 1 [Source:HGNC Symbol;Acc:HGNC:30365] |
| **PRMT3** | 0.29 | 2.91 | | 0.002 | | 1 x 47 | | 11 p15 | | protein arginine methyltransferase 3 [Source:HGNC Symbol;Acc:HGNC:30163] |
| **NUP205** | 0.29 | 2.84 | | 0.003 | | 1 x 47 | | 7 q33 | | nucleoporin 205kDa [Source:HGNC Symbol;Acc:HGNC:18658] |
| **CEP112** | 0.29 | 2.83 | | 0.003 | | 1 x 48 | | 17 q24 | | centrosomal protein 112kDa [Source:HGNC Symbol;Acc:HGNC:28514] |
| **TREX1** | 0.29 | 2.83 | | 0.003 | | 1 x 47 | | 3 p21 | | three prime repair exonuclease 1 [Source:HGNC Symbol;Acc:HGNC:12269] |
| **G2E3** | 0.28 | 2.79 | | 0.003 | | 8 x 50 | | 14 q12 | | G2/M-phase specific E3 ubiquitin protein ligase [Source:HGNC Symbol;Acc:HGNC:20338] |
| **CASP3** | 0.28 | 2.75 | | 0.004 | | 1 x 47 | | 4 q35 | | caspase 3, apoptosis-related cysteine peptidase [Source:HGNC Symbol;Acc:HGNC:1504] |
| **CDKN2AIP** | 0.27 | 2.66 | | 0.005 | | 1 x 47 | | 4 q35 | | CDKN2A interacting protein [Source:HGNC Symbol;Acc:HGNC:24325] |
| **PLEKHJ1** | 0.27 | 2.62 | | 0.005 | | 1 x 47 | | 19 p13 | | pleckstrin homology domain containing, family J member 1 [Source:HGNC Symbol;Acc:HGNC:18211] |
| **PRIMPOL** | 0.26 | 2.60 | | 0.005 | | 1 x 48 | | 4 q35 | | primase and polymerase (DNA-directed) [Source:HGNC Symbol;Acc:HGNC:26575] |
| **DBR1** | 0.26 | 2.60 | | 0.005 | | 1 x 48 | | 3 q22 | | debranching RNA lariats 1 [Source:HGNC Symbol;Acc:HGNC:15594] |
| **SMCO4** | 0.25 | 2.48 | | 0.008 | | 1 x 47 | | 11 q21 | | single-pass membrane protein with coiled-coil domains 4 [Source:HGNC Symbol;Acc:HGNC:24810] |
| **UBAC1** | 0.25 | 2.45 | | 0.008 | | 1 x 47 | | 9 q34 | | UBA domain containing 1 [Source:HGNC Symbol;Acc:HGNC:30221] |
| **IMPA2** | 0.24 | 2.39 | | 0.009 | | 1 x 48 | | 18 p11 | | inositol(myo)-1(or 4)-monophosphatase 2 [Source:HGNC Symbol;Acc:HGNC:6051] |
| **TIPIN** | 0.24 | 2.35 | | 0.011 | | 1 x 47 | | 15 q22 | | TIMELESS interacting protein [Source:HGNC Symbol;Acc:HGNC:30750] |
| **TEN1** | 0.24 | 2.30 | | 0.012 | | 1 x 48 | | 17 q25 | | TEN1 CST complex subunit [Source:HGNC Symbol;Acc:HGNC:37242] |
| **TRMT11** | 0.23 | 2.29 | | 0.012 | | 1 x 47 | | 6 q22 | | tRNA methyltransferase 11 homolog (S. cerevisiae) [Source:HGNC Symbol;Acc:HGNC:21080] |
| **SMCHD1** | 0.22 | 2.19 | | 0.016 | | 1 x 48 | | 18 p11 | | structural maintenance of chromosomes flexible hinge domain containing 1 [Source:HGNC Symbol;Acc:HGNC:29090] |
| **POLE2** | 0.22 | 2.18 | | 0.016 | | 1 x 47 | | 14 q21 | | polymerase (DNA directed), epsilon 2, accessory subunit [Source:HGNC Symbol;Acc:HGNC:9178] |
| **CEP97** | 0.22 | 2.14 | | 0.017 | | 2 x 47 | | 3 q12 | | centrosomal protein 97kDa [Source:HGNC Symbol;Acc:HGNC:26244] |
| **PHF5A** | 0.22 | 2.14 | | 0.017 | | 1 x 47 | | 22 q13 | | PHD finger protein 5A [Source:HGNC Symbol;Acc:HGNC:18000] |
| **CTPS1** | 0.22 | 2.10 | | 0.019 | | 1 x 47 | | 1 p34 | | CTP synthase 1 [Source:HGNC Symbol;Acc:HGNC:2519] |
| **C1GALT1** | 0.21 | 2.01 | | 0.024 | | 1 x 48 | | 7 p22 | | core 1 synthase, glycoprotein-N-acetylgalactosamine 3-beta-galactosyltransferase 1 [Source:HGNC Symbol;Acc:HGNC:24337] |
| **GSE1** | 0.20 | 1.91 | | 0.030 | | 1 x 47 | | 16 q24 | | Gse1 coiled-coil protein [Source:HGNC Symbol;Acc:HGNC:28979] |

| **Genes of spot B** | | | | | | |
| --- | --- | --- | --- | --- | --- | --- |
| **Symbol** | **Correlation ^1^** | **->t.score** | **->p.value** | **Metagene ^2^** | **Chromosome** | **Description** |
| **CDK2** | 0.76 | 10.94 | 0 | 1 x 43 | 12 q13 | cyclin-dependent kinase 2 [Source:HGNC Symbol;Acc:HGNC:1771] |
| **ARHGAP8** | 0.75 | 10.90 | 0 | 1 x 43 | 22 q13 | Rho GTPase activating protein 8 [Source:HGNC Symbol;Acc:HGNC:677] |
| **MLANA** | 0.75 | 10.75 | 0 | 1 x 43 | 9 p24 | melan-A [Source:HGNC Symbol;Acc:HGNC:7124] |
| **TRPM1** | 0.74 | 10.33 | 0 | 1 x 42 | NA | transient receptor potential cation channel, subfamily M, member 1 [Source:HGNC Symbol;Acc:HGNC:7146] |
| **CHCHD6** | 0.73 | 10.10 | 1.E-16 | 1 x 43 | 3 q21 | coiled-coil-helix-coiled-coil-helix domain containing 6 [Source:HGNC Symbol;Acc:HGNC:28184] |
| **FAM207A** | 0.72 | 9.83 | 3.E-16 | 1 x 43 | 21 q22 | family with sequence similarity 207, member A [Source:HGNC Symbol;Acc:HGNC:15811] |
| **NMRK2** | 0.71 | 9.52 | 1.E-15 | 1 x 44 | 19 p13 | nicotinamide riboside kinase 2 [Source:HGNC Symbol;Acc:HGNC:17871] |
| **ASAH1** | 0.71 | 9.51 | 2.E-15 | 1 x 40 | 8 p22 | N-acylsphingosine amidohydrolase (acid ceramidase) 1 [Source:HGNC Symbol;Acc:HGNC:735] |
| **TYR** | 0.70 | 9.27 | 5.E-15 | 1 x 42 | 11 q14 | tyrosinase [Source:HGNC Symbol;Acc:HGNC:12442] |
| **SVIP** | 0.69 | 9.09 | 1.E-14 | 1 x 44 | 11 p14 | small VCP/p97-interacting protein [Source:HGNC Symbol;Acc:HGNC:25238] |
| **CITED1** | 0.68 | 8.75 | 6.E-14 | 1 x 41 | X q13 | Cbp/p300-interacting transactivator, with Glu/Asp-rich carboxy-terminal domain, 1 [Source:HGNC Symbol;Acc:HGNC:1986] |
| **IDH3A** | 0.67 | 8.62 | 1.E-13 | 1 x 42 | 15 q25 | isocitrate dehydrogenase 3 (NAD+) alpha [Source:HGNC Symbol;Acc:HGNC:5384] |
| **TBC1D7** | 0.67 | 8.57 | 1.E-13 | 1 x 42 | 6 p24 | TBC1 domain family, member 7 [Source:HGNC Symbol;Acc:HGNC:21066] |
| **ATP5G3** | 0.66 | 8.41 | 3.E-13 | 1 x 41 | 2 q31 | ATP synthase, H+ transporting, mitochondrial Fo complex, subunit C3 (subunit 9) [Source:HGNC Symbol;Acc:HGNC:843] |
| **TIMM13** | 0.66 | 8.37 | 4.E-13 | 3 x 41 | 19 p13 | translocase of inner mitochondrial membrane 13 homolog (yeast) [Source:HGNC Symbol;Acc:HGNC:11816] |
| **EXOC3** | 0.66 | 8.35 | 4.E-13 | 1 x 42 | 5 p15 | exocyst complex component 3 [Source:HGNC Symbol;Acc:HGNC:30378] |
| **DCTPP1** | 0.66 | 8.31 | 5.E-13 | 1 x 44 | 16 p11 | dCTP pyrophosphatase 1 [Source:HGNC Symbol;Acc:HGNC:28777] |
| **PEPD** | 0.65 | 8.19 | 8.E-13 | 2 x 42 | 19 q13 | peptidase D [Source:HGNC Symbol;Acc:HGNC:8840] |
| **SLC39A6** | 0.65 | 8.05 | 2.E-12 | 1 x 42 | 18 q12 | solute carrier family 39 (zinc transporter), member 6 [Source:HGNC Symbol;Acc:HGNC:18607] |
| **BACE2** | 0.64 | 7.86 | 4.E-12 | 1 x 42 | 21 q22 | beta-site APP-cleaving enzyme 2 [Source:HGNC Symbol;Acc:HGNC:934] |
| **ST6GALNAC1** | 0.64 | 7.83 | 5.E-12 | 1 x 44 | 17 q25 | ST6 (alpha-N-acetyl-neuraminyl-2,3-beta-galactosyl-1,3)-N-acetylgalactosaminide alpha-2,6-sialyltransferase 1 [Source:HGNC Symbol;Acc:HGNC:23614] |
| **GSTP1** | 0.63 | 7.78 | 6.E-12 | 4 x 42 | 11 q13 | glutathione S-transferase pi 1 [Source:HGNC Symbol;Acc:HGNC:4638] |
| **NME1-NME2** | 0.63 | 7.73 | 7.E-12 | 4 x 39 | 17 q21 | NME1-NME2 readthrough [Source:HGNC Symbol;Acc:HGNC:33531] |
| **MBP** | 0.63 | 7.68 | 9.E-12 | 1 x 42 | 18 q23 | myelin basic protein [Source:HGNC Symbol;Acc:HGNC:6925] |
| **SLC18B1** | 0.63 | 7.61 | 1.E-11 | 1 x 42 | 6 q23 | solute carrier family 18, subfamily B, member 1 [Source:HGNC Symbol;Acc:HGNC:21573] |
| **NARS2** | 0.62 | 7.58 | 2.E-11 | 1 x 42 | 11 q14 | asparaginyl-tRNA synthetase 2, mitochondrial (putative) [Source:HGNC Symbol;Acc:HGNC:26274] |
| **SLC29A1** | 0.62 | 7.52 | 2.E-11 | 1 x 42 | 6 p21 | solute carrier family 29 (equilibrative nucleoside transporter), member 1 [Source:HGNC Symbol;Acc:HGNC:11003] |
| **CD63** | 0.62 | 7.50 | 2.E-11 | 1 x 37 | 12 q13 | CD63 molecule [Source:HGNC Symbol;Acc:HGNC:1692] |
| **C10orf11** | 0.61 | 7.37 | 4.E-11 | 1 x 43 | 10 q22 | chromosome 10 open reading frame 11 [Source:HGNC Symbol;Acc:HGNC:23405] |
| **PLP1** | 0.61 | 7.35 | 4.E-11 | 1 x 42 | X q22 | proteolipid protein 1 [Source:HGNC Symbol;Acc:HGNC:9086] |
| **BOLA3** | 0.61 | 7.34 | 5.E-11 | 1 x 40 | 2 p13 | bolA family member 3 [Source:HGNC Symbol;Acc:HGNC:24415] |
| **PMEL** | 0.61 | 7.33 | 5.E-11 | 2 x 42 | 12 q13 | premelanosome protein [Source:HGNC Symbol;Acc:HGNC:10880] |
| **PSMG1** | 0.61 | 7.30 | 6.E-11 | 3 x 43 | 21 q22 | proteasome (prosome, macropain) assembly chaperone 1 [Source:HGNC Symbol;Acc:HGNC:3043] |
| **SLC24A5** | 0.61 | 7.23 | 8.E-11 | 1 x 43 | 15 q21 | solute carrier family 24 (sodium/potassium/calcium exchanger), member 5 [Source:HGNC Symbol;Acc:HGNC:20611] |
| **VDAC1** | 0.60 | 7.19 | 9.E-11 | 2 x 40 | 5 q31 | voltage-dependent anion channel 1 [Source:HGNC Symbol;Acc:HGNC:12669] |
| **TTC39A** | 0.60 | 7.18 | 1.E-10 | 1 x 43 | 1 p32 | tetratricopeptide repeat domain 39A [Source:HGNC Symbol;Acc:HGNC:18657] |
| **SNCA** | 0.60 | 7.09 | 1.E-10 | 1 x 40 | 4 q22 | synuclein, alpha (non A4 component of amyloid precursor) [Source:HGNC Symbol;Acc:HGNC:11138] |
| **RNF14** | 0.60 | 7.06 | 2.E-10 | 1 x 43 | 5 q31 | ring finger protein 14 [Source:HGNC Symbol;Acc:HGNC:10058] |
| **GAPDHS** | 0.59 | 7.01 | 2.E-10 | 1 x 43 | 19 q13 | glyceraldehyde-3-phosphate dehydrogenase, spermatogenic [Source:HGNC Symbol;Acc:HGNC:24864] |
| **RRAGD** | 0.59 | 7.00 | 2.E-10 | 1 x 43 | 6 q15 | Ras-related GTP binding D [Source:HGNC Symbol;Acc:HGNC:19903] |
| **SLC45A2** | 0.59 | 6.97 | 3.E-10 | 1 x 42 | 5 p13 | solute carrier family 45, member 2 [Source:HGNC Symbol;Acc:HGNC:16472] |
| **ATP1A1** | 0.59 | 6.92 | 3.E-10 | 1 x 42 | 1 p13 | ATPase, Na+/K+ transporting, alpha 1 polypeptide [Source:HGNC Symbol;Acc:HGNC:799] |
| **NT5DC3** | 0.59 | 6.89 | 4.E-10 | 1 x 41 | 12 q23 | 5'-nucleotidase domain containing 3 [Source:HGNC Symbol;Acc:HGNC:30826] |
| **NCCRP1** | 0.59 | 6.88 | 4.E-10 | 1 x 43 | 19 q13 | non-specific cytotoxic cell receptor protein 1 homolog (zebrafish) [Source:HGNC Symbol;Acc:HGNC:33739] |
| **MPC1** | 0.59 | 6.84 | 5.E-10 | 1 x 43 | 6 q27 | mitochondrial pyruvate carrier 1 [Source:HGNC Symbol;Acc:HGNC:21606] |
| **MITF** | 0.58 | 6.83 | 5.E-10 | 1 x 41 | 3 p13 | microphthalmia-associated transcription factor [Source:HGNC Symbol;Acc:HGNC:7105] |
| **STX7** | 0.58 | 6.82 | 5.E-10 | 1 x 42 | 6 q23 | syntaxin 7 [Source:HGNC Symbol;Acc:HGNC:11442] |
| **MRPL34** | 0.58 | 6.71 | 8.E-10 | 1 x 40 | 19 p13 | mitochondrial ribosomal protein L34 [Source:HGNC Symbol;Acc:HGNC:14488] |
| **BIRC7** | 0.57 | 6.65 | 1.E-09 | 1 x 42 | 20 q13 | baculoviral IAP repeat containing 7 [Source:HGNC Symbol;Acc:HGNC:13702] |
| **GNPDA1** | 0.57 | 6.64 | 1.E-09 | 1 x 42 | 5 q31 | glucosamine-6-phosphate deaminase 1 [Source:HGNC Symbol;Acc:HGNC:4417] |
| **DERA** | 0.57 | 6.55 | 2.E-09 | 1 x 43 | 12 p12 | deoxyribose-phosphate aldolase (putative) [Source:HGNC Symbol;Acc:HGNC:24269] |
| **HIGD1A** | 0.57 | 6.54 | 2.E-09 | 1 x 37 | 3 p22 | HIG1 hypoxia inducible domain family, member 1A [Source:HGNC Symbol;Acc:HGNC:29527] |
| **DDT** | 0.56 | 6.48 | 2.E-09 | 1 x 39 | NA | D-dopachrome tautomerase [Source:HGNC Symbol;Acc:HGNC:2732] |
| **PEMT** | 0.56 | 6.47 | 2.E-09 | 2 x 42 | 17 p11 | phosphatidylethanolamine N-methyltransferase [Source:HGNC Symbol;Acc:HGNC:8830] |
| **AIFM1** | 0.56 | 6.45 | 3.E-09 | 1 x 42 | X q26 | apoptosis-inducing factor, mitochondrion-associated, 1 [Source:HGNC Symbol;Acc:HGNC:8768] |
| **CAPN3** | 0.56 | 6.43 | 3.E-09 | 1 x 43 | 15 q15 | calpain 3, (p94) [Source:HGNC Symbol;Acc:HGNC:1480] |
| **PDE4DIP** | 0.56 | 6.43 | 3.E-09 | 1 x 43 | 1 q21 | phosphodiesterase 4D interacting protein [Source:HGNC Symbol;Acc:HGNC:15580] |
| **EIF4EBP1** | 0.56 | 6.41 | 3.E-09 | 2 x 43 | 8 p11 | eukaryotic translation initiation factor 4E binding protein 1 [Source:HGNC Symbol;Acc:HGNC:3288] |
| **MDH2** | 0.56 | 6.40 | 4.E-09 | 3 x 39 | 7 q11 | malate dehydrogenase 2, NAD (mitochondrial) [Source:HGNC Symbol;Acc:HGNC:6971] |
| **MRPL12** | 0.56 | 6.34 | 4.E-09 | 1 x 40 | 17 q25 | mitochondrial ribosomal protein L12 [Source:HGNC Symbol;Acc:HGNC:10378] |
| **CTSL** | 0.55 | 6.33 | 5.E-09 | 2 x 40 | 9 q21 | cathepsin L [Source:HGNC Symbol;Acc:HGNC:2537] |
| **WSB2** | 0.55 | 6.32 | 5.E-09 | 1 x 38 | 12 q24 | WD repeat and SOCS box containing 2 [Source:HGNC Symbol;Acc:HGNC:19222] |
| **MLPH** | 0.55 | 6.31 | 5.E-09 | 1 x 43 | 2 q37 | melanophilin [Source:HGNC Symbol;Acc:HGNC:29643] |
| **TUBB4A** | 0.55 | 6.24 | 7.E-09 | 1 x 42 | 19 p13 | tubulin, beta 4A class IVa [Source:HGNC Symbol;Acc:HGNC:20774] |
| **FAM210A** | 0.55 | 6.23 | 7.E-09 | 4 x 40 | 18 p11 | family with sequence similarity 210, member A [Source:HGNC Symbol;Acc:HGNC:28346] |
| **METTL9** | 0.55 | 6.22 | 8.E-09 | 1 x 43 | 16 p12 | methyltransferase like 9 [Source:HGNC Symbol;Acc:HGNC:24586] |
| **C6orf1** | 0.54 | 6.14 | 1.E-08 | 1 x 39 | 6 p21 | chromosome 6 open reading frame 1 [Source:HGNC Symbol;Acc:HGNC:1340] |
| **IDH1** | 0.54 | 6.13 | 1.E-08 | 3 x 41 | 2 q34 | isocitrate dehydrogenase 1 (NADP+), soluble [Source:HGNC Symbol;Acc:HGNC:5382] |
| **GYPC** | 0.54 | 6.07 | 1.E-08 | 1 x 38 | 2 q14 | glycophorin C (Gerbich blood group) [Source:HGNC Symbol;Acc:HGNC:4704] |
| **MTCH2** | 0.54 | 6.06 | 2.E-08 | 2 x 42 | 11 p11 | mitochondrial carrier 2 [Source:HGNC Symbol;Acc:HGNC:17587] |
| **HIGD2A** | 0.54 | 6.02 | 2.E-08 | 1 x 40 | 5 q35 | HIG1 hypoxia inducible domain family, member 2A [Source:HGNC Symbol;Acc:HGNC:28311] |
| **PSMB10** | 0.53 | 5.96 | 2.E-08 | 1 x 42 | 16 q22 | proteasome (prosome, macropain) subunit, beta type, 10 [Source:HGNC Symbol;Acc:HGNC:9538] |
| **SUCLG1** | 0.53 | 5.86 | 4.E-08 | 1 x 42 | 2 p11 | succinate-CoA ligase, alpha subunit [Source:HGNC Symbol;Acc:HGNC:11449] |
| **TPCN2** | 0.53 | 5.86 | 4.E-08 | 1 x 41 | 11 q13 | two pore segment channel 2 [Source:HGNC Symbol;Acc:HGNC:20820] |
| **FAHD1** | 0.52 | 5.85 | 4.E-08 | 1 x 40 | 16 p13 | fumarylacetoacetate hydrolase domain containing 1 [Source:HGNC Symbol;Acc:HGNC:14169] |
| **CTSC** | 0.52 | 5.83 | 4.E-08 | 1 x 42 | 11 q14 | cathepsin C [Source:HGNC Symbol;Acc:HGNC:2528] |
| **NDUFA9** | 0.52 | 5.80 | 5.E-08 | 4 x 42 | 12 p13 | NADH dehydrogenase (ubiquinone) 1 alpha subcomplex, 9, 39kDa [Source:HGNC Symbol;Acc:HGNC:7693] |
| **HSPE1** | 0.52 | 5.78 | 5.E-08 | 3 x 38 | 2 q33 | heat shock 10kDa protein 1 [Source:HGNC Symbol;Acc:HGNC:5269] |
| **M6PR** | 0.52 | 5.74 | 6.E-08 | 3 x 41 | 12 p13 | mannose-6-phosphate receptor (cation dependent) [Source:HGNC Symbol;Acc:HGNC:6752] |
| **HPGD** | 0.52 | 5.74 | 6.E-08 | 1 x 44 | 4 q34 | hydroxyprostaglandin dehydrogenase 15-(NAD) [Source:HGNC Symbol;Acc:HGNC:5154] |
| **HES6** | 0.52 | 5.73 | 7.E-08 | 1 x 41 | 2 q37 | hes family bHLH transcription factor 6 [Source:HGNC Symbol;Acc:HGNC:18254] |
| **SAMM50** | 0.51 | 5.68 | 8.E-08 | 1 x 42 | 22 q13 | SAMM50 sorting and assembly machinery component [Source:HGNC Symbol;Acc:HGNC:24276] |
| **CYCS** | 0.51 | 5.66 | 9.E-08 | 1 x 37 | 7 p15 | cytochrome c, somatic [Source:HGNC Symbol;Acc:HGNC:19986] |
| **MINOS1** | 0.51 | 5.60 | 1.E-07 | 1 x 37 | 1 p36 | mitochondrial inner membrane organizing system 1 [Source:HGNC Symbol;Acc:HGNC:32068] |
| **APEH** | 0.51 | 5.57 | 1.E-07 | 1 x 42 | 3 p21 | acylaminoacyl-peptide hydrolase [Source:HGNC Symbol;Acc:HGNC:586] |
| **ECHS1** | 0.50 | 5.54 | 2.E-07 | 3 x 38 | 10 q26 | enoyl CoA hydratase, short chain, 1, mitochondrial [Source:HGNC Symbol;Acc:HGNC:3151] |
| **SFXN4** | 0.50 | 5.52 | 2.E-07 | 1 x 41 | 10 q26 | sideroflexin 4 [Source:HGNC Symbol;Acc:HGNC:16088] |
| **LYPLA1** | 0.50 | 5.45 | 2.E-07 | 3 x 43 | 8 q11 | lysophospholipase I [Source:HGNC Symbol;Acc:HGNC:6737] |
| **EIF4A3** | 0.49 | 5.38 | 3.E-07 | 4 x 40 | 17 q25 | eukaryotic translation initiation factor 4A3 [Source:HGNC Symbol;Acc:HGNC:18683] |
| **ITPKB** | 0.49 | 5.34 | 3.E-07 | 4 x 41 | 1 q42 | inositol-trisphosphate 3-kinase B [Source:HGNC Symbol;Acc:HGNC:6179] |
| **NDUFA8** | 0.49 | 5.31 | 4.E-07 | 3 x 39 | 9 q33 | NADH dehydrogenase (ubiquinone) 1 alpha subcomplex, 8, 19kDa [Source:HGNC Symbol;Acc:HGNC:7692] |
| **HSD17B10** | 0.49 | 5.29 | 4.E-07 | 3 x 39 | X p11 | hydroxysteroid (17-beta) dehydrogenase 10 [Source:HGNC Symbol;Acc:HGNC:4800] |
| **SOX10** | 0.49 | 5.28 | 4.E-07 | 1 x 41 | 22 q13 | SRY (sex determining region Y)-box 10 [Source:HGNC Symbol;Acc:HGNC:11190] |
| **PFKM** | 0.49 | 5.26 | 5.E-07 | 1 x 41 | 12 q13 | phosphofructokinase, muscle [Source:HGNC Symbol;Acc:HGNC:8877] |
| **DAB2** | 0.48 | 5.24 | 5.E-07 | 3 x 41 | 5 p13 | Dab, mitogen-responsive phosphoprotein, homolog 2 (Drosophila) [Source:HGNC Symbol;Acc:HGNC:2662] |
| **SOAT1** | 0.48 | 5.21 | 6.E-07 | 1 x 44 | 1 q25 | sterol O-acyltransferase 1 [Source:HGNC Symbol;Acc:HGNC:11177] |
| **AHCY** | 0.48 | 5.20 | 6.E-07 | 1 x 41 | 20 q11 | adenosylhomocysteinase [Source:HGNC Symbol;Acc:HGNC:343] |
| **MICAL1** | 0.48 | 5.13 | 8.E-07 | 1 x 41 | 6 q21 | microtubule associated monooxygenase, calponin and LIM domain containing 1 [Source:HGNC Symbol;Acc:HGNC:20619] |
| **MYH10** | 0.47 | 5.10 | 9.E-07 | 1 x 44 | 17 p13 | myosin, heavy chain 10, non-muscle [Source:HGNC Symbol;Acc:HGNC:7568] |
| **SLC25A39** | 0.47 | 5.10 | 9.E-07 | 3 x 39 | 17 q21 | solute carrier family 25, member 39 [Source:HGNC Symbol;Acc:HGNC:24279] |
| **SDHB** | 0.47 | 5.10 | 9.E-07 | 3 x 39 | 1 p36 | succinate dehydrogenase complex, subunit B, iron sulfur (Ip) [Source:HGNC Symbol;Acc:HGNC:10681] |
| **GOT1** | 0.47 | 5.08 | 1.E-06 | 1 x 39 | 10 q24 | glutamic-oxaloacetic transaminase 1, soluble [Source:HGNC Symbol;Acc:HGNC:4432] |
| **USF2** | 0.47 | 5.05 | 1.E-06 | 1 x 44 | 19 q13 | upstream transcription factor 2, c-fos interacting [Source:HGNC Symbol;Acc:HGNC:12594] |
| **ZNF749** | 0.47 | 5.05 | 1.E-06 | 1 x 42 | 19 q13 | zinc finger protein 749 [Source:HGNC Symbol;Acc:HGNC:32783] |
| **AK1** | 0.47 | 5.04 | 1.E-06 | 2 x 40 | 9 q34 | adenylate kinase 1 [Source:HGNC Symbol;Acc:HGNC:361] |
| **RTFDC1** | 0.47 | 5.01 | 1.E-06 | 4 x 39 | 20 q13 | replication termination factor 2 domain containing 1 [Source:HGNC Symbol;Acc:HGNC:15890] |
| **SNRPA** | 0.47 | 5.01 | 1.E-06 | 1 x 42 | 19 q13 | small nuclear ribonucleoprotein polypeptide A [Source:HGNC Symbol;Acc:HGNC:11151] |
| **CISD1** | 0.47 | 4.99 | 1.E-06 | 3 x 42 | 10 q21 | CDGSH iron sulfur domain 1 [Source:HGNC Symbol;Acc:HGNC:30880] |
| **GSTM4** | 0.46 | 4.97 | 2.E-06 | 1 x 41 | 1 p13 | glutathione S-transferase mu 4 [Source:HGNC Symbol;Acc:HGNC:4636] |
| **QARS** | 0.46 | 4.96 | 2.E-06 | 3 x 43 | 3 p21 | glutaminyl-tRNA synthetase [Source:HGNC Symbol;Acc:HGNC:9751] |
| **HPS4** | 0.46 | 4.88 | 2.E-06 | 1 x 42 | 22 q12 | Hermansky-Pudlak syndrome 4 [Source:HGNC Symbol;Acc:HGNC:15844] |
| **GPM6B** | 0.46 | 4.88 | 2.E-06 | 3 x 42 | X p22 | glycoprotein M6B [Source:HGNC Symbol;Acc:HGNC:4461] |
| **PRADC1** | 0.46 | 4.86 | 2.E-06 | 1 x 43 | 2 p13 | protease-associated domain containing 1 [Source:HGNC Symbol;Acc:HGNC:16047] |
| **ITGB1BP1** | 0.46 | 4.85 | 3.E-06 | 1 x 44 | 2 p25 | integrin beta 1 binding protein 1 [Source:HGNC Symbol;Acc:HGNC:23927] |
| **TMEM9B** | 0.46 | 4.85 | 3.E-06 | 3 x 42 | 11 p15 | TMEM9 domain family, member B [Source:HGNC Symbol;Acc:HGNC:1168] |
| **RBPMS2** | 0.45 | 4.84 | 3.E-06 | 1 x 42 | 15 q22 | RNA binding protein with multiple splicing 2 [Source:HGNC Symbol;Acc:HGNC:19098] |
| **MPDU1** | 0.45 | 4.82 | 3.E-06 | 1 x 41 | 17 p13 | mannose-P-dolichol utilization defect 1 [Source:HGNC Symbol;Acc:HGNC:7207] |
| **SLC25A11** | 0.45 | 4.81 | 3.E-06 | 1 x 39 | 17 p13 | solute carrier family 25 (mitochondrial carrier; oxoglutarate carrier), member 11 [Source:HGNC Symbol;Acc:HGNC:10981] |
| **ZC3H13** | 0.45 | 4.81 | 3.E-06 | 3 x 42 | 13 q14 | zinc finger CCCH-type containing 13 [Source:HGNC Symbol;Acc:HGNC:20368] |
| **FAM174B** | 0.45 | 4.80 | 3.E-06 | 1 x 42 | 15 q26 | family with sequence similarity 174, member B [Source:HGNC Symbol;Acc:HGNC:34339] |
| **POLR2I** | 0.45 | 4.80 | 3.E-06 | 1 x 44 | 19 q13 | polymerase (RNA) II (DNA directed) polypeptide I, 14.5kDa [Source:HGNC Symbol;Acc:HGNC:9196] |
| **MTX2** | 0.45 | 4.71 | 4.E-06 | 1 x 42 | 2 q31 | metaxin 2 [Source:HGNC Symbol;Acc:HGNC:7506] |
| **TOMM22** | 0.44 | 4.68 | 5.E-06 | 2 x 39 | 22 q13 | translocase of outer mitochondrial membrane 22 homolog (yeast) [Source:HGNC Symbol;Acc:HGNC:18002] |
| **UBE2F** | 0.44 | 4.68 | 5.E-06 | 1 x 38 | 2 q37 | ubiquitin-conjugating enzyme E2F (putative) [Source:HGNC Symbol;Acc:HGNC:12480] |
| **LSM7** | 0.44 | 4.67 | 5.E-06 | 2 x 38 | 19 p13 | LSM7 homolog, U6 small nuclear RNA associated (S. cerevisiae) [Source:HGNC Symbol;Acc:HGNC:20470] |
| **BAD** | 0.44 | 4.67 | 5.E-06 | 1 x 43 | 11 q13 | BCL2-associated agonist of cell death [Source:HGNC Symbol;Acc:HGNC:936] |
| **MAPK10** | 0.44 | 4.65 | 6.E-06 | 1 x 41 | 4 q21 | mitogen-activated protein kinase 10 [Source:HGNC Symbol;Acc:HGNC:6872] |
| **HOXD1** | 0.44 | 4.64 | 6.E-06 | 1 x 41 | 2 q31 | homeobox D1 [Source:HGNC Symbol;Acc:HGNC:5132] |
| **ABCB5** | 0.44 | 4.60 | 7.E-06 | 1 x 43 | 7 p21 | ATP-binding cassette, sub-family B (MDR/TAP), member 5 [Source:HGNC Symbol;Acc:HGNC:46] |
| **DUSP23** | 0.44 | 4.59 | 7.E-06 | 1 x 39 | 1 q23 | dual specificity phosphatase 23 [Source:HGNC Symbol;Acc:HGNC:21480] |
| **LAP3** | 0.43 | 4.55 | 9.E-06 | 1 x 41 | 4 p15 | leucine aminopeptidase 3 [Source:HGNC Symbol;Acc:HGNC:18449] |
| **CHKA** | 0.43 | 4.50 | 1.E-05 | 1 x 42 | 11 q13 | choline kinase alpha [Source:HGNC Symbol;Acc:HGNC:1937] |
| **KIAA1191** | 0.43 | 4.48 | 1.E-05 | 1 x 39 | 5 q35 | KIAA1191 [Source:HGNC Symbol;Acc:HGNC:29209] |
| **FAM213A** | 0.43 | 4.47 | 1.E-05 | 1 x 41 | 10 q23 | family with sequence similarity 213, member A [Source:HGNC Symbol;Acc:HGNC:28651] |
| **ECSIT** | 0.43 | 4.46 | 1.E-05 | 2 x 42 | 19 p13 | ECSIT signalling integrator [Source:HGNC Symbol;Acc:HGNC:29548] |
| **DOK5** | 0.43 | 4.46 | 1.E-05 | 1 x 44 | 20 q13 | docking protein 5 [Source:HGNC Symbol;Acc:HGNC:16173] |
| **FASTKD2** | 0.42 | 4.45 | 1.E-05 | 1 x 43 | 2 q33 | FAST kinase domains 2 [Source:HGNC Symbol;Acc:HGNC:29160] |
| **MLST8** | 0.42 | 4.45 | 1.E-05 | 1 x 41 | 16 p13 | MTOR associated protein, LST8 homolog (S. cerevisiae) [Source:HGNC Symbol;Acc:HGNC:24825] |
| **EEF1E1** | 0.42 | 4.44 | 1.E-05 | 3 x 40 | 6 p24 | eukaryotic translation elongation factor 1 epsilon 1 [Source:HGNC Symbol;Acc:HGNC:3212] |
| **HADHB** | 0.42 | 4.42 | 1.E-05 | 1 x 39 | 2 p23 | hydroxyacyl-CoA dehydrogenase/3-ketoacyl-CoA thiolase/enoyl-CoA hydratase (trifunctional protein), beta subunit [Source:HGNC Symbol;Acc:HGNC:4803] |
| **UNG** | 0.42 | 4.40 | 1.E-05 | 2 x 38 | 12 q24 | uracil-DNA glycosylase [Source:HGNC Symbol;Acc:HGNC:12572] |
| **NDUFAF5** | 0.42 | 4.40 | 1.E-05 | 1 x 43 | 20 p12 | NADH dehydrogenase (ubiquinone) complex I, assembly factor 5 [Source:HGNC Symbol;Acc:HGNC:15899] |
| **GLRX3** | 0.42 | 4.37 | 2.E-05 | 4 x 42 | 10 q26 | glutaredoxin 3 [Source:HGNC Symbol;Acc:HGNC:15987] |
| **AP1S2** | 0.42 | 4.37 | 2.E-05 | 4 x 41 | X p22 | adaptor-related protein complex 1, sigma 2 subunit [Source:HGNC Symbol;Acc:HGNC:560] |
| **NDUFAF4** | 0.41 | 4.33 | 2.E-05 | 4 x 41 | 6 q16 | NADH dehydrogenase (ubiquinone) complex I, assembly factor 4 [Source:HGNC Symbol;Acc:HGNC:21034] |
| **CCNB1IP1** | 0.41 | 4.31 | 2.E-05 | 1 x 41 | 14 q11 | cyclin B1 interacting protein 1, E3 ubiquitin protein ligase [Source:HGNC Symbol;Acc:HGNC:19437] |
| **WBP11** | 0.41 | 4.30 | 2.E-05 | 1 x 37 | 12 p12 | WW domain binding protein 11 [Source:HGNC Symbol;Acc:HGNC:16461] |
| **UAP1L1** | 0.41 | 4.29 | 2.E-05 | 1 x 44 | 9 q34 | UDP-N-acetylglucosamine pyrophosphorylase 1 like 1 [Source:HGNC Symbol;Acc:HGNC:28082] |
| **LGALS3** | 0.41 | 4.29 | 2.E-05 | 1 x 43 | 14 q22 | lectin, galactoside-binding, soluble, 3 [Source:HGNC Symbol;Acc:HGNC:6563] |
| **RGS20** | 0.41 | 4.24 | 3.E-05 | 1 x 41 | 8 q11 | regulator of G-protein signaling 20 [Source:HGNC Symbol;Acc:HGNC:14600] |
| **HADHA** | 0.41 | 4.24 | 3.E-05 | 1 x 38 | 2 p23 | hydroxyacyl-CoA dehydrogenase/3-ketoacyl-CoA thiolase/enoyl-CoA hydratase (trifunctional protein), alpha subunit [Source:HGNC Symbol;Acc:HGNC:4801] |
| **KIF17** | 0.41 | 4.22 | 3.E-05 | 1 x 44 | 1 p36 | kinesin family member 17 [Source:HGNC Symbol;Acc:HGNC:19167] |
| **UBL3** | 0.41 | 4.21 | 3.E-05 | 1 x 40 | 13 q12 | ubiquitin-like 3 [Source:HGNC Symbol;Acc:HGNC:12504] |
| **FAM167B** | 0.41 | 4.21 | 3.E-05 | 1 x 44 | 1 p35 | family with sequence similarity 167, member B [Source:HGNC Symbol;Acc:HGNC:28133] |
| **STRADB** | 0.41 | 4.21 | 3.E-05 | 1 x 43 | 2 q33 | STE20-related kinase adaptor beta [Source:HGNC Symbol;Acc:HGNC:13205] |
| **SDHA** | 0.40 | 4.19 | 3.E-05 | 1 x 38 | 5 p15 | succinate dehydrogenase complex, subunit A, flavoprotein (Fp) [Source:HGNC Symbol;Acc:HGNC:10680] |
| **TXN2** | 0.40 | 4.16 | 4.E-05 | 4 x 42 | 22 q12 | thioredoxin 2 [Source:HGNC Symbol;Acc:HGNC:17772] |
| **AP3S1** | 0.40 | 4.13 | 4.E-05 | 1 x 39 | 5 q22 | adaptor-related protein complex 3, sigma 1 subunit [Source:HGNC Symbol;Acc:HGNC:2013] |
| **IFRD2** | 0.40 | 4.13 | 4.E-05 | 1 x 44 | 3 p21 | interferon-related developmental regulator 2 [Source:HGNC Symbol;Acc:HGNC:5457] |
| **PTDSS1** | 0.40 | 4.11 | 4.E-05 | 1 x 39 | 8 q22 | phosphatidylserine synthase 1 [Source:HGNC Symbol;Acc:HGNC:9587] |
| **AZIN1** | 0.40 | 4.10 | 5.E-05 | 4 x 41 | 8 q22 | antizyme inhibitor 1 [Source:HGNC Symbol;Acc:HGNC:16432] |
| **CNDP2** | 0.40 | 4.08 | 5.E-05 | 3 x 40 | 18 q22 | CNDP dipeptidase 2 (metallopeptidase M20 family) [Source:HGNC Symbol;Acc:HGNC:24437] |
| **TRAPPC2L** | 0.40 | 4.08 | 5.E-05 | 3 x 41 | 16 q24 | trafficking protein particle complex 2-like [Source:HGNC Symbol;Acc:HGNC:30887] |
| **C19orf12** | 0.40 | 4.08 | 5.E-05 | 1 x 39 | 19 q12 | chromosome 19 open reading frame 12 [Source:HGNC Symbol;Acc:HGNC:25443] |
| **GLOD4** | 0.39 | 4.07 | 5.E-05 | 3 x 43 | 17 p13 | glyoxalase domain containing 4 [Source:HGNC Symbol;Acc:HGNC:14111] |
| **NANS** | 0.39 | 4.06 | 5.E-05 | 1 x 40 | 9 q22 | N-acetylneuraminic acid synthase [Source:HGNC Symbol;Acc:HGNC:19237] |
| **SNX8** | 0.39 | 4.06 | 5.E-05 | 1 x 42 | 7 p22 | sorting nexin 8 [Source:HGNC Symbol;Acc:HGNC:14972] |
| **CAPG** | 0.39 | 4.05 | 5.E-05 | 1 x 43 | 2 p11 | capping protein (actin filament), gelsolin-like [Source:HGNC Symbol;Acc:HGNC:1474] |
| **CNOT7** | 0.39 | 4.05 | 5.E-05 | 4 x 42 | 8 p22 | CCR4-NOT transcription complex, subunit 7 [Source:HGNC Symbol;Acc:HGNC:14101] |
| **MAZ** | 0.39 | 4.04 | 6.E-05 | 4 x 40 | 16 p11 | MYC-associated zinc finger protein (purine-binding transcription factor) [Source:HGNC Symbol;Acc:HGNC:6914] |
| **CLN6** | 0.39 | 4.02 | 6.E-05 | 3 x 38 | 15 q23 | ceroid-lipofuscinosis, neuronal 6, late infantile, variant [Source:HGNC Symbol;Acc:HGNC:2077] |
| **NAMPT** | 0.39 | 4.01 | 6.E-05 | 4 x 42 | 7 q22 | nicotinamide phosphoribosyltransferase [Source:HGNC Symbol;Acc:HGNC:30092] |
| **OAT** | 0.39 | 4.00 | 6.E-05 | 1 x 37 | 10 q26 | ornithine aminotransferase [Source:HGNC Symbol;Acc:HGNC:8091] |
| **FDFT1** | 0.39 | 4.00 | 7.E-05 | 1 x 38 | 8 p23 | farnesyl-diphosphate farnesyltransferase 1 [Source:HGNC Symbol;Acc:HGNC:3629] |
| **PIR** | 0.39 | 3.98 | 7.E-05 | 1 x 42 | X p22 | pirin (iron-binding nuclear protein) [Source:HGNC Symbol;Acc:HGNC:30048] |
| **GABRB3** | 0.39 | 3.98 | 7.E-05 | 1 x 41 | 15 q12 | gamma-aminobutyric acid (GABA) A receptor, beta 3 [Source:HGNC Symbol;Acc:HGNC:4083] |
| **RGS10** | 0.39 | 3.96 | 7.E-05 | 1 x 40 | 10 q26 | regulator of G-protein signaling 10 [Source:HGNC Symbol;Acc:HGNC:9992] |
| **CLPP** | 0.38 | 3.95 | 8.E-05 | 1 x 38 | 19 p13 | caseinolytic mitochondrial matrix peptidase proteolytic subunit [Source:HGNC Symbol;Acc:HGNC:2084] |
| **ABRACL** | 0.38 | 3.95 | 8.E-05 | 3 x 41 | 6 q24 | ABRA C-terminal like [Source:HGNC Symbol;Acc:HGNC:21230] |
| **PDCD5** | 0.38 | 3.92 | 9.E-05 | 1 x 37 | 19 q13 | programmed cell death 5 [Source:HGNC Symbol;Acc:HGNC:8764] |
| **HAGHL** | 0.38 | 3.92 | 9.E-05 | 4 x 40 | 16 p13 | hydroxyacylglutathione hydrolase-like [Source:HGNC Symbol;Acc:HGNC:14177] |
| **ATP6V1C1** | 0.38 | 3.91 | 9.E-05 | 4 x 40 | 8 q22 | ATPase, H+ transporting, lysosomal 42kDa, V1 subunit C1 [Source:HGNC Symbol;Acc:HGNC:856] |
| **RRP1** | 0.38 | 3.91 | 9.E-05 | 1 x 40 | 21 q22 | ribosomal RNA processing 1 [Source:HGNC Symbol;Acc:HGNC:18785] |
| **FRG1** | 0.38 | 3.90 | 9.E-05 | 3 x 39 | NA | FSHD region gene 1 [Source:HGNC Symbol;Acc:HGNC:3954] |
| **NR4A3** | 0.38 | 3.90 | 9.E-05 | 2 x 40 | 9 q31 | nuclear receptor subfamily 4, group A, member 3 [Source:HGNC Symbol;Acc:HGNC:7982] |
| **NTHL1** | 0.38 | 3.89 | 1.E-04 | 3 x 43 | 16 p13 | nth endonuclease III-like 1 (E. coli) [Source:HGNC Symbol;Acc:HGNC:8028] |
| **ATP6V1F** | 0.38 | 3.89 | 1.E-04 | 2 x 38 | 7 q32 | ATPase, H+ transporting, lysosomal 14kDa, V1 subunit F [Source:HGNC Symbol;Acc:HGNC:16832] |
| **RAB29** | 0.38 | 3.88 | 1.E-04 | 1 x 37 | 1 q32 | RAB29, member RAS oncogene family [Source:HGNC Symbol;Acc:HGNC:9789] |
| **CTSD** | 0.38 | 3.85 | 1.E-04 | 1 x 37 | 11 p15 | cathepsin D [Source:HGNC Symbol;Acc:HGNC:2529] |
| **PMPCA** | 0.37 | 3.83 | 1.E-04 | 1 x 41 | 9 q34 | peptidase (mitochondrial processing) alpha [Source:HGNC Symbol;Acc:HGNC:18667] |
| **EIF5A2** | 0.37 | 3.82 | 1.E-04 | 3 x 41 | 3 q26 | eukaryotic translation initiation factor 5A2 [Source:HGNC Symbol;Acc:HGNC:3301] |
| **GOT2** | 0.37 | 3.81 | 1.E-04 | 1 x 40 | 16 q21 | glutamic-oxaloacetic transaminase 2, mitochondrial [Source:HGNC Symbol;Acc:HGNC:4433] |
| **PDE6D** | 0.37 | 3.80 | 1.E-04 | 1 x 44 | 2 q37 | phosphodiesterase 6D, cGMP-specific, rod, delta [Source:HGNC Symbol;Acc:HGNC:8788] |
| **BCKDK** | 0.37 | 3.80 | 1.E-04 | 1 x 43 | 16 p11 | branched chain ketoacid dehydrogenase kinase [Source:HGNC Symbol;Acc:HGNC:16902] |
| **ARMC6** | 0.37 | 3.78 | 1.E-04 | 4 x 40 | 19 p13 | armadillo repeat containing 6 [Source:HGNC Symbol;Acc:HGNC:25049] |
| **SIRT2** | 0.37 | 3.78 | 1.E-04 | 1 x 38 | 19 q13 | sirtuin 2 [Source:HGNC Symbol;Acc:HGNC:10886] |
| **STXBP1** | 0.37 | 3.78 | 1.E-04 | 2 x 43 | 9 q34 | syntaxin binding protein 1 [Source:HGNC Symbol;Acc:HGNC:11444] |
| **ZFYVE16** | 0.37 | 3.77 | 1.E-04 | 1 x 40 | 5 q14 | zinc finger, FYVE domain containing 16 [Source:HGNC Symbol;Acc:HGNC:20756] |
| **ACOT7** | 0.37 | 3.75 | 2.E-04 | 1 x 38 | 1 p36 | acyl-CoA thioesterase 7 [Source:HGNC Symbol;Acc:HGNC:24157] |
| **VPS41** | 0.37 | 3.75 | 2.E-04 | 4 x 42 | 7 p14 | vacuolar protein sorting 41 homolog (S. cerevisiae) [Source:HGNC Symbol;Acc:HGNC:12713] |
| **NDUFAF3** | 0.37 | 3.74 | 2.E-04 | 3 x 38 | 3 p21 | NADH dehydrogenase (ubiquinone) complex I, assembly factor 3 [Source:HGNC Symbol;Acc:HGNC:29918] |
| **PTGFRN** | 0.37 | 3.73 | 2.E-04 | 1 x 42 | 1 p13 | prostaglandin F2 receptor inhibitor [Source:HGNC Symbol;Acc:HGNC:9601] |
| **TMC6** | 0.37 | 3.73 | 2.E-04 | 1 x 42 | 17 q25 | transmembrane channel-like 6 [Source:HGNC Symbol;Acc:HGNC:18021] |
| **EXOSC2** | 0.36 | 3.70 | 2.E-04 | 2 x 38 | 9 q34 | exosome component 2 [Source:HGNC Symbol;Acc:HGNC:17097] |
| **OLA1** | 0.36 | 3.70 | 2.E-04 | 1 x 38 | 2 q31 | Obg-like ATPase 1 [Source:HGNC Symbol;Acc:HGNC:28833] |
| **FAM49B** | 0.36 | 3.68 | 2.E-04 | 1 x 42 | 8 q24 | family with sequence similarity 49, member B [Source:HGNC Symbol;Acc:HGNC:25216] |
| **TRMT1** | 0.36 | 3.67 | 2.E-04 | 1 x 40 | 19 p13 | tRNA methyltransferase 1 homolog (S. cerevisiae) [Source:HGNC Symbol;Acc:HGNC:25980] |
| **OXA1L** | 0.36 | 3.65 | 2.E-04 | 1 x 39 | 14 q11 | oxidase (cytochrome c) assembly 1-like [Source:HGNC Symbol;Acc:HGNC:8526] |
| **EIF3K** | 0.36 | 3.64 | 2.E-04 | 1 x 37 | 19 q13 | eukaryotic translation initiation factor 3, subunit K [Source:HGNC Symbol;Acc:HGNC:24656] |
| **PLGRKT** | 0.36 | 3.63 | 2.E-04 | 1 x 41 | 9 p24 | plasminogen receptor, C-terminal lysine transmembrane protein [Source:HGNC Symbol;Acc:HGNC:23633] |
| **ISCA1** | 0.36 | 3.63 | 2.E-04 | 1 x 38 | 9 q21 | iron-sulfur cluster assembly 1 [Source:HGNC Symbol;Acc:HGNC:28660] |
| **SLC19A1** | 0.36 | 3.62 | 2.E-04 | 3 x 41 | 21 q22 | solute carrier family 19 (folate transporter), member 1 [Source:HGNC Symbol;Acc:HGNC:10937] |
| **DLGAP1** | 0.35 | 3.60 | 3.E-04 | 1 x 40 | 18 p11 | discs, large (Drosophila) homolog-associated protein 1 [Source:HGNC Symbol;Acc:HGNC:2905] |
| **MTG1** | 0.35 | 3.60 | 3.E-04 | 2 x 39 | 10 q26 | mitochondrial ribosome-associated GTPase 1 [Source:HGNC Symbol;Acc:HGNC:32159] |
| **CD68** | 0.35 | 3.58 | 3.E-04 | 1 x 38 | 17 p13 | CD68 molecule [Source:HGNC Symbol;Acc:HGNC:1693] |
| **ZFYVE21** | 0.35 | 3.55 | 3.E-04 | 1 x 38 | 14 q32 | zinc finger, FYVE domain containing 21 [Source:HGNC Symbol;Acc:HGNC:20760] |
| **UTP18** | 0.35 | 3.55 | 3.E-04 | 1 x 39 | 17 q21 | UTP18 small subunit (SSU) processome component homolog (yeast) [Source:HGNC Symbol;Acc:HGNC:24274] |
| **BTK** | 0.35 | 3.54 | 3.E-04 | 3 x 43 | X q22 | Bruton agammaglobulinemia tyrosine kinase [Source:HGNC Symbol;Acc:HGNC:1133] |
| **SLC5A4** | 0.35 | 3.54 | 3.E-04 | 1 x 43 | 22 q12 | solute carrier family 5 (glucose activated ion channel), member 4 [Source:HGNC Symbol;Acc:HGNC:11039] |
| **SLC25A16** | 0.35 | 3.53 | 3.E-04 | 1 x 39 | 10 q21 | solute carrier family 25 (mitochondrial carrier), member 16 [Source:HGNC Symbol;Acc:HGNC:10986] |
| **CHCHD4** | 0.35 | 3.52 | 3.E-04 | 1 x 38 | 3 p25 | coiled-coil-helix-coiled-coil-helix domain containing 4 [Source:HGNC Symbol;Acc:HGNC:26467] |
| **PRKAA1** | 0.35 | 3.52 | 3.E-04 | 1 x 39 | 5 p13 | protein kinase, AMP-activated, alpha 1 catalytic subunit [Source:HGNC Symbol;Acc:HGNC:9376] |
| **IMP4** | 0.35 | 3.52 | 3.E-04 | 2 x 39 | 2 q21 | IMP4, U3 small nucleolar ribonucleoprotein [Source:HGNC Symbol;Acc:HGNC:30856] |
| **SLC16A7** | 0.35 | 3.51 | 3.E-04 | 4 x 39 | 12 q14 | solute carrier family 16 (monocarboxylate transporter), member 7 [Source:HGNC Symbol;Acc:HGNC:10928] |
| **REEP6** | 0.35 | 3.51 | 4.E-04 | 1 x 43 | 19 p13 | receptor accessory protein 6 [Source:HGNC Symbol;Acc:HGNC:30078] |
| **DHCR7** | 0.35 | 3.50 | 4.E-04 | 1 x 44 | 11 q13 | 7-dehydrocholesterol reductase [Source:HGNC Symbol;Acc:HGNC:2860] |
| **MAP3K6** | 0.35 | 3.49 | 4.E-04 | 1 x 37 | 1 p36 | mitogen-activated protein kinase kinase kinase 6 [Source:HGNC Symbol;Acc:HGNC:6858] |
| **FARSA** | 0.35 | 3.49 | 4.E-04 | 3 x 41 | 19 p13 | phenylalanyl-tRNA synthetase, alpha subunit [Source:HGNC Symbol;Acc:HGNC:3592] |
| **PAFAH1B3** | 0.34 | 3.48 | 4.E-04 | 1 x 43 | 19 q13 | platelet-activating factor acetylhydrolase 1b, catalytic subunit 3 (29kDa) [Source:HGNC Symbol;Acc:HGNC:8576] |
| **ANO2** | 0.34 | 3.46 | 4.E-04 | 1 x 42 | 12 p13 | anoctamin 2, calcium activated chloride channel [Source:HGNC Symbol;Acc:HGNC:1183] |
| **RABIF** | 0.34 | 3.46 | 4.E-04 | 4 x 42 | 1 q32 | RAB interacting factor [Source:HGNC Symbol;Acc:HGNC:9797] |
| **CPSF3** | 0.34 | 3.45 | 4.E-04 | 1 x 40 | 2 p25 | cleavage and polyadenylation specific factor 3, 73kDa [Source:HGNC Symbol;Acc:HGNC:2326] |
| **ST3GAL1** | 0.34 | 3.45 | 4.E-04 | 1 x 40 | 8 q24 | ST3 beta-galactoside alpha-2,3-sialyltransferase 1 [Source:HGNC Symbol;Acc:HGNC:10862] |
| **PARL** | 0.34 | 3.45 | 4.E-04 | 1 x 39 | 3 q27 | presenilin associated, rhomboid-like [Source:HGNC Symbol;Acc:HGNC:18253] |
| **TCN2** | 0.34 | 3.44 | 4.E-04 | 1 x 44 | 22 q12 | transcobalamin II [Source:HGNC Symbol;Acc:HGNC:11653] |
| **C1orf21** | 0.34 | 3.44 | 4.E-04 | 3 x 43 | 1 q25 | chromosome 1 open reading frame 21 [Source:HGNC Symbol;Acc:HGNC:15494] |
| **CD58** | 0.34 | 3.44 | 4.E-04 | 1 x 43 | 1 p13 | CD58 molecule [Source:HGNC Symbol;Acc:HGNC:1688] |
| **NTMT1** | 0.34 | 3.43 | 5.E-04 | 4 x 40 | 9 q34 | N-terminal Xaa-Pro-Lys N-methyltransferase 1 [Source:HGNC Symbol;Acc:HGNC:23373] |
| **C8orf76** | 0.34 | 3.42 | 5.E-04 | 1 x 41 | 8 q24 | chromosome 8 open reading frame 76 [Source:HGNC Symbol;Acc:HGNC:25924] |
| **ERC1** | 0.34 | 3.42 | 5.E-04 | 1 x 38 | 12 p13 | ELKS/RAB6-interacting/CAST family member 1 [Source:HGNC Symbol;Acc:HGNC:17072] |
| **ACTR3** | 0.34 | 3.42 | 5.E-04 | 4 x 42 | 2 q14 | ARP3 actin-related protein 3 homolog (yeast) [Source:HGNC Symbol;Acc:HGNC:170] |
| **IDH3G** | 0.34 | 3.41 | 5.E-04 | 1 x 38 | X q28 | isocitrate dehydrogenase 3 (NAD+) gamma [Source:HGNC Symbol;Acc:HGNC:5386] |
| **NOP16** | 0.34 | 3.41 | 5.E-04 | 1 x 38 | 5 q35 | NOP16 nucleolar protein [Source:HGNC Symbol;Acc:HGNC:26934] |
| **GLCE** | 0.34 | 3.40 | 5.E-04 | 4 x 41 | 15 q23 | glucuronic acid epimerase [Source:HGNC Symbol;Acc:HGNC:17855] |
| **WDR91** | 0.33 | 3.36 | 6.E-04 | 1 x 44 | 7 q33 | WD repeat domain 91 [Source:HGNC Symbol;Acc:HGNC:24997] |
| **SGSH** | 0.33 | 3.36 | 6.E-04 | 1 x 44 | 17 q25 | N-sulfoglucosamine sulfohydrolase [Source:HGNC Symbol;Acc:HGNC:10818] |
| **NENF** | 0.33 | 3.35 | 6.E-04 | 1 x 42 | 1 q32 | neudesin neurotrophic factor [Source:HGNC Symbol;Acc:HGNC:30384] |
| **TMEM251** | 0.33 | 3.35 | 6.E-04 | 1 x 38 | NA | transmembrane protein 251 [Source:HGNC Symbol;Acc:HGNC:20218] |
| **DUS3L** | 0.33 | 3.34 | 6.E-04 | 1 x 40 | 19 p13 | dihydrouridine synthase 3-like (S. cerevisiae) [Source:HGNC Symbol;Acc:HGNC:26920] |
| **CHCHD5** | 0.33 | 3.33 | 6.E-04 | 4 x 41 | 2 q14 | coiled-coil-helix-coiled-coil-helix domain containing 5 [Source:HGNC Symbol;Acc:HGNC:17840] |
| **Sept9** | 0.33 | 3.32 | 6.E-04 | 4 x 41 | 17 q25 | septin 9 [Source:HGNC Symbol;Acc:HGNC:7323] |
| **TBPL1** | 0.33 | 3.31 | 7.E-04 | 4 x 41 | 6 q23 | TBP-like 1 [Source:HGNC Symbol;Acc:HGNC:11589] |
| **WIPF3** | 0.33 | 3.31 | 7.E-04 | 1 x 41 | 7 p14 | WAS/WASL interacting protein family, member 3 [Source:HGNC Symbol;Acc:HGNC:22004] |
| **COQ10A** | 0.33 | 3.31 | 7.E-04 | 1 x 41 | 12 q13 | coenzyme Q10 homolog A (S. cerevisiae) [Source:HGNC Symbol;Acc:HGNC:26515] |
| **NETO2** | 0.33 | 3.30 | 7.E-04 | 1 x 43 | 16 q12 | neuropilin (NRP) and tolloid (TLL)-like 2 [Source:HGNC Symbol;Acc:HGNC:14644] |
| **TEX264** | 0.33 | 3.29 | 7.E-04 | 3 x 41 | 3 p21 | testis expressed 264 [Source:HGNC Symbol;Acc:HGNC:30247] |
| **ELOF1** | 0.33 | 3.29 | 7.E-04 | 2 x 39 | 19 p13 | elongation factor 1 homolog (S. cerevisiae) [Source:HGNC Symbol;Acc:HGNC:28691] |
| **FLOT2** | 0.33 | 3.29 | 7.E-04 | 1 x 39 | 17 q11 | flotillin 2 [Source:HGNC Symbol;Acc:HGNC:3758] |
| **HDAC3** | 0.33 | 3.29 | 7.E-04 | 1 x 41 | 5 q31 | histone deacetylase 3 [Source:HGNC Symbol;Acc:HGNC:4854] |
| **AAMP** | 0.33 | 3.28 | 7.E-04 | 1 x 43 | 2 q35 | angio-associated, migratory cell protein [Source:HGNC Symbol;Acc:HGNC:18] |
| **SLC4A7** | 0.32 | 3.25 | 8.E-04 | 1 x 44 | 3 p24 | solute carrier family 4, sodium bicarbonate cotransporter, member 7 [Source:HGNC Symbol;Acc:HGNC:11033] |
| **TMEM141** | 0.32 | 3.25 | 8.E-04 | 1 x 44 | 9 q34 | transmembrane protein 141 [Source:HGNC Symbol;Acc:HGNC:28211] |
| **MARS2** | 0.32 | 3.25 | 8.E-04 | 1 x 39 | 2 q33 | methionyl-tRNA synthetase 2, mitochondrial [Source:HGNC Symbol;Acc:HGNC:25133] |
| **SEPSECS** | 0.32 | 3.24 | 8.E-04 | 1 x 37 | 4 p15 | Sep (O-phosphoserine) tRNA:Sec (selenocysteine) tRNA synthase [Source:HGNC Symbol;Acc:HGNC:30605] |
| **SPG21** | 0.32 | 3.23 | 9.E-04 | 1 x 39 | 15 q22 | spastic paraplegia 21 (autosomal recessive, Mast syndrome) [Source:HGNC Symbol;Acc:HGNC:20373] |
| **CCDC59** | 0.32 | 3.23 | 9.E-04 | 3 x 40 | 12 q21 | coiled-coil domain containing 59 [Source:HGNC Symbol;Acc:HGNC:25005] |
| **RTN4R** | 0.32 | 3.23 | 9.E-04 | 4 x 42 | 22 q11 | reticulon 4 receptor [Source:HGNC Symbol;Acc:HGNC:18601] |
| **IGSF8** | 0.32 | 3.21 | 9.E-04 | 1 x 41 | 1 q23 | immunoglobulin superfamily, member 8 [Source:HGNC Symbol;Acc:HGNC:17813] |
| **IFI35** | 0.32 | 3.21 | 9.E-04 | 3 x 41 | 17 q21 | interferon-induced protein 35 [Source:HGNC Symbol;Acc:HGNC:5399] |
| **ST6GALNAC3** | 0.32 | 3.20 | 9.E-04 | 1 x 44 | 1 p31 | ST6 (alpha-N-acetyl-neuraminyl-2,3-beta-galactosyl-1,3)-N-acetylgalactosaminide alpha-2,6-sialyltransferase 3 [Source:HGNC Symbol;Acc:HGNC:19343] |
| **SQLE** | 0.32 | 3.18 | 0.001 | 1 x 38 | 8 q24 | squalene epoxidase [Source:HGNC Symbol;Acc:HGNC:11279] |
| **ATP6V0E2** | 0.32 | 3.17 | 0.001 | 1 x 44 | 7 q36 | ATPase, H+ transporting V0 subunit e2 [Source:HGNC Symbol;Acc:HGNC:21723] |
| **STARD10** | 0.32 | 3.16 | 0.001 | 1 x 40 | 11 q13 | StAR-related lipid transfer (START) domain containing 10 [Source:HGNC Symbol;Acc:HGNC:10666] |
| **TAF9** | 0.32 | 3.16 | 0.001 | 1 x 38 | NA | TAF9 RNA polymerase II, TATA box binding protein (TBP)-associated factor, 32kDa [Source:HGNC Symbol;Acc:HGNC:11542] |
| **CRYL1** | 0.32 | 3.15 | 0.001 | 1 x 41 | 13 q12 | crystallin, lambda 1 [Source:HGNC Symbol;Acc:HGNC:18246] |
| **FAM104B** | 0.31 | 3.14 | 0.001 | 1 x 44 | X p11 | family with sequence similarity 104, member B [Source:HGNC Symbol;Acc:HGNC:25085] |
| **FAM222A** | 0.31 | 3.13 | 0.001 | 4 x 39 | 12 q24 | family with sequence similarity 222, member A [Source:HGNC Symbol;Acc:HGNC:25915] |
| **EPT1** | 0.31 | 3.13 | 0.001 | 1 x 39 | 2 p23 | ethanolaminephosphotransferase 1 [Source:HGNC Symbol;Acc:HGNC:29361] |
| **ULK3** | 0.31 | 3.12 | 0.001 | 3 x 41 | 15 q24 | unc-51 like kinase 3 [Source:HGNC Symbol;Acc:HGNC:19703] |
| **AIP** | 0.31 | 3.11 | 0.001 | 1 x 40 | 11 q13 | aryl hydrocarbon receptor interacting protein [Source:HGNC Symbol;Acc:HGNC:358] |
| **UCHL3** | 0.31 | 3.11 | 0.001 | 2 x 38 | 13 q22 | ubiquitin carboxyl-terminal esterase L3 (ubiquitin thiolesterase) [Source:HGNC Symbol;Acc:HGNC:12515] |
| **NR4A1** | 0.31 | 3.09 | 0.001 | 4 x 41 | 12 q13 | nuclear receptor subfamily 4, group A, member 1 [Source:HGNC Symbol;Acc:HGNC:7980] |
| **TMEM199** | 0.31 | 3.09 | 0.001 | 4 x 40 | 17 q11 | transmembrane protein 199 [Source:HGNC Symbol;Acc:HGNC:18085] |
| **CDK5RAP2** | 0.31 | 3.08 | 0.001 | 1 x 42 | 9 q33 | CDK5 regulatory subunit associated protein 2 [Source:HGNC Symbol;Acc:HGNC:18672] |
| **COQ9** | 0.31 | 3.07 | 0.001 | 1 x 38 | 16 q21 | coenzyme Q9 [Source:HGNC Symbol;Acc:HGNC:25302] |
| **HSD17B14** | 0.31 | 3.06 | 0.001 | 1 x 43 | 19 q13 | hydroxysteroid (17-beta) dehydrogenase 14 [Source:HGNC Symbol;Acc:HGNC:23238] |
| **SMIM8** | 0.31 | 3.04 | 0.002 | 1 x 39 | 6 q15 | small integral membrane protein 8 [Source:HGNC Symbol;Acc:HGNC:21401] |
| **FRMD3** | 0.30 | 3.03 | 0.002 | 1 x 41 | 9 q21 | FERM domain containing 3 [Source:HGNC Symbol;Acc:HGNC:24125] |
| **SAT2** | 0.30 | 3.03 | 0.002 | 1 x 38 | 17 p13 | spermidine/spermine N1-acetyltransferase family member 2 [Source:HGNC Symbol;Acc:HGNC:23160] |
| **OSBPL1A** | 0.30 | 2.99 | 0.002 | 1 x 44 | 18 q11 | oxysterol binding protein-like 1A [Source:HGNC Symbol;Acc:HGNC:16398] |
| **NOL8** | 0.30 | 2.98 | 0.002 | 3 x 40 | 9 q22 | nucleolar protein 8 [Source:HGNC Symbol;Acc:HGNC:23387] |
| **ZNF22** | 0.30 | 2.97 | 0.002 | 4 x 41 | 10 q11 | zinc finger protein 22 [Source:HGNC Symbol;Acc:HGNC:13012] |
| **SNAP29** | 0.30 | 2.96 | 0.002 | 1 x 40 | 22 q11 | synaptosomal-associated protein, 29kDa [Source:HGNC Symbol;Acc:HGNC:11133] |
| **NME2** | 0.30 | 2.95 | 0.002 | 1 x 40 | 17 q21 | NME/NM23 nucleoside diphosphate kinase 2 [Source:HGNC Symbol;Acc:HGNC:7850] |
| **EGR1** | 0.30 | 2.95 | 0.002 | 1 x 40 | 5 q31 | early growth response 1 [Source:HGNC Symbol;Acc:HGNC:3238] |
| **CASP7** | 0.30 | 2.93 | 0.002 | 1 x 44 | 10 q25 | caspase 7, apoptosis-related cysteine peptidase [Source:HGNC Symbol;Acc:HGNC:1508] |
| **CXXC1** | 0.30 | 2.93 | 0.002 | 3 x 39 | 18 q21 | CXXC finger protein 1 [Source:HGNC Symbol;Acc:HGNC:24343] |
| **HPS1** | 0.29 | 2.88 | 0.002 | 1 x 37 | 10 q24 | Hermansky-Pudlak syndrome 1 [Source:HGNC Symbol;Acc:HGNC:5163] |
| **DDRGK1** | 0.29 | 2.87 | 0.003 | 1 x 41 | 20 p13 | DDRGK domain containing 1 [Source:HGNC Symbol;Acc:HGNC:16110] |
| **RBM18** | 0.29 | 2.86 | 0.003 | 3 x 40 | 9 q33 | RNA binding motif protein 18 [Source:HGNC Symbol;Acc:HGNC:28413] |
| **TOM1L1** | 0.29 | 2.86 | 0.003 | 1 x 38 | 17 q22 | target of myb1 (chicken)-like 1 [Source:HGNC Symbol;Acc:HGNC:11983] |
| **WDR77** | 0.29 | 2.85 | 0.003 | 3 x 40 | 1 p13 | WD repeat domain 77 [Source:HGNC Symbol;Acc:HGNC:29652] |
| **NIFK** | 0.29 | 2.82 | 0.003 | 1 x 37 | 2 q14 | nucleolar protein interacting with the FHA domain of MKI67 [Source:HGNC Symbol;Acc:HGNC:17838] |
| **NT5DC1** | 0.28 | 2.82 | 0.003 | 1 x 44 | 6 q22 | 5'-nucleotidase domain containing 1 [Source:HGNC Symbol;Acc:HGNC:21556] |
| **MRPL40** | 0.28 | 2.81 | 0.003 | 1 x 40 | 22 q11 | mitochondrial ribosomal protein L40 [Source:HGNC Symbol;Acc:HGNC:14491] |
| **NPAS2** | 0.28 | 2.81 | 0.003 | 4 x 39 | 2 q11 | neuronal PAS domain protein 2 [Source:HGNC Symbol;Acc:HGNC:7895] |
| **SLC35A5** | 0.28 | 2.80 | 0.003 | 3 x 43 | 3 q13 | solute carrier family 35, member A5 [Source:HGNC Symbol;Acc:HGNC:20792] |
| **CEP76** | 0.28 | 2.80 | 0.003 | 1 x 43 | 18 p11 | centrosomal protein 76kDa [Source:HGNC Symbol;Acc:HGNC:25727] |
| **TCEAL1** | 0.28 | 2.80 | 0.003 | 2 x 40 | X q22 | transcription elongation factor A (SII)-like 1 [Source:HGNC Symbol;Acc:HGNC:11616] |
| **MERTK** | 0.28 | 2.79 | 0.003 | 1 x 39 | 2 q13 | MER proto-oncogene, tyrosine kinase [Source:HGNC Symbol;Acc:HGNC:7027] |
| **GYG1** | 0.28 | 2.78 | 0.003 | 3 x 39 | 3 q24 | glycogenin 1 [Source:HGNC Symbol;Acc:HGNC:4699] |
| **ITFG2** | 0.28 | 2.76 | 0.003 | 1 x 41 | 12 p13 | integrin alpha FG-GAP repeat containing 2 [Source:HGNC Symbol;Acc:HGNC:30879] |
| **THOC3** | 0.28 | 2.75 | 0.004 | 3 x 38 | 5 q35 | THO complex 3 [Source:HGNC Symbol;Acc:HGNC:19072] |
| **CLPB** | 0.28 | 2.74 | 0.004 | 1 x 39 | 11 q13 | ClpB caseinolytic peptidase B homolog (E. coli) [Source:HGNC Symbol;Acc:HGNC:30664] |
| **QDPR** | 0.28 | 2.74 | 0.004 | 1 x 41 | 4 p15 | quinoid dihydropteridine reductase [Source:HGNC Symbol;Acc:HGNC:9752] |
| **EIF2B3** | 0.27 | 2.71 | 0.004 | 1 x 39 | 1 p34 | eukaryotic translation initiation factor 2B, subunit 3 gamma, 58kDa [Source:HGNC Symbol;Acc:HGNC:3259] |
| **GCFC2** | 0.27 | 2.71 | 0.004 | 3 x 38 | 2 p12 | GC-rich sequence DNA-binding factor 2 [Source:HGNC Symbol;Acc:HGNC:1317] |
| **FASTK** | 0.27 | 2.70 | 0.004 | 1 x 39 | 7 q36 | Fas-activated serine/threonine kinase [Source:HGNC Symbol;Acc:HGNC:24676] |
| **ZNF577** | 0.27 | 2.69 | 0.004 | 2 x 39 | 19 q13 | zinc finger protein 577 [Source:HGNC Symbol;Acc:HGNC:28673] |
| **CORO1C** | 0.27 | 2.68 | 0.004 | 1 x 37 | 12 q24 | coronin, actin binding protein, 1C [Source:HGNC Symbol;Acc:HGNC:2254] |
| **HMGN5** | 0.27 | 2.67 | 0.004 | 1 x 37 | X q21 | high mobility group nucleosome binding domain 5 [Source:HGNC Symbol;Acc:HGNC:8013] |
| **MRPS11** | 0.27 | 2.67 | 0.004 | 1 x 37 | 15 q25 | mitochondrial ribosomal protein S11 [Source:HGNC Symbol;Acc:HGNC:14050] |
| **PEX11B** | 0.27 | 2.65 | 0.005 | 1 x 38 | 1 q21 | peroxisomal biogenesis factor 11 beta [Source:HGNC Symbol;Acc:HGNC:8853] |
| **CUEDC2** | 0.27 | 2.63 | 0.005 | 1 x 38 | 10 q24 | CUE domain containing 2 [Source:HGNC Symbol;Acc:HGNC:28352] |
| **BCS1L** | 0.27 | 2.62 | 0.005 | 1 x 38 | 2 q35 | BC1 (ubiquinol-cytochrome c reductase) synthesis-like [Source:HGNC Symbol;Acc:HGNC:1020] |
| **FAM193B** | 0.27 | 2.61 | 0.005 | 3 x 39 | 5 q35 | family with sequence similarity 193, member B [Source:HGNC Symbol;Acc:HGNC:25524] |
| **KCTD15** | 0.27 | 2.61 | 0.005 | 3 x 39 | 19 q13 | potassium channel tetramerization domain containing 15 [Source:HGNC Symbol;Acc:HGNC:23297] |
| **DCTN3** | 0.27 | 2.61 | 0.005 | 1 x 38 | 9 p13 | dynactin 3 (p22) [Source:HGNC Symbol;Acc:HGNC:2713] |
| **C19orf24** | 0.26 | 2.59 | 0.006 | 3 x 39 | 19 p13 | chromosome 19 open reading frame 24 [Source:HGNC Symbol;Acc:HGNC:26073] |
| **ADAT1** | 0.26 | 2.58 | 0.006 | 1 x 44 | 16 q23 | adenosine deaminase, tRNA-specific 1 [Source:HGNC Symbol;Acc:HGNC:228] |
| **TOE1** | 0.26 | 2.57 | 0.006 | 1 x 38 | 1 p34 | target of EGR1, member 1 (nuclear) [Source:HGNC Symbol;Acc:HGNC:15954] |
| **MPV17** | 0.26 | 2.53 | 0.006 | 4 x 40 | 2 p23 | MpV17 mitochondrial inner membrane protein [Source:HGNC Symbol;Acc:HGNC:7224] |
| **LINS** | 0.26 | 2.51 | 0.007 | 4 x 39 | 15 q26 | lines homolog (Drosophila) [Source:HGNC Symbol;Acc:HGNC:30922] |
| **ASUN** | 0.25 | 2.50 | 0.007 | 1 x 39 | 12 p11 | asunder spermatogenesis regulator [Source:HGNC Symbol;Acc:HGNC:20174] |
| **DDX56** | 0.25 | 2.50 | 0.007 | 1 x 38 | 7 p13 | DEAD (Asp-Glu-Ala-Asp) box helicase 56 [Source:HGNC Symbol;Acc:HGNC:18193] |
| **MAPK3** | 0.25 | 2.49 | 0.007 | 1 x 37 | 16 p11 | mitogen-activated protein kinase 3 [Source:HGNC Symbol;Acc:HGNC:6877] |
| **NUDT5** | 0.25 | 2.46 | 0.008 | 1 x 39 | 10 p14 | nudix (nucleoside diphosphate linked moiety X)-type motif 5 [Source:HGNC Symbol;Acc:HGNC:8052] |
| **SF3A1** | 0.25 | 2.43 | 0.009 | 3 x 38 | 22 q12 | splicing factor 3a, subunit 1, 120kDa [Source:HGNC Symbol;Acc:HGNC:10765] |
| **PPAP2C** | 0.25 | 2.42 | 0.009 | 1 x 40 | 19 p13 | phosphatidic acid phosphatase type 2C [Source:HGNC Symbol;Acc:HGNC:9230] |
| **IFIT3** | 0.24 | 2.40 | 0.009 | 1 x 37 | 10 q23 | interferon-induced protein with tetratricopeptide repeats 3 [Source:HGNC Symbol;Acc:HGNC:5411] |
| **PPWD1** | 0.24 | 2.40 | 0.009 | 1 x 44 | 5 q12 | peptidylprolyl isomerase domain and WD repeat containing 1 [Source:HGNC Symbol;Acc:HGNC:28954] |
| **DDX55** | 0.24 | 2.38 | 0.010 | 1 x 44 | 12 q24 | DEAD (Asp-Glu-Ala-Asp) box polypeptide 55 [Source:HGNC Symbol;Acc:HGNC:20085] |
| **RABEPK** | 0.24 | 2.37 | 0.010 | 3 x 40 | 9 q33 | Rab9 effector protein with kelch motifs [Source:HGNC Symbol;Acc:HGNC:16896] |
| **ISY1** | 0.24 | 2.36 | 0.010 | 1 x 40 | 3 q21 | ISY1 splicing factor homolog (S. cerevisiae) [Source:HGNC Symbol;Acc:HGNC:29201] |
| **MTAP** | 0.24 | 2.36 | 0.010 | 1 x 40 | 9 p21 | methylthioadenosine phosphorylase [Source:HGNC Symbol;Acc:HGNC:7413] |
| **XPNPEP1** | 0.24 | 2.35 | 0.011 | 1 x 37 | 10 q25 | X-prolyl aminopeptidase (aminopeptidase P) 1, soluble [Source:HGNC Symbol;Acc:HGNC:12822] |
| **C12orf65** | 0.23 | 2.28 | 0.012 | 1 x 37 | 12 q24 | chromosome 12 open reading frame 65 [Source:HGNC Symbol;Acc:HGNC:26784] |
| **AMD1** | 0.23 | 2.28 | 0.013 | 1 x 37 | 6 q21 | adenosylmethionine decarboxylase 1 [Source:HGNC Symbol;Acc:HGNC:457] |
| **SLC25A37** | 0.22 | 2.18 | 0.016 | 1 x 44 | 8 p21 | solute carrier family 25 (mitochondrial iron transporter), member 37 [Source:HGNC Symbol;Acc:HGNC:29786] |
| **MSRB1** | 0.22 | 2.16 | 0.017 | 2 x 38 | 16 p13 | methionine sulfoxide reductase B1 [Source:HGNC Symbol;Acc:HGNC:14133] |
| **CEP63** | 0.21 | 2.05 | 0.021 | 1 x 37 | 3 q22 | centrosomal protein 63kDa [Source:HGNC Symbol;Acc:HGNC:25815] |
| **CHMP6** | 0.20 | 1.94 | 0.028 | 1 x 39 | 17 q25 | charged multivesicular body protein 6 [Source:HGNC Symbol;Acc:HGNC:25675] |

| **Genes of spot C** | | | | | | |
| --- | --- | --- | --- | --- | --- | --- |
| **Symbol** | **Correlation ^1^** | **->t.score** | **->p.value** | **Metagene ^2^** | **Chromosome** | **Description** |
| **CCDC171** | 0.66 | 8.31 | 4.61E-13 | 1 x 11 | 9 p22 | coiled-coil domain containing 171 [Source:HGNC Symbol;Acc:HGNC:29828] |
| **GPR143** | 0.64 | 7.80 | 5.24E-12 | 1 x 11 | X p22 | G protein-coupled receptor 143 [Source:HGNC Symbol;Acc:HGNC:20145] |
| **RAB27A** | 0.63 | 7.76 | 6.31E-12 | 1 x 11 | 15 q21 | RAB27A, member RAS oncogene family [Source:HGNC Symbol;Acc:HGNC:9766] |
| **TBC1D16** | 0.63 | 7.65 | 1.08E-11 | 1 x 10 | 17 q25 | TBC1 domain family, member 16 [Source:HGNC Symbol;Acc:HGNC:28356] |
| **SOCS6** | 0.62 | 7.57 | 1.57E-11 | 1 x 11 | 18 q22 | suppressor of cytokine signaling 6 [Source:HGNC Symbol;Acc:HGNC:16833] |
| **DSTYK** | 0.61 | 7.28 | 6.01E-11 | 1 x 12 | 1 q32 | dual serine/threonine and tyrosine protein kinase [Source:HGNC Symbol;Acc:HGNC:29043] |
| **VEPH1** | 0.60 | 7.08 | 1.50E-10 | 1 x 11 | 3 q25 | ventricular zone expressed PH domain-containing 1 [Source:HGNC Symbol;Acc:HGNC:25735] |
| **ATP5B** | 0.57 | 6.65 | 1.11E-09 | 5 x 12 | 12 q13 | ATP synthase, H+ transporting, mitochondrial F1 complex, beta polypeptide [Source:HGNC Symbol;Acc:HGNC:830] |
| **CDH3** | 0.56 | 6.38 | 3.77E-09 | 1 x 11 | 16 q22 | cadherin 3, type 1, P-cadherin (placental) [Source:HGNC Symbol;Acc:HGNC:1762] |
| **SNAI2** | 0.55 | 6.27 | 6.22E-09 | 1 x 11 | 8 q11 | snail family zinc finger 2 [Source:HGNC Symbol;Acc:HGNC:11094] |
| **DAAM1** | 0.55 | 6.22 | 7.67E-09 | 1 x 12 | 14 q23 | dishevelled associated activator of morphogenesis 1 [Source:HGNC Symbol;Acc:HGNC:18142] |
| **SEMA6A** | 0.54 | 6.12 | 1.22E-08 | 1 x 11 | 5 q23 | sema domain, transmembrane domain (TM), and cytoplasmic domain, (semaphorin) 6A [Source:HGNC Symbol;Acc:HGNC:10738] |
| **GALNT3** | 0.54 | 6.08 | 1.44E-08 | 1 x 12 | 2 q24 | polypeptide N-acetylgalactosaminyltransferase 3 [Source:HGNC Symbol;Acc:HGNC:4125] |
| **BHLHE41** | 0.54 | 6.06 | 1.58E-08 | 1 x 12 | 12 p12 | basic helix-loop-helix family, member e41 [Source:HGNC Symbol;Acc:HGNC:16617] |
| **TTYH3** | 0.53 | 5.99 | 2.15E-08 | 1 x 12 | 7 p22 | tweety family member 3 [Source:HGNC Symbol;Acc:HGNC:22222] |
| **ANXA5** | 0.53 | 5.96 | 2.47E-08 | 1 x 14 | 4 q27 | annexin A5 [Source:HGNC Symbol;Acc:HGNC:543] |
| **PFKFB2** | 0.53 | 5.94 | 2.66E-08 | 2 x 13 | 1 q32 | 6-phosphofructo-2-kinase/fructose-2,6-biphosphatase 2 [Source:HGNC Symbol;Acc:HGNC:8873] |
| **OCA2** | 0.52 | 5.80 | 4.86E-08 | 1 x 12 | 15 q13 | oculocutaneous albinism II [Source:HGNC Symbol;Acc:HGNC:8101] |
| **SERINC5** | 0.52 | 5.80 | 4.87E-08 | 1 x 11 | 5 q14 | serine incorporator 5 [Source:HGNC Symbol;Acc:HGNC:18825] |
| **EDNRB** | 0.52 | 5.75 | 6.13E-08 | 1 x 11 | 13 q22 | endothelin receptor type B [Source:HGNC Symbol;Acc:HGNC:3180] |
| **STK32A** | 0.52 | 5.70 | 7.36E-08 | 1 x 10 | 5 q32 | serine/threonine kinase 32A [Source:HGNC Symbol;Acc:HGNC:28317] |
| **KCNAB2** | 0.51 | 5.69 | 7.86E-08 | 1 x 10 | 1 p36 | potassium channel, voltage gated subfamily A regulatory beta subunit 2 [Source:HGNC Symbol;Acc:HGNC:6229] |
| **SS18L1** | 0.51 | 5.68 | 8.00E-08 | 1 x 10 | 20 q13 | synovial sarcoma translocation gene on chromosome 18-like 1 [Source:HGNC Symbol;Acc:HGNC:15592] |
| **TRIM63** | 0.51 | 5.68 | 8.18E-08 | 1 x 12 | 1 p36 | tripartite motif containing 63, E3 ubiquitin protein ligase [Source:HGNC Symbol;Acc:HGNC:16007] |
| **SEMA3C** | 0.51 | 5.58 | 1.25E-07 | 1 x 10 | 7 q21 | sema domain, immunoglobulin domain (Ig), short basic domain, secreted, (semaphorin) 3C [Source:HGNC Symbol;Acc:HGNC:10725] |
| **PLXNC1** | 0.50 | 5.52 | 1.62E-07 | 1 x 11 | 12 q22 | plexin C1 [Source:HGNC Symbol;Acc:HGNC:9106] |
| **GPRC5B** | 0.50 | 5.49 | 1.84E-07 | 1 x 12 | 16 p12 | G protein-coupled receptor, class C, group 5, member B [Source:HGNC Symbol;Acc:HGNC:13308] |
| **SLC5A10** | 0.50 | 5.46 | 2.12E-07 | 2 x 10 | 17 p11 | solute carrier family 5 (sodium/sugar cotransporter), member 10 [Source:HGNC Symbol;Acc:HGNC:23155] |
| **FNIP2** | 0.50 | 5.41 | 2.53E-07 | 1 x 11 | 4 q32 | folliculin interacting protein 2 [Source:HGNC Symbol;Acc:HGNC:29280] |
| **UGCG** | 0.49 | 5.39 | 2.86E-07 | 1 x 11 | 9 q31 | UDP-glucose ceramide glucosyltransferase [Source:HGNC Symbol;Acc:HGNC:12524] |
| **MET** | 0.49 | 5.35 | 3.32E-07 | 1 x 11 | 7 q31 | MET proto-oncogene, receptor tyrosine kinase [Source:HGNC Symbol;Acc:HGNC:7029] |
| **HSPB8** | 0.49 | 5.34 | 3.41E-07 | 1 x 11 | 12 q24 | heat shock 22kDa protein 8 [Source:HGNC Symbol;Acc:HGNC:30171] |
| **ATP6V1D** | 0.49 | 5.28 | 4.39E-07 | 1 x 11 | 14 q23 | ATPase, H+ transporting, lysosomal 34kDa, V1 subunit D [Source:HGNC Symbol;Acc:HGNC:13527] |
| **SH3BP4** | 0.49 | 5.27 | 4.67E-07 | 1 x 10 | 2 q37 | SH3-domain binding protein 4 [Source:HGNC Symbol;Acc:HGNC:10826] |
| **FAM96A** | 0.49 | 5.26 | 4.75E-07 | 1 x 11 | 15 q22 | family with sequence similarity 96, member A [Source:HGNC Symbol;Acc:HGNC:26235] |
| **SLC7A5** | 0.48 | 5.24 | 5.31E-07 | 1 x 11 | 16 q24 | solute carrier family 7 (amino acid transporter light chain, L system), member 5 [Source:HGNC Symbol;Acc:HGNC:11063] |
| **TYRP1** | 0.48 | 5.14 | 8.00E-07 | 2 x 13 | 9 p23 | tyrosinase-related protein 1 [Source:HGNC Symbol;Acc:HGNC:12450] |
| **GALNTL6** | 0.48 | 5.13 | 8.35E-07 | 1 x 11 | 4 q34 | polypeptide N-acetylgalactosaminyltransferase-like 6 [Source:HGNC Symbol;Acc:HGNC:33844] |
| **ATP6V0A1** | 0.47 | 5.06 | 1.10E-06 | 1 x 10 | 17 q21 | ATPase, H+ transporting, lysosomal V0 subunit a1 [Source:HGNC Symbol;Acc:HGNC:865] |
| **ALDH1B1** | 0.47 | 5.00 | 1.38E-06 | 1 x 12 | 9 p13 | aldehyde dehydrogenase 1 family, member B1 [Source:HGNC Symbol;Acc:HGNC:407] |
| **DCT** | 0.46 | 4.93 | 1.89E-06 | 1 x 11 | 13 q32 | dopachrome tautomerase [Source:HGNC Symbol;Acc:HGNC:2709] |
| **HMGCR** | 0.46 | 4.88 | 2.31E-06 | 2 x 11 | 5 q13 | 3-hydroxy-3-methylglutaryl-CoA reductase [Source:HGNC Symbol;Acc:HGNC:5006] |
| **TNFRSF14** | 0.46 | 4.87 | 2.34E-06 | 1 x 11 | NA | tumor necrosis factor receptor superfamily, member 14 [Source:HGNC Symbol;Acc:HGNC:11912] |
| **SIRPA** | 0.45 | 4.81 | 2.96E-06 | 1 x 12 | 20 p13 | signal-regulatory protein alpha [Source:HGNC Symbol;Acc:HGNC:9662] |
| **SGCD** | 0.45 | 4.77 | 3.48E-06 | 1 x 11 | 5 q33 | sarcoglycan, delta (35kDa dystrophin-associated glycoprotein) [Source:HGNC Symbol;Acc:HGNC:10807] |
| **C6orf10** | 0.45 | 4.75 | 3.79E-06 | 4 x 12 | NA | chromosome 6 open reading frame 10 [Source:HGNC Symbol;Acc:HGNC:13922] |
| **SCD** | 0.44 | 4.66 | 5.36E-06 | 1 x 14 | 10 q24 | stearoyl-CoA desaturase (delta-9-desaturase) [Source:HGNC Symbol;Acc:HGNC:10571] |
| **SCUBE3** | 0.44 | 4.62 | 6.44E-06 | 1 x 12 | 6 p21 | signal peptide, CUB domain, EGF-like 3 [Source:HGNC Symbol;Acc:HGNC:13655] |
| **ROPN1** | 0.44 | 4.59 | 7.27E-06 | 1 x 9 | 3 q21 | rhophilin associated tail protein 1 [Source:HGNC Symbol;Acc:HGNC:17692] |
| **MCC** | 0.43 | 4.58 | 7.39E-06 | 1 x 10 | 5 q22 | mutated in colorectal cancers [Source:HGNC Symbol;Acc:HGNC:6935] |
| **MYO5A** | 0.43 | 4.58 | 7.41E-06 | 1 x 13 | 15 q21 | myosin VA (heavy chain 12, myoxin) [Source:HGNC Symbol;Acc:HGNC:7602] |
| **TMEM101** | 0.43 | 4.57 | 7.60E-06 | 1 x 11 | 17 q21 | transmembrane protein 101 [Source:HGNC Symbol;Acc:HGNC:28653] |
| **TGDS** | 0.43 | 4.55 | 8.39E-06 | 1 x 11 | 13 q32 | TDP-glucose 4,6-dehydratase [Source:HGNC Symbol;Acc:HGNC:20324] |
| **POU3F1** | 0.43 | 4.54 | 8.64E-06 | 2 x 8 | 1 p34 | POU class 3 homeobox 1 [Source:HGNC Symbol;Acc:HGNC:9214] |
| **CHMP2B** | 0.43 | 4.53 | 8.88E-06 | 3 x 10 | 3 p11 | charged multivesicular body protein 2B [Source:HGNC Symbol;Acc:HGNC:24537] |
| **ATP11A** | 0.43 | 4.53 | 8.94E-06 | 1 x 12 | 13 q34 | ATPase, class VI, type 11A [Source:HGNC Symbol;Acc:HGNC:13552] |
| **PPARGC1A** | 0.43 | 4.53 | 9.11E-06 | 1 x 10 | 4 p15 | peroxisome proliferator-activated receptor gamma, coactivator 1 alpha [Source:HGNC Symbol;Acc:HGNC:9237] |
| **SIAH1** | 0.43 | 4.52 | 9.40E-06 | 1 x 12 | 16 q12 | siah E3 ubiquitin protein ligase 1 [Source:HGNC Symbol;Acc:HGNC:10857] |
| **CFAP61** | 0.43 | 4.46 | 1.17E-05 | 1 x 12 | 20 p11 | cilia and flagella associated protein 61 [Source:HGNC Symbol;Acc:HGNC:15872] |
| **SPRYD4** | 0.43 | 4.46 | 1.19E-05 | 5 x 10 | 12 q13 | SPRY domain containing 4 [Source:HGNC Symbol;Acc:HGNC:27468] |
| **POPDC2** | 0.42 | 4.37 | 1.67E-05 | 1 x 9 | 3 q13 | popeye domain containing 2 [Source:HGNC Symbol;Acc:HGNC:17648] |
| **ZZZ3** | 0.42 | 4.34 | 1.86E-05 | 1 x 11 | 1 p31 | zinc finger, ZZ-type containing 3 [Source:HGNC Symbol;Acc:HGNC:24523] |
| **FMN1** | 0.42 | 4.34 | 1.88E-05 | 2 x 10 | 15 q13 | formin 1 [Source:HGNC Symbol;Acc:HGNC:3768] |
| **PHACTR1** | 0.42 | 4.34 | 1.88E-05 | 4 x 14 | 6 p24 | phosphatase and actin regulator 1 [Source:HGNC Symbol;Acc:HGNC:20990] |
| **LRCH1** | 0.41 | 4.30 | 2.16E-05 | 3 x 11 | 13 q14 | leucine-rich repeats and calponin homology (CH) domain containing 1 [Source:HGNC Symbol;Acc:HGNC:20309] |
| **NBL1** | 0.41 | 4.27 | 2.39E-05 | 2 x 14 | 1 p36 | neuroblastoma 1, DAN family BMP antagonist [Source:HGNC Symbol;Acc:HGNC:7650] |
| **FAM69A** | 0.41 | 4.25 | 2.56E-05 | 1 x 12 | 1 p22 | family with sequence similarity 69, member A [Source:HGNC Symbol;Acc:HGNC:32213] |
| **TRIB1** | 0.41 | 4.23 | 2.82E-05 | 1 x 11 | 8 q24 | tribbles pseudokinase 1 [Source:HGNC Symbol;Acc:HGNC:16891] |
| **PDE3A** | 0.41 | 4.22 | 2.95E-05 | 2 x 12 | 12 p12 | phosphodiesterase 3A, cGMP-inhibited [Source:HGNC Symbol;Acc:HGNC:8778] |
| **TSPAN10** | 0.41 | 4.21 | 2.98E-05 | 4 x 11 | 17 q25 | tetraspanin 10 [Source:HGNC Symbol;Acc:HGNC:29942] |
| **SOS1** | 0.41 | 4.21 | 3.04E-05 | 4 x 12 | 2 p22 | son of sevenless homolog 1 (Drosophila) [Source:HGNC Symbol;Acc:HGNC:11187] |
| **SNX27** | 0.40 | 4.17 | 3.45E-05 | 1 x 12 | 1 q21 | sorting nexin family member 27 [Source:HGNC Symbol;Acc:HGNC:20073] |
| **KLHL24** | 0.40 | 4.16 | 3.60E-05 | 1 x 11 | 3 q27 | kelch-like family member 24 [Source:HGNC Symbol;Acc:HGNC:25947] |
| **TIMM17A** | 0.40 | 4.13 | 3.99E-05 | 5 x 9 | 1 q32 | translocase of inner mitochondrial membrane 17 homolog A (yeast) [Source:HGNC Symbol;Acc:HGNC:17315] |
| **IGSF11** | 0.40 | 4.10 | 4.59E-05 | 1 x 11 | 3 q13 | immunoglobulin superfamily, member 11 [Source:HGNC Symbol;Acc:HGNC:16669] |
| **FYCO1** | 0.40 | 4.09 | 4.67E-05 | 4 x 13 | 3 p21 | FYVE and coiled-coil domain containing 1 [Source:HGNC Symbol;Acc:HGNC:14673] |
| **ZNF330** | 0.39 | 4.08 | 4.95E-05 | 2 x 13 | 4 q31 | zinc finger protein 330 [Source:HGNC Symbol;Acc:HGNC:15462] |
| **DDA1** | 0.39 | 4.07 | 4.96E-05 | 3 x 10 | 19 p13 | DET1 and DDB1 associated 1 [Source:HGNC Symbol;Acc:HGNC:28360] |
| **RAP2B** | 0.39 | 4.04 | 5.62E-05 | 5 x 14 | 3 q25 | RAP2B, member of RAS oncogene family [Source:HGNC Symbol;Acc:HGNC:9862] |
| **SDCBP** | 0.39 | 4.04 | 5.64E-05 | 3 x 11 | 8 q12 | syndecan binding protein (syntenin) [Source:HGNC Symbol;Acc:HGNC:10662] |
| **MOB3B** | 0.39 | 4.02 | 6.14E-05 | 1 x 12 | 9 p21 | MOB kinase activator 3B [Source:HGNC Symbol;Acc:HGNC:23825] |
| **NHEJ1** | 0.39 | 4.00 | 6.56E-05 | 1 x 12 | 2 q35 | nonhomologous end-joining factor 1 [Source:HGNC Symbol;Acc:HGNC:25737] |
| **PTTG1IP** | 0.39 | 3.99 | 6.68E-05 | 1 x 16 | 21 q22 | pituitary tumor-transforming 1 interacting protein [Source:HGNC Symbol;Acc:HGNC:13524] |
| **FAM69B** | 0.39 | 3.98 | 6.93E-05 | 1 x 12 | 9 q34 | family with sequence similarity 69, member B [Source:HGNC Symbol;Acc:HGNC:28290] |
| **CHST11** | 0.39 | 3.98 | 7.09E-05 | 4 x 10 | 12 q23 | carbohydrate (chondroitin 4) sulfotransferase 11 [Source:HGNC Symbol;Acc:HGNC:17422] |
| **ESRP1** | 0.39 | 3.96 | 7.41E-05 | 1 x 12 | 8 q22 | epithelial splicing regulatory protein 1 [Source:HGNC Symbol;Acc:HGNC:25966] |
| **ADSS** | 0.39 | 3.96 | 7.51E-05 | 1 x 9 | 1 q44 | adenylosuccinate synthase [Source:HGNC Symbol;Acc:HGNC:292] |
| **C10orf90** | 0.39 | 3.96 | 7.52E-05 | 1 x 11 | 10 q26 | chromosome 10 open reading frame 90 [Source:HGNC Symbol;Acc:HGNC:26563] |
| **EPG5** | 0.38 | 3.95 | 7.77E-05 | 1 x 10 | 18 q21 | ectopic P-granules autophagy protein 5 homolog (C. elegans) [Source:HGNC Symbol;Acc:HGNC:29331] |
| **ACO2** | 0.38 | 3.94 | 7.96E-05 | 2 x 10 | 22 q13 | aconitase 2, mitochondrial [Source:HGNC Symbol;Acc:HGNC:118] |
| **ADRBK2** | 0.38 | 3.92 | 8.65E-05 | 1 x 10 | 22 q12 | adrenergic, beta, receptor kinase 2 [Source:HGNC Symbol;Acc:HGNC:290] |
| **BIN1** | 0.38 | 3.92 | 8.68E-05 | 1 x 10 | 2 q14 | bridging integrator 1 [Source:HGNC Symbol;Acc:HGNC:1052] |
| **TRAK2** | 0.38 | 3.91 | 8.95E-05 | 1 x 12 | 2 q33 | trafficking protein, kinesin binding 2 [Source:HGNC Symbol;Acc:HGNC:13206] |
| **SGK1** | 0.38 | 3.87 | 1.E-04 | 1 x 13 | 6 q23 | serum/glucocorticoid regulated kinase 1 [Source:HGNC Symbol;Acc:HGNC:10810] |
| **C1orf54** | 0.38 | 3.86 | 1.E-04 | 1 x 11 | 1 q21 | chromosome 1 open reading frame 54 [Source:HGNC Symbol;Acc:HGNC:26258] |
| **MREG** | 0.38 | 3.85 | 1.E-04 | 3 x 11 | 2 q35 | melanoregulin [Source:HGNC Symbol;Acc:HGNC:25478] |
| **XG** | 0.37 | 3.83 | 1.E-04 | 2 x 9 | X p22 | Xg blood group [Source:HGNC Symbol;Acc:HGNC:12806] |
| **ABR** | 0.37 | 3.83 | 1.E-04 | 1 x 13 | NA | active BCR-related [Source:HGNC Symbol;Acc:HGNC:81] |
| **CABLES1** | 0.37 | 3.83 | 1.E-04 | 1 x 13 | 18 q11 | Cdk5 and Abl enzyme substrate 1 [Source:HGNC Symbol;Acc:HGNC:25097] |
| **DNAJC3** | 0.37 | 3.82 | 1.E-04 | 1 x 14 | 13 q32 | DnaJ (Hsp40) homolog, subfamily C, member 3 [Source:HGNC Symbol;Acc:HGNC:9439] |
| **ROPN1B** | 0.37 | 3.79 | 1.E-04 | 1 x 10 | 3 q21 | rhophilin associated tail protein 1B [Source:HGNC Symbol;Acc:HGNC:31927] |
| **SORBS1** | 0.37 | 3.77 | 1.E-04 | 1 x 12 | 10 q24 | sorbin and SH3 domain containing 1 [Source:HGNC Symbol;Acc:HGNC:14565] |
| **AGPAT3** | 0.37 | 3.76 | 2.E-04 | 1 x 13 | 21 q22 | 1-acylglycerol-3-phosphate O-acyltransferase 3 [Source:HGNC Symbol;Acc:HGNC:326] |
| **RASGRP3** | 0.37 | 3.75 | 2.E-04 | 1 x 12 | 2 p22 | RAS guanyl releasing protein 3 (calcium and DAG-regulated) [Source:HGNC Symbol;Acc:HGNC:14545] |
| **PCSK2** | 0.37 | 3.74 | 2.E-04 | 1 x 10 | 20 p12 | proprotein convertase subtilisin/kexin type 2 [Source:HGNC Symbol;Acc:HGNC:8744] |
| **TULP4** | 0.37 | 3.73 | 2.E-04 | 1 x 11 | 6 q25 | tubby like protein 4 [Source:HGNC Symbol;Acc:HGNC:15530] |
| **HAS2** | 0.36 | 3.71 | 2.E-04 | 1 x 10 | 8 q24 | hyaluronan synthase 2 [Source:HGNC Symbol;Acc:HGNC:4819] |
| **NAV2** | 0.36 | 3.70 | 2.E-04 | 1 x 12 | 11 p15 | neuron navigator 2 [Source:HGNC Symbol;Acc:HGNC:15997] |
| **LETM1** | 0.36 | 3.69 | 2.E-04 | 6 x 14 | 4 p16 | leucine zipper-EF-hand containing transmembrane protein 1 [Source:HGNC Symbol;Acc:HGNC:6556] |
| **EVC** | 0.36 | 3.68 | 2.E-04 | 5 x 9 | 4 p16 | Ellis van Creveld syndrome [Source:HGNC Symbol;Acc:HGNC:3497] |
| **IGSF3** | 0.36 | 3.65 | 2.E-04 | 1 x 13 | 1 p13 | immunoglobulin superfamily, member 3 [Source:HGNC Symbol;Acc:HGNC:5950] |
| **NRP2** | 0.36 | 3.64 | 2.E-04 | 1 x 10 | 2 q33 | neuropilin 2 [Source:HGNC Symbol;Acc:HGNC:8005] |
| **SEMA6D** | 0.36 | 3.64 | 2.E-04 | 1 x 11 | 15 q21 | sema domain, transmembrane domain (TM), and cytoplasmic domain, (semaphorin) 6D [Source:HGNC Symbol;Acc:HGNC:16770] |
| **STAT3** | 0.36 | 3.63 | 2.E-04 | 1 x 8 | 17 q21 | signal transducer and activator of transcription 3 (acute-phase response factor) [Source:HGNC Symbol;Acc:HGNC:11364] |
| **C15orf61** | 0.36 | 3.62 | 2.E-04 | 3 x 16 | 15 q23 | chromosome 15 open reading frame 61 [Source:HGNC Symbol;Acc:HGNC:34453] |
| **BEST1** | 0.36 | 3.62 | 2.E-04 | 1 x 10 | 11 q12 | bestrophin 1 [Source:HGNC Symbol;Acc:HGNC:12703] |
| **TMEM177** | 0.36 | 3.61 | 3.E-04 | 3 x 10 | 2 q14 | transmembrane protein 177 [Source:HGNC Symbol;Acc:HGNC:28143] |
| **FAM161A** | 0.36 | 3.61 | 3.E-04 | 1 x 12 | 2 p15 | family with sequence similarity 161, member A [Source:HGNC Symbol;Acc:HGNC:25808] |
| **PIK3CB** | 0.35 | 3.59 | 3.E-04 | 1 x 11 | 3 q22 | phosphatidylinositol-4,5-bisphosphate 3-kinase, catalytic subunit beta [Source:HGNC Symbol;Acc:HGNC:8976] |
| **CEACAM1** | 0.35 | 3.59 | 3.E-04 | 1 x 10 | 19 q13 | carcinoembryonic antigen-related cell adhesion molecule 1 (biliary glycoprotein) [Source:HGNC Symbol;Acc:HGNC:1814] |
| **MCF2** | 0.35 | 3.58 | 3.E-04 | 5 x 10 | X q27 | MCF.2 cell line derived transforming sequence [Source:HGNC Symbol;Acc:HGNC:6940] |
| **EGLN3** | 0.35 | 3.57 | 3.E-04 | 2 x 12 | 14 q13 | egl-9 family hypoxia-inducible factor 3 [Source:HGNC Symbol;Acc:HGNC:14661] |
| **C4orf45** | 0.35 | 3.57 | 3.E-04 | 1 x 11 | 4 q32 | chromosome 4 open reading frame 45 [Source:HGNC Symbol;Acc:HGNC:26342] |
| **TDRD7** | 0.35 | 3.57 | 3.E-04 | 1 x 11 | 9 q22 | tudor domain containing 7 [Source:HGNC Symbol;Acc:HGNC:30831] |
| **DGKI** | 0.35 | 3.55 | 3.E-04 | 1 x 11 | 7 q33 | diacylglycerol kinase, iota [Source:HGNC Symbol;Acc:HGNC:2855] |
| **KRTAP19-1** | 0.35 | 3.54 | 3.E-04 | 1 x 8 | 21 q22 | keratin associated protein 19-1 [Source:HGNC Symbol;Acc:HGNC:18936] |
| **EXOC7** | 0.35 | 3.53 | 3.E-04 | 1 x 8 | 17 q25 | exocyst complex component 7 [Source:HGNC Symbol;Acc:HGNC:23214] |
| **IRF4** | 0.35 | 3.52 | 3.E-04 | 1 x 11 | 6 p25 | interferon regulatory factor 4 [Source:HGNC Symbol;Acc:HGNC:6119] |
| **STOX2** | 0.35 | 3.51 | 3.E-04 | 5 x 10 | 4 q35 | storkhead box 2 [Source:HGNC Symbol;Acc:HGNC:25450] |
| **GDPD5** | 0.35 | 3.51 | 4.E-04 | 1 x 12 | 11 q13 | glycerophosphodiester phosphodiesterase domain containing 5 [Source:HGNC Symbol;Acc:HGNC:28804] |
| **AGPAT6** | 0.35 | 3.50 | 4.E-04 | 1 x 9 | 8 p11 | 1-acylglycerol-3-phosphate O-acyltransferase 6 [Source:HGNC Symbol;Acc:HGNC:20880] |
| **GM2A** | 0.35 | 3.49 | 4.E-04 | 1 x 8 | 5 q33 | GM2 ganglioside activator [Source:HGNC Symbol;Acc:HGNC:4367] |
| **BCAT2** | 0.35 | 3.49 | 4.E-04 | 1 x 11 | 19 q13 | branched chain amino-acid transaminase 2, mitochondrial [Source:HGNC Symbol;Acc:HGNC:977] |
| **ANGPTL2** | 0.34 | 3.49 | 4.E-04 | 3 x 13 | 9 q33 | angiopoietin-like 2 [Source:HGNC Symbol;Acc:HGNC:490] |
| **FAM63B** | 0.34 | 3.48 | 4.E-04 | 1 x 11 | 15 q21 | family with sequence similarity 63, member B [Source:HGNC Symbol;Acc:HGNC:26954] |
| **RAB5B** | 0.34 | 3.46 | 4.E-04 | 4 x 10 | 12 q13 | RAB5B, member RAS oncogene family [Source:HGNC Symbol;Acc:HGNC:9784] |
| **MSI2** | 0.34 | 3.46 | 4.E-04 | 1 x 10 | 17 q22 | musashi RNA-binding protein 2 [Source:HGNC Symbol;Acc:HGNC:18585] |
| **MTURN** | 0.34 | 3.46 | 4.E-04 | 4 x 14 | 7 p14 | maturin, neural progenitor differentiation regulator homolog (Xenopus) [Source:HGNC Symbol;Acc:HGNC:25457] |
| **C6orf106** | 0.34 | 3.45 | 4.E-04 | 4 x 11 | 6 p21 | chromosome 6 open reading frame 106 [Source:HGNC Symbol;Acc:HGNC:21215] |
| **WDR43** | 0.34 | 3.44 | 4.E-04 | 4 x 11 | 2 p23 | WD repeat domain 43 [Source:HGNC Symbol;Acc:HGNC:28945] |
| **KIAA2026** | 0.34 | 3.44 | 4.E-04 | 1 x 9 | 9 p24 | KIAA2026 [Source:HGNC Symbol;Acc:HGNC:23378] |
| **KCNQ5** | 0.34 | 3.44 | 4.E-04 | 1 x 10 | 6 q13 | potassium channel, voltage gated KQT-like subfamily Q, member 5 [Source:HGNC Symbol;Acc:HGNC:6299] |
| **VPS13D** | 0.34 | 3.43 | 5.E-04 | 1 x 9 | 1 p36 | vacuolar protein sorting 13 homolog D (S. cerevisiae) [Source:HGNC Symbol;Acc:HGNC:23595] |
| **OSBPL2** | 0.34 | 3.43 | 5.E-04 | 2 x 13 | 20 q13 | oxysterol binding protein-like 2 [Source:HGNC Symbol;Acc:HGNC:15761] |
| **SLC26A4** | 0.34 | 3.42 | 5.E-04 | 2 x 15 | 7 q22 | solute carrier family 26 (anion exchanger), member 4 [Source:HGNC Symbol;Acc:HGNC:8818] |
| **SLC19A2** | 0.34 | 3.42 | 5.E-04 | 4 x 12 | 1 q24 | solute carrier family 19 (thiamine transporter), member 2 [Source:HGNC Symbol;Acc:HGNC:10938] |
| **ID2** | 0.34 | 3.40 | 5.E-04 | 1 x 13 | 2 p25 | inhibitor of DNA binding 2, dominant negative helix-loop-helix protein [Source:HGNC Symbol;Acc:HGNC:5361] |
| **GREB1** | 0.34 | 3.40 | 5.E-04 | 1 x 9 | 2 p25 | growth regulation by estrogen in breast cancer 1 [Source:HGNC Symbol;Acc:HGNC:24885] |
| **MCF2L** | 0.34 | 3.39 | 5.E-04 | 1 x 10 | 13 q34 | MCF.2 cell line derived transforming sequence-like [Source:HGNC Symbol;Acc:HGNC:14576] |
| **LARP1** | 0.33 | 3.37 | 6.E-04 | 6 x 13 | 5 q33 | La ribonucleoprotein domain family, member 1 [Source:HGNC Symbol;Acc:HGNC:29531] |
| **ENPP2** | 0.33 | 3.36 | 6.E-04 | 1 x 9 | 8 q24 | ectonucleotide pyrophosphatase/phosphodiesterase 2 [Source:HGNC Symbol;Acc:HGNC:3357] |
| **VAV3** | 0.33 | 3.34 | 6.E-04 | 1 x 14 | 1 p13 | vav 3 guanine nucleotide exchange factor [Source:HGNC Symbol;Acc:HGNC:12659] |
| **SLC9A1** | 0.33 | 3.33 | 6.E-04 | 3 x 10 | 1 p36 | solute carrier family 9, subfamily A (NHE1, cation proton antiporter 1), member 1 [Source:HGNC Symbol;Acc:HGNC:11071] |
| **MGAT5** | 0.33 | 3.32 | 6.E-04 | 1 x 9 | 2 q21 | mannosyl (alpha-1,6-)-glycoprotein beta-1,6-N-acetyl-glucosaminyltransferase [Source:HGNC Symbol;Acc:HGNC:7049] |
| **SSH1** | 0.33 | 3.31 | 7.E-04 | 6 x 10 | 12 q24 | slingshot protein phosphatase 1 [Source:HGNC Symbol;Acc:HGNC:30579] |
| **PPM1H** | 0.33 | 3.31 | 7.E-04 | 1 x 11 | 12 q14 | protein phosphatase, Mg2+/Mn2+ dependent, 1H [Source:HGNC Symbol;Acc:HGNC:18583] |
| **HMGCS1** | 0.33 | 3.31 | 7.E-04 | 1 x 15 | 5 p12 | 3-hydroxy-3-methylglutaryl-CoA synthase 1 (soluble) [Source:HGNC Symbol;Acc:HGNC:5007] |
| **KIAA1551** | 0.33 | 3.30 | 7.E-04 | 2 x 13 | 12 p11 | KIAA1551 [Source:HGNC Symbol;Acc:HGNC:25559] |
| **ZHX1-C8orf76** | 0.33 | 3.30 | 7.E-04 | 1 x 9 | 8 q24 | ZHX1-C8orf76 readthrough [Source:HGNC Symbol;Acc:HGNC:42975] |
| **LYST** | 0.33 | 3.29 | 7.E-04 | 6 x 11 | 1 q42 | lysosomal trafficking regulator [Source:HGNC Symbol;Acc:HGNC:1968] |
| **LONP2** | 0.33 | 3.29 | 7.E-04 | 3 x 7 | 16 q12 | lon peptidase 2, peroxisomal [Source:HGNC Symbol;Acc:HGNC:20598] |
| **HMG20A** | 0.33 | 3.28 | 7.E-04 | 1 x 13 | 15 q24 | high mobility group 20A [Source:HGNC Symbol;Acc:HGNC:5001] |
| **C21orf91** | 0.33 | 3.28 | 7.E-04 | 1 x 9 | 21 q21 | chromosome 21 open reading frame 91 [Source:HGNC Symbol;Acc:HGNC:16459] |
| **ARHGAP35** | 0.33 | 3.28 | 7.E-04 | 4 x 8 | 19 q13 | Rho GTPase activating protein 35 [Source:HGNC Symbol;Acc:HGNC:4591] |
| **APOL2** | 0.33 | 3.27 | 8.E-04 | 1 x 9 | 22 q12 | apolipoprotein L, 2 [Source:HGNC Symbol;Acc:HGNC:619] |
| **NCALD** | 0.33 | 3.27 | 8.E-04 | 1 x 15 | 8 q22 | neurocalcin delta [Source:HGNC Symbol;Acc:HGNC:7655] |
| **MOB1B** | 0.33 | 3.27 | 8.E-04 | 4 x 13 | 4 q13 | MOB kinase activator 1B [Source:HGNC Symbol;Acc:HGNC:29801] |
| **MTFMT** | 0.33 | 3.27 | 8.E-04 | 3 x 9 | 15 q22 | mitochondrial methionyl-tRNA formyltransferase [Source:HGNC Symbol;Acc:HGNC:29666] |
| **MYO10** | 0.33 | 3.27 | 8.E-04 | 5 x 16 | 5 p15 | myosin X [Source:HGNC Symbol;Acc:HGNC:7593] |
| **TMEM150A** | 0.32 | 3.25 | 8.E-04 | 4 x 11 | 2 p11 | transmembrane protein 150A [Source:HGNC Symbol;Acc:HGNC:24677] |
| **TAPBPL** | 0.32 | 3.24 | 8.E-04 | 3 x 13 | 12 p13 | TAP binding protein-like [Source:HGNC Symbol;Acc:HGNC:30683] |
| **NKAIN4** | 0.32 | 3.23 | 9.E-04 | 5 x 10 | 20 q13 | Na+/K+ transporting ATPase interacting 4 [Source:HGNC Symbol;Acc:HGNC:16191] |
| **C5orf22** | 0.32 | 3.21 | 9.E-04 | 2 x 12 | 5 p13 | chromosome 5 open reading frame 22 [Source:HGNC Symbol;Acc:HGNC:25639] |
| **VDAC2** | 0.32 | 3.21 | 9.E-04 | 6 x 9 | 10 q22 | voltage-dependent anion channel 2 [Source:HGNC Symbol;Acc:HGNC:12672] |
| **PHLDA1** | 0.32 | 3.21 | 9.E-04 | 3 x 14 | 12 q21 | pleckstrin homology-like domain, family A, member 1 [Source:HGNC Symbol;Acc:HGNC:8933] |
| **ATP6V1E2** | 0.32 | 3.20 | 9.E-04 | 5 x 12 | 2 p21 | ATPase, H+ transporting, lysosomal 31kDa, V1 subunit E2 [Source:HGNC Symbol;Acc:HGNC:18125] |
| **MTDH** | 0.32 | 3.20 | 1.E-03 | 4 x 15 | 8 q22 | metadherin [Source:HGNC Symbol;Acc:HGNC:29608] |
| **NFKB1** | 0.32 | 3.19 | 1.E-03 | 3 x 15 | 4 q24 | nuclear factor of kappa light polypeptide gene enhancer in B-cells 1 [Source:HGNC Symbol;Acc:HGNC:7794] |
| **CD81** | 0.32 | 3.19 | 1.E-03 | 3 x 9 | 11 p15 | CD81 molecule [Source:HGNC Symbol;Acc:HGNC:1701] |
| **PAIP1** | 0.32 | 3.19 | 1.E-03 | 1 x 9 | 5 p12 | poly(A) binding protein interacting protein 1 [Source:HGNC Symbol;Acc:HGNC:16945] |
| **WDR81** | 0.32 | 3.18 | 1.E-03 | 2 x 12 | NA | WD repeat domain 81 [Source:HGNC Symbol;Acc:HGNC:26600] |
| **COPG1** | 0.32 | 3.18 | 0.001 | 1 x 9 | 3 q21 | coatomer protein complex, subunit gamma 1 [Source:HGNC Symbol;Acc:HGNC:2236] |
| **ZNF284** | 0.32 | 3.16 | 0.001 | 2 x 10 | 19 q13 | zinc finger protein 284 [Source:HGNC Symbol;Acc:HGNC:13078] |
| **TUB** | 0.32 | 3.15 | 0.001 | 2 x 15 | 11 p15 | tubby bipartite transcription factor [Source:HGNC Symbol;Acc:HGNC:12406] |
| **LRRC8D** | 0.32 | 3.15 | 0.001 | 1 x 8 | 1 p22 | leucine rich repeat containing 8 family, member D [Source:HGNC Symbol;Acc:HGNC:16992] |
| **MRPL47** | 0.31 | 3.14 | 0.001 | 4 x 9 | 3 q26 | mitochondrial ribosomal protein L47 [Source:HGNC Symbol;Acc:HGNC:16652] |
| **TEX2** | 0.31 | 3.13 | 0.001 | 1 x 13 | 17 q23 | testis expressed 2 [Source:HGNC Symbol;Acc:HGNC:30884] |
| **FAM124A** | 0.31 | 3.12 | 0.001 | 1 x 11 | 13 q14 | family with sequence similarity 124A [Source:HGNC Symbol;Acc:HGNC:26413] |
| **SNX30** | 0.31 | 3.12 | 0.001 | 4 x 12 | 9 q32 | sorting nexin family member 30 [Source:HGNC Symbol;Acc:HGNC:23685] |
| **MIEF1** | 0.31 | 3.11 | 0.001 | 3 x 13 | 22 q13 | mitochondrial elongation factor 1 [Source:HGNC Symbol;Acc:HGNC:25979] |
| **NUDT3** | 0.31 | 3.10 | 0.001 | 4 x 16 | 6 p21 | nudix (nucleoside diphosphate linked moiety X)-type motif 3 [Source:HGNC Symbol;Acc:HGNC:8050] |
| **LPIN1** | 0.31 | 3.10 | 0.001 | 1 x 8 | 2 p25 | lipin 1 [Source:HGNC Symbol;Acc:HGNC:13345] |
| **SAT1** | 0.31 | 3.08 | 0.001 | 1 x 11 | X p22 | spermidine/spermine N1-acetyltransferase 1 [Source:HGNC Symbol;Acc:HGNC:10540] |
| **RINT1** | 0.31 | 3.08 | 0.001 | 2 x 11 | 7 q22 | RAD50 interactor 1 [Source:HGNC Symbol;Acc:HGNC:21876] |
| **BLOC1S6** | 0.31 | 3.06 | 0.001 | 5 x 13 | 15 q21 | biogenesis of lysosomal organelles complex-1, subunit 6, pallidin [Source:HGNC Symbol;Acc:HGNC:8549] |
| **EMCN** | 0.31 | 3.05 | 0.001 | 5 x 12 | 4 q24 | endomucin [Source:HGNC Symbol;Acc:HGNC:16041] |
| **PLA1A** | 0.31 | 3.05 | 0.001 | 1 x 10 | 3 q13 | phospholipase A1 member A [Source:HGNC Symbol;Acc:HGNC:17661] |
| **DMXL1** | 0.31 | 3.05 | 0.001 | 1 x 9 | 5 q23 | Dmx-like 1 [Source:HGNC Symbol;Acc:HGNC:2937] |
| **ALS2CR12** | 0.31 | 3.04 | 0.002 | 1 x 7 | 2 q33 | amyotrophic lateral sclerosis 2 (juvenile) chromosome region, candidate 12 [Source:HGNC Symbol;Acc:HGNC:14439] |
| **PLXNA4** | 0.31 | 3.04 | 0.002 | 6 x 14 | 7 q32 | plexin A4 [Source:HGNC Symbol;Acc:HGNC:9102] |
| **SETX** | 0.30 | 3.03 | 0.002 | 4 x 14 | 9 q34 | senataxin [Source:HGNC Symbol;Acc:HGNC:445] |
| **NFU1** | 0.30 | 3.03 | 0.002 | 1 x 10 | 2 p13 | NFU1 iron-sulfur cluster scaffold [Source:HGNC Symbol;Acc:HGNC:16287] |
| **CXorf23** | 0.30 | 3.03 | 0.002 | 1 x 9 | X p22 | chromosome X open reading frame 23 [Source:HGNC Symbol;Acc:HGNC:27413] |
| **NPM3** | 0.30 | 3.02 | 0.002 | 4 x 10 | 10 q24 | nucleophosmin/nucleoplasmin 3 [Source:HGNC Symbol;Acc:HGNC:7931] |
| **NCOA4** | 0.30 | 3.02 | 0.002 | 1 x 7 | 10 q11 | nuclear receptor coactivator 4 [Source:HGNC Symbol;Acc:HGNC:7671] |
| **GPR161** | 0.30 | 3.01 | 0.002 | 3 x 16 | 1 q24 | G protein-coupled receptor 161 [Source:HGNC Symbol;Acc:HGNC:23694] |
| **PLXNA1** | 0.30 | 2.99 | 0.002 | 2 x 7 | 3 q21 | plexin A1 [Source:HGNC Symbol;Acc:HGNC:9099] |
| **ADAM10** | 0.30 | 2.99 | 0.002 | 4 x 11 | 15 q21 | ADAM metallopeptidase domain 10 [Source:HGNC Symbol;Acc:HGNC:188] |
| **ADIPOR2** | 0.30 | 2.99 | 0.002 | 1 x 10 | 12 p13 | adiponectin receptor 2 [Source:HGNC Symbol;Acc:HGNC:24041] |
| **PLA2G4B** | 0.30 | 2.97 | 0.002 | 3 x 9 | 15 q15 | phospholipase A2, group IVB (cytosolic) [Source:HGNC Symbol;Acc:HGNC:9036] |
| **TRIO** | 0.30 | 2.97 | 0.002 | 3 x 11 | 5 p15 | trio Rho guanine nucleotide exchange factor [Source:HGNC Symbol;Acc:HGNC:12303] |
| **MMP8** | 0.30 | 2.97 | 0.002 | 5 x 12 | 11 q22 | matrix metallopeptidase 8 [Source:HGNC Symbol;Acc:HGNC:7175] |
| **LONRF1** | 0.30 | 2.96 | 0.002 | 1 x 12 | 8 p23 | LON peptidase N-terminal domain and ring finger 1 [Source:HGNC Symbol;Acc:HGNC:26302] |
| **MTIF3** | 0.30 | 2.95 | 0.002 | 1 x 9 | 13 q12 | mitochondrial translational initiation factor 3 [Source:HGNC Symbol;Acc:HGNC:29788] |
| **BBS5** | 0.30 | 2.95 | 0.002 | 1 x 11 | 2 q31 | Bardet-Biedl syndrome 5 [Source:HGNC Symbol;Acc:HGNC:970] |
| **RGS12** | 0.30 | 2.95 | 0.002 | 1 x 9 | 4 p16 | regulator of G-protein signaling 12 [Source:HGNC Symbol;Acc:HGNC:9994] |
| **PMVK** | 0.30 | 2.94 | 0.002 | 4 x 10 | 1 q21 | phosphomevalonate kinase [Source:HGNC Symbol;Acc:HGNC:9141] |
| **RCHY1** | 0.29 | 2.92 | 0.002 | 2 x 13 | 4 q21 | ring finger and CHY zinc finger domain containing 1, E3 ubiquitin protein ligase [Source:HGNC Symbol;Acc:HGNC:17479] |
| **SLC7A6OS** | 0.29 | 2.91 | 0.002 | 1 x 11 | 16 q22 | solute carrier family 7, member 6 opposite strand [Source:HGNC Symbol;Acc:HGNC:25807] |
| **RWDD4** | 0.29 | 2.91 | 0.002 | 3 x 7 | 4 q35 | RWD domain containing 4 [Source:HGNC Symbol;Acc:HGNC:23750] |
| **PRRC2C** | 0.29 | 2.91 | 0.002 | 5 x 11 | 1 q24 | proline-rich coiled-coil 2C [Source:HGNC Symbol;Acc:HGNC:24903] |
| **APH1A** | 0.29 | 2.91 | 0.002 | 1 x 11 | 1 q21 | APH1A gamma secretase subunit [Source:HGNC Symbol;Acc:HGNC:29509] |
| **HDHD3** | 0.29 | 2.91 | 0.002 | 1 x 13 | 9 q32 | haloacid dehalogenase-like hydrolase domain containing 3 [Source:HGNC Symbol;Acc:HGNC:28171] |
| **ATP5G1** | 0.29 | 2.90 | 0.002 | 3 x 7 | 17 q21 | ATP synthase, H+ transporting, mitochondrial Fo complex, subunit C1 (subunit 9) [Source:HGNC Symbol;Acc:HGNC:841] |
| **FBLN2** | 0.29 | 2.90 | 0.002 | 6 x 9 | 3 p25 | fibulin 2 [Source:HGNC Symbol;Acc:HGNC:3601] |
| **PDE3B** | 0.29 | 2.89 | 0.002 | 4 x 14 | 11 p15 | phosphodiesterase 3B, cGMP-inhibited [Source:HGNC Symbol;Acc:HGNC:8779] |
| **MSANTD3-TMEFF1** | 0.29 | 2.88 | 0.002 | 1 x 12 | 9 q31 | MSANTD3-TMEFF1 readthrough [Source:HGNC Symbol;Acc:HGNC:38838] |
| **USP54** | 0.29 | 2.88 | 0.002 | 4 x 11 | 10 q22 | ubiquitin specific peptidase 54 [Source:HGNC Symbol;Acc:HGNC:23513] |
| **GBA** | 0.29 | 2.88 | 0.002 | 4 x 12 | NA | glucosidase, beta, acid [Source:HGNC Symbol;Acc:HGNC:4177] |
| **JAG1** | 0.29 | 2.88 | 0.002 | 4 x 9 | 20 p12 | jagged 1 [Source:HGNC Symbol;Acc:HGNC:6188] |
| **PRKCH** | 0.29 | 2.88 | 0.003 | 1 x 12 | 14 q23 | protein kinase C, eta [Source:HGNC Symbol;Acc:HGNC:9403] |
| **SNX32** | 0.29 | 2.84 | 0.003 | 6 x 15 | 11 q13 | sorting nexin 32 [Source:HGNC Symbol;Acc:HGNC:26423] |
| **ELK4** | 0.29 | 2.84 | 0.003 | 3 x 10 | 1 q32 | ELK4, ETS-domain protein (SRF accessory protein 1) [Source:HGNC Symbol;Acc:HGNC:3326] |
| **H1F0** | 0.29 | 2.84 | 0.003 | 1 x 7 | 22 q13 | H1 histone family, member 0 [Source:HGNC Symbol;Acc:HGNC:4714] |
| **BAIAP2** | 0.29 | 2.83 | 0.003 | 1 x 12 | 17 q25 | BAI1-associated protein 2 [Source:HGNC Symbol;Acc:HGNC:947] |
| **CHP1** | 0.29 | 2.83 | 0.003 | 3 x 15 | 15 q15 | calcineurin-like EF-hand protein 1 [Source:HGNC Symbol;Acc:HGNC:17433] |
| **ENC1** | 0.29 | 2.83 | 0.003 | 3 x 14 | 5 q13 | ectodermal-neural cortex 1 (with BTB domain) [Source:HGNC Symbol;Acc:HGNC:3345] |
| **VPS18** | 0.29 | 2.83 | 0.003 | 1 x 9 | 15 q15 | vacuolar protein sorting 18 homolog (S. cerevisiae) [Source:HGNC Symbol;Acc:HGNC:15972] |
| **GPR19** | 0.28 | 2.81 | 0.003 | 2 x 12 | 12 p13 | G protein-coupled receptor 19 [Source:HGNC Symbol;Acc:HGNC:4473] |
| **C9orf41** | 0.28 | 2.81 | 0.003 | 2 x 15 | 9 q21 | chromosome 9 open reading frame 41 [Source:HGNC Symbol;Acc:HGNC:23435] |
| **BTBD1** | 0.28 | 2.81 | 0.003 | 6 x 12 | 15 q25 | BTB (POZ) domain containing 1 [Source:HGNC Symbol;Acc:HGNC:1120] |
| **TOM1** | 0.28 | 2.80 | 0.003 | 2 x 10 | 22 q12 | target of myb1 (chicken) [Source:HGNC Symbol;Acc:HGNC:11982] |
| **CARD14** | 0.28 | 2.80 | 0.003 | 3 x 7 | 17 q25 | caspase recruitment domain family, member 14 [Source:HGNC Symbol;Acc:HGNC:16446] |
| **ABCC5** | 0.28 | 2.79 | 0.003 | 1 x 7 | 3 q27 | ATP-binding cassette, sub-family C (CFTR/MRP), member 5 [Source:HGNC Symbol;Acc:HGNC:56] |
| **BHLHE40** | 0.28 | 2.79 | 0.003 | 3 x 12 | 3 p26 | basic helix-loop-helix family, member e40 [Source:HGNC Symbol;Acc:HGNC:1046] |
| **POMGNT1** | 0.28 | 2.78 | 0.003 | 1 x 9 | 1 p34 | protein O-linked mannose N-acetylglucosaminyltransferase 1 (beta 1,2-) [Source:HGNC Symbol;Acc:HGNC:19139] |
| **TPPP** | 0.28 | 2.78 | 0.003 | 6 x 14 | 5 p15 | tubulin polymerization promoting protein [Source:HGNC Symbol;Acc:HGNC:24164] |
| **ZNF18** | 0.28 | 2.78 | 0.003 | 2 x 8 | 17 p12 | zinc finger protein 18 [Source:HGNC Symbol;Acc:HGNC:12969] |
| **TRIM26** | 0.28 | 2.78 | 0.003 | 7 x 15 | NA | tripartite motif containing 26 [Source:HGNC Symbol;Acc:HGNC:12962] |
| **PIP5K1B** | 0.28 | 2.78 | 0.003 | 1 x 9 | 9 q21 | phosphatidylinositol-4-phosphate 5-kinase, type I, beta [Source:HGNC Symbol;Acc:HGNC:8995] |
| **WDR3** | 0.28 | 2.78 | 0.003 | 1 x 12 | 1 p12 | WD repeat domain 3 [Source:HGNC Symbol;Acc:HGNC:12755] |
| **PSME4** | 0.28 | 2.77 | 0.003 | 5 x 10 | 2 p16 | proteasome (prosome, macropain) activator subunit 4 [Source:HGNC Symbol;Acc:HGNC:20635] |
| **SOX6** | 0.28 | 2.77 | 0.003 | 4 x 12 | 11 p15 | SRY (sex determining region Y)-box 6 [Source:HGNC Symbol;Acc:HGNC:16421] |
| **ABCC2** | 0.28 | 2.76 | 0.003 | 1 x 9 | 10 q24 | ATP-binding cassette, sub-family C (CFTR/MRP), member 2 [Source:HGNC Symbol;Acc:HGNC:53] |
| **CNBD2** | 0.28 | 2.76 | 0.003 | 1 x 8 | 20 q11 | cyclic nucleotide binding domain containing 2 [Source:HGNC Symbol;Acc:HGNC:16145] |
| **TFRC** | 0.28 | 2.76 | 0.004 | 6 x 15 | 3 q29 | transferrin receptor [Source:HGNC Symbol;Acc:HGNC:11763] |
| **NR6A1** | 0.28 | 2.73 | 0.004 | 4 x 13 | 9 q33 | nuclear receptor subfamily 6, group A, member 1 [Source:HGNC Symbol;Acc:HGNC:7985] |
| **BCCIP** | 0.28 | 2.73 | 0.004 | 2 x 15 | 10 q26 | BRCA2 and CDKN1A interacting protein [Source:HGNC Symbol;Acc:HGNC:978] |
| **STAT6** | 0.28 | 2.73 | 0.004 | 3 x 13 | 12 q13 | signal transducer and activator of transcription 6, interleukin-4 induced [Source:HGNC Symbol;Acc:HGNC:11368] |
| **PPAP2B** | 0.28 | 2.72 | 0.004 | 3 x 16 | 1 p32 | phosphatidic acid phosphatase type 2B [Source:HGNC Symbol;Acc:HGNC:9229] |
| **BIN3** | 0.28 | 2.72 | 0.004 | 4 x 13 | 8 p21 | bridging integrator 3 [Source:HGNC Symbol;Acc:HGNC:1054] |
| **OSBPL8** | 0.27 | 2.70 | 0.004 | 1 x 16 | 12 q21 | oxysterol binding protein-like 8 [Source:HGNC Symbol;Acc:HGNC:16396] |
| **GTF2F2** | 0.27 | 2.69 | 0.004 | 4 x 9 | 13 q14 | general transcription factor IIF, polypeptide 2, 30kDa [Source:HGNC Symbol;Acc:HGNC:4653] |
| **VPS53** | 0.27 | 2.68 | 0.004 | 6 x 9 | 17 p13 | vacuolar protein sorting 53 homolog (S. cerevisiae) [Source:HGNC Symbol;Acc:HGNC:25608] |
| **GAS8** | 0.27 | 2.68 | 0.004 | 1 x 10 | 16 q24 | growth arrest-specific 8 [Source:HGNC Symbol;Acc:HGNC:4166] |
| **CAMTA2** | 0.27 | 2.67 | 0.004 | 1 x 8 | 17 p13 | calmodulin binding transcription activator 2 [Source:HGNC Symbol;Acc:HGNC:18807] |
| **RTKN** | 0.27 | 2.67 | 0.004 | 6 x 10 | 2 p13 | rhotekin [Source:HGNC Symbol;Acc:HGNC:10466] |
| **ANKRD44** | 0.27 | 2.67 | 0.004 | 1 x 15 | 2 q33 | ankyrin repeat domain 44 [Source:HGNC Symbol;Acc:HGNC:25259] |
| **GPR137B** | 0.27 | 2.66 | 0.005 | 3 x 13 | 1 q42 | G protein-coupled receptor 137B [Source:HGNC Symbol;Acc:HGNC:11862] |
| **TIAM1** | 0.27 | 2.65 | 0.005 | 1 x 10 | 21 q22 | T-cell lymphoma invasion and metastasis 1 [Source:HGNC Symbol;Acc:HGNC:11805] |
| **DNAJC25** | 0.27 | 2.65 | 0.005 | 1 x 7 | 9 q31 | DnaJ (Hsp40) homolog, subfamily C , member 25 [Source:HGNC Symbol;Acc:HGNC:34187] |
| **FARP1** | 0.27 | 2.65 | 0.005 | 1 x 11 | 13 q32 | FERM, RhoGEF (ARHGEF) and pleckstrin domain protein 1 (chondrocyte-derived) [Source:HGNC Symbol;Acc:HGNC:3591] |
| **WAPAL** | 0.27 | 2.64 | 0.005 | 4 x 12 | 10 q23 | wings apart-like homolog (Drosophila) [Source:HGNC Symbol;Acc:HGNC:23293] |
| **CCDC58** | 0.27 | 2.63 | 0.005 | 6 x 11 | 3 q21 | coiled-coil domain containing 58 [Source:HGNC Symbol;Acc:HGNC:31136] |
| **LNPEP** | 0.27 | 2.63 | 0.005 | 5 x 10 | 5 q15 | leucyl/cystinyl aminopeptidase [Source:HGNC Symbol;Acc:HGNC:6656] |
| **WDR82** | 0.27 | 2.63 | 0.005 | 6 x 11 | 3 p21 | WD repeat domain 82 [Source:HGNC Symbol;Acc:HGNC:28826] |
| **CIPC** | 0.27 | 2.63 | 0.005 | 1 x 8 | 14 q24 | CLOCK-interacting pacemaker [Source:HGNC Symbol;Acc:HGNC:20365] |
| **PKIB** | 0.27 | 2.62 | 0.005 | 3 x 13 | 6 q22 | protein kinase (cAMP-dependent, catalytic) inhibitor beta [Source:HGNC Symbol;Acc:HGNC:9018] |
| **COQ7** | 0.27 | 2.62 | 0.005 | 3 x 11 | 16 p12 | coenzyme Q7 homolog, ubiquinone (yeast) [Source:HGNC Symbol;Acc:HGNC:2244] |
| **LYRM9** | 0.27 | 2.62 | 0.005 | 1 x 12 | 17 q11 | LYR motif containing 9 [Source:HGNC Symbol;Acc:HGNC:27314] |
| **MRS2** | 0.27 | 2.62 | 0.005 | 1 x 13 | 6 p22 | MRS2 magnesium transporter [Source:HGNC Symbol;Acc:HGNC:13785] |
| **ZC3HC1** | 0.27 | 2.61 | 0.005 | 1 x 9 | 7 q32 | zinc finger, C3HC-type containing 1 [Source:HGNC Symbol;Acc:HGNC:29913] |
| **C6orf136** | 0.27 | 2.61 | 0.005 | 6 x 13 | NA | chromosome 6 open reading frame 136 [Source:HGNC Symbol;Acc:HGNC:21301] |
| **TRAF6** | 0.26 | 2.59 | 0.006 | 3 x 14 | 11 p12 | TNF receptor-associated factor 6, E3 ubiquitin protein ligase [Source:HGNC Symbol;Acc:HGNC:12036] |
| **FARP2** | 0.26 | 2.59 | 0.006 | 5 x 14 | 2 q37 | FERM, RhoGEF and pleckstrin domain protein 2 [Source:HGNC Symbol;Acc:HGNC:16460] |
| **FBXW9** | 0.26 | 2.58 | 0.006 | 4 x 9 | 19 p13 | F-box and WD repeat domain containing 9 [Source:HGNC Symbol;Acc:HGNC:28136] |
| **SNX1** | 0.26 | 2.58 | 0.006 | 1 x 8 | 15 q22 | sorting nexin 1 [Source:HGNC Symbol;Acc:HGNC:11172] |
| **RUNX2** | 0.26 | 2.58 | 0.006 | 6 x 13 | 6 p21 | runt-related transcription factor 2 [Source:HGNC Symbol;Acc:HGNC:10472] |
| **LCMT2** | 0.26 | 2.58 | 0.006 | 3 x 13 | 15 q15 | leucine carboxyl methyltransferase 2 [Source:HGNC Symbol;Acc:HGNC:17558] |
| **FAM168A** | 0.26 | 2.58 | 0.006 | 5 x 9 | 11 q13 | family with sequence similarity 168, member A [Source:HGNC Symbol;Acc:HGNC:28999] |
| **RAB3A** | 0.26 | 2.57 | 0.006 | 3 x 7 | 19 p13 | RAB3A, member RAS oncogene family [Source:HGNC Symbol;Acc:HGNC:9777] |
| **TAF13** | 0.26 | 2.57 | 0.006 | 7 x 11 | 1 p13 | TAF13 RNA polymerase II, TATA box binding protein (TBP)-associated factor, 18kDa [Source:HGNC Symbol;Acc:HGNC:11546] |
| **ZSCAN21** | 0.26 | 2.56 | 0.006 | 3 x 13 | 7 q22 | zinc finger and SCAN domain containing 21 [Source:HGNC Symbol;Acc:HGNC:13104] |
| **FCRLA** | 0.26 | 2.55 | 0.006 | 1 x 8 | 1 q23 | Fc receptor-like A [Source:HGNC Symbol;Acc:HGNC:18504] |
| **SORD** | 0.26 | 2.55 | 0.006 | 1 x 11 | 15 q21 | sorbitol dehydrogenase [Source:HGNC Symbol;Acc:HGNC:11184] |
| **RDH14** | 0.26 | 2.55 | 0.006 | 4 x 9 | 2 p24 | retinol dehydrogenase 14 (all-trans/9-cis/11-cis) [Source:HGNC Symbol;Acc:HGNC:19979] |
| **CAMSAP1** | 0.26 | 2.55 | 0.006 | 5 x 13 | 9 q34 | calmodulin regulated spectrin-associated protein 1 [Source:HGNC Symbol;Acc:HGNC:19946] |
| **SUCO** | 0.26 | 2.54 | 0.006 | 1 x 7 | 1 q24 | SUN domain containing ossification factor [Source:HGNC Symbol;Acc:HGNC:1240] |
| **MYO1D** | 0.26 | 2.54 | 0.006 | 1 x 12 | 17 q11 | myosin ID [Source:HGNC Symbol;Acc:HGNC:7598] |
| **TXNDC16** | 0.26 | 2.54 | 0.006 | 3 x 11 | 14 q22 | thioredoxin domain containing 16 [Source:HGNC Symbol;Acc:HGNC:19965] |
| **ARID1B** | 0.26 | 2.53 | 0.007 | 5 x 14 | 6 q25 | AT rich interactive domain 1B (SWI1-like) [Source:HGNC Symbol;Acc:HGNC:18040] |
| **PTPDC1** | 0.26 | 2.53 | 0.007 | 3 x 15 | 9 q22 | protein tyrosine phosphatase domain containing 1 [Source:HGNC Symbol;Acc:HGNC:30184] |
| **PLEKHA2** | 0.26 | 2.52 | 0.007 | 3 x 8 | 8 p11 | pleckstrin homology domain containing, family A (phosphoinositide binding specific) member 2 [Source:HGNC Symbol;Acc:HGNC:14336] |
| **CYP27A1** | 0.26 | 2.51 | 0.007 | 4 x 14 | 2 q35 | cytochrome P450, family 27, subfamily A, polypeptide 1 [Source:HGNC Symbol;Acc:HGNC:2605] |
| **TMEM133** | 0.26 | 2.51 | 0.007 | 3 x 14 | 11 q22 | transmembrane protein 133 [Source:HGNC Symbol;Acc:HGNC:24033] |
| **SSPN** | 0.26 | 2.50 | 0.007 | 4 x 13 | 12 p12 | sarcospan [Source:HGNC Symbol;Acc:HGNC:11322] |
| **MTHFD2L** | 0.26 | 2.50 | 0.007 | 2 x 8 | 4 q13 | methylenetetrahydrofolate dehydrogenase (NADP+ dependent) 2-like [Source:HGNC Symbol;Acc:HGNC:31865] |
| **PDE4D** | 0.25 | 2.50 | 0.007 | 3 x 8 | 5 q12 | phosphodiesterase 4D, cAMP-specific [Source:HGNC Symbol;Acc:HGNC:8783] |
| **PLEKHH1** | 0.25 | 2.49 | 0.007 | 2 x 13 | 14 q24 | pleckstrin homology domain containing, family H (with MyTH4 domain) member 1 [Source:HGNC Symbol;Acc:HGNC:17733] |
| **FAM69C** | 0.25 | 2.49 | 0.007 | 1 x 14 | 18 q22 | family with sequence similarity 69, member C [Source:HGNC Symbol;Acc:HGNC:31729] |
| **ZNF16** | 0.25 | 2.49 | 0.007 | 1 x 16 | 8 q24 | zinc finger protein 16 [Source:HGNC Symbol;Acc:HGNC:12947] |
| **TKTL1** | 0.25 | 2.49 | 0.007 | 4 x 15 | X q28 | transketolase-like 1 [Source:HGNC Symbol;Acc:HGNC:11835] |
| **CCDC71L** | 0.25 | 2.49 | 0.007 | 5 x 16 | 7 q22 | coiled-coil domain containing 71-like [Source:HGNC Symbol;Acc:HGNC:26685] |
| **SMEK1** | 0.25 | 2.49 | 0.007 | 1 x 13 | 14 q32 | SMEK homolog 1, suppressor of mek1 (Dictyostelium) [Source:HGNC Symbol;Acc:HGNC:20219] |
| **TSPAN4** | 0.25 | 2.49 | 0.007 | 6 x 13 | 11 p15 | tetraspanin 4 [Source:HGNC Symbol;Acc:HGNC:11859] |
| **USO1** | 0.25 | 2.48 | 0.007 | 4 x 14 | 4 q21 | USO1 vesicle transport factor [Source:HGNC Symbol;Acc:HGNC:30904] |
| **LYRM2** | 0.25 | 2.48 | 0.008 | 1 x 16 | 6 q15 | LYR motif containing 2 [Source:HGNC Symbol;Acc:HGNC:25229] |
| **UBE3C** | 0.25 | 2.47 | 0.008 | 4 x 8 | 7 q36 | ubiquitin protein ligase E3C [Source:HGNC Symbol;Acc:HGNC:16803] |
| **KIAA1456** | 0.25 | 2.47 | 0.008 | 2 x 14 | 8 p22 | KIAA1456 [Source:HGNC Symbol;Acc:HGNC:26725] |
| **BID** | 0.25 | 2.47 | 0.008 | 1 x 16 | 22 q11 | BH3 interacting domain death agonist [Source:HGNC Symbol;Acc:HGNC:1050] |
| **PRAME** | 0.25 | 2.47 | 0.008 | 4 x 8 | 22 q11 | preferentially expressed antigen in melanoma [Source:HGNC Symbol;Acc:HGNC:9336] |
| **MARK1** | 0.25 | 2.46 | 0.008 | 1 x 9 | 1 q41 | MAP/microtubule affinity-regulating kinase 1 [Source:HGNC Symbol;Acc:HGNC:6896] |
| **ANKLE2** | 0.25 | 2.46 | 0.008 | 1 x 14 | 12 q24 | ankyrin repeat and LEM domain containing 2 [Source:HGNC Symbol;Acc:HGNC:29101] |
| **ORC4** | 0.25 | 2.46 | 0.008 | 4 x 10 | 2 q23 | origin recognition complex, subunit 4 [Source:HGNC Symbol;Acc:HGNC:8490] |
| **SPACA1** | 0.25 | 2.45 | 0.008 | 1 x 14 | 6 q15 | sperm acrosome associated 1 [Source:HGNC Symbol;Acc:HGNC:14967] |
| **NOP14** | 0.25 | 2.44 | 0.008 | 4 x 14 | 4 p16 | NOP14 nucleolar protein [Source:HGNC Symbol;Acc:HGNC:16821] |
| **HEATR6** | 0.25 | 2.44 | 0.008 | 4 x 10 | 17 q23 | HEAT repeat containing 6 [Source:HGNC Symbol;Acc:HGNC:24076] |
| **PRKAB1** | 0.25 | 2.44 | 0.008 | 5 x 11 | 12 q24 | protein kinase, AMP-activated, beta 1 non-catalytic subunit [Source:HGNC Symbol;Acc:HGNC:9378] |
| **FUBP3** | 0.25 | 2.43 | 0.009 | 1 x 8 | 9 q34 | far upstream element (FUSE) binding protein 3 [Source:HGNC Symbol;Acc:HGNC:4005] |
| **ZSCAN9** | 0.25 | 2.43 | 0.009 | 1 x 7 | 6 p22 | zinc finger and SCAN domain containing 9 [Source:HGNC Symbol;Acc:HGNC:12984] |
| **CLUH** | 0.25 | 2.42 | 0.009 | 2 x 15 | 17 p13 | clustered mitochondria (cluA/CLU1) homolog [Source:HGNC Symbol;Acc:HGNC:29094] |
| **MEF2C** | 0.25 | 2.42 | 0.009 | 5 x 15 | 5 q14 | myocyte enhancer factor 2C [Source:HGNC Symbol;Acc:HGNC:6996] |
| **EPOR** | 0.25 | 2.41 | 0.009 | 5 x 8 | 19 p13 | erythropoietin receptor [Source:HGNC Symbol;Acc:HGNC:3416] |
| **R3HCC1L** | 0.25 | 2.41 | 0.009 | 5 x 14 | 10 q24 | R3H domain and coiled-coil containing 1-like [Source:HGNC Symbol;Acc:HGNC:23512] |
| **SLC44A5** | 0.25 | 2.40 | 0.009 | 4 x 16 | 1 p31 | solute carrier family 44, member 5 [Source:HGNC Symbol;Acc:HGNC:28524] |
| **KAZN** | 0.25 | 2.40 | 0.009 | 5 x 10 | 1 p36 | kazrin, periplakin interacting protein [Source:HGNC Symbol;Acc:HGNC:29173] |
| **QRICH1** | 0.24 | 2.39 | 0.009 | 1 x 8 | 3 p21 | glutamine-rich 1 [Source:HGNC Symbol;Acc:HGNC:24713] |
| **CMTR2** | 0.24 | 2.39 | 0.009 | 5 x 12 | 16 q22 | cap methyltransferase 2 [Source:HGNC Symbol;Acc:HGNC:25635] |
| **FEM1B** | 0.24 | 2.39 | 0.010 | 5 x 11 | 15 q23 | fem-1 homolog b (C. elegans) [Source:HGNC Symbol;Acc:HGNC:3649] |
| **ITPK1** | 0.24 | 2.39 | 0.010 | 6 x 16 | NA | inositol-tetrakisphosphate 1-kinase [Source:HGNC Symbol;Acc:HGNC:6177] |
| **CS** | 0.24 | 2.39 | 0.010 | 6 x 9 | 12 q13 | citrate synthase [Source:HGNC Symbol;Acc:HGNC:2422] |
| **ERAP2** | 0.24 | 2.38 | 0.010 | 5 x 11 | 5 q15 | endoplasmic reticulum aminopeptidase 2 [Source:HGNC Symbol;Acc:HGNC:29499] |
| **SLC7A8** | 0.24 | 2.38 | 0.010 | 2 x 15 | 14 q11 | solute carrier family 7 (amino acid transporter light chain, L system), member 8 [Source:HGNC Symbol;Acc:HGNC:11066] |
| **PPP1R3B** | 0.24 | 2.37 | 0.010 | 5 x 10 | 8 p23 | protein phosphatase 1, regulatory subunit 3B [Source:HGNC Symbol;Acc:HGNC:14942] |
| **POGK** | 0.24 | 2.37 | 0.010 | 4 x 14 | 1 q24 | pogo transposable element with KRAB domain [Source:HGNC Symbol;Acc:HGNC:18800] |
| **VCPIP1** | 0.24 | 2.37 | 0.010 | 6 x 13 | 8 q13 | valosin containing protein (p97)/p47 complex interacting protein 1 [Source:HGNC Symbol;Acc:HGNC:30897] |
| **FZD3** | 0.24 | 2.36 | 0.010 | 4 x 13 | 8 p21 | frizzled class receptor 3 [Source:HGNC Symbol;Acc:HGNC:4041] |
| **FEZ1** | 0.24 | 2.36 | 0.010 | 1 x 7 | 11 q24 | fasciculation and elongation protein zeta 1 (zygin I) [Source:HGNC Symbol;Acc:HGNC:3659] |
| **ZNF827** | 0.24 | 2.36 | 0.010 | 6 x 12 | 4 q31 | zinc finger protein 827 [Source:HGNC Symbol;Acc:HGNC:27193] |
| **SULT1C2** | 0.24 | 2.36 | 0.010 | 1 x 9 | 2 q12 | sulfotransferase family, cytosolic, 1C, member 2 [Source:HGNC Symbol;Acc:HGNC:11456] |
| **KIF3C** | 0.24 | 2.35 | 0.010 | 1 x 12 | 2 p23 | kinesin family member 3C [Source:HGNC Symbol;Acc:HGNC:6321] |
| **SLC37A1** | 0.24 | 2.35 | 0.010 | 1 x 7 | 21 q22 | solute carrier family 37 (glucose-6-phosphate transporter), member 1 [Source:HGNC Symbol;Acc:HGNC:11024] |
| **MRPS14** | 0.24 | 2.35 | 0.011 | 6 x 13 | 1 q25 | mitochondrial ribosomal protein S14 [Source:HGNC Symbol;Acc:HGNC:14049] |
| **SUOX** | 0.24 | 2.34 | 0.011 | 1 x 12 | 12 q13 | sulfite oxidase [Source:HGNC Symbol;Acc:HGNC:11460] |
| **MRPS25** | 0.24 | 2.33 | 0.011 | 2 x 16 | 3 p25 | mitochondrial ribosomal protein S25 [Source:HGNC Symbol;Acc:HGNC:14511] |
| **UBA1** | 0.24 | 2.33 | 0.011 | 1 x 15 | X p11 | ubiquitin-like modifier activating enzyme 1 [Source:HGNC Symbol;Acc:HGNC:12469] |
| **SMIM4** | 0.24 | 2.32 | 0.011 | 4 x 14 | 3 p21 | small integral membrane protein 4 [Source:HGNC Symbol;Acc:HGNC:37257] |
| **GNG10** | 0.24 | 2.32 | 0.011 | 1 x 14 | 9 q31 | guanine nucleotide binding protein (G protein), gamma 10 [Source:HGNC Symbol;Acc:HGNC:4402] |
| **MYC** | 0.24 | 2.32 | 0.011 | 5 x 15 | 8 q24 | v-myc avian myelocytomatosis viral oncogene homolog [Source:HGNC Symbol;Acc:HGNC:7553] |
| **MTSS1** | 0.24 | 2.31 | 0.012 | 5 x 16 | 8 q24 | metastasis suppressor 1 [Source:HGNC Symbol;Acc:HGNC:20443] |
| **TP53RK** | 0.24 | 2.30 | 0.012 | 1 x 16 | 20 q13 | TP53 regulating kinase [Source:HGNC Symbol;Acc:HGNC:16197] |
| **BEAN1** | 0.24 | 2.30 | 0.012 | 3 x 14 | 16 q21 | brain expressed, associated with NEDD4, 1 [Source:HGNC Symbol;Acc:HGNC:24160] |
| **MRPS2** | 0.23 | 2.29 | 0.012 | 1 x 13 | 9 q34 | mitochondrial ribosomal protein S2 [Source:HGNC Symbol;Acc:HGNC:14495] |
| **LRPPRC** | 0.23 | 2.29 | 0.012 | 3 x 8 | 2 p21 | leucine-rich pentatricopeptide repeat containing [Source:HGNC Symbol;Acc:HGNC:15714] |
| **USP38** | 0.23 | 2.29 | 0.012 | 1 x 7 | 4 q31 | ubiquitin specific peptidase 38 [Source:HGNC Symbol;Acc:HGNC:20067] |
| **UBE2G2** | 0.23 | 2.29 | 0.012 | 7 x 14 | 21 q22 | ubiquitin-conjugating enzyme E2G 2 [Source:HGNC Symbol;Acc:HGNC:12483] |
| **UBE2L3** | 0.23 | 2.28 | 0.012 | 5 x 13 | 22 q11 | ubiquitin-conjugating enzyme E2L 3 [Source:HGNC Symbol;Acc:HGNC:12488] |
| **RMDN3** | 0.23 | 2.28 | 0.012 | 3 x 11 | 15 q15 | regulator of microtubule dynamics 3 [Source:HGNC Symbol;Acc:HGNC:25550] |
| **ZNF559-ZNF177** | 0.23 | 2.27 | 0.013 | 4 x 16 | 19 p13 | ZNF559-ZNF177 readthrough [Source:HGNC Symbol;Acc:HGNC:42964] |
| **ACADSB** | 0.23 | 2.27 | 0.013 | 1 x 8 | 10 q26 | acyl-CoA dehydrogenase, short/branched chain [Source:HGNC Symbol;Acc:HGNC:91] |
| **MSANTD2** | 0.23 | 2.27 | 0.013 | 6 x 11 | 11 q24 | Myb/SANT-like DNA-binding domain containing 2 [Source:HGNC Symbol;Acc:HGNC:26266] |
| **C12orf73** | 0.23 | 2.27 | 0.013 | 1 x 10 | 12 q23 | chromosome 12 open reading frame 73 [Source:HGNC Symbol;Acc:HGNC:34450] |
| **DRG2** | 0.23 | 2.27 | 0.013 | 1 x 8 | 17 p11 | developmentally regulated GTP binding protein 2 [Source:HGNC Symbol;Acc:HGNC:3030] |
| **NXN** | 0.23 | 2.26 | 0.013 | 6 x 15 | 17 p13 | nucleoredoxin [Source:HGNC Symbol;Acc:HGNC:18008] |
| **UBQLN4** | 0.23 | 2.26 | 0.013 | 4 x 14 | 1 q22 | ubiquilin 4 [Source:HGNC Symbol;Acc:HGNC:1237] |
| **GIPC1** | 0.23 | 2.26 | 0.013 | 5 x 12 | 19 p13 | GIPC PDZ domain containing family, member 1 [Source:HGNC Symbol;Acc:HGNC:1226] |
| **ZNF44** | 0.23 | 2.26 | 0.013 | 3 x 12 | 19 p13 | zinc finger protein 44 [Source:HGNC Symbol;Acc:HGNC:13110] |
| **MSMO1** | 0.23 | 2.25 | 0.013 | 1 x 8 | 4 q32 | methylsterol monooxygenase 1 [Source:HGNC Symbol;Acc:HGNC:10545] |
| **NOP2** | 0.23 | 2.24 | 0.014 | 6 x 12 | 12 p13 | NOP2 nucleolar protein [Source:HGNC Symbol;Acc:HGNC:7867] |
| **BCOR** | 0.23 | 2.24 | 0.014 | 2 x 13 | X p11 | BCL6 corepressor [Source:HGNC Symbol;Acc:HGNC:20893] |
| **KIAA0430** | 0.23 | 2.23 | 0.014 | 1 x 8 | NA | KIAA0430 [Source:HGNC Symbol;Acc:HGNC:29562] |
| **RASGEF1B** | 0.23 | 2.23 | 0.014 | 6 x 14 | 4 q21 | RasGEF domain family, member 1B [Source:HGNC Symbol;Acc:HGNC:24881] |
| **CYP7B1** | 0.23 | 2.23 | 0.014 | 2 x 14 | 8 q12 | cytochrome P450, family 7, subfamily B, polypeptide 1 [Source:HGNC Symbol;Acc:HGNC:2652] |
| **CNTN3** | 0.23 | 2.22 | 0.014 | 6 x 9 | 3 p12 | contactin 3 (plasmacytoma associated) [Source:HGNC Symbol;Acc:HGNC:2173] |
| **FOXN2** | 0.23 | 2.22 | 0.015 | 1 x 13 | 2 p16 | forkhead box N2 [Source:HGNC Symbol;Acc:HGNC:5281] |
| **LIN52** | 0.23 | 2.21 | 0.015 | 1 x 13 | 14 q24 | lin-52 DREAM MuvB core complex component [Source:HGNC Symbol;Acc:HGNC:19856] |
| **CRBN** | 0.23 | 2.20 | 0.015 | 1 x 8 | 3 p26 | cereblon [Source:HGNC Symbol;Acc:HGNC:30185] |
| **ECHDC2** | 0.23 | 2.20 | 0.015 | 1 x 14 | 1 p32 | enoyl CoA hydratase domain containing 2 [Source:HGNC Symbol;Acc:HGNC:23408] |
| **NFX1** | 0.23 | 2.20 | 0.015 | 5 x 10 | 9 p13 | nuclear transcription factor, X-box binding 1 [Source:HGNC Symbol;Acc:HGNC:7803] |
| **ARHGAP12** | 0.23 | 2.19 | 0.015 | 5 x 14 | 10 p11 | Rho GTPase activating protein 12 [Source:HGNC Symbol;Acc:HGNC:16348] |
| **CHST12** | 0.22 | 2.19 | 0.016 | 4 x 15 | 7 p22 | carbohydrate (chondroitin 4) sulfotransferase 12 [Source:HGNC Symbol;Acc:HGNC:17423] |
| **WDR48** | 0.22 | 2.19 | 0.016 | 1 x 9 | 3 p22 | WD repeat domain 48 [Source:HGNC Symbol;Acc:HGNC:30914] |
| **KMT2C** | 0.22 | 2.18 | 0.016 | 4 x 15 | 7 q36 | lysine (K)-specific methyltransferase 2C [Source:HGNC Symbol;Acc:HGNC:13726] |
| **SCRN2** | 0.22 | 2.18 | 0.016 | 5 x 15 | 17 q21 | secernin 2 [Source:HGNC Symbol;Acc:HGNC:30381] |
| **METTL16** | 0.22 | 2.18 | 0.016 | 1 x 10 | 17 p13 | methyltransferase like 16 [Source:HGNC Symbol;Acc:HGNC:28484] |
| **ACVR2A** | 0.22 | 2.18 | 0.016 | 1 x 15 | 2 q22 | activin A receptor, type IIA [Source:HGNC Symbol;Acc:HGNC:173] |
| **ROBO2** | 0.22 | 2.17 | 0.016 | 1 x 16 | 3 p12 | roundabout, axon guidance receptor, homolog 2 (Drosophila) [Source:HGNC Symbol;Acc:HGNC:10250] |
| **SVIL** | 0.22 | 2.17 | 0.016 | 1 x 7 | 10 p11 | supervillin [Source:HGNC Symbol;Acc:HGNC:11480] |
| **TDG** | 0.22 | 2.17 | 0.016 | 6 x 11 | 12 q23 | thymine-DNA glycosylase [Source:HGNC Symbol;Acc:HGNC:11700] |
| **DDX18** | 0.22 | 2.17 | 0.016 | 5 x 15 | 2 q14 | DEAD (Asp-Glu-Ala-Asp) box polypeptide 18 [Source:HGNC Symbol;Acc:HGNC:2741] |
| **PI4KB** | 0.22 | 2.16 | 0.017 | 5 x 9 | 1 q21 | phosphatidylinositol 4-kinase, catalytic, beta [Source:HGNC Symbol;Acc:HGNC:8984] |
| **C20orf196** | 0.22 | 2.16 | 0.017 | 1 x 7 | 20 p12 | chromosome 20 open reading frame 196 [Source:HGNC Symbol;Acc:HGNC:26318] |
| **ZNF559** | 0.22 | 2.16 | 0.017 | 3 x 15 | 19 p13 | zinc finger protein 559 [Source:HGNC Symbol;Acc:HGNC:28197] |
| **MRPS5** | 0.22 | 2.16 | 0.017 | 3 x 7 | 2 q11 | mitochondrial ribosomal protein S5 [Source:HGNC Symbol;Acc:HGNC:14498] |
| **ZDHHC16** | 0.22 | 2.14 | 0.018 | 1 x 7 | 10 q24 | zinc finger, DHHC-type containing 16 [Source:HGNC Symbol;Acc:HGNC:20714] |
| **CDH7** | 0.22 | 2.13 | 0.018 | 4 x 15 | 18 q22 | cadherin 7, type 2 [Source:HGNC Symbol;Acc:HGNC:1766] |
| **BLVRA** | 0.22 | 2.13 | 0.018 | 2 x 14 | 7 p13 | biliverdin reductase A [Source:HGNC Symbol;Acc:HGNC:1062] |
| **ARHGDIA** | 0.22 | 2.13 | 0.018 | 7 x 13 | 17 q25 | Rho GDP dissociation inhibitor (GDI) alpha [Source:HGNC Symbol;Acc:HGNC:678] |
| **PEX19** | 0.22 | 2.13 | 0.018 | 1 x 8 | 1 q23 | peroxisomal biogenesis factor 19 [Source:HGNC Symbol;Acc:HGNC:9713] |
| **SLC25A10** | 0.22 | 2.12 | 0.018 | 3 x 7 | 17 q25 | solute carrier family 25 (mitochondrial carrier; dicarboxylate transporter), member 10 [Source:HGNC Symbol;Acc:HGNC:10980] |
| **SEMA5A** | 0.22 | 2.12 | 0.018 | 4 x 9 | 5 p15 | sema domain, seven thrombospondin repeats (type 1 and type 1-like), transmembrane domain (TM) and short cytoplasmic domain, (semaphorin) 5A [Source:HGNC Symbol;Acc:HGNC:10736] |
| **IQCE** | 0.22 | 2.12 | 0.019 | 5 x 15 | 7 p22 | IQ motif containing E [Source:HGNC Symbol;Acc:HGNC:29171] |
| **ENTPD5** | 0.22 | 2.12 | 0.019 | 3 x 14 | 14 q24 | ectonucleoside triphosphate diphosphohydrolase 5 [Source:HGNC Symbol;Acc:HGNC:3367] |
| **BCL2** | 0.22 | 2.11 | 0.019 | 7 x 13 | 18 q21 | B-cell CLL/lymphoma 2 [Source:HGNC Symbol;Acc:HGNC:990] |
| **MADD** | 0.22 | 2.11 | 0.019 | 1 x 8 | 11 p11 | MAP-kinase activating death domain [Source:HGNC Symbol;Acc:HGNC:6766] |
| **RBM45** | 0.22 | 2.10 | 0.019 | 1 x 16 | 2 q31 | RNA binding motif protein 45 [Source:HGNC Symbol;Acc:HGNC:24468] |
| **CNOT6** | 0.22 | 2.10 | 0.019 | 7 x 12 | 5 q35 | CCR4-NOT transcription complex, subunit 6 [Source:HGNC Symbol;Acc:HGNC:14099] |
| **YAF2** | 0.22 | 2.09 | 0.020 | 1 x 13 | 12 q12 | YY1 associated factor 2 [Source:HGNC Symbol;Acc:HGNC:17363] |
| **SMAD1** | 0.21 | 2.09 | 0.020 | 4 x 16 | 4 q31 | SMAD family member 1 [Source:HGNC Symbol;Acc:HGNC:6767] |
| **SCIN** | 0.21 | 2.09 | 0.020 | 1 x 12 | 7 p21 | scinderin [Source:HGNC Symbol;Acc:HGNC:21695] |
| **ADD1** | 0.21 | 2.08 | 0.020 | 1 x 8 | 4 p16 | adducin 1 (alpha) [Source:HGNC Symbol;Acc:HGNC:243] |
| **TMED10** | 0.21 | 2.08 | 0.020 | 7 x 14 | 14 q24 | transmembrane emp24-like trafficking protein 10 (yeast) [Source:HGNC Symbol;Acc:HGNC:16998] |
| **ANKRD54** | 0.21 | 2.07 | 0.020 | 4 x 10 | 22 q13 | ankyrin repeat domain 54 [Source:HGNC Symbol;Acc:HGNC:25185] |
| **KIAA1109** | 0.21 | 2.07 | 0.021 | 3 x 15 | 4 q27 | KIAA1109 [Source:HGNC Symbol;Acc:HGNC:26953] |
| **SLC25A14** | 0.21 | 2.06 | 0.021 | 4 x 14 | X q26 | solute carrier family 25 (mitochondrial carrier, brain), member 14 [Source:HGNC Symbol;Acc:HGNC:10984] |
| **PMF1-BGLAP** | 0.21 | 2.06 | 0.021 | 3 x 16 | 1 q22 | PMF1-BGLAP readthrough [Source:HGNC Symbol;Acc:HGNC:42953] |
| **NCOA1** | 0.21 | 2.06 | 0.021 | 2 x 15 | 2 p23 | nuclear receptor coactivator 1 [Source:HGNC Symbol;Acc:HGNC:7668] |
| **AFG3L2** | 0.21 | 2.06 | 0.021 | 1 x 16 | 18 p11 | AFG3-like AAA ATPase 2 [Source:HGNC Symbol;Acc:HGNC:315] |
| **RCL1** | 0.21 | 2.06 | 0.021 | 6 x 13 | 9 p24 | RNA terminal phosphate cyclase-like 1 [Source:HGNC Symbol;Acc:HGNC:17687] |
| **SEMA3A** | 0.21 | 2.05 | 0.022 | 7 x 12 | 7 q21 | sema domain, immunoglobulin domain (Ig), short basic domain, secreted, (semaphorin) 3A [Source:HGNC Symbol;Acc:HGNC:10723] |
| **ARAP1** | 0.21 | 2.05 | 0.022 | 1 x 11 | 11 q13 | ArfGAP with RhoGAP domain, ankyrin repeat and PH domain 1 [Source:HGNC Symbol;Acc:HGNC:16925] |
| **CNTLN** | 0.21 | 2.05 | 0.022 | 6 x 14 | 9 p22 | centlein, centrosomal protein [Source:HGNC Symbol;Acc:HGNC:23432] |
| **TRAPPC3** | 0.21 | 2.04 | 0.022 | 1 x 8 | 1 p34 | trafficking protein particle complex 3 [Source:HGNC Symbol;Acc:HGNC:19942] |
| **MAFB** | 0.21 | 2.04 | 0.022 | 7 x 13 | 20 q12 | v-maf avian musculoaponeurotic fibrosarcoma oncogene homolog B [Source:HGNC Symbol;Acc:HGNC:6408] |
| **C18orf21** | 0.21 | 2.04 | 0.022 | 4 x 12 | 18 q12 | chromosome 18 open reading frame 21 [Source:HGNC Symbol;Acc:HGNC:28802] |
| **BICD2** | 0.21 | 2.04 | 0.022 | 7 x 14 | 9 q22 | bicaudal D homolog 2 (Drosophila) [Source:HGNC Symbol;Acc:HGNC:17208] |
| **OSBPL9** | 0.21 | 2.04 | 0.022 | 1 x 8 | 1 p32 | oxysterol binding protein-like 9 [Source:HGNC Symbol;Acc:HGNC:16386] |
| **CLYBL** | 0.21 | 2.03 | 0.023 | 5 x 15 | 13 q32 | citrate lyase beta like [Source:HGNC Symbol;Acc:HGNC:18355] |
| **OXNAD1** | 0.21 | 2.03 | 0.023 | 1 x 15 | 3 p25 | oxidoreductase NAD-binding domain containing 1 [Source:HGNC Symbol;Acc:HGNC:25128] |
| **PEX1** | 0.21 | 2.02 | 0.023 | 1 x 7 | 7 q21 | peroxisomal biogenesis factor 1 [Source:HGNC Symbol;Acc:HGNC:8850] |
| **RAB18** | 0.21 | 2.01 | 0.024 | 1 x 14 | 10 p12 | RAB18, member RAS oncogene family [Source:HGNC Symbol;Acc:HGNC:14244] |
| **TRMT10C** | 0.21 | 2.01 | 0.024 | 7 x 12 | 3 q12 | tRNA methyltransferase 10 homolog C (S. cerevisiae) [Source:HGNC Symbol;Acc:HGNC:26022] |
| **HIVEP3** | 0.21 | 2.01 | 0.024 | 1 x 15 | 1 p34 | human immunodeficiency virus type I enhancer binding protein 3 [Source:HGNC Symbol;Acc:HGNC:13561] |
| **IQCK** | 0.21 | 2.00 | 0.024 | 4 x 13 | 16 p12 | IQ motif containing K [Source:HGNC Symbol;Acc:HGNC:28556] |
| **SLC7A6** | 0.21 | 2.00 | 0.024 | 1 x 11 | 16 q22 | solute carrier family 7 (amino acid transporter light chain, y+L system), member 6 [Source:HGNC Symbol;Acc:HGNC:11064] |
| **CD109** | 0.21 | 1.99 | 0.025 | 1 x 8 | 6 q13 | CD109 molecule [Source:HGNC Symbol;Acc:HGNC:21685] |
| **OBFC1** | 0.21 | 1.99 | 0.025 | 4 x 13 | 10 q24 | oligonucleotide/oligosaccharide-binding fold containing 1 [Source:HGNC Symbol;Acc:HGNC:26200] |
| **ALAD** | 0.21 | 1.99 | 0.025 | 1 x 14 | 9 q32 | aminolevulinate dehydratase [Source:HGNC Symbol;Acc:HGNC:395] |
| **PIP4K2C** | 0.20 | 1.98 | 0.025 | 1 x 10 | 12 q13 | phosphatidylinositol-5-phosphate 4-kinase, type II, gamma [Source:HGNC Symbol;Acc:HGNC:23786] |
| **C14orf37** | 0.20 | 1.98 | 0.025 | 2 x 14 | 14 q23 | chromosome 14 open reading frame 37 [Source:HGNC Symbol;Acc:HGNC:19846] |
| **PIGX** | 0.20 | 1.98 | 0.025 | 1 x 16 | 3 q29 | phosphatidylinositol glycan anchor biosynthesis, class X [Source:HGNC Symbol;Acc:HGNC:26046] |
| **ZNF527** | 0.20 | 1.98 | 0.026 | 4 x 14 | 19 q13 | zinc finger protein 527 [Source:HGNC Symbol;Acc:HGNC:29385] |
| **FECH** | 0.20 | 1.97 | 0.026 | 1 x 8 | 18 q21 | ferrochelatase [Source:HGNC Symbol;Acc:HGNC:3647] |
| **KDR** | 0.20 | 1.96 | 0.026 | 5 x 15 | 4 q12 | kinase insert domain receptor [Source:HGNC Symbol;Acc:HGNC:6307] |
| **GAPVD1** | 0.20 | 1.95 | 0.027 | 5 x 11 | 9 q33 | GTPase activating protein and VPS9 domains 1 [Source:HGNC Symbol;Acc:HGNC:23375] |
| **PDHX** | 0.20 | 1.95 | 0.027 | 1 x 7 | 11 p13 | pyruvate dehydrogenase complex, component X [Source:HGNC Symbol;Acc:HGNC:21350] |
| **CLCN7** | 0.20 | 1.95 | 0.027 | 6 x 14 | 16 p13 | chloride channel, voltage-sensitive 7 [Source:HGNC Symbol;Acc:HGNC:2025] |
| **STARD3** | 0.20 | 1.94 | 0.027 | 1 x 15 | 17 q12 | StAR-related lipid transfer (START) domain containing 3 [Source:HGNC Symbol;Acc:HGNC:17579] |
| **RAB13** | 0.20 | 1.94 | 0.028 | 4 x 8 | 1 q21 | RAB13, member RAS oncogene family [Source:HGNC Symbol;Acc:HGNC:9762] |
| **TACR2** | 0.20 | 1.93 | 0.028 | 7 x 13 | 10 q22 | tachykinin receptor 2 [Source:HGNC Symbol;Acc:HGNC:11527] |
| **SLC33A1** | 0.20 | 1.93 | 0.029 | 5 x 12 | 3 q25 | solute carrier family 33 (acetyl-CoA transporter), member 1 [Source:HGNC Symbol;Acc:HGNC:95] |
| **DLD** | 0.20 | 1.93 | 0.029 | 1 x 7 | 7 q31 | dihydrolipoamide dehydrogenase [Source:HGNC Symbol;Acc:HGNC:2898] |
| **AZI2** | 0.20 | 1.92 | 0.029 | 1 x 13 | 3 p24 | 5-azacytidine induced 2 [Source:HGNC Symbol;Acc:HGNC:24002] |
| **ARFGEF1** | 0.20 | 1.92 | 0.029 | 1 x 15 | 8 q13 | ADP-ribosylation factor guanine nucleotide-exchange factor 1 (brefeldin A-inhibited) [Source:HGNC Symbol;Acc:HGNC:15772] |
| **FASTKD1** | 0.20 | 1.91 | 0.030 | 1 x 8 | 2 q31 | FAST kinase domains 1 [Source:HGNC Symbol;Acc:HGNC:26150] |
| **HIAT1** | 0.20 | 1.91 | 0.030 | 1 x 16 | 1 p21 | hippocampus abundant transcript 1 [Source:HGNC Symbol;Acc:HGNC:23363] |
| **VCPKMT** | 0.20 | 1.90 | 0.030 | 1 x 7 | 14 q21 | valosin containing protein lysine (K) methyltransferase [Source:HGNC Symbol;Acc:HGNC:20352] |
| **CINP** | 0.20 | 1.89 | 0.031 | 1 x 16 | 14 q32 | cyclin-dependent kinase 2 interacting protein [Source:HGNC Symbol;Acc:HGNC:23789] |
| **PDXDC1** | 0.19 | 1.89 | 0.031 | 1 x 7 | NA | pyridoxal-dependent decarboxylase domain containing 1 [Source:HGNC Symbol;Acc:HGNC:28995] |
| **GFOD1** | 0.19 | 1.88 | 0.031 | 5 x 15 | 6 p23 | glucose-fructose oxidoreductase domain containing 1 [Source:HGNC Symbol;Acc:HGNC:21096] |
| **PRICKLE1** | 0.19 | 1.88 | 0.032 | 5 x 16 | 12 q12 | prickle homolog 1 (Drosophila) [Source:HGNC Symbol;Acc:HGNC:17019] |
| **DPP7** | 0.19 | 1.88 | 0.032 | 1 x 15 | 9 q34 | dipeptidyl-peptidase 7 [Source:HGNC Symbol;Acc:HGNC:14892] |
| **PARP8** | 0.19 | 1.87 | 0.032 | 1 x 15 | 5 q11 | poly (ADP-ribose) polymerase family, member 8 [Source:HGNC Symbol;Acc:HGNC:26124] |
| **SLC25A32** | 0.19 | 1.87 | 0.032 | 1 x 15 | 8 q22 | solute carrier family 25 (mitochondrial folate carrier), member 32 [Source:HGNC Symbol;Acc:HGNC:29683] |
| **ATXN3** | 0.19 | 1.87 | 0.032 | 1 x 8 | 14 q32 | ataxin 3 [Source:HGNC Symbol;Acc:HGNC:7106] |
| **PTRH2** | 0.19 | 1.86 | 0.033 | 6 x 11 | 17 q23 | peptidyl-tRNA hydrolase 2 [Source:HGNC Symbol;Acc:HGNC:24265] |
| **PPP2R1B** | 0.19 | 1.85 | 0.033 | 1 x 14 | 11 q23 | protein phosphatase 2, regulatory subunit A, beta [Source:HGNC Symbol;Acc:HGNC:9303] |
| **CC2D1B** | 0.19 | 1.84 | 0.034 | 1 x 9 | 1 p32 | coiled-coil and C2 domain containing 1B [Source:HGNC Symbol;Acc:HGNC:29386] |
| **RBM7** | 0.19 | 1.84 | 0.034 | 3 x 13 | 11 q23 | RNA binding motif protein 7 [Source:HGNC Symbol;Acc:HGNC:9904] |
| **IPO4** | 0.19 | 1.84 | 0.035 | 6 x 10 | 14 q12 | importin 4 [Source:HGNC Symbol;Acc:HGNC:19426] |
| **RAPGEF6** | 0.19 | 1.84 | 0.035 | 1 x 10 | 5 q31 | Rap guanine nucleotide exchange factor (GEF) 6 [Source:HGNC Symbol;Acc:HGNC:20655] |
| **TRIP4** | 0.19 | 1.83 | 0.035 | 1 x 9 | 15 q22 | thyroid hormone receptor interactor 4 [Source:HGNC Symbol;Acc:HGNC:12310] |
| **INPP4B** | 0.19 | 1.83 | 0.035 | 5 x 15 | 4 q31 | inositol polyphosphate-4-phosphatase, type II, 105kDa [Source:HGNC Symbol;Acc:HGNC:6075] |
| **DCTN5** | 0.19 | 1.82 | 0.036 | 4 x 11 | 16 p12 | dynactin 5 (p25) [Source:HGNC Symbol;Acc:HGNC:24594] |
| **WDFY1** | 0.19 | 1.82 | 0.036 | 1 x 12 | 2 q36 | WD repeat and FYVE domain containing 1 [Source:HGNC Symbol;Acc:HGNC:20451] |
| **C9orf91** | 0.19 | 1.82 | 0.036 | 1 x 16 | 9 q32 | chromosome 9 open reading frame 91 [Source:HGNC Symbol;Acc:HGNC:24513] |
| **BRAT1** | 0.19 | 1.82 | 0.036 | 6 x 15 | 7 p22 | BRCA1-associated ATM activator 1 [Source:HGNC Symbol;Acc:HGNC:21701] |
| **TWF2** | 0.19 | 1.81 | 0.036 | 5 x 16 | 3 p21 | twinfilin actin-binding protein 2 [Source:HGNC Symbol;Acc:HGNC:9621] |
| **SUPT4H1** | 0.19 | 1.81 | 0.037 | 6 x 9 | 17 q22 | suppressor of Ty 4 homolog 1 (S. cerevisiae) [Source:HGNC Symbol;Acc:HGNC:11467] |
| **NOLC1** | 0.19 | 1.81 | 0.037 | 1 x 16 | 10 q24 | nucleolar and coiled-body phosphoprotein 1 [Source:HGNC Symbol;Acc:HGNC:15608] |
| **ERN1** | 0.19 | 1.80 | 0.037 | 7 x 14 | 17 q23 | endoplasmic reticulum to nucleus signaling 1 [Source:HGNC Symbol;Acc:HGNC:3449] |
| **TRIM69** | 0.19 | 1.80 | 0.038 | 1 x 7 | NA | tripartite motif containing 69 [Source:HGNC Symbol;Acc:HGNC:17857] |
| **SUSD5** | 0.19 | 1.79 | 0.038 | 6 x 16 | 3 p22 | sushi domain containing 5 [Source:HGNC Symbol;Acc:HGNC:29061] |
| **APEX2** | 0.19 | 1.79 | 0.038 | 3 x 8 | X p11 | APEX nuclease (apurinic/apyrimidinic endonuclease) 2 [Source:HGNC Symbol;Acc:HGNC:17889] |
| **TAF1C** | 0.18 | 1.78 | 0.039 | 6 x 9 | 16 q24 | TATA box binding protein (TBP)-associated factor, RNA polymerase I, C, 110kDa [Source:HGNC Symbol;Acc:HGNC:11534] |
| **POLB** | 0.18 | 1.78 | 0.039 | 1 x 9 | 8 p11 | polymerase (DNA directed), beta [Source:HGNC Symbol;Acc:HGNC:9174] |
| **CAB39L** | 0.18 | 1.78 | 0.039 | 3 x 8 | 13 q14 | calcium binding protein 39-like [Source:HGNC Symbol;Acc:HGNC:20290] |
| **DBNDD1** | 0.18 | 1.77 | 0.040 | 3 x 7 | 16 q24 | dysbindin (dystrobrevin binding protein 1) domain containing 1 [Source:HGNC Symbol;Acc:HGNC:28455] |
| **BMPR1B** | 0.18 | 1.77 | 0.040 | 6 x 13 | 4 q22 | bone morphogenetic protein receptor, type IB [Source:HGNC Symbol;Acc:HGNC:1077] |
| **SIRT3** | 0.18 | 1.76 | 0.041 | 4 x 16 | 11 p15 | sirtuin 3 [Source:HGNC Symbol;Acc:HGNC:14931] |
| **TSPAN14** | 0.18 | 1.76 | 0.041 | 6 x 12 | 10 q23 | tetraspanin 14 [Source:HGNC Symbol;Acc:HGNC:23303] |
| **MKS1** | 0.18 | 1.75 | 0.042 | 3 x 13 | 17 q22 | Meckel syndrome, type 1 [Source:HGNC Symbol;Acc:HGNC:7121] |
| **ANGEL2** | 0.18 | 1.75 | 0.042 | 1 x 7 | 1 q32 | angel homolog 2 (Drosophila) [Source:HGNC Symbol;Acc:HGNC:30534] |
| **MAEA** | 0.18 | 1.73 | 0.043 | 1 x 14 | 4 p16 | macrophage erythroblast attacher [Source:HGNC Symbol;Acc:HGNC:13731] |
| **ST3GAL6** | 0.18 | 1.72 | 0.044 | 4 x 8 | 3 q12 | ST3 beta-galactoside alpha-2,3-sialyltransferase 6 [Source:HGNC Symbol;Acc:HGNC:18080] |
| **MEF2BNB** | 0.18 | 1.72 | 0.045 | 1 x 16 | 19 p13 | MEF2B neighbor [Source:HGNC Symbol;Acc:HGNC:37247] |
| **UBA6** | 0.18 | 1.72 | 0.045 | 6 x 10 | 4 q13 | ubiquitin-like modifier activating enzyme 6 [Source:HGNC Symbol;Acc:HGNC:25581] |
| **RBM3** | 0.18 | 1.71 | 0.045 | 5 x 15 | X p11 | RNA binding motif (RNP1, RRM) protein 3 [Source:HGNC Symbol;Acc:HGNC:9900] |
| **GAA** | 0.18 | 1.71 | 0.046 | 1 x 7 | 17 q25 | glucosidase, alpha; acid [Source:HGNC Symbol;Acc:HGNC:4065] |
| **DAP3** | 0.18 | 1.70 | 0.046 | 7 x 11 | 1 q22 | death associated protein 3 [Source:HGNC Symbol;Acc:HGNC:2673] |
| **ARHGAP42** | 0.18 | 1.70 | 0.046 | 6 x 14 | 11 q22 | Rho GTPase activating protein 42 [Source:HGNC Symbol;Acc:HGNC:26545] |
| **SFSWAP** | 0.18 | 1.69 | 0.047 | 2 x 7 | 12 q24 | splicing factor, suppressor of white-apricot family [Source:HGNC Symbol;Acc:HGNC:10790] |
| **SLMAP** | 0.18 | 1.69 | 0.047 | 1 x 10 | 3 p14 | sarcolemma associated protein [Source:HGNC Symbol;Acc:HGNC:16643] |
| **CDK2AP1** | 0.18 | 1.69 | 0.047 | 5 x 14 | 12 q24 | cyclin-dependent kinase 2 associated protein 1 [Source:HGNC Symbol;Acc:HGNC:14002] |
| **KCTD21** | 0.17 | 1.69 | 0.048 | 3 x 9 | 11 q14 | potassium channel tetramerization domain containing 21 [Source:HGNC Symbol;Acc:HGNC:27452] |
| **CEP104** | 0.17 | 1.68 | 0.048 | 1 x 7 | 1 p36 | centrosomal protein 104kDa [Source:HGNC Symbol;Acc:HGNC:24866] |
| **SLC5A2** | 0.17 | 1.67 | 0.049 | 1 x 7 | 16 p11 | solute carrier family 5 (sodium/glucose cotransporter), member 2 [Source:HGNC Symbol;Acc:HGNC:11037] |
| **EEFSEC** | 0.17 | 1.67 | 0.049 | 1 x 7 | 3 q21 | eukaryotic elongation factor, selenocysteine-tRNA-specific [Source:HGNC Symbol;Acc:HGNC:24614] |
| **CASD1** | 0.17 | 1.67 | 0.049 | 4 x 8 | 7 q21 | CAS1 domain containing 1 [Source:HGNC Symbol;Acc:HGNC:16014] |
| **ANKRD13A** | 0.17 | 1.67 | 0.050 | 1 x 16 | 12 q24 | ankyrin repeat domain 13A [Source:HGNC Symbol;Acc:HGNC:21268] |
| **MSANTD3** | 0.17 | 1.67 | 0.050 | 5 x 12 | 9 q31 | Myb/SANT-like DNA-binding domain containing 3 [Source:HGNC Symbol;Acc:HGNC:23370] |
| **TRDMT1** | 0.17 | 1.67 | 0.050 | 7 x 14 | 10 p13 | tRNA aspartic acid methyltransferase 1 [Source:HGNC Symbol;Acc:HGNC:2977] |
| **IMPDH2** | 0.17 | 1.66 | 0.050 | 5 x 15 | 3 p21 | IMP (inosine 5'-monophosphate) dehydrogenase 2 [Source:HGNC Symbol;Acc:HGNC:6053] |
| **ACOX1** | 0.17 | 1.64 | 0.052 | 1 x 7 | 17 q25 | acyl-CoA oxidase 1, palmitoyl [Source:HGNC Symbol;Acc:HGNC:119] |
| **SLAIN1** | 0.17 | 1.64 | 0.053 | 6 x 14 | 13 q22 | SLAIN motif family, member 1 [Source:HGNC Symbol;Acc:HGNC:26387] |
| **TMEM55A** | 0.17 | 1.64 | 0.053 | 4 x 9 | 8 q21 | transmembrane protein 55A [Source:HGNC Symbol;Acc:HGNC:25452] |
| **OSBP** | 0.17 | 1.63 | 0.054 | 1 x 7 | 11 q12 | oxysterol binding protein [Source:HGNC Symbol;Acc:HGNC:8503] |
| **SLMO1** | 0.17 | 1.62 | 0.054 | 7 x 11 | 18 p11 | slowmo homolog 1 (Drosophila) [Source:HGNC Symbol;Acc:HGNC:24639] |
| **DNAJC5** | 0.17 | 1.62 | 0.054 | 6 x 16 | 20 q13 | DnaJ (Hsp40) homolog, subfamily C, member 5 [Source:HGNC Symbol;Acc:HGNC:16235] |
| **ATAD1** | 0.17 | 1.62 | 0.055 | 6 x 9 | 10 q23 | ATPase family, AAA domain containing 1 [Source:HGNC Symbol;Acc:HGNC:25903] |
| **ERCC6L2** | 0.17 | 1.62 | 0.055 | 1 x 7 | 9 q22 | excision repair cross-complementation group 6-like 2 [Source:HGNC Symbol;Acc:HGNC:26922] |
| **ARMCX5** | 0.17 | 1.61 | 0.055 | 2 x 7 | X q22 | armadillo repeat containing, X-linked 5 [Source:HGNC Symbol;Acc:HGNC:25772] |
| **CEP83** | 0.17 | 1.61 | 0.055 | 6 x 12 | 12 q22 | centrosomal protein 83kDa [Source:HGNC Symbol;Acc:HGNC:17966] |
| **PDP2** | 0.17 | 1.61 | 0.055 | 7 x 13 | 16 q22 | pyruvate dehyrogenase phosphatase catalytic subunit 2 [Source:HGNC Symbol;Acc:HGNC:30263] |
| **PCCB** | 0.17 | 1.60 | 0.056 | 1 x 7 | 3 q22 | propionyl CoA carboxylase, beta polypeptide [Source:HGNC Symbol;Acc:HGNC:8654] |
| **KIAA0368** | 0.17 | 1.60 | 0.057 | 3 x 16 | 9 q31 | KIAA0368 [Source:HGNC Symbol;Acc:HGNC:29020] |
| **ERBB2IP** | 0.17 | 1.59 | 0.057 | 6 x 15 | 5 q12 | erbb2 interacting protein [Source:HGNC Symbol;Acc:HGNC:15842] |
| **PELI2** | 0.17 | 1.59 | 0.058 | 6 x 14 | 14 q22 | pellino E3 ubiquitin protein ligase family member 2 [Source:HGNC Symbol;Acc:HGNC:8828] |
| **AGA** | 0.16 | 1.58 | 0.059 | 1 x 7 | 4 q34 | aspartylglucosaminidase [Source:HGNC Symbol;Acc:HGNC:318] |
| **PRKAR2B** | 0.16 | 1.57 | 0.059 | 6 x 9 | 7 q22 | protein kinase, cAMP-dependent, regulatory, type II, beta [Source:HGNC Symbol;Acc:HGNC:9392] |
| **MEX3C** | 0.16 | 1.57 | 0.060 | 3 x 16 | 18 q21 | mex-3 RNA binding family member C [Source:HGNC Symbol;Acc:HGNC:28040] |
| **BTD** | 0.16 | 1.56 | 0.061 | 2 x 16 | 3 p25 | biotinidase [Source:HGNC Symbol;Acc:HGNC:1122] |
| **MFSD5** | 0.16 | 1.55 | 0.063 | 1 x 8 | 12 q13 | major facilitator superfamily domain containing 5 [Source:HGNC Symbol;Acc:HGNC:28156] |
| **CHSY1** | 0.16 | 1.54 | 0.063 | 1 x 16 | 15 q26 | chondroitin sulfate synthase 1 [Source:HGNC Symbol;Acc:HGNC:17198] |
| **TARS2** | 0.16 | 1.54 | 0.064 | 5 x 10 | 1 q21 | threonyl-tRNA synthetase 2, mitochondrial (putative) [Source:HGNC Symbol;Acc:HGNC:30740] |
| **MTA3** | 0.16 | 1.54 | 0.064 | 1 x 8 | 2 p21 | metastasis associated 1 family, member 3 [Source:HGNC Symbol;Acc:HGNC:23784] |
| **MAPK1** | 0.16 | 1.52 | 0.065 | 6 x 9 | 22 q11 | mitogen-activated protein kinase 1 [Source:HGNC Symbol;Acc:HGNC:6871] |
| **ZNF721** | 0.16 | 1.52 | 0.066 | 5 x 15 | 4 p16 | zinc finger protein 721 [Source:HGNC Symbol;Acc:HGNC:29425] |
| **TOP1** | 0.16 | 1.52 | 0.066 | 7 x 14 | 20 q12 | topoisomerase (DNA) I [Source:HGNC Symbol;Acc:HGNC:11986] |
| **ST20** | 0.16 | 1.52 | 0.066 | 1 x 8 | 15 q25 | suppressor of tumorigenicity 20 [Source:HGNC Symbol;Acc:HGNC:33520] |
| **MTHFS** | 0.16 | 1.52 | 0.066 | 1 x 16 | 15 q25 | 5,10-methenyltetrahydrofolate synthetase (5-formyltetrahydrofolate cyclo-ligase) [Source:HGNC Symbol;Acc:HGNC:7437] |
| **BZW2** | 0.16 | 1.51 | 0.067 | 2 x 16 | 7 p21 | basic leucine zipper and W2 domains 2 [Source:HGNC Symbol;Acc:HGNC:18808] |
| **ZNF121** | 0.16 | 1.51 | 0.068 | 7 x 12 | 19 p13 | zinc finger protein 121 [Source:HGNC Symbol;Acc:HGNC:12904] |
| **GEMIN8** | 0.16 | 1.50 | 0.069 | 1 x 7 | X p22 | gem (nuclear organelle) associated protein 8 [Source:HGNC Symbol;Acc:HGNC:26044] |
| **HHAT** | 0.16 | 1.50 | 0.069 | 6 x 14 | 1 q32 | hedgehog acyltransferase [Source:HGNC Symbol;Acc:HGNC:18270] |
| **GOSR2** | 0.16 | 1.50 | 0.069 | 5 x 15 | 17 q21 | golgi SNAP receptor complex member 2 [Source:HGNC Symbol;Acc:HGNC:4431] |
| **ENTPD4** | 0.16 | 1.49 | 0.070 | 1 x 15 | 8 p21 | ectonucleoside triphosphate diphosphohydrolase 4 [Source:HGNC Symbol;Acc:HGNC:14573] |
| **SRRD** | 0.15 | 1.47 | 0.073 | 5 x 9 | 22 q12 | SRR1 domain containing [Source:HGNC Symbol;Acc:HGNC:33910] |
| **RNF40** | 0.15 | 1.47 | 0.073 | 1 x 7 | 16 p11 | ring finger protein 40, E3 ubiquitin protein ligase [Source:HGNC Symbol;Acc:HGNC:16867] |
| **WDR13** | 0.15 | 1.46 | 0.073 | 1 x 8 | X p11 | WD repeat domain 13 [Source:HGNC Symbol;Acc:HGNC:14352] |
| **RNMTL1** | 0.15 | 1.45 | 0.075 | 5 x 16 | 17 p13 | RNA methyltransferase like 1 [Source:HGNC Symbol;Acc:HGNC:18485] |
| **ZNF589** | 0.15 | 1.45 | 0.075 | 1 x 14 | 3 p21 | zinc finger protein 589 [Source:HGNC Symbol;Acc:HGNC:16747] |
| **FBXO45** | 0.15 | 1.45 | 0.076 | 5 x 14 | 3 q29 | F-box protein 45 [Source:HGNC Symbol;Acc:HGNC:29148] |
| **GTPBP4** | 0.15 | 1.42 | 0.080 | 7 x 12 | 10 p15 | GTP binding protein 4 [Source:HGNC Symbol;Acc:HGNC:21535] |
| **MBD3** | 0.15 | 1.40 | 0.083 | 7 x 13 | 19 p13 | methyl-CpG binding domain protein 3 [Source:HGNC Symbol;Acc:HGNC:6918] |
| **PDGFD** | 0.15 | 1.40 | 0.083 | 1 x 7 | 11 q22 | platelet derived growth factor D [Source:HGNC Symbol;Acc:HGNC:30620] |
| **FANCF** | 0.15 | 1.39 | 0.084 | 6 x 16 | 11 p14 | Fanconi anemia, complementation group F [Source:HGNC Symbol;Acc:HGNC:3587] |
| **DUSP7** | 0.14 | 1.37 | 0.087 | 7 x 12 | 3 p21 | dual specificity phosphatase 7 [Source:HGNC Symbol;Acc:HGNC:3073] |
| **UBAP2** | 0.14 | 1.37 | 0.088 | 4 x 16 | 9 p13 | ubiquitin associated protein 2 [Source:HGNC Symbol;Acc:HGNC:14185] |
| **HLA-DPB1** | 0.14 | 1.36 | 0.088 | 7 x 13 | NA | major histocompatibility complex, class II, DP beta 1 [Source:HGNC Symbol;Acc:HGNC:4940] |
| **RUVBL2** | 0.14 | 1.36 | 0.089 | 6 x 13 | 19 q13 | RuvB-like AAA ATPase 2 [Source:HGNC Symbol;Acc:HGNC:10475] |
| **CPS1** | 0.14 | 1.36 | 0.089 | 5 x 15 | 2 q34 | carbamoyl-phosphate synthase 1, mitochondrial [Source:HGNC Symbol;Acc:HGNC:2323] |
| **FUNDC1** | 0.14 | 1.35 | 0.090 | 1 x 7 | X p11 | FUN14 domain containing 1 [Source:HGNC Symbol;Acc:HGNC:28746] |
| **SPECC1L** | 0.14 | 1.35 | 0.090 | 5 x 10 | 22 q11 | sperm antigen with calponin homology and coiled-coil domains 1-like [Source:HGNC Symbol;Acc:HGNC:29022] |
| **C15orf40** | 0.14 | 1.30 | 0.098 | 2 x 15 | 15 q25 | chromosome 15 open reading frame 40 [Source:HGNC Symbol;Acc:HGNC:28443] |
| **GRWD1** | 0.13 | 1.28 | 0.101 | 7 x 14 | 19 q13 | glutamate-rich WD repeat containing 1 [Source:HGNC Symbol;Acc:HGNC:21270] |
| **SPESP1** | 0.13 | 1.27 | 0.103 | 2 x 16 | 15 q23 | sperm equatorial segment protein 1 [Source:HGNC Symbol;Acc:HGNC:15570] |
| **PNO1** | 0.13 | 1.25 | 0.107 | 5 x 16 | 2 p14 | partner of NOB1 homolog (S. cerevisiae) [Source:HGNC Symbol;Acc:HGNC:32790] |
| **SMAD7** | 0.13 | 1.23 | 0.112 | 6 x 15 | 18 q21 | SMAD family member 7 [Source:HGNC Symbol;Acc:HGNC:6773] |
| **PPP2R3A** | 0.13 | 1.22 | 0.112 | 7 x 13 | 3 q22 | protein phosphatase 2, regulatory subunit B'', alpha [Source:HGNC Symbol;Acc:HGNC:9307] |
| **PGP** | 0.13 | 1.21 | 0.115 | 7 x 14 | 16 p13 | phosphoglycolate phosphatase [Source:HGNC Symbol;Acc:HGNC:8909] |
| **NFE2L1** | 0.12 | 1.19 | 0.118 | 1 x 7 | 17 q21 | nuclear factor, erythroid 2-like 1 [Source:HGNC Symbol;Acc:HGNC:7781] |
| **NEK4** | 0.12 | 1.15 | 0.126 | 7 x 11 | 3 p21 | NIMA-related kinase 4 [Source:HGNC Symbol;Acc:HGNC:11399] |
| **ERCC8** | 0.12 | 1.12 | 0.134 | 1 x 15 | 5 q12 | excision repair cross-complementation group 8 [Source:HGNC Symbol;Acc:HGNC:3439] |
| **FAM162A** | 0.11 | 1.08 | 0.142 | 1 x 7 | 3 q21 | family with sequence similarity 162, member A [Source:HGNC Symbol;Acc:HGNC:17865] |
| **SEPHS2** | 0.11 | 1.05 | 0.149 | 2 x 16 | 16 p11 | selenophosphate synthetase 2 [Source:HGNC Symbol;Acc:HGNC:19686] |
| **PTPN11** | 0.11 | 1.01 | 0.158 | 5 x 16 | 12 q24 | protein tyrosine phosphatase, non-receptor type 11 [Source:HGNC Symbol;Acc:HGNC:9644] |
| **HECTD3** | 0.10 | 0.99 | 0.163 | 7 x 13 | 1 p34 | HECT domain containing E3 ubiquitin protein ligase 3 [Source:HGNC Symbol;Acc:HGNC:26117] |
| **IL6ST** | 0.10 | 0.94 | 0.175 | 5 x 16 | 5 q11 | interleukin 6 signal transducer [Source:HGNC Symbol;Acc:HGNC:6021] |
| **BRWD1** | 0.09 | 0.88 | 0.190 | 7 x 14 | 21 q22 | bromodomain and WD repeat domain containing 1 [Source:HGNC Symbol;Acc:HGNC:12760] |
| **TTC7B** | 0.07 | 0.65 | 0.258 | 3 x 16 | 14 q32 | tetratricopeptide repeat domain 7B [Source:HGNC Symbol;Acc:HGNC:19858] |
| **GADD45B** | 0.06 | 0.56 | 0.288 | 6 x 15 | 19 p13 | growth arrest and DNA-damage-inducible, beta [Source:HGNC Symbol;Acc:HGNC:4096] |
| **ZNF43** | 0.06 | 0.55 | 0.290 | 3 x 16 | NA | zinc finger protein 43 [Source:HGNC Symbol;Acc:HGNC:13109] |

| **Genes of spot D** | | | | | | | | |
| --- | --- | --- | --- | --- | --- | --- | --- | --- |
| **Symbol** | **Correlation ^1^** | | **->t.score** | **->p.value** | | **Metagene ^2^** | **Chromosome** | **Description** |
| **CRIM1** | | 0.82 | 13.59 | 0 | 50 x 1 | | NA | cysteine rich transmembrane BMP regulator 1 (chordin-like) [Source:HGNC Symbol;Acc:HGNC:2359] |
| **ADAMTS5** | | 0.79 | 12.23 | 0 | 45 x 3 | | 21 q21 | ADAM metallopeptidase with thrombospondin type 1 motif, 5 [Source:HGNC Symbol;Acc:HGNC:221] |
| **MYL12B** | | 0.79 | 12.21 | 0 | 48 x 4 | | 18 p11 | myosin, light chain 12B, regulatory [Source:HGNC Symbol;Acc:HGNC:29827] |
| **THBS1** | | 0.78 | 11.94 | 0 | 50 x 1 | | 15 q14 | thrombospondin 1 [Source:HGNC Symbol;Acc:HGNC:11785] |
| **ANXA1** | | 0.77 | 11.41 | 0 | 50 x 1 | | 9 q21 | annexin A1 [Source:HGNC Symbol;Acc:HGNC:533] |
| **NAV3** | | 0.74 | 10.57 | 0 | 50 x 1 | | 12 q21 | neuron navigator 3 [Source:HGNC Symbol;Acc:HGNC:15998] |
| **PZP** | | 0.74 | 10.44 | 0 | 50 x 2 | | 12 p13 | pregnancy-zone protein [Source:HGNC Symbol;Acc:HGNC:9750] |
| **NTN4** | | 0.74 | 10.42 | 0 | 46 x 2 | | 12 q22 | netrin 4 [Source:HGNC Symbol;Acc:HGNC:13658] |
| **FRMD6** | | 0.74 | 10.33 | 0 | 50 x 1 | | 14 q22 | FERM domain containing 6 [Source:HGNC Symbol;Acc:HGNC:19839] |
| **ITGA1** | | 0.72 | 9.91 | 2.E-16 | 50 x 1 | | 5 q11 | integrin, alpha 1 [Source:HGNC Symbol;Acc:HGNC:6134] |
| **PRKG1** | | 0.71 | 9.69 | 7.E-16 | 47 x 4 | | 10 q11 | protein kinase, cGMP-dependent, type I [Source:HGNC Symbol;Acc:HGNC:9414] |
| **ADAM19** | | 0.71 | 9.66 | 7.E-16 | 45 x 2 | | 5 q33 | ADAM metallopeptidase domain 19 [Source:HGNC Symbol;Acc:HGNC:197] |
| **MYOF** | | 0.71 | 9.66 | 7.E-16 | 50 x 1 | | 10 q23 | myoferlin [Source:HGNC Symbol;Acc:HGNC:3656] |
| **MYL12A** | | 0.71 | 9.57 | 1.E-15 | 48 x 3 | | 18 p11 | myosin, light chain 12A, regulatory, non-sarcomeric [Source:HGNC Symbol;Acc:HGNC:16701] |
| **PRELP** | | 0.70 | 9.26 | 5.E-15 | 50 x 1 | | 1 q32 | proline/arginine-rich end leucine-rich repeat protein [Source:HGNC Symbol;Acc:HGNC:9357] |
| **CALD1** | | 0.70 | 9.26 | 5.E-15 | 50 x 1 | | 7 q33 | caldesmon 1 [Source:HGNC Symbol;Acc:HGNC:1441] |
| **KCNJ2** | | 0.69 | 9.13 | 9.E-15 | 48 x 1 | | 17 q24 | potassium channel, inwardly rectifying subfamily J, member 2 [Source:HGNC Symbol;Acc:HGNC:6263] |
| **CDK6** | | 0.69 | 9.11 | 1.E-14 | 49 x 1 | | 7 q21 | cyclin-dependent kinase 6 [Source:HGNC Symbol;Acc:HGNC:1777] |
| **C10orf10** | | 0.69 | 9.07 | 1.E-14 | 49 x 1 | | 10 q11 | chromosome 10 open reading frame 10 [Source:HGNC Symbol;Acc:HGNC:23355] |
| **PTPRZ1** | | 0.69 | 8.98 | 2.E-14 | 50 x 1 | | 7 q31 | protein tyrosine phosphatase, receptor-type, Z polypeptide 1 [Source:HGNC Symbol;Acc:HGNC:9685] |
| **F2R** | | 0.69 | 8.95 | 2.E-14 | 50 x 1 | | 5 q13 | coagulation factor II (thrombin) receptor [Source:HGNC Symbol;Acc:HGNC:3537] |
| **EDIL3** | | 0.68 | 8.87 | 3.E-14 | 50 x 1 | | 5 q14 | EGF-like repeats and discoidin I-like domains 3 [Source:HGNC Symbol;Acc:HGNC:3173] |
| **HDAC9** | | 0.68 | 8.82 | 4.E-14 | 50 x 1 | | 7 p21 | histone deacetylase 9 [Source:HGNC Symbol;Acc:HGNC:14065] |
| **SFRP1** | | 0.68 | 8.80 | 5.E-14 | 50 x 1 | | 8 p11 | secreted frizzled-related protein 1 [Source:HGNC Symbol;Acc:HGNC:10776] |
| **SAMD4A** | | 0.68 | 8.75 | 6.E-14 | 49 x 1 | | 14 q22 | sterile alpha motif domain containing 4A [Source:HGNC Symbol;Acc:HGNC:23023] |
| **DSEL** | | 0.68 | 8.71 | 7.E-14 | 46 x 5 | | NA | dermatan sulfate epimerase-like [Source:HGNC Symbol;Acc:HGNC:18144] |
| **TMSB10** | | 0.66 | 8.44 | 2.E-13 | 49 x 5 | | 2 p11 | thymosin beta 10 [Source:HGNC Symbol;Acc:HGNC:11879] |
| **HSPB2** | | 0.66 | 8.43 | 3.E-13 | 50 x 1 | | 11 q23 | heat shock 27kDa protein 2 [Source:HGNC Symbol;Acc:HGNC:5247] |
| **ANXA2** | | 0.66 | 8.38 | 3.E-13 | 50 x 3 | | 15 q22 | annexin A2 [Source:HGNC Symbol;Acc:HGNC:537] |
| **PLSCR4** | | 0.66 | 8.35 | 4.E-13 | 49 x 1 | | 3 q24 | phospholipid scramblase 4 [Source:HGNC Symbol;Acc:HGNC:16497] |
| **DSTN** | | 0.66 | 8.32 | 4.E-13 | 48 x 3 | | 20 p12 | destrin (actin depolymerizing factor) [Source:HGNC Symbol;Acc:HGNC:15750] |
| **FAT4** | | 0.66 | 8.26 | 6.E-13 | 46 x 6 | | 4 q28 | FAT atypical cadherin 4 [Source:HGNC Symbol;Acc:HGNC:23109] |
| **GDNF** | | 0.65 | 8.08 | 1.E-12 | 50 x 3 | | 5 p13 | glial cell derived neurotrophic factor [Source:HGNC Symbol;Acc:HGNC:4232] |
| **VTN** | | 0.65 | 8.04 | 2.E-12 | 50 x 1 | | 17 q11 | vitronectin [Source:HGNC Symbol;Acc:HGNC:12724] |
| **CTNNA1** | | 0.64 | 7.97 | 2.E-12 | 46 x 4 | | 5 q31 | catenin (cadherin-associated protein), alpha 1, 102kDa [Source:HGNC Symbol;Acc:HGNC:2509] |
| **TMEM45A** | | 0.64 | 7.95 | 3.E-12 | 50 x 1 | | 3 q12 | transmembrane protein 45A [Source:HGNC Symbol;Acc:HGNC:25480] |
| **PKD2** | | 0.64 | 7.85 | 4.E-12 | 50 x 1 | | 4 q22 | polycystic kidney disease 2 (autosomal dominant) [Source:HGNC Symbol;Acc:HGNC:9009] |
| **EPAS1** | | 0.63 | 7.75 | 7.E-12 | 50 x 2 | | 2 p21 | endothelial PAS domain protein 1 [Source:HGNC Symbol;Acc:HGNC:3374] |
| **CRYAB** | | 0.63 | 7.64 | 1.E-11 | 50 x 1 | | 11 q23 | crystallin, alpha B [Source:HGNC Symbol;Acc:HGNC:2389] |
| **AHNAK** | | 0.63 | 7.63 | 1.E-11 | 50 x 1 | | 11 q12 | AHNAK nucleoprotein [Source:HGNC Symbol;Acc:HGNC:347] |
| **NDRG1** | | 0.63 | 7.61 | 1.E-11 | 49 x 5 | | 8 q24 | N-myc downstream regulated 1 [Source:HGNC Symbol;Acc:HGNC:7679] |
| **OLR1** | | 0.62 | 7.57 | 2.E-11 | 46 x 2 | | 12 p13 | oxidized low density lipoprotein (lectin-like) receptor 1 [Source:HGNC Symbol;Acc:HGNC:8133] |
| **HSPB3** | | 0.62 | 7.54 | 2.E-11 | 48 x 5 | | 5 q11 | heat shock 27kDa protein 3 [Source:HGNC Symbol;Acc:HGNC:5248] |
| **LIMCH1** | | 0.62 | 7.44 | 3.E-11 | 50 x 1 | | 4 p13 | LIM and calponin homology domains 1 [Source:HGNC Symbol;Acc:HGNC:29191] |
| **DKK1** | | 0.62 | 7.43 | 3.E-11 | 50 x 1 | | 10 q21 | dickkopf WNT signaling pathway inhibitor 1 [Source:HGNC Symbol;Acc:HGNC:2891] |
| **HSPB2-C11orf52** | | 0.61 | 7.34 | 5.E-11 | 50 x 1 | | 11 q23 | HSPB2-C11orf52 readthrough (NMD candidate) [Source:HGNC Symbol;Acc:HGNC:41996] |
| **CDKN2B** | | 0.61 | 7.33 | 5.E-11 | 46 x 4 | | 9 p21 | cyclin-dependent kinase inhibitor 2B (p15, inhibits CDK4) [Source:HGNC Symbol;Acc:HGNC:1788] |
| **DNAJC6** | | 0.61 | 7.32 | 5.E-11 | 50 x 1 | | 1 p31 | DnaJ (Hsp40) homolog, subfamily C, member 6 [Source:HGNC Symbol;Acc:HGNC:15469] |
| **SLC5A3** | | 0.61 | 7.28 | 6.E-11 | 50 x 2 | | 21 q22 | solute carrier family 5 (sodium/myo-inositol cotransporter), member 3 [Source:HGNC Symbol;Acc:HGNC:11038] |
| **SPOCK1** | | 0.61 | 7.22 | 8.E-11 | 50 x 1 | | 5 q31 | sparc/osteonectin, cwcv and kazal-like domains proteoglycan (testican) 1 [Source:HGNC Symbol;Acc:HGNC:11251] |
| **NES** | | 0.60 | 7.20 | 9.E-11 | 45 x 1 | | 1 q23 | nestin [Source:HGNC Symbol;Acc:HGNC:7756] |
| **FAM65B** | | 0.60 | 7.20 | 9.E-11 | 46 x 6 | | 6 p22 | family with sequence similarity 65, member B [Source:HGNC Symbol;Acc:HGNC:13872] |
| **MYOZ2** | | 0.60 | 7.14 | 1.E-10 | 48 x 1 | | 4 q26 | myozenin 2 [Source:HGNC Symbol;Acc:HGNC:1330] |
| **SLIT2** | | 0.60 | 7.09 | 1.E-10 | 50 x 1 | | 4 p15 | slit homolog 2 (Drosophila) [Source:HGNC Symbol;Acc:HGNC:11086] |
| **S100A16** | | 0.60 | 7.07 | 2.E-10 | 50 x 3 | | 1 q21 | S100 calcium binding protein A16 [Source:HGNC Symbol;Acc:HGNC:20441] |
| **MAP2** | | 0.60 | 7.03 | 2.E-10 | 50 x 2 | | 2 q34 | microtubule-associated protein 2 [Source:HGNC Symbol;Acc:HGNC:6839] |
| **SYNJ2** | | 0.60 | 7.03 | 2.E-10 | 48 x 3 | | 6 q25 | synaptojanin 2 [Source:HGNC Symbol;Acc:HGNC:11504] |
| **FTL** | | 0.59 | 7.01 | 2.E-10 | 48 x 6 | | 19 q13 | ferritin, light polypeptide [Source:HGNC Symbol;Acc:HGNC:3999] |
| **RHOBTB3** | | 0.59 | 6.99 | 2.E-10 | 50 x 1 | | 5 q15 | Rho-related BTB domain containing 3 [Source:HGNC Symbol;Acc:HGNC:18757] |
| **CITED2** | | 0.59 | 6.99 | 2.E-10 | 44 x 1 | | 6 q24 | Cbp/p300-interacting transactivator, with Glu/Asp-rich carboxy-terminal domain, 2 [Source:HGNC Symbol;Acc:HGNC:1987] |
| **AMOTL2** | | 0.59 | 6.97 | 3.E-10 | 49 x 1 | | 3 q22 | angiomotin like 2 [Source:HGNC Symbol;Acc:HGNC:17812] |
| **L1CAM** | | 0.59 | 6.96 | 3.E-10 | 46 x 4 | | X q28 | L1 cell adhesion molecule [Source:HGNC Symbol;Acc:HGNC:6470] |
| **KLF9** | | 0.59 | 6.95 | 3.E-10 | 50 x 1 | | 9 q21 | Kruppel-like factor 9 [Source:HGNC Symbol;Acc:HGNC:1123] |
| **S100A4** | | 0.59 | 6.95 | 3.E-10 | 50 x 1 | | 1 q21 | S100 calcium binding protein A4 [Source:HGNC Symbol;Acc:HGNC:10494] |
| **FRY** | | 0.59 | 6.93 | 3.E-10 | 46 x 3 | | 13 q13 | furry homolog (Drosophila) [Source:HGNC Symbol;Acc:HGNC:20367] |
| **USP53** | | 0.59 | 6.88 | 4.E-10 | 48 x 1 | | 4 q26 | ubiquitin specific peptidase 53 [Source:HGNC Symbol;Acc:HGNC:29255] |
| **MYL6** | | 0.58 | 6.84 | 5.E-10 | 50 x 6 | | 12 q13 | myosin, light chain 6, alkali, smooth muscle and non-muscle [Source:HGNC Symbol;Acc:HGNC:7587] |
| **SAMD5** | | 0.58 | 6.77 | 6.E-10 | 47 x 1 | | 6 q24 | sterile alpha motif domain containing 5 [Source:HGNC Symbol;Acc:HGNC:21180] |
| **CNN3** | | 0.58 | 6.76 | 7.E-10 | 47 x 1 | | 1 p21 | calponin 3, acidic [Source:HGNC Symbol;Acc:HGNC:2157] |
| **IGFBP2** | | 0.58 | 6.75 | 7.E-10 | 50 x 3 | | 2 q35 | insulin-like growth factor binding protein 2, 36kDa [Source:HGNC Symbol;Acc:HGNC:5471] |
| **FOSL2** | | 0.58 | 6.72 | 8.E-10 | 46 x 4 | | 2 p23 | FOS-like antigen 2 [Source:HGNC Symbol;Acc:HGNC:3798] |
| **PNRC1** | | 0.58 | 6.70 | 9.E-10 | 50 x 1 | | 6 q15 | proline-rich nuclear receptor coactivator 1 [Source:HGNC Symbol;Acc:HGNC:17278] |
| **CTHRC1** | | 0.58 | 6.69 | 9.E-10 | 49 x 1 | | 8 q22 | collagen triple helix repeat containing 1 [Source:HGNC Symbol;Acc:HGNC:18831] |
| **GFRA1** | | 0.57 | 6.66 | 1.E-09 | 44 x 2 | | 10 q25 | GDNF family receptor alpha 1 [Source:HGNC Symbol;Acc:HGNC:4243] |
| **ALCAM** | | 0.57 | 6.66 | 1.E-09 | 50 x 1 | | 3 q13 | activated leukocyte cell adhesion molecule [Source:HGNC Symbol;Acc:HGNC:400] |
| **TFPI** | | 0.57 | 6.64 | 1.E-09 | 50 x 1 | | 2 q32 | tissue factor pathway inhibitor (lipoprotein-associated coagulation inhibitor) [Source:HGNC Symbol;Acc:HGNC:11760] |
| **ARID5B** | | 0.57 | 6.60 | 1.E-09 | 50 x 1 | | 10 q21 | AT rich interactive domain 5B (MRF1-like) [Source:HGNC Symbol;Acc:HGNC:17362] |
| **HSPB7** | | 0.57 | 6.60 | 1.E-09 | 46 x 5 | | 1 p36 | heat shock 27kDa protein family, member 7 (cardiovascular) [Source:HGNC Symbol;Acc:HGNC:5249] |
| **HIST1H2BC** | | 0.57 | 6.54 | 2.E-09 | 50 x 1 | | 6 p22 | histone cluster 1, H2bc [Source:HGNC Symbol;Acc:HGNC:4757] |
| **BDNF** | | 0.57 | 6.52 | 2.E-09 | 46 x 7 | | 11 p14 | brain-derived neurotrophic factor [Source:HGNC Symbol;Acc:HGNC:1033] |
| **CYTH3** | | 0.57 | 6.50 | 2.E-09 | 46 x 5 | | 7 p22 | cytohesin 3 [Source:HGNC Symbol;Acc:HGNC:9504] |
| **AMT** | | 0.56 | 6.46 | 3.E-09 | 45 x 1 | | 3 p21 | aminomethyltransferase [Source:HGNC Symbol;Acc:HGNC:473] |
| **TXNIP** | | 0.56 | 6.44 | 3.E-09 | 50 x 1 | | 1 q21 | thioredoxin interacting protein [Source:HGNC Symbol;Acc:HGNC:16952] |
| **SPTA1** | | 0.55 | 6.19 | 9.E-09 | 50 x 1 | | 1 q23 | spectrin, alpha, erythrocytic 1 [Source:HGNC Symbol;Acc:HGNC:11272] |
| **TNFRSF12A** | | 0.55 | 6.18 | 9.E-09 | 50 x 1 | | 16 p13 | tumor necrosis factor receptor superfamily, member 12A [Source:HGNC Symbol;Acc:HGNC:18152] |
| **ATF3** | | 0.54 | 6.17 | 1.E-08 | 46 x 1 | | 1 q32 | activating transcription factor 3 [Source:HGNC Symbol;Acc:HGNC:785] |
| **BCL6** | | 0.54 | 6.16 | 1.E-08 | 50 x 1 | | 3 q27 | B-cell CLL/lymphoma 6 [Source:HGNC Symbol;Acc:HGNC:1001] |
| **RDH10** | | 0.54 | 6.14 | 1.E-08 | 48 x 1 | | 8 q21 | retinol dehydrogenase 10 (all-trans) [Source:HGNC Symbol;Acc:HGNC:19975] |
| **MARCKS** | | 0.54 | 6.11 | 1.E-08 | 46 x 1 | | 6 q21 | myristoylated alanine-rich protein kinase C substrate [Source:HGNC Symbol;Acc:HGNC:6759] |
| **SLC12A8** | | 0.54 | 6.09 | 1.E-08 | 50 x 4 | | 3 q21 | solute carrier family 12, member 8 [Source:HGNC Symbol;Acc:HGNC:15595] |
| **MBNL2** | | 0.54 | 6.08 | 1.E-08 | 50 x 1 | | 13 q32 | muscleblind-like splicing regulator 2 [Source:HGNC Symbol;Acc:HGNC:16746] |
| **KLHL30** | | 0.54 | 6.04 | 2.E-08 | 44 x 4 | | 2 q37 | kelch-like family member 30 [Source:HGNC Symbol;Acc:HGNC:24770] |
| **PTPRM** | | 0.54 | 6.02 | 2.E-08 | 50 x 1 | | 18 p11 | protein tyrosine phosphatase, receptor type, M [Source:HGNC Symbol;Acc:HGNC:9675] |
| **CREB5** | | 0.53 | 5.98 | 2.E-08 | 49 x 1 | | 7 p15 | cAMP responsive element binding protein 5 [Source:HGNC Symbol;Acc:HGNC:16844] |
| **HBEGF** | | 0.53 | 5.98 | 2.E-08 | 48 x 1 | | 5 q31 | heparin-binding EGF-like growth factor [Source:HGNC Symbol;Acc:HGNC:3059] |
| **ITGB8** | | 0.53 | 5.90 | 3.E-08 | 47 x 4 | | 7 p21 | integrin, beta 8 [Source:HGNC Symbol;Acc:HGNC:6163] |
| **DRAM1** | | 0.53 | 5.89 | 3.E-08 | 50 x 2 | | 12 q23 | DNA-damage regulated autophagy modulator 1 [Source:HGNC Symbol;Acc:HGNC:25645] |
| **IL24** | | 0.53 | 5.88 | 3.E-08 | 50 x 3 | | 1 q32 | interleukin 24 [Source:HGNC Symbol;Acc:HGNC:11346] |
| **TPM4** | | 0.53 | 5.85 | 4.E-08 | 50 x 1 | | 19 p13 | tropomyosin 4 [Source:HGNC Symbol;Acc:HGNC:12013] |
| **GXYLT2** | | 0.52 | 5.82 | 4.E-08 | 49 x 6 | | 3 p13 | glucoside xylosyltransferase 2 [Source:HGNC Symbol;Acc:HGNC:33383] |
| **MATN2** | | 0.52 | 5.78 | 5.E-08 | 50 x 1 | | 8 q22 | matrilin 2 [Source:HGNC Symbol;Acc:HGNC:6908] |
| **TAGLN** | | 0.52 | 5.72 | 7.E-08 | 50 x 6 | | 11 q23 | transgelin [Source:HGNC Symbol;Acc:HGNC:11553] |
| **FADS3** | | 0.52 | 5.71 | 7.E-08 | 50 x 4 | | 11 q12 | fatty acid desaturase 3 [Source:HGNC Symbol;Acc:HGNC:3576] |
| **HIST1H2AC** | | 0.51 | 5.69 | 8.E-08 | 50 x 1 | | 6 p22 | histone cluster 1, H2ac [Source:HGNC Symbol;Acc:HGNC:4733] |
| **DLC1** | | 0.51 | 5.68 | 8.E-08 | 49 x 1 | | 8 p22 | DLC1 Rho GTPase activating protein [Source:HGNC Symbol;Acc:HGNC:2897] |
| **OPN3** | | 0.51 | 5.66 | 9.E-08 | 46 x 1 | | 1 q43 | opsin 3 [Source:HGNC Symbol;Acc:HGNC:14007] |
| **NT5E** | | 0.51 | 5.66 | 9.E-08 | 50 x 1 | | 6 q14 | 5'-nucleotidase, ecto (CD73) [Source:HGNC Symbol;Acc:HGNC:8021] |
| **TIMP2** | | 0.51 | 5.65 | 9.E-08 | 50 x 4 | | 17 q25 | TIMP metallopeptidase inhibitor 2 [Source:HGNC Symbol;Acc:HGNC:11821] |
| **PLXNB2** | | 0.51 | 5.64 | 1.E-07 | 50 x 1 | | 22 q13 | plexin B2 [Source:HGNC Symbol;Acc:HGNC:9104] |
| **MRPS6** | | 0.51 | 5.62 | 1.E-07 | 50 x 3 | | 21 q22 | mitochondrial ribosomal protein S6 [Source:HGNC Symbol;Acc:HGNC:14051] |
| **C15orf52** | | 0.51 | 5.61 | 1.E-07 | 47 x 1 | | 15 q15 | chromosome 15 open reading frame 52 [Source:HGNC Symbol;Acc:HGNC:33488] |
| **CDKN1A** | | 0.51 | 5.61 | 1.E-07 | 50 x 1 | | 6 p21 | cyclin-dependent kinase inhibitor 1A (p21, Cip1) [Source:HGNC Symbol;Acc:HGNC:1784] |
| **ACOT12** | | 0.51 | 5.56 | 1.E-07 | 46 x 4 | | 5 q14 | acyl-CoA thioesterase 12 [Source:HGNC Symbol;Acc:HGNC:24436] |
| **SLC8A1** | | 0.50 | 5.51 | 2.E-07 | 50 x 7 | | 2 p22 | solute carrier family 8 (sodium/calcium exchanger), member 1 [Source:HGNC Symbol;Acc:HGNC:11068] |
| **TNFSF9** | | 0.50 | 5.43 | 2.E-07 | 47 x 4 | | 19 p13 | tumor necrosis factor (ligand) superfamily, member 9 [Source:HGNC Symbol;Acc:HGNC:11939] |
| **CLIP3** | | 0.50 | 5.42 | 2.E-07 | 47 x 1 | | 19 q13 | CAP-GLY domain containing linker protein 3 [Source:HGNC Symbol;Acc:HGNC:24314] |
| **ACCS** | | 0.50 | 5.42 | 2.E-07 | 44 x 4 | | 11 p11 | 1-aminocyclopropane-1-carboxylate synthase homolog (Arabidopsis)(non-functional) [Source:HGNC Symbol;Acc:HGNC:23989] |
| **A2M** | | 0.50 | 5.40 | 3.E-07 | 50 x 1 | | 12 p13 | alpha-2-macroglobulin [Source:HGNC Symbol;Acc:HGNC:7] |
| **KLF5** | | 0.49 | 5.39 | 3.E-07 | 46 x 4 | | 13 q22 | Kruppel-like factor 5 (intestinal) [Source:HGNC Symbol;Acc:HGNC:6349] |
| **MAP1B** | | 0.49 | 5.39 | 3.E-07 | 50 x 2 | | 5 q13 | microtubule-associated protein 1B [Source:HGNC Symbol;Acc:HGNC:6836] |
| **ARRDC3** | | 0.49 | 5.34 | 3.E-07 | 50 x 1 | | 5 q14 | arrestin domain containing 3 [Source:HGNC Symbol;Acc:HGNC:29263] |
| **HLA-B** | | 0.49 | 5.32 | 4.E-07 | 44 x 5 | | NA | major histocompatibility complex, class I, B [Source:HGNC Symbol;Acc:HGNC:4932] |
| **LMO7** | | 0.49 | 5.31 | 4.E-07 | 50 x 2 | | 13 q22 | LIM domain 7 [Source:HGNC Symbol;Acc:HGNC:6646] |
| **ELL2** | | 0.49 | 5.29 | 4.E-07 | 50 x 1 | | 5 q15 | elongation factor, RNA polymerase II, 2 [Source:HGNC Symbol;Acc:HGNC:17064] |
| **PBX1** | | 0.49 | 5.28 | 5.E-07 | 46 x 6 | | 1 q23 | pre-B-cell leukemia homeobox 1 [Source:HGNC Symbol;Acc:HGNC:8632] |
| **KRTAP7-1** | | 0.48 | 5.26 | 5.E-07 | 50 x 1 | | 21 q22 | keratin associated protein 7-1 (gene/pseudogene) [Source:HGNC Symbol;Acc:HGNC:18934] |
| **PLEKHG3** | | 0.48 | 5.25 | 5.E-07 | 46 x 1 | | 14 q23 | pleckstrin homology domain containing, family G (with RhoGef domain) member 3 [Source:HGNC Symbol;Acc:HGNC:20364] |
| **FGF7** | | 0.48 | 5.23 | 5.E-07 | 50 x 6 | | 15 q21 | fibroblast growth factor 7 [Source:HGNC Symbol;Acc:HGNC:3685] |
| **APBB2** | | 0.48 | 5.23 | 5.E-07 | 47 x 1 | | 4 p13 | amyloid beta (A4) precursor protein-binding, family B, member 2 [Source:HGNC Symbol;Acc:HGNC:582] |
| **AJUBA** | | 0.48 | 5.22 | 6.E-07 | 46 x 1 | | 14 q11 | ajuba LIM protein [Source:HGNC Symbol;Acc:HGNC:20250] |
| **PRICKLE2** | | 0.48 | 5.20 | 6.E-07 | 44 x 3 | | 3 p14 | prickle homolog 2 (Drosophila) [Source:HGNC Symbol;Acc:HGNC:20340] |
| **PCDH20** | | 0.48 | 5.18 | 7.E-07 | 46 x 1 | | 13 q21 | protocadherin 20 [Source:HGNC Symbol;Acc:HGNC:14257] |
| **C1orf198** | | 0.48 | 5.18 | 7.E-07 | 49 x 1 | | 1 q42 | chromosome 1 open reading frame 198 [Source:HGNC Symbol;Acc:HGNC:25900] |
| **MLLT3** | | 0.48 | 5.15 | 7.E-07 | 50 x 4 | | 9 p21 | myeloid/lymphoid or mixed-lineage leukemia (trithorax homolog, Drosophila); translocated to, 3 [Source:HGNC Symbol;Acc:HGNC:7136] |
| **ADRB2** | | 0.48 | 5.13 | 8.E-07 | 47 x 1 | | 5 q32 | adrenoceptor beta 2, surface [Source:HGNC Symbol;Acc:HGNC:286] |
| **G6PD** | | 0.47 | 5.10 | 9.E-07 | 47 x 3 | | X q28 | glucose-6-phosphate dehydrogenase [Source:HGNC Symbol;Acc:HGNC:4057] |
| **PDLIM1** | | 0.47 | 5.09 | 1.E-06 | 50 x 1 | | 10 q23 | PDZ and LIM domain 1 [Source:HGNC Symbol;Acc:HGNC:2067] |
| **STAT2** | | 0.47 | 5.08 | 1.E-06 | 49 x 5 | | 12 q13 | signal transducer and activator of transcription 2, 113kDa [Source:HGNC Symbol;Acc:HGNC:11363] |
| **CAMK2N1** | | 0.47 | 5.05 | 1.E-06 | 48 x 1 | | 1 p36 | calcium/calmodulin-dependent protein kinase II inhibitor 1 [Source:HGNC Symbol;Acc:HGNC:24190] |
| **TMEM47** | | 0.47 | 5.04 | 1.E-06 | 48 x 4 | | X p21 | transmembrane protein 47 [Source:HGNC Symbol;Acc:HGNC:18515] |
| **TXNRD1** | | 0.47 | 5.02 | 1.E-06 | 48 x 4 | | 12 q23 | thioredoxin reductase 1 [Source:HGNC Symbol;Acc:HGNC:12437] |
| **ANKRD30B** | | 0.47 | 5.01 | 1.E-06 | 45 x 1 | | 18 p11 | ankyrin repeat domain 30B [Source:HGNC Symbol;Acc:HGNC:24165] |
| **EBF1** | | 0.47 | 5.01 | 1.E-06 | 47 x 1 | | 5 q33 | early B-cell factor 1 [Source:HGNC Symbol;Acc:HGNC:3126] |
| **TLE4** | | 0.46 | 4.97 | 2.E-06 | 46 x 2 | | 9 q21 | transducin-like enhancer of split 4 [Source:HGNC Symbol;Acc:HGNC:11840] |
| **AFAP1** | | 0.46 | 4.96 | 2.E-06 | 44 x 1 | | 4 p16 | actin filament associated protein 1 [Source:HGNC Symbol;Acc:HGNC:24017] |
| **SORBS2** | | 0.46 | 4.94 | 2.E-06 | 50 x 1 | | 4 q35 | sorbin and SH3 domain containing 2 [Source:HGNC Symbol;Acc:HGNC:24098] |
| **SAV1** | | 0.46 | 4.89 | 2.E-06 | 46 x 1 | | 14 q22 | salvador family WW domain containing protein 1 [Source:HGNC Symbol;Acc:HGNC:17795] |
| **SERTAD2** | | 0.46 | 4.87 | 2.E-06 | 43 x 1 | | 2 p14 | SERTA domain containing 2 [Source:HGNC Symbol;Acc:HGNC:30784] |
| **RIN2** | | 0.45 | 4.84 | 3.E-06 | 50 x 1 | | 20 p11 | Ras and Rab interactor 2 [Source:HGNC Symbol;Acc:HGNC:18750] |
| **FSTL1** | | 0.45 | 4.83 | 3.E-06 | 50 x 1 | | 3 q13 | follistatin-like 1 [Source:HGNC Symbol;Acc:HGNC:3972] |
| **PRSS23** | | 0.45 | 4.83 | 3.E-06 | 50 x 7 | | 11 q14 | protease, serine, 23 [Source:HGNC Symbol;Acc:HGNC:14370] |
| **HIPK3** | | 0.45 | 4.83 | 3.E-06 | 46 x 3 | | 11 p13 | homeodomain interacting protein kinase 3 [Source:HGNC Symbol;Acc:HGNC:4915] |
| **ARHGEF17** | | 0.45 | 4.82 | 3.E-06 | 50 x 4 | | 11 q13 | Rho guanine nucleotide exchange factor (GEF) 17 [Source:HGNC Symbol;Acc:HGNC:21726] |
| **PDLIM5** | | 0.45 | 4.81 | 3.E-06 | 50 x 1 | | 4 q22 | PDZ and LIM domain 5 [Source:HGNC Symbol;Acc:HGNC:17468] |
| **PEA15** | | 0.45 | 4.79 | 3.E-06 | 50 x 1 | | 1 q23 | phosphoprotein enriched in astrocytes 15 [Source:HGNC Symbol;Acc:HGNC:8822] |
| **FAT1** | | 0.45 | 4.79 | 3.E-06 | 49 x 1 | | 4 q35 | FAT atypical cadherin 1 [Source:HGNC Symbol;Acc:HGNC:3595] |
| **RBFOX2** | | 0.45 | 4.76 | 4.E-06 | 44 x 1 | | 22 q12 | RNA binding protein, fox-1 homolog (C. elegans) 2 [Source:HGNC Symbol;Acc:HGNC:9906] |
| **GPR37** | | 0.45 | 4.76 | 4.E-06 | 50 x 1 | | 7 q31 | G protein-coupled receptor 37 (endothelin receptor type B-like) [Source:HGNC Symbol;Acc:HGNC:4494] |
| **CBLB** | | 0.45 | 4.75 | 4.E-06 | 50 x 2 | | 3 q13 | Cbl proto-oncogene B, E3 ubiquitin protein ligase [Source:HGNC Symbol;Acc:HGNC:1542] |
| **PLK2** | | 0.44 | 4.71 | 4.E-06 | 50 x 1 | | 5 q11 | polo-like kinase 2 [Source:HGNC Symbol;Acc:HGNC:19699] |
| **RAB30** | | 0.44 | 4.71 | 5.E-06 | 50 x 5 | | 11 q14 | RAB30, member RAS oncogene family [Source:HGNC Symbol;Acc:HGNC:9770] |
| **PLCD3** | | 0.44 | 4.70 | 5.E-06 | 47 x 5 | | 17 q21 | phospholipase C, delta 3 [Source:HGNC Symbol;Acc:HGNC:9061] |
| **TRIM22** | | 0.44 | 4.70 | 5.E-06 | 50 x 3 | | 11 p15 | tripartite motif containing 22 [Source:HGNC Symbol;Acc:HGNC:16379] |
| **SLFN12** | | 0.44 | 4.70 | 5.E-06 | 49 x 1 | | 17 q12 | schlafen family member 12 [Source:HGNC Symbol;Acc:HGNC:25500] |
| **ZFHX3** | | 0.44 | 4.68 | 5.E-06 | 44 x 4 | | 16 q22 | zinc finger homeobox 3 [Source:HGNC Symbol;Acc:HGNC:777] |
| **SRPX** | | 0.44 | 4.68 | 5.E-06 | 50 x 1 | | X p11 | sushi-repeat containing protein, X-linked [Source:HGNC Symbol;Acc:HGNC:11309] |
| **ACTA2** | | 0.44 | 4.64 | 6.E-06 | 50 x 1 | | 10 q23 | actin, alpha 2, smooth muscle, aorta [Source:HGNC Symbol;Acc:HGNC:130] |
| **FOXO1** | | 0.44 | 4.61 | 7.E-06 | 50 x 5 | | 13 q14 | forkhead box O1 [Source:HGNC Symbol;Acc:HGNC:3819] |
| **LENG8** | | 0.44 | 4.60 | 7.E-06 | 44 x 5 | | 19 q13 | leukocyte receptor cluster (LRC) member 8 [Source:HGNC Symbol;Acc:HGNC:15500] |
| **DLGAP4** | | 0.44 | 4.59 | 7.E-06 | 50 x 1 | | 20 q11 | discs, large (Drosophila) homolog-associated protein 4 [Source:HGNC Symbol;Acc:HGNC:24476] |
| **GNG11** | | 0.44 | 4.59 | 7.E-06 | 46 x 4 | | 7 q21 | guanine nucleotide binding protein (G protein), gamma 11 [Source:HGNC Symbol;Acc:HGNC:4403] |
| **CDH6** | | 0.43 | 4.56 | 8.E-06 | 45 x 1 | | 5 p13 | cadherin 6, type 2, K-cadherin (fetal kidney) [Source:HGNC Symbol;Acc:HGNC:1765] |
| **RAPH1** | | 0.43 | 4.54 | 9.E-06 | 46 x 1 | | 2 q33 | Ras association (RalGDS/AF-6) and pleckstrin homology domains 1 [Source:HGNC Symbol;Acc:HGNC:14436] |
| **CAV1** | | 0.43 | 4.53 | 9.E-06 | 50 x 3 | | 7 q31 | caveolin 1, caveolae protein, 22kDa [Source:HGNC Symbol;Acc:HGNC:1527] |
| **JUND** | | 0.43 | 4.52 | 1.E-05 | 45 x 4 | | 19 p13 | jun D proto-oncogene [Source:HGNC Symbol;Acc:HGNC:6206] |
| **UBE2E2** | | 0.43 | 4.51 | 1.E-05 | 50 x 1 | | 3 p24 | ubiquitin-conjugating enzyme E2E 2 [Source:HGNC Symbol;Acc:HGNC:12478] |
| **HIST2H4A** | | 0.43 | 4.51 | 1.E-05 | 50 x 4 | | 1 q21 | histone cluster 2, H4a [Source:HGNC Symbol;Acc:HGNC:4794] |
| **TBL1XR1** | | 0.43 | 4.51 | 1.E-05 | 50 x 5 | | 3 q26 | transducin (beta)-like 1 X-linked receptor 1 [Source:HGNC Symbol;Acc:HGNC:29529] |
| **SYTL2** | | 0.43 | 4.50 | 1.E-05 | 50 x 4 | | 11 q14 | synaptotagmin-like 2 [Source:HGNC Symbol;Acc:HGNC:15585] |
| **STK17B** | | 0.43 | 4.50 | 1.E-05 | 50 x 2 | | 2 q32 | serine/threonine kinase 17b [Source:HGNC Symbol;Acc:HGNC:11396] |
| **SSBP2** | | 0.43 | 4.49 | 1.E-05 | 50 x 3 | | 5 q14 | single-stranded DNA binding protein 2 [Source:HGNC Symbol;Acc:HGNC:15831] |
| **ITGB1** | | 0.43 | 4.49 | 1.E-05 | 50 x 5 | | 10 p11 | integrin, beta 1 (fibronectin receptor, beta polypeptide, antigen CD29 includes MDF2, MSK12) [Source:HGNC Symbol;Acc:HGNC:6153] |
| **HIST2H4B** | | 0.43 | 4.47 | 1.E-05 | 49 x 4 | | 1 q21 | histone cluster 2, H4b [Source:HGNC Symbol;Acc:HGNC:29607] |
| **PXDC1** | | 0.43 | 4.47 | 1.E-05 | 50 x 1 | | 6 p25 | PX domain containing 1 [Source:HGNC Symbol;Acc:HGNC:21361] |
| **CYR61** | | 0.43 | 4.47 | 1.E-05 | 47 x 1 | | 1 p22 | cysteine-rich, angiogenic inducer, 61 [Source:HGNC Symbol;Acc:HGNC:2654] |
| **CWC25** | | 0.43 | 4.46 | 1.E-05 | 45 x 1 | | NA | CWC25 spliceosome-associated protein homolog (S. cerevisiae) [Source:HGNC Symbol;Acc:HGNC:25989] |
| **ARHGAP29** | | 0.42 | 4.42 | 1.E-05 | 44 x 3 | | 1 p22 | Rho GTPase activating protein 29 [Source:HGNC Symbol;Acc:HGNC:30207] |
| **DNAJB4** | | 0.42 | 4.42 | 1.E-05 | 50 x 1 | | 1 p31 | DnaJ (Hsp40) homolog, subfamily B, member 4 [Source:HGNC Symbol;Acc:HGNC:14886] |
| **DNM2** | | 0.42 | 4.40 | 1.E-05 | 45 x 3 | | 19 p13 | dynamin 2 [Source:HGNC Symbol;Acc:HGNC:2974] |
| **EVI5** | | 0.42 | 4.39 | 2.E-05 | 43 x 1 | | 1 p22 | ecotropic viral integration site 5 [Source:HGNC Symbol;Acc:HGNC:3501] |
| **MTMR10** | | 0.42 | 4.39 | 2.E-05 | 44 x 2 | | NA | myotubularin related protein 10 [Source:HGNC Symbol;Acc:HGNC:25999] |
| **ATXN1** | | 0.42 | 4.38 | 2.E-05 | 46 x 5 | | 6 p22 | ataxin 1 [Source:HGNC Symbol;Acc:HGNC:10548] |
| **ABHD4** | | 0.42 | 4.37 | 2.E-05 | 49 x 1 | | 14 q11 | abhydrolase domain containing 4 [Source:HGNC Symbol;Acc:HGNC:20154] |
| **PEG10** | | 0.42 | 4.37 | 2.E-05 | 46 x 2 | | 7 q21 | paternally expressed 10 [Source:HGNC Symbol;Acc:HGNC:14005] |
| **ZFP36L1** | | 0.42 | 4.36 | 2.E-05 | 45 x 4 | | 14 q24 | ZFP36 ring finger protein-like 1 [Source:HGNC Symbol;Acc:HGNC:1107] |
| **PRKCA** | | 0.42 | 4.35 | 2.E-05 | 48 x 1 | | 17 q24 | protein kinase C, alpha [Source:HGNC Symbol;Acc:HGNC:9393] |
| **GLRX** | | 0.42 | 4.35 | 2.E-05 | 50 x 2 | | 5 q15 | glutaredoxin (thioltransferase) [Source:HGNC Symbol;Acc:HGNC:4330] |
| **FMN2** | | 0.42 | 4.35 | 2.E-05 | 48 x 1 | | 1 q43 | formin 2 [Source:HGNC Symbol;Acc:HGNC:14074] |
| **WRNIP1** | | 0.42 | 4.33 | 2.E-05 | 47 x 1 | | 6 p25 | Werner helicase interacting protein 1 [Source:HGNC Symbol;Acc:HGNC:20876] |
| **TGIF1** | | 0.42 | 4.33 | 2.E-05 | 50 x 1 | | 18 p11 | TGFB-induced factor homeobox 1 [Source:HGNC Symbol;Acc:HGNC:11776] |
| **FAM46A** | | 0.41 | 4.33 | 2.E-05 | 47 x 1 | | 6 q14 | family with sequence similarity 46, member A [Source:HGNC Symbol;Acc:HGNC:18345] |
| **ITGA3** | | 0.41 | 4.32 | 2.E-05 | 48 x 5 | | 17 q21 | integrin, alpha 3 (antigen CD49C, alpha 3 subunit of VLA-3 receptor) [Source:HGNC Symbol;Acc:HGNC:6139] |
| **MYH9** | | 0.41 | 4.32 | 2.E-05 | 46 x 6 | | 22 q12 | myosin, heavy chain 9, non-muscle [Source:HGNC Symbol;Acc:HGNC:7579] |
| **PRRG1** | | 0.41 | 4.31 | 2.E-05 | 46 x 1 | | X p21 | proline rich Gla (G-carboxyglutamic acid) 1 [Source:HGNC Symbol;Acc:HGNC:9469] |
| **PDGFA** | | 0.41 | 4.30 | 2.E-05 | 49 x 1 | | 7 p22 | platelet-derived growth factor alpha polypeptide [Source:HGNC Symbol;Acc:HGNC:8799] |
| **KLF13** | | 0.41 | 4.30 | 2.E-05 | 47 x 5 | | NA | Kruppel-like factor 13 [Source:HGNC Symbol;Acc:HGNC:13672] |
| **SPARC** | | 0.41 | 4.29 | 2.E-05 | 50 x 2 | | 5 q33 | secreted protein, acidic, cysteine-rich (osteonectin) [Source:HGNC Symbol;Acc:HGNC:11219] |
| **PDLIM3** | | 0.41 | 4.27 | 2.E-05 | 50 x 2 | | 4 q35 | PDZ and LIM domain 3 [Source:HGNC Symbol;Acc:HGNC:20767] |
| **NEDD9** | | 0.41 | 4.27 | 2.E-05 | 48 x 2 | | 6 p24 | neural precursor cell expressed, developmentally down-regulated 9 [Source:HGNC Symbol;Acc:HGNC:7733] |
| **CAMK2D** | | 0.41 | 4.27 | 2.E-05 | 50 x 1 | | 4 q26 | calcium/calmodulin-dependent protein kinase II delta [Source:HGNC Symbol;Acc:HGNC:1462] |
| **CDC42BPB** | | 0.41 | 4.27 | 2.E-05 | 46 x 5 | | 14 q32 | CDC42 binding protein kinase beta (DMPK-like) [Source:HGNC Symbol;Acc:HGNC:1738] |
| **SNAP23** | | 0.41 | 4.25 | 3.E-05 | 50 x 1 | | 15 q15 | synaptosomal-associated protein, 23kDa [Source:HGNC Symbol;Acc:HGNC:11131] |
| **WDR47** | | 0.41 | 4.23 | 3.E-05 | 45 x 1 | | 1 p13 | WD repeat domain 47 [Source:HGNC Symbol;Acc:HGNC:29141] |
| **ACTN4** | | 0.41 | 4.22 | 3.E-05 | 45 x 1 | | 19 q13 | actinin, alpha 4 [Source:HGNC Symbol;Acc:HGNC:166] |
| **PPP1R15A** | | 0.40 | 4.19 | 3.E-05 | 49 x 4 | | 19 q13 | protein phosphatase 1, regulatory subunit 15A [Source:HGNC Symbol;Acc:HGNC:14375] |
| **CAPN12** | | 0.40 | 4.18 | 3.E-05 | 47 x 7 | | 19 q13 | calpain 12 [Source:HGNC Symbol;Acc:HGNC:13249] |
| **CTDSPL** | | 0.40 | 4.16 | 4.E-05 | 49 x 1 | | 3 p22 | CTD (carboxy-terminal domain, RNA polymerase II, polypeptide A) small phosphatase-like [Source:HGNC Symbol;Acc:HGNC:16890] |
| **CTTNBP2NL** | | 0.40 | 4.16 | 4.E-05 | 46 x 1 | | 1 p13 | CTTNBP2 N-terminal like [Source:HGNC Symbol;Acc:HGNC:25330] |
| **JDP2** | | 0.40 | 4.15 | 4.E-05 | 44 x 1 | | 14 q24 | Jun dimerization protein 2 [Source:HGNC Symbol;Acc:HGNC:17546] |
| **PPP3CA** | | 0.40 | 4.15 | 4.E-05 | 46 x 4 | | 4 q24 | protein phosphatase 3, catalytic subunit, alpha isozyme [Source:HGNC Symbol;Acc:HGNC:9314] |
| **CLEC2B** | | 0.40 | 4.10 | 5.E-05 | 47 x 4 | | 12 p13 | C-type lectin domain family 2, member B [Source:HGNC Symbol;Acc:HGNC:2053] |
| **MEF2D** | | 0.40 | 4.09 | 5.E-05 | 44 x 3 | | 1 q22 | myocyte enhancer factor 2D [Source:HGNC Symbol;Acc:HGNC:6997] |
| **KCND3** | | 0.39 | 4.07 | 5.E-05 | 44 x 2 | | 1 p13 | potassium channel, voltage gated Shal related subfamily D, member 3 [Source:HGNC Symbol;Acc:HGNC:6239] |
| **ABL2** | | 0.39 | 4.07 | 5.E-05 | 46 x 1 | | 1 q25 | ABL proto-oncogene 2, non-receptor tyrosine kinase [Source:HGNC Symbol;Acc:HGNC:77] |
| **DTWD1** | | 0.39 | 4.06 | 5.E-05 | 50 x 3 | | 15 q21 | DTW domain containing 1 [Source:HGNC Symbol;Acc:HGNC:30926] |
| **SHC4** | | 0.39 | 4.05 | 5.E-05 | 44 x 1 | | 15 q21 | SHC (Src homology 2 domain containing) family, member 4 [Source:HGNC Symbol;Acc:HGNC:16743] |
| **PURB** | | 0.39 | 4.05 | 5.E-05 | 43 x 3 | | 7 p13 | purine-rich element binding protein B [Source:HGNC Symbol;Acc:HGNC:9702] |
| **FAM3C** | | 0.39 | 4.05 | 5.E-05 | 46 x 1 | | 7 q31 | family with sequence similarity 3, member C [Source:HGNC Symbol;Acc:HGNC:18664] |
| **FUK** | | 0.39 | 4.03 | 6.E-05 | 46 x 7 | | 16 q22 | fucokinase [Source:HGNC Symbol;Acc:HGNC:29500] |
| **DHFRL1** | | 0.39 | 4.03 | 6.E-05 | 44 x 1 | | 3 q11 | dihydrofolate reductase-like 1 [Source:HGNC Symbol;Acc:HGNC:27309] |
| **ADAMTSL5** | | 0.39 | 4.03 | 6.E-05 | 45 x 3 | | 19 p13 | ADAMTS-like 5 [Source:HGNC Symbol;Acc:HGNC:27912] |
| **HIST2H2AA4** | | 0.39 | 4.02 | 6.E-05 | 44 x 5 | | 1 q21 | histone cluster 2, H2aa4 [Source:HGNC Symbol;Acc:HGNC:29668] |
| **PCLO** | | 0.39 | 4.02 | 6.E-05 | 46 x 3 | | 7 q21 | piccolo presynaptic cytomatrix protein [Source:HGNC Symbol;Acc:HGNC:13406] |
| **DCLK2** | | 0.39 | 4.01 | 6.E-05 | 44 x 4 | | 4 q31 | doublecortin-like kinase 2 [Source:HGNC Symbol;Acc:HGNC:19002] |
| **PIK3CA** | | 0.39 | 4.00 | 7.E-05 | 45 x 1 | | 3 q26 | phosphatidylinositol-4,5-bisphosphate 3-kinase, catalytic subunit alpha [Source:HGNC Symbol;Acc:HGNC:8975] |
| **ZNF217** | | 0.39 | 3.99 | 7.E-05 | 47 x 4 | | 20 q13 | zinc finger protein 217 [Source:HGNC Symbol;Acc:HGNC:13009] |
| **VIM** | | 0.39 | 3.99 | 7.E-05 | 50 x 2 | | 10 p13 | vimentin [Source:HGNC Symbol;Acc:HGNC:12692] |
| **KIAA0355** | | 0.39 | 3.99 | 7.E-05 | 44 x 2 | | NA | KIAA0355 [Source:HGNC Symbol;Acc:HGNC:29016] |
| **SMAD3** | | 0.39 | 3.98 | 7.E-05 | 47 x 1 | | 15 q22 | SMAD family member 3 [Source:HGNC Symbol;Acc:HGNC:6769] |
| **RNF220** | | 0.39 | 3.98 | 7.E-05 | 47 x 2 | | 1 p34 | ring finger protein 220 [Source:HGNC Symbol;Acc:HGNC:25552] |
| **DST** | | 0.39 | 3.97 | 7.E-05 | 46 x 1 | | 6 p12 | dystonin [Source:HGNC Symbol;Acc:HGNC:1090] |
| **RGS4** | | 0.39 | 3.97 | 7.E-05 | 50 x 1 | | 1 q23 | regulator of G-protein signaling 4 [Source:HGNC Symbol;Acc:HGNC:10000] |
| **CASC4** | | 0.39 | 3.97 | 7.E-05 | 47 x 4 | | 15 q15 | cancer susceptibility candidate 4 [Source:HGNC Symbol;Acc:HGNC:24892] |
| **CHML** | | 0.38 | 3.96 | 8.E-05 | 46 x 1 | | 1 q43 | choroideremia-like (Rab escort protein 2) [Source:HGNC Symbol;Acc:HGNC:1941] |
| **P4HA1** | | 0.38 | 3.95 | 8.E-05 | 50 x 1 | | 10 q22 | prolyl 4-hydroxylase, alpha polypeptide I [Source:HGNC Symbol;Acc:HGNC:8546] |
| **TAGLN2** | | 0.38 | 3.94 | 8.E-05 | 50 x 2 | | 1 q23 | transgelin 2 [Source:HGNC Symbol;Acc:HGNC:11554] |
| **OTUD7B** | | 0.38 | 3.93 | 8.E-05 | 43 x 4 | | 1 q21 | OTU deubiquitinase 7B [Source:HGNC Symbol;Acc:HGNC:16683] |
| **PIEZO2** | | 0.38 | 3.92 | 9.E-05 | 50 x 4 | | 18 p11 | piezo-type mechanosensitive ion channel component 2 [Source:HGNC Symbol;Acc:HGNC:26270] |
| **S100A10** | | 0.38 | 3.92 | 9.E-05 | 50 x 4 | | 1 q21 | S100 calcium binding protein A10 [Source:HGNC Symbol;Acc:HGNC:10487] |
| **SERPINI1** | | 0.38 | 3.92 | 9.E-05 | 50 x 1 | | 3 q26 | serpin peptidase inhibitor, clade I (neuroserpin), member 1 [Source:HGNC Symbol;Acc:HGNC:8943] |
| **KIRREL** | | 0.38 | 3.91 | 9.E-05 | 45 x 4 | | 1 q23 | kin of IRRE like (Drosophila) [Source:HGNC Symbol;Acc:HGNC:15734] |
| **SATB1** | | 0.38 | 3.89 | 9.E-05 | 50 x 1 | | 3 p24 | SATB homeobox 1 [Source:HGNC Symbol;Acc:HGNC:10541] |
| **SYNE1** | | 0.38 | 3.88 | 1.E-04 | 49 x 1 | | 6 q25 | spectrin repeat containing, nuclear envelope 1 [Source:HGNC Symbol;Acc:HGNC:17089] |
| **GADD45A** | | 0.38 | 3.88 | 1.E-04 | 50 x 5 | | 1 p31 | growth arrest and DNA-damage-inducible, alpha [Source:HGNC Symbol;Acc:HGNC:4095] |
| **HHIP** | | 0.38 | 3.88 | 1.E-04 | 48 x 4 | | 4 q31 | hedgehog interacting protein [Source:HGNC Symbol;Acc:HGNC:14866] |
| **ZNF383** | | 0.38 | 3.87 | 1.E-04 | 46 x 7 | | 19 q13 | zinc finger protein 383 [Source:HGNC Symbol;Acc:HGNC:18609] |
| **MBOAT2** | | 0.38 | 3.87 | 1.E-04 | 50 x 1 | | 2 p25 | membrane bound O-acyltransferase domain containing 2 [Source:HGNC Symbol;Acc:HGNC:25193] |
| **ZNF608** | | 0.38 | 3.86 | 1.E-04 | 46 x 4 | | 5 q23 | zinc finger protein 608 [Source:HGNC Symbol;Acc:HGNC:29238] |
| **ARHGEF12** | | 0.38 | 3.85 | 1.E-04 | 45 x 5 | | 11 q23 | Rho guanine nucleotide exchange factor (GEF) 12 [Source:HGNC Symbol;Acc:HGNC:14193] |
| **ARHGEF10** | | 0.38 | 3.85 | 1.E-04 | 46 x 4 | | NA | Rho guanine nucleotide exchange factor (GEF) 10 [Source:HGNC Symbol;Acc:HGNC:14103] |
| **CBR4** | | 0.38 | 3.84 | 1.E-04 | 50 x 5 | | 4 q32 | carbonyl reductase 4 [Source:HGNC Symbol;Acc:HGNC:25891] |
| **REST** | | 0.38 | 3.84 | 1.E-04 | 45 x 1 | | 4 q12 | RE1-silencing transcription factor [Source:HGNC Symbol;Acc:HGNC:9966] |
| **RASAL2** | | 0.37 | 3.83 | 1.E-04 | 44 x 1 | | 1 q25 | RAS protein activator like 2 [Source:HGNC Symbol;Acc:HGNC:9874] |
| **SLC7A11** | | 0.37 | 3.83 | 1.E-04 | 49 x 4 | | 4 q28 | solute carrier family 7 (anionic amino acid transporter light chain, xc- system), member 11 [Source:HGNC Symbol;Acc:HGNC:11059] |
| **TEAD1** | | 0.37 | 3.82 | 1.E-04 | 46 x 1 | | 11 p15 | TEA domain family member 1 (SV40 transcriptional enhancer factor) [Source:HGNC Symbol;Acc:HGNC:11714] |
| **FBXW7** | | 0.37 | 3.78 | 1.E-04 | 47 x 1 | | 4 q31 | F-box and WD repeat domain containing 7, E3 ubiquitin protein ligase [Source:HGNC Symbol;Acc:HGNC:16712] |
| **GBE1** | | 0.37 | 3.77 | 1.E-04 | 50 x 3 | | 3 p12 | glucan (1,4-alpha-), branching enzyme 1 [Source:HGNC Symbol;Acc:HGNC:4180] |
| **SLC40A1** | | 0.37 | 3.74 | 2.E-04 | 50 x 4 | | 2 q32 | solute carrier family 40 (iron-regulated transporter), member 1 [Source:HGNC Symbol;Acc:HGNC:10909] |
| **PGRMC2** | | 0.37 | 3.74 | 2.E-04 | 48 x 5 | | 4 q28 | progesterone receptor membrane component 2 [Source:HGNC Symbol;Acc:HGNC:16089] |
| **CLOCK** | | 0.37 | 3.73 | 2.E-04 | 47 x 1 | | 4 q12 | clock circadian regulator [Source:HGNC Symbol;Acc:HGNC:2082] |
| **NBEAL1** | | 0.36 | 3.72 | 2.E-04 | 46 x 5 | | 2 q33 | neurobeachin-like 1 [Source:HGNC Symbol;Acc:HGNC:20681] |
| **GLG1** | | 0.36 | 3.71 | 2.E-04 | 48 x 5 | | 16 q23 | golgi glycoprotein 1 [Source:HGNC Symbol;Acc:HGNC:4316] |
| **TCF7L2** | | 0.36 | 3.71 | 2.E-04 | 46 x 3 | | 10 q25 | transcription factor 7-like 2 (T-cell specific, HMG-box) [Source:HGNC Symbol;Acc:HGNC:11641] |
| **PANK1** | | 0.36 | 3.70 | 2.E-04 | 46 x 1 | | 10 q23 | pantothenate kinase 1 [Source:HGNC Symbol;Acc:HGNC:8598] |
| **FAM110B** | | 0.36 | 3.70 | 2.E-04 | 50 x 6 | | 8 q12 | family with sequence similarity 110, member B [Source:HGNC Symbol;Acc:HGNC:28587] |
| **CNN2** | | 0.36 | 3.70 | 2.E-04 | 50 x 1 | | 19 p13 | calponin 2 [Source:HGNC Symbol;Acc:HGNC:2156] |
| **LIN7A** | | 0.36 | 3.69 | 2.E-04 | 48 x 6 | | 12 q21 | lin-7 homolog A (C. elegans) [Source:HGNC Symbol;Acc:HGNC:17787] |
| **TSC22D2** | | 0.36 | 3.69 | 2.E-04 | 44 x 1 | | 3 q25 | TSC22 domain family, member 2 [Source:HGNC Symbol;Acc:HGNC:29095] |
| **PHACTR2** | | 0.36 | 3.68 | 2.E-04 | 47 x 4 | | 6 q24 | phosphatase and actin regulator 2 [Source:HGNC Symbol;Acc:HGNC:20956] |
| **HEG1** | | 0.36 | 3.68 | 2.E-04 | 47 x 7 | | 3 q21 | heart development protein with EGF-like domains 1 [Source:HGNC Symbol;Acc:HGNC:29227] |
| **TPST1** | | 0.36 | 3.67 | 2.E-04 | 48 x 1 | | 7 q11 | tyrosylprotein sulfotransferase 1 [Source:HGNC Symbol;Acc:HGNC:12020] |
| **PICALM** | | 0.36 | 3.66 | 2.E-04 | 49 x 7 | | 11 q14 | phosphatidylinositol binding clathrin assembly protein [Source:HGNC Symbol;Acc:HGNC:15514] |
| **FN1** | | 0.36 | 3.66 | 2.E-04 | 50 x 1 | | 2 q35 | fibronectin 1 [Source:HGNC Symbol;Acc:HGNC:3778] |
| **RTN2** | | 0.36 | 3.64 | 2.E-04 | 44 x 5 | | 19 q13 | reticulon 2 [Source:HGNC Symbol;Acc:HGNC:10468] |
| **PLBD2** | | 0.36 | 3.64 | 2.E-04 | 45 x 1 | | 12 q24 | phospholipase B domain containing 2 [Source:HGNC Symbol;Acc:HGNC:27283] |
| **RARG** | | 0.36 | 3.63 | 2.E-04 | 43 x 1 | | 12 q13 | retinoic acid receptor, gamma [Source:HGNC Symbol;Acc:HGNC:9866] |
| **FNDC1** | | 0.36 | 3.63 | 2.E-04 | 45 x 1 | | 6 q25 | fibronectin type III domain containing 1 [Source:HGNC Symbol;Acc:HGNC:21184] |
| **COL11A1** | | 0.36 | 3.62 | 2.E-04 | 45 x 1 | | 1 p21 | collagen, type XI, alpha 1 [Source:HGNC Symbol;Acc:HGNC:2186] |
| **PELO** | | 0.36 | 3.62 | 2.E-04 | 50 x 1 | | 5 q11 | pelota homolog (Drosophila) [Source:HGNC Symbol;Acc:HGNC:8829] |
| **LRP12** | | 0.36 | 3.62 | 2.E-04 | 48 x 1 | | 8 q22 | low density lipoprotein receptor-related protein 12 [Source:HGNC Symbol;Acc:HGNC:31708] |
| **MCL1** | | 0.36 | 3.61 | 2.E-04 | 44 x 1 | | 1 q21 | myeloid cell leukemia 1 [Source:HGNC Symbol;Acc:HGNC:6943] |
| **YPEL5** | | 0.35 | 3.60 | 3.E-04 | 50 x 1 | | 2 p23 | yippee-like 5 (Drosophila) [Source:HGNC Symbol;Acc:HGNC:18329] |
| **ARMC9** | | 0.35 | 3.60 | 3.E-04 | 50 x 3 | | 2 q37 | armadillo repeat containing 9 [Source:HGNC Symbol;Acc:HGNC:20730] |
| **HIST1H2BF** | | 0.35 | 3.59 | 3.E-04 | 46 x 4 | | 6 p22 | histone cluster 1, H2bf [Source:HGNC Symbol;Acc:HGNC:4752] |
| **NCOA3** | | 0.35 | 3.59 | 3.E-04 | 47 x 1 | | 20 q13 | nuclear receptor coactivator 3 [Source:HGNC Symbol;Acc:HGNC:7670] |
| **HIST1H3D** | | 0.35 | 3.58 | 3.E-04 | 50 x 1 | | 6 p22 | histone cluster 1, H3d [Source:HGNC Symbol;Acc:HGNC:4767] |
| **TOB1** | | 0.35 | 3.58 | 3.E-04 | 50 x 5 | | 17 q21 | transducer of ERBB2, 1 [Source:HGNC Symbol;Acc:HGNC:11979] |
| **PALMD** | | 0.35 | 3.58 | 3.E-04 | 50 x 1 | | 1 p21 | palmdelphin [Source:HGNC Symbol;Acc:HGNC:15846] |
| **CSAD** | | 0.35 | 3.57 | 3.E-04 | 47 x 4 | | 12 q13 | cysteine sulfinic acid decarboxylase [Source:HGNC Symbol;Acc:HGNC:18966] |
| **SFXN3** | | 0.35 | 3.57 | 3.E-04 | 47 x 4 | | 10 q24 | sideroflexin 3 [Source:HGNC Symbol;Acc:HGNC:16087] |
| **RND3** | | 0.35 | 3.57 | 3.E-04 | 50 x 1 | | 2 q23 | Rho family GTPase 3 [Source:HGNC Symbol;Acc:HGNC:671] |
| **MBTPS1** | | 0.35 | 3.57 | 3.E-04 | 46 x 6 | | 16 q24 | membrane-bound transcription factor peptidase, site 1 [Source:HGNC Symbol;Acc:HGNC:15456] |
| **FABP3** | | 0.35 | 3.55 | 3.E-04 | 50 x 1 | | 1 p35 | fatty acid binding protein 3, muscle and heart [Source:HGNC Symbol;Acc:HGNC:3557] |
| **C5orf15** | | 0.35 | 3.55 | 3.E-04 | 48 x 5 | | 5 q31 | chromosome 5 open reading frame 15 [Source:HGNC Symbol;Acc:HGNC:20656] |
| **PPAP2A** | | 0.35 | 3.55 | 3.E-04 | 50 x 4 | | 5 q11 | phosphatidic acid phosphatase type 2A [Source:HGNC Symbol;Acc:HGNC:9228] |
| **PTPRS** | | 0.35 | 3.53 | 3.E-04 | 43 x 4 | | 19 p13 | protein tyrosine phosphatase, receptor type, S [Source:HGNC Symbol;Acc:HGNC:9681] |
| **TUBB2B** | | 0.35 | 3.53 | 3.E-04 | 46 x 6 | | 6 p25 | tubulin, beta 2B class IIb [Source:HGNC Symbol;Acc:HGNC:30829] |
| **GZF1** | | 0.35 | 3.52 | 3.E-04 | 46 x 1 | | 20 p11 | GDNF-inducible zinc finger protein 1 [Source:HGNC Symbol;Acc:HGNC:15808] |
| **ID4** | | 0.35 | 3.52 | 3.E-04 | 47 x 1 | | 6 p22 | inhibitor of DNA binding 4, dominant negative helix-loop-helix protein [Source:HGNC Symbol;Acc:HGNC:5363] |
| **RHOB** | | 0.35 | 3.52 | 3.E-04 | 44 x 1 | | 2 p24 | ras homolog family member B [Source:HGNC Symbol;Acc:HGNC:668] |
| **LRP2BP** | | 0.35 | 3.51 | 3.E-04 | 50 x 5 | | 4 q35 | LRP2 binding protein [Source:HGNC Symbol;Acc:HGNC:25434] |
| **ANKRD28** | | 0.35 | 3.50 | 4.E-04 | 50 x 5 | | 3 p25 | ankyrin repeat domain 28 [Source:HGNC Symbol;Acc:HGNC:29024] |
| **WWTR1** | | 0.35 | 3.50 | 4.E-04 | 45 x 1 | | 3 q25 | WW domain containing transcription regulator 1 [Source:HGNC Symbol;Acc:HGNC:24042] |
| **CD151** | | 0.35 | 3.49 | 4.E-04 | 44 x 3 | | 11 p15 | CD151 molecule (Raph blood group) [Source:HGNC Symbol;Acc:HGNC:1630] |
| **GPBP1L1** | | 0.35 | 3.49 | 4.E-04 | 50 x 4 | | 1 p34 | GC-rich promoter binding protein 1-like 1 [Source:HGNC Symbol;Acc:HGNC:28843] |
| **ECM1** | | 0.34 | 3.48 | 4.E-04 | 49 x 1 | | 1 q21 | extracellular matrix protein 1 [Source:HGNC Symbol;Acc:HGNC:3153] |
| **KITLG** | | 0.34 | 3.46 | 4.E-04 | 48 x 1 | | 12 q21 | KIT ligand [Source:HGNC Symbol;Acc:HGNC:6343] |
| **BICC1** | | 0.34 | 3.46 | 4.E-04 | 47 x 1 | | 10 q21 | BicC family RNA binding protein 1 [Source:HGNC Symbol;Acc:HGNC:19351] |
| **MID1** | | 0.34 | 3.46 | 4.E-04 | 50 x 3 | | X p22 | midline 1 [Source:HGNC Symbol;Acc:HGNC:7095] |
| **SULF2** | | 0.34 | 3.45 | 4.E-04 | 50 x 3 | | 20 q13 | sulfatase 2 [Source:HGNC Symbol;Acc:HGNC:20392] |
| **GOLPH3** | | 0.34 | 3.45 | 4.E-04 | 46 x 1 | | 5 p13 | golgi phosphoprotein 3 (coat-protein) [Source:HGNC Symbol;Acc:HGNC:15452] |
| **CWF19L1** | | 0.34 | 3.44 | 4.E-04 | 49 x 4 | | 10 q24 | CWF19-like 1, cell cycle control (S. pombe) [Source:HGNC Symbol;Acc:HGNC:25613] |
| **ABCB10** | | 0.34 | 3.44 | 4.E-04 | 45 x 3 | | 1 q42 | ATP-binding cassette, sub-family B (MDR/TAP), member 10 [Source:HGNC Symbol;Acc:HGNC:41] |
| **KIF13A** | | 0.34 | 3.44 | 4.E-04 | 45 x 3 | | 6 p22 | kinesin family member 13A [Source:HGNC Symbol;Acc:HGNC:14566] |
| **RALGPS2** | | 0.34 | 3.44 | 4.E-04 | 50 x 5 | | 1 q25 | Ral GEF with PH domain and SH3 binding motif 2 [Source:HGNC Symbol;Acc:HGNC:30279] |
| **IRF2BPL** | | 0.34 | 3.44 | 4.E-04 | 42 x 1 | | 14 q24 | interferon regulatory factor 2 binding protein-like [Source:HGNC Symbol;Acc:HGNC:14282] |
| **PTPRG** | | 0.34 | 3.44 | 4.E-04 | 45 x 4 | | 3 p14 | protein tyrosine phosphatase, receptor type, G [Source:HGNC Symbol;Acc:HGNC:9671] |
| **C11orf54** | | 0.34 | 3.43 | 5.E-04 | 48 x 5 | | 11 q21 | chromosome 11 open reading frame 54 [Source:HGNC Symbol;Acc:HGNC:30204] |
| **RELN** | | 0.34 | 3.41 | 5.E-04 | 44 x 2 | | 7 q22 | reelin [Source:HGNC Symbol;Acc:HGNC:9957] |
| **TMEM39A** | | 0.34 | 3.40 | 5.E-04 | 50 x 1 | | 3 q13 | transmembrane protein 39A [Source:HGNC Symbol;Acc:HGNC:25600] |
| **DCBLD2** | | 0.34 | 3.39 | 5.E-04 | 47 x 1 | | 3 q12 | discoidin, CUB and LCCL domain containing 2 [Source:HGNC Symbol;Acc:HGNC:24627] |
| **FAM171B** | | 0.34 | 3.38 | 5.E-04 | 46 x 1 | | 2 q32 | family with sequence similarity 171, member B [Source:HGNC Symbol;Acc:HGNC:29412] |
| **ZEB2** | | 0.33 | 3.36 | 6.E-04 | 42 x 1 | | 2 q22 | zinc finger E-box binding homeobox 2 [Source:HGNC Symbol;Acc:HGNC:14881] |
| **PALLD** | | 0.33 | 3.35 | 6.E-04 | 50 x 4 | | 4 q32 | palladin, cytoskeletal associated protein [Source:HGNC Symbol;Acc:HGNC:17068] |
| **PMP22** | | 0.33 | 3.35 | 6.E-04 | 48 x 1 | | 17 p12 | peripheral myelin protein 22 [Source:HGNC Symbol;Acc:HGNC:9118] |
| **CTGF** | | 0.33 | 3.35 | 6.E-04 | 50 x 1 | | 6 q23 | connective tissue growth factor [Source:HGNC Symbol;Acc:HGNC:2500] |
| **SP1** | | 0.33 | 3.35 | 6.E-04 | 45 x 1 | | 12 q13 | Sp1 transcription factor [Source:HGNC Symbol;Acc:HGNC:11205] |
| **ANTXR1** | | 0.33 | 3.34 | 6.E-04 | 45 x 5 | | 2 p13 | anthrax toxin receptor 1 [Source:HGNC Symbol;Acc:HGNC:21014] |
| **FAM107B** | | 0.33 | 3.33 | 6.E-04 | 46 x 3 | | 10 p13 | family with sequence similarity 107, member B [Source:HGNC Symbol;Acc:HGNC:23726] |
| **CKAP4** | | 0.33 | 3.32 | 6.E-04 | 44 x 3 | | 12 q23 | cytoskeleton-associated protein 4 [Source:HGNC Symbol;Acc:HGNC:16991] |
| **RERE** | | 0.33 | 3.32 | 6.E-04 | 48 x 5 | | 1 p36 | arginine-glutamic acid dipeptide (RE) repeats [Source:HGNC Symbol;Acc:HGNC:9965] |
| **RAB3IP** | | 0.33 | 3.31 | 7.E-04 | 50 x 3 | | 12 q15 | RAB3A interacting protein [Source:HGNC Symbol;Acc:HGNC:16508] |
| **PLOD2** | | 0.33 | 3.31 | 7.E-04 | 49 x 3 | | 3 q24 | procollagen-lysine, 2-oxoglutarate 5-dioxygenase 2 [Source:HGNC Symbol;Acc:HGNC:9082] |
| **MYEOV** | | 0.33 | 3.31 | 7.E-04 | 47 x 4 | | 11 q13 | myeloma overexpressed [Source:HGNC Symbol;Acc:HGNC:7563] |
| **PKIG** | | 0.33 | 3.31 | 7.E-04 | 50 x 5 | | 20 q13 | protein kinase (cAMP-dependent, catalytic) inhibitor gamma [Source:HGNC Symbol;Acc:HGNC:9019] |
| **TRIP6** | | 0.33 | 3.29 | 7.E-04 | 50 x 6 | | 7 q22 | thyroid hormone receptor interactor 6 [Source:HGNC Symbol;Acc:HGNC:12311] |
| **ROCK2** | | 0.33 | 3.28 | 7.E-04 | 46 x 1 | | 2 p25 | Rho-associated, coiled-coil containing protein kinase 2 [Source:HGNC Symbol;Acc:HGNC:10252] |
| **RECK** | | 0.33 | 3.26 | 8.E-04 | 45 x 1 | | 9 p13 | reversion-inducing-cysteine-rich protein with kazal motifs [Source:HGNC Symbol;Acc:HGNC:11345] |
| **MACF1** | | 0.32 | 3.26 | 8.E-04 | 49 x 6 | | 1 p34 | microtubule-actin crosslinking factor 1 [Source:HGNC Symbol;Acc:HGNC:13664] |
| **EXOC6B** | | 0.32 | 3.26 | 8.E-04 | 45 x 6 | | 2 p13 | exocyst complex component 6B [Source:HGNC Symbol;Acc:HGNC:17085] |
| **PRKCI** | | 0.32 | 3.26 | 8.E-04 | 47 x 3 | | 3 q26 | protein kinase C, iota [Source:HGNC Symbol;Acc:HGNC:9404] |
| **ADCY9** | | 0.32 | 3.25 | 8.E-04 | 44 x 1 | | 16 p13 | adenylate cyclase 9 [Source:HGNC Symbol;Acc:HGNC:240] |
| **CSNK1E** | | 0.32 | 3.25 | 8.E-04 | 45 x 6 | | 22 q13 | casein kinase 1, epsilon [Source:HGNC Symbol;Acc:HGNC:2453] |
| **LRRC59** | | 0.32 | 3.25 | 8.E-04 | 44 x 3 | | 17 q21 | leucine rich repeat containing 59 [Source:HGNC Symbol;Acc:HGNC:28817] |
| **GALNT2** | | 0.32 | 3.25 | 8.E-04 | 44 x 1 | | 1 q42 | polypeptide N-acetylgalactosaminyltransferase 2 [Source:HGNC Symbol;Acc:HGNC:4124] |
| **ETV1** | | 0.32 | 3.23 | 9.E-04 | 48 x 4 | | 7 p21 | ets variant 1 [Source:HGNC Symbol;Acc:HGNC:3490] |
| **JUP** | | 0.32 | 3.22 | 9.E-04 | 43 x 1 | | 17 q21 | junction plakoglobin [Source:HGNC Symbol;Acc:HGNC:6207] |
| **DUSP14** | | 0.32 | 3.21 | 9.E-04 | 49 x 1 | | NA | dual specificity phosphatase 14 [Source:HGNC Symbol;Acc:HGNC:17007] |
| **EXOG** | | 0.32 | 3.20 | 9.E-04 | 47 x 3 | | 3 p22 | endo/exonuclease (5'-3'), endonuclease G-like [Source:HGNC Symbol;Acc:HGNC:3347] |
| **MPP5** | | 0.32 | 3.20 | 1.E-03 | 50 x 1 | | 14 q23 | membrane protein, palmitoylated 5 (MAGUK p55 subfamily member 5) [Source:HGNC Symbol;Acc:HGNC:18669] |
| **FGD4** | | 0.32 | 3.19 | 1.E-03 | 50 x 7 | | 12 p11 | FYVE, RhoGEF and PH domain containing 4 [Source:HGNC Symbol;Acc:HGNC:19125] |
| **PRKAA2** | | 0.32 | 3.18 | 1.E-03 | 45 x 6 | | 1 p32 | protein kinase, AMP-activated, alpha 2 catalytic subunit [Source:HGNC Symbol;Acc:HGNC:9377] |
| **NCKIPSD** | | 0.32 | 3.18 | 0.001 | 45 x 1 | | 3 p21 | NCK interacting protein with SH3 domain [Source:HGNC Symbol;Acc:HGNC:15486] |
| **BDP1** | | 0.32 | 3.18 | 0.001 | 44 x 5 | | NA | B double prime 1, subunit of RNA polymerase III transcription initiation factor IIIB [Source:HGNC Symbol;Acc:HGNC:13652] |
| **ZBTB25** | | 0.32 | 3.18 | 0.001 | 46 x 4 | | 14 q23 | zinc finger and BTB domain containing 25 [Source:HGNC Symbol;Acc:HGNC:13112] |
| **CDH19** | | 0.32 | 3.17 | 0.001 | 50 x 1 | | 18 q22 | cadherin 19, type 2 [Source:HGNC Symbol;Acc:HGNC:1758] |
| **EFNA1** | | 0.32 | 3.17 | 0.001 | 50 x 1 | | 1 q22 | ephrin-A1 [Source:HGNC Symbol;Acc:HGNC:3221] |
| **LASP1** | | 0.32 | 3.17 | 0.001 | 44 x 1 | | 17 q12 | LIM and SH3 protein 1 [Source:HGNC Symbol;Acc:HGNC:6513] |
| **RNF7** | | 0.32 | 3.16 | 0.001 | 47 x 6 | | 3 q23 | ring finger protein 7 [Source:HGNC Symbol;Acc:HGNC:10070] |
| **PVRL3** | | 0.32 | 3.15 | 0.001 | 46 x 1 | | 3 q13 | poliovirus receptor-related 3 [Source:HGNC Symbol;Acc:HGNC:17664] |
| **HDAC5** | | 0.32 | 3.15 | 0.001 | 42 x 1 | | 17 q21 | histone deacetylase 5 [Source:HGNC Symbol;Acc:HGNC:14068] |
| **ZNF160** | | 0.31 | 3.15 | 0.001 | 46 x 2 | | 19 q13 | zinc finger protein 160 [Source:HGNC Symbol;Acc:HGNC:12948] |
| **TUFT1** | | 0.31 | 3.14 | 0.001 | 49 x 6 | | 1 q21 | tuftelin 1 [Source:HGNC Symbol;Acc:HGNC:12422] |
| **PPP1R14B** | | 0.31 | 3.13 | 0.001 | 44 x 5 | | 11 q13 | protein phosphatase 1, regulatory (inhibitor) subunit 14B [Source:HGNC Symbol;Acc:HGNC:9057] |
| **ITFG1** | | 0.31 | 3.12 | 0.001 | 50 x 4 | | 16 q12 | integrin alpha FG-GAP repeat containing 1 [Source:HGNC Symbol;Acc:HGNC:30697] |
| **KDM4B** | | 0.31 | 3.11 | 0.001 | 43 x 1 | | 19 p13 | lysine (K)-specific demethylase 4B [Source:HGNC Symbol;Acc:HGNC:29136] |
| **KIAA0922** | | 0.31 | 3.11 | 0.001 | 49 x 1 | | 4 q31 | KIAA0922 [Source:HGNC Symbol;Acc:HGNC:29146] |
| **SLAIN2** | | 0.31 | 3.11 | 0.001 | 50 x 4 | | 4 p11 | SLAIN motif family, member 2 [Source:HGNC Symbol;Acc:HGNC:29282] |
| **LEAP2** | | 0.31 | 3.10 | 0.001 | 47 x 6 | | 5 q31 | liver expressed antimicrobial peptide 2 [Source:HGNC Symbol;Acc:HGNC:29571] |
| **LIMA1** | | 0.31 | 3.10 | 0.001 | 49 x 1 | | 12 q13 | LIM domain and actin binding 1 [Source:HGNC Symbol;Acc:HGNC:24636] |
| **WWC2** | | 0.31 | 3.09 | 0.001 | 44 x 3 | | 4 q35 | WW and C2 domain containing 2 [Source:HGNC Symbol;Acc:HGNC:24148] |
| **ZNF227** | | 0.31 | 3.09 | 0.001 | 50 x 6 | | 19 q13 | zinc finger protein 227 [Source:HGNC Symbol;Acc:HGNC:13020] |
| **STARD13** | | 0.31 | 3.09 | 0.001 | 44 x 5 | | 13 q13 | StAR-related lipid transfer (START) domain containing 13 [Source:HGNC Symbol;Acc:HGNC:19164] |
| **RAB31** | | 0.31 | 3.09 | 0.001 | 50 x 3 | | 18 p11 | RAB31, member RAS oncogene family [Source:HGNC Symbol;Acc:HGNC:9771] |
| **XPR1** | | 0.31 | 3.08 | 0.001 | 44 x 1 | | 1 q25 | xenotropic and polytropic retrovirus receptor 1 [Source:HGNC Symbol;Acc:HGNC:12827] |
| **STK36** | | 0.31 | 3.08 | 0.001 | 50 x 3 | | 2 q35 | serine/threonine kinase 36 [Source:HGNC Symbol;Acc:HGNC:17209] |
| **TANC1** | | 0.31 | 3.08 | 0.001 | 50 x 5 | | 2 q24 | tetratricopeptide repeat, ankyrin repeat and coiled-coil containing 1 [Source:HGNC Symbol;Acc:HGNC:29364] |
| **FAM177A1** | | 0.31 | 3.08 | 0.001 | 45 x 1 | | 14 q13 | family with sequence similarity 177, member A1 [Source:HGNC Symbol;Acc:HGNC:19829] |
| **NOV** | | 0.31 | 3.08 | 0.001 | 50 x 1 | | 8 q24 | nephroblastoma overexpressed [Source:HGNC Symbol;Acc:HGNC:7885] |
| **C16orf72** | | 0.31 | 3.07 | 0.001 | 45 x 1 | | 16 p13 | chromosome 16 open reading frame 72 [Source:HGNC Symbol;Acc:HGNC:30103] |
| **PINK1** | | 0.31 | 3.07 | 0.001 | 50 x 7 | | 1 p36 | PTEN induced putative kinase 1 [Source:HGNC Symbol;Acc:HGNC:14581] |
| **BCL11A** | | 0.31 | 3.06 | 0.001 | 47 x 1 | | 2 p16 | B-cell CLL/lymphoma 11A (zinc finger protein) [Source:HGNC Symbol;Acc:HGNC:13221] |
| **CCNL2** | | 0.31 | 3.05 | 0.001 | 45 x 4 | | 1 p36 | cyclin L2 [Source:HGNC Symbol;Acc:HGNC:20570] |
| **UGGT2** | | 0.31 | 3.05 | 0.002 | 48 x 3 | | 13 q32 | UDP-glucose glycoprotein glucosyltransferase 2 [Source:HGNC Symbol;Acc:HGNC:15664] |
| **FERMT2** | | 0.31 | 3.05 | 0.002 | 48 x 2 | | 14 q22 | fermitin family member 2 [Source:HGNC Symbol;Acc:HGNC:15767] |
| **TUBB2A** | | 0.31 | 3.05 | 0.002 | 49 x 6 | | 6 p25 | tubulin, beta 2A class IIa [Source:HGNC Symbol;Acc:HGNC:12412] |
| **HSPG2** | | 0.30 | 3.03 | 0.002 | 44 x 5 | | 1 p36 | heparan sulfate proteoglycan 2 [Source:HGNC Symbol;Acc:HGNC:5273] |
| **CD55** | | 0.30 | 3.03 | 0.002 | 50 x 1 | | 1 q32 | CD55 molecule, decay accelerating factor for complement (Cromer blood group) [Source:HGNC Symbol;Acc:HGNC:2665] |
| **DYNC1LI1** | | 0.30 | 3.02 | 0.002 | 47 x 1 | | 3 p22 | dynein, cytoplasmic 1, light intermediate chain 1 [Source:HGNC Symbol;Acc:HGNC:18745] |
| **ESYT1** | | 0.30 | 3.02 | 0.002 | 50 x 5 | | 12 q13 | extended synaptotagmin-like protein 1 [Source:HGNC Symbol;Acc:HGNC:29534] |
| **TRMT10B** | | 0.30 | 3.01 | 0.002 | 45 x 5 | | 9 p13 | tRNA methyltransferase 10 homolog B (S. cerevisiae) [Source:HGNC Symbol;Acc:HGNC:26454] |
| **FRS2** | | 0.30 | 3.01 | 0.002 | 50 x 5 | | 12 q15 | fibroblast growth factor receptor substrate 2 [Source:HGNC Symbol;Acc:HGNC:16971] |
| **EFNA5** | | 0.30 | 3.01 | 0.002 | 48 x 1 | | 5 q21 | ephrin-A5 [Source:HGNC Symbol;Acc:HGNC:3225] |
| **SERPINH1** | | 0.30 | 3.00 | 0.002 | 42 x 1 | | 11 q13 | serpin peptidase inhibitor, clade H (heat shock protein 47), member 1, (collagen binding protein 1) [Source:HGNC Symbol;Acc:HGNC:1546] |
| **LRRFIP2** | | 0.30 | 3.00 | 0.002 | 50 x 4 | | 3 p22 | leucine rich repeat (in FLII) interacting protein 2 [Source:HGNC Symbol;Acc:HGNC:6703] |
| **CHPF** | | 0.30 | 3.00 | 0.002 | 46 x 7 | | 2 q35 | chondroitin polymerizing factor [Source:HGNC Symbol;Acc:HGNC:24291] |
| **SMURF2** | | 0.30 | 2.99 | 0.002 | 43 x 1 | | 17 q24 | SMAD specific E3 ubiquitin protein ligase 2 [Source:HGNC Symbol;Acc:HGNC:16809] |
| **TSC22D1** | | 0.30 | 2.98 | 0.002 | 45 x 1 | | 13 q14 | TSC22 domain family, member 1 [Source:HGNC Symbol;Acc:HGNC:16826] |
| **TATDN1** | | 0.30 | 2.96 | 0.002 | 46 x 1 | | 8 q24 | TatD DNase domain containing 1 [Source:HGNC Symbol;Acc:HGNC:24220] |
| **CCDC112** | | 0.30 | 2.96 | 0.002 | 45 x 1 | | 5 q22 | coiled-coil domain containing 112 [Source:HGNC Symbol;Acc:HGNC:28599] |
| **LMCD1** | | 0.30 | 2.96 | 0.002 | 47 x 1 | | 3 p25 | LIM and cysteine-rich domains 1 [Source:HGNC Symbol;Acc:HGNC:6633] |
| **SCAMP4** | | 0.30 | 2.95 | 0.002 | 43 x 4 | | 19 p13 | secretory carrier membrane protein 4 [Source:HGNC Symbol;Acc:HGNC:30385] |
| **ITFG3** | | 0.30 | 2.94 | 0.002 | 44 x 3 | | 16 p13 | integrin alpha FG-GAP repeat containing 3 [Source:HGNC Symbol;Acc:HGNC:14163] |
| **DPCD** | | 0.30 | 2.94 | 0.002 | 45 x 4 | | 10 q24 | deleted in primary ciliary dyskinesia homolog (mouse) [Source:HGNC Symbol;Acc:HGNC:24542] |
| **NHSL1** | | 0.30 | 2.94 | 0.002 | 44 x 5 | | 6 q24 | NHS-like 1 [Source:HGNC Symbol;Acc:HGNC:21021] |
| **HIST1H1C** | | 0.30 | 2.94 | 0.002 | 48 x 4 | | 6 p22 | histone cluster 1, H1c [Source:HGNC Symbol;Acc:HGNC:4716] |
| **EFHC1** | | 0.30 | 2.94 | 0.002 | 46 x 1 | | 6 p12 | EF-hand domain (C-terminal) containing 1 [Source:HGNC Symbol;Acc:HGNC:16406] |
| **LATS1** | | 0.30 | 2.93 | 0.002 | 44 x 4 | | 6 q25 | large tumor suppressor kinase 1 [Source:HGNC Symbol;Acc:HGNC:6514] |
| **REV3L** | | 0.30 | 2.93 | 0.002 | 50 x 4 | | 6 q21 | REV3-like, polymerase (DNA directed), zeta, catalytic subunit [Source:HGNC Symbol;Acc:HGNC:9968] |
| **ADK** | | 0.29 | 2.92 | 0.002 | 49 x 5 | | 10 q22 | adenosine kinase [Source:HGNC Symbol;Acc:HGNC:257] |
| **NOMO1** | | 0.29 | 2.92 | 0.002 | 43 x 4 | | 16 p13 | NODAL modulator 1 [Source:HGNC Symbol;Acc:HGNC:30060] |
| **PVRL2** | | 0.29 | 2.92 | 0.002 | 50 x 4 | | 19 q13 | poliovirus receptor-related 2 (herpesvirus entry mediator B) [Source:HGNC Symbol;Acc:HGNC:9707] |
| **MMGT1** | | 0.29 | 2.92 | 0.002 | 50 x 3 | | X q26 | membrane magnesium transporter 1 [Source:HGNC Symbol;Acc:HGNC:28100] |
| **BBX** | | 0.29 | 2.90 | 0.002 | 50 x 7 | | 3 q13 | bobby sox homolog (Drosophila) [Source:HGNC Symbol;Acc:HGNC:14422] |
| **MRPL30** | | 0.29 | 2.90 | 0.002 | 42 x 1 | | 2 q11 | mitochondrial ribosomal protein L30 [Source:HGNC Symbol;Acc:HGNC:14036] |
| **ADRA2A** | | 0.29 | 2.89 | 0.002 | 42 x 1 | | 10 q25 | adrenoceptor alpha 2A [Source:HGNC Symbol;Acc:HGNC:281] |
| **SH3D19** | | 0.29 | 2.89 | 0.002 | 50 x 6 | | 4 q31 | SH3 domain containing 19 [Source:HGNC Symbol;Acc:HGNC:30418] |
| **CREBBP** | | 0.29 | 2.89 | 0.002 | 46 x 7 | | 16 p13 | CREB binding protein [Source:HGNC Symbol;Acc:HGNC:2348] |
| **LAMB2** | | 0.29 | 2.88 | 0.002 | 50 x 6 | | 3 p21 | laminin, beta 2 (laminin S) [Source:HGNC Symbol;Acc:HGNC:6487] |
| **RGS2** | | 0.29 | 2.88 | 0.002 | 49 x 1 | | 1 q31 | regulator of G-protein signaling 2 [Source:HGNC Symbol;Acc:HGNC:9998] |
| **AMMECR1** | | 0.29 | 2.88 | 0.002 | 42 x 1 | | X q23 | Alport syndrome, mental retardation, midface hypoplasia and elliptocytosis chromosomal region gene 1 [Source:HGNC Symbol;Acc:HGNC:467] |
| **CSGALNACT2** | | 0.29 | 2.88 | 0.002 | 45 x 2 | | 10 q11 | chondroitin sulfate N-acetylgalactosaminyltransferase 2 [Source:HGNC Symbol;Acc:HGNC:24292] |
| **GATAD1** | | 0.29 | 2.87 | 0.003 | 44 x 4 | | 7 q21 | GATA zinc finger domain containing 1 [Source:HGNC Symbol;Acc:HGNC:29941] |
| **IP6K3** | | 0.29 | 2.87 | 0.003 | 50 x 7 | | 6 p21 | inositol hexakisphosphate kinase 3 [Source:HGNC Symbol;Acc:HGNC:17269] |
| **DDR2** | | 0.29 | 2.87 | 0.003 | 50 x 4 | | 1 q23 | discoidin domain receptor tyrosine kinase 2 [Source:HGNC Symbol;Acc:HGNC:2731] |
| **LAMC1** | | 0.29 | 2.86 | 0.003 | 50 x 3 | | 1 q25 | laminin, gamma 1 (formerly LAMB2) [Source:HGNC Symbol;Acc:HGNC:6492] |
| **MPRIP** | | 0.29 | 2.86 | 0.003 | 42 x 2 | | 17 p11 | myosin phosphatase Rho interacting protein [Source:HGNC Symbol;Acc:HGNC:30321] |
| **NFIB** | | 0.29 | 2.85 | 0.003 | 50 x 6 | | 9 p22 | nuclear factor I/B [Source:HGNC Symbol;Acc:HGNC:7785] |
| **DCAF6** | | 0.29 | 2.85 | 0.003 | 44 x 4 | | 1 q24 | DDB1 and CUL4 associated factor 6 [Source:HGNC Symbol;Acc:HGNC:30002] |
| **UBE2D4** | | 0.29 | 2.84 | 0.003 | 48 x 1 | | 7 p13 | ubiquitin-conjugating enzyme E2D 4 (putative) [Source:HGNC Symbol;Acc:HGNC:21647] |
| **MSL3** | | 0.29 | 2.84 | 0.003 | 46 x 1 | | X p22 | male-specific lethal 3 homolog (Drosophila) [Source:HGNC Symbol;Acc:HGNC:7370] |
| **CAV2** | | 0.29 | 2.83 | 0.003 | 45 x 1 | | 7 q31 | caveolin 2 [Source:HGNC Symbol;Acc:HGNC:1528] |
| **PRNP** | | 0.28 | 2.82 | 0.003 | 47 x 2 | | 20 p13 | prion protein [Source:HGNC Symbol;Acc:HGNC:9449] |
| **ANKZF1** | | 0.28 | 2.82 | 0.003 | 47 x 1 | | 2 q35 | ankyrin repeat and zinc finger domain containing 1 [Source:HGNC Symbol;Acc:HGNC:25527] |
| **MAFG** | | 0.28 | 2.81 | 0.003 | 42 x 1 | | 17 q25 | v-maf avian musculoaponeurotic fibrosarcoma oncogene homolog G [Source:HGNC Symbol;Acc:HGNC:6781] |
| **NSF** | | 0.28 | 2.81 | 0.003 | 47 x 7 | | NA | N-ethylmaleimide-sensitive factor [Source:HGNC Symbol;Acc:HGNC:8016] |
| **PEAK1** | | 0.28 | 2.81 | 0.003 | 45 x 1 | | 15 q24 | pseudopodium-enriched atypical kinase 1 [Source:HGNC Symbol;Acc:HGNC:29431] |
| **DDAH2** | | 0.28 | 2.81 | 0.003 | 46 x 7 | |  | dimethylarginine dimethylaminohydrolase 2 [Source:HGNC Symbol;Acc:HGNC:2716] |
| **CD276** | | 0.28 | 2.80 | 0.003 | 47 x 1 | | 15 q24 | CD276 molecule [Source:HGNC Symbol;Acc:HGNC:19137] |
| **FLNB** | | 0.28 | 2.80 | 0.003 | 45 x 1 | | 3 p14 | filamin B, beta [Source:HGNC Symbol;Acc:HGNC:3755] |
| **RUNX1** | | 0.28 | 2.80 | 0.003 | 44 x 5 | | 21 q22 | runt-related transcription factor 1 [Source:HGNC Symbol;Acc:HGNC:10471] |
| **TPM1** | | 0.28 | 2.80 | 0.003 | 47 x 1 | | 15 q22 | tropomyosin 1 (alpha) [Source:HGNC Symbol;Acc:HGNC:12010] |
| **FKBP7** | | 0.28 | 2.80 | 0.003 | 50 x 1 | | 2 q31 | FK506 binding protein 7 [Source:HGNC Symbol;Acc:HGNC:3723] |
| **ABCB9** | | 0.28 | 2.79 | 0.003 | 43 x 2 | | 12 q24 | ATP-binding cassette, sub-family B (MDR/TAP), member 9 [Source:HGNC Symbol;Acc:HGNC:50] |
| **TJP1** | | 0.28 | 2.79 | 0.003 | 42 x 1 | | NA | tight junction protein 1 [Source:HGNC Symbol;Acc:HGNC:11827] |
| **CLUAP1** | | 0.28 | 2.79 | 0.003 | 50 x 5 | | 16 p13 | clusterin associated protein 1 [Source:HGNC Symbol;Acc:HGNC:19009] |
| **ACOT9** | | 0.28 | 2.79 | 0.003 | 47 x 1 | | X p22 | acyl-CoA thioesterase 9 [Source:HGNC Symbol;Acc:HGNC:17152] |
| **SPRTN** | | 0.28 | 2.79 | 0.003 | 50 x 4 | | 1 q42 | SprT-like N-terminal domain [Source:HGNC Symbol;Acc:HGNC:25356] |
| **COBLL1** | | 0.28 | 2.78 | 0.003 | 50 x 6 | | 2 q24 | cordon-bleu WH2 repeat protein-like 1 [Source:HGNC Symbol;Acc:HGNC:23571] |
| **HTT** | | 0.28 | 2.78 | 0.003 | 47 x 1 | | 4 p16 | huntingtin [Source:HGNC Symbol;Acc:HGNC:4851] |
| **TUT1** | | 0.28 | 2.78 | 0.003 | 50 x 6 | | 11 q12 | terminal uridylyl transferase 1, U6 snRNA-specific [Source:HGNC Symbol;Acc:HGNC:26184] |
| **AKAP2** | | 0.28 | 2.77 | 0.003 | 42 x 1 | | 9 q31 | A kinase (PRKA) anchor protein 2 [Source:HGNC Symbol;Acc:HGNC:372] |
| **MAFF** | | 0.28 | 2.77 | 0.003 | 43 x 1 | | 22 q13 | v-maf avian musculoaponeurotic fibrosarcoma oncogene homolog F [Source:HGNC Symbol;Acc:HGNC:6780] |
| **NOMO3** | | 0.28 | 2.77 | 0.003 | 43 x 4 | | NA | NODAL modulator 3 [Source:HGNC Symbol;Acc:HGNC:25242] |
| **ASXL1** | | 0.28 | 2.76 | 0.003 | 45 x 4 | | 20 q11 | additional sex combs like transcriptional regulator 1 [Source:HGNC Symbol;Acc:HGNC:18318] |
| **MEF2A** | | 0.28 | 2.75 | 0.004 | 44 x 5 | | 15 q26 | myocyte enhancer factor 2A [Source:HGNC Symbol;Acc:HGNC:6993] |
| **RIC8A** | | 0.28 | 2.75 | 0.004 | 48 x 6 | | 11 p15 | RIC8 guanine nucleotide exchange factor A [Source:HGNC Symbol;Acc:HGNC:29550] |
| **FPGT** | | 0.28 | 2.74 | 0.004 | 50 x 6 | | 1 p31 | fucose-1-phosphate guanylyltransferase [Source:HGNC Symbol;Acc:HGNC:3825] |
| **HBP1** | | 0.28 | 2.73 | 0.004 | 50 x 4 | | 7 q22 | HMG-box transcription factor 1 [Source:HGNC Symbol;Acc:HGNC:23200] |
| **TMBIM1** | | 0.28 | 2.72 | 0.004 | 46 x 1 | | 2 q35 | transmembrane BAX inhibitor motif containing 1 [Source:HGNC Symbol;Acc:HGNC:23410] |
| **DNAJC16** | | 0.27 | 2.71 | 0.004 | 50 x 5 | | 1 p36 | DnaJ (Hsp40) homolog, subfamily C, member 16 [Source:HGNC Symbol;Acc:HGNC:29157] |
| **MT2A** | | 0.27 | 2.71 | 0.004 | 50 x 3 | | 16 q13 | metallothionein 2A [Source:HGNC Symbol;Acc:HGNC:7406] |
| **CSRP2** | | 0.27 | 2.71 | 0.004 | 48 x 1 | | 12 q21 | cysteine and glycine-rich protein 2 [Source:HGNC Symbol;Acc:HGNC:2470] |
| **SNX25** | | 0.27 | 2.70 | 0.004 | 47 x 6 | | 4 q35 | sorting nexin 25 [Source:HGNC Symbol;Acc:HGNC:21883] |
| **GRAMD3** | | 0.27 | 2.70 | 0.004 | 45 x 3 | | 5 q23 | GRAM domain containing 3 [Source:HGNC Symbol;Acc:HGNC:24911] |
| **TRIM16** | | 0.27 | 2.70 | 0.004 | 50 x 5 | | 17 p12 | tripartite motif containing 16 [Source:HGNC Symbol;Acc:HGNC:17241] |
| **EXT2** | | 0.27 | 2.70 | 0.004 | 47 x 1 | | 11 p11 | exostosin glycosyltransferase 2 [Source:HGNC Symbol;Acc:HGNC:3513] |
| **MCAM** | | 0.27 | 2.70 | 0.004 | 46 x 1 | | 11 q23 | melanoma cell adhesion molecule [Source:HGNC Symbol;Acc:HGNC:6934] |
| **RAPGEF2** | | 0.27 | 2.70 | 0.004 | 50 x 4 | | 4 q32 | Rap guanine nucleotide exchange factor (GEF) 2 [Source:HGNC Symbol;Acc:HGNC:16854] |
| **FNDC3B** | | 0.27 | 2.70 | 0.004 | 47 x 4 | | 3 q26 | fibronectin type III domain containing 3B [Source:HGNC Symbol;Acc:HGNC:24670] |
| **ZNF562** | | 0.27 | 2.70 | 0.004 | 46 x 6 | | 19 p13 | zinc finger protein 562 [Source:HGNC Symbol;Acc:HGNC:25950] |
| **PITX2** | | 0.27 | 2.70 | 0.004 | 50 x 4 | | 4 q25 | paired-like homeodomain 2 [Source:HGNC Symbol;Acc:HGNC:9005] |
| **VPS39** | | 0.27 | 2.69 | 0.004 | 42 x 1 | | 15 q15 | vacuolar protein sorting 39 homolog (S. cerevisiae) [Source:HGNC Symbol;Acc:HGNC:20593] |
| **LGALS1** | | 0.27 | 2.68 | 0.004 | 50 x 1 | | 22 q13 | lectin, galactoside-binding, soluble, 1 [Source:HGNC Symbol;Acc:HGNC:6561] |
| **SHC1** | | 0.27 | 2.68 | 0.004 | 50 x 5 | | 1 q21 | SHC (Src homology 2 domain containing) transforming protein 1 [Source:HGNC Symbol;Acc:HGNC:10840] |
| **SCRN3** | | 0.27 | 2.67 | 0.004 | 50 x 5 | | 2 q31 | secernin 3 [Source:HGNC Symbol;Acc:HGNC:30382] |
| **RRAS2** | | 0.27 | 2.67 | 0.004 | 44 x 1 | | 11 p15 | related RAS viral (r-ras) oncogene homolog 2 [Source:HGNC Symbol;Acc:HGNC:17271] |
| **ITGB5** | | 0.27 | 2.67 | 0.004 | 49 x 3 | | 3 q21 | integrin, beta 5 [Source:HGNC Symbol;Acc:HGNC:6160] |
| **GPR22** | | 0.27 | 2.67 | 0.005 | 42 x 1 | | 7 q22 | G protein-coupled receptor 22 [Source:HGNC Symbol;Acc:HGNC:4477] |
| **PPM1A** | | 0.27 | 2.66 | 0.005 | 48 x 6 | | 14 q23 | protein phosphatase, Mg2+/Mn2+ dependent, 1A [Source:HGNC Symbol;Acc:HGNC:9275] |
| **TMEM179B** | | 0.27 | 2.66 | 0.005 | 49 x 7 | | 11 q12 | transmembrane protein 179B [Source:HGNC Symbol;Acc:HGNC:33744] |
| **NOMO2** | | 0.27 | 2.65 | 0.005 | 43 x 4 | | 16 p12 | NODAL modulator 2 [Source:HGNC Symbol;Acc:HGNC:22652] |
| **PYCR2** | | 0.27 | 2.65 | 0.005 | 42 x 1 | | 1 q42 | pyrroline-5-carboxylate reductase family, member 2 [Source:HGNC Symbol;Acc:HGNC:30262] |
| **TLN1** | | 0.27 | 2.65 | 0.005 | 50 x 7 | | 9 p13 | talin 1 [Source:HGNC Symbol;Acc:HGNC:11845] |
| **DBN1** | | 0.27 | 2.65 | 0.005 | 45 x 1 | | 5 q35 | drebrin 1 [Source:HGNC Symbol;Acc:HGNC:2695] |
| **KCNE4** | | 0.27 | 2.65 | 0.005 | 50 x 7 | | 2 q36 | potassium channel, voltage gated subfamily E regulatory beta subunit 4 [Source:HGNC Symbol;Acc:HGNC:6244] |
| **ALKBH3** | | 0.27 | 2.64 | 0.005 | 44 x 1 | | 11 p11 | alkB, alkylation repair homolog 3 (E. coli) [Source:HGNC Symbol;Acc:HGNC:30141] |
| **IFT20** | | 0.27 | 2.63 | 0.005 | 50 x 6 | | 17 q11 | intraflagellar transport 20 [Source:HGNC Symbol;Acc:HGNC:30989] |
| **C9orf156** | | 0.27 | 2.61 | 0.005 | 45 x 1 | | 9 q22 | chromosome 9 open reading frame 156 [Source:HGNC Symbol;Acc:HGNC:30967] |
| **LEPROT** | | 0.27 | 2.61 | 0.005 | 49 x 7 | | 1 p31 | leptin receptor overlapping transcript [Source:HGNC Symbol;Acc:HGNC:29477] |
| **RAI14** | | 0.27 | 2.61 | 0.005 | 44 x 1 | | 5 p13 | retinoic acid induced 14 [Source:HGNC Symbol;Acc:HGNC:14873] |
| **NR3C1** | | 0.26 | 2.60 | 0.005 | 49 x 5 | | 5 q31 | nuclear receptor subfamily 3, group C, member 1 (glucocorticoid receptor) [Source:HGNC Symbol;Acc:HGNC:7978] |
| **ALDH3A2** | | 0.26 | 2.60 | 0.005 | 50 x 5 | | 17 p11 | aldehyde dehydrogenase 3 family, member A2 [Source:HGNC Symbol;Acc:HGNC:403] |
| **SYF2** | | 0.26 | 2.59 | 0.006 | 45 x 5 | | 1 p36 | SYF2 pre-mRNA-splicing factor [Source:HGNC Symbol;Acc:HGNC:19824] |
| **PAWR** | | 0.26 | 2.58 | 0.006 | 46 x 4 | | 12 q21 | PRKC, apoptosis, WT1, regulator [Source:HGNC Symbol;Acc:HGNC:8614] |
| **CSF1** | | 0.26 | 2.58 | 0.006 | 42 x 1 | | 1 p13 | colony stimulating factor 1 (macrophage) [Source:HGNC Symbol;Acc:HGNC:2432] |
| **OXSR1** | | 0.26 | 2.57 | 0.006 | 46 x 1 | | 3 p22 | oxidative stress responsive 1 [Source:HGNC Symbol;Acc:HGNC:8508] |
| **APOOL** | | 0.26 | 2.57 | 0.006 | 42 x 1 | | X q21 | apolipoprotein O-like [Source:HGNC Symbol;Acc:HGNC:24009] |
| **AASS** | | 0.26 | 2.56 | 0.006 | 50 x 3 | | 7 q31 | aminoadipate-semialdehyde synthase [Source:HGNC Symbol;Acc:HGNC:17366] |
| **NEK6** | | 0.26 | 2.55 | 0.006 | 50 x 4 | | 9 q33 | NIMA-related kinase 6 [Source:HGNC Symbol;Acc:HGNC:7749] |
| **LRPAP1** | | 0.26 | 2.55 | 0.006 | 42 x 1 | | 4 p16 | low density lipoprotein receptor-related protein associated protein 1 [Source:HGNC Symbol;Acc:HGNC:6701] |
| **DOCK5** | | 0.26 | 2.55 | 0.006 | 45 x 3 | | 8 p21 | dedicator of cytokinesis 5 [Source:HGNC Symbol;Acc:HGNC:23476] |
| **PCYOX1** | | 0.26 | 2.55 | 0.006 | 49 x 4 | | 2 p13 | prenylcysteine oxidase 1 [Source:HGNC Symbol;Acc:HGNC:20588] |
| **IFT122** | | 0.26 | 2.55 | 0.006 | 50 x 5 | | 3 q21 | intraflagellar transport 122 [Source:HGNC Symbol;Acc:HGNC:13556] |
| **TMEM67** | | 0.26 | 2.55 | 0.006 | 50 x 1 | | 8 q22 | transmembrane protein 67 [Source:HGNC Symbol;Acc:HGNC:28396] |
| **TBC1D13** | | 0.26 | 2.54 | 0.006 | 44 x 1 | | 9 q34 | TBC1 domain family, member 13 [Source:HGNC Symbol;Acc:HGNC:25571] |
| **ZNF839** | | 0.26 | 2.52 | 0.007 | 44 x 1 | | 14 q32 | zinc finger protein 839 [Source:HGNC Symbol;Acc:HGNC:20345] |
| **PLEKHA3** | | 0.26 | 2.52 | 0.007 | 43 x 1 | | 2 q31 | pleckstrin homology domain containing, family A (phosphoinositide binding specific) member 3 [Source:HGNC Symbol;Acc:HGNC:14338] |
| **TMEM43** | | 0.26 | 2.52 | 0.007 | 43 x 3 | | 3 p25 | transmembrane protein 43 [Source:HGNC Symbol;Acc:HGNC:28472] |
| **SGTB** | | 0.26 | 2.51 | 0.007 | 46 x 4 | | 5 q12 | small glutamine-rich tetratricopeptide repeat (TPR)-containing, beta [Source:HGNC Symbol;Acc:HGNC:23567] |
| **RNF13** | | 0.26 | 2.51 | 0.007 | 50 x 7 | | 3 q25 | ring finger protein 13 [Source:HGNC Symbol;Acc:HGNC:10057] |
| **BBS2** | | 0.26 | 2.51 | 0.007 | 50 x 6 | | 16 q13 | Bardet-Biedl syndrome 2 [Source:HGNC Symbol;Acc:HGNC:967] |
| **RNF141** | | 0.26 | 2.51 | 0.007 | 50 x 5 | | 11 p15 | ring finger protein 141 [Source:HGNC Symbol;Acc:HGNC:21159] |
| **METTL8** | | 0.26 | 2.51 | 0.007 | 45 x 1 | | 2 q31 | methyltransferase like 8 [Source:HGNC Symbol;Acc:HGNC:25856] |
| **NBR1** | | 0.25 | 2.50 | 0.007 | 44 x 1 | | 17 q21 | neighbor of BRCA1 gene 1 [Source:HGNC Symbol;Acc:HGNC:6746] |
| **PIAS3** | | 0.25 | 2.50 | 0.007 | 47 x 7 | | 1 q21 | protein inhibitor of activated STAT, 3 [Source:HGNC Symbol;Acc:HGNC:16861] |
| **ZYG11B** | | 0.25 | 2.50 | 0.007 | 43 x 3 | | 1 p32 | zyg-11 family member B, cell cycle regulator [Source:HGNC Symbol;Acc:HGNC:25820] |
| **CEP162** | | 0.25 | 2.49 | 0.007 | 50 x 6 | | 6 q14 | centrosomal protein 162kDa [Source:HGNC Symbol;Acc:HGNC:21107] |
| **ZNFX1** | | 0.25 | 2.48 | 0.007 | 50 x 7 | | 20 q13 | zinc finger, NFX1-type containing 1 [Source:HGNC Symbol;Acc:HGNC:29271] |
| **PLOD1** | | 0.25 | 2.48 | 0.007 | 44 x 1 | | 1 p36 | procollagen-lysine, 2-oxoglutarate 5-dioxygenase 1 [Source:HGNC Symbol;Acc:HGNC:9081] |
| **SLFN5** | | 0.25 | 2.48 | 0.008 | 50 x 7 | | 17 q12 | schlafen family member 5 [Source:HGNC Symbol;Acc:HGNC:28286] |
| **CFL2** | | 0.25 | 2.47 | 0.008 | 50 x 7 | | 14 q13 | cofilin 2 (muscle) [Source:HGNC Symbol;Acc:HGNC:1875] |
| **MAP4K4** | | 0.25 | 2.47 | 0.008 | 50 x 5 | | 2 q11 | mitogen-activated protein kinase kinase kinase kinase 4 [Source:HGNC Symbol;Acc:HGNC:6866] |
| **UBE2R2** | | 0.25 | 2.47 | 0.008 | 43 x 3 | | 9 p13 | ubiquitin-conjugating enzyme E2R 2 [Source:HGNC Symbol;Acc:HGNC:19907] |
| **STAM** | | 0.25 | 2.46 | 0.008 | 50 x 3 | | 10 p12 | signal transducing adaptor molecule (SH3 domain and ITAM motif) 1 [Source:HGNC Symbol;Acc:HGNC:11357] |
| **AKAP13** | | 0.25 | 2.45 | 0.008 | 50 x 6 | | 15 q25 | A kinase (PRKA) anchor protein 13 [Source:HGNC Symbol;Acc:HGNC:371] |
| **RAD50** | | 0.25 | 2.45 | 0.008 | 45 x 6 | | 5 q31 | RAD50 homolog (S. cerevisiae) [Source:HGNC Symbol;Acc:HGNC:9816] |
| **PHLDB1** | | 0.25 | 2.44 | 0.008 | 44 x 1 | | 11 q23 | pleckstrin homology-like domain, family B, member 1 [Source:HGNC Symbol;Acc:HGNC:23697] |
| **MYO9A** | | 0.25 | 2.44 | 0.008 | 45 x 1 | | 15 q23 | myosin IXA [Source:HGNC Symbol;Acc:HGNC:7608] |
| **RBMS3** | | 0.25 | 2.42 | 0.009 | 50 x 6 | | 3 p24 | RNA binding motif, single stranded interacting protein 3 [Source:HGNC Symbol;Acc:HGNC:13427] |
| **OSGIN1** | | 0.25 | 2.42 | 0.009 | 50 x 6 | | 16 q23 | oxidative stress induced growth inhibitor 1 [Source:HGNC Symbol;Acc:HGNC:30093] |
| **P2RX4** | | 0.25 | 2.40 | 0.009 | 43 x 1 | | 12 q24 | purinergic receptor P2X, ligand gated ion channel, 4 [Source:HGNC Symbol;Acc:HGNC:8535] |
| **FAM8A1** | | 0.24 | 2.39 | 0.009 | 46 x 5 | | 6 p22 | family with sequence similarity 8, member A1 [Source:HGNC Symbol;Acc:HGNC:16372] |
| **B4GALT4** | | 0.24 | 2.39 | 0.009 | 50 x 4 | | 3 q13 | UDP-Gal:betaGlcNAc beta 1,4- galactosyltransferase, polypeptide 4 [Source:HGNC Symbol;Acc:HGNC:927] |
| **TM9SF4** | | 0.24 | 2.39 | 0.010 | 44 x 5 | | 20 q11 | transmembrane 9 superfamily protein member 4 [Source:HGNC Symbol;Acc:HGNC:30797] |
| **COTL1** | | 0.24 | 2.38 | 0.010 | 47 x 1 | | 16 q24 | coactosin-like F-actin binding protein 1 [Source:HGNC Symbol;Acc:HGNC:18304] |
| **AKAP9** | | 0.24 | 2.38 | 0.010 | 42 x 1 | | 7 q21 | A kinase (PRKA) anchor protein 9 [Source:HGNC Symbol;Acc:HGNC:379] |
| **CENPT** | | 0.24 | 2.37 | 0.010 | 50 x 5 | | 16 q22 | centromere protein T [Source:HGNC Symbol;Acc:HGNC:25787] |
| **FAM98A** | | 0.24 | 2.37 | 0.010 | 49 x 1 | | 2 p22 | family with sequence similarity 98, member A [Source:HGNC Symbol;Acc:HGNC:24520] |
| **AIFM2** | | 0.24 | 2.36 | 0.010 | 44 x 3 | | 10 q22 | apoptosis-inducing factor, mitochondrion-associated, 2 [Source:HGNC Symbol;Acc:HGNC:21411] |
| **DCAF8** | | 0.24 | 2.34 | 0.011 | 50 x 7 | | 1 q23 | DDB1 and CUL4 associated factor 8 [Source:HGNC Symbol;Acc:HGNC:24891] |
| **MRPL10** | | 0.24 | 2.34 | 0.011 | 50 x 7 | | 17 q21 | mitochondrial ribosomal protein L10 [Source:HGNC Symbol;Acc:HGNC:14055] |
| **DOLK** | | 0.24 | 2.32 | 0.011 | 46 x 1 | | 9 q34 | dolichol kinase [Source:HGNC Symbol;Acc:HGNC:23406] |
| **SLC35F5** | | 0.24 | 2.30 | 0.012 | 42 x 1 | | 2 q14 | solute carrier family 35, member F5 [Source:HGNC Symbol;Acc:HGNC:23617] |
| **LUM** | | 0.24 | 2.30 | 0.012 | 49 x 7 | | 12 q21 | lumican [Source:HGNC Symbol;Acc:HGNC:6724] |
| **ITSN1** | | 0.23 | 2.29 | 0.012 | 46 x 6 | | 21 q22 | intersectin 1 (SH3 domain protein) [Source:HGNC Symbol;Acc:HGNC:6183] |
| **FYN** | | 0.23 | 2.28 | 0.013 | 49 x 7 | | 6 q21 | FYN proto-oncogene, Src family tyrosine kinase [Source:HGNC Symbol;Acc:HGNC:4037] |
| **ACTR1A** | | 0.23 | 2.28 | 0.013 | 42 x 1 | | 10 q24 | ARP1 actin-related protein 1 homolog A, centractin alpha (yeast) [Source:HGNC Symbol;Acc:HGNC:167] |
| **FAM104A** | | 0.23 | 2.27 | 0.013 | 50 x 6 | | 17 q25 | family with sequence similarity 104, member A [Source:HGNC Symbol;Acc:HGNC:25918] |
| **ZCRB1** | | 0.23 | 2.27 | 0.013 | 50 x 6 | | 12 q12 | zinc finger CCHC-type and RNA binding motif 1 [Source:HGNC Symbol;Acc:HGNC:29620] |
| **HIVEP1** | | 0.23 | 2.27 | 0.013 | 42 x 1 | | 6 p24 | human immunodeficiency virus type I enhancer binding protein 1 [Source:HGNC Symbol;Acc:HGNC:4920] |
| **TCTN1** | | 0.23 | 2.27 | 0.013 | 47 x 3 | | 12 q24 | tectonic family member 1 [Source:HGNC Symbol;Acc:HGNC:26113] |
| **SUGT1** | | 0.23 | 2.24 | 0.014 | 43 x 2 | | 13 q14 | SGT1, suppressor of G2 allele of SKP1 (S. cerevisiae) [Source:HGNC Symbol;Acc:HGNC:16987] |
| **ADAMTS1** | | 0.23 | 2.24 | 0.014 | 44 x 1 | | 21 q21 | ADAM metallopeptidase with thrombospondin type 1 motif, 1 [Source:HGNC Symbol;Acc:HGNC:217] |
| **SLC16A6** | | 0.23 | 2.22 | 0.015 | 44 x 1 | | 17 q24 | solute carrier family 16, member 6 [Source:HGNC Symbol;Acc:HGNC:10927] |
| **ITM2C** | | 0.23 | 2.21 | 0.015 | 42 x 3 | | 2 q37 | integral membrane protein 2C [Source:HGNC Symbol;Acc:HGNC:6175] |
| **TM2D1** | | 0.23 | 2.21 | 0.015 | 42 x 1 | | 1 p31 | TM2 domain containing 1 [Source:HGNC Symbol;Acc:HGNC:24142] |
| **THOC5** | | 0.22 | 2.19 | 0.016 | 46 x 1 | | 22 q12 | THO complex 5 [Source:HGNC Symbol;Acc:HGNC:19074] |
| **PEX11A** | | 0.22 | 2.18 | 0.016 | 50 x 6 | | 15 q26 | peroxisomal biogenesis factor 11 alpha [Source:HGNC Symbol;Acc:HGNC:8852] |
| **LATS2** | | 0.22 | 2.18 | 0.016 | 44 x 1 | | 13 q12 | large tumor suppressor kinase 2 [Source:HGNC Symbol;Acc:HGNC:6515] |
| **NRP1** | | 0.22 | 2.18 | 0.016 | 50 x 5 | | 10 p11 | neuropilin 1 [Source:HGNC Symbol;Acc:HGNC:8004] |
| **PKP4** | | 0.22 | 2.17 | 0.016 | 50 x 7 | | 2 q24 | plakophilin 4 [Source:HGNC Symbol;Acc:HGNC:9026] |
| **C9orf85** | | 0.22 | 2.17 | 0.016 | 43 x 3 | | 9 q21 | chromosome 9 open reading frame 85 [Source:HGNC Symbol;Acc:HGNC:28784] |
| **RFTN1** | | 0.22 | 2.17 | 0.016 | 43 x 1 | | 3 p24 | raftlin, lipid raft linker 1 [Source:HGNC Symbol;Acc:HGNC:30278] |
| **ASF1A** | | 0.22 | 2.16 | 0.017 | 50 x 7 | | 6 q22 | anti-silencing function 1A histone chaperone [Source:HGNC Symbol;Acc:HGNC:20995] |
| **RNF41** | | 0.22 | 2.16 | 0.017 | 42 x 1 | | 12 q13 | ring finger protein 41, E3 ubiquitin protein ligase [Source:HGNC Symbol;Acc:HGNC:18401] |
| **AFF4** | | 0.22 | 2.15 | 0.017 | 50 x 6 | | 5 q31 | AF4/FMR2 family, member 4 [Source:HGNC Symbol;Acc:HGNC:17869] |
| **YIPF1** | | 0.22 | 2.15 | 0.017 | 50 x 5 | | 1 p32 | Yip1 domain family, member 1 [Source:HGNC Symbol;Acc:HGNC:25231] |
| **GLS** | | 0.22 | 2.15 | 0.017 | 46 x 7 | | 2 q32 | glutaminase [Source:HGNC Symbol;Acc:HGNC:4331] |
| **OSER1** | | 0.22 | 2.14 | 0.018 | 45 x 6 | | 20 q13 | oxidative stress responsive serine-rich 1 [Source:HGNC Symbol;Acc:HGNC:16105] |
| **SLC41A1** | | 0.22 | 2.11 | 0.019 | 49 x 7 | | 1 q32 | solute carrier family 41 (magnesium transporter), member 1 [Source:HGNC Symbol;Acc:HGNC:19429] |
| **IGF1R** | | 0.22 | 2.09 | 0.020 | 50 x 7 | | 15 q26 | insulin-like growth factor 1 receptor [Source:HGNC Symbol;Acc:HGNC:5465] |
| **CWC27** | | 0.22 | 2.09 | 0.020 | 43 x 1 | | 5 q12 | CWC27 spliceosome-associated protein homolog (S. cerevisiae) [Source:HGNC Symbol;Acc:HGNC:10664] |
| **LCAT** | | 0.21 | 2.09 | 0.020 | 46 x 4 | | 16 q22 | lecithin-cholesterol acyltransferase [Source:HGNC Symbol;Acc:HGNC:6522] |
| **RNASEH1** | | 0.21 | 2.08 | 0.020 | 44 x 4 | | 2 p25 | ribonuclease H1 [Source:HGNC Symbol;Acc:HGNC:18466] |
| **ST5** | | 0.21 | 2.08 | 0.020 | 50 x 5 | | 11 p15 | suppression of tumorigenicity 5 [Source:HGNC Symbol;Acc:HGNC:11350] |
| **GJC1** | | 0.21 | 2.07 | 0.021 | 46 x 1 | | 17 q21 | gap junction protein, gamma 1, 45kDa [Source:HGNC Symbol;Acc:HGNC:4280] |
| **EFCAB14** | | 0.21 | 2.07 | 0.021 | 50 x 7 | | 1 p33 | EF-hand calcium binding domain 14 [Source:HGNC Symbol;Acc:HGNC:29051] |
| **RSRP1** | | 0.21 | 2.06 | 0.021 | 50 x 4 | | 1 p36 | arginine/serine-rich protein 1 [Source:HGNC Symbol;Acc:HGNC:25234] |
| **PPP4R1** | | 0.21 | 2.05 | 0.022 | 50 x 7 | | 18 p11 | protein phosphatase 4, regulatory subunit 1 [Source:HGNC Symbol;Acc:HGNC:9320] |
| **PTPN12** | | 0.21 | 2.05 | 0.022 | 46 x 1 | | 7 q11 | protein tyrosine phosphatase, non-receptor type 12 [Source:HGNC Symbol;Acc:HGNC:9645] |
| **SMARCA1** | | 0.21 | 2.05 | 0.022 | 50 x 6 | | X q26 | SWI/SNF related, matrix associated, actin dependent regulator of chromatin, subfamily a, member 1 [Source:HGNC Symbol;Acc:HGNC:11097] |
| **CRNKL1** | | 0.21 | 2.04 | 0.022 | 44 x 3 | | 20 p11 | crooked neck pre-mRNA splicing factor 1 [Source:HGNC Symbol;Acc:HGNC:15762] |
| **HIST1H2BK** | | 0.21 | 2.01 | 0.024 | 46 x 7 | | 6 p22 | histone cluster 1, H2bk [Source:HGNC Symbol;Acc:HGNC:13954] |
| **NOL10** | | 0.21 | 2.00 | 0.024 | 42 x 1 | | 2 p25 | nucleolar protein 10 [Source:HGNC Symbol;Acc:HGNC:25862] |
| **TIPARP** | | 0.20 | 1.98 | 0.026 | 46 x 5 | | 3 q25 | TCDD-inducible poly(ADP-ribose) polymerase [Source:HGNC Symbol;Acc:HGNC:23696] |
| **ARG2** | | 0.20 | 1.97 | 0.026 | 50 x 7 | | 14 q24 | arginase 2 [Source:HGNC Symbol;Acc:HGNC:664] |
| **RTCA** | | 0.20 | 1.97 | 0.026 | 42 x 3 | | 1 p21 | RNA 3'-terminal phosphate cyclase [Source:HGNC Symbol;Acc:HGNC:17981] |
| **POLR3GL** | | 0.20 | 1.93 | 0.028 | 50 x 6 | | 1 q21 | polymerase (RNA) III (DNA directed) polypeptide G (32kD)-like [Source:HGNC Symbol;Acc:HGNC:28466] |
| **ZNF468** | | 0.20 | 1.93 | 0.028 | 42 x 3 | | 19 q13 | zinc finger protein 468 [Source:HGNC Symbol;Acc:HGNC:33105] |
| **GNAI1** | | 0.20 | 1.91 | 0.030 | 42 x 1 | | 7 q21 | guanine nucleotide binding protein (G protein), alpha inhibiting activity polypeptide 1 [Source:HGNC Symbol;Acc:HGNC:4384] |
| **ANKRD13C** | | 0.20 | 1.90 | 0.030 | 43 x 2 | | 1 p31 | ankyrin repeat domain 13C [Source:HGNC Symbol;Acc:HGNC:25374] |
| **TM7SF3** | | 0.19 | 1.88 | 0.032 | 50 x 5 | | 12 p11 | transmembrane 7 superfamily member 3 [Source:HGNC Symbol;Acc:HGNC:23049] |
| **LRRC8A** | | 0.19 | 1.87 | 0.033 | 45 x 1 | | 9 q34 | leucine rich repeat containing 8 family, member A [Source:HGNC Symbol;Acc:HGNC:19027] |
| **WDR25** | | 0.19 | 1.86 | 0.033 | 43 x 3 | | 14 q32 | WD repeat domain 25 [Source:HGNC Symbol;Acc:HGNC:21064] |
| **TMEM165** | | 0.19 | 1.85 | 0.034 | 45 x 1 | | 4 q12 | transmembrane protein 165 [Source:HGNC Symbol;Acc:HGNC:30760] |
| **MFN1** | | 0.19 | 1.84 | 0.034 | 42 x 1 | | 3 q26 | mitofusin 1 [Source:HGNC Symbol;Acc:HGNC:18262] |
| **ARL4C** | | 0.19 | 1.84 | 0.034 | 43 x 1 | | 2 q37 | ADP-ribosylation factor-like 4C [Source:HGNC Symbol;Acc:HGNC:698] |
| **BCR** | | 0.18 | 1.78 | 0.039 | 42 x 1 | | 22 q11 | breakpoint cluster region [Source:HGNC Symbol;Acc:HGNC:1014] |
| **ORC3** | | 0.18 | 1.75 | 0.042 | 50 x 7 | | 6 q15 | origin recognition complex, subunit 3 [Source:HGNC Symbol;Acc:HGNC:8489] |
| **ABCC10** | | 0.18 | 1.72 | 0.045 | 43 x 1 | | 6 p21 | ATP-binding cassette, sub-family C (CFTR/MRP), member 10 [Source:HGNC Symbol;Acc:HGNC:52] |
| **CCDC86** | | 0.18 | 1.69 | 0.047 | 42 x 1 | | 11 q12 | coiled-coil domain containing 86 [Source:HGNC Symbol;Acc:HGNC:28359] |
| **ZCCHC17** | | 0.17 | 1.66 | 0.051 | 46 x 7 | | 1 p35 | zinc finger, CCHC domain containing 17 [Source:HGNC Symbol;Acc:HGNC:30246] |
| **AKR1A1** | | 0.17 | 1.62 | 0.054 | 50 x 7 | | 1 p34 | aldo-keto reductase family 1, member A1 (aldehyde reductase) [Source:HGNC Symbol;Acc:HGNC:380] |
| **BAZ2A** | | 0.16 | 1.58 | 0.059 | 46 x 7 | | 12 q13 | bromodomain adjacent to zinc finger domain, 2A [Source:HGNC Symbol;Acc:HGNC:962] |
| **ATP2B4** | | 0.16 | 1.57 | 0.060 | 50 x 7 | | 1 q32 | ATPase, Ca++ transporting, plasma membrane 4 [Source:HGNC Symbol;Acc:HGNC:817] |
| **SSSCA1** | | 0.16 | 1.50 | 0.068 | 50 x 7 | | 11 q13 | Sjogren syndrome/scleroderma autoantigen 1 [Source:HGNC Symbol;Acc:HGNC:11328] |
| **DDB2** | | 0.16 | 1.50 | 0.069 | 50 x 7 | | 11 p11 | damage-specific DNA binding protein 2, 48kDa [Source:HGNC Symbol;Acc:HGNC:2718] |
| **CUL2** | | 0.15 | 1.42 | 0.079 | 42 x 1 | | 10 p11 | cullin 2 [Source:HGNC Symbol;Acc:HGNC:2552] |
| **PABPC4** | | 0.14 | 1.38 | 0.086 | 50 x 7 | | 1 p34 | poly(A) binding protein, cytoplasmic 4 (inducible form) [Source:HGNC Symbol;Acc:HGNC:8557] |
| **TMX3** | | 0.14 | 1.33 | 0.093 | 44 x 1 | | 18 q22 | thioredoxin-related transmembrane protein 3 [Source:HGNC Symbol;Acc:HGNC:24718] |
| **FAM76A** | | 0.12 | 1.19 | 0.118 | 50 x 7 | | 1 p35 | family with sequence similarity 76, member A [Source:HGNC Symbol;Acc:HGNC:28530] |
| **S100A13** | | 0.11 | 1.08 | 0.140 | 50 x 7 | | 1 q21 | S100 calcium binding protein A13 [Source:HGNC Symbol;Acc:HGNC:10490] |

^1^correlation to spot profile; ^2^ x- and y-coordinates of gene position in SOM;
