# Supplementary material for: Mapping heterogeneity in patient-derived melanoma cultures by single-cell RNA-seq
Source: Oncotarget. 2016 Nov 26;8(1):846–62. doi: 10.18632/oncotarget.13666 (PMC5352202; doi:10.18632/oncotarget.13666)
Supplement: Supplementary file 3 [file oncotarget-08-846-s003.docx]

**Supplementary Table 2. Complete list of genes of spot E: Gene name, statistics, position in SOM, and description.**

| **Genes of spot E** | | | | | | |
| --- | --- | --- | --- | --- | --- | --- |
| **Symbol** | **Correlation ^1^** | **->t.score** | **->p.value** | **Metagene ^2^** | **Chromosome** | **Description** |
| ABCC9 | 0,95 | 28,14 | 0 | 28 x 4 | 12 p12 | ATP-binding cassette, sub-family C (CFTR/MRP), member 9 [Source:HGNC Symbol;Acc:HGNC:60] |
| ZNF90 | 0,95 | 27,69 | 0 | 28 x 3 | 19 p12 | zinc finger protein 90 [Source:HGNC Symbol;Acc:HGNC:13165] |
| LRRD1 | 0,95 | 27,49 | 0 | 28 x 4 | 7 q21 | leucine-rich repeats and death domain containing 1 [Source:HGNC Symbol;Acc:HGNC:34300] |
| ABCA1 | 0,94 | 27,04 | 0 | 28 x 4 | 9 q31 | ATP-binding cassette, sub-family A (ABC1), member 1 [Source:HGNC Symbol;Acc:HGNC:29] |
| ABCG1 | 0,94 | 26,84 | 0 | 28 x 4 | 21 q22 | ATP-binding cassette, sub-family G (WHITE), member 1 [Source:HGNC Symbol;Acc:HGNC:73] |
| AC104532.2 | 0,94 | 26,84 | 0 | 28 x 4 | NA |  |
| ABHD12B | 0,94 | 26,53 | 0 | 28 x 4 | 14 q22 | abhydrolase domain containing 12B [Source:HGNC Symbol;Acc:HGNC:19837] |
| AC025263.3 | 0,94 | 26,39 | 0 | 28 x 4 | NA |  |
| ACE | 0,94 | 26,13 | 0 | 28 x 4 | 17 q23 | angiotensin I converting enzyme [Source:HGNC Symbol;Acc:HGNC:2707] |
| ACOT4 | 0,94 | 25,88 | 0 | 28 x 4 | 14 q24 | acyl-CoA thioesterase 4 [Source:HGNC Symbol;Acc:HGNC:19748] |
| ACTG2 | 0,94 | 25,66 | 0 | 28 x 4 | 2 p13 | actin, gamma 2, smooth muscle, enteric [Source:HGNC Symbol;Acc:HGNC:145] |
| AD000671.6 | 0,94 | 25,59 | 0 | 28 x 4 | NA |  |
| AC006486.9 | 0,94 | 25,56 | 0 | 28 x 4 | NA |  |
| AC011530.4 | 0,94 | 25,52 | 0 | 28 x 4 | NA |  |
| ACSM2A | 0,94 | 25,46 | 0 | 28 x 4 | 16 p12 | acyl-CoA synthetase medium-chain family member 2A [Source:HGNC Symbol;Acc:HGNC:32017] |
| ADCY5 | 0,94 | 25,33 | 0 | 28 x 5 | 3 q21 | adenylate cyclase 5 [Source:HGNC Symbol;Acc:HGNC:236] |
| ABCG2 | 0,94 | 25,27 | 0 | 28 x 4 | 4 q22 | ATP-binding cassette, sub-family G (WHITE), member 2 (Junior blood group) [Source:HGNC Symbol;Acc:HGNC:74] |
| ADAMTSL2 | 0,94 | 25,25 | 0 | 28 x 5 | 9 q34 | ADAMTS-like 2 [Source:HGNC Symbol;Acc:HGNC:14631] |
| ADCY4 | 0,94 | 25,17 | 0 | 28 x 5 | 14 q12 | adenylate cyclase 4 [Source:HGNC Symbol;Acc:HGNC:235] |
| ABLIM2 | 0,94 | 25,14 | 0 | 28 x 4 | 4 p16 | actin binding LIM protein family, member 2 [Source:HGNC Symbol;Acc:HGNC:19195] |
| ADAP1 | 0,94 | 25,13 | 0 | 28 x 5 | 7 p22 | ArfGAP with dual PH domains 1 [Source:HGNC Symbol;Acc:HGNC:16486] |
| ADM2 | 0,93 | 24,81 | 0 | 28 x 5 | 22 q13 | adrenomedullin 2 [Source:HGNC Symbol;Acc:HGNC:28898] |
| ACSL6 | 0,93 | 24,78 | 0 | 28 x 3 | 5 q31 | acyl-CoA synthetase long-chain family member 6 [Source:HGNC Symbol;Acc:HGNC:16496] |
| ADAMTS6 | 0,93 | 24,78 | 0 | 28 x 5 | 5 q12 | ADAM metallopeptidase with thrombospondin type 1 motif, 6 [Source:HGNC Symbol;Acc:HGNC:222] |
| ADAMTSL1 | 0,93 | 24,59 | 0 | 28 x 5 | 9 p22 | ADAMTS-like 1 [Source:HGNC Symbol;Acc:HGNC:14632] |
| ACAP1 | 0,93 | 24,55 | 0 | 28 x 4 | 17 p13 | ArfGAP with coiled-coil, ankyrin repeat and PH domains 1 [Source:HGNC Symbol;Acc:HGNC:16467] |
| AGTR1 | 0,93 | 24,37 | 0 | 28 x 5 | 3 q24 | angiotensin II receptor, type 1 [Source:HGNC Symbol;Acc:HGNC:336] |
| ACOT11 | 0,93 | 24,32 | 0 | 28 x 4 | 1 p32 | acyl-CoA thioesterase 11 [Source:HGNC Symbol;Acc:HGNC:18156] |
| AGXT | 0,93 | 24,17 | 0 | 28 x 5 | 2 q37 | alanine-glyoxylate aminotransferase [Source:HGNC Symbol;Acc:HGNC:341] |
| AICDA | 0,93 | 24,13 | 0 | 28 x 5 | 12 p13 | activation-induced cytidine deaminase [Source:HGNC Symbol;Acc:HGNC:13203] |
| ACOXL | 0,93 | 24,11 | 0 | 28 x 4 | 2 q13 | acyl-CoA oxidase-like [Source:HGNC Symbol;Acc:HGNC:25621] |
| ALDH1A3 | 0,93 | 24,04 | 0 | 28 x 5 | 15 q26 | aldehyde dehydrogenase 1 family, member A3 [Source:HGNC Symbol;Acc:HGNC:409] |
| ADRA1A | 0,93 | 23,99 | 0 | 28 x 5 | 8 p21 | adrenoceptor alpha 1A [Source:HGNC Symbol;Acc:HGNC:277] |
| ADAMTS10 | 0,93 | 23,84 | 0 | 28 x 5 | 19 p13 | ADAM metallopeptidase with thrombospondin type 1 motif, 10 [Source:HGNC Symbol;Acc:HGNC:13201] |
| ADARB2 | 0,93 | 23,75 | 0 | 28 x 5 | 10 p15 | adenosine deaminase, RNA-specific, B2 (non-functional) [Source:HGNC Symbol;Acc:HGNC:227] |
| ACSM2B | 0,93 | 23,71 | 0 | 28 x 4 | 16 p12 | acyl-CoA synthetase medium-chain family member 2B [Source:HGNC Symbol;Acc:HGNC:30931] |
| AIPL1 | 0,93 | 23,62 | 0 | 28 x 5 | 17 p13 | aryl hydrocarbon receptor interacting protein-like 1 [Source:HGNC Symbol;Acc:HGNC:359] |
| AMICA1 | 0,93 | 23,60 | 0 | 28 x 5 | 11 q23 | adhesion molecule, interacts with CXADR antigen 1 [Source:HGNC Symbol;Acc:HGNC:19084] |
| ALPK2 | 0,93 | 23,59 | 0 | 28 x 5 | 18 q21 | alpha-kinase 2 [Source:HGNC Symbol;Acc:HGNC:20565] |
| ALDH2 | 0,93 | 23,59 | 0 | 28 x 5 | 12 q24 | aldehyde dehydrogenase 2 family (mitochondrial) [Source:HGNC Symbol;Acc:HGNC:404] |
| ABCA6 | 0,93 | 23,58 | 0 | 28 x 4 | 17 q24 | ATP-binding cassette, sub-family A (ABC1), member 6 [Source:HGNC Symbol;Acc:HGNC:36] |
| AMER2 | 0,93 | 23,48 | 0 | 28 x 5 | 13 q12 | APC membrane recruitment protein 2 [Source:HGNC Symbol;Acc:HGNC:26360] |
| ADIRF | 0,93 | 23,40 | 0 | 28 x 5 | 10 q23 | adipogenesis regulatory factor [Source:HGNC Symbol;Acc:HGNC:24043] |
| ALPP | 0,93 | 23,32 | 0 | 28 x 5 | 2 q37 | alkaline phosphatase, placental [Source:HGNC Symbol;Acc:HGNC:439] |
| ANGPT4 | 0,93 | 23,22 | 0 | 28 x 5 | 20 p13 | angiopoietin 4 [Source:HGNC Symbol;Acc:HGNC:487] |
| RGPD1 | 0,92 | 23,07 | 0 | 27 x 1 | 2 p11 | RANBP2-like and GRIP domain containing 1 [Source:HGNC Symbol;Acc:HGNC:32414] |
| ABCC3 | 0,92 | 22,98 | 0 | 28 x 4 | 17 q21 | ATP-binding cassette, sub-family C (CFTR/MRP), member 3 [Source:HGNC Symbol;Acc:HGNC:54] |
| ACMSD | 0,92 | 22,97 | 0 | 28 x 4 | 2 q21 | aminocarboxymuconate semialdehyde decarboxylase [Source:HGNC Symbol;Acc:HGNC:19288] |
| ANKRD2 | 0,92 | 22,79 | 0 | 28 x 5 | 10 q24 | ankyrin repeat domain 2 (stretch responsive muscle) [Source:HGNC Symbol;Acc:HGNC:495] |
| A1CF | 0,92 | 22,76 | 0 | 28 x 4 | 10 q11 | APOBEC1 complementation factor [Source:HGNC Symbol;Acc:HGNC:24086] |
| AMN | 0,92 | 22,74 | 0 | 28 x 5 | 14 q32 | amnion associated transmembrane protein [Source:HGNC Symbol;Acc:HGNC:14604] |
| ADAMTS8 | 0,92 | 22,72 | 0 | 28 x 5 | 11 q24 | ADAM metallopeptidase with thrombospondin type 1 motif, 8 [Source:HGNC Symbol;Acc:HGNC:224] |
| ACTA1 | 0,92 | 22,69 | 0 | 28 x 5 | 1 q42 | actin, alpha 1, skeletal muscle [Source:HGNC Symbol;Acc:HGNC:129] |
| ANKRD35 | 0,92 | 22,68 | 0 | 28 x 5 | 1 q21 | ankyrin repeat domain 35 [Source:HGNC Symbol;Acc:HGNC:26323] |
| ANO3 | 0,92 | 22,48 | 0 | 28 x 5 | 11 p14 | anoctamin 3 [Source:HGNC Symbol;Acc:HGNC:14004] |
| ADORA1 | 0,92 | 22,36 | 0 | 28 x 5 | 1 q32 | adenosine A1 receptor [Source:HGNC Symbol;Acc:HGNC:262] |
| ABHD16B | 0,92 | 22,29 | 0 | 28 x 4 | 20 q13 | abhydrolase domain containing 16B [Source:HGNC Symbol;Acc:HGNC:16128] |
| ANKFN1 | 0,92 | 22,14 | 0 | 28 x 5 | 17 q22 | ankyrin-repeat and fibronectin type III domain containing 1 [Source:HGNC Symbol;Acc:HGNC:26766] |
| ANKRD34C | 0,92 | 22,10 | 0 | 28 x 5 | 15 q25 | ankyrin repeat domain 34C [Source:HGNC Symbol;Acc:HGNC:33888] |
| ANKRD30BL | 0,92 | 22,01 | 0 | 28 x 5 | 2 q21 | ankyrin repeat domain 30B-like [Source:HGNC Symbol;Acc:HGNC:35167] |
| ALPL | 0,92 | 21,93 | 0 | 28 x 5 | 1 p36 | alkaline phosphatase, liver/bone/kidney [Source:HGNC Symbol;Acc:HGNC:438] |
| ABHD1 | 0,92 | 21,80 | 0 | 28 x 4 | 2 p23 | abhydrolase domain containing 1 [Source:HGNC Symbol;Acc:HGNC:17553] |
| ABCC11 | 0,92 | 21,76 | 0 | 28 x 4 | 16 q12 | ATP-binding cassette, sub-family C (CFTR/MRP), member 11 [Source:HGNC Symbol;Acc:HGNC:14639] |
| AF165138.7 | 0,92 | 21,56 | 0 | 28 x 5 | NA |  |
| AKAP14 | 0,91 | 21,40 | 0 | 28 x 5 | X q24 | A kinase (PRKA) anchor protein 14 [Source:HGNC Symbol;Acc:HGNC:24061] |
| ADH4 | 0,91 | 21,30 | 0 | 28 x 5 | 4 q23 | alcohol dehydrogenase 4 (class II), pi polypeptide [Source:HGNC Symbol;Acc:HGNC:252] |
| AFF2 | 0,91 | 21,17 | 0 | 28 x 5 | X q28 | AF4/FMR2 family, member 2 [Source:HGNC Symbol;Acc:HGNC:3776] |
| ABTB2 | 0,91 | 20,94 | 0 | 28 x 4 | 11 p13 | ankyrin repeat and BTB (POZ) domain containing 2 [Source:HGNC Symbol;Acc:HGNC:23842] |
| ANKRD24 | 0,91 | 20,84 | 0 | 28 x 5 | 19 p13 | ankyrin repeat domain 24 [Source:HGNC Symbol;Acc:HGNC:29424] |
| AGXT2 | 0,91 | 20,79 | 0 | 28 x 5 | 5 p13 | alanine--glyoxylate aminotransferase 2 [Source:HGNC Symbol;Acc:HGNC:14412] |
| ALDH8A1 | 0,91 | 20,37 | 0 | 28 x 5 | 6 q23 | aldehyde dehydrogenase 8 family, member A1 [Source:HGNC Symbol;Acc:HGNC:15471] |
| ABCC6 | 0,91 | 20,35 | 0 | 28 x 4 | 16 p13 | ATP-binding cassette, sub-family C (CFTR/MRP), member 6 [Source:HGNC Symbol;Acc:HGNC:57] |
| ADRA1D | 0,91 | 20,31 | 0 | 28 x 4 | 20 p13 | adrenoceptor alpha 1D [Source:HGNC Symbol;Acc:HGNC:280] |
| PPIAL4G | 0,90 | 20,09 | 0 | 28 x 1 | 1 q21 | peptidylprolyl isomerase A (cyclophilin A)-like 4G [Source:HGNC Symbol;Acc:HGNC:33996] |
| AMIGO2 | 0,90 | 19,59 | 0 | 28 x 5 | 12 q13 | adhesion molecule with Ig-like domain 2 [Source:HGNC Symbol;Acc:HGNC:24073] |
| ALDH1L2 | 0,90 | 19,15 | 0 | 28 x 5 | 12 q23 | aldehyde dehydrogenase 1 family, member L2 [Source:HGNC Symbol;Acc:HGNC:26777] |
| RGPD4 | 0,90 | 19,10 | 0 | 27 x 1 | 2 q12 | RANBP2-like and GRIP domain containing 4 [Source:HGNC Symbol;Acc:HGNC:32417] |
| ANKS4B | 0,89 | 19,01 | 0 | 28 x 5 | 16 p12 | ankyrin repeat and sterile alpha motif domain containing 4B [Source:HGNC Symbol;Acc:HGNC:26795] |
| ADAT3 | 0,89 | 18,79 | 0 | 28 x 4 | 19 p13 | adenosine deaminase, tRNA-specific 3 [Source:HGNC Symbol;Acc:HGNC:25151] |
| ARL2-SNX15 | 0,89 | 18,49 | 0 | 27 x 4 | 11 q13 | ARL2-SNX15 readthrough (NMD candidate) [Source:HGNC Symbol;Acc:HGNC:49197] |
| APOL1 | 0,89 | 18,33 | 0 | 28 x 5 | 22 q12 | apolipoprotein L, 1 [Source:HGNC Symbol;Acc:HGNC:618] |
| ABI3BP | 0,89 | 18,20 | 0 | 28 x 4 | 3 q12 | ABI family, member 3 (NESH) binding protein [Source:HGNC Symbol;Acc:HGNC:17265] |
| ADCY2 | 0,88 | 17,74 | 0 | 26 x 3 | 5 p15 | adenylate cyclase 2 (brain) [Source:HGNC Symbol;Acc:HGNC:233] |
| RGPD3 | 0,88 | 17,56 | 0 | 27 x 1 | 2 q12 | RANBP2-like and GRIP domain containing 3 [Source:HGNC Symbol;Acc:HGNC:32416] |
| ADAMTS14 | 0,88 | 17,51 | 0 | 28 x 5 | 10 q22 | ADAM metallopeptidase with thrombospondin type 1 motif, 14 [Source:HGNC Symbol;Acc:HGNC:14899] |
| ADAMTS19 | 0,87 | 17,03 | 0 | 28 x 4 | 5 q23 | ADAM metallopeptidase with thrombospondin type 1 motif, 19 [Source:HGNC Symbol;Acc:HGNC:17111] |
| ANKLE1 | 0,87 | 16,95 | 0 | 28 x 5 | 19 p13 | ankyrin repeat and LEM domain containing 1 [Source:HGNC Symbol;Acc:HGNC:26812] |
| ADM | 0,87 | 16,87 | 0 | 28 x 4 | 11 p15 | adrenomedullin [Source:HGNC Symbol;Acc:HGNC:259] |
| AC002985.3 | 0,87 | 16,83 | 0 | 28 x 4 | NA |  |
| ABCA13 | 0,87 | 16,73 | 0 | 28 x 4 | 7 p12 | ATP-binding cassette, sub-family A (ABC1), member 13 [Source:HGNC Symbol;Acc:HGNC:14638] |
| ACBD7 | 0,87 | 16,73 | 0 | 28 x 4 | 10 p13 | acyl-CoA binding domain containing 7 [Source:HGNC Symbol;Acc:HGNC:17715] |
| ADAMTS20 | 0,87 | 16,55 | 0 | 28 x 5 | 12 q12 | ADAM metallopeptidase with thrombospondin type 1 motif, 20 [Source:HGNC Symbol;Acc:HGNC:17178] |
| ADAM32 | 0,86 | 16,34 | 0 | 28 x 4 | NA | ADAM metallopeptidase domain 32 [Source:HGNC Symbol;Acc:HGNC:15479] |
| ABCA3 | 0,86 | 16,29 | 0 | 28 x 5 | 16 p13 | ATP-binding cassette, sub-family A (ABC1), member 3 [Source:HGNC Symbol;Acc:HGNC:33] |
| ABHD8 | 0,86 | 16,11 | 0 | 28 x 4 | 19 p13 | abhydrolase domain containing 8 [Source:HGNC Symbol;Acc:HGNC:23759] |
| AF196779.12 | 0,86 | 16,03 | 0 | 28 x 5 | NA |  |
| AKR1B15 | 0,86 | 15,93 | 0 | 28 x 5 | 7 q33 | aldo-keto reductase family 1, member B15 [Source:HGNC Symbol;Acc:HGNC:37281] |
| AGBL1 | 0,86 | 15,90 | 0 | 28 x 5 | 15 q25 | ATP/GTP binding protein-like 1 [Source:HGNC Symbol;Acc:HGNC:26504] |
| ACVR1C | 0,85 | 15,58 | 0 | 28 x 5 | 2 q24 | activin A receptor, type IC [Source:HGNC Symbol;Acc:HGNC:18123] |
| ACKR3 | 0,85 | 15,38 | 0 | 28 x 5 | 2 q37 | atypical chemokine receptor 3 [Source:HGNC Symbol;Acc:HGNC:23692] |
| AOC2 | 0,85 | 15,24 | 0 | 28 x 5 | 17 q21 | amine oxidase, copper containing 2 (retina-specific) [Source:HGNC Symbol;Acc:HGNC:549] |
| ACADL | 0,85 | 15,24 | 0 | 28 x 4 | 2 q34 | acyl-CoA dehydrogenase, long chain [Source:HGNC Symbol;Acc:HGNC:88] |
| ANKRD18B | 0,84 | 14,72 | 0 | 28 x 5 | 9 p13 | ankyrin repeat domain 18B [Source:HGNC Symbol;Acc:HGNC:23644] |
| AKR1C4 | 0,84 | 14,56 | 0 | 28 x 2 | 10 p15 | aldo-keto reductase family 1, member C4 [Source:HGNC Symbol;Acc:HGNC:387] |
| C4orf32 | 0,83 | 13,94 | 0 | 29 x 2 | 4 q25 | chromosome 4 open reading frame 32 [Source:HGNC Symbol;Acc:HGNC:26813] |
| ACHE | 0,82 | 13,60 | 0 | 27 x 4 | 7 q22 | acetylcholinesterase (Yt blood group) [Source:HGNC Symbol;Acc:HGNC:108] |
| ADAMTS2 | 0,82 | 13,50 | 0 | 28 x 5 | 5 q35 | ADAM metallopeptidase with thrombospondin type 1 motif, 2 [Source:HGNC Symbol;Acc:HGNC:218] |
| ACKR4 | 0,82 | 13,50 | 0 | 28 x 4 | 3 q22 | atypical chemokine receptor 4 [Source:HGNC Symbol;Acc:HGNC:1611] |
| RGPD2 | 0,81 | 13,32 | 0 | 28 x 1 | 2 p11 | RANBP2-like and GRIP domain containing 2 [Source:HGNC Symbol;Acc:HGNC:32415] |
| ANO8 | 0,81 | 13,25 | 0 | 28 x 5 | 19 p13 | anoctamin 8 [Source:HGNC Symbol;Acc:HGNC:29329] |
| ANKRD20A3 | 0,81 | 13,23 | 0 | 27 x 4 | 9 q21 | ankyrin repeat domain 20 family, member A3 [Source:HGNC Symbol;Acc:HGNC:31981] |
| POTEI | 0,81 | 13,21 | 0 | 28 x 1 | 2 q21 | POTE ankyrin domain family, member I [Source:HGNC Symbol;Acc:HGNC:37093] |
| ANO1 | 0,81 | 13,12 | 0 | 26 x 5 | 11 q13 | anoctamin 1, calcium activated chloride channel [Source:HGNC Symbol;Acc:HGNC:21625] |
| ABHD17A | 0,81 | 13,06 | 0 | 29 x 3 | 19 p13 | abhydrolase domain containing 17A [Source:HGNC Symbol;Acc:HGNC:28756] |
| NBPF8 | 0,80 | 12,85 | 0 | 27 x 1 | 1 q21 | neuroblastoma breakpoint family, member 8 [Source:HGNC Symbol;Acc:HGNC:31990] |
| ACAN | 0,80 | 12,82 | 0 | 28 x 4 | 15 q26 | aggrecan [Source:HGNC Symbol;Acc:HGNC:319] |
| ACSBG1 | 0,80 | 12,81 | 0 | 26 x 2 | 15 q25 | acyl-CoA synthetase bubblegum family member 1 [Source:HGNC Symbol;Acc:HGNC:29567] |
| AC003002.6 | 0,80 | 12,75 | 0 | 28 x 4 | NA |  |
| AATK | 0,80 | 12,68 | 0 | 28 x 4 | 17 q25 | apoptosis-associated tyrosine kinase [Source:HGNC Symbol;Acc:HGNC:21] |
| FSIP2 | 0,80 | 12,66 | 0 | 26 x 2 | 2 q32 | fibrous sheath interacting protein 2 [Source:HGNC Symbol;Acc:HGNC:21675] |
| ALG1L | 0,80 | 12,50 | 0 | 29 x 4 | 3 q21 | ALG1, chitobiosyldiphosphodolichol beta-mannosyltransferase-like [Source:HGNC Symbol;Acc:HGNC:33721] |
| AANAT | 0,79 | 12,40 | 0 | 28 x 4 | 17 q25 | aralkylamine N-acetyltransferase [Source:HGNC Symbol;Acc:HGNC:19] |
| C12orf50 | 0,79 | 12,36 | 0 | 28 x 4 | 12 q21 | chromosome 12 open reading frame 50 [Source:HGNC Symbol;Acc:HGNC:26665] |
| AMY1C | 0,79 | 12,24 | 0 | 25 x 5 | 1 p21 | amylase, alpha 1C (salivary) [Source:HGNC Symbol;Acc:HGNC:476] |
| AIFM3 | 0,79 | 12,23 | 0 | 29 x 4 | 22 q11 | apoptosis-inducing factor, mitochondrion-associated, 3 [Source:HGNC Symbol;Acc:HGNC:26398] |
| GIGYF1 | 0,79 | 12,23 | 0 | 27 x 1 | 7 q22 | GRB10 interacting GYF protein 1 [Source:HGNC Symbol;Acc:HGNC:9126] |
| ADAMTS4 | 0,79 | 12,13 | 0 | 29 x 4 | 1 q23 | ADAM metallopeptidase with thrombospondin type 1 motif, 4 [Source:HGNC Symbol;Acc:HGNC:220] |
| CCL5 | 0,79 | 12,11 | 0 | 27 x 3 | NA | chemokine (C-C motif) ligand 5 [Source:HGNC Symbol;Acc:HGNC:10632] |
| AC007040.11 | 0,78 | 11,94 | 0 | 28 x 4 | NA |  |
| ANGPTL5 | 0,78 | 11,87 | 0 | 27 x 5 | 11 q22 | angiopoietin-like 5 [Source:HGNC Symbol;Acc:HGNC:19705] |
| ALX3 | 0,78 | 11,75 | 0 | 27 x 5 | 1 p13 | ALX homeobox 3 [Source:HGNC Symbol;Acc:HGNC:449] |
| ANGPTL4 | 0,77 | 11,60 | 0 | 28 x 5 | 19 p13 | angiopoietin-like 4 [Source:HGNC Symbol;Acc:HGNC:16039] |
| AC069368.3 | 0,77 | 11,55 | 0 | 28 x 4 | NA |  |
| WASH1 | 0,77 | 11,35 | 0 | 27 x 1 | 9 p24 | WAS protein family homolog 1 [Source:HGNC Symbol;Acc:HGNC:24361] |
| POTEF | 0,76 | 11,26 | 0 | 28 x 1 | 2 q21 | POTE ankyrin domain family, member F [Source:HGNC Symbol;Acc:HGNC:33905] |
| ABCG5 | 0,76 | 11,24 | 0 | 29 x 4 | 2 p21 | ATP-binding cassette, sub-family G (WHITE), member 5 [Source:HGNC Symbol;Acc:HGNC:13886] |
| ALK | 0,76 | 11,21 | 0 | 27 x 5 | 2 p23 | anaplastic lymphoma receptor tyrosine kinase [Source:HGNC Symbol;Acc:HGNC:427] |
| PPIAL4C | 0,76 | 11,07 | 0 | 28 x 1 | 1 q21 | peptidylprolyl isomerase A (cyclophilin A)-like 4C [Source:HGNC Symbol;Acc:HGNC:33995] |
| ADRA2C | 0,76 | 11,02 | 0 | 28 x 5 | 4 p16 | adrenoceptor alpha 2C [Source:HGNC Symbol;Acc:HGNC:283] |
| CYSLTR1 | 0,76 | 10,97 | 0 | 29 x 4 | X q21 | cysteinyl leukotriene receptor 1 [Source:HGNC Symbol;Acc:HGNC:17451] |
| ADORA2A | 0,76 | 10,95 | 0 | 29 x 4 | 22 q11 | adenosine A2a receptor [Source:HGNC Symbol;Acc:HGNC:263] |
| AC006538.4 | 0,75 | 10,76 | 0 | 26 x 3 | NA |  |
| AGER | 0,75 | 10,67 | 0 | 28 x 5 |  | advanced glycosylation end product-specific receptor [Source:HGNC Symbol;Acc:HGNC:320] |
| ADRB3 | 0,74 | 10,51 | 0 | 27 x 5 | 8 p11 | adrenoceptor beta 3 [Source:HGNC Symbol;Acc:HGNC:288] |
| SLC35G2 | 0,74 | 10,45 | 0 | 26 x 3 | 3 q22 | solute carrier family 35, member G2 [Source:HGNC Symbol;Acc:HGNC:28480] |
| ANKRD20A4 | 0,74 | 10,45 | 0 | 28 x 5 | 9 q13 | ankyrin repeat domain 20 family, member A4 [Source:HGNC Symbol;Acc:HGNC:31982] |
| ANP32D | 0,74 | 10,29 | 0 | 25 x 1 | 12 q13 | acidic (leucine-rich) nuclear phosphoprotein 32 family, member D [Source:HGNC Symbol;Acc:HGNC:16676] |
| ABCA2 | 0,73 | 10,26 | 0 | 27 x 3 | 9 q34 | ATP-binding cassette, sub-family A (ABC1), member 2 [Source:HGNC Symbol;Acc:HGNC:32] |
| NPIPA2 | 0,73 | 10,23 | 0 | 28 x 1 | 16 p13 | nuclear pore complex interacting protein family, member A2 [Source:HGNC Symbol;Acc:HGNC:41979] |
| AKR7A3 | 0,73 | 10,23 | 0 | 28 x 3 | 1 p36 | aldo-keto reductase family 7, member A3 (aflatoxin aldehyde reductase) [Source:HGNC Symbol;Acc:HGNC:390] |
| ZNF471 | 0,73 | 10,23 | 0 | 25 x 4 | 19 q13 | zinc finger protein 471 [Source:HGNC Symbol;Acc:HGNC:23226] |
| POTEE | 0,73 | 10,17 | 0 | 29 x 1 | 2 q21 | POTE ankyrin domain family, member E [Source:HGNC Symbol;Acc:HGNC:33895] |
| ANKRD20A1 | 0,73 | 10,06 | 1,E-16 | 26 x 4 | 9 q21 | ankyrin repeat domain 20 family, member A1 [Source:HGNC Symbol;Acc:HGNC:23665] |
| AGAP5 | 0,73 | 10,03 | 1,E-16 | 27 x 4 | 10 q22 | ArfGAP with GTPase domain, ankyrin repeat and PH domain 5 [Source:HGNC Symbol;Acc:HGNC:23467] |
| NBPF10 | 0,72 | 9,91 | 2,E-16 | 28 x 1 | 1 q21 | neuroblastoma breakpoint family, member 10 [Source:HGNC Symbol;Acc:HGNC:31992] |
| COL6A3 | 0,72 | 9,89 | 2,E-16 | 25 x 4 | 2 q37 | collagen, type VI, alpha 3 [Source:HGNC Symbol;Acc:HGNC:2213] |
| AC015688.3 | 0,72 | 9,84 | 3,E-16 | 29 x 4 | NA |  |
| SULT1B1 | 0,72 | 9,76 | 4,E-16 | 29 x 3 | 4 q13 | sulfotransferase family, cytosolic, 1B, member 1 [Source:HGNC Symbol;Acc:HGNC:17845] |
| ANKUB1 | 0,71 | 9,58 | 1,E-15 | 30 x 5 | 3 q25 | ankyrin repeat and ubiquitin domain containing 1 [Source:HGNC Symbol;Acc:HGNC:29642] |
| SEMA3E | 0,71 | 9,54 | 1,E-15 | 28 x 2 | 7 q21 | sema domain, immunoglobulin domain (Ig), short basic domain, secreted, (semaphorin) 3E [Source:HGNC Symbol;Acc:HGNC:10727] |
| ACVRL1 | 0,71 | 9,49 | 2,E-15 | 28 x 4 | 12 q13 | activin A receptor type II-like 1 [Source:HGNC Symbol;Acc:HGNC:175] |
| MANSC1 | 0,71 | 9,44 | 2,E-15 | 25 x 4 | 12 p13 | MANSC domain containing 1 [Source:HGNC Symbol;Acc:HGNC:25505] |
| MEX3D | 0,70 | 9,42 | 2,E-15 | 27 x 4 | 19 p13 | mex-3 RNA binding family member D [Source:HGNC Symbol;Acc:HGNC:16734] |
| A3GALT2 | 0,70 | 9,36 | 3,E-15 | 30 x 4 | 1 p35 | alpha 1,3-galactosyltransferase 2 [Source:HGNC Symbol;Acc:HGNC:30005] |
| FEM1A | 0,70 | 9,30 | 4,E-15 | 28 x 1 | 19 p13 | fem-1 homolog a (C. elegans) [Source:HGNC Symbol;Acc:HGNC:16934] |
| ACSBG2 | 0,70 | 9,24 | 6,E-15 | 27 x 5 | 19 p13 | acyl-CoA synthetase bubblegum family member 2 [Source:HGNC Symbol;Acc:HGNC:24174] |
| DSG3 | 0,70 | 9,22 | 6,E-15 | 25 x 4 | 18 q12 | desmoglein 3 [Source:HGNC Symbol;Acc:HGNC:3050] |
| PPIAL4B | 0,70 | 9,21 | 6,E-15 | 28 x 2 | NA |  |
| ABRA | 0,70 | 9,21 | 6,E-15 | 29 x 5 | 8 q23 | actin-binding Rho activating protein [Source:HGNC Symbol;Acc:HGNC:30655] |
| AC003005.4 | 0,69 | 9,09 | 1,E-14 | 29 x 4 | NA |  |
| AP3B2 | 0,69 | 9,07 | 1,E-14 | 27 x 5 | 15 q25 | adaptor-related protein complex 3, beta 2 subunit [Source:HGNC Symbol;Acc:HGNC:567] |
| SPDYE1 | 0,69 | 9,06 | 1,E-14 | 28 x 3 | 7 p13 | speedy/RINGO cell cycle regulator family member E1 [Source:HGNC Symbol;Acc:HGNC:16408] |
| ETV3L | 0,69 | 9,05 | 1,E-14 | 25 x 1 | 1 q23 | ets variant 3-like [Source:HGNC Symbol;Acc:HGNC:33834] |
| ACTR3C | 0,69 | 9,02 | 2,E-14 | 27 x 5 | 7 q36 | ARP3 actin-related protein 3 homolog C (yeast) [Source:HGNC Symbol;Acc:HGNC:37282] |
| TUBB3 | 0,69 | 9,01 | 2,E-14 | 28 x 1 | 16 q24 | tubulin, beta 3 class III [Source:HGNC Symbol;Acc:HGNC:20772] |
| POTEJ | 0,69 | 8,95 | 2,E-14 | 29 x 1 | 2 q21 | POTE ankyrin domain family, member J [Source:HGNC Symbol;Acc:HGNC:37094] |
| NBPF11 | 0,68 | 8,84 | 4,E-14 | 28 x 1 | 1 q21 | neuroblastoma breakpoint family, member 11 [Source:HGNC Symbol;Acc:HGNC:31993] |
| NBPF12 | 0,68 | 8,80 | 4,E-14 | 28 x 1 | 1 q21 | neuroblastoma breakpoint family, member 12 [Source:HGNC Symbol;Acc:HGNC:24297] |
| MALL | 0,68 | 8,76 | 6,E-14 | 24 x 4 | 2 q13 | mal, T-cell differentiation protein-like [Source:HGNC Symbol;Acc:HGNC:6818] |
| PDE6A | 0,68 | 8,68 | 8,E-14 | 27 x 3 | 5 q32 | phosphodiesterase 6A, cGMP-specific, rod, alpha [Source:HGNC Symbol;Acc:HGNC:8785] |
| ADRB1 | 0,67 | 8,66 | 9,E-14 | 27 x 5 | 10 q25 | adrenoceptor beta 1 [Source:HGNC Symbol;Acc:HGNC:285] |
| AMHR2 | 0,67 | 8,62 | 1,E-13 | 28 x 5 | 12 q13 | anti-Mullerian hormone receptor, type II [Source:HGNC Symbol;Acc:HGNC:465] |
| SSX7 | 0,67 | 8,58 | 1,E-13 | 24 x 2 | X p11 | synovial sarcoma, X breakpoint 7 [Source:HGNC Symbol;Acc:HGNC:19653] |
| ADAM11 | 0,67 | 8,55 | 2,E-13 | 28 x 3 | 17 q21 | ADAM metallopeptidase domain 11 [Source:HGNC Symbol;Acc:HGNC:189] |
| NBPF9 | 0,67 | 8,54 | 2,E-13 | 28 x 1 | 1 q21 | neuroblastoma breakpoint family, member 9 [Source:HGNC Symbol;Acc:HGNC:31991] |
| PCDHGA6 | 0,67 | 8,48 | 2,E-13 | 25 x 2 | 5 q31 | protocadherin gamma subfamily A, 6 [Source:HGNC Symbol;Acc:HGNC:8704] |
| HNRNPCL1 | 0,66 | 8,35 | 4,E-13 | 29 x 1 | 1 p36 | heterogeneous nuclear ribonucleoprotein C-like 1 [Source:HGNC Symbol;Acc:HGNC:29295] |
| CCDC144NL | 0,66 | 8,30 | 5,E-13 | 25 x 3 | 17 p11 | coiled-coil domain containing 144 family, N-terminal like [Source:HGNC Symbol;Acc:HGNC:33735] |
| ACSS1 | 0,66 | 8,28 | 6,E-13 | 27 x 3 | 20 p11 | acyl-CoA synthetase short-chain family member 1 [Source:HGNC Symbol;Acc:HGNC:16091] |
| NLRP12 | 0,66 | 8,23 | 7,E-13 | 25 x 4 | 19 q13 | NLR family, pyrin domain containing 12 [Source:HGNC Symbol;Acc:HGNC:22938] |
| DSE | 0,65 | 8,08 | 1,E-12 | 27 x 1 | 6 q22 | dermatan sulfate epimerase [Source:HGNC Symbol;Acc:HGNC:21144] |
| ADCYAP1 | 0,65 | 8,08 | 1,E-12 | 27 x 4 | 18 p11 | adenylate cyclase activating polypeptide 1 (pituitary) [Source:HGNC Symbol;Acc:HGNC:241] |
| NPIPA8 | 0,64 | 7,92 | 3,E-12 | 28 x 1 | 16 p12 | nuclear pore complex interacting protein family, member A8 [Source:HGNC Symbol;Acc:HGNC:41983] |
| ADCY10 | 0,64 | 7,88 | 4,E-12 | 27 x 5 | 1 q24 | adenylate cyclase 10 (soluble) [Source:HGNC Symbol;Acc:HGNC:21285] |
| AGAP2 | 0,63 | 7,73 | 7,E-12 | 28 x 5 | 12 q14 | ArfGAP with GTPase domain, ankyrin repeat and PH domain 2 [Source:HGNC Symbol;Acc:HGNC:16921] |
| AGAP10 | 0,62 | 7,56 | 2,E-11 | 26 x 3 | NA |  |
| AMER1 | 0,62 | 7,54 | 2,E-11 | 27 x 5 | X q11 | APC membrane recruitment protein 1 [Source:HGNC Symbol;Acc:HGNC:26837] |
| ANKRD61 | 0,62 | 7,49 | 2,E-11 | 26 x 4 | 7 p22 | ankyrin repeat domain 61 [Source:HGNC Symbol;Acc:HGNC:22467] |
| ACCSL | 0,62 | 7,48 | 2,E-11 | 27 x 4 | 11 p11 | 1-aminocyclopropane-1-carboxylate synthase homolog (Arabidopsis)(non-functional)-like [Source:HGNC Symbol;Acc:HGNC:34391] |
| DDTL | 0,62 | 7,45 | 3,E-11 | 27 x 1 | NA | D-dopachrome tautomerase-like [Source:HGNC Symbol;Acc:HGNC:33446] |
| ABCD1 | 0,62 | 7,45 | 3,E-11 | 26 x 1 | X q28 | ATP-binding cassette, sub-family D (ALD), member 1 [Source:HGNC Symbol;Acc:HGNC:61] |
| USP6 | 0,62 | 7,44 | 3,E-11 | 29 x 2 | 17 p13 | ubiquitin specific peptidase 6 [Source:HGNC Symbol;Acc:HGNC:12629] |
| MTA2 | 0,61 | 7,39 | 4,E-11 | 28 x 3 | 11 q12 | metastasis associated 1 family, member 2 [Source:HGNC Symbol;Acc:HGNC:7411] |
| MTRNR2L9 | 0,61 | 7,39 | 4,E-11 | 28 x 1 | 6 q11 | MT-RNR2-like 9 (pseudogene) [Source:HGNC Symbol;Acc:HGNC:37166] |
| FAM71D | 0,61 | 7,39 | 4,E-11 | 28 x 2 | 14 q23 | family with sequence similarity 71, member D [Source:HGNC Symbol;Acc:HGNC:20101] |
| ADAMTS15 | 0,61 | 7,35 | 4,E-11 | 31 x 3 | 11 q24 | ADAM metallopeptidase with thrombospondin type 1 motif, 15 [Source:HGNC Symbol;Acc:HGNC:16305] |
| ADCY7 | 0,61 | 7,24 | 7,E-11 | 29 x 5 | 16 q12 | adenylate cyclase 7 [Source:HGNC Symbol;Acc:HGNC:238] |
| SSX2B | 0,60 | 7,18 | 1,E-10 | 24 x 1 | X p11 | synovial sarcoma, X breakpoint 2B [Source:HGNC Symbol;Acc:HGNC:22263] |
| BAHD1 | 0,60 | 7,14 | 1,E-10 | 28 x 3 | 15 q15 | bromo adjacent homology domain containing 1 [Source:HGNC Symbol;Acc:HGNC:29153] |
| ACRBP | 0,60 | 7,07 | 2,E-10 | 26 x 4 | 12 p13 | acrosin binding protein [Source:HGNC Symbol;Acc:HGNC:17195] |
| NPIPB7 | 0,60 | 7,06 | 2,E-10 | 27 x 1 | 16 p12 | nuclear pore complex interacting protein family, member B7 [Source:HGNC Symbol;Acc:HGNC:33832] |
| ZNF554 | 0,60 | 7,03 | 2,E-10 | 29 x 1 | 19 p13 | zinc finger protein 554 [Source:HGNC Symbol;Acc:HGNC:26629] |
| ALDH3A1 | 0,60 | 7,03 | 2,E-10 | 27 x 4 | 17 p11 | aldehyde dehydrogenase 3 family, member A1 [Source:HGNC Symbol;Acc:HGNC:405] |
| ATP5L2 | 0,59 | 7,02 | 2,E-10 | 28 x 1 | 22 q13 | ATP synthase, H+ transporting, mitochondrial Fo complex, subunit G2 [Source:HGNC Symbol;Acc:HGNC:13213] |
| AC002310.13 | 0,59 | 6,93 | 3,E-10 | 29 x 3 | NA |  |
| FBLIM1 | 0,59 | 6,89 | 4,E-10 | 25 x 3 | 1 p36 | filamin binding LIM protein 1 [Source:HGNC Symbol;Acc:HGNC:24686] |
| ADAM20 | 0,59 | 6,87 | 4,E-10 | 28 x 5 | 14 q24 | ADAM metallopeptidase domain 20 [Source:HGNC Symbol;Acc:HGNC:199] |
| ABHD15 | 0,58 | 6,84 | 5,E-10 | 26 x 2 | 17 q11 | abhydrolase domain containing 15 [Source:HGNC Symbol;Acc:HGNC:26971] |
| ALDH5A1 | 0,58 | 6,83 | 5,E-10 | 28 x 1 | 6 p22 | aldehyde dehydrogenase 5 family, member A1 [Source:HGNC Symbol;Acc:HGNC:408] |
| SSX2 | 0,58 | 6,73 | 8,E-10 | 24 x 1 | X p11 | synovial sarcoma, X breakpoint 2 [Source:HGNC Symbol;Acc:HGNC:11336] |
| CADM2 | 0,57 | 6,64 | 1,E-09 | 24 x 4 | 3 p12 | cell adhesion molecule 2 [Source:HGNC Symbol;Acc:HGNC:29849] |
| NPIPA3 | 0,57 | 6,58 | 2,E-09 | 28 x 1 | NA | nuclear pore complex interacting protein family, member A3 [Source:HGNC Symbol;Acc:HGNC:41978] |
| SMN1 | 0,57 | 6,52 | 2,E-09 | 27 x 2 | NA | survival of motor neuron 1, telomeric [Source:HGNC Symbol;Acc:HGNC:11117] |
| SERF1B | 0,56 | 6,48 | 2,E-09 | 27 x 1 | NA | small EDRK-rich factor 1B (centromeric) [Source:HGNC Symbol;Acc:HGNC:10756] |
| SPDYE5 | 0,56 | 6,40 | 3,E-09 | 27 x 1 | 7 q11 | speedy/RINGO cell cycle regulator family member E5 [Source:HGNC Symbol;Acc:HGNC:35464] |
| AKR1B10 | 0,56 | 6,40 | 3,E-09 | 27 x 5 | 7 q33 | aldo-keto reductase family 1, member B10 (aldose reductase) [Source:HGNC Symbol;Acc:HGNC:382] |
| LRRC37A | 0,56 | 6,34 | 4,E-09 | 27 x 3 | 17 q21 | leucine rich repeat containing 37A [Source:HGNC Symbol;Acc:HGNC:29069] |
| ZNF286B | 0,55 | 6,33 | 5,E-09 | 28 x 1 | 17 p11 | zinc finger protein 286B [Source:HGNC Symbol;Acc:HGNC:33241] |
| HLA-E | 0,55 | 6,29 | 6,E-09 | 28 x 1 | NA | major histocompatibility complex, class I, E [Source:HGNC Symbol;Acc:HGNC:4962] |
| PRICKLE4 | 0,55 | 6,27 | 6,E-09 | 27 x 2 | 6 p21 | prickle homolog 4 (Drosophila) [Source:HGNC Symbol;Acc:HGNC:16805] |
| NPIPA5 | 0,55 | 6,23 | 7,E-09 | 28 x 1 | 16 p13 | nuclear pore complex interacting protein family, member A5 [Source:HGNC Symbol;Acc:HGNC:41980] |
| RGPD6 | 0,55 | 6,21 | 8,E-09 | 27 x 1 | 2 q13 | RANBP2-like and GRIP domain containing 6 [Source:HGNC Symbol;Acc:HGNC:32419] |
| CSNK1A1L | 0,55 | 6,20 | 8,E-09 | 29 x 1 | 13 q13 | casein kinase 1, alpha 1-like [Source:HGNC Symbol;Acc:HGNC:20289] |
| ARL17A | 0,55 | 6,19 | 9,E-09 | 24 x 1 | NA | ADP-ribosylation factor-like 17A [Source:HGNC Symbol;Acc:HGNC:24096] |
| ADAM12 | 0,55 | 6,17 | 9,E-09 | 28 x 3 | 10 q26 | ADAM metallopeptidase domain 12 [Source:HGNC Symbol;Acc:HGNC:190] |
| AP000350.10 | 0,54 | 6,16 | 1,E-08 | 29 x 4 | NA |  |
| ABAT | 0,54 | 6,14 | 1,E-08 | 28 x 5 | 16 p13 | 4-aminobutyrate aminotransferase [Source:HGNC Symbol;Acc:HGNC:23] |
| SEMA6B | 0,54 | 6,13 | 1,E-08 | 24 x 3 | 19 p13 | sema domain, transmembrane domain (TM), and cytoplasmic domain, (semaphorin) 6B [Source:HGNC Symbol;Acc:HGNC:10739] |
| KIAA1919 | 0,54 | 6,11 | 1,E-08 | 29 x 2 | 6 q21 | KIAA1919 [Source:HGNC Symbol;Acc:HGNC:21053] |
| ABCC1 | 0,54 | 6,05 | 2,E-08 | 29 x 2 | NA | ATP-binding cassette, sub-family C (CFTR/MRP), member 1 [Source:HGNC Symbol;Acc:HGNC:51] |
| CHD2 | 0,54 | 6,03 | 2,E-08 | 25 x 1 | 15 q26 | chromodomain helicase DNA binding protein 2 [Source:HGNC Symbol;Acc:HGNC:1917] |
| RGPD8 | 0,53 | 5,98 | 2,E-08 | 26 x 2 | 2 q14 | RANBP2-like and GRIP domain containing 8 [Source:HGNC Symbol;Acc:HGNC:9849] |
| ZBTB8B | 0,53 | 5,88 | 3,E-08 | 24 x 4 | 1 p35 | zinc finger and BTB domain containing 8B [Source:HGNC Symbol;Acc:HGNC:37057] |
| CTD-2545G14.7 | 0,53 | 5,86 | 4,E-08 | 28 x 4 | NA |  |
| ARHGAP22 | 0,53 | 5,85 | 4,E-08 | 28 x 2 | 10 q11 | Rho GTPase activating protein 22 [Source:HGNC Symbol;Acc:HGNC:30320] |
| AC037459.4 | 0,52 | 5,81 | 5,E-08 | 30 x 5 | NA |  |
| ALKBH6 | 0,52 | 5,79 | 5,E-08 | 25 x 1 | 19 q13 | alkB, alkylation repair homolog 6 (E. coli) [Source:HGNC Symbol;Acc:HGNC:28243] |
| AKR1C1 | 0,52 | 5,79 | 5,E-08 | 30 x 1 | 10 p15 | aldo-keto reductase family 1, member C1 [Source:HGNC Symbol;Acc:HGNC:384] |
| ARAP3 | 0,52 | 5,78 | 5,E-08 | 29 x 2 | 5 q31 | ArfGAP with RhoGAP domain, ankyrin repeat and PH domain 3 [Source:HGNC Symbol;Acc:HGNC:24097] |
| ANKRD9 | 0,52 | 5,71 | 7,E-08 | 30 x 4 | 14 q32 | ankyrin repeat domain 9 [Source:HGNC Symbol;Acc:HGNC:20096] |
| NBPF14 | 0,52 | 5,71 | 7,E-08 | 31 x 3 | 1 q21 | neuroblastoma breakpoint family, member 14 [Source:HGNC Symbol;Acc:HGNC:25232] |
| PEX26 | 0,51 | 5,69 | 8,E-08 | 27 x 4 | 22 q11 | peroxisomal biogenesis factor 26 [Source:HGNC Symbol;Acc:HGNC:22965] |
| ATP6AP1L | 0,51 | 5,69 | 8,E-08 | 25 x 5 | 5 q14 | ATPase, H+ transporting, lysosomal accessory protein 1-like [Source:HGNC Symbol;Acc:HGNC:28091] |
| PELP1 | 0,51 | 5,68 | 8,E-08 | 25 x 1 | 17 p13 | proline, glutamate and leucine rich protein 1 [Source:HGNC Symbol;Acc:HGNC:30134] |
| ST3GAL2 | 0,51 | 5,68 | 8,E-08 | 29 x 5 | 16 q22 | ST3 beta-galactoside alpha-2,3-sialyltransferase 2 [Source:HGNC Symbol;Acc:HGNC:10863] |
| ADAMTS13 | 0,51 | 5,66 | 9,E-08 | 28 x 5 | 9 q34 | ADAM metallopeptidase with thrombospondin type 1 motif, 13 [Source:HGNC Symbol;Acc:HGNC:1366] |
| SERF1A | 0,51 | 5,57 | 1,E-07 | 27 x 1 | NA | small EDRK-rich factor 1A (telomeric) [Source:HGNC Symbol;Acc:HGNC:10755] |
| AKR1C3 | 0,50 | 5,51 | 2,E-07 | 26 x 1 | 10 p15 | aldo-keto reductase family 1, member C3 [Source:HGNC Symbol;Acc:HGNC:386] |
| MAVS | 0,50 | 5,49 | 2,E-07 | 29 x 1 | 20 p13 | mitochondrial antiviral signaling protein [Source:HGNC Symbol;Acc:HGNC:29233] |
| ANKRD34A | 0,50 | 5,46 | 2,E-07 | 28 x 5 | 1 q21 | ankyrin repeat domain 34A [Source:HGNC Symbol;Acc:HGNC:27639] |
| ADD2 | 0,50 | 5,41 | 3,E-07 | 29 x 5 | 2 p13 | adducin 2 (beta) [Source:HGNC Symbol;Acc:HGNC:244] |
| KIAA1244 | 0,49 | 5,37 | 3,E-07 | 29 x 1 | NA |  |
| AKT1S1 | 0,49 | 5,37 | 3,E-07 | 24 x 1 | 19 q13 | AKT1 substrate 1 (proline-rich) [Source:HGNC Symbol;Acc:HGNC:28426] |
| AHDC1 | 0,49 | 5,31 | 4,E-07 | 28 x 3 | 1 p35 | AT hook, DNA binding motif, containing 1 [Source:HGNC Symbol;Acc:HGNC:25230] |
| ALOX12 | 0,49 | 5,29 | 4,E-07 | 28 x 5 | 17 p13 | arachidonate 12-lipoxygenase [Source:HGNC Symbol;Acc:HGNC:429] |
| MAML2 | 0,49 | 5,27 | 5,E-07 | 26 x 1 | 11 q21 | mastermind-like 2 (Drosophila) [Source:HGNC Symbol;Acc:HGNC:16259] |
| RAB12 | 0,48 | 5,20 | 6,E-07 | 26 x 3 | 18 p11 | RAB12, member RAS oncogene family [Source:HGNC Symbol;Acc:HGNC:31332] |
| JARID2 | 0,48 | 5,19 | 7,E-07 | 28 x 1 | 6 p22 | jumonji, AT rich interactive domain 2 [Source:HGNC Symbol;Acc:HGNC:6196] |
| EIF5AL1 | 0,48 | 5,16 | 7,E-07 | 29 x 1 | 10 q22 | eukaryotic translation initiation factor 5A-like 1 [Source:HGNC Symbol;Acc:HGNC:17419] |
| EP300 | 0,48 | 5,15 | 8,E-07 | 27 x 1 | 22 q13 | E1A binding protein p300 [Source:HGNC Symbol;Acc:HGNC:3373] |
| IER5 | 0,48 | 5,13 | 8,E-07 | 30 x 5 | 1 q25 | immediate early response 5 [Source:HGNC Symbol;Acc:HGNC:5393] |
| BCORL1 | 0,48 | 5,12 | 8,E-07 | 29 x 1 | X q26 | BCL6 corepressor-like 1 [Source:HGNC Symbol;Acc:HGNC:25657] |
| ADAMTS3 | 0,47 | 5,10 | 9,E-07 | 27 x 5 | 4 q13 | ADAM metallopeptidase with thrombospondin type 1 motif, 3 [Source:HGNC Symbol;Acc:HGNC:219] |
| SMN2 | 0,47 | 5,09 | 1,E-06 | 27 x 2 | 5 q13 | survival of motor neuron 2, centromeric [Source:HGNC Symbol;Acc:HGNC:11118] |
| ABLIM3 | 0,47 | 5,09 | 1,E-06 | 29 x 3 | 5 q32 | actin binding LIM protein family, member 3 [Source:HGNC Symbol;Acc:HGNC:29132] |
| TLE1 | 0,47 | 4,99 | 1,E-06 | 26 x 1 | 9 q21 | transducin-like enhancer of split 1 (E(sp1) homolog, Drosophila) [Source:HGNC Symbol;Acc:HGNC:11837] |
| ADAM21 | 0,46 | 4,96 | 2,E-06 | 28 x 5 | 14 q24 | ADAM metallopeptidase domain 21 [Source:HGNC Symbol;Acc:HGNC:200] |
| ACTR1B | 0,46 | 4,96 | 2,E-06 | 30 x 1 | 2 q11 | ARP1 actin-related protein 1 homolog B, centractin beta (yeast) [Source:HGNC Symbol;Acc:HGNC:168] |
| PLCB3 | 0,46 | 4,94 | 2,E-06 | 29 x 2 | 11 q13 | phospholipase C, beta 3 (phosphatidylinositol-specific) [Source:HGNC Symbol;Acc:HGNC:9056] |
| KRT10 | 0,46 | 4,92 | 2,E-06 | 26 x 1 | 17 q21 | keratin 10, type I [Source:HGNC Symbol;Acc:HGNC:6413] |
| AHNAK2 | 0,46 | 4,92 | 2,E-06 | 28 x 5 | 14 q32 | AHNAK nucleoprotein 2 [Source:HGNC Symbol;Acc:HGNC:20125] |
| MIDN | 0,46 | 4,91 | 2,E-06 | 30 x 1 | 19 p13 | midnolin [Source:HGNC Symbol;Acc:HGNC:16298] |
| RNF152 | 0,45 | 4,85 | 3,E-06 | 27 x 1 | 18 q21 | ring finger protein 152 [Source:HGNC Symbol;Acc:HGNC:26811] |
| APOBEC3A | 0,45 | 4,83 | 3,E-06 | 31 x 4 | NA | apolipoprotein B mRNA editing enzyme, catalytic polypeptide-like 3A [Source:HGNC Symbol;Acc:HGNC:17343] |
| ACSL5 | 0,45 | 4,80 | 3,E-06 | 26 x 4 | 10 q25 | acyl-CoA synthetase long-chain family member 5 [Source:HGNC Symbol;Acc:HGNC:16526] |
| ABCB4 | 0,45 | 4,74 | 4,E-06 | 28 x 5 | 7 q21 | ATP-binding cassette, sub-family B (MDR/TAP), member 4 [Source:HGNC Symbol;Acc:HGNC:45] |
| AK7 | 0,44 | 4,70 | 5,E-06 | 28 x 5 | 14 q32 | adenylate kinase 7 [Source:HGNC Symbol;Acc:HGNC:20091] |
| CCDC180 | 0,44 | 4,68 | 5,E-06 | 25 x 3 | 9 q22 | coiled-coil domain containing 180 [Source:HGNC Symbol;Acc:HGNC:29303] |
| GTF2IRD2 | 0,44 | 4,68 | 5,E-06 | 25 x 1 | 7 q11 | GTF2I repeat domain containing 2 [Source:HGNC Symbol;Acc:HGNC:30775] |
| YWHAG | 0,44 | 4,67 | 5,E-06 | 30 x 1 | 7 q11 | tyrosine 3-monooxygenase/tryptophan 5-monooxygenase activation protein, gamma [Source:HGNC Symbol;Acc:HGNC:12852] |
| ATAT1 | 0,44 | 4,65 | 6,E-06 | 28 x 1 | NA | alpha tubulin acetyltransferase 1 [Source:HGNC Symbol;Acc:HGNC:21186] |
| LAIR1 | 0,44 | 4,62 | 6,E-06 | 24 x 4 | NA | leukocyte-associated immunoglobulin-like receptor 1 [Source:HGNC Symbol;Acc:HGNC:6477] |
| ATG2B | 0,44 | 4,62 | 6,E-06 | 26 x 1 | 14 q32 | autophagy related 2B [Source:HGNC Symbol;Acc:HGNC:20187] |
| POLR2J2 | 0,44 | 4,60 | 7,E-06 | 25 x 1 | 7 q22 | polymerase (RNA) II (DNA directed) polypeptide J2 [Source:HGNC Symbol;Acc:HGNC:23208] |
| NOTCH2NL | 0,44 | 4,60 | 7,E-06 | 30 x 1 | 1 q21 | notch 2 N-terminal like [Source:HGNC Symbol;Acc:HGNC:31862] |
| CDC14B | 0,44 | 4,59 | 7,E-06 | 29 x 1 | 9 q22 | cell division cycle 14B [Source:HGNC Symbol;Acc:HGNC:1719] |
| GAN | 0,43 | 4,53 | 9,E-06 | 28 x 2 | 16 q23 | gigaxonin [Source:HGNC Symbol;Acc:HGNC:4137] |
| PURA | 0,43 | 4,50 | 1,E-05 | 28 x 2 | 5 q31 | purine-rich element binding protein A [Source:HGNC Symbol;Acc:HGNC:9701] |
| FKBP14 | 0,43 | 4,49 | 1,E-05 | 26 x 1 | 7 p14 | FK506 binding protein 14, 22 kDa [Source:HGNC Symbol;Acc:HGNC:18625] |
| GBF1 | 0,43 | 4,47 | 1,E-05 | 30 x 1 | 10 q24 | golgi brefeldin A resistant guanine nucleotide exchange factor 1 [Source:HGNC Symbol;Acc:HGNC:4181] |
| AGAP6 | 0,43 | 4,46 | 1,E-05 | 27 x 2 | 10 q11 | ArfGAP with GTPase domain, ankyrin repeat and PH domain 6 [Source:HGNC Symbol;Acc:HGNC:23466] |
| ABCC4 | 0,42 | 4,45 | 1,E-05 | 26 x 3 | 13 q32 | ATP-binding cassette, sub-family C (CFTR/MRP), member 4 [Source:HGNC Symbol;Acc:HGNC:55] |
| AKNAD1 | 0,42 | 4,42 | 1,E-05 | 29 x 1 | 1 p13 | AKNA domain containing 1 [Source:HGNC Symbol;Acc:HGNC:28398] |
| BRAF | 0,42 | 4,39 | 2,E-05 | 28 x 1 | 7 q34 | B-Raf proto-oncogene, serine/threonine kinase [Source:HGNC Symbol;Acc:HGNC:1097] |
| MAP2K7 | 0,42 | 4,38 | 2,E-05 | 26 x 1 | 19 p13 | mitogen-activated protein kinase kinase 7 [Source:HGNC Symbol;Acc:HGNC:6847] |
| AC003002.4 | 0,42 | 4,36 | 2,E-05 | 28 x 5 | NA |  |
| NUS1 | 0,42 | 4,35 | 2,E-05 | 28 x 1 | 6 q22 | nuclear undecaprenyl pyrophosphate synthase 1 homolog (S. cerevisiae) [Source:HGNC Symbol;Acc:HGNC:21042] |
| ZNF264 | 0,42 | 4,33 | 2,E-05 | 29 x 2 | 19 q13 | zinc finger protein 264 [Source:HGNC Symbol;Acc:HGNC:13057] |
| C20orf194 | 0,41 | 4,32 | 2,E-05 | 26 x 1 | 20 p13 | chromosome 20 open reading frame 194 [Source:HGNC Symbol;Acc:HGNC:17721] |
| ADCY6 | 0,41 | 4,32 | 2,E-05 | 25 x 1 | 12 q13 | adenylate cyclase 6 [Source:HGNC Symbol;Acc:HGNC:237] |
| AC006116.20 | 0,41 | 4,28 | 2,E-05 | 28 x 4 | NA |  |
| AKIRIN1 | 0,41 | 4,22 | 3,E-05 | 28 x 1 | 1 p34 | akirin 1 [Source:HGNC Symbol;Acc:HGNC:25744] |
| ETS1 | 0,40 | 4,19 | 3,E-05 | 28 x 1 | 11 q24 | v-ets avian erythroblastosis virus E26 oncogene homolog 1 [Source:HGNC Symbol;Acc:HGNC:3488] |
| AKAP11 | 0,40 | 4,17 | 4,E-05 | 26 x 4 | 13 q14 | A kinase (PRKA) anchor protein 11 [Source:HGNC Symbol;Acc:HGNC:369] |
| PRKCSH | 0,40 | 4,16 | 4,E-05 | 27 x 1 | 19 p13 | protein kinase C substrate 80K-H [Source:HGNC Symbol;Acc:HGNC:9411] |
| RRP12 | 0,40 | 4,16 | 4,E-05 | 25 x 1 | 10 q24 | ribosomal RNA processing 12 homolog (S. cerevisiae) [Source:HGNC Symbol;Acc:HGNC:29100] |
| NCKAP5L | 0,40 | 4,14 | 4,E-05 | 27 x 1 | 12 q13 | NCK-associated protein 5-like [Source:HGNC Symbol;Acc:HGNC:29321] |
| ENAH | 0,40 | 4,13 | 4,E-05 | 30 x 3 | 1 q42 | enabled homolog (Drosophila) [Source:HGNC Symbol;Acc:HGNC:18271] |
| BOD1L1 | 0,40 | 4,13 | 4,E-05 | 28 x 1 | 4 p15 | biorientation of chromosomes in cell division 1-like 1 [Source:HGNC Symbol;Acc:HGNC:31792] |
| ACVR1B | 0,40 | 4,09 | 5,E-05 | 29 x 5 | 12 q13 | activin A receptor, type IB [Source:HGNC Symbol;Acc:HGNC:172] |
| TRIM27 | 0,39 | 4,07 | 5,E-05 | 30 x 1 | 6 p22 | tripartite motif containing 27 [Source:HGNC Symbol;Acc:HGNC:9975] |
| COCH | 0,39 | 4,07 | 5,E-05 | 29 x 5 | 14 q12 | cochlin [Source:HGNC Symbol;Acc:HGNC:2180] |
| ZNF585B | 0,39 | 4,07 | 5,E-05 | 25 x 1 | 19 q13 | zinc finger protein 585B [Source:HGNC Symbol;Acc:HGNC:30948] |
| ACVR2B | 0,39 | 4,07 | 5,E-05 | 30 x 5 | 3 p22 | activin A receptor, type IIB [Source:HGNC Symbol;Acc:HGNC:174] |
| ZNF543 | 0,39 | 4,06 | 5,E-05 | 27 x 1 | 19 q13 | zinc finger protein 543 [Source:HGNC Symbol;Acc:HGNC:25281] |
| FAM185A | 0,39 | 4,04 | 6,E-05 | 24 x 1 | 7 q22 | family with sequence similarity 185, member A [Source:HGNC Symbol;Acc:HGNC:22412] |
| BCL2L2 | 0,39 | 4,01 | 6,E-05 | 24 x 4 | 14 q11 | BCL2-like 2 [Source:HGNC Symbol;Acc:HGNC:995] |
| RAB21 | 0,39 | 4,01 | 6,E-05 | 26 x 4 | 12 q21 | RAB21, member RAS oncogene family [Source:HGNC Symbol;Acc:HGNC:18263] |
| FNIP1 | 0,39 | 3,99 | 7,E-05 | 29 x 1 | 5 q31 | folliculin interacting protein 1 [Source:HGNC Symbol;Acc:HGNC:29418] |
| GTF2IRD2B | 0,39 | 3,98 | 7,E-05 | 24 x 1 | 7 q11 | GTF2I repeat domain containing 2B [Source:HGNC Symbol;Acc:HGNC:33125] |
| CELSR2 | 0,38 | 3,94 | 8,E-05 | 28 x 1 | 1 p13 | cadherin, EGF LAG seven-pass G-type receptor 2 [Source:HGNC Symbol;Acc:HGNC:3231] |
| SREBF2 | 0,38 | 3,94 | 8,E-05 | 28 x 1 | 22 q13 | sterol regulatory element binding transcription factor 2 [Source:HGNC Symbol;Acc:HGNC:11290] |
| ZNF813 | 0,38 | 3,93 | 8,E-05 | 27 x 1 | 19 q13 | zinc finger protein 813 [Source:HGNC Symbol;Acc:HGNC:33257] |
| KPNA6 | 0,38 | 3,93 | 8,E-05 | 30 x 1 | 1 p35 | karyopherin alpha 6 (importin alpha 7) [Source:HGNC Symbol;Acc:HGNC:6399] |
| MAF | 0,38 | 3,92 | 9,E-05 | 26 x 1 | 16 q23 | v-maf avian musculoaponeurotic fibrosarcoma oncogene homolog [Source:HGNC Symbol;Acc:HGNC:6776] |
| CBL | 0,38 | 3,89 | 1,E-04 | 24 x 4 | 11 q23 | Cbl proto-oncogene, E3 ubiquitin protein ligase [Source:HGNC Symbol;Acc:HGNC:1541] |
| PRPF31 | 0,38 | 3,87 | 1,E-04 | 29 x 1 | NA | pre-mRNA processing factor 31 [Source:HGNC Symbol;Acc:HGNC:15446] |
| BMPR1A | 0,38 | 3,87 | 1,E-04 | 29 x 3 | 10 q23 | bone morphogenetic protein receptor, type IA [Source:HGNC Symbol;Acc:HGNC:1076] |
| PRPF4B | 0,38 | 3,85 | 1,E-04 | 27 x 1 | 6 p25 | pre-mRNA processing factor 4B [Source:HGNC Symbol;Acc:HGNC:17346] |
| ZFYVE26 | 0,37 | 3,83 | 1,E-04 | 27 x 3 | 14 q24 | zinc finger, FYVE domain containing 26 [Source:HGNC Symbol;Acc:HGNC:20761] |
| B4GALT5 | 0,37 | 3,79 | 1,E-04 | 30 x 4 | 20 q13 | UDP-Gal:betaGlcNAc beta 1,4- galactosyltransferase, polypeptide 5 [Source:HGNC Symbol;Acc:HGNC:928] |
| UBE2E1 | 0,37 | 3,78 | 1,E-04 | 27 x 1 | 3 p24 | ubiquitin-conjugating enzyme E2E 1 [Source:HGNC Symbol;Acc:HGNC:12477] |
| PKD1 | 0,37 | 3,76 | 2,E-04 | 26 x 2 | 16 p13 | polycystic kidney disease 1 (autosomal dominant) [Source:HGNC Symbol;Acc:HGNC:9008] |
| GNAI3 | 0,37 | 3,74 | 2,E-04 | 25 x 1 | 1 p13 | guanine nucleotide binding protein (G protein), alpha inhibiting activity polypeptide 3 [Source:HGNC Symbol;Acc:HGNC:4387] |
| CCDC117 | 0,36 | 3,72 | 2,E-04 | 25 x 3 | 22 q12 | coiled-coil domain containing 117 [Source:HGNC Symbol;Acc:HGNC:26599] |
| NBPF15 | 0,36 | 3,70 | 2,E-04 | 26 x 1 | 1 q21 | neuroblastoma breakpoint family, member 15 [Source:HGNC Symbol;Acc:HGNC:28791] |
| BOLA2 | 0,36 | 3,68 | 2,E-04 | 24 x 3 | 16 p11 | bolA family member 2 [Source:HGNC Symbol;Acc:HGNC:29488] |
| AKT3 | 0,36 | 3,68 | 2,E-04 | 24 x 1 | 1 q44 | v-akt murine thymoma viral oncogene homolog 3 [Source:HGNC Symbol;Acc:HGNC:393] |
| CLEC16A | 0,36 | 3,67 | 2,E-04 | 29 x 1 | 16 p13 | C-type lectin domain family 16, member A [Source:HGNC Symbol;Acc:HGNC:29013] |
| PACS2 | 0,36 | 3,66 | 2,E-04 | 28 x 1 | 14 q32 | phosphofurin acidic cluster sorting protein 2 [Source:HGNC Symbol;Acc:HGNC:23794] |
| FAM222B | 0,35 | 3,59 | 3,E-04 | 25 x 2 | 17 q11 | family with sequence similarity 222, member B [Source:HGNC Symbol;Acc:HGNC:25563] |
| AGAP4 | 0,35 | 3,58 | 3,E-04 | 27 x 4 | 10 q11 | ArfGAP with GTPase domain, ankyrin repeat and PH domain 4 [Source:HGNC Symbol;Acc:HGNC:23459] |
| CDK11B | 0,35 | 3,58 | 3,E-04 | 26 x 3 | 1 p36 | cyclin-dependent kinase 11B [Source:HGNC Symbol;Acc:HGNC:1729] |
| AFF3 | 0,35 | 3,57 | 3,E-04 | 28 x 1 | 2 q11 | AF4/FMR2 family, member 3 [Source:HGNC Symbol;Acc:HGNC:6473] |
| DIRC2 | 0,35 | 3,55 | 3,E-04 | 24 x 1 | 3 q21 | disrupted in renal carcinoma 2 [Source:HGNC Symbol;Acc:HGNC:16628] |
| STAG3 | 0,35 | 3,53 | 3,E-04 | 26 x 1 | 7 q22 | stromal antigen 3 [Source:HGNC Symbol;Acc:HGNC:11356] |
| FAM120A | 0,35 | 3,50 | 4,E-04 | 25 x 1 | 9 q22 | family with sequence similarity 120A [Source:HGNC Symbol;Acc:HGNC:13247] |
| ZNF525 | 0,35 | 3,50 | 4,E-04 | 26 x 1 | 19 q13 | zinc finger protein 525 [Source:HGNC Symbol;Acc:HGNC:29423] |
| DDX27 | 0,35 | 3,49 | 4,E-04 | 27 x 1 | 20 q13 | DEAD (Asp-Glu-Ala-Asp) box polypeptide 27 [Source:HGNC Symbol;Acc:HGNC:15837] |
| BRD4 | 0,34 | 3,49 | 4,E-04 | 25 x 1 | 19 p13 | bromodomain containing 4 [Source:HGNC Symbol;Acc:HGNC:13575] |
| ADCK3 | 0,34 | 3,45 | 4,E-04 | 24 x 1 | 1 q42 | aarF domain containing kinase 3 [Source:HGNC Symbol;Acc:HGNC:16812] |
| ANP32A | 0,34 | 3,45 | 4,E-04 | 26 x 1 | 15 q23 | acidic (leucine-rich) nuclear phosphoprotein 32 family, member A [Source:HGNC Symbol;Acc:HGNC:13233] |
| BMP4 | 0,34 | 3,44 | 4,E-04 | 24 x 1 | 14 q22 | bone morphogenetic protein 4 [Source:HGNC Symbol;Acc:HGNC:1071] |
| ARNT | 0,34 | 3,44 | 4,E-04 | 27 x 1 | 1 q21 | aryl hydrocarbon receptor nuclear translocator [Source:HGNC Symbol;Acc:HGNC:700] |
| BTG1 | 0,34 | 3,43 | 5,E-04 | 25 x 1 | 12 q21 | B-cell translocation gene 1, anti-proliferative [Source:HGNC Symbol;Acc:HGNC:1130] |
| AC068533.7 | 0,34 | 3,43 | 5,E-04 | 30 x 5 | NA |  |
| MIB1 | 0,34 | 3,41 | 5,E-04 | 29 x 1 | 18 q11 | mindbomb E3 ubiquitin protein ligase 1 [Source:HGNC Symbol;Acc:HGNC:21086] |
| CSTF2T | 0,34 | 3,41 | 5,E-04 | 28 x 1 | 10 q21 | cleavage stimulation factor, 3' pre-RNA, subunit 2, 64kDa, tau variant [Source:HGNC Symbol;Acc:HGNC:17086] |
| DNAJC24 | 0,34 | 3,40 | 5,E-04 | 25 x 1 | 11 p13 | DnaJ (Hsp40) homolog, subfamily C, member 24 [Source:HGNC Symbol;Acc:HGNC:26979] |
| NELFA | 0,33 | 3,36 | 6,E-04 | 24 x 1 | 4 p16 | negative elongation factor complex member A [Source:HGNC Symbol;Acc:HGNC:12768] |
| PTPN14 | 0,33 | 3,36 | 6,E-04 | 25 x 3 | 1 q41 | protein tyrosine phosphatase, non-receptor type 14 [Source:HGNC Symbol;Acc:HGNC:9647] |
| ANTXR2 | 0,33 | 3,34 | 6,E-04 | 25 x 5 | 4 q21 | anthrax toxin receptor 2 [Source:HGNC Symbol;Acc:HGNC:21732] |
| ACSM3 | 0,33 | 3,34 | 6,E-04 | 27 x 6 | 16 p12 | acyl-CoA synthetase medium-chain family member 3 [Source:HGNC Symbol;Acc:HGNC:10522] |
| ZBTB24 | 0,33 | 3,34 | 6,E-04 | 26 x 2 | 6 q21 | zinc finger and BTB domain containing 24 [Source:HGNC Symbol;Acc:HGNC:21143] |
| ARFGEF2 | 0,33 | 3,33 | 6,E-04 | 29 x 1 | 20 q13 | ADP-ribosylation factor guanine nucleotide-exchange factor 2 (brefeldin A-inhibited) [Source:HGNC Symbol;Acc:HGNC:15853] |
| SGK494 | 0,33 | 3,32 | 7,E-04 | 24 x 1 | NA |  |
| RYBP | 0,33 | 3,29 | 7,E-04 | 25 x 1 | 3 p13 | RING1 and YY1 binding protein [Source:HGNC Symbol;Acc:HGNC:10480] |
| RSBN1L | 0,33 | 3,27 | 8,E-04 | 29 x 2 | 7 q11 | round spermatid basic protein 1-like [Source:HGNC Symbol;Acc:HGNC:24765] |
| TRIM2 | 0,32 | 3,25 | 8,E-04 | 30 x 1 | 4 q31 | tripartite motif containing 2 [Source:HGNC Symbol;Acc:HGNC:15974] |
| ZNF281 | 0,32 | 3,25 | 8,E-04 | 30 x 5 | 1 q32 | zinc finger protein 281 [Source:HGNC Symbol;Acc:HGNC:13075] |
| ARIH1 | 0,32 | 3,23 | 9,E-04 | 26 x 1 | 15 q24 | ariadne RBR E3 ubiquitin protein ligase 1 [Source:HGNC Symbol;Acc:HGNC:689] |
| SSX5 | 0,32 | 3,22 | 9,E-04 | 24 x 2 | X p11 | synovial sarcoma, X breakpoint 5 [Source:HGNC Symbol;Acc:HGNC:11339] |
| QSER1 | 0,32 | 3,20 | 9,E-04 | 29 x 1 | 11 p13 | glutamine and serine rich 1 [Source:HGNC Symbol;Acc:HGNC:26154] |
| FAM73A | 0,32 | 3,20 | 9,E-04 | 25 x 2 | 1 p31 | family with sequence similarity 73, member A [Source:HGNC Symbol;Acc:HGNC:24741] |
| SSX4B | 0,31 | 3,14 | 0,001 | 24 x 1 | X p11 | synovial sarcoma, X breakpoint 4B [Source:HGNC Symbol;Acc:HGNC:16880] |
| SSX4 | 0,31 | 3,11 | 0,001 | 24 x 1 | X p11 | synovial sarcoma, X breakpoint 4 [Source:HGNC Symbol;Acc:HGNC:11338] |
| SH3BGRL2 | 0,31 | 3,10 | 0,001 | 24 x 1 | 6 q14 | SH3 domain binding glutamate-rich protein like 2 [Source:HGNC Symbol;Acc:HGNC:15567] |
| PAPD5 | 0,31 | 3,09 | 0,001 | 30 x 1 | 16 q12 | PAP associated domain containing 5 [Source:HGNC Symbol;Acc:HGNC:30758] |
| C14orf39 | 0,31 | 3,09 | 0,001 | 30 x 2 | 14 q23 | chromosome 14 open reading frame 39 [Source:HGNC Symbol;Acc:HGNC:19849] |
| ICK | 0,31 | 3,08 | 0,001 | 27 x 1 | 6 p12 | intestinal cell (MAK-like) kinase [Source:HGNC Symbol;Acc:HGNC:21219] |
| MAF1 | 0,31 | 3,06 | 0,001 | 27 x 1 | 8 q24 | MAF1 homolog (S. cerevisiae) [Source:HGNC Symbol;Acc:HGNC:24966] |
| BANP | 0,31 | 3,05 | 0,002 | 29 x 1 | 16 q24 | BTG3 associated nuclear protein [Source:HGNC Symbol;Acc:HGNC:13450] |
| PRKCE | 0,30 | 3,01 | 0,002 | 27 x 1 | 2 p21 | protein kinase C, epsilon [Source:HGNC Symbol;Acc:HGNC:9401] |
| MMS19 | 0,30 | 3,00 | 0,002 | 28 x 1 | 10 q24 | MMS19 nucleotide excision repair homolog (S. cerevisiae) [Source:HGNC Symbol;Acc:HGNC:13824] |
| SLC35E2B | 0,30 | 2,99 | 0,002 | 30 x 1 | 1 p36 | solute carrier family 35, member E2B [Source:HGNC Symbol;Acc:HGNC:33941] |
| AAK1 | 0,30 | 2,97 | 0,002 | 30 x 1 | 2 p13 | AP2 associated kinase 1 [Source:HGNC Symbol;Acc:HGNC:19679] |
| BTBD7 | 0,30 | 2,95 | 0,002 | 30 x 2 | NA | BTB (POZ) domain containing 7 [Source:HGNC Symbol;Acc:HGNC:18269] |
| HCFC1R1 | 0,30 | 2,95 | 0,002 | 30 x 1 | 16 p13 | host cell factor C1 regulator 1 (XPO1 dependent) [Source:HGNC Symbol;Acc:HGNC:21198] |
| RNF11 | 0,29 | 2,93 | 0,002 | 29 x 3 | 1 p32 | ring finger protein 11 [Source:HGNC Symbol;Acc:HGNC:10056] |
| DENND1A | 0,29 | 2,92 | 0,002 | 26 x 5 | 9 q33 | DENN/MADD domain containing 1A [Source:HGNC Symbol;Acc:HGNC:29324] |
| RCBTB2 | 0,29 | 2,91 | 0,002 | 24 x 1 | 13 q14 | regulator of chromosome condensation (RCC1) and BTB (POZ) domain containing protein 2 [Source:HGNC Symbol;Acc:HGNC:1914] |
| DDI2 | 0,29 | 2,90 | 0,002 | 24 x 1 | 1 p36 | DNA-damage inducible 1 homolog 2 (S. cerevisiae) [Source:HGNC Symbol;Acc:HGNC:24578] |
| LSM3 | 0,29 | 2,88 | 0,002 | 27 x 6 | 3 p25 | LSM3 homolog, U6 small nuclear RNA associated (S. cerevisiae) [Source:HGNC Symbol;Acc:HGNC:17874] |
| FBXL18 | 0,29 | 2,84 | 0,003 | 25 x 5 | 7 p22 | F-box and leucine-rich repeat protein 18 [Source:HGNC Symbol;Acc:HGNC:21874] |
| ZNF557 | 0,29 | 2,83 | 0,003 | 26 x 1 | 19 p13 | zinc finger protein 557 [Source:HGNC Symbol;Acc:HGNC:28632] |
| PDE12 | 0,29 | 2,82 | 0,003 | 24 x 1 | 3 p14 | phosphodiesterase 12 [Source:HGNC Symbol;Acc:HGNC:25386] |
| TBC1D24 | 0,28 | 2,80 | 0,003 | 24 x 2 | 16 p13 | TBC1 domain family, member 24 [Source:HGNC Symbol;Acc:HGNC:29203] |
| SOX12 | 0,28 | 2,79 | 0,003 | 30 x 3 | 20 p13 | SRY (sex determining region Y)-box 12 [Source:HGNC Symbol;Acc:HGNC:11198] |
| UPF2 | 0,28 | 2,77 | 0,003 | 25 x 3 | 10 p14 | UPF2 regulator of nonsense transcripts homolog (yeast) [Source:HGNC Symbol;Acc:HGNC:17854] |
| RNF25 | 0,28 | 2,76 | 0,003 | 27 x 1 | 2 q35 | ring finger protein 25 [Source:HGNC Symbol;Acc:HGNC:14662] |
| RC3H1 | 0,28 | 2,72 | 0,004 | 27 x 5 | 1 q25 | ring finger and CCCH-type domains 1 [Source:HGNC Symbol;Acc:HGNC:29434] |
| DR1 | 0,27 | 2,71 | 0,004 | 31 x 4 | 1 p22 | down-regulator of transcription 1, TBP-binding (negative cofactor 2) [Source:HGNC Symbol;Acc:HGNC:3017] |
| DCTN4 | 0,27 | 2,68 | 0,004 | 26 x 1 | 5 q33 | dynactin 4 (p62) [Source:HGNC Symbol;Acc:HGNC:15518] |
| ATOH8 | 0,27 | 2,64 | 0,005 | 30 x 5 | 2 p11 | atonal homolog 8 (Drosophila) [Source:HGNC Symbol;Acc:HGNC:24126] |
| UBE2K | 0,27 | 2,63 | 0,005 | 24 x 1 | 4 p14 | ubiquitin-conjugating enzyme E2K [Source:HGNC Symbol;Acc:HGNC:4914] |
| GALK2 | 0,27 | 2,63 | 0,005 | 27 x 1 | 15 q21 | galactokinase 2 [Source:HGNC Symbol;Acc:HGNC:4119] |
| C12orf66 | 0,27 | 2,62 | 0,005 | 27 x 5 | 12 q14 | chromosome 12 open reading frame 66 [Source:HGNC Symbol;Acc:HGNC:26517] |
| ABHD13 | 0,26 | 2,57 | 0,006 | 28 x 1 | 13 q33 | abhydrolase domain containing 13 [Source:HGNC Symbol;Acc:HGNC:20293] |
| PLEKHA4 | 0,26 | 2,56 | 0,006 | 25 x 1 | 19 q13 | pleckstrin homology domain containing, family A (phosphoinositide binding specific) member 4 [Source:HGNC Symbol;Acc:HGNC:14339] |
| ANKRD50 | 0,26 | 2,51 | 0,007 | 31 x 3 | 4 q28 | ankyrin repeat domain 50 [Source:HGNC Symbol;Acc:HGNC:29223] |
| PKN2 | 0,25 | 2,50 | 0,007 | 29 x 1 | 1 p22 | protein kinase N2 [Source:HGNC Symbol;Acc:HGNC:9406] |
| KIF3A | 0,25 | 2,47 | 0,008 | 25 x 2 | 5 q31 | kinesin family member 3A [Source:HGNC Symbol;Acc:HGNC:6319] |
| R3HDM4 | 0,25 | 2,47 | 0,008 | 24 x 1 | 19 p13 | R3H domain containing 4 [Source:HGNC Symbol;Acc:HGNC:28270] |
| SMIM11 | 0,25 | 2,47 | 0,008 | 25 x 4 | 21 p11 | small integral membrane protein 11 [Source:HGNC Symbol;Acc:HGNC:1293] |
| SPOP | 0,25 | 2,46 | 0,008 | 25 x 1 | 17 q21 | speckle-type POZ protein [Source:HGNC Symbol;Acc:HGNC:11254] |
| SCAF8 | 0,25 | 2,44 | 0,008 | 26 x 1 | 6 q25 | SR-related CTD-associated factor 8 [Source:HGNC Symbol;Acc:HGNC:20959] |
| PPRC1 | 0,25 | 2,40 | 0,009 | 24 x 1 | 10 q24 | peroxisome proliferator-activated receptor gamma, coactivator-related 1 [Source:HGNC Symbol;Acc:HGNC:30025] |
| NEDD4 | 0,25 | 2,40 | 0,009 | 27 x 5 | 15 q21 | neural precursor cell expressed, developmentally down-regulated 4, E3 ubiquitin protein ligase [Source:HGNC Symbol;Acc:HGNC:7727] |
| NRAS | 0,24 | 2,37 | 0,010 | 27 x 1 | 1 p13 | neuroblastoma RAS viral (v-ras) oncogene homolog [Source:HGNC Symbol;Acc:HGNC:7989] |
| ATG9A | 0,24 | 2,33 | 0,011 | 31 x 4 | 2 q35 | autophagy related 9A [Source:HGNC Symbol;Acc:HGNC:22408] |
| FBXO30 | 0,24 | 2,33 | 0,011 | 24 x 1 | 6 q24 | F-box protein 30 [Source:HGNC Symbol;Acc:HGNC:15600] |
| RNF149 | 0,24 | 2,32 | 0,011 | 29 x 1 | 2 q11 | ring finger protein 149 [Source:HGNC Symbol;Acc:HGNC:23137] |
| ARRB1 | 0,24 | 2,32 | 0,011 | 24 x 1 | 11 q13 | arrestin, beta 1 [Source:HGNC Symbol;Acc:HGNC:711] |
| LRP8 | 0,24 | 2,32 | 0,011 | 24 x 1 | 1 p32 | low density lipoprotein receptor-related protein 8, apolipoprotein e receptor [Source:HGNC Symbol;Acc:HGNC:6700] |
| CTC-479C5.12 | 0,23 | 2,27 | 0,013 | 25 x 1 | NA |  |
| ZNHIT6 | 0,23 | 2,26 | 0,013 | 30 x 1 | 1 p22 | zinc finger, HIT-type containing 6 [Source:HGNC Symbol;Acc:HGNC:26089] |
| HNRNPA0 | 0,22 | 2,17 | 0,016 | 30 x 4 | 5 q31 | heterogeneous nuclear ribonucleoprotein A0 [Source:HGNC Symbol;Acc:HGNC:5030] |
| ZNF550 | 0,22 | 2,13 | 0,018 | 30 x 5 | 19 q13 | zinc finger protein 550 [Source:HGNC Symbol;Acc:HGNC:28643] |
| KLF6 | 0,21 | 2,08 | 0,020 | 27 x 1 | 10 p15 | Kruppel-like factor 6 [Source:HGNC Symbol;Acc:HGNC:2235] |
| ALAS1 | 0,21 | 2,08 | 0,020 | 24 x 2 | 3 p21 | 5'-aminolevulinate synthase 1 [Source:HGNC Symbol;Acc:HGNC:396] |
| PHYH | 0,21 | 2,07 | 0,021 | 26 x 5 | 10 p13 | phytanoyl-CoA 2-hydroxylase [Source:HGNC Symbol;Acc:HGNC:8940] |
| FAHD2B | 0,21 | 2,04 | 0,022 | 25 x 2 | 2 q11 | fumarylacetoacetate hydrolase domain containing 2B [Source:HGNC Symbol;Acc:HGNC:25318] |
| CLN5 | 0,20 | 1,98 | 0,026 | 24 x 1 | 13 q22 | ceroid-lipofuscinosis, neuronal 5 [Source:HGNC Symbol;Acc:HGNC:2076] |
| TBC1D14 | 0,20 | 1,90 | 0,031 | 28 x 4 | 4 p16 | TBC1 domain family, member 14 [Source:HGNC Symbol;Acc:HGNC:29246] |
| CSRP2BP | 0,19 | 1,86 | 0,033 | 24 x 1 | 20 p11 | CSRP2 binding protein [Source:HGNC Symbol;Acc:HGNC:15904] |
| YTHDF1 | 0,19 | 1,79 | 0,039 | 30 x 1 | 20 q13 | YTH N(6)-methyladenosine RNA binding protein 1 [Source:HGNC Symbol;Acc:HGNC:15867] |
| MED7 | 0,15 | 1,48 | 0,071 | 31 x 4 | 5 q33 | mediator complex subunit 7 [Source:HGNC Symbol;Acc:HGNC:2378] |
| STAM2 | 0,14 | 1,36 | 0,088 | 24 x 1 | 2 q23 | signal transducing adaptor molecule (SH3 domain and ITAM motif) 2 [Source:HGNC Symbol;Acc:HGNC:11358] |

^1^correlation to spot profile; ^2^ x- and y-coordinates of gene position in SOM;
