# Supplementary material for: Mapping heterogeneity in patient-derived melanoma cultures by single-cell RNA-seq
Source: Oncotarget. 2016 Nov 26;8(1):846–62. doi: 10.18632/oncotarget.13666 (PMC5352202; doi:10.18632/oncotarget.13666)
Supplement: Supplementary file 4 [file oncotarget-08-846-s004.rtf]

Genes upregulated in wt/wt	
Symbol	correlation	->t.score	->p.value	Metagene	Chromosome	Description	
PMEL	0,94	50,32	0	1 x 1	12 q13	premelanosome protein [Source:HGNC Symbol;Acc:HGNC:10880]	
GSTP1	0,92	40,96	0	1 x 1	11 q13	glutathione S-transferase pi 1 [Source:HGNC Symbol;Acc:HGNC:4638]	
GPR143	0,91	38,77	0	1 x 1	X p22	G protein-coupled receptor 143 [Source:HGNC Symbol;Acc:HGNC:20145]	
MBP	0,91	38,63	0	1 x 1	18 q23	myelin basic protein [Source:HGNC Symbol;Acc:HGNC:6925]	
SLC45A2	0,90	37,00	0	1 x 1	CHR_HSCHR5_	solute carrier family 45 member 2 [Source:HGNC Symbol;Acc:HGNC:16472]	
TRPM1	0,89	34,99	0	1 x 1	CHR_HSCHR15	transient receptor potential cation channel subfamily M member 1 [Source:HGNC Symbol;Acc:HGNC:7146]	
CDK2	0,89	34,26	0	1 x 1	12 q13	cyclin dependent kinase 2 [Source:HGNC Symbol;Acc:HGNC:1771]	
NARS2	0,89	33,71	0	1 x 1	11 q14	asparaginyl-tRNA synthetase 2, mitochondrial (putative) [Source:HGNC Symbol;Acc:HGNC:26274]	
GPR161	0,89	33,45	0	1 x 1	1 q24	G protein-coupled receptor 161 [Source:HGNC Symbol;Acc:HGNC:23694]	
KDR	0,89	33,20	0	1 x 1	4 q12	kinase insert domain receptor [Source:HGNC Symbol;Acc:HGNC:6307]	
TYRP1	0,88	32,84	0	1 x 1	9 p23	tyrosinase related protein 1 [Source:HGNC Symbol;Acc:HGNC:12450]	
EXOC3	0,88	32,33	0	1 x 1	5 p15	exocyst complex component 3 [Source:HGNC Symbol;Acc:HGNC:30378]	
SEMA6A	0,87	31,48	0	1 x 1	5 q23	semaphorin 6A [Source:HGNC Symbol;Acc:HGNC:10738]	
CITED1	0,86	29,44	0	1 x 1	X q13	Cbp/p300 interacting transactivator with Glu/Asp rich carboxy-terminal domain 1 [Source:HGNC Symbol;Acc:HGNC:1986]	
COL11A1	0,85	28,73	0	1 x 1	1 p21	collagen type XI alpha 1 chain [Source:HGNC Symbol;Acc:HGNC:2186]	
DAB2	0,85	27,99	0	1 x 1	5 p13	DAB2, clathrin adaptor protein [Source:HGNC Symbol;Acc:HGNC:2662]	
PHACTR1	0,84	27,12	0	1 x 1	CHR_HSCHR6_	phosphatase and actin regulator 1 [Source:HGNC Symbol;Acc:HGNC:20990]	
CAPN3	0,84	26,94	0	1 x 1	15 q15	calpain 3 [Source:HGNC Symbol;Acc:HGNC:1480]	
SPCS2	0,83	25,84	0	1 x 1	11 q13	signal peptidase complex subunit 2 [Source:HGNC Symbol;Acc:HGNC:28962]	
RCN1	0,82	25,20	0	1 x 1	11 p13	reticulocalbin 1 [Source:HGNC Symbol;Acc:HGNC:9934]	
TTC39A	0,82	24,84	0	1 x 1	1 p32	tetratricopeptide repeat domain 39A [Source:HGNC Symbol;Acc:HGNC:18657]	
CLNS1A	0,81	24,40	0	1 x 1	11 q14	chloride nucleotide-sensitive channel 1A [Source:HGNC Symbol;Acc:HGNC:2080]	
NDUFC2	0,81	24,38	0	3 x 1	11 q14	NADH:ubiquinone oxidoreductase subunit C2 [Source:HGNC Symbol;Acc:HGNC:7706]	
OCA2	0,81	24,29	0	1 x 1	CHR_HSCHR15	OCA2 melanosomal transmembrane protein [Source:HGNC Symbol;Acc:HGNC:8101]	
VAT1	0,80	23,43	0	1 x 1	17 q21	vesicle amine transport 1 [Source:HGNC Symbol;Acc:HGNC:16919]	
MTMR2	0,80	23,11	0	1 x 1	11 q21	myotubularin related protein 2 [Source:HGNC Symbol;Acc:HGNC:7450]	
ST6GALNAC1	0,80	22,98	0	1 x 1	17 q25	ST6 N-acetylgalactosaminide alpha-2,6-sialyltransferase 1 [Source:HGNC Symbol;Acc:HGNC:23614]	
QPCT	0,79	22,82	0	1 x 1	2 p22	glutaminyl-peptide cyclotransferase [Source:HGNC Symbol;Acc:HGNC:9753]	
LIMS2	0,79	22,71	0	1 x 1	2 q14	LIM zinc finger domain containing 2 [Source:HGNC Symbol;Acc:HGNC:16084]	
DCUN1D5	0,79	22,52	0	1 x 1	11 q22	defective in cullin neddylation 1 domain containing 5 [Source:HGNC Symbol;Acc:HGNC:28409]	
TUBB4A	0,79	22,51	0	1 x 1	19 p13	tubulin beta 4A class IVa [Source:HGNC Symbol;Acc:HGNC:20774]	


MLIP	0,78	22,07	0	1 x 1	6 p12	muscular LMNA-interacting protein [Source:HGNC Symbol;Acc:HGNC:21355]	
EEF1G	0,78	21,89	0	1 x 5	11 q12	eukaryotic translation elongation factor 1 gamma [Source:HGNC Symbol;Acc:HGNC:3213]	
PDE4DIP	0,78	21,85	0	1 x 1	1 q21	phosphodiesterase 4D interacting protein [Source:HGNC Symbol;Acc:HGNC:15580]	
SORT1	0,78	21,64	0	1 x 1	1 p13	sortilin 1 [Source:HGNC Symbol;Acc:HGNC:11186]	
TWF1	0,77	21,29	0	1 x 1	12 q12	twinfilin actin binding protein 1 [Source:HGNC Symbol;Acc:HGNC:9620]	
ARHGAP8	0,77	21,21	0	1 x 1	22 q13	Rho GTPase activating protein 8 [Source:HGNC Symbol;Acc:HGNC:677]	
SNCA	0,77	21,20	0	1 x 1	4 q22	synuclein alpha [Source:HGNC Symbol;Acc:HGNC:11138]	
CCDC171	0,77	21,18	0	1 x 1	9 p22	coiled-coil domain containing 171 [Source:HGNC Symbol;Acc:HGNC:29828]	
CHCHD6	0,77	21,00	0	1 x 1	3 q21	coiled-coil-helix-coiled-coil-helix domain containing 6 [Source:HGNC Symbol;Acc:HGNC:28184]	
	0,77	20,91	0	1 x 1			
CEP57	0,77	20,87	0	1 x 1	11 q21	centrosomal protein 57 [Source:HGNC Symbol;Acc:HGNC:30794]	
SLC24A5	0,77	20,83	0	1 x 1	15 q21	solute carrier family 24 member 5 [Source:HGNC Symbol;Acc:HGNC:20611]	
NARF	0,76	20,68	0	1 x 1	17 q25	nuclear prelamin A recognition factor [Source:HGNC Symbol;Acc:HGNC:29916]	
FTH1	0,76	20,51	0	1 x 2	11 q12	ferritin heavy chain 1 [Source:HGNC Symbol;Acc:HGNC:3976]	
RAB32	0,76	20,41	0	1 x 1	6 q24	RAB32, member RAS oncogene family [Source:HGNC Symbol;Acc:HGNC:9772]	
ALG8	0,76	20,17	0	1 x 1	11 q14	ALG8, alpha-1,3-glucosyltransferase [Source:HGNC Symbol;Acc:HGNC:23161]	
TBC1D7	0,76	20,15	0	1 x 1	6 p24	TBC1 domain family member 7 [Source:HGNC Symbol;Acc:HGNC:21066]	
PPARGC1A	0,75	19,80	0	1 x 1	4 p15	PPARG coactivator 1 alpha [Source:HGNC Symbol;Acc:HGNC:9237]	
SCD	0,75	19,53	0	2 x 1	10 q24	stearoyl-CoA desaturase [Source:HGNC Symbol;Acc:HGNC:10571]	
ALDOA	0,74	19,46	0	2 x 6	16 p11	aldolase, fructose-bisphosphate A [Source:HGNC Symbol;Acc:HGNC:414]	
TTYH3	0,74	19,39	0	1 x 2	7 p22	tweety family member 3 [Source:HGNC Symbol;Acc:HGNC:22222]	
MLANA	0,74	19,30	0	1 x 1	9 p24	melan-A [Source:HGNC Symbol;Acc:HGNC:7124]	
PGM1	0,74	19,02	0	1 x 1	1 p31	phosphoglucomutase 1 [Source:HGNC Symbol;Acc:HGNC:8905]	
PDE4D	0,73	18,69	0	1 x 1	5 q12	phosphodiesterase 4D [Source:HGNC Symbol;Acc:HGNC:8783]	
METTL9	0,73	18,52	0	1 x 1	16 p12	methyltransferase like 9 [Source:HGNC Symbol;Acc:HGNC:24586]	
FAM207A	0,72	18,22	0	1 x 1	21 q22	family with sequence similarity 207 member A [Source:HGNC Symbol;Acc:HGNC:15811]	
CD63	0,72	18,15	0	4 x 5	12 q13	CD63 molecule [Source:HGNC Symbol;Acc:HGNC:1692]	
UCP2	0,72	18,06	0	1 x 1	11 q13	uncoupling protein 2 [Source:HGNC Symbol;Acc:HGNC:12518]	
SDHA	0,72	18,01	0	1 x 1	5 p15	succinate dehydrogenase complex flavoprotein subunit A [Source:HGNC Symbol;Acc:HGNC:10680]	
KIT	0,72	17,94	0	1 x 1	4 q12	KIT proto-oncogene receptor tyrosine kinase [Source:HGNC Symbol;Acc:HGNC:6342]	
TMEM123	0,71	17,80	0	1 x 1	11 q22	transmembrane protein 123 [Source:HGNC Symbol;Acc:HGNC:30138]	
HSP90AA1	0,71	17,68	0	5 x 5	14 q32	heat shock protein 90kDa alpha family class A member 1 [Source:HGNC Symbol;Acc:HGNC:5253]	
DUS1L	0,71	17,51	0	1 x 2	17 q25	dihydrouridine synthase 1 like [Source:HGNC Symbol;Acc:HGNC:30086]	


PEBP1	0,71	17,49	0	3 x 5	12 q24	phosphatidylethanolamine binding protein 1 [Source:HGNC Symbol;Acc:HGNC:8630]	
NMRK2	0,70	17,36	0	1 x 1	19 p13	nicotinamide riboside kinase 2 [Source:HGNC Symbol;Acc:HGNC:17871]	
CCDC82	0,70	17,31	0	1 x 1	11 q21	coiled-coil domain containing 82 [Source:HGNC Symbol;Acc:HGNC:26282]	
GPS1	0,70	17,26	0	1 x 3	17 q25	G protein pathway suppressor 1 [Source:HGNC Symbol;Acc:HGNC:4549]	
NAV2	0,70	17,16	0	1 x 1	11 p15	neuron navigator 2 [Source:HGNC Symbol;Acc:HGNC:15997]	
SLC25A5	0,70	17,12	0	6 x 1	X q24	solute carrier family 25 member 5 [Source:HGNC Symbol;Acc:HGNC:10991]	
DYNC2H1	0,70	17,12	0	1 x 1	11 q22	dynein cytoplasmic 2 heavy chain 1 [Source:HGNC Symbol;Acc:HGNC:2962]	
CABLES1	0,70	17,09	0	1 x 1	18 q11	Cdk5 and Abl enzyme substrate 1 [Source:HGNC Symbol;Acc:HGNC:25097]	
RSF1	0,69	16,83	0	1 x 1	11 q14	remodeling and spacing factor 1 [Source:HGNC Symbol;Acc:HGNC:18118]	
OSTM1	0,69	16,81	0	1 x 3	6 q21	osteopetrosis associated transmembrane protein 1 [Source:HGNC Symbol;Acc:HGNC:21652]	
NDUFV1	0,69	16,72	0	1 x 1	11 q13	NADH:ubiquinone oxidoreductase core subunit V1 [Source:HGNC Symbol;Acc:HGNC:7716]	
ITPKB	0,69	16,66	0	2 x 1	1 q42	inositol-trisphosphate 3-kinase B [Source:HGNC Symbol;Acc:HGNC:6179]	
TNFRSF14	0,69	16,62	0	1 x 3	1 p36	tumor necrosis factor receptor superfamily member 14 [Source:HGNC Symbol;Acc:HGNC:11912]	
PFKFB2	0,69	16,54	0	2 x 1	1 q32	6-phosphofructo-2-kinase/fructose-2,6-biphosphatase 2 [Source:HGNC Symbol;Acc:HGNC:8873]	
CA14	0,69	16,52	0	1 x 1	1 q21	carbonic anhydrase 14 [Source:HGNC Symbol;Acc:HGNC:1372]	
SLC6A15	0,69	16,43	0	1 x 1	12 q21	solute carrier family 6 member 15 [Source:HGNC Symbol;Acc:HGNC:13621]	
MSC	0,68	16,36	0	1 x 1	8 q13	musculin [Source:HGNC Symbol;Acc:HGNC:7321]	
TRAK2	0,68	16,28	0	1 x 1	2 q33	trafficking kinesin protein 2 [Source:HGNC Symbol;Acc:HGNC:13206]	
NDUFS8	0,68	16,27	0	1 x 2	11 q13	NADH:ubiquinone oxidoreductase core subunit S8 [Source:HGNC Symbol;Acc:HGNC:7715]	
RAB27A	0,68	16,15	0,00E+00	1 x 1	15 q21	RAB27A, member RAS oncogene family [Source:HGNC Symbol;Acc:HGNC:9766]	
MTRNR2L2	0,68	16,14	0,00E+00	1 x 5	3 q11	MT-RNR2-like 12 [Source:HGNC Symbol;Acc:HGNC:37169]	
ID2	0,68	16,08	0,00E+00	1 x 1	2 p25	inhibitor of DNA binding 2, HLH protein [Source:HGNC Symbol;Acc:HGNC:5361]	
CNDP2	0,68	16,07	0,00E+00	1 x 1	18 q22	CNDP dipeptidase 2 (metallopeptidase M20 family) [Source:HGNC Symbol;Acc:HGNC:24437]	
MAGEC1	0,68	16,06	0,00E+00	1 x 1	X q27	MAGE family member C1 [Source:HGNC Symbol;Acc:HGNC:6812]	
NCCRP1	0,68	16,00	0,00E+00	2 x 1	19 q13	non-specific cytotoxic cell receptor protein 1 homolog (zebrafish) [Source:HGNC Symbol;Acc:HGNC:33739]	
PPP6R3	0,67	15,93	0,00E+00	3 x 1	11 q13	protein phosphatase 6 regulatory subunit 3 [Source:HGNC Symbol;Acc:HGNC:1173]	
RAP2B	0,67	15,88	0,00E+00	5 x 1	3 q25	RAP2B, member of RAS oncogene family [Source:HGNC Symbol;Acc:HGNC:9862]	
FABP3	0,67	15,87	0,00E+00	1 x 1	1 p35	fatty acid binding protein 3 [Source:HGNC Symbol;Acc:HGNC:3557]	
LAMTOR1	0,67	15,87	0,00E+00	1 x 2	11 q13	late endosomal/lysosomal adaptor, MAPK and MTOR activator 1 [Source:HGNC Symbol;Acc:HGNC:26068]	
FAM76B	0,67	15,82	0,00E+00	1 x 1	11 q21	family with sequence similarity 76 member B [Source:HGNC Symbol;Acc:HGNC:28492]	
CTNNB1	0,67	15,75	0,00E+00	6 x 1	3 p22	catenin beta 1 [Source:HGNC Symbol;Acc:HGNC:2514]	
ATP5O	0,67	15,73	0,00E+00	5 x 4	21 q22	ATP synthase, H+ transporting, mitochondrial F1 complex, O subunit [Source:HGNC Symbol;Acc:HGNC:850]	
MTRNR2L8	0,67	15,68	0,00E+00	1 x 6	11 p15	MT-RNR2-like 8 [Source:HGNC Symbol;Acc:HGNC:37165]	


GYPC	0,67	15,60	0,00E+00	1 x 2	2 q14	glycophorin C (Gerbich blood group) [Source:HGNC Symbol;Acc:HGNC:4704]	
BOLA2	0,66	15,48	0,00E+00	1 x 5	16 p11	bolA family member 2B [Source:HGNC Symbol;Acc:HGNC:32479]	
MITF	0,66	15,45	0,00E+00	1 x 1	3 p13	melanogenesis associated transcription factor [Source:HGNC Symbol;Acc:HGNC:7105]	
PPM1K	0,66	15,43	0,00E+00	1 x 1	4 q22	protein phosphatase, Mg2+/Mn2+ dependent 1K [Source:HGNC Symbol;Acc:HGNC:25415]	
C21orf91	0,66	15,29	0,00E+00	1 x 1	21 q21	chromosome 21 open reading frame 91 [Source:HGNC Symbol;Acc:HGNC:16459]	
SLC25A4	0,66	15,22	0,00E+00	1 x 2	4 q35	solute carrier family 25 member 4 [Source:HGNC Symbol;Acc:HGNC:10990]	
MYO10	0,66	15,20	0,00E+00	2 x 3	5 p15	myosin X [Source:HGNC Symbol;Acc:HGNC:7593]	
BHLHE41	0,66	15,19	0,00E+00	3 x 1	12 p12	basic helix-loop-helix family member e41 [Source:HGNC Symbol;Acc:HGNC:16617]	
FAM101B	0,66	15,14	0,00E+00	2 x 1	17 p13	family with sequence similarity 101 member B [Source:HGNC Symbol;Acc:HGNC:28705]	
AAMDC	0,65	15,05	0,00E+00	1 x 1	11 q14	adipogenesis associated, Mth938 domain containing [Source:HGNC Symbol;Acc:HGNC:30205]	
MRE11A	0,65	14,95	0,00E+00	1 x 1	11 q21	MRE11 homolog A, double strand break repair nuclease [Source:HGNC Symbol;Acc:HGNC:7230]	
PRUNE2	0,65	14,93	0,00E+00	1 x 1	9 q21	prune homolog 2 [Source:HGNC Symbol;Acc:HGNC:25209]	
GAPDHS	0,65	14,90	0,00E+00	1 x 1	19 q13	glyceraldehyde-3-phosphate dehydrogenase, spermatogenic [Source:HGNC Symbol;Acc:HGNC:24864]	
DFNA5	0,65	14,83	0,00E+00	1 x 2	7 p15	DFNA5, deafness associated tumor suppressor [Source:HGNC Symbol;Acc:HGNC:2810]	
NNT	0,65	14,80	0,00E+00	1 x 1	5 p12	nicotinamide nucleotide transhydrogenase [Source:HGNC Symbol;Acc:HGNC:7863]	
HPGD	0,64	14,70	0,00E+00	1 x 1	4 q34	hydroxyprostaglandin dehydrogenase 15-(NAD) [Source:HGNC Symbol;Acc:HGNC:5154]	
NDUFV2	0,64	14,65	0,00E+00	1 x 5	18 p11	NADH:ubiquinone oxidoreductase core subunit V2 [Source:HGNC Symbol;Acc:HGNC:7717]	
CCT5	0,64	14,64	0,00E+00	4 x 5	5 p15	chaperonin containing TCP1 subunit 5 [Source:HGNC Symbol;Acc:HGNC:1618]	
ASAH1	0,64	14,64	0,00E+00	1 x 2	8 p22	N-acylsphingosine amidohydrolase (acid ceramidase) 1 [Source:HGNC Symbol;Acc:HGNC:735]	
FAM213A	0,64	14,61	0,00E+00	1 x 1	10 q23	family with sequence similarity 213 member A [Source:HGNC Symbol;Acc:HGNC:28651]	
EIF4A3	0,64	14,57	0,00E+00	1 x 3	17 q25	eukaryotic translation initiation factor 4A3 [Source:HGNC Symbol;Acc:HGNC:18683]	
OLFM1	0,64	14,45	0,00E+00	1 x 1	9 q34	olfactomedin 1 [Source:HGNC Symbol;Acc:HGNC:17187]	
ROBO2	0,64	14,44	0,00E+00	3 x 1	3 p12	roundabout guidance receptor 2 [Source:HGNC Symbol;Acc:HGNC:10250]	
IFT81	0,64	14,36	0,00E+00	1 x 1	12 q24	intraflagellar transport 81 [Source:HGNC Symbol;Acc:HGNC:14313]	
MRPL48	0,63	14,29	0,00E+00	1 x 1	11 q13	mitochondrial ribosomal protein L48 [Source:HGNC Symbol;Acc:HGNC:16653]	
BEST1	0,63	14,23	0,00E+00	3 x 1	11 q12	bestrophin 1 [Source:HGNC Symbol;Acc:HGNC:12703]	
PIGY	0,63	14,19	0,00E+00	1 x 5	4 q22	phosphatidylinositol glycan anchor biosynthesis class Y [Source:HGNC Symbol;Acc:HGNC:28213]	
PDCD6	0,63	14,11	0,00E+00	1 x 3	5 p15	programmed cell death 6 [Source:HGNC Symbol;Acc:HGNC:8765]	
MRPL12	0,63	14,04	0,00E+00	1 x 3	17 q25	mitochondrial ribosomal protein L12 [Source:HGNC Symbol;Acc:HGNC:10378]	
SULT1C2	0,62	13,95	0,00E+00	2 x 1	2 q12	sulfotransferase family 1C member 2 [Source:HGNC Symbol;Acc:HGNC:11456]	
CWF19L2	0,62	13,81	0,00E+00	3 x 1	11 q22	CWF19-like 2, cell cycle control (S. pombe) [Source:HGNC Symbol;Acc:HGNC:26508]	
MYEF2	0,62	13,80	0,00E+00	2 x 1	15 q21	myelin expression factor 2 [Source:HGNC Symbol;Acc:HGNC:17940]	
H3F3C	0,62	13,78	0,00E+00	1 x 6	12 p11	H3 histone, family 3C [Source:HGNC Symbol;Acc:HGNC:33164]	


SLC39A6	0,62	13,76	0,00E+00	1 x 2	18 q12	solute carrier family 39 member 6 [Source:HGNC Symbol;Acc:HGNC:18607]	
	0,62	13,75	0,00E+00	4 x 9			
APOE	0,62	13,69	0,00E+00	2 x 3	19 q13	apolipoprotein E [Source:HGNC Symbol;Acc:HGNC:613]	
SLC3A2	0,62	13,69	0,00E+00	1 x 4	11 q12	solute carrier family 3 member 2 [Source:HGNC Symbol;Acc:HGNC:11026]	
BTG3	0,62	13,64	0,00E+00	1 x 3	CHR_HSCHR21	BTG family member 3 [Source:HGNC Symbol;Acc:HGNC:1132]	
HSPB8	0,61	13,54	0,00E+00	1 x 1	12 q24	heat shock protein family B (small) member 8 [Source:HGNC Symbol;Acc:HGNC:30171]	
BIRC7	0,61	13,53	0,00E+00	1 x 4	20 q13	baculoviral IAP repeat containing 7 [Source:HGNC Symbol;Acc:HGNC:13702]	
P4HB	0,61	13,53	0,00E+00	1 x 6	17 q25	prolyl 4-hydroxylase subunit beta [Source:HGNC Symbol;Acc:HGNC:8548]	
INTS4	0,61	13,52	0,00E+00	1 x 1	11 q14	integrator complex subunit 4 [Source:HGNC Symbol;Acc:HGNC:25048]	
WFDC1	0,61	13,51	0,00E+00	4 x 1	16 q24	WAP four-disulfide core domain 1 [Source:HGNC Symbol;Acc:HGNC:15466]	
PCBP2	0,61	13,43	0,00E+00	3 x 3	12 q13	poly(rC) binding protein 2 [Source:HGNC Symbol;Acc:HGNC:8648]	
SCARB1	0,61	13,41	0,00E+00	1 x 3	12 q24	scavenger receptor class B member 1 [Source:HGNC Symbol;Acc:HGNC:1664]	
EIF4G2	0,61	13,39	0,00E+00	2 x 6	11 p15	eukaryotic translation initiation factor 4 gamma 2 [Source:HGNC Symbol;Acc:HGNC:3297]	
SLC7A5	0,61	13,39	0,00E+00	5 x 1	16 q24	solute carrier family 7 member 5 [Source:HGNC Symbol;Acc:HGNC:11063]	
CTSD	0,61	13,37	0,00E+00	1 x 3	11 p15	cathepsin D [Source:HGNC Symbol;Acc:HGNC:2529]	
CHKA	0,61	13,33	0,00E+00	1 x 1	11 q13	choline kinase alpha [Source:HGNC Symbol;Acc:HGNC:1937]	
C17orf62	0,61	13,29	0,00E+00	1 x 3	17 q25	chromosome 17 open reading frame 62 [Source:HGNC Symbol;Acc:HGNC:28672]	
TMC6	0,60	13,24	0,00E+00	1 x 5	17 q25	transmembrane channel like 6 [Source:HGNC Symbol;Acc:HGNC:18021]	
BIRC2	0,60	13,23	0,00E+00	2 x 3	11 q22	baculoviral IAP repeat containing 2 [Source:HGNC Symbol;Acc:HGNC:590]	
MRPL21	0,60	13,22	0,00E+00	2 x 1	11 q13	mitochondrial ribosomal protein L21 [Source:HGNC Symbol;Acc:HGNC:14479]	
RRAGD	0,60	13,01	0,00E+00	2 x 1	6 q15	Ras related GTP binding D [Source:HGNC Symbol;Acc:HGNC:19903]	
HES6	0,60	12,97	0,00E+00	5 x 1	2 q37	hes family bHLH transcription factor 6 [Source:HGNC Symbol;Acc:HGNC:18254]	
SLC12A7	0,60	12,96	0,00E+00	6 x 3	5 p15	solute carrier family 12 member 7 [Source:HGNC Symbol;Acc:HGNC:10915]	
	0,60	12,96	0,00E+00	2 x 7	13 q12	ATP synthase, H+ transporting, mitochondrial F1 complex, epsilon subunit pseudogene 2 [Source:HGNC Symbol;Acc:HGNC:34026]	
UCK1	0,59	12,93	0,00E+00	1 x 2	9 q34	uridine-cytidine kinase 1 [Source:HGNC Symbol;Acc:HGNC:14859]	
ERC2	0,59	12,80	0,00E+00	1 x 3	3 p14	ELKS/RAB6-interacting/CAST family member 2 [Source:HGNC Symbol;Acc:HGNC:31922]	
PAIP1	0,59	12,80	0,00E+00	3 x 2	5 p12	poly(A) binding protein interacting protein 1 [Source:HGNC Symbol;Acc:HGNC:16945]	
PDK4	0,59	12,78	0,00E+00	1 x 1	7 q21	pyruvate dehydrogenase kinase 4 [Source:HGNC Symbol;Acc:HGNC:8812]	
	0,59	12,74	0,00E+00	1 x 4			
CD81	0,59	12,70	0,00E+00	1 x 2	11 p15	CD81 molecule [Source:HGNC Symbol;Acc:HGNC:1701]	
FAHD1	0,59	12,67	0,00E+00	1 x 1	16 p13	fumarylacetoacetate hydrolase domain containing 1 [Source:HGNC Symbol;Acc:HGNC:14169]	
DCXR	0,59	12,64	0,00E+00	1 x 5	17 q25	dicarbonyl/L-xylulose reductase [Source:HGNC Symbol;Acc:HGNC:18985]	
EIF3C	0,59	12,64	0,00E+00	1 x 6	16 p11	eukaryotic translation initiation factor 3 subunit C [Source:HGNC Symbol;Acc:HGNC:3279]	


TSPAN10	0,59	12,64	0,00E+00	5 x 2	17 q25	tetraspanin 10 [Source:HGNC Symbol;Acc:HGNC:29942]	
NR4A3	0,59	12,62	0,00E+00	4 x 1	9 q31	nuclear receptor subfamily 4 group A member 3 [Source:HGNC Symbol;Acc:HGNC:7982]	
YAP1	0,58	12,59	0,00E+00	1 x 2	11 q22	Yes associated protein 1 [Source:HGNC Symbol;Acc:HGNC:16262]	
GNS	0,58	12,54	0,00E+00	1 x 3	12 q14	glucosamine (N-acetyl)-6-sulfatase [Source:HGNC Symbol;Acc:HGNC:4422]	
	0,58	12,48	0,00E+00	5 x 1			
ATP5A1	0,58	12,43	0,00E+00	6 x 3	18 q21	ATP synthase, H+ transporting, mitochondrial F1 complex, alpha subunit 1, cardiac muscle [Source:HGNC Symbol;Acc:HGNC:823]	
FAM210A	0,58	12,41	0,00E+00	3 x 1	18 p11	family with sequence similarity 210 member A [Source:HGNC Symbol;Acc:HGNC:28346]	
NQO1	0,58	12,41	0,00E+00	1 x 3	16 q22	NAD(P)H quinone dehydrogenase 1 [Source:HGNC Symbol;Acc:HGNC:2874]	
ABCB5	0,58	12,40	0,00E+00	1 x 1	7 p21	ATP binding cassette subfamily B member 5 [Source:HGNC Symbol;Acc:HGNC:46]	
PTTG1IP	0,58	12,40	0,00E+00	1 x 5	21 q22	pituitary tumor-transforming 1 interacting protein [Source:HGNC Symbol;Acc:HGNC:13524]	
PSAT1	0,58	12,39	0,00E+00	1 x 1	9 q21	phosphoserine aminotransferase 1 [Source:HGNC Symbol;Acc:HGNC:19129]	
PIR	0,58	12,36	0,00E+00	1 x 1	X p22	pirin [Source:HGNC Symbol;Acc:HGNC:30048]	
CTSL	0,58	12,35	0,00E+00	1 x 2	9 q21	cathepsin L [Source:HGNC Symbol;Acc:HGNC:2537]	
FNIP2	0,57	12,27	0,00E+00	5 x 1	4 q32	folliculin interacting protein 2 [Source:HGNC Symbol;Acc:HGNC:29280]	
HNRNPF	0,57	12,24	0,00E+00	1 x 2	10 q11	heterogeneous nuclear ribonucleoprotein F [Source:HGNC Symbol;Acc:HGNC:5039]	
MLPH	0,57	12,24	0,00E+00	1 x 1	2 q37	melanophilin [Source:HGNC Symbol;Acc:HGNC:29643]	
PDE3A	0,57	12,22	0,00E+00	4 x 1	12 p12	phosphodiesterase 3A [Source:HGNC Symbol;Acc:HGNC:8778]	
ANKRD49	0,57	12,18	0,00E+00	3 x 1	11 q21	ankyrin repeat domain 49 [Source:HGNC Symbol;Acc:HGNC:25970]	
SEMA6D	0,57	12,12	0,00E+00	4 x 1	15 q21	semaphorin 6D [Source:HGNC Symbol;Acc:HGNC:16770]	
SOD1	0,57	12,04	0,00E+00	6 x 9	21 q22	superoxide dismutase 1, soluble [Source:HGNC Symbol;Acc:HGNC:11179]	
BACE2	0,57	12,00	0,00E+00	1 x 3	21 q22	beta-site APP-cleaving enzyme 2 [Source:HGNC Symbol;Acc:HGNC:934]	
AVPI1	0,57	12,00	0,00E+00	1 x 3	10 q24	arginine vasopressin induced 1 [Source:HGNC Symbol;Acc:HGNC:30898]	
WSB1	0,57	11,99	0,00E+00	3 x 3	17 q11	WD repeat and SOCS box containing 1 [Source:HGNC Symbol;Acc:HGNC:19221]	
SLC16A6	0,56	11,94	0,00E+00	3 x 3	17 q24	solute carrier family 16 member 6 [Source:HGNC Symbol;Acc:HGNC:10927]	
SIRPA	0,56	11,94	0,00E+00	6 x 1	20 p13	signal regulatory protein alpha [Source:HGNC Symbol;Acc:HGNC:9662]	
EIF3CL	0,56	11,93	0,00E+00	1 x 7	16 p12	eukaryotic translation initiation factor 3 subunit C-like [Source:HGNC Symbol;Acc:HGNC:26347]	
CD40	0,56	11,92	0,00E+00	4 x 1	20 q13	CD40 molecule [Source:HGNC Symbol;Acc:HGNC:11919]	
PRMT2	0,56	11,88	0,00E+00	1 x 3	21 q22	protein arginine methyltransferase 2 [Source:HGNC Symbol;Acc:HGNC:5186]	
ATP5J	0,56	11,84	0,00E+00	9 x 3	21 q21	ATP synthase, H+ transporting, mitochondrial Fo complex subunit F6 [Source:HGNC Symbol;Acc:HGNC:847]	
CHCHD3	0,56	11,81	0,00E+00	1 x 5	7 q33	coiled-coil-helix-coiled-coil-helix domain containing 3 [Source:HGNC Symbol;Acc:HGNC:21906]	
NPLOC4	0,56	11,80	0,00E+00	1 x 4	17 q25	NPL4 homolog, ubiquitin recognition factor [Source:HGNC Symbol;Acc:HGNC:18261]	
HOXD1	0,56	11,76	0,00E+00	5 x 1	2 q31	homeobox D1 [Source:HGNC Symbol;Acc:HGNC:5132]	
ANAPC15	0,56	11,73	0,00E+00	3 x 1	11 q13	anaphase promoting complex subunit 15 [Source:HGNC Symbol;Acc:HGNC:24531]	


PEMT	0,56	11,70	0,00E+00	1 x 3	17 p11	phosphatidylethanolamine N-methyltransferase [Source:HGNC Symbol;Acc:HGNC:8830]	
IVNS1ABP	0,55	11,65	0,00E+00	3 x 1	1 q25	influenza virus NS1A binding protein [Source:HGNC Symbol;Acc:HGNC:16951]	
KCNAB2	0,55	11,64	0,00E+00	5 x 1	1 p36	potassium voltage-gated channel subfamily A regulatory beta subunit 2 [Source:HGNC Symbol;Acc:HGNC:6229]	
NUDT3	0,55	11,58	0,00E+00	4 x 1	6 p21	nudix hydrolase 3 [Source:HGNC Symbol;Acc:HGNC:8050]	
MAZ	0,55	11,58	0,00E+00	1 x 4	16 p11	MYC associated zinc finger protein [Source:HGNC Symbol;Acc:HGNC:6914]	
TPCN2	0,55	11,54	0,00E+00	4 x 2	11 q13	two pore segment channel 2 [Source:HGNC Symbol;Acc:HGNC:20820]	
GALNT3	0,55	11,53	0,00E+00	3 x 1	2 q24	polypeptide N-acetylgalactosaminyltransferase 3 [Source:HGNC Symbol;Acc:HGNC:4125]	
DCT	0,55	11,50	0,00E+00	1 x 1	13 q32	dopachrome tautomerase [Source:HGNC Symbol;Acc:HGNC:2709]	
NDUFS6	0,55	11,43	0,00E+00	3 x 5	5 p15	NADH:ubiquinone oxidoreductase subunit S6 [Source:HGNC Symbol;Acc:HGNC:7713]	
GADD45B	0,55	11,41	0,00E+00	1 x 3	19 p13	growth arrest and DNA damage inducible beta [Source:HGNC Symbol;Acc:HGNC:4096]	
FAM167B	0,55	11,39	0,00E+00	1 x 5	1 p35	family with sequence similarity 167 member B [Source:HGNC Symbol;Acc:HGNC:28133]	
DOK5	0,55	11,38	0,00E+00	1 x 1	20 q13	docking protein 5 [Source:HGNC Symbol;Acc:HGNC:16173]	
HDDC2	0,55	11,37	0,00E+00	3 x 1	6 q22	HD domain containing 2 [Source:HGNC Symbol;Acc:HGNC:21078]	
NACA2	0,54	11,32	0,00E+00	1 x 6	17 q23	nascent polypeptide associated complex alpha subunit 2 [Source:HGNC Symbol;Acc:HGNC:23290]	
MIA3	0,54	11,28	0,00E+00	1 x 6	1 q41	melanoma inhibitory activity family member 3 [Source:HGNC Symbol;Acc:HGNC:24008]	
DDX43	0,54	11,27	0,00E+00	3 x 2	6 q13	DEAD-box helicase 43 [Source:HGNC Symbol;Acc:HGNC:18677]	
GALNTL6	0,54	11,25	0,00E+00	5 x 1	4 q34	polypeptide N-acetylgalactosaminyltransferase-like 6 [Source:HGNC Symbol;Acc:HGNC:33844]	
NRN1	0,54	11,24	0,00E+00	1 x 4	6 p25	neuritin 1 [Source:HGNC Symbol;Acc:HGNC:17972]	
PRKAR1A	0,54	11,23	0,00E+00	3 x 5	17 q24	protein kinase cAMP-dependent type I regulatory subunit alpha [Source:HGNC Symbol;Acc:HGNC:9388]	
PSEN2	0,54	11,19	0,00E+00	3 x 2	1 q42	presenilin 2 [Source:HGNC Symbol;Acc:HGNC:9509]	
SLC18B1	0,54	11,19	0,00E+00	1 x 1	6 q23	solute carrier family 18 member B1 [Source:HGNC Symbol;Acc:HGNC:21573]	
SEC11C	0,54	11,17	0,00E+00	2 x 4	18 q21	SEC11 homolog C, signal peptidase complex subunit [Source:HGNC Symbol;Acc:HGNC:23400]	
HMGCS1	0,54	11,16	0,00E+00	3 x 1	5 p12	3-hydroxy-3-methylglutaryl-CoA synthase 1 [Source:HGNC Symbol;Acc:HGNC:5007]	
GNAI1	0,54	11,09	0,00E+00	1 x 2	7 q21	G protein subunit alpha i1 [Source:HGNC Symbol;Acc:HGNC:4384]	
SLC25A3	0,54	11,08	0,00E+00	1 x 6	12 q23	solute carrier family 25 member 3 [Source:HGNC Symbol;Acc:HGNC:10989]	
STX7	0,54	11,06	0,00E+00	1 x 1	6 q23	syntaxin 7 [Source:HGNC Symbol;Acc:HGNC:11442]	
CAND1	0,53	11,05	0,00E+00	2 x 2	12 q14	cullin associated and neddylation dissociated 1 [Source:HGNC Symbol;Acc:HGNC:30688]	
MET	0,53	11,02	0,00E+00	4 x 1	7 q31	MET proto-oncogene, receptor tyrosine kinase [Source:HGNC Symbol;Acc:HGNC:7029]	
PLP1	0,53	10,97	0,00E+00	1 x 1	X q22	proteolipid protein 1 [Source:HGNC Symbol;Acc:HGNC:9086]	
PFKM	0,53	10,95	0,00E+00	2 x 1	12 q13	phosphofructokinase, muscle [Source:HGNC Symbol;Acc:HGNC:8877]	
FASN	0,53	10,95	0,00E+00	1 x 6	17 q25	fatty acid synthase [Source:HGNC Symbol;Acc:HGNC:3594]	
HNRNPA1	0,53	10,94	0,00E+00	4 x 6	12 q13	heterogeneous nuclear ribonucleoprotein A1 [Source:HGNC Symbol;Acc:HGNC:5031]	
GPRC5B	0,53	10,91	0,00E+00	3 x 1	16 p12	G protein-coupled receptor class C group 5 member B [Source:HGNC Symbol;Acc:HGNC:13308]	


TKT	0,53	10,91	0,00E+00	1 x 3	3 p21	transketolase [Source:HGNC Symbol;Acc:HGNC:11834]	
ASRGL1	0,53	10,90	0,00E+00	1 x 3	11 q12	asparaginase like 1 [Source:HGNC Symbol;Acc:HGNC:16448]	
TYR	0,53	10,84	0,00E+00	1 x 1	11 q14	tyrosinase [Source:HGNC Symbol;Acc:HGNC:12442]	
MOK	0,53	10,80	0,00E+00	1 x 4	14 q32	MOK protein kinase [Source:HGNC Symbol;Acc:HGNC:9833]	
TMEM251	0,53	10,80	0,00E+00	3 x 1	CHR_HSCHR14	transmembrane protein 251 [Source:HGNC Symbol;Acc:HGNC:20218]	
UQCR10	0,53	10,78	0,00E+00	5 x 3	22 q12	ubiquinol-cytochrome c reductase, complex III subunit X [Source:HGNC Symbol;Acc:HGNC:30863]	
CS	0,53	10,78	0,00E+00	4 x 3	12 q13	citrate synthase [Source:HGNC Symbol;Acc:HGNC:2422]	
CDKN2A	0,52	10,72	0,00E+00	2 x 5	9 p21	cyclin dependent kinase inhibitor 2A [Source:HGNC Symbol;Acc:HGNC:1787]	
ARL2BP	0,52	10,72	0,00E+00	1 x 6	16 q13	ADP ribosylation factor like GTPase 2 binding protein [Source:HGNC Symbol;Acc:HGNC:17146]	
FLII	0,52	10,69	0,00E+00	1 x 3	17 p11	FLII, actin remodeling protein [Source:HGNC Symbol;Acc:HGNC:3750]	
PPHLN1	0,52	10,67	0,00E+00	3 x 2	12 q12	periphilin 1 [Source:HGNC Symbol;Acc:HGNC:19369]	
EEF1B2	0,52	10,66	0,00E+00	1 x 6	CHR_HSCHR2_	eukaryotic translation elongation factor 1 beta 2 [Source:HGNC Symbol;Acc:HGNC:3208]	
RTFDC1	0,52	10,64	0,00E+00	4 x 4	20 q13	replication termination factor 2 domain containing 1 [Source:HGNC Symbol;Acc:HGNC:15890]	
ACAT1	0,52	10,63	0,00E+00	1 x 4	11 q22	acetyl-CoA acetyltransferase 1 [Source:HGNC Symbol;Acc:HGNC:93]	
MARCH6	0,52	10,62	0,00E+00	6 x 2	5 p15	membrane associated ring-CH-type finger 6 [Source:HGNC Symbol;Acc:HGNC:30550]	
ALDH9A1	0,52	10,62	0,00E+00	1 x 3	1 q24	aldehyde dehydrogenase 9 family member A1 [Source:HGNC Symbol;Acc:HGNC:412]	
QARS	0,52	10,61	0,00E+00	1 x 3	3 p21	glutaminyl-tRNA synthetase [Source:HGNC Symbol;Acc:HGNC:9751]	
CBX1	0,52	10,61	0,00E+00	4 x 3	17 q21	chromobox 1 [Source:HGNC Symbol;Acc:HGNC:1551]	
JRKL	0,52	10,57	0,00E+00	4 x 1	11 q21	JRK-like [Source:HGNC Symbol;Acc:HGNC:6200]	
C14orf2	0,52	10,54	0,00E+00	3 x 7	14 q32	chromosome 14 open reading frame 2 [Source:HGNC Symbol;Acc:HGNC:1188]	
SLC25A39	0,52	10,54	0,00E+00	1 x 4	17 q21	solute carrier family 25 member 39 [Source:HGNC Symbol;Acc:HGNC:24279]	
QPRT	0,52	10,54	0,00E+00	1 x 5	16 p11	quinolinate phosphoribosyltransferase [Source:HGNC Symbol;Acc:HGNC:9755]	
ENDOV	0,51	10,49	0,00E+00	1 x 4	17 q25	endonuclease V [Source:HGNC Symbol;Acc:HGNC:26640]	
OSBPL2	0,51	10,47	0,00E+00	4 x 1	20 q13	oxysterol binding protein like 2 [Source:HGNC Symbol;Acc:HGNC:15761]	
	0,51	10,43	0,00E+00	3 x 8			
PPME1	0,51	10,42	0,00E+00	3 x 1	11 q13	protein phosphatase methylesterase 1 [Source:HGNC Symbol;Acc:HGNC:30178]	
MRPS25	0,51	10,40	0,00E+00	3 x 1	3 p25	mitochondrial ribosomal protein S25 [Source:HGNC Symbol;Acc:HGNC:14511]	
ACAA2	0,51	10,35	0,00E+00	4 x 1	18 q21	acetyl-CoA acyltransferase 2 [Source:HGNC Symbol;Acc:HGNC:83]	
EDNRB	0,51	10,34	0,00E+00	2 x 1	13 q22	endothelin receptor type B [Source:HGNC Symbol;Acc:HGNC:3180]	
ASPSCR1	0,51	10,32	0,00E+00	1 x 5	17 q25	ASPSCR1, UBX domain containing tether for SLC2A4 [Source:HGNC Symbol;Acc:HGNC:13825]	
PGAM1	0,51	10,30	0,00E+00	1 x 7	10 q24	phosphoglycerate mutase 1 [Source:HGNC Symbol;Acc:HGNC:8888]	
TUBA1C	0,51	10,30	0,00E+00	1 x 5	12 q13	tubulin alpha 1c [Source:HGNC Symbol;Acc:HGNC:20768]	
PEPD	0,51	10,29	0,00E+00	1 x 4	19 q13	peptidase D [Source:HGNC Symbol;Acc:HGNC:8840]	


IDH3A	0,51	10,28	0,00E+00	3 x 1	15 q25	isocitrate dehydrogenase 3 (NAD(+)) alpha [Source:HGNC Symbol;Acc:HGNC:5384]	
TMBIM4	0,51	10,27	0,00E+00	5 x 4	12 q14	transmembrane BAX inhibitor motif containing 4 [Source:HGNC Symbol;Acc:HGNC:24257]	
SUB1	0,51	10,25	0,00E+00	7 x 1	5 p13	SUB1 homolog, transcriptional regulator [Source:HGNC Symbol;Acc:HGNC:19985]	
COX7B	0,51	10,25	0,00E+00	7 x 5	X q21	cytochrome c oxidase subunit 7B [Source:HGNC Symbol;Acc:HGNC:2291]	
PIK3CB	0,51	10,22	0,00E+00	1 x 3	3 q22	phosphatidylinositol-4,5-bisphosphate 3-kinase catalytic subunit beta [Source:HGNC Symbol;Acc:HGNC:8976]	
TOB1	0,50	10,17	0,00E+00	3 x 1	17 q21	transducer of ERBB2, 1 [Source:HGNC Symbol;Acc:HGNC:11979]	
STRADB	0,50	10,17	0,00E+00	4 x 1	2 q33	STE20-related kinase adaptor beta [Source:HGNC Symbol;Acc:HGNC:13205]	
GRN	0,50	10,14	0,00E+00	1 x 5	17 q21	granulin [Source:HGNC Symbol;Acc:HGNC:4601]	
FAM174B	0,50	10,14	0,00E+00	4 x 1	15 q26	family with sequence similarity 174 member B [Source:HGNC Symbol;Acc:HGNC:34339]	
PLXNC1	0,50	10,13	0,00E+00	5 x 1	12 q22	plexin C1 [Source:HGNC Symbol;Acc:HGNC:9106]	
NT5DC3	0,50	10,13	0,00E+00	5 x 1	12 q23	5'-nucleotidase domain containing 3 [Source:HGNC Symbol;Acc:HGNC:30826]	
MYH10	0,50	10,12	0,00E+00	1 x 3	17 p13	myosin, heavy chain 10, non-muscle [Source:HGNC Symbol;Acc:HGNC:7568]	
SLC25A13	0,50	10,10	0,00E+00	3 x 1	7 q21	solute carrier family 25 member 13 [Source:HGNC Symbol;Acc:HGNC:10983]	
CXADR	0,50	10,10	0,00E+00	5 x 1	21 q21	coxsackie virus and adenovirus receptor [Source:HGNC Symbol;Acc:HGNC:2559]	
AHCYL1	0,50	10,07	0,00E+00	5 x 1	1 p13	adenosylhomocysteinase like 1 [Source:HGNC Symbol;Acc:HGNC:344]	
COQ9	0,50	10,06	0,00E+00	1 x 3	16 q21	coenzyme Q9 [Source:HGNC Symbol;Acc:HGNC:25302]	
ZFYVE16	0,50	10,05	0,00E+00	3 x 2	5 q14	zinc finger FYVE-type containing 16 [Source:HGNC Symbol;Acc:HGNC:20756]	
PPFIA1	0,50	10,04	0,00E+00	1 x 3	11 q13	PTPRF interacting protein alpha 1 [Source:HGNC Symbol;Acc:HGNC:9245]	
	0,50	10,00	0,00E+00	1 x 9	6 q12	FK506 binding protein 1C [Source:HGNC Symbol;Acc:HGNC:21376]	
PPIC	0,50	10,00	0,00E+00	3 x 2	5 q23	peptidylprolyl isomerase C [Source:HGNC Symbol;Acc:HGNC:9256]	
TPP1	0,50	9,96	0,00E+00	3 x 3	11 p15	tripeptidyl peptidase 1 [Source:HGNC Symbol;Acc:HGNC:2073]	
HGS	0,49	9,94	0,00E+00	1 x 6	17 q25	hepatocyte growth factor-regulated tyrosine kinase substrate [Source:HGNC Symbol;Acc:HGNC:4897]	
NUPR1	0,49	9,93	0,00E+00	1 x 4	16 p11	nuclear protein 1, transcriptional regulator [Source:HGNC Symbol;Acc:HGNC:29990]	
FAM96A	0,49	9,92	0,00E+00	4 x 1	15 q22	family with sequence similarity 96 member A [Source:HGNC Symbol;Acc:HGNC:26235]	
POLR2J3	0,49	9,86	0,00E+00	1 x 6	7 q22	polymerase (RNA) II subunit J3 [Source:HGNC Symbol;Acc:HGNC:33853]	
GCSH	0,49	9,86	0,00E+00	1 x 7	16 q23	glycine cleavage system protein H [Source:HGNC Symbol;Acc:HGNC:4208]	
EIF4B	0,49	9,85	0,00E+00	5 x 1	12 q13	eukaryotic translation initiation factor 4B [Source:HGNC Symbol;Acc:HGNC:3285]	
HIGD2A	0,49	9,83	0,00E+00	3 x 2	5 q35	HIG1 hypoxia inducible domain family member 2A [Source:HGNC Symbol;Acc:HGNC:28311]	
HEXA	0,49	9,80	0,00E+00	3 x 4	15 q23	hexosaminidase subunit alpha [Source:HGNC Symbol;Acc:HGNC:4878]	
ARHGDIA	0,49	9,80	0,00E+00	4 x 4	17 q25	Rho GDP dissociation inhibitor alpha [Source:HGNC Symbol;Acc:HGNC:678]	
UQCRC1	0,49	9,78	0,00E+00	1 x 6	3 p21	ubiquinol-cytochrome c reductase core protein I [Source:HGNC Symbol;Acc:HGNC:12585]	
PELI2	0,49	9,77	0,00E+00	5 x 1	14 q22	pellino E3 ubiquitin protein ligase family member 2 [Source:HGNC Symbol;Acc:HGNC:8828]	
RFC5	0,49	9,76	0,00E+00	2 x 1	12 q24	replication factor C subunit 5 [Source:HGNC Symbol;Acc:HGNC:9973]	


NPC2	0,49	9,75	0,00E+00	1 x 5	14 q24	NPC intracellular cholesterol transporter 2 [Source:HGNC Symbol;Acc:HGNC:14537]	
COA4	0,49	9,75	0,00E+00	3 x 4	11 q13	cytochrome c oxidase assembly factor 4 homolog [Source:HGNC Symbol;Acc:HGNC:24604]	
	0,49	9,75	0,00E+00	3 x 4			
STRA13	0,49	9,73	0,00E+00	3 x 4	17 q25	stimulated by retinoic acid 13 [Source:HGNC Symbol;Acc:HGNC:11422]	
CEACAM1	0,49	9,72	0,00E+00	1 x 4	19 q13	carcinoembryonic antigen related cell adhesion molecule 1 [Source:HGNC Symbol;Acc:HGNC:1814]	
TRIM63	0,49	9,71	0,00E+00	1 x 7	1 p36	tripartite motif containing 63 [Source:HGNC Symbol;Acc:HGNC:16007]	
CHMP2B	0,49	9,69	0,00E+00	4 x 1	3 p11	charged multivesicular body protein 2B [Source:HGNC Symbol;Acc:HGNC:24537]	
MAPK10	0,48	9,67	0,00E+00	5 x 1	4 q21	mitogen-activated protein kinase 10 [Source:HGNC Symbol;Acc:HGNC:6872]	
ATP6V0D1	0,48	9,65	0,00E+00	1 x 5	16 q22	ATPase H+ transporting V0 subunit d1 [Source:HGNC Symbol;Acc:HGNC:13724]	
MRPL38	0,48	9,65	0,00E+00	1 x 6	17 q25	mitochondrial ribosomal protein L38 [Source:HGNC Symbol;Acc:HGNC:14033]	
TXN2	0,48	9,64	0,00E+00	3 x 1	22 q12	thioredoxin 2 [Source:HGNC Symbol;Acc:HGNC:17772]	
MRPS7	0,48	9,64	0,00E+00	3 x 4	17 q25	mitochondrial ribosomal protein S7 [Source:HGNC Symbol;Acc:HGNC:14499]	
MICAL1	0,48	9,62	0,00E+00	4 x 4	6 q21	microtubule associated monooxygenase, calponin and LIM domain containing 1 [Source:HGNC Symbol;Acc:HGNC:20619]	
TALDO1	0,48	9,61	0,00E+00	1 x 7	11 p15	transaldolase 1 [Source:HGNC Symbol;Acc:HGNC:11559]	
CTSA	0,48	9,61	0,00E+00	1 x 6	20 q13	cathepsin A [Source:HGNC Symbol;Acc:HGNC:9251]	
CDH3	0,48	9,59	0,00E+00	6 x 3	16 q22	cadherin 3 [Source:HGNC Symbol;Acc:HGNC:1762]	
RBPMS2	0,48	9,51	0,00E+00	6 x 1	15 q22	RNA binding protein with multiple splicing 2 [Source:HGNC Symbol;Acc:HGNC:19098]	
SLC9A3R1	0,48	9,51	0,00E+00	1 x 5	17 q25	SLC9A3 regulator 1 [Source:HGNC Symbol;Acc:HGNC:11075]	
SLC19A2	0,48	9,49	0,00E+00	5 x 1	1 q24	solute carrier family 19 member 2 [Source:HGNC Symbol;Acc:HGNC:10938]	
H3F3A	0,48	9,48	0,00E+00	1 x 7	1 q42	H3 histone, family 3A [Source:HGNC Symbol;Acc:HGNC:4764]	
UBXN1	0,48	9,45	0,00E+00	1 x 5	11 q12	UBX domain protein 1 [Source:HGNC Symbol;Acc:HGNC:18402]	
FLOT2	0,48	9,45	0,00E+00	1 x 5	17 q11	flotillin 2 [Source:HGNC Symbol;Acc:HGNC:3758]	
MIS18A	0,48	9,45	0,00E+00	2 x 2	21 q22	MIS18 kinetochore protein A [Source:HGNC Symbol;Acc:HGNC:1286]	
NCALD	0,48	9,43	0,00E+00	3 x 1	8 q22	neurocalcin delta [Source:HGNC Symbol;Acc:HGNC:7655]	
SLC35F2	0,47	9,42	0,00E+00	6 x 1	11 q22	solute carrier family 35 member F2 [Source:HGNC Symbol;Acc:HGNC:23615]	
TUB	0,47	9,40	0,00E+00	5 x 1	11 p15	tubby bipartite transcription factor [Source:HGNC Symbol;Acc:HGNC:12406]	
ILKAP	0,47	9,39	0,00E+00	1 x 4	2 q37	ILK associated serine/threonine phosphatase [Source:HGNC Symbol;Acc:HGNC:15566]	
U2AF1	0,47	9,37	0,00E+00	5 x 9	21 q22	U2 small nuclear RNA auxiliary factor 1 [Source:HGNC Symbol;Acc:HGNC:12453]	
MTRR	0,47	9,36	0,00E+00	1 x 4	5 p15	5-methyltetrahydrofolate-homocysteine methyltransferase reductase [Source:HGNC Symbol;Acc:HGNC:7473]	
FAM210B	0,47	9,35	0,00E+00	5 x 1	20 q13	family with sequence similarity 210 member B [Source:HGNC Symbol;Acc:HGNC:16102]	
RTTN	0,47	9,34	0,00E+00	4 x 1	18 q22	rotatin [Source:HGNC Symbol;Acc:HGNC:18654]	
SWAP70	0,47	9,33	0,00E+00	4 x 2	11 p15	SWAP switching B-cell complex 70kDa subunit [Source:HGNC Symbol;Acc:HGNC:17070]	
ZDHHC11	0,47	9,32	0,00E+00	3 x 4	5 p15	zinc finger DHHC-type containing 11 [Source:HGNC Symbol;Acc:HGNC:19158]	


TMEM223	0,47	9,32	0,00E+00	4 x 2	11 q12	transmembrane protein 223 [Source:HGNC Symbol;Acc:HGNC:28464]	
ZZZ3	0,47	9,31	0,00E+00	4 x 1	1 p31	zinc finger ZZ-type containing 3 [Source:HGNC Symbol;Acc:HGNC:24523]	
HERC3	0,47	9,30	0,00E+00	1 x 6	4 q22	HECT and RLD domain containing E3 ubiquitin protein ligase 3 [Source:HGNC Symbol;Acc:HGNC:4876]	
SIVA1	0,47	9,29	0,00E+00	1 x 3	14 q32	SIVA1 apoptosis inducing factor [Source:HGNC Symbol;Acc:HGNC:17712]	
SACM1L	0,47	9,28	0,00E+00	3 x 3	3 p21	SAC1 suppressor of actin mutations 1-like (yeast) [Source:HGNC Symbol;Acc:HGNC:17059]	
GABRB3	0,47	9,27	0,00E+00	6 x 3	15 q12	gamma-aminobutyric acid type A receptor beta3 subunit [Source:HGNC Symbol;Acc:HGNC:4083]	
SLC29A1	0,47	9,25	0,00E+00	4 x 1	6 p21	solute carrier family 29 member 1 (Augustine blood group) [Source:HGNC Symbol;Acc:HGNC:11003]	
C2CD3	0,47	9,25	0,00E+00	3 x 4	11 q13	C2 calcium dependent domain containing 3 [Source:HGNC Symbol;Acc:HGNC:24564]	
TFAP2A	0,47	9,25	0,00E+00	6 x 2	6 p24	transcription factor AP-2 alpha [Source:HGNC Symbol;Acc:HGNC:11742]	
APIP	0,47	9,24	0,00E+00	5 x 4	11 p13	APAF1 interacting protein [Source:HGNC Symbol;Acc:HGNC:17581]	
SEMA5A	0,47	9,23	0,00E+00	6 x 1	5 p15	semaphorin 5A [Source:HGNC Symbol;Acc:HGNC:10736]	
CDK4	0,47	9,22	0,00E+00	1 x 5	12 q14	cyclin dependent kinase 4 [Source:HGNC Symbol;Acc:HGNC:1773]	
BPTF	0,47	9,19	0,00E+00	5 x 4	17 q24	bromodomain PHD finger transcription factor [Source:HGNC Symbol;Acc:HGNC:3581]	
VEGFB	0,47	9,18	0,00E+00	1 x 4	11 q13	vascular endothelial growth factor B [Source:HGNC Symbol;Acc:HGNC:12681]	
DAAM1	0,46	9,17	0,00E+00	5 x 1	14 q23	dishevelled associated activator of morphogenesis 1 [Source:HGNC Symbol;Acc:HGNC:18142]	
STARD10	0,46	9,16	0,00E+00	3 x 5	11 q13	StAR related lipid transfer domain containing 10 [Source:HGNC Symbol;Acc:HGNC:10666]	
ELP5	0,46	9,13	0,00E+00	3 x 1	17 p13	elongator acetyltransferase complex subunit 5 [Source:HGNC Symbol;Acc:HGNC:30617]	
LPIN3	0,46	9,13	0,00E+00	6 x 1	20 q12	lipin 3 [Source:HGNC Symbol;Acc:HGNC:14451]	
UGCG	0,46	9,13	0,00E+00	5 x 1	9 q31	UDP-glucose ceramide glucosyltransferase [Source:HGNC Symbol;Acc:HGNC:12524]	
CAPG	0,46	9,12	0,00E+00	5 x 1	2 p11	capping actin protein, gelsolin like [Source:HGNC Symbol;Acc:HGNC:1474]	
RASGRP3	0,46	9,11	0,00E+00	6 x 1	2 p22	RAS guanyl releasing protein 3 [Source:HGNC Symbol;Acc:HGNC:14545]	
RAB6A	0,46	9,11	0,00E+00	4 x 5	11 q13	RAB6A, member RAS oncogene family [Source:HGNC Symbol;Acc:HGNC:9786]	
DYNC1H1	0,46	9,09	0,00E+00	4 x 5	14 q32	dynein cytoplasmic 1 heavy chain 1 [Source:HGNC Symbol;Acc:HGNC:2961]	
ENO3	0,46	9,09	0,00E+00	2 x 3	17 p13	enolase 3 [Source:HGNC Symbol;Acc:HGNC:3354]	
TWSG1	0,46	9,04	0,00E+00	5 x 1	18 p11	twisted gastrulation BMP signaling modulator 1 [Source:HGNC Symbol;Acc:HGNC:12429]	
RHOG	0,46	9,04	0,00E+00	1 x 4	11 p15	ras homolog family member G [Source:HGNC Symbol;Acc:HGNC:672]	
TMEM98	0,46	9,02	0,00E+00	3 x 2	17 q11	transmembrane protein 98 [Source:HGNC Symbol;Acc:HGNC:24529]	
RXRG	0,46	8,96	0,00E+00	5 x 1	1 q23	retinoid X receptor gamma [Source:HGNC Symbol;Acc:HGNC:10479]	
TPD52	0,46	8,95	0,00E+00	4 x 1	8 q21	tumor protein D52 [Source:HGNC Symbol;Acc:HGNC:12005]	
NCOA4	0,46	8,95	0,00E+00	4 x 3	10 q11	nuclear receptor coactivator 4 [Source:HGNC Symbol;Acc:HGNC:7671]	
GDPD5	0,46	8,93	0,00E+00	6 x 2	11 q13	glycerophosphodiester phosphodiesterase domain containing 5 [Source:HGNC Symbol;Acc:HGNC:28804]	
UVRAG	0,46	8,93	0,00E+00	1 x 5	11 q13	UV radiation resistance associated [Source:HGNC Symbol;Acc:HGNC:12640]	
SOAT1	0,46	8,93	0,00E+00	5 x 1	1 q25	sterol O-acyltransferase 1 [Source:HGNC Symbol;Acc:HGNC:11177]	


M6PR	0,45	8,92	0	1 x 6	12 p13	mannose-6-phosphate receptor, cation dependent [Source:HGNC Symbol;Acc:HGNC:6752]	
PYCR1	0,45	8,91	0	1 x 6	17 q25	pyrroline-5-carboxylate reductase 1 [Source:HGNC Symbol;Acc:HGNC:9721]	
PI4KB	0,45	8,90	0	1 x 4	1 q21	phosphatidylinositol 4-kinase beta [Source:HGNC Symbol;Acc:HGNC:8984]	
SGK1	0,45	8,89	0	1 x 4	6 q23	serum/glucocorticoid regulated kinase 1 [Source:HGNC Symbol;Acc:HGNC:10810]	
MRPL34	0,45	8,89	0	1 x 8	19 p13	mitochondrial ribosomal protein L34 [Source:HGNC Symbol;Acc:HGNC:14488]	
ZNF330	0,45	8,89	0	3 x 3	4 q31	zinc finger protein 330 [Source:HGNC Symbol;Acc:HGNC:15462]	
RAB5B	0,45	8,86	0	6 x 2	12 q13	RAB5B, member RAS oncogene family [Source:HGNC Symbol;Acc:HGNC:9784]	
GPR19	0,45	8,83	0	4 x 1	12 p13	G protein-coupled receptor 19 [Source:HGNC Symbol;Acc:HGNC:4473]	
ARF6	0,45	8,81	0	7 x 1	14 q21	ADP ribosylation factor 6 [Source:HGNC Symbol;Acc:HGNC:659]	
LSS	0,45	8,79	0	4 x 5	CHR_HSCHR21	lanosterol synthase (2,3-oxidosqualene-lanosterol cyclase) [Source:HGNC Symbol;Acc:HGNC:6708]	
NELFCD	0,45	8,79	0	4 x 4	20 q13	negative elongation factor complex member C/D [Source:HGNC Symbol;Acc:HGNC:15934]	
TSG101	0,45	8,77	0	1 x 5	11 p15	tumor susceptibility 101 [Source:HGNC Symbol;Acc:HGNC:15971]	
TOM1	0,45	8,76	0	4 x 4	22 q12	target of myb1 membrane trafficking protein [Source:HGNC Symbol;Acc:HGNC:11982]	
ALDOC	0,45	8,74	0	4 x 2	17 q11	aldolase, fructose-bisphosphate C [Source:HGNC Symbol;Acc:HGNC:418]	
COQ10A	0,45	8,74	0	4 x 1	12 q13	coenzyme Q10A [Source:HGNC Symbol;Acc:HGNC:26515]	
MST1R	0,45	8,73	1,1102E-16	1 x 6	3 p21	macrophage stimulating 1 receptor [Source:HGNC Symbol;Acc:HGNC:7381]	
SERPINB1	0,45	8,73	1,1102E-16	5 x 1	6 p25	serpin family B member 1 [Source:HGNC Symbol;Acc:HGNC:3311]	
GSTM4	0,45	8,72	1,1102E-16	2 x 3	1 p13	glutathione S-transferase mu 4 [Source:HGNC Symbol;Acc:HGNC:4636]	
SLC37A1	0,45	8,70	1,1102E-16	6 x 4	21 q22	solute carrier family 37 member 1 [Source:HGNC Symbol;Acc:HGNC:11024]	
RNF121	0,45	8,69	1,1102E-16	2 x 5	11 q13	ring finger protein 121 [Source:HGNC Symbol;Acc:HGNC:21070]	
OXA1L	0,45	8,69	1,1102E-16	4 x 1	14 q11	OXA1L, mitochondrial inner membrane protein [Source:HGNC Symbol;Acc:HGNC:8526]	
GPR89B	0,44	8,67	1,1102E-16	2 x 7	1 q21	G protein-coupled receptor 89B [Source:HGNC Symbol;Acc:HGNC:13840]	
KRTAP19-1	0,44	8,67	1,1102E-16	5 x 3	21 q22	keratin associated protein 19-1 [Source:HGNC Symbol;Acc:HGNC:18936]	
ZNF595	0,44	8,66	1,1102E-16	4 x 5	4 p16	zinc finger protein 595 [Source:HGNC Symbol;Acc:HGNC:27196]	
HSPA9	0,44	8,66	2,2204E-16	1 x 8	5 q31	heat shock protein family A (Hsp70) member 9 [Source:HGNC Symbol;Acc:HGNC:5244]	
PCYT2	0,44	8,66	2,2204E-16	1 x 6	17 q25	phosphate cytidylyltransferase 2, ethanolamine [Source:HGNC Symbol;Acc:HGNC:8756]	
GLUL	0,44	8,65	2,2204E-16	4 x 1	1 q25	glutamate-ammonia ligase [Source:HGNC Symbol;Acc:HGNC:4341]	
DSTYK	0,44	8,65	2,2204E-16	6 x 1	1 q32	dual serine/threonine and tyrosine protein kinase [Source:HGNC Symbol;Acc:HGNC:29043]	
CCDC88C	0,44	8,64	2,2204E-16	1 x 6	14 q32	coiled-coil domain containing 88C [Source:HGNC Symbol;Acc:HGNC:19967]	
TMEM11	0,44	8,63	2,2204E-16	1 x 4	17 p11	transmembrane protein 11 [Source:HGNC Symbol;Acc:HGNC:16823]	
SLC25A44	0,44	8,62	2,2204E-16	3 x 4	1 q22	solute carrier family 25 member 44 [Source:HGNC Symbol;Acc:HGNC:29036]	
RASSF3	0,44	8,60	2,2204E-16	6 x 1	12 q14	Ras association domain family member 3 [Source:HGNC Symbol;Acc:HGNC:14271]	
SUGP2	0,44	8,57	2,2204E-16	2 x 5	19 p13	SURP and G-patch domain containing 2 [Source:HGNC Symbol;Acc:HGNC:18641]	


KIF17	0,44	8,55	3,3307E-16	5 x 4	1 p36	kinesin family member 17 [Source:HGNC Symbol;Acc:HGNC:19167]	
THOC3	0,44	8,54	3,3307E-16	1 x 6	5 q35	THO complex 3 [Source:HGNC Symbol;Acc:HGNC:19072]	
SCIN	0,44	8,53	3,3307E-16	5 x 1	7 p21	scinderin [Source:HGNC Symbol;Acc:HGNC:21695]	
XRRA1	0,44	8,52	3,3307E-16	5 x 2	11 q13	X-ray radiation resistance associated 1 [Source:HGNC Symbol;Acc:HGNC:18868]	
C5orf22	0,44	8,50	4,4409E-16	5 x 1	5 p13	chromosome 5 open reading frame 22 [Source:HGNC Symbol;Acc:HGNC:25639]	
C10orf11	0,44	8,50	4,4409E-16	1 x 4	10 q22	chromosome 10 open reading frame 11 [Source:HGNC Symbol;Acc:HGNC:23405]	
SORBS1	0,44	8,47	4,4409E-16	5 x 1	10 q24	sorbin and SH3 domain containing 1 [Source:HGNC Symbol;Acc:HGNC:14565]	
CLPB	0,44	8,46	5,5511E-16	1 x 6	11 q13	ClpB homolog, mitochondrial AAA ATPase chaperonin [Source:HGNC Symbol;Acc:HGNC:30664]	
NPIPB5	0,44	8,46	5,5511E-16	5 x 8	16 p12	nuclear pore complex interacting protein family member B5 [Source:HGNC Symbol;Acc:HGNC:37233]	
TFRC	0,43	8,43	6,6613E-16	4 x 6	3 q29	transferrin receptor [Source:HGNC Symbol;Acc:HGNC:11763]	
	0,43	8,40	8,8818E-16	1 x 10			
SS18L1	0,43	8,38	9,992E-16	6 x 1	20 q13	SS18L1, nBAF chromatin remodeling complex subunit [Source:HGNC Symbol;Acc:HGNC:15592]	
	0,43	8,38	9,992E-16	2 x 8			
HADH	0,43	8,37	1,1102E-15	3 x 2	4 q25	hydroxyacyl-CoA dehydrogenase [Source:HGNC Symbol;Acc:HGNC:4799]	
CANX	0,43	8,36	1,1102E-15	5 x 9	5 q35	calnexin [Source:HGNC Symbol;Acc:HGNC:1473]	
ENPP2	0,43	8,33	1,3323E-15	6 x 5	8 q24	ectonucleotide pyrophosphatase/phosphodiesterase 2 [Source:HGNC Symbol;Acc:HGNC:3357]	
	0,43	8,33	1,3323E-15	4 x 7	16 p12	nuclear pore complex interacting protein family member B3 [Source:HGNC Symbol;Acc:HGNC:28989]	
ZFAND5	0,43	8,33	1,4433E-15	6 x 1	9 q21	zinc finger AN1-type containing 5 [Source:HGNC Symbol;Acc:HGNC:13008]	
ATP6V0A1	0,43	8,31	1,5543E-15	1 x 4	17 q21	ATPase H+ transporting V0 subunit a1 [Source:HGNC Symbol;Acc:HGNC:865]	
CHURC1-FNTB	0,43	8,31	1,5543E-15	1 x 6	14 q23	CHURC1-FNTB readthrough [Source:HGNC Symbol;Acc:HGNC:42960]	
NUMA1	0,43	8,26	2,2204E-15	3 x 3	11 q13	nuclear mitotic apparatus protein 1 [Source:HGNC Symbol;Acc:HGNC:8059]	
SNRPD1	0,43	8,25	2,4425E-15	1 x 9	18 q11	small nuclear ribonucleoprotein D1 polypeptide [Source:HGNC Symbol;Acc:HGNC:11158]	
PSMC1	0,43	8,25	2,4425E-15	4 x 7	14 q32	proteasome 26S subunit, ATPase 1 [Source:HGNC Symbol;Acc:HGNC:9547]	
IPO7	0,43	8,23	2,6645E-15	5 x 5	11 p15	importin 7 [Source:HGNC Symbol;Acc:HGNC:9852]	
OSGIN1	0,43	8,23	2,6645E-15	1 x 5	16 q23	oxidative stress induced growth inhibitor 1 [Source:HGNC Symbol;Acc:HGNC:30093]	
ST6GALNAC2	0,43	8,23	2,7756E-15	4 x 1	17 q25	ST6 N-acetylgalactosaminide alpha-2,6-sialyltransferase 2 [Source:HGNC Symbol;Acc:HGNC:10867]	
GNAS	0,43	8,23	2,8866E-15	5 x 7	20 q13	GNAS complex locus [Source:HGNC Symbol;Acc:HGNC:4392]	
PGAM4	0,43	8,22	2,8866E-15	1 x 8	X q21	phosphoglycerate mutase family member 4 [Source:HGNC Symbol;Acc:HGNC:21731]	
ACY1	0,43	8,21	3,1086E-15	4 x 1	3 p21	aminoacylase 1 [Source:HGNC Symbol;Acc:HGNC:177]	
EIF3M	0,43	8,20	3,3307E-15	1 x 10	11 p13	eukaryotic translation initiation factor 3 subunit M [Source:HGNC Symbol;Acc:HGNC:24460]	
FADS1	0,42	8,19	3,7748E-15	5 x 4	11 q12	fatty acid desaturase 1 [Source:HGNC Symbol;Acc:HGNC:3574]	
COX5A	0,42	8,18	3,9968E-15	1 x 10	15 q24	cytochrome c oxidase subunit 5A [Source:HGNC Symbol;Acc:HGNC:2267]	
DAZAP2	0,42	8,17	3,9968E-15	4 x 5	12 q13	DAZ associated protein 2 [Source:HGNC Symbol;Acc:HGNC:2684]	


COPE	0,42	8,17	4,2188E-15	1 x 8	19 p13	coatomer protein complex subunit epsilon [Source:HGNC Symbol;Acc:HGNC:2234]	
SLC7A8	0,42	8,17	4,2188E-15	4 x 4	14 q11	solute carrier family 7 member 8 [Source:HGNC Symbol;Acc:HGNC:11066]	
FBXW5	0,42	8,16	4,6629E-15	1 x 8	9 q34	F-box and WD repeat domain containing 5 [Source:HGNC Symbol;Acc:HGNC:13613]	
TSPAN3	0,42	8,15	4,6629E-15	1 x 8	15 q24	tetraspanin 3 [Source:HGNC Symbol;Acc:HGNC:17752]	
PTRHD1	0,42	8,15	4,6629E-15	2 x 4	2 p23	peptidyl-tRNA hydrolase domain containing 1 [Source:HGNC Symbol;Acc:HGNC:33782]	
P2RX4	0,42	8,14	4,885E-15	1 x 5	12 q24	purinergic receptor P2X 4 [Source:HGNC Symbol;Acc:HGNC:8535]	
PSMD12	0,42	8,14	5,107E-15	5 x 10	17 q24	proteasome 26S subunit, non-ATPase 12 [Source:HGNC Symbol;Acc:HGNC:9557]	
CCNG1	0,42	8,12	5,8842E-15	4 x 2	5 q34	cyclin G1 [Source:HGNC Symbol;Acc:HGNC:1592]	
PDHB	0,42	8,12	5,9952E-15	4 x 5	3 p14	pyruvate dehydrogenase (lipoamide) beta [Source:HGNC Symbol;Acc:HGNC:8808]	
PARP1	0,42	8,10	6,6613E-15	3 x 5	1 q42	poly(ADP-ribose) polymerase 1 [Source:HGNC Symbol;Acc:HGNC:270]	
EID1	0,42	8,09	7,2164E-15	2 x 8	15 q21	EP300 interacting inhibitor of differentiation 1 [Source:HGNC Symbol;Acc:HGNC:1191]	
POR	0,42	8,08	7,5495E-15	1 x 5	7 q11	cytochrome p450 oxidoreductase [Source:HGNC Symbol;Acc:HGNC:9208]	
MPC2	0,42	8,07	8,1046E-15	4 x 4	1 q24	mitochondrial pyruvate carrier 2 [Source:HGNC Symbol;Acc:HGNC:24515]	
SHMT1	0,42	8,06	8,4377E-15	1 x 4	17 p11	serine hydroxymethyltransferase 1 [Source:HGNC Symbol;Acc:HGNC:10850]	
IGSF11	0,42	8,06	8,7708E-15	7 x 6	3 q13	immunoglobulin superfamily member 11 [Source:HGNC Symbol;Acc:HGNC:16669]	
TXNRD1	0,42	8,04	1,0214E-14	1 x 5	12 q23	thioredoxin reductase 1 [Source:HGNC Symbol;Acc:HGNC:12437]	
VPS53	0,42	8,02	1,1213E-14	5 x 4	17 p13	VPS53, GARP complex subunit [Source:HGNC Symbol;Acc:HGNC:25608]	
STIP1	0,42	8,01	1,1879E-14	1 x 6	11 q13	stress induced phosphoprotein 1 [Source:HGNC Symbol;Acc:HGNC:11387]	
SVIP	0,42	8,01	1,2323E-14	5 x 1	11 p14	small VCP/p97-interacting protein [Source:HGNC Symbol;Acc:HGNC:25238]	
SFXN1	0,42	8,01	1,2657E-14	6 x 1	5 q35	sideroflexin 1 [Source:HGNC Symbol;Acc:HGNC:16085]	
PTTG2	0,42	8,00	1,3434E-14	2 x 8	4 p14	pituitary tumor-transforming 2 [Source:HGNC Symbol;Acc:HGNC:9691]	
DDX49	0,42	7,99	1,41E-14	1 x 6	19 p13	DEAD-box helicase 49 [Source:HGNC Symbol;Acc:HGNC:18684]	
UBE2G2	0,42	7,98	1,4766E-14	5 x 6	21 q22	ubiquitin conjugating enzyme E2 G2 [Source:HGNC Symbol;Acc:HGNC:12483]	
MTX2	0,41	7,96	1,6542E-14	1 x 5	2 q31	metaxin 2 [Source:HGNC Symbol;Acc:HGNC:7506]	
ATP11A	0,41	7,96	1,6875E-14	7 x 2	13 q34	ATPase phospholipid transporting 11A [Source:HGNC Symbol;Acc:HGNC:13552]	
NDUFB5	0,41	7,96	1,7097E-14	2 x 7	3 q26	NADH:ubiquinone oxidoreductase subunit B5 [Source:HGNC Symbol;Acc:HGNC:7700]	
TDRD9	0,41	7,95	1,8541E-14	7 x 2	14 q32	tudor domain containing 9 [Source:HGNC Symbol;Acc:HGNC:20122]	
RNF144B	0,41	7,95	1,8652E-14	6 x 1	6 p22	ring finger protein 144B [Source:HGNC Symbol;Acc:HGNC:21578]	
HSD17B14	0,41	7,94	1,9207E-14	4 x 7	19 q13	hydroxysteroid 17-beta dehydrogenase 14 [Source:HGNC Symbol;Acc:HGNC:23238]	
NUP160	0,41	7,93	2,065E-14	3 x 4	11 p11	nucleoporin 160 [Source:HGNC Symbol;Acc:HGNC:18017]	
IFRD2	0,41	7,92	2,2871E-14	1 x 5	3 p21	interferon-related developmental regulator 2 [Source:HGNC Symbol;Acc:HGNC:5457]	
ENTPD1	0,41	7,91	2,3315E-14	7 x 1	10 q24	ectonucleoside triphosphate diphosphohydrolase 1 [Source:HGNC Symbol;Acc:HGNC:3363]	
PDCD4	0,41	7,90	2,609E-14	5 x 1	10 q25	programmed cell death 4 (neoplastic transformation inhibitor) [Source:HGNC Symbol;Acc:HGNC:8763]	


C5orf51	0,41	7,88	2,9421E-14	5 x 1	5 p13	chromosome 5 open reading frame 51 [Source:HGNC Symbol;Acc:HGNC:27750]	
TBC1D16	0,41	7,88	2,9754E-14	6 x 4	17 q25	TBC1 domain family member 16 [Source:HGNC Symbol;Acc:HGNC:28356]	
MYC	0,41	7,87	3,153E-14	4 x 5	8 q24	v-myc avian myelocytomatosis viral oncogene homolog [Source:HGNC Symbol;Acc:HGNC:7553]	
SOCS6	0,41	7,86	3,3085E-14	6 x 1	18 q22	suppressor of cytokine signaling 6 [Source:HGNC Symbol;Acc:HGNC:16833]	
COPS3	0,41	7,86	3,3529E-14	2 x 5	17 p11	COP9 signalosome subunit 3 [Source:HGNC Symbol;Acc:HGNC:2239]	
SLC6A8	0,41	7,85	3,5083E-14	4 x 6	X q28	solute carrier family 6 member 8 [Source:HGNC Symbol;Acc:HGNC:11055]	
CCDC137	0,41	7,85	3,6415E-14	7 x 2	17 q25	coiled-coil domain containing 137 [Source:HGNC Symbol;Acc:HGNC:33451]	
NRSN2	0,41	7,84	3,8192E-14	1 x 5	20 p13	neurensin 2 [Source:HGNC Symbol;Acc:HGNC:16229]	
LAMA1	0,41	7,84	3,9191E-14	7 x 3	18 p11	laminin subunit alpha 1 [Source:HGNC Symbol;Acc:HGNC:6481]	
SUOX	0,41	7,80	4,8406E-14	6 x 1	12 q13	sulfite oxidase [Source:HGNC Symbol;Acc:HGNC:11460]	
	0,41	7,80	5,0182E-14	5 x 1			
MCF2L	0,41	7,79	5,1292E-14	5 x 4	13 q34	MCF.2 cell line derived transforming sequence like [Source:HGNC Symbol;Acc:HGNC:14576]	
CACYBP	0,41	7,78	5,6288E-14	1 x 8	1 q25	calcyclin binding protein [Source:HGNC Symbol;Acc:HGNC:30423]	
PAAF1	0,41	7,76	6,4171E-14	3 x 2	11 q13	proteasomal ATPase associated factor 1 [Source:HGNC Symbol;Acc:HGNC:25687]	
PDHX	0,41	7,75	6,839E-14	4 x 4	11 p13	pyruvate dehydrogenase complex component X [Source:HGNC Symbol;Acc:HGNC:21350]	
SFXN4	0,40	7,74	7,5939E-14	4 x 1	10 q26	sideroflexin 4 [Source:HGNC Symbol;Acc:HGNC:16088]	
FAM69B	0,40	7,73	7,7272E-14	5 x 8	9 q34	family with sequence similarity 69 member B [Source:HGNC Symbol;Acc:HGNC:28290]	
TRIO	0,40	7,72	8,5265E-14	4 x 4	5 p15	trio Rho guanine nucleotide exchange factor [Source:HGNC Symbol;Acc:HGNC:12303]	
MTFP1	0,40	7,72	8,5709E-14	2 x 4	22 q12	mitochondrial fission process 1 [Source:HGNC Symbol;Acc:HGNC:26945]	
INSIG2	0,40	7,71	9,015E-14	1 x 7	2 q14	insulin induced gene 2 [Source:HGNC Symbol;Acc:HGNC:20452]	
FAIM	0,40	7,70	9,4813E-14	3 x 5	3 q22	Fas apoptotic inhibitory molecule [Source:HGNC Symbol;Acc:HGNC:18703]	
FAM69A	0,40	7,69	1,017E-13	6 x 1	1 p22	family with sequence similarity 69 member A [Source:HGNC Symbol;Acc:HGNC:32213]	
WDR45B	0,40	7,67	1,1413E-13	1 x 6	17 q25	WD repeat domain 45B [Source:HGNC Symbol;Acc:HGNC:25072]	
FOS	0,40	7,64	1,3878E-13	4 x 3	14 q24	Fos proto-oncogene, AP-1 transcription factor subunit [Source:HGNC Symbol;Acc:HGNC:3796]	
PNPLA4	0,40	7,61	1,7086E-13	6 x 6	X p22	patatin like phospholipase domain containing 4 [Source:HGNC Symbol;Acc:HGNC:24887]	
LLPH	0,40	7,61	1,7641E-13	6 x 1	12 q14	LLP homolog, long-term synaptic facilitation [Source:HGNC Symbol;Acc:HGNC:28229]	
MGAT4A	0,40	7,60	1,8585E-13	5 x 4	2 q11	mannosyl (alpha-1,3-)-glycoprotein beta-1,4-N-acetylglucosaminyltransferase, isozyme A [Source:HGNC Symbol;Acc:HGNC:7047]	
ADI1	0,40	7,58	2,1005E-13	1 x 7	2 p25	acireductone dioxygenase 1 [Source:HGNC Symbol;Acc:HGNC:30576]	
FAM21A	0,40	7,58	2,1261E-13	3 x 6	10 q11	family with sequence similarity 21 member A [Source:HGNC Symbol;Acc:HGNC:23416]	
APOL2	0,40	7,53	2,8355E-13	6 x 1	22 q12	apolipoprotein L2 [Source:HGNC Symbol;Acc:HGNC:619]	
MRPS30	0,40	7,52	3,0731E-13	3 x 4	5 p12	mitochondrial ribosomal protein S30 [Source:HGNC Symbol;Acc:HGNC:8769]	
CEP95	0,40	7,52	3,1575E-13	4 x 5	17 q23	centrosomal protein 95 [Source:HGNC Symbol;Acc:HGNC:25141]	
BCKDK	0,40	7,51	3,213E-13	1 x 6	16 p11	branched chain ketoacid dehydrogenase kinase [Source:HGNC Symbol;Acc:HGNC:16902]	


DPP7	0,40	7,51	3,2463E-13	1 x 7	9 q34	dipeptidyl peptidase 7 [Source:HGNC Symbol;Acc:HGNC:14892]	
SNRPF	0,39	7,50	3,4772E-13	9 x 1	12 q23	small nuclear ribonucleoprotein polypeptide F [Source:HGNC Symbol;Acc:HGNC:11162]	
PRPSAP2	0,39	7,50	3,5061E-13	1 x 6	17 p11	phosphoribosyl pyrophosphate synthetase associated protein 2 [Source:HGNC Symbol;Acc:HGNC:9467]	
TVP23C	0,39	7,49	3,6393E-13	2 x 6	17 p12	trans-golgi network vesicle protein 23 homolog C (S. cerevisiae) [Source:HGNC Symbol;Acc:HGNC:30453]	
ECHDC2	0,39	7,49	3,7192E-13	5 x 6	1 p32	enoyl-CoA hydratase domain containing 2 [Source:HGNC Symbol;Acc:HGNC:23408]	
MLH3	0,39	7,48	3,9269E-13	4 x 4	14 q24	mutL homolog 3 [Source:HGNC Symbol;Acc:HGNC:7128]	
CHCHD4	0,39	7,48	3,9901E-13	5 x 1	3 p25	coiled-coil-helix-coiled-coil-helix domain containing 4 [Source:HGNC Symbol;Acc:HGNC:26467]	
ESRP1	0,39	7,47	4,2721E-13	7 x 1	8 q22	epithelial splicing regulatory protein 1 [Source:HGNC Symbol;Acc:HGNC:25966]	
RGS12	0,39	7,46	4,4098E-13	1 x 6	4 p16	regulator of G-protein signaling 12 [Source:HGNC Symbol;Acc:HGNC:9994]	
ZC3H13	0,39	7,46	4,4142E-13	4 x 6	13 q14	zinc finger CCCH-type containing 13 [Source:HGNC Symbol;Acc:HGNC:20368]	
GPRIN3	0,39	7,46	4,563E-13	8 x 2	4 q22	GPRIN family member 3 [Source:HGNC Symbol;Acc:HGNC:27733]	
HMGN1	0,39	7,45	4,7717E-13	5 x 9	21 q22	high mobility group nucleosome binding domain 1 [Source:HGNC Symbol;Acc:HGNC:4984]	
MCRS1	0,39	7,45	4,8495E-13	5 x 1	12 q13	microspherule protein 1 [Source:HGNC Symbol;Acc:HGNC:6960]	
TRPC6	0,39	7,45	4,8606E-13	10 x 6	11 q22	transient receptor potential cation channel subfamily C member 6 [Source:HGNC Symbol;Acc:HGNC:12338]	
SUCLG1	0,39	7,44	5,0915E-13	6 x 1	2 p11	succinate-CoA ligase alpha subunit [Source:HGNC Symbol;Acc:HGNC:11449]	
CINP	0,39	7,43	5,3613E-13	5 x 4	14 q32	cyclin dependent kinase 2 interacting protein [Source:HGNC Symbol;Acc:HGNC:23789]	
SYNPR	0,39	7,43	5,6011E-13	5 x 5	3 p14	synaptoporin [Source:HGNC Symbol;Acc:HGNC:16507]	
C11orf24	0,39	7,42	5,9985E-13	1 x 7	11 q13	chromosome 11 open reading frame 24 [Source:HGNC Symbol;Acc:HGNC:1174]	
ZNF131	0,39	7,41	6,3127E-13	3 x 5	5 p12	zinc finger protein 131 [Source:HGNC Symbol;Acc:HGNC:12915]	
CDIP1	0,39	7,40	6,809E-13	8 x 4	CHR_HSCHR16	cell death-inducing p53 target 1 [Source:HGNC Symbol;Acc:HGNC:13234]	
KIF21A	0,39	7,39	6,9256E-13	6 x 3	12 q12	kinesin family member 21A [Source:HGNC Symbol;Acc:HGNC:19349]	
ATP6V1D	0,39	7,38	7,7816E-13	1 x 8	14 q23	ATPase H+ transporting V1 subunit D [Source:HGNC Symbol;Acc:HGNC:13527]	
EVL	0,39	7,37	7,998E-13	6 x 6	14 q32	Enah/Vasp-like [Source:HGNC Symbol;Acc:HGNC:20234]	
CAPRIN1	0,39	7,37	8,1368E-13	4 x 6	11 p13	cell cycle associated protein 1 [Source:HGNC Symbol;Acc:HGNC:6743]	
CCDC57	0,39	7,36	8,4621E-13	3 x 5	17 q25	coiled-coil domain containing 57 [Source:HGNC Symbol;Acc:HGNC:27564]	
PPT1	0,39	7,36	8,4766E-13	1 x 6	1 p34	palmitoyl-protein thioesterase 1 [Source:HGNC Symbol;Acc:HGNC:9325]	
TBK1	0,39	7,36	8,6409E-13	4 x 4	12 q14	TANK binding kinase 1 [Source:HGNC Symbol;Acc:HGNC:11584]	
UAP1L1	0,39	7,33	1,0469E-12	9 x 2	9 q34	UDP-N-acetylglucosamine pyrophosphorylase 1 like 1 [Source:HGNC Symbol;Acc:HGNC:28082]	
OSBPL9	0,39	7,31	1,1884E-12	1 x 5	1 p32	oxysterol binding protein like 9 [Source:HGNC Symbol;Acc:HGNC:16386]	
CCDC43	0,39	7,30	1,2315E-12	6 x 1	17 q21	coiled-coil domain containing 43 [Source:HGNC Symbol;Acc:HGNC:26472]	
TIMM50	0,39	7,30	1,257E-12	1 x 7	19 q13	translocase of inner mitochondrial membrane 50 [Source:HGNC Symbol;Acc:HGNC:23656]	
CFAP61	0,39	7,30	1,2648E-12	6 x 4	20 p11	cilia and flagella associated protein 61 [Source:HGNC Symbol;Acc:HGNC:15872]	
BAIAP2	0,38	7,28	1,4437E-12	4 x 5	17 q25	BAI1 associated protein 2 [Source:HGNC Symbol;Acc:HGNC:947]	


C19orf12	0,38	7,27	1,5222E-12	6 x 1	19 q12	chromosome 19 open reading frame 12 [Source:HGNC Symbol;Acc:HGNC:25443]	
MPC1	0,38	7,26	1,6149E-12	4 x 1	6 q27	mitochondrial pyruvate carrier 1 [Source:HGNC Symbol;Acc:HGNC:21606]	
DSCR3	0,38	7,24	1,7981E-12	2 x 5	21 q22	DSCR3 arrestin fold containing [Source:HGNC Symbol;Acc:HGNC:3044]	
NUP210	0,38	7,22	2,1225E-12	9 x 5	3 p25	nucleoporin 210 [Source:HGNC Symbol;Acc:HGNC:30052]	
GPM6B	0,38	7,21	2,1669E-12	4 x 2	X p22	glycoprotein M6B [Source:HGNC Symbol;Acc:HGNC:4461]	
SLC25A16	0,38	7,19	2,4611E-12	6 x 1	10 q21	solute carrier family 25 member 16 [Source:HGNC Symbol;Acc:HGNC:10986]	
HDHD2	0,38	7,19	2,538E-12	3 x 3	18 q21	haloacid dehalogenase like hydrolase domain containing 2 [Source:HGNC Symbol;Acc:HGNC:25364]	
EPB41L3	0,38	7,17	2,9214E-12	4 x 6	18 p11	erythrocyte membrane protein band 4.1 like 3 [Source:HGNC Symbol;Acc:HGNC:3380]	
FBXO9	0,38	7,15	3,1887E-12	3 x 6	6 p12	F-box protein 9 [Source:HGNC Symbol;Acc:HGNC:13588]	
CKB	0,38	7,15	3,2602E-12	1 x 4	14 q32	creatine kinase B [Source:HGNC Symbol;Acc:HGNC:1991]	
ATXN10	0,38	7,14	3,3652E-12	1 x 6	22 q13	ataxin 10 [Source:HGNC Symbol;Acc:HGNC:10549]	
NLK	0,38	7,14	3,4891E-12	6 x 5	17 q11	nemo like kinase [Source:HGNC Symbol;Acc:HGNC:29858]	
SIAH1	0,38	7,13	3,5847E-12	6 x 1	16 q12	siah E3 ubiquitin protein ligase 1 [Source:HGNC Symbol;Acc:HGNC:10857]	
FARP1	0,38	7,13	3,6959E-12	5 x 1	13 q32	FERM, ARH/RhoGEF and pleckstrin domain protein 1 [Source:HGNC Symbol;Acc:HGNC:3591]	
HMGCR	0,38	7,12	3,8888E-12	1 x 6	5 q13	3-hydroxy-3-methylglutaryl-CoA reductase [Source:HGNC Symbol;Acc:HGNC:5006]	
SDHB	0,38	7,12	3,9488E-12	1 x 8	1 p36	succinate dehydrogenase complex iron sulfur subunit B [Source:HGNC Symbol;Acc:HGNC:10681]	
LPIN1	0,38	7,10	4,4822E-12	7 x 3	2 p25	lipin 1 [Source:HGNC Symbol;Acc:HGNC:13345]	
DHRS7B	0,38	7,09	4,5686E-12	1 x 5	17 p11	dehydrogenase/reductase 7B [Source:HGNC Symbol;Acc:HGNC:24547]	
CISD2	0,38	7,09	4,8137E-12	4 x 8	4 q24	CDGSH iron sulfur domain 2 [Source:HGNC Symbol;Acc:HGNC:24212]	
CYB5R4	0,38	7,07	5,425E-12	2 x 5	6 q14	cytochrome b5 reductase 4 [Source:HGNC Symbol;Acc:HGNC:20147]	
MAD2L1BP	0,37	7,06	5,5815E-12	4 x 4	6 p21	MAD2L1 binding protein [Source:HGNC Symbol;Acc:HGNC:21059]	
METTL23	0,37	7,05	6,1018E-12	1 x 6	17 q25	methyltransferase like 23 [Source:HGNC Symbol;Acc:HGNC:26988]	
GNG10	0,37	7,04	6,3323E-12	5 x 8	9 q31	G protein subunit gamma 10 [Source:HGNC Symbol;Acc:HGNC:4402]	
CHST11	0,37	7,04	6,5634E-12	8 x 4	12 q23	carbohydrate sulfotransferase 11 [Source:HGNC Symbol;Acc:HGNC:17422]	
PRMT3	0,37	7,03	6,7688E-12	1 x 7	11 p15	protein arginine methyltransferase 3 [Source:HGNC Symbol;Acc:HGNC:30163]	
DHCR7	0,37	7,03	6,8713E-12	1 x 8	11 q13	7-dehydrocholesterol reductase [Source:HGNC Symbol;Acc:HGNC:2860]	
IFI6	0,37	7,02	7,0448E-12	7 x 3	1 p35	interferon alpha inducible protein 6 [Source:HGNC Symbol;Acc:HGNC:4054]	
VEPH1	0,37	7,02	7,0453E-12	4 x 1	3 q25	ventricular zone expressed PH domain containing 1 [Source:HGNC Symbol;Acc:HGNC:25735]	
FRMD3	0,37	7,02	7,0806E-12	6 x 1	9 q21	FERM domain containing 3 [Source:HGNC Symbol;Acc:HGNC:24125]	
TUFM	0,37	7,02	7,3355E-12	1 x 7	16 p11	Tu translation elongation factor, mitochondrial [Source:HGNC Symbol;Acc:HGNC:12420]	
PAG1	0,37	7,01	7,532E-12	7 x 4	8 q21	phosphoprotein membrane anchor with glycosphingolipid microdomains 1 [Source:HGNC Symbol;Acc:HGNC:30043]	
FUOM	0,37	7,01	7,5475E-12	5 x 6	10 q26	fucose mutarotase [Source:HGNC Symbol;Acc:HGNC:24733]	
PPIF	0,37	7,00	8,1484E-12	8 x 1	10 q22	peptidylprolyl isomerase F [Source:HGNC Symbol;Acc:HGNC:9259]	


FOXK2	0,37	6,99	8,6127E-12	7 x 1	17 q25	forkhead box K2 [Source:HGNC Symbol;Acc:HGNC:6036]	
GUCD1	0,37	6,99	8,7939E-12	4 x 2	22 q11	guanylyl cyclase domain containing 1 [Source:HGNC Symbol;Acc:HGNC:14237]	
SRSF5	0,37	6,98	9,1664E-12	3 x 5	14 q24	serine and arginine rich splicing factor 5 [Source:HGNC Symbol;Acc:HGNC:10787]	
SPESP1	0,37	6,98	9,2086E-12	7 x 1	15 q23	sperm equatorial segment protein 1 [Source:HGNC Symbol;Acc:HGNC:15570]	
DUSP3	0,37	6,95	1,1269E-11	5 x 4	17 q21	dual specificity phosphatase 3 [Source:HGNC Symbol;Acc:HGNC:3069]	
BLVRB	0,37	6,94	1,1731E-11	4 x 4	19 q13	biliverdin reductase B [Source:HGNC Symbol;Acc:HGNC:1063]	
CARD14	0,37	6,92	1,3273E-11	8 x 4	17 q25	caspase recruitment domain family member 14 [Source:HGNC Symbol;Acc:HGNC:16446]	
TFAM	0,37	6,90	1,4796E-11	6 x 1	10 q21	transcription factor A, mitochondrial [Source:HGNC Symbol;Acc:HGNC:11741]	
G6PC3	0,37	6,90	1,5457E-11	1 x 8	17 q21	glucose-6-phosphatase catalytic subunit 3 [Source:HGNC Symbol;Acc:HGNC:24861]	
RWDD2B	0,36	6,84	2,1253E-11	4 x 4	21 q21	RWD domain containing 2B [Source:HGNC Symbol;Acc:HGNC:1302]	
LRRC8D	0,36	6,84	2,1708E-11	8 x 1	1 p22	leucine rich repeat containing 8 family member D [Source:HGNC Symbol;Acc:HGNC:16992]	
PLEKHH1	0,36	6,80	2,7552E-11	8 x 1	14 q24	pleckstrin homology, MyTH4 and FERM domain containing H1 [Source:HGNC Symbol;Acc:HGNC:17733]	
COQ4	0,36	6,80	2,7557E-11	3 x 9	9 q34	coenzyme Q4 [Source:HGNC Symbol;Acc:HGNC:19693]	
CCND3	0,36	6,80	2,782E-11	1 x 7	6 p21	cyclin D3 [Source:HGNC Symbol;Acc:HGNC:1585]	
HPS1	0,36	6,79	2,9439E-11	4 x 4	10 q24	HPS1, biogenesis of lysosomal organelles complex 3 subunit 1 [Source:HGNC Symbol;Acc:HGNC:5163]	
TARS	0,36	6,77	3,2318E-11	4 x 9	5 p13	threonyl-tRNA synthetase [Source:HGNC Symbol;Acc:HGNC:11572]	
PDIA3	0,36	6,76	3,5395E-11	1 x 8	15 q15	protein disulfide isomerase family A member 3 [Source:HGNC Symbol;Acc:HGNC:4606]	
CAB39L	0,36	6,76	3,5453E-11	5 x 6	13 q14	calcium binding protein 39 like [Source:HGNC Symbol;Acc:HGNC:20290]	
OXLD1	0,36	6,75	3,6951E-11	2 x 6	17 q25	oxidoreductase like domain containing 1 [Source:HGNC Symbol;Acc:HGNC:27901]	
KCTD21	0,36	6,75	3,8339E-11	8 x 1	11 q14	potassium channel tetramerization domain containing 21 [Source:HGNC Symbol;Acc:HGNC:27452]	
WRB	0,36	6,74	3,9698E-11	1 x 6	21 q22	tryptophan rich basic protein [Source:HGNC Symbol;Acc:HGNC:12790]	
ULK4	0,36	6,73	4,1444E-11	7 x 1	3 p22	unc-51 like kinase 4 [Source:HGNC Symbol;Acc:HGNC:15784]	
APEH	0,36	6,72	4,5581E-11	1 x 6	3 p21	acylaminoacyl-peptide hydrolase [Source:HGNC Symbol;Acc:HGNC:586]	
FN3KRP	0,36	6,71	4,647E-11	3 x 5	17 q25	fructosamine 3 kinase related protein [Source:HGNC Symbol;Acc:HGNC:25700]	
KANK2	0,36	6,70	4,9147E-11	6 x 1	19 p13	KN motif and ankyrin repeat domains 2 [Source:HGNC Symbol;Acc:HGNC:29300]	
AP5M1	0,36	6,70	4,9969E-11	5 x 3	14 q22	adaptor related protein complex 5 mu 1 subunit [Source:HGNC Symbol;Acc:HGNC:20192]	
EIF4A1	0,36	6,70	5,0699E-11	1 x 8	17 p13	eukaryotic translation initiation factor 4A1 [Source:HGNC Symbol;Acc:HGNC:3282]	
CCNB1IP1	0,36	6,69	5,2962E-11	5 x 1	14 q11	cyclin B1 interacting protein 1 [Source:HGNC Symbol;Acc:HGNC:19437]	
ARHGAP42	0,36	6,68	5,5016E-11	7 x 4	11 q22	Rho GTPase activating protein 42 [Source:HGNC Symbol;Acc:HGNC:26545]	
USP22	0,36	6,66	6,376E-11	9 x 3	17 p11	ubiquitin specific peptidase 22 [Source:HGNC Symbol;Acc:HGNC:12621]	
HIP1R	0,36	6,66	6,3947E-11	6 x 8	12 q24	huntingtin interacting protein 1 related [Source:HGNC Symbol;Acc:HGNC:18415]	
ATP6V1H	0,36	6,65	6,7087E-11	1 x 6	8 q11	ATPase H+ transporting V1 subunit H [Source:HGNC Symbol;Acc:HGNC:18303]	
MSI2	0,36	6,65	6,9416E-11	7 x 2	17 q22	musashi RNA binding protein 2 [Source:HGNC Symbol;Acc:HGNC:18585]	


ECSIT	0,36	6,64	7,1318E-11	1 x 8	19 p13	ECSIT signalling integrator [Source:HGNC Symbol;Acc:HGNC:29548]	
	0,35	6,63	7,6586E-11	6 x 2			
KIAA1191	0,35	6,62	7,9935E-11	6 x 1	5 q35	KIAA1191 [Source:HGNC Symbol;Acc:HGNC:29209]	
DERA	0,35	6,61	8,4516E-11	5 x 1	12 p12	deoxyribose-phosphate aldolase [Source:HGNC Symbol;Acc:HGNC:24269]	
ELOVL5	0,35	6,61	8,556E-11	4 x 6	6 p12	ELOVL fatty acid elongase 5 [Source:HGNC Symbol;Acc:HGNC:21308]	
TMEM183A	0,35	6,61	8,7455E-11	6 x 8	1 q32	transmembrane protein 183A [Source:HGNC Symbol;Acc:HGNC:20173]	
POU3F1	0,35	6,60	8,9048E-11	9 x 4	1 p34	POU class 3 homeobox 1 [Source:HGNC Symbol;Acc:HGNC:9214]	
NXF1	0,35	6,60	9,1598E-11	1 x 6	11 q12	nuclear RNA export factor 1 [Source:HGNC Symbol;Acc:HGNC:8071]	
ATP6V1C1	0,35	6,60	9,3674E-11	5 x 4	8 q22	ATPase H+ transporting V1 subunit C1 [Source:HGNC Symbol;Acc:HGNC:856]	
MSMO1	0,35	6,59	9,4495E-11	4 x 4	4 q32	methylsterol monooxygenase 1 [Source:HGNC Symbol;Acc:HGNC:10545]	
WBP2	0,35	6,59	9,6788E-11	1 x 10	17 q25	WW domain binding protein 2 [Source:HGNC Symbol;Acc:HGNC:12738]	
UQCC1	0,35	6,59	9,7325E-11	1 x 6	20 q11	ubiquinol-cytochrome c reductase complex assembly factor 1 [Source:HGNC Symbol;Acc:HGNC:15891]	
LUM	0,35	6,58	9,9768E-11	6 x 4	12 q21	lumican [Source:HGNC Symbol;Acc:HGNC:6724]	
SQLE	0,35	6,58	1,0198E-10	1 x 8	8 q24	squalene epoxidase [Source:HGNC Symbol;Acc:HGNC:11279]	
PRKD3	0,35	6,58	1,0309E-10	6 x 4	2 p22	protein kinase D3 [Source:HGNC Symbol;Acc:HGNC:9408]	
CHURC1	0,35	6,57	1,0785E-10	5 x 5	14 q23	churchill domain containing 1 [Source:HGNC Symbol;Acc:HGNC:20099]	
RANBP17	0,35	6,56	1,1562E-10	5 x 3	5 q35	RAN binding protein 17 [Source:HGNC Symbol;Acc:HGNC:14428]	
PNRC2	0,35	6,54	1,2678E-10	4 x 5	1 p36	proline rich nuclear receptor coactivator 2 [Source:HGNC Symbol;Acc:HGNC:23158]	
ORAOV1	0,35	6,54	1,2992E-10	5 x 6	11 q13	oral cancer overexpressed 1 [Source:HGNC Symbol;Acc:HGNC:17589]	
ARMC6	0,35	6,53	1,3453E-10	2 x 8	19 p13	armadillo repeat containing 6 [Source:HGNC Symbol;Acc:HGNC:25049]	
	0,35	6,53	1,3766E-10	6 x 8			
SH3PXD2B	0,35	6,52	1,422E-10	4 x 8	5 q35	SH3 and PX domains 2B [Source:HGNC Symbol;Acc:HGNC:29242]	
TMEM117	0,35	6,51	1,5633E-10	7 x 4	12 q12	transmembrane protein 117 [Source:HGNC Symbol;Acc:HGNC:25308]	
C4orf3	0,35	6,50	1,6151E-10	1 x 7	4 q26	chromosome 4 open reading frame 3 [Source:HGNC Symbol;Acc:HGNC:19225]	
ADAM10	0,35	6,49	1,7736E-10	8 x 2	15 q21	ADAM metallopeptidase domain 10 [Source:HGNC Symbol;Acc:HGNC:188]	
FAM86C1	0,35	6,48	1,8444E-10	3 x 10	11 q13	family with sequence similarity 86 member C1 [Source:HGNC Symbol;Acc:HGNC:25561]	
NME2	0,35	6,47	1,9791E-10	1 x 7	17 q21	NME/NM23 nucleoside diphosphate kinase 2 [Source:HGNC Symbol;Acc:HGNC:7850]	
MCF2	0,35	6,47	1,9914E-10	8 x 4	X q27	MCF.2 cell line derived transforming sequence [Source:HGNC Symbol;Acc:HGNC:6940]	
TSEN54	0,35	6,47	1,9961E-10	2 x 7	17 q25	tRNA splicing endonuclease subunit 54 [Source:HGNC Symbol;Acc:HGNC:27561]	
THUMPD2	0,35	6,46	2,0637E-10	1 x 6	2 p22	THUMP domain containing 2 [Source:HGNC Symbol;Acc:HGNC:14890]	
GUF1	0,35	6,46	2,0721E-10	6 x 5	4 p12	GUF1 homolog, GTPase [Source:HGNC Symbol;Acc:HGNC:25799]	
CDH7	0,35	6,44	2,2811E-10	9 x 5	18 q22	cadherin 7 [Source:HGNC Symbol;Acc:HGNC:1766]	
TBCD	0,35	6,44	2,2815E-10	1 x 6	17 q25	tubulin folding cofactor D [Source:HGNC Symbol;Acc:HGNC:11581]	


PPP4C	0,35	6,43	2,4076E-10	1 x 7	16 p11	protein phosphatase 4 catalytic subunit [Source:HGNC Symbol;Acc:HGNC:9319]	
SPTBN1	0,35	6,43	2,5307E-10	9 x 6	2 p16	spectrin beta, non-erythrocytic 1 [Source:HGNC Symbol;Acc:HGNC:11275]	
DDB1	0,35	6,42	2,5884E-10	4 x 6	11 q12	damage specific DNA binding protein 1 [Source:HGNC Symbol;Acc:HGNC:2717]	
C14orf37	0,34	6,40	2,9926E-10	8 x 5	14 q23	chromosome 14 open reading frame 37 [Source:HGNC Symbol;Acc:HGNC:19846]	
ITSN1	0,34	6,39	3,1E-10	4 x 6	21 q22	intersectin 1 [Source:HGNC Symbol;Acc:HGNC:6183]	
NSUN2	0,34	6,38	3,1962E-10	4 x 6	5 p15	NOP2/Sun RNA methyltransferase family member 2 [Source:HGNC Symbol;Acc:HGNC:25994]	
SLC5A10	0,34	6,38	3,205E-10	9 x 4	17 p11	solute carrier family 5 member 10 [Source:HGNC Symbol;Acc:HGNC:23155]	
GBA	0,34	6,38	3,2077E-10	6 x 7	CHR_HSCHR1_	glucosylceramidase beta [Source:HGNC Symbol;Acc:HGNC:4177]	
YAF2	0,34	6,38	3,2419E-10	5 x 1	12 q12	YY1 associated factor 2 [Source:HGNC Symbol;Acc:HGNC:17363]	
ENOSF1	0,34	6,37	3,5621E-10	4 x 5	18 p11	enolase superfamily member 1 [Source:HGNC Symbol;Acc:HGNC:30365]	
UNK	0,34	6,36	3,6216E-10	1 x 7	17 q25	unkempt family zinc finger [Source:HGNC Symbol;Acc:HGNC:29369]	
ZADH2	0,34	6,34	4,0161E-10	6 x 2	18 q22	zinc binding alcohol dehydrogenase domain containing 2 [Source:HGNC Symbol;Acc:HGNC:28697]	
GNPDA1	0,34	6,33	4,4023E-10	1 x 10	5 q31	glucosamine-6-phosphate deaminase 1 [Source:HGNC Symbol;Acc:HGNC:4417]	
TMEM38B	0,34	6,33	4,4605E-10	1 x 6	9 q31	transmembrane protein 38B [Source:HGNC Symbol;Acc:HGNC:25535]	
GADL1	0,34	6,32	4,5947E-10	9 x 3	3 p23	glutamate decarboxylase like 1 [Source:HGNC Symbol;Acc:HGNC:27949]	
SNRNP25	0,34	6,31	4,818E-10	1 x 10	16 p13	small nuclear ribonucleoprotein U11/U12 subunit 25 [Source:HGNC Symbol;Acc:HGNC:14161]	
MRPL28	0,34	6,30	5,0712E-10	1 x 7	16 p13	mitochondrial ribosomal protein L28 [Source:HGNC Symbol;Acc:HGNC:14484]	
CDCA4	0,34	6,30	5,3096E-10	5 x 5	14 q32	cell division cycle associated 4 [Source:HGNC Symbol;Acc:HGNC:14625]	
CYB5A	0,34	6,29	5,457E-10	1 x 5	18 q22	cytochrome b5 type A [Source:HGNC Symbol;Acc:HGNC:2570]	
GSTA1	0,34	6,29	5,5385E-10	6 x 8	6 p12	glutathione S-transferase alpha 1 [Source:HGNC Symbol;Acc:HGNC:4626]	
STX17	0,34	6,26	6,5465E-10	6 x 4	9 q31	syntaxin 17 [Source:HGNC Symbol;Acc:HGNC:11432]	
PSMG1	0,34	6,26	6,6256E-10	1 x 10	21 q22	proteasome assembly chaperone 1 [Source:HGNC Symbol;Acc:HGNC:3043]	
KIAA0100	0,34	6,26	6,6999E-10	1 x 7	17 q11	KIAA0100 [Source:HGNC Symbol;Acc:HGNC:28960]	
RAB4A	0,34	6,25	6,9489E-10	1 x 8	1 q42	RAB4A, member RAS oncogene family [Source:HGNC Symbol;Acc:HGNC:9781]	
GART	0,34	6,24	7,1597E-10	9 x 1	21 q22	phosphoribosylglycinamide formyltransferase, phosphoribosylglycinamide synthetase, phosphoribosylaminoimidazole synthetase [Source:	
FAM129A	0,34	6,22	8,0307E-10	9 x 4	1 q25	family with sequence similarity 129 member A [Source:HGNC Symbol;Acc:HGNC:16784]	
EIF3F	0,34	6,22	8,2982E-10	1 x 9	CHR_HSCHR11	eukaryotic translation initiation factor 3 subunit F [Source:HGNC Symbol;Acc:HGNC:3275]	
MPZL1	0,34	6,22	8,3586E-10	1 x 10	1 q24	myelin protein zero like 1 [Source:HGNC Symbol;Acc:HGNC:7226]	
CAMTA1	0,34	6,21	8,5563E-10	1 x 8	1 p36	calmodulin binding transcription activator 1 [Source:HGNC Symbol;Acc:HGNC:18806]	
VTI1B	0,33	6,21	8,7941E-10	3 x 6	14 q24	vesicle transport through interaction with t-SNAREs 1B [Source:HGNC Symbol;Acc:HGNC:17793]	
PMPCA	0,33	6,20	9,2887E-10	5 x 5	9 q34	peptidase, mitochondrial processing alpha subunit [Source:HGNC Symbol;Acc:HGNC:18667]	
PLEKHJ1	0,33	6,18	1,0308E-09	1 x 9	19 p13	pleckstrin homology domain containing J1 [Source:HGNC Symbol;Acc:HGNC:18211]	
PPFIBP2	0,33	6,17	1,0824E-09	2 x 6	11 p15	PPFIA binding protein 2 [Source:HGNC Symbol;Acc:HGNC:9250]	


TIPRL	0,33	6,17	1,1077E-09	4 x 7	1 q24	TOR signaling pathway regulator [Source:HGNC Symbol;Acc:HGNC:30231]	
PAPOLA	0,33	6,16	1,1582E-09	4 x 6	14 q32	poly(A) polymerase alpha [Source:HGNC Symbol;Acc:HGNC:14981]	
RAB7A	0,33	6,14	1,318E-09	1 x 9	3 q21	RAB7A, member RAS oncogene family [Source:HGNC Symbol;Acc:HGNC:9788]	
TRIB1	0,33	6,12	1,4352E-09	7 x 4	8 q24	tribbles pseudokinase 1 [Source:HGNC Symbol;Acc:HGNC:16891]	
FXN	0,33	6,12	1,46E-09	1 x 8	9 q21	frataxin [Source:HGNC Symbol;Acc:HGNC:3951]	
STARD3	0,33	6,12	1,4606E-09	2 x 8	17 q12	StAR related lipid transfer domain containing 3 [Source:HGNC Symbol;Acc:HGNC:17579]	
SYK	0,33	6,11	1,4962E-09	8 x 6	9 q22	spleen tyrosine kinase [Source:HGNC Symbol;Acc:HGNC:11491]	
FAM21C	0,33	6,10	1,5856E-09	2 x 8	10 q11	family with sequence similarity 21 member C [Source:HGNC Symbol;Acc:HGNC:23414]	
KCTD15	0,33	6,08	1,7532E-09	6 x 6	19 q13	potassium channel tetramerization domain containing 15 [Source:HGNC Symbol;Acc:HGNC:23297]	
RALGAPA1	0,33	6,08	1,7658E-09	7 x 6	14 q13	Ral GTPase activating protein catalytic alpha subunit 1 [Source:HGNC Symbol;Acc:HGNC:17770]	
INPP4B	0,33	6,08	1,8288E-09	6 x 4	4 q31	inositol polyphosphate-4-phosphatase type II B [Source:HGNC Symbol;Acc:HGNC:6075]	
TOP2B	0,33	6,05	2,0796E-09	9 x 1	3 p24	topoisomerase (DNA) II beta [Source:HGNC Symbol;Acc:HGNC:11990]	
DDX10	0,33	6,05	2,0965E-09	6 x 2	11 q22	DEAD-box helicase 10 [Source:HGNC Symbol;Acc:HGNC:2735]	
FMN1	0,33	6,05	2,1414E-09	9 x 1	CHR_HSCHR15	formin 1 [Source:HGNC Symbol;Acc:HGNC:3768]	
FLRT2	0,33	6,04	2,209E-09	6 x 3	14 q31	fibronectin leucine rich transmembrane protein 2 [Source:HGNC Symbol;Acc:HGNC:3761]	
TREX1	0,33	6,04	2,2495E-09	4 x 5	3 p21	three prime repair exonuclease 1 [Source:HGNC Symbol;Acc:HGNC:12269]	
CLN3	0,33	6,03	2,3267E-09	4 x 8	16 p12	ceroid-lipofuscinosis, neuronal 3 [Source:HGNC Symbol;Acc:HGNC:2074]	
KLHDC3	0,33	6,03	2,4015E-09	1 x 8	6 p21	kelch domain containing 3 [Source:HGNC Symbol;Acc:HGNC:20704]	
PAXBP1	0,33	6,03	2,4118E-09	7 x 2	21 q22	PAX3 and PAX7 binding protein 1 [Source:HGNC Symbol;Acc:HGNC:13579]	
ZNF76	0,33	6,02	2,5113E-09	6 x 7	6 p21	zinc finger protein 76 [Source:HGNC Symbol;Acc:HGNC:13149]	
SH3BP4	0,33	6,02	2,5405E-09	7 x 1	2 q37	SH3 domain binding protein 4 [Source:HGNC Symbol;Acc:HGNC:10826]	
UBAC1	0,33	6,01	2,6263E-09	6 x 4	9 q34	UBA domain containing 1 [Source:HGNC Symbol;Acc:HGNC:30221]	
USP10	0,32	6,00	2,8458E-09	4 x 5	16 q24	ubiquitin specific peptidase 10 [Source:HGNC Symbol;Acc:HGNC:12608]	
NIPBL	0,32	5,99	2,9419E-09	5 x 5	5 p13	NIPBL, cohesin loading factor [Source:HGNC Symbol;Acc:HGNC:28862]	
NOL11	0,32	5,99	3,025E-09	1 x 7	17 q24	nucleolar protein 11 [Source:HGNC Symbol;Acc:HGNC:24557]	
KTN1	0,32	5,97	3,2967E-09	5 x 7	14 q22	kinectin 1 [Source:HGNC Symbol;Acc:HGNC:6467]	
SPECC1	0,32	5,97	3,2974E-09	9 x 1	17 p11	sperm antigen with calponin homology and coiled-coil domains 1 [Source:HGNC Symbol;Acc:HGNC:30615]	
SRSF1	0,32	5,97	3,3723E-09	5 x 6	17 q22	serine and arginine rich splicing factor 1 [Source:HGNC Symbol;Acc:HGNC:10780]	
MAFG	0,32	5,95	3,617E-09	8 x 4	17 q25	MAF bZIP transcription factor G [Source:HGNC Symbol;Acc:HGNC:6781]	
LRTOMT	0,32	5,95	3,7524E-09	7 x 3	11 q13	leucine rich transmembrane and O-methyltransferase domain containing [Source:HGNC Symbol;Acc:HGNC:25033]	
SYNE1	0,32	5,93	4,0977E-09	2 x 6	6 q25	spectrin repeat containing nuclear envelope protein 1 [Source:HGNC Symbol;Acc:HGNC:17089]	
RNH1	0,32	5,93	4,1447E-09	3 x 9	CHR_HSCHR11	ribonuclease/angiogenin inhibitor 1 [Source:HGNC Symbol;Acc:HGNC:10074]	
C12orf10	0,32	5,93	4,1508E-09	4 x 4	12 q13	chromosome 12 open reading frame 10 [Source:HGNC Symbol;Acc:HGNC:17590]	


MFSD5	0,32	5,92	4,3825E-09	1 x 7	12 q13	major facilitator superfamily domain containing 5 [Source:HGNC Symbol;Acc:HGNC:28156]	
PMF1	0,32	5,92	4,4174E-09	1 x 9	1 q22	polyamine-modulated factor 1 [Source:HGNC Symbol;Acc:HGNC:9112]	
C4orf45	0,32	5,92	4,4174E-09	8 x 5	4 q32	chromosome 4 open reading frame 45 [Source:HGNC Symbol;Acc:HGNC:26342]	
H1F0	0,32	5,92	4,4297E-09	9 x 5	22 q13	H1 histone family member 0 [Source:HGNC Symbol;Acc:HGNC:4714]	
ATXN7L3B	0,32	5,91	4,5992E-09	7 x 2	12 q21	ataxin 7 like 3B [Source:HGNC Symbol;Acc:HGNC:37931]	
RIMKLB	0,32	5,89	5,056E-09	6 x 1	12 p13	ribosomal modification protein rimK-like family member B [Source:HGNC Symbol;Acc:HGNC:29228]	
PDE4B	0,32	5,88	5,3478E-09	4 x 6	1 p31	phosphodiesterase 4B [Source:HGNC Symbol;Acc:HGNC:8781]	
EFTUD2	0,32	5,87	5,6904E-09	4 x 6	17 q21	elongation factor Tu GTP binding domain containing 2 [Source:HGNC Symbol;Acc:HGNC:30858]	
PSMF1	0,32	5,87	5,8117E-09	1 x 7	20 p13	proteasome inhibitor subunit 1 [Source:HGNC Symbol;Acc:HGNC:9571]	
MAPKAPK3	0,32	5,86	5,8417E-09	2 x 8	3 p21	mitogen-activated protein kinase-activated protein kinase 3 [Source:HGNC Symbol;Acc:HGNC:6888]	
HNRNPA1L2	0,32	5,86	5,8445E-09	9 x 6	13 q14	heterogeneous nuclear ribonucleoprotein A1-like 2 [Source:HGNC Symbol;Acc:HGNC:27067]	
RFNG	0,32	5,86	6,031E-09	4 x 9	17 q25	RFNG O-fucosylpeptide 3-beta-N-acetylglucosaminyltransferase [Source:HGNC Symbol;Acc:HGNC:9974]	
NUDT14	0,32	5,86	6,0351E-09	6 x 8	14 q32	nudix hydrolase 14 [Source:HGNC Symbol;Acc:HGNC:20141]	
TMED10	0,32	5,85	6,171E-09	3 x 9	14 q24	transmembrane p24 trafficking protein 10 [Source:HGNC Symbol;Acc:HGNC:16998]	
AFG3L2	0,32	5,85	6,188E-09	7 x 4	18 p11	AFG3 like matrix AAA peptidase subunit 2 [Source:HGNC Symbol;Acc:HGNC:315]	
NAPA	0,32	5,85	6,2418E-09	1 x 8	19 q13	NSF attachment protein alpha [Source:HGNC Symbol;Acc:HGNC:7641]	
MTX1	0,32	5,85	6,4326E-09	6 x 6	1 q22	metaxin 1 [Source:HGNC Symbol;Acc:HGNC:7504]	
AK1	0,32	5,84	6,5705E-09	1 x 10	9 q34	adenylate kinase 1 [Source:HGNC Symbol;Acc:HGNC:361]	
MZT2B	0,32	5,84	6,5888E-09	1 x 8	2 q21	mitotic spindle organizing protein 2B [Source:HGNC Symbol;Acc:HGNC:25886]	
FAR1	0,32	5,84	6,6159E-09	6 x 1	11 p15	fatty acyl-CoA reductase 1 [Source:HGNC Symbol;Acc:HGNC:26222]	
DUSP23	0,32	5,81	7,691E-09	1 x 8	1 q23	dual specificity phosphatase 23 [Source:HGNC Symbol;Acc:HGNC:21480]	
CCL28	0,32	5,80	8,1965E-09	6 x 3	5 p12	C-C motif chemokine ligand 28 [Source:HGNC Symbol;Acc:HGNC:17700]	
MTHFD1	0,31	5,79	8,5697E-09	6 x 1	14 q23	methylenetetrahydrofolate dehydrogenase, cyclohydrolase and formyltetrahydrofolate synthetase 1 [Source:HGNC Symbol;Acc:HGNC:743	
SETD5	0,31	5,77	9,6219E-09	1 x 6	3 p25	SET domain containing 5 [Source:HGNC Symbol;Acc:HGNC:25566]	
SIMC1	0,31	5,76	1,0142E-08	6 x 8	5 q35	SUMO interacting motifs containing 1 [Source:HGNC Symbol;Acc:HGNC:24779]	
C1orf43	0,31	5,76	1,0419E-08	1 x 9	1 q21	chromosome 1 open reading frame 43 [Source:HGNC Symbol;Acc:HGNC:29876]	
WIPI1	0,31	5,75	1,0632E-08	1 x 7	17 q24	WD repeat domain, phosphoinositide interacting 1 [Source:HGNC Symbol;Acc:HGNC:25471]	
JAGN1	0,31	5,75	1,0651E-08	1 x 6	3 p25	jagunal homolog 1 [Source:HGNC Symbol;Acc:HGNC:26926]	
PRR5-ARHGAP8	0,31	5,74	1,1132E-08	7 x 1	22 q13	PRR5-ARHGAP8 readthrough [Source:HGNC Symbol;Acc:HGNC:34512]	
SLC16A1	0,31	5,74	1,1352E-08	9 x 1	CHR_HG2104_	solute carrier family 16 member 1 [Source:HGNC Symbol;Acc:HGNC:10922]	
PSMB10	0,31	5,74	1,1539E-08	4 x 5	16 q22	proteasome subunit beta 10 [Source:HGNC Symbol;Acc:HGNC:9538]	
	0,31	5,73	1,2109E-08	7 x 1			
MZT2A	0,31	5,72	1,2529E-08	1 x 8	2 q21	mitotic spindle organizing protein 2A [Source:HGNC Symbol;Acc:HGNC:33187]	


ARL6IP4	0,31	5,72	1,26E-08	1 x 8	12 q24	ADP ribosylation factor like GTPase 6 interacting protein 4 [Source:HGNC Symbol;Acc:HGNC:18076]	
SERPINF1	0,31	5,72	1,2786E-08	4 x 9	CHR_HSCHR17	serpin family F member 1 [Source:HGNC Symbol;Acc:HGNC:8824]	
PITX2	0,31	5,71	1,3195E-08	6 x 6	4 q25	paired like homeodomain 2 [Source:HGNC Symbol;Acc:HGNC:9005]	
HELZ	0,31	5,68	1,5509E-08	7 x 2	17 q24	helicase with zinc finger [Source:HGNC Symbol;Acc:HGNC:16878]	
NEU3	0,31	5,68	1,576E-08	7 x 2	11 q13	neuraminidase 3 (membrane sialidase) [Source:HGNC Symbol;Acc:HGNC:7760]	
GTF2H2C	0,31	5,67	1,6454E-08	4 x 8	5 q13	GTF2H2 family member C [Source:HGNC Symbol;Acc:HGNC:31394]	
RGS20	0,31	5,67	1,6739E-08	7 x 1	8 q11	regulator of G-protein signaling 20 [Source:HGNC Symbol;Acc:HGNC:14600]	
NUP98	0,31	5,67	1,678E-08	3 x 6	11 p15	nucleoporin 98 [Source:HGNC Symbol;Acc:HGNC:8068]	
MIF	0,31	5,66	1,7467E-08	1 x 10	CHR_HSCHR22	macrophage migration inhibitory factor (glycosylation-inhibiting factor) [Source:HGNC Symbol;Acc:HGNC:7097]	
MLST8	0,31	5,66	1,7658E-08	1 x 10	16 p13	MTOR associated protein, LST8 homolog [Source:HGNC Symbol;Acc:HGNC:24825]	
GYS1	0,31	5,65	1,8377E-08	7 x 6	19 q13	glycogen synthase 1 [Source:HGNC Symbol;Acc:HGNC:4706]	
DOCK7	0,31	5,64	1,9092E-08	7 x 3	1 p31	dedicator of cytokinesis 7 [Source:HGNC Symbol;Acc:HGNC:19190]	
PDE3B	0,31	5,64	1,9164E-08	9 x 5	11 p15	phosphodiesterase 3B [Source:HGNC Symbol;Acc:HGNC:8779]	
HDAC4	0,31	5,64	1,9674E-08	4 x 7	2 q37	histone deacetylase 4 [Source:HGNC Symbol;Acc:HGNC:14063]	
SOS1	0,31	5,63	1,9938E-08	7 x 1	2 p22	SOS Ras/Rac guanine nucleotide exchange factor 1 [Source:HGNC Symbol;Acc:HGNC:11187]	
MTMR4	0,31	5,63	2,0043E-08	7 x 5	17 q22	myotubularin related protein 4 [Source:HGNC Symbol;Acc:HGNC:7452]	
SLC38A7	0,31	5,62	2,1145E-08	8 x 3	16 q21	solute carrier family 38 member 7 [Source:HGNC Symbol;Acc:HGNC:25582]	
USF2	0,31	5,62	2,1896E-08	1 x 8	19 q13	upstream transcription factor 2, c-fos interacting [Source:HGNC Symbol;Acc:HGNC:12594]	
TGFBR1	0,31	5,62	2,1998E-08	7 x 2	9 q22	transforming growth factor beta receptor 1 [Source:HGNC Symbol;Acc:HGNC:11772]	
NCKAP5	0,31	5,60	2,3486E-08	9 x 3	2 q21	NCK associated protein 5 [Source:HGNC Symbol;Acc:HGNC:29847]	
TDG	0,31	5,60	2,3823E-08	7 x 2	12 q23	thymine DNA glycosylase [Source:HGNC Symbol;Acc:HGNC:11700]	
KLHL24	0,31	5,60	2,4092E-08	7 x 5	3 q27	kelch like family member 24 [Source:HGNC Symbol;Acc:HGNC:25947]	
OSBPL1A	0,31	5,59	2,4718E-08	7 x 5	18 q11	oxysterol binding protein like 1A [Source:HGNC Symbol;Acc:HGNC:16398]	
FAM45A	0,30	5,58	2,5981E-08	3 x 9	10 q26	family with sequence similarity 45 member A [Source:HGNC Symbol;Acc:HGNC:31793]	
PTBP2	0,30	5,57	2,7443E-08	7 x 1	1 p21	polypyrimidine tract binding protein 2 [Source:HGNC Symbol;Acc:HGNC:17662]	
RGS3	0,30	5,57	2,8314E-08	5 x 6	9 q32	regulator of G-protein signaling 3 [Source:HGNC Symbol;Acc:HGNC:9999]	
EIF1AX	0,30	5,57	2,8351E-08	8 x 7	X p22	eukaryotic translation initiation factor 1A, X-linked [Source:HGNC Symbol;Acc:HGNC:3250]	
KAZN	0,30	5,56	2,8834E-08	8 x 7	1 p36	kazrin, periplakin interacting protein [Source:HGNC Symbol;Acc:HGNC:29173]	
MRPL11	0,30	5,56	2,8976E-08	1 x 10	11 q13	mitochondrial ribosomal protein L11 [Source:HGNC Symbol;Acc:HGNC:14042]	
SSH2	0,30	5,56	2,9436E-08	7 x 4	17 q11	slingshot protein phosphatase 2 [Source:HGNC Symbol;Acc:HGNC:30580]	
PPM1H	0,30	5,56	2,9583E-08	7 x 1	12 q14	protein phosphatase, Mg2+/Mn2+ dependent 1H [Source:HGNC Symbol;Acc:HGNC:18583]	
CRBN	0,30	5,56	2,9729E-08	3 x 6	3 p26	cereblon [Source:HGNC Symbol;Acc:HGNC:30185]	
FNTA	0,30	5,55	3,1267E-08	2 x 6	8 p11	farnesyltransferase, CAAX box, alpha [Source:HGNC Symbol;Acc:HGNC:3782]	


CDC25B	0,30	5,53	3,4542E-08	4 x 9	20 p13	cell division cycle 25B [Source:HGNC Symbol;Acc:HGNC:1726]	
ACADM	0,30	5,53	3,5052E-08	9 x 1	1 p31	acyl-CoA dehydrogenase, C-4 to C-12 straight chain [Source:HGNC Symbol;Acc:HGNC:89]	
HYPK	0,30	5,52	3,5941E-08	5 x 6	15 q15	huntingtin interacting protein K [Source:HGNC Symbol;Acc:HGNC:18418]	
GYG2	0,30	5,52	3,6959E-08	8 x 7	X p22	glycogenin 2 [Source:HGNC Symbol;Acc:HGNC:4700]	
RHOT2	0,30	5,51	3,8597E-08	2 x 8	16 p13	ras homolog family member T2 [Source:HGNC Symbol;Acc:HGNC:21169]	
NDUFS2	0,30	5,49	4,1781E-08	1 x 10	1 q23	NADH:ubiquinone oxidoreductase core subunit S2 [Source:HGNC Symbol;Acc:HGNC:7708]	
MRPL37	0,30	5,48	4,4871E-08	1 x 10	1 p32	mitochondrial ribosomal protein L37 [Source:HGNC Symbol;Acc:HGNC:14034]	
CSNK1D	0,30	5,47	4,6302E-08	5 x 7	17 q25	casein kinase 1 delta [Source:HGNC Symbol;Acc:HGNC:2452]	
C12orf73	0,30	5,47	4,735E-08	7 x 3	12 q23	chromosome 12 open reading frame 73 [Source:HGNC Symbol;Acc:HGNC:34450]	
KAT7	0,30	5,47	4,7531E-08	4 x 7	17 q21	lysine acetyltransferase 7 [Source:HGNC Symbol;Acc:HGNC:17016]	
BRD9	0,30	5,47	4,7594E-08	8 x 6	5 p15	bromodomain containing 9 [Source:HGNC Symbol;Acc:HGNC:25818]	
SPNS1	0,30	5,47	4,7952E-08	1 x 9	16 p11	sphingolipid transporter 1 (putative) [Source:HGNC Symbol;Acc:HGNC:30621]	
TEX2	0,30	5,46	5,0511E-08	7 x 2	17 q23	testis expressed 2 [Source:HGNC Symbol;Acc:HGNC:30884]	
FAM133B	0,30	5,45	5,2814E-08	5 x 7	7 q21	family with sequence similarity 133 member B [Source:HGNC Symbol;Acc:HGNC:28629]	
TMEM41B	0,30	5,44	5,3486E-08	8 x 5	11 p15	transmembrane protein 41B [Source:HGNC Symbol;Acc:HGNC:28948]	
HNRNPM	0,30	5,44	5,3807E-08	1 x 9	19 p13	heterogeneous nuclear ribonucleoprotein M [Source:HGNC Symbol;Acc:HGNC:5046]	
GGA2	0,30	5,44	5,461E-08	9 x 1	16 p12	golgi associated, gamma adaptin ear containing, ARF binding protein 2 [Source:HGNC Symbol;Acc:HGNC:16064]	
MED9	0,30	5,44	5,6098E-08	10 x 5	17 p11	mediator complex subunit 9 [Source:HGNC Symbol;Acc:HGNC:25487]	
XXYLT1	0,30	5,42	5,9878E-08	8 x 6	3 q29	xyloside xylosyltransferase 1 [Source:HGNC Symbol;Acc:HGNC:26639]	
ZNF32	0,30	5,42	5,9962E-08	1 x 7	10 q11	zinc finger protein 32 [Source:HGNC Symbol;Acc:HGNC:13095]	
GFOD1	0,30	5,42	6,1235E-08	9 x 5	6 p23	glucose-fructose oxidoreductase domain containing 1 [Source:HGNC Symbol;Acc:HGNC:21096]	
MTG1	0,30	5,41	6,4083E-08	1 x 8	10 q26	mitochondrial ribosome associated GTPase 1 [Source:HGNC Symbol;Acc:HGNC:32159]	
PIGW	0,30	5,40	6,8461E-08	7 x 6	CHR_HSCHR17	phosphatidylinositol glycan anchor biosynthesis class W [Source:HGNC Symbol;Acc:HGNC:23213]	
CTNNA1	0,30	5,39	6,9824E-08	1 x 8	5 q31	catenin alpha 1 [Source:HGNC Symbol;Acc:HGNC:2509]	
DYRK1A	0,29	5,38	7,4023E-08	8 x 2	21 q22	dual specificity tyrosine phosphorylation regulated kinase 1A [Source:HGNC Symbol;Acc:HGNC:3091]	
CYB561A3	0,29	5,36	8,0424E-08	1 x 9	11 q12	cytochrome b561 family member A3 [Source:HGNC Symbol;Acc:HGNC:23014]	
RBBP7	0,29	5,36	8,0612E-08	1 x 9	X p22	RB binding protein 7, chromatin remodeling factor [Source:HGNC Symbol;Acc:HGNC:9890]	
MXRA7	0,29	5,36	8,0675E-08	7 x 6	17 q25	matrix remodeling associated 7 [Source:HGNC Symbol;Acc:HGNC:7541]	
TRAP1	0,29	5,36	8,1504E-08	4 x 8	16 p13	TNF receptor associated protein 1 [Source:HGNC Symbol;Acc:HGNC:16264]	
MRPS27	0,29	5,36	8,2153E-08	5 x 3	5 q13	mitochondrial ribosomal protein S27 [Source:HGNC Symbol;Acc:HGNC:14512]	
DONSON	0,29	5,36	8,2289E-08	7 x 1	21 q22	downstream neighbor of SON [Source:HGNC Symbol;Acc:HGNC:2993]	
WIPF3	0,29	5,36	8,2758E-08	9 x 3	7 p14	WAS/WASL interacting protein family member 3 [Source:HGNC Symbol;Acc:HGNC:22004]	
ARAP1	0,29	5,36	8,3423E-08	5 x 7	11 q13	ArfGAP with RhoGAP domain, ankyrin repeat and PH domain 1 [Source:HGNC Symbol;Acc:HGNC:16925]	


APEX1	0,29	5,35	8,4784E-08	1 x 7	14 q11	apurinic/apyrimidinic endodeoxyribonuclease 1 [Source:HGNC Symbol;Acc:HGNC:587]	
SLC11A2	0,29	5,35	8,5752E-08	5 x 6	12 q13	solute carrier family 11 member 2 [Source:HGNC Symbol;Acc:HGNC:10908]	
	0,29	5,34	8,9579E-08	5 x 1			
CDK5RAP3	0,29	5,34	9,0948E-08	4 x 6	17 q21	CDK5 regulatory subunit associated protein 3 [Source:HGNC Symbol;Acc:HGNC:18673]	
ABCC2	0,29	5,34	9,3249E-08	8 x 6	10 q24	ATP binding cassette subfamily C member 2 [Source:HGNC Symbol;Acc:HGNC:53]	
ZNF518B	0,29	5,32	9,9619E-08	8 x 1	4 p16	zinc finger protein 518B [Source:HGNC Symbol;Acc:HGNC:29365]	
SMARCC1	0,29	5,32	1,0029E-07	9 x 1	3 p21	SWI/SNF related, matrix associated, actin dependent regulator of chromatin subfamily c member 1 [Source:HGNC Symbol;Acc:HGNC:1110	
PLN	0,29	5,31	1,046E-07	9 x 3	6 q22	phospholamban [Source:HGNC Symbol;Acc:HGNC:9080]	
TMEM199	0,29	5,31	1,0631E-07	1 x 10	17 q11	transmembrane protein 199 [Source:HGNC Symbol;Acc:HGNC:18085]	
TMEM115	0,29	5,31	1,0742E-07	3 x 9	3 p21	transmembrane protein 115 [Source:HGNC Symbol;Acc:HGNC:30055]	
IL10RB	0,29	5,30	1,0915E-07	1 x 9	21 q22	interleukin 10 receptor subunit beta [Source:HGNC Symbol;Acc:HGNC:5965]	
PPP5C	0,29	5,28	1,2145E-07	1 x 8	19 q13	protein phosphatase 5 catalytic subunit [Source:HGNC Symbol;Acc:HGNC:9322]	
FAM193B	0,29	5,28	1,2205E-07	4 x 9	5 q35	family with sequence similarity 193 member B [Source:HGNC Symbol;Acc:HGNC:25524]	
LZTFL1	0,29	5,28	1,2236E-07	7 x 5	3 p21	leucine zipper transcription factor like 1 [Source:HGNC Symbol;Acc:HGNC:6741]	
SOD2	0,29	5,27	1,2722E-07	7 x 1	6 q25	superoxide dismutase 2, mitochondrial [Source:HGNC Symbol;Acc:HGNC:11180]	
KIAA1324L	0,29	5,27	1,3093E-07	9 x 5	7 q21	KIAA1324 like [Source:HGNC Symbol;Acc:HGNC:21945]	
MRPL17	0,29	5,26	1,3257E-07	1 x 8	11 p15	mitochondrial ribosomal protein L17 [Source:HGNC Symbol;Acc:HGNC:14053]	
HIST3H2A	0,29	5,25	1,4098E-07	4 x 9	1 q42	histone cluster 3, H2a [Source:HGNC Symbol;Acc:HGNC:20507]	
EXOSC3	0,29	5,25	1,4136E-07	1 x 7	9 p13	exosome component 3 [Source:HGNC Symbol;Acc:HGNC:17944]	
TMEM177	0,29	5,25	1,4585E-07	7 x 3	2 q14	transmembrane protein 177 [Source:HGNC Symbol;Acc:HGNC:28143]	
MRPL9	0,29	5,24	1,4814E-07	1 x 7	1 q21	mitochondrial ribosomal protein L9 [Source:HGNC Symbol;Acc:HGNC:14277]	
MRPS2	0,29	5,24	1,5099E-07	1 x 8	9 q34	mitochondrial ribosomal protein S2 [Source:HGNC Symbol;Acc:HGNC:14495]	
ANO2	0,29	5,24	1,5122E-07	8 x 3	12 p13	anoctamin 2 [Source:HGNC Symbol;Acc:HGNC:1183]	
MAEA	0,29	5,24	1,5251E-07	5 x 7	4 p16	macrophage erythroblast attacher [Source:HGNC Symbol;Acc:HGNC:13731]	
	0,29	5,23	1,5741E-07	7 x 5			
EXD2	0,29	5,22	1,6274E-07	8 x 6	14 q24	exonuclease 3'-5' domain containing 2 [Source:HGNC Symbol;Acc:HGNC:20217]	
OTULIN	0,29	5,22	1,6717E-07	8 x 7	5 p15	OTU deubiquitinase with linear linkage specificity [Source:HGNC Symbol;Acc:HGNC:25118]	
PTPN2	0,29	5,22	1,6781E-07	1 x 8	18 p11	protein tyrosine phosphatase, non-receptor type 2 [Source:HGNC Symbol;Acc:HGNC:9650]	
ZBTB43	0,29	5,20	1,8259E-07	7 x 5	9 q33	zinc finger and BTB domain containing 43 [Source:HGNC Symbol;Acc:HGNC:17908]	
NADSYN1	0,29	5,20	1,8327E-07	4 x 8	11 q13	NAD synthetase 1 [Source:HGNC Symbol;Acc:HGNC:29832]	
SYNJ2BP	0,28	5,19	1,9409E-07	8 x 4	14 q24	synaptojanin 2 binding protein [Source:HGNC Symbol;Acc:HGNC:18955]	
SHPRH	0,28	5,18	2,0406E-07	8 x 4	6 q24	SNF2 histone linker PHD RING helicase [Source:HGNC Symbol;Acc:HGNC:19336]	
LRRC39	0,28	5,18	2,0529E-07	9 x 6	1 p21	leucine rich repeat containing 39 [Source:HGNC Symbol;Acc:HGNC:28228]	


GAMT	0,28	5,17	2,0839E-07	2 x 9	19 p13	guanidinoacetate N-methyltransferase [Source:HGNC Symbol;Acc:HGNC:4136]	
GRHPR	0,28	5,16	2,1864E-07	1 x 10	9 p13	glyoxylate reductase/hydroxypyruvate reductase [Source:HGNC Symbol;Acc:HGNC:4570]	
SSPN	0,28	5,15	2,3393E-07	9 x 3	12 p12	sarcospan [Source:HGNC Symbol;Acc:HGNC:11322]	
TMEM134	0,28	5,14	2,4396E-07	1 x 8	11 q13	transmembrane protein 134 [Source:HGNC Symbol;Acc:HGNC:26142]	
PCCB	0,28	5,13	2,5689E-07	5 x 6	3 q22	propionyl-CoA carboxylase beta subunit [Source:HGNC Symbol;Acc:HGNC:8654]	
CFL2	0,28	5,12	2,7012E-07	6 x 1	14 q13	cofilin 2 [Source:HGNC Symbol;Acc:HGNC:1875]	
ZNF44	0,28	5,08	3,3544E-07	8 x 4	19 p13	zinc finger protein 44 [Source:HGNC Symbol;Acc:HGNC:13110]	
UBQLN1	0,28	5,06	3,6204E-07	2 x 10	9 q21	ubiquilin 1 [Source:HGNC Symbol;Acc:HGNC:12508]	
CISD1	0,28	5,06	3,6328E-07	8 x 1	10 q21	CDGSH iron sulfur domain 1 [Source:HGNC Symbol;Acc:HGNC:30880]	
CASP7	0,28	5,06	3,6939E-07	9 x 1	10 q25	caspase 7 [Source:HGNC Symbol;Acc:HGNC:1508]	
OXNAD1	0,28	5,05	3,7475E-07	7 x 6	3 p25	oxidoreductase NAD binding domain containing 1 [Source:HGNC Symbol;Acc:HGNC:25128]	
MTHFS	0,28	5,05	3,7528E-07	5 x 9	15 q25	5,10-methenyltetrahydrofolate synthetase (5-formyltetrahydrofolate cyclo-ligase) [Source:HGNC Symbol;Acc:HGNC:7437]	
IGHMBP2	0,28	5,05	3,7697E-07	5 x 9	11 q13	immunoglobulin mu binding protein 2 [Source:HGNC Symbol;Acc:HGNC:5542]	
TMEM138	0,28	5,05	3,7763E-07	1 x 6	11 q12	transmembrane protein 138 [Source:HGNC Symbol;Acc:HGNC:26944]	
PSAP	0,28	5,05	3,8856E-07	9 x 7	10 q22	prosaposin [Source:HGNC Symbol;Acc:HGNC:9498]	
TTC28	0,28	5,04	4,083E-07	9 x 5	22 q12	tetratricopeptide repeat domain 28 [Source:HGNC Symbol;Acc:HGNC:29179]	
TTC7B	0,28	5,03	4,2149E-07	5 x 9	14 q32	tetratricopeptide repeat domain 7B [Source:HGNC Symbol;Acc:HGNC:19858]	
LITAF	0,28	5,02	4,4765E-07	3 x 6	16 p13	lipopolysaccharide induced TNF factor [Source:HGNC Symbol;Acc:HGNC:16841]	
BFAR	0,28	5,01	4,6273E-07	8 x 1	CHR_HSCHR16	bifunctional apoptosis regulator [Source:HGNC Symbol;Acc:HGNC:17613]	
SLC25A19	0,28	5,00	4,8608E-07	5 x 6	17 q25	solute carrier family 25 member 19 [Source:HGNC Symbol;Acc:HGNC:14409]	
STT3B	0,27	4,99	4,9898E-07	9 x 1	3 p23	STT3B, catalytic subunit of the oligosaccharyltransferase complex [Source:HGNC Symbol;Acc:HGNC:30611]	
ZFAND2B	0,27	4,99	5,0284E-07	3 x 8	2 q35	zinc finger AN1-type containing 2B [Source:HGNC Symbol;Acc:HGNC:25206]	
RNF10	0,27	4,99	5,183E-07	1 x 7	12 q24	ring finger protein 10 [Source:HGNC Symbol;Acc:HGNC:10055]	
GFM1	0,27	4,98	5,2811E-07	5 x 7	3 q25	G elongation factor, mitochondrial 1 [Source:HGNC Symbol;Acc:HGNC:13780]	
DCTN5	0,27	4,97	5,4679E-07	8 x 1	16 p12	dynactin subunit 5 [Source:HGNC Symbol;Acc:HGNC:24594]	
BAAT	0,27	4,97	5,5097E-07	8 x 3	CHR_HSCHR9_	bile acid-CoA:amino acid N-acyltransferase [Source:HGNC Symbol;Acc:HGNC:932]	
GATB	0,27	4,97	5,5863E-07	9 x 2	4 q31	glutamyl-tRNA(Gln) amidotransferase, subunit B [Source:HGNC Symbol;Acc:HGNC:8849]	
NTAN1	0,27	4,97	5,6867E-07	3 x 10	CHR_HSCHR16	N-terminal asparagine amidase [Source:HGNC Symbol;Acc:HGNC:29909]	
TIMM9	0,27	4,96	5,8456E-07	1 x 8	14 q23	translocase of inner mitochondrial membrane 9 [Source:HGNC Symbol;Acc:HGNC:11819]	
C9orf91	0,27	4,96	5,8556E-07	8 x 5	9 q32	chromosome 9 open reading frame 91 [Source:HGNC Symbol;Acc:HGNC:24513]	
UNG	0,27	4,95	6,0261E-07	1 x 9	12 q24	uracil DNA glycosylase [Source:HGNC Symbol;Acc:HGNC:12572]	
C5orf42	0,27	4,95	6,0283E-07	7 x 3	5 p13	chromosome 5 open reading frame 42 [Source:HGNC Symbol;Acc:HGNC:25801]	
TMEM101	0,27	4,95	6,0773E-07	7 x 1	17 q21	transmembrane protein 101 [Source:HGNC Symbol;Acc:HGNC:28653]	


XPO5	0,27	4,95	6,0796E-07	4 x 7	6 p21	exportin 5 [Source:HGNC Symbol;Acc:HGNC:17675]	
SP110	0,27	4,95	6,1464E-07	9 x 4	2 q37	SP110 nuclear body protein [Source:HGNC Symbol;Acc:HGNC:5401]	
ARIH2	0,27	4,95	6,1504E-07	2 x 8	3 p21	ariadne RBR E3 ubiquitin protein ligase 2 [Source:HGNC Symbol;Acc:HGNC:690]	
MPDU1	0,27	4,95	6,2158E-07	1 x 10	17 p13	mannose-P-dolichol utilization defect 1 [Source:HGNC Symbol;Acc:HGNC:7207]	
DROSHA	0,27	4,94	6,356E-07	3 x 5	5 p13	drosha ribonuclease III [Source:HGNC Symbol;Acc:HGNC:17904]	
MECR	0,27	4,94	6,3576E-07	9 x 1	1 p35	mitochondrial trans-2-enoyl-CoA reductase [Source:HGNC Symbol;Acc:HGNC:19691]	
EGLN3	0,27	4,94	6,4671E-07	9 x 5	14 q13	egl-9 family hypoxia inducible factor 3 [Source:HGNC Symbol;Acc:HGNC:14661]	
RAD51B	0,27	4,94	6,5151E-07	3 x 7	14 q24	RAD51 paralog B [Source:HGNC Symbol;Acc:HGNC:9822]	
PIGP	0,27	4,93	6,6205E-07	3 x 10	21 q22	phosphatidylinositol glycan anchor biosynthesis class P [Source:HGNC Symbol;Acc:HGNC:3046]	
SPCS3	0,27	4,93	6,7686E-07	9 x 1	4 q34	signal peptidase complex subunit 3 [Source:HGNC Symbol;Acc:HGNC:26212]	
AP3D1	0,27	4,91	7,2658E-07	1 x 9	19 p13	adaptor related protein complex 3 delta 1 subunit [Source:HGNC Symbol;Acc:HGNC:568]	
USP25	0,27	4,91	7,2746E-07	7 x 6	21 q21	ubiquitin specific peptidase 25 [Source:HGNC Symbol;Acc:HGNC:12624]	
HNMT	0,27	4,91	7,4731E-07	6 x 7	2 q22	histamine N-methyltransferase [Source:HGNC Symbol;Acc:HGNC:5028]	
AHCY	0,27	4,91	7,4804E-07	4 x 9	20 q11	adenosylhomocysteinase [Source:HGNC Symbol;Acc:HGNC:343]	
ATXN2	0,27	4,91	7,5292E-07	3 x 7	12 q24	ataxin 2 [Source:HGNC Symbol;Acc:HGNC:10555]	
THOC7	0,27	4,88	8,4743E-07	1 x 10	3 p14	THO complex 7 [Source:HGNC Symbol;Acc:HGNC:29874]	
CCNDBP1	0,27	4,88	8,4998E-07	4 x 7	15 q15	cyclin D1 binding protein 1 [Source:HGNC Symbol;Acc:HGNC:1587]	
USP32	0,27	4,87	8,8039E-07	3 x 8	17 q23	ubiquitin specific peptidase 32 [Source:HGNC Symbol;Acc:HGNC:19143]	
MDH2	0,27	4,87	8,9049E-07	1 x 10	7 q11	malate dehydrogenase 2 [Source:HGNC Symbol;Acc:HGNC:6971]	
SOX6	0,27	4,86	9,1891E-07	8 x 4	11 p15	SRY-box 6 [Source:HGNC Symbol;Acc:HGNC:16421]	
OAS3	0,27	4,86	9,4683E-07	9 x 1	12 q24	2'-5'-oligoadenylate synthetase 3 [Source:HGNC Symbol;Acc:HGNC:8088]	
GNA13	0,27	4,86	9,5924E-07	7 x 1	17 q24	G protein subunit alpha 13 [Source:HGNC Symbol;Acc:HGNC:4381]	
DHCR24	0,27	4,86	9,6161E-07	5 x 9	1 p32	24-dehydrocholesterol reductase [Source:HGNC Symbol;Acc:HGNC:2859]	
CCSAP	0,27	4,85	9,6677E-07	9 x 1	1 q42	centriole, cilia and spindle associated protein [Source:HGNC Symbol;Acc:HGNC:29578]	
MAPK1IP1L	0,27	4,85	9,8161E-07	10 x 6	14 q22	mitogen-activated protein kinase 1 interacting protein 1-like [Source:HGNC Symbol;Acc:HGNC:19840]	
CDK5RAP2	0,27	4,84	1,0178E-06	7 x 4	9 q33	CDK5 regulatory subunit associated protein 2 [Source:HGNC Symbol;Acc:HGNC:18672]	
	0,27	4,84	1,0218E-06	4 x 9			
PIK3C2A	0,27	4,82	1,1283E-06	8 x 6	11 p15	phosphatidylinositol-4-phosphate 3-kinase catalytic subunit type 2 alpha [Source:HGNC Symbol;Acc:HGNC:8971]	
GLOD4	0,27	4,82	1,1575E-06	1 x 10	17 p13	glyoxalase domain containing 4 [Source:HGNC Symbol;Acc:HGNC:14111]	
GRSF1	0,27	4,81	1,1777E-06	8 x 6	4 q13	G-rich RNA sequence binding factor 1 [Source:HGNC Symbol;Acc:HGNC:4610]	
SOX10	0,27	4,81	1,1903E-06	8 x 6	22 q13	SRY-box 10 [Source:HGNC Symbol;Acc:HGNC:11190]	
CFAP97	0,27	4,80	1,2376E-06	5 x 8	4 q35	cilia and flagella associated protein 97 [Source:HGNC Symbol;Acc:HGNC:29276]	
UBE2I	0,27	4,80	1,2405E-06	1 x 7	16 p13	ubiquitin conjugating enzyme E2 I [Source:HGNC Symbol;Acc:HGNC:12485]	


ADARB1	0,26	4,80	1,2503E-06	8 x 5	21 q22	adenosine deaminase, RNA specific B1 [Source:HGNC Symbol;Acc:HGNC:226]	
SYNJ2BP-COX16	0,26	4,80	1,271E-06	5 x 8	14 q24	SYNJ2BP-COX16 readthrough [Source:HGNC Symbol;Acc:HGNC:48350]	
AKAP10	0,26	4,80	1,2717E-06	7 x 6	17 p11	A-kinase anchoring protein 10 [Source:HGNC Symbol;Acc:HGNC:368]	
LRRK2	0,26	4,79	1,2876E-06	8 x 6	12 q12	leucine rich repeat kinase 2 [Source:HGNC Symbol;Acc:HGNC:18618]	
PDGFD	0,26	4,78	1,3509E-06	9 x 7	11 q22	platelet derived growth factor D [Source:HGNC Symbol;Acc:HGNC:30620]	
SRPK2	0,26	4,78	1,3628E-06	8 x 3	7 q22	SRSF protein kinase 2 [Source:HGNC Symbol;Acc:HGNC:11306]	
NTHL1	0,26	4,78	1,3816E-06	1 x 8	16 p13	nth-like DNA glycosylase 1 [Source:HGNC Symbol;Acc:HGNC:8028]	
	0,26	4,78	1,3881E-06	9 x 1			
MRPL14	0,26	4,77	1,4185E-06	1 x 10	6 p21	mitochondrial ribosomal protein L14 [Source:HGNC Symbol;Acc:HGNC:14279]	
ESCO1	0,26	4,77	1,4562E-06	7 x 2	18 q11	establishment of sister chromatid cohesion N-acetyltransferase 1 [Source:HGNC Symbol;Acc:HGNC:24645]	
OSBPL8	0,26	4,76	1,4739E-06	8 x 6	12 q21	oxysterol binding protein like 8 [Source:HGNC Symbol;Acc:HGNC:16396]	
COX6A2	0,26	4,75	1,5654E-06	9 x 5	16 p11	cytochrome c oxidase subunit 6A2 [Source:HGNC Symbol;Acc:HGNC:2279]	
NDUFAF5	0,26	4,74	1,6234E-06	4 x 6	20 p12	NADH:ubiquinone oxidoreductase complex assembly factor 5 [Source:HGNC Symbol;Acc:HGNC:15899]	
38961	0,26	4,74	1,6263E-06	9 x 3	X q24	septin 6 [Source:HGNC Symbol;Acc:HGNC:15848]	
MRPL24	0,26	4,74	1,6265E-06	1 x 9	1 q23	mitochondrial ribosomal protein L24 [Source:HGNC Symbol;Acc:HGNC:14037]	
MIF4GD	0,26	4,74	1,6391E-06	1 x 8	17 q25	MIF4G domain containing [Source:HGNC Symbol;Acc:HGNC:24030]	
CHFR	0,26	4,74	1,6696E-06	8 x 7	12 q24	checkpoint with forkhead and ring finger domains, E3 ubiquitin protein ligase [Source:HGNC Symbol;Acc:HGNC:20455]	
IRAK4	0,26	4,73	1,6853E-06	5 x 8	12 q12	interleukin 1 receptor associated kinase 4 [Source:HGNC Symbol;Acc:HGNC:17967]	
MARK1	0,26	4,71	1,8569E-06	9 x 7	1 q41	microtubule affinity regulating kinase 1 [Source:HGNC Symbol;Acc:HGNC:6896]	
TPGS2	0,26	4,71	1,8619E-06	1 x 9	18 q12	tubulin polyglutamylase complex subunit 2 [Source:HGNC Symbol;Acc:HGNC:24561]	
LRCH1	0,26	4,71	1,9108E-06	9 x 6	13 q14	leucine rich repeats and calponin homology domain containing 1 [Source:HGNC Symbol;Acc:HGNC:20309]	
ITGAX	0,26	4,70	1,954E-06	9 x 5	16 p11	integrin subunit alpha X [Source:HGNC Symbol;Acc:HGNC:6152]	
DGKI	0,26	4,70	2,0004E-06	9 x 1	7 q33	diacylglycerol kinase iota [Source:HGNC Symbol;Acc:HGNC:2855]	
GXYLT1	0,26	4,69	2,034E-06	8 x 1	12 q12	glucoside xylosyltransferase 1 [Source:HGNC Symbol;Acc:HGNC:27482]	
EPG5	0,26	4,69	2,0535E-06	8 x 4	18 q21	ectopic P-granules autophagy protein 5 homolog (C. elegans) [Source:HGNC Symbol;Acc:HGNC:29331]	
EPB41	0,26	4,69	2,1026E-06	4 x 8	1 p35	erythrocyte membrane protein band 4.1 [Source:HGNC Symbol;Acc:HGNC:3377]	
STIM1	0,26	4,69	2,1043E-06	5 x 9	11 p15	stromal interaction molecule 1 [Source:HGNC Symbol;Acc:HGNC:11386]	
FAM161A	0,26	4,68	2,2084E-06	9 x 1	2 p15	family with sequence similarity 161 member A [Source:HGNC Symbol;Acc:HGNC:25808]	
RDH11	0,26	4,67	2,2755E-06	4 x 7	14 q24	retinol dehydrogenase 11 (all-trans/9-cis/11-cis) [Source:HGNC Symbol;Acc:HGNC:17964]	
GM2A	0,26	4,66	2,4063E-06	8 x 3	5 q33	GM2 ganglioside activator [Source:HGNC Symbol;Acc:HGNC:4367]	
LUZP6	0,26	4,65	2,4679E-06	1 x 7	7 q33	leucine zipper protein 6 [Source:HGNC Symbol;Acc:HGNC:33955]	
RNF138	0,26	4,64	2,5799E-06	3 x 7	18 q12	ring finger protein 138 [Source:HGNC Symbol;Acc:HGNC:17765]	
PSTK	0,26	4,64	2,6155E-06	1 x 8	10 q26	phosphoseryl-tRNA kinase [Source:HGNC Symbol;Acc:HGNC:28578]	


ATP6V1A	0,26	4,63	2,6571E-06	9 x 5	3 q13	ATPase H+ transporting V1 subunit A [Source:HGNC Symbol;Acc:HGNC:851]	
OSGEP	0,26	4,63	2,7536E-06	9 x 1	14 q11	O-sialoglycoprotein endopeptidase [Source:HGNC Symbol;Acc:HGNC:18028]	
RMND5A	0,26	4,63	2,7614E-06	8 x 2	2 p11	required for meiotic nuclear division 5 homolog A [Source:HGNC Symbol;Acc:HGNC:25850]	
WWOX	0,26	4,62	2,7748E-06	6 x 8	16 q23	WW domain containing oxidoreductase [Source:HGNC Symbol;Acc:HGNC:12799]	
UROS	0,26	4,61	2,9237E-06	1 x 8	10 q26	uroporphyrinogen III synthase [Source:HGNC Symbol;Acc:HGNC:12592]	
LMBRD1	0,26	4,61	2,9333E-06	8 x 1	6 q13	LMBR1 domain containing 1 [Source:HGNC Symbol;Acc:HGNC:23038]	
RHOQ	0,26	4,61	2,9502E-06	8 x 1	2 p21	ras homolog family member Q [Source:HGNC Symbol;Acc:HGNC:17736]	
FBXO21	0,25	4,60	3,0784E-06	9 x 1	12 q24	F-box protein 21 [Source:HGNC Symbol;Acc:HGNC:13592]	
MLX	0,25	4,59	3,25E-06	4 x 10	17 q21	MLX, MAX dimerization protein [Source:HGNC Symbol;Acc:HGNC:11645]	
GUK1	0,25	4,58	3,4204E-06	1 x 9	1 q42	guanylate kinase 1 [Source:HGNC Symbol;Acc:HGNC:4693]	
BTBD10	0,25	4,57	3,6032E-06	7 x 3	11 p15	BTB domain containing 10 [Source:HGNC Symbol;Acc:HGNC:21445]	
HNRNPLL	0,25	4,56	3,7023E-06	7 x 6	2 p22	heterogeneous nuclear ribonucleoprotein L like [Source:HGNC Symbol;Acc:HGNC:25127]	
ZNF197	0,25	4,54	4,0567E-06	9 x 5	CHR_HG2066_	zinc finger protein 197 [Source:HGNC Symbol;Acc:HGNC:12988]	
THOC1	0,25	4,52	4,3691E-06	5 x 6	18 p11	THO complex 1 [Source:HGNC Symbol;Acc:HGNC:19070]	
CFLAR	0,25	4,52	4,4099E-06	5 x 7	2 q33	CASP8 and FADD like apoptosis regulator [Source:HGNC Symbol;Acc:HGNC:1876]	
NUP214	0,25	4,51	4,5381E-06	3 x 8	9 q34	nucleoporin 214 [Source:HGNC Symbol;Acc:HGNC:8064]	
RALB	0,25	4,51	4,7181E-06	8 x 1	2 q14	RALB Ras like proto-oncogene B [Source:HGNC Symbol;Acc:HGNC:9840]	
PRCC	0,25	4,50	4,8751E-06	1 x 7	1 q23	papillary renal cell carcinoma (translocation-associated) [Source:HGNC Symbol;Acc:HGNC:9343]	
JMJD6	0,25	4,49	4,9961E-06	5 x 8	17 q25	arginine demethylase and lysine hydroxylase [Source:HGNC Symbol;Acc:HGNC:19355]	
RHBDD2	0,25	4,49	5,0816E-06	2 x 9	7 q11	rhomboid domain containing 2 [Source:HGNC Symbol;Acc:HGNC:23082]	
LYRM9	0,25	4,48	5,196E-06	9 x 6	17 q11	LYR motif containing 9 [Source:HGNC Symbol;Acc:HGNC:27314]	
MSANTD3	0,25	4,47	5,4138E-06	7 x 6	9 q31	Myb/SANT DNA binding domain containing 3 [Source:HGNC Symbol;Acc:HGNC:23370]	
HSPA4L	0,25	4,47	5,6288E-06	4 x 10	4 q28	heat shock protein family A (Hsp70) member 4 like [Source:HGNC Symbol;Acc:HGNC:17041]	
DYM	0,25	4,45	6,0751E-06	1 x 9	18 q21	dymeclin [Source:HGNC Symbol;Acc:HGNC:21317]	
UBXN6	0,25	4,44	6,255E-06	5 x 8	19 p13	UBX domain protein 6 [Source:HGNC Symbol;Acc:HGNC:14928]	
DUS3L	0,25	4,44	6,4284E-06	1 x 9	19 p13	dihydrouridine synthase 3 like [Source:HGNC Symbol;Acc:HGNC:26920]	
SH3BP5	0,24	4,41	7,2425E-06	9 x 5	3 p25	SH3 domain binding protein 5 [Source:HGNC Symbol;Acc:HGNC:10827]	
LPGAT1	0,24	4,41	7,2927E-06	8 x 1	1 q32	lysophosphatidylglycerol acyltransferase 1 [Source:HGNC Symbol;Acc:HGNC:28985]	
EYA3	0,24	4,40	7,3935E-06	7 x 2	1 p35	EYA transcriptional coactivator and phosphatase 3 [Source:HGNC Symbol;Acc:HGNC:3521]	
UBE2D2	0,24	4,40	7,5078E-06	4 x 9	5 q31	ubiquitin conjugating enzyme E2 D2 [Source:HGNC Symbol;Acc:HGNC:12475]	
HCFC2	0,24	4,39	7,8998E-06	9 x 4	12 q23	host cell factor C2 [Source:HGNC Symbol;Acc:HGNC:24972]	
NFIA	0,24	4,39	7,9538E-06	8 x 6	1 p31	nuclear factor I A [Source:HGNC Symbol;Acc:HGNC:7784]	
CXXC1	0,24	4,38	8,0429E-06	1 x 8	18 q21	CXXC finger protein 1 [Source:HGNC Symbol;Acc:HGNC:24343]	


C6orf106	0,24	4,38	8,2973E-06	8 x 3	6 p21	chromosome 6 open reading frame 106 [Source:HGNC Symbol;Acc:HGNC:21215]	
PCSK2	0,24	4,36	8,7441E-06	9 x 5	20 p12	proprotein convertase subtilisin/kexin type 2 [Source:HGNC Symbol;Acc:HGNC:8744]	
MMAB	0,24	4,36	9,0201E-06	1 x 10	12 q24	methylmalonic aciduria (cobalamin deficiency) cblB type [Source:HGNC Symbol;Acc:HGNC:19331]	
PPP2R3C	0,24	4,34	9,5364E-06	5 x 7	14 q13	protein phosphatase 2 regulatory subunit B''gamma [Source:HGNC Symbol;Acc:HGNC:17485]	
SF3B4	0,24	4,32	1,0378E-05	1 x 9	1 q21	splicing factor 3b subunit 4 [Source:HGNC Symbol;Acc:HGNC:10771]	
ARL8B	0,24	4,32	1,0391E-05	1 x 10	3 p26	ADP ribosylation factor like GTPase 8B [Source:HGNC Symbol;Acc:HGNC:25564]	
RALY	0,24	4,32	1,0558E-05	1 x 10	20 q11	RALY heterogeneous nuclear ribonucleoprotein [Source:HGNC Symbol;Acc:HGNC:15921]	
ZNF704	0,24	4,30	1,1293E-05	8 x 5	8 q21	zinc finger protein 704 [Source:HGNC Symbol;Acc:HGNC:32291]	
CHID1	0,24	4,29	1,2013E-05	1 x 8	11 p15	chitinase domain containing 1 [Source:HGNC Symbol;Acc:HGNC:28474]	
USP47	0,24	4,27	1,2806E-05	4 x 9	11 p15	ubiquitin specific peptidase 47 [Source:HGNC Symbol;Acc:HGNC:20076]	
DNAJC4	0,24	4,26	1,3428E-05	1 x 8	11 q13	DnaJ heat shock protein family (Hsp40) member C4 [Source:HGNC Symbol;Acc:HGNC:5271]	
ZFYVE26	0,24	4,26	1,3656E-05	9 x 7	14 q24	zinc finger FYVE-type containing 26 [Source:HGNC Symbol;Acc:HGNC:20761]	
GSTA3	0,24	4,26	1,3872E-05	9 x 3	6 p12	glutathione S-transferase alpha 3 [Source:HGNC Symbol;Acc:HGNC:4628]	
PDE6D	0,24	4,25	1,4275E-05	4 x 8	2 q37	phosphodiesterase 6D [Source:HGNC Symbol;Acc:HGNC:8788]	
PDHA1	0,24	4,24	1,4642E-05	1 x 9	X p22	pyruvate dehydrogenase (lipoamide) alpha 1 [Source:HGNC Symbol;Acc:HGNC:8806]	
SLC31A1	0,24	4,24	1,5072E-05	8 x 6	9 q32	solute carrier family 31 member 1 [Source:HGNC Symbol;Acc:HGNC:11016]	
PSMD13	0,24	4,23	1,5355E-05	3 x 10	11 p15	proteasome 26S subunit, non-ATPase 13 [Source:HGNC Symbol;Acc:HGNC:9558]	
NIPSNAP1	0,24	4,22	1,5855E-05	10 x 4	22 q12	nipsnap homolog 1 (C. elegans) [Source:HGNC Symbol;Acc:HGNC:7827]	
TMEM144	0,23	4,22	1,6122E-05	8 x 3	4 q32	transmembrane protein 144 [Source:HGNC Symbol;Acc:HGNC:25633]	
ANAPC7	0,23	4,22	1,6192E-05	8 x 1	12 q24	anaphase promoting complex subunit 7 [Source:HGNC Symbol;Acc:HGNC:17380]	
SNRPA	0,23	4,21	1,6504E-05	1 x 8	19 q13	small nuclear ribonucleoprotein polypeptide A [Source:HGNC Symbol;Acc:HGNC:11151]	
GBA2	0,23	4,20	1,7833E-05	9 x 6	9 p13	glucosylceramidase beta 2 [Source:HGNC Symbol;Acc:HGNC:18986]	
POLDIP2	0,23	4,19	1,8093E-05	1 x 10	17 q11	polymerase (DNA) delta interacting protein 2 [Source:HGNC Symbol;Acc:HGNC:23781]	
ALKBH5	0,23	4,18	1,9129E-05	8 x 6	17 p11	alkB homolog 5, RNA demethylase [Source:HGNC Symbol;Acc:HGNC:25996]	
TAF8	0,23	4,17	1,9877E-05	8 x 4	6 p21	TATA-box binding protein associated factor 8 [Source:HGNC Symbol;Acc:HGNC:17300]	
HMGN4	0,23	4,16	2,0356E-05	4 x 9	6 p22	high mobility group nucleosomal binding domain 4 [Source:HGNC Symbol;Acc:HGNC:4989]	
RNF213	0,23	4,16	2,0509E-05	9 x 6	17 q25	ring finger protein 213 [Source:HGNC Symbol;Acc:HGNC:14539]	
SPATS1	0,23	4,16	2,1105E-05	9 x 6	6 p21	spermatogenesis associated serine rich 1 [Source:HGNC Symbol;Acc:HGNC:22957]	
SF1	0,23	4,15	2,1622E-05	4 x 9	11 q13	splicing factor 1 [Source:HGNC Symbol;Acc:HGNC:12950]	
DIP2B	0,23	4,15	2,1837E-05	9 x 6	12 q13	disco interacting protein 2 homolog B [Source:HGNC Symbol;Acc:HGNC:29284]	
RNF114	0,23	4,15	2,1867E-05	4 x 10	20 q13	ring finger protein 114 [Source:HGNC Symbol;Acc:HGNC:13094]	
ZNF33B	0,23	4,14	2,2802E-05	9 x 7	10 q11	zinc finger protein 33B [Source:HGNC Symbol;Acc:HGNC:13097]	
ERAL1	0,23	4,14	2,2878E-05	5 x 8	17 q11	Era like 12S mitochondrial rRNA chaperone 1 [Source:HGNC Symbol;Acc:HGNC:3424]	


RAB29	0,23	4,13	2,3199E-05	8 x 7	1 q32	RAB29, member RAS oncogene family [Source:HGNC Symbol;Acc:HGNC:9789]	
TTC8	0,23	4,12	2,413E-05	9 x 7	14 q31	tetratricopeptide repeat domain 8 [Source:HGNC Symbol;Acc:HGNC:20087]	
PARL	0,23	4,11	2,4965E-05	1 x 9	3 q27	presenilin associated rhomboid like [Source:HGNC Symbol;Acc:HGNC:18253]	
ISOC1	0,23	4,10	2,6426E-05	4 x 9	5 q23	isochorismatase domain containing 1 [Source:HGNC Symbol;Acc:HGNC:24254]	
CGRRF1	0,23	4,09	2,7859E-05	5 x 7	14 q22	cell growth regulator with ring finger domain 1 [Source:HGNC Symbol;Acc:HGNC:15528]	
SNRPN	0,23	4,09	2,8169E-05	1 x 9	15 q11	small nuclear ribonucleoprotein polypeptide N [Source:HGNC Symbol;Acc:HGNC:11164]	
CXorf23	0,23	4,08	2,852E-05	8 x 4	X p22	chromosome X open reading frame 23 [Source:HGNC Symbol;Acc:HGNC:27413]	
GTF3C2	0,23	4,07	3,0027E-05	8 x 1	2 p23	general transcription factor IIIC subunit 2 [Source:HGNC Symbol;Acc:HGNC:4665]	
MRPL45	0,23	4,06	3,1191E-05	8 x 5	CHR_HSCHR17	mitochondrial ribosomal protein L45 [Source:HGNC Symbol;Acc:HGNC:16651]	
PSME3	0,23	4,05	3,2894E-05	1 x 10	17 q21	proteasome activator subunit 3 [Source:HGNC Symbol;Acc:HGNC:9570]	
DIP2A	0,23	4,04	3,3384E-05	9 x 6	21 q22	disco interacting protein 2 homolog A [Source:HGNC Symbol;Acc:HGNC:17217]	
RNLS	0,23	4,04	3,3763E-05	10 x 5	10 q23	renalase, FAD dependent amine oxidase [Source:HGNC Symbol;Acc:HGNC:25641]	
RRAGC	0,22	4,03	3,5391E-05	8 x 4	1 p34	Ras related GTP binding C [Source:HGNC Symbol;Acc:HGNC:19902]	
VAMP8	0,22	4,03	3,5647E-05	9 x 5	2 p11	vesicle associated membrane protein 8 [Source:HGNC Symbol;Acc:HGNC:12647]	
NAA25	0,22	4,02	3,6724E-05	10 x 4	12 q24	N(alpha)-acetyltransferase 25, NatB auxiliary subunit [Source:HGNC Symbol;Acc:HGNC:25783]	
LUC7L	0,22	4,01	3,7955E-05	8 x 6	16 p13	LUC7 like [Source:HGNC Symbol;Acc:HGNC:6723]	
IMPACT	0,22	4,01	3,8143E-05	1 x 10	18 q11	impact RWD domain protein [Source:HGNC Symbol;Acc:HGNC:20387]	
RHOT1	0,22	4,00	3,9365E-05	9 x 1	17 q11	ras homolog family member T1 [Source:HGNC Symbol;Acc:HGNC:21168]	
TCF12	0,22	4,00	3,9482E-05	2 x 10	15 q21	transcription factor 12 [Source:HGNC Symbol;Acc:HGNC:11623]	
STUB1	0,22	4,00	3,9508E-05	5 x 9	16 p13	STIP1 homology and U-box containing protein 1 [Source:HGNC Symbol;Acc:HGNC:11427]	
BLCAP	0,22	4,00	4,0103E-05	6 x 9	20 q11	bladder cancer associated protein [Source:HGNC Symbol;Acc:HGNC:1055]	
NUBP2	0,22	3,98	4,2883E-05	1 x 9	16 p13	nucleotide binding protein 2 [Source:HGNC Symbol;Acc:HGNC:8042]	
APRT	0,22	3,98	4,2998E-05	1 x 9	16 q24	adenine phosphoribosyltransferase [Source:HGNC Symbol;Acc:HGNC:626]	
CSRP2	0,22	3,97	4,4414E-05	1 x 7	12 q21	cysteine and glycine rich protein 2 [Source:HGNC Symbol;Acc:HGNC:2470]	
TSPAN6	0,22	3,96	4,6539E-05	9 x 1	X q22	tetraspanin 6 [Source:HGNC Symbol;Acc:HGNC:11858]	
HIST1H2BD	0,22	3,95	4,7783E-05	3 x 9	6 p22	histone cluster 1, H2bd [Source:HGNC Symbol;Acc:HGNC:4747]	
SORD	0,22	3,95	4,8905E-05	9 x 1	15 q21	sorbitol dehydrogenase [Source:HGNC Symbol;Acc:HGNC:11184]	
DCP1A	0,22	3,95	4,9259E-05	9 x 1	3 p21	decapping mRNA 1A [Source:HGNC Symbol;Acc:HGNC:18714]	
TRAF5	0,22	3,95	4,9448E-05	9 x 4	1 q32	TNF receptor associated factor 5 [Source:HGNC Symbol;Acc:HGNC:12035]	
ENTPD6	0,22	3,94	5,0015E-05	2 x 8	20 p11	ectonucleoside triphosphate diphosphohydrolase 6 (putative) [Source:HGNC Symbol;Acc:HGNC:3368]	
SLC17A5	0,22	3,92	5,4129E-05	9 x 3	6 q13	solute carrier family 17 member 5 [Source:HGNC Symbol;Acc:HGNC:10933]	
QSER1	0,22	3,90	5,9993E-05	9 x 5	11 p13	glutamine and serine rich 1 [Source:HGNC Symbol;Acc:HGNC:26154]	
CHD6	0,22	3,89	6,2099E-05	8 x 5	20 q12	chromodomain helicase DNA binding protein 6 [Source:HGNC Symbol;Acc:HGNC:19057]	


PTBP3	0,21	3,84	7,3553E-05	9 x 1	9 q32	polypyrimidine tract binding protein 3 [Source:HGNC Symbol;Acc:HGNC:10253]	
TRIM5	0,21	3,84	7,4394E-05	8 x 5	11 p15	tripartite motif containing 5 [Source:HGNC Symbol;Acc:HGNC:16276]	
SRSF4	0,21	3,83	7,6656E-05	6 x 9	1 p35	serine and arginine rich splicing factor 4 [Source:HGNC Symbol;Acc:HGNC:10786]	
APPL1	0,21	3,82	8,0108E-05	8 x 1	3 p14	adaptor protein, phosphotyrosine interacting with PH domain and leucine zipper 1 [Source:HGNC Symbol;Acc:HGNC:24035]	
IQCK	0,21	3,82	8,066E-05	8 x 4	16 p12	IQ motif containing K [Source:HGNC Symbol;Acc:HGNC:28556]	
LCMT1	0,21	3,82	8,1924E-05	1 x 8	16 p12	leucine carboxyl methyltransferase 1 [Source:HGNC Symbol;Acc:HGNC:17557]	
TTC9C	0,21	3,81	8,3123E-05	5 x 8	11 q12	tetratricopeptide repeat domain 9C [Source:HGNC Symbol;Acc:HGNC:28432]	
SLC35C2	0,21	3,80	8,735E-05	1 x 9	20 q13	solute carrier family 35 member C2 [Source:HGNC Symbol;Acc:HGNC:17117]	
USP51	0,21	3,77	9,6855E-05	9 x 1	X p11	ubiquitin specific peptidase 51 [Source:HGNC Symbol;Acc:HGNC:23086]	
IFNGR1	0,21	3,74	0,00011158	8 x 6	6 q23	interferon gamma receptor 1 [Source:HGNC Symbol;Acc:HGNC:5439]	
DDX17	0,21	3,73	0,00011582	9 x 5	22 q13	DEAD-box helicase 17 [Source:HGNC Symbol;Acc:HGNC:2740]	
CHD4	0,21	3,72	0,00011758	1 x 8	12 p13	chromodomain helicase DNA binding protein 4 [Source:HGNC Symbol;Acc:HGNC:1919]	
RETSAT	0,21	3,72	0,00011832	10 x 6	2 p11	retinol saturase [Source:HGNC Symbol;Acc:HGNC:25991]	
USB1	0,21	3,70	0,00012936	1 x 9	16 q21	U6 snRNA biogenesis phosphodiesterase 1 [Source:HGNC Symbol;Acc:HGNC:25792]	
FECH	0,21	3,68	0,00013791	8 x 3	18 q21	ferrochelatase [Source:HGNC Symbol;Acc:HGNC:3647]	
SMAD2	0,20	3,66	0,00015033	9 x 2	18 q21	SMAD family member 2 [Source:HGNC Symbol;Acc:HGNC:6768]	
NPRL2	0,20	3,59	0,0001918	1 x 8	3 p21	NPR2-like, GATOR1 complex subunit [Source:HGNC Symbol;Acc:HGNC:24969]	
DPP9	0,20	3,57	0,00020696	5 x 10	19 p13	dipeptidyl peptidase 9 [Source:HGNC Symbol;Acc:HGNC:18648]	
ELP6	0,20	3,57	0,00020942	4 x 9	3 p21	elongator acetyltransferase complex subunit 6 [Source:HGNC Symbol;Acc:HGNC:25976]	
ANKRD12	0,20	3,56	0,00021149	8 x 5	18 p11	ankyrin repeat domain 12 [Source:HGNC Symbol;Acc:HGNC:29135]	
ASPH	0,20	3,56	0,0002169	1 x 8	8 q12	aspartate beta-hydroxylase [Source:HGNC Symbol;Acc:HGNC:757]	
CMTR2	0,20	3,56	0,00021786	9 x 3	CHR_HSCHR16	cap methyltransferase 2 [Source:HGNC Symbol;Acc:HGNC:25635]	
RBM4B	0,20	3,54	0,0002295	4 x 9	11 q13	RNA binding motif protein 4B [Source:HGNC Symbol;Acc:HGNC:28842]	
UBE2J2	0,20	3,54	0,00023201	4 x 9	1 p36	ubiquitin conjugating enzyme E2 J2 [Source:HGNC Symbol;Acc:HGNC:19268]	
PUS7L	0,20	3,53	0,00024199	8 x 1	12 q12	pseudouridylate synthase 7 like [Source:HGNC Symbol;Acc:HGNC:25276]	
ZNF254	0,20	3,52	0,00024442	9 x 6	19 p12	zinc finger protein 254 [Source:HGNC Symbol;Acc:HGNC:13047]	
RBCK1	0,20	3,52	0,00025026	1 x 9	20 p13	RANBP2-type and C3HC4-type zinc finger containing 1 [Source:HGNC Symbol;Acc:HGNC:15864]	
GLB1	0,20	3,52	0,00025047	1 x 10	3 p22	galactosidase beta 1 [Source:HGNC Symbol;Acc:HGNC:4298]	
MTFMT	0,20	3,52	0,00025195	9 x 6	15 q22	mitochondrial methionyl-tRNA formyltransferase [Source:HGNC Symbol;Acc:HGNC:29666]	
FASTKD1	0,20	3,49	0,00027785	9 x 1	2 q31	FAST kinase domains 1 [Source:HGNC Symbol;Acc:HGNC:26150]	
CNOT10	0,20	3,48	0,00028644	1 x 8	3 p22	CCR4-NOT transcription complex subunit 10 [Source:HGNC Symbol;Acc:HGNC:23817]	
NPC1	0,19	3,47	0,00030064	2 x 8	18 q11	NPC intracellular cholesterol transporter 1 [Source:HGNC Symbol;Acc:HGNC:7897]	
C14orf142	0,19	3,45	0,00032083	9 x 7	CHR_HSCHR14	chromosome 14 open reading frame 142 [Source:HGNC Symbol;Acc:HGNC:20356]	


ZNF326	0,19	3,45	0,00032477	9 x 2	1 p22	zinc finger protein 326 [Source:HGNC Symbol;Acc:HGNC:14104]	
SLC35B4	0,19	3,42	0,00035126	8 x 6	7 q33	solute carrier family 35 member B4 [Source:HGNC Symbol;Acc:HGNC:20584]	
ZFP64	0,19	3,42	0,00035354	9 x 4	20 q13	ZFP64 zinc finger protein [Source:HGNC Symbol;Acc:HGNC:15940]	
MTFR1L	0,19	3,39	0,00040038	4 x 10	1 p36	mitochondrial fission regulator 1 like [Source:HGNC Symbol;Acc:HGNC:28836]	
AIFM1	0,19	3,37	0,00043016	8 x 3	X q26	apoptosis inducing factor, mitochondria associated 1 [Source:HGNC Symbol;Acc:HGNC:8768]	
RAB9A	0,19	3,37	0,00043074	1 x 9	X p22	RAB9A, member RAS oncogene family [Source:HGNC Symbol;Acc:HGNC:9792]	
ZNF138	0,19	3,35	0,00045576	9 x 5	7 q11	zinc finger protein 138 [Source:HGNC Symbol;Acc:HGNC:12922]	
CBWD1	0,19	3,33	0,00048473	9 x 7	9 p24	COBW domain containing 1 [Source:HGNC Symbol;Acc:HGNC:17134]	
ISG20L2	0,19	3,33	0,00048632	10 x 5	1 q23	interferon stimulated exonuclease gene 20 like 2 [Source:HGNC Symbol;Acc:HGNC:25745]	
EN2	0,18	3,26	0,00062904	9 x 3	7 q36	engrailed homeobox 2 [Source:HGNC Symbol;Acc:HGNC:3343]	
TINF2	0,18	3,25	0,00064844	1 x 10	14 q12	TERF1 interacting nuclear factor 2 [Source:HGNC Symbol;Acc:HGNC:11824]	
UEVLD	0,18	3,24	0,00065323	9 x 5	11 p15	UEV and lactate/malate dehyrogenase domains [Source:HGNC Symbol;Acc:HGNC:30866]	
SAFB	0,18	3,23	0,00067542	1 x 10	19 p13	scaffold attachment factor B [Source:HGNC Symbol;Acc:HGNC:10520]	
DDHD1	0,18	3,19	0,00077285	9 x 1	14 q22	DDHD domain containing 1 [Source:HGNC Symbol;Acc:HGNC:19714]	
ZNF567	0,18	3,19	0,00079194	9 x 6	19 q13	zinc finger protein 567 [Source:HGNC Symbol;Acc:HGNC:28696]	
GMPR2	0,18	3,18	0,00081701	1 x 8	14 q12	guanosine monophosphate reductase 2 [Source:HGNC Symbol;Acc:HGNC:4377]	
RABGGTA	0,18	3,14	0,00091706	1 x 10	14 q12	Rab geranylgeranyltransferase alpha subunit [Source:HGNC Symbol;Acc:HGNC:9795]	
FAM208A	0,17	3,10	0,00104982	9 x 1	3 p14	family with sequence similarity 208 member A [Source:HGNC Symbol;Acc:HGNC:30314]	
GGA3	0,17	3,09	0,0010922	9 x 6	17 q25	golgi associated, gamma adaptin ear containing, ARF binding protein 3 [Source:HGNC Symbol;Acc:HGNC:17079]	
MKS1	0,17	3,07	0,00115262	9 x 6	17 q22	Meckel syndrome, type 1 [Source:HGNC Symbol;Acc:HGNC:7121]	
PAM16	0,17	3,06	0,00121176	1 x 9	16 p13	presequence translocase-associated motor 16 homolog (S. cerevisiae) [Source:HGNC Symbol;Acc:HGNC:29679]	
HDAC2	0,17	3,06	0,00122054	1 x 10	6 q21	histone deacetylase 2 [Source:HGNC Symbol;Acc:HGNC:4853]	
JAG1	0,17	3,04	0,00128475	9 x 2	20 p12	jagged 1 [Source:HGNC Symbol;Acc:HGNC:6188]	
SLC7A6	0,17	3,03	0,00133553	9 x 1	16 q22	solute carrier family 7 member 6 [Source:HGNC Symbol;Acc:HGNC:11064]	
RFXANK	0,17	2,97	0,00158572	1 x 9	19 p13	regulatory factor X associated ankyrin containing protein [Source:HGNC Symbol;Acc:HGNC:9987]	
OGDH	0,17	2,93	0,00180362	10 x 4	7 p13	oxoglutarate dehydrogenase [Source:HGNC Symbol;Acc:HGNC:8124]	
RIN2	0,16	2,92	0,00187745	8 x 3	20 p11	Ras and Rab interactor 2 [Source:HGNC Symbol;Acc:HGNC:18750]	
ARL14EP	0,16	2,87	0,00219013	9 x 7	11 p14	ADP ribosylation factor like GTPase 14 effector protein [Source:HGNC Symbol;Acc:HGNC:26798]	
VPS13D	0,16	2,85	0,00234346	9 x 6	1 p36	vacuolar protein sorting 13 homolog D [Source:HGNC Symbol;Acc:HGNC:23595]	
HAUS7	0,16	2,83	0,00247053	1 x 10	X q28	HAUS augmin like complex subunit 7 [Source:HGNC Symbol;Acc:HGNC:32979]	
GGPS1	0,16	2,83	0,00248623	1 x 10	1 q42	geranylgeranyl diphosphate synthase 1 [Source:HGNC Symbol;Acc:HGNC:4249]	
PIGC	0,16	2,82	0,00255954	1 x 9	1 q24	phosphatidylinositol glycan anchor biosynthesis class C [Source:HGNC Symbol;Acc:HGNC:8960]	
UCK2	0,16	2,82	0,00258392	1 x 10	1 q24	uridine-cytidine kinase 2 [Source:HGNC Symbol;Acc:HGNC:12562]	


YTHDF1	0,15	2,69	0,00376306	9 x 6	20 q13	YTH N6-methyladenosine RNA binding protein 1 [Source:HGNC Symbol;Acc:HGNC:15867]	
TCF25	0,15	2,62	0,00463696	1 x 8	16 q24	transcription factor 25 [Source:HGNC Symbol;Acc:HGNC:29181]	
RBM19	0,15	2,61	0,00472007	10 x 6	12 q24	RNA binding motif protein 19 [Source:HGNC Symbol;Acc:HGNC:29098]	
CREG1	0,14	2,41	0,00823654	9 x 7	1 q24	cellular repressor of E1A stimulated genes 1 [Source:HGNC Symbol;Acc:HGNC:2351]	
WDR45	0,13	2,28	0,01178298	1 x 10	X p11	WD repeat domain 45 [Source:HGNC Symbol;Acc:HGNC:28912]	
GNPNAT1	0,13	2,27	0,01202928	1 x 10	14 q22	glucosamine-phosphate N-acetyltransferase 1 [Source:HGNC Symbol;Acc:HGNC:19980]	
MOSPD1	0,08	1,46	0,07283793	10 x 4	X q26	motile sperm domain containing 1 [Source:HGNC Symbol;Acc:HGNC:25235]	


Genes upregulated in mut/wt (BRAF_mut)	
Symbol	correlation	->t.score	->p.value	Metagene	Chromosome	Description	
CD36	0,91	39,47	0	1 x 50	7 q21	CD36 molecule [Source:HGNC Symbol;Acc:HGNC:1663]	
CBR1	0,86	29,35	0	1 x 50	21 q22	carbonyl reductase 1 [Source:HGNC Symbol;Acc:HGNC:1548]	
NELL1	0,86	29,19	0	1 x 50	11 p15	neural EGFL like 1 [Source:HGNC Symbol;Acc:HGNC:7750]	
SNX10	0,83	25,95	0	1 x 50	7 p15	sorting nexin 10 [Source:HGNC Symbol;Acc:HGNC:14974]	
HTR2B	0,82	25,09	0	1 x 50	2 q37	5-hydroxytryptamine receptor 2B [Source:HGNC Symbol;Acc:HGNC:5294]	
PLCD1	0,81	24,32	0	1 x 50	3 p22	phospholipase C delta 1 [Source:HGNC Symbol;Acc:HGNC:9060]	
PRDX2	0,81	24,01	0	1 x 50	19 p13	peroxiredoxin 2 [Source:HGNC Symbol;Acc:HGNC:9353]	
CFI	0,78	21,80	0	1 x 50	4 q25	complement factor I [Source:HGNC Symbol;Acc:HGNC:5394]	
BCAS3	0,75	20,09	0	1 x 50	17 q23	breast carcinoma amplified sequence 3 [Source:HGNC Symbol;Acc:HGNC:14347]	
MOXD1	0,74	19,50	0	1 x 50	6 q23	monooxygenase DBH like 1 [Source:HGNC Symbol;Acc:HGNC:21063]	
LGI4	0,74	19,08	0	1 x 50	19 q13	leucine rich repeat LGI family member 4 [Source:HGNC Symbol;Acc:HGNC:18712]	
ANGPT2	0,73	18,53	0	1 x 50	8 p23	angiopoietin 2 [Source:HGNC Symbol;Acc:HGNC:485]	
SHISA2	0,72	18,33	0	1 x 50	13 q12	shisa family member 2 [Source:HGNC Symbol;Acc:HGNC:20366]	
SLITRK2	0,72	18,07	0	1 x 50	X q27	SLIT and NTRK like family member 2 [Source:HGNC Symbol;Acc:HGNC:13449]	
FXYD3	0,72	17,93	0	1 x 50	19 q13	FXYD domain containing ion transport regulator 3 [Source:HGNC Symbol;Acc:HGNC:4027]	
SDC2	0,72	17,90	0	1 x 50	8 q22	syndecan 2 [Source:HGNC Symbol;Acc:HGNC:10659]	
LY96	0,71	17,77	0	1 x 50	8 q21	lymphocyte antigen 96 [Source:HGNC Symbol;Acc:HGNC:17156]	
PTPRZ1	0,70	17,20	0	1 x 50	7 q31	protein tyrosine phosphatase, receptor type Z1 [Source:HGNC Symbol;Acc:HGNC:9685]	
ZNF880	0,70	16,95	0	1 x 50	19 q13	zinc finger protein 880 [Source:HGNC Symbol;Acc:HGNC:37249]	
IFITM2	0,69	16,61	0	1 x 49	11 p15	interferon induced transmembrane protein 2 [Source:HGNC Symbol;Acc:HGNC:5413]	
FAP	0,69	16,58	0	1 x 50	2 q24	fibroblast activation protein alpha [Source:HGNC Symbol;Acc:HGNC:3590]	
	0,67	15,86	0	1 x 50			
CARD16	0,67	15,67	0	1 x 50	11 q22	caspase recruitment domain family member 16 [Source:HGNC Symbol;Acc:HGNC:33701]	
LRP2	0,66	15,37	0	1 x 50	2 q31	LDL receptor related protein 2 [Source:HGNC Symbol;Acc:HGNC:6694]	
RNASE1	0,66	15,20	0	1 x 50	14 q11	ribonuclease A family member 1, pancreatic [Source:HGNC Symbol;Acc:HGNC:10044]	
ZNF783	0,65	15,05	0	1 x 50	7 q36	zinc finger family member 783 [Source:HGNC Symbol;Acc:HGNC:27222]	
LZTS1	0,65	15,01	0	1 x 50	8 p21	leucine zipper, putative tumor suppressor 1 [Source:HGNC Symbol;Acc:HGNC:13861]	
PRKCDBP	0,65	14,88	0	1 x 50	11 p15	protein kinase C delta binding protein [Source:HGNC Symbol;Acc:HGNC:9400]	
NOV	0,64	14,70	0	1 x 49	8 q24	nephroblastoma overexpressed [Source:HGNC Symbol;Acc:HGNC:7885]	
PYGL	0,64	14,64	0	1 x 50	14 q22	phosphorylase, glycogen, liver [Source:HGNC Symbol;Acc:HGNC:9725]	
SESN3	0,64	14,47	0	1 x 50	11 q21	sestrin 3 [Source:HGNC Symbol;Acc:HGNC:23060]	


HOXB3	0,64	14,38	0	1 x 50	17 q21	homeobox B3 [Source:HGNC Symbol;Acc:HGNC:5114]	
PCDHB2	0,63	14,12	0	2 x 49	5 q31	protocadherin beta 2 [Source:HGNC Symbol;Acc:HGNC:8687]	
RTP4	0,62	13,89	0	1 x 50	3 q27	receptor transporter protein 4 [Source:HGNC Symbol;Acc:HGNC:23992]	
CYP26A1	0,62	13,81	0	1 x 50	10 q23	cytochrome P450 family 26 subfamily A member 1 [Source:HGNC Symbol;Acc:HGNC:2603]	
CASP1	0,61	13,60	0	1 x 50	11 q22	caspase 1 [Source:HGNC Symbol;Acc:HGNC:1499]	
JOSD2	0,61	13,56	0	1 x 50	19 q13	Josephin domain containing 2 [Source:HGNC Symbol;Acc:HGNC:28853]	
CYGB	0,61	13,52	0	1 x 50	17 q25	cytoglobin [Source:HGNC Symbol;Acc:HGNC:16505]	
MGP	0,61	13,52	0	1 x 50	12 p12	matrix Gla protein [Source:HGNC Symbol;Acc:HGNC:7060]	
MRPL41	0,61	13,28	0	1 x 48	9 q34	mitochondrial ribosomal protein L41 [Source:HGNC Symbol;Acc:HGNC:14492]	
LXN	0,61	13,28	0	1 x 49	3 q25	latexin [Source:HGNC Symbol;Acc:HGNC:13347]	
ZNF528	0,60	13,25	0	1 x 50	19 q13	zinc finger protein 528 [Source:HGNC Symbol;Acc:HGNC:29384]	
CD320	0,59	12,77	0	1 x 50	19 p13	CD320 molecule [Source:HGNC Symbol;Acc:HGNC:16692]	
IFITM3	0,59	12,65	0	1 x 50	11 p15	interferon induced transmembrane protein 3 [Source:HGNC Symbol;Acc:HGNC:5414]	
CTTNBP2	0,59	12,63	0	1 x 50	7 q31	cortactin binding protein 2 [Source:HGNC Symbol;Acc:HGNC:15679]	
NR2F1	0,58	12,39	0	7 x 50	5 q15	nuclear receptor subfamily 2 group F member 1 [Source:HGNC Symbol;Acc:HGNC:7975]	
NINJ1	0,58	12,39	0	1 x 50	9 q22	ninjurin 1 [Source:HGNC Symbol;Acc:HGNC:7824]	
ZDHHC12	0,58	12,32	0	1 x 50	9 q34	zinc finger DHHC-type containing 12 [Source:HGNC Symbol;Acc:HGNC:19159]	
CTSB	0,57	12,20	0	1 x 50	8 p23	cathepsin B [Source:HGNC Symbol;Acc:HGNC:2527]	
HMG20B	0,57	12,18	0	1 x 46	19 p13	high mobility group 20B [Source:HGNC Symbol;Acc:HGNC:5002]	
PERP	0,57	12,17	0	1 x 50	6 q23	PERP, TP53 apoptosis effector [Source:HGNC Symbol;Acc:HGNC:17637]	
ZNF667	0,57	12,15	0	4 x 50	19 q13	zinc finger protein 667 [Source:HGNC Symbol;Acc:HGNC:28854]	
LGALS3	0,57	12,05	0	1 x 48	14 q22	galectin 3 [Source:HGNC Symbol;Acc:HGNC:6563]	
C19orf60	0,57	12,01	0	1 x 49	19 p13	chromosome 19 open reading frame 60 [Source:HGNC Symbol;Acc:HGNC:26098]	
SYTL2	0,57	11,97	0	1 x 50	11 q14	synaptotagmin like 2 [Source:HGNC Symbol;Acc:HGNC:15585]	
ABCA1	0,56	11,96	0	3 x 50	9 q31	ATP binding cassette subfamily A member 1 [Source:HGNC Symbol;Acc:HGNC:29]	
CFL1	0,56	11,93	0	1 x 44	11 q13	cofilin 1 [Source:HGNC Symbol;Acc:HGNC:1874]	
LRRTM4	0,56	11,88	0	2 x 50	2 p12	leucine rich repeat transmembrane neuronal 4 [Source:HGNC Symbol;Acc:HGNC:19411]	
AGGF1	0,56	11,86	0	2 x 50	5 q13	angiogenic factor with G-patch and FHA domains 1 [Source:HGNC Symbol;Acc:HGNC:24684]	
FAM84B	0,56	11,74	0	1 x 50	8 q24	family with sequence similarity 84 member B [Source:HGNC Symbol;Acc:HGNC:24166]	
ZNF677	0,56	11,72	0	2 x 48	19 q13	zinc finger protein 677 [Source:HGNC Symbol;Acc:HGNC:28730]	
INPP5K	0,56	11,71	0	1 x 48	17 p13	inositol polyphosphate-5-phosphatase K [Source:HGNC Symbol;Acc:HGNC:33882]	
KLHL40	0,56	11,71	0	5 x 49	3 p22	kelch like family member 40 [Source:HGNC Symbol;Acc:HGNC:30372]	
ENDOD1	0,56	11,67	0	1 x 50	11 q21	endonuclease domain containing 1 [Source:HGNC Symbol;Acc:HGNC:29129]	


RNF130	0,56	11,67	0	1 x 48	5 q35	ring finger protein 130 [Source:HGNC Symbol;Acc:HGNC:18280]	
THBS2	0,55	11,39	0	4 x 50	6 q27	thrombospondin 2 [Source:HGNC Symbol;Acc:HGNC:11786]	
PLA2G4A	0,54	11,32	0	1 x 50	1 q31	phospholipase A2 group IVA [Source:HGNC Symbol;Acc:HGNC:9035]	
APLP2	0,54	11,26	0	1 x 49	11 q24	amyloid beta precursor like protein 2 [Source:HGNC Symbol;Acc:HGNC:598]	
IRF4	0,54	11,20	0	1 x 49	6 p25	interferon regulatory factor 4 [Source:HGNC Symbol;Acc:HGNC:6119]	
CADPS	0,54	11,15	0	1 x 50	3 p14	calcium dependent secretion activator [Source:HGNC Symbol;Acc:HGNC:1426]	
CASP4	0,54	11,11	0	1 x 50	11 q22	caspase 4 [Source:HGNC Symbol;Acc:HGNC:1505]	
PHPT1	0,53	10,91	0	1 x 47	9 q34	phosphohistidine phosphatase 1 [Source:HGNC Symbol;Acc:HGNC:30033]	
ZNF320	0,53	10,86	0	3 x 49	19 q13	zinc finger protein 320 [Source:HGNC Symbol;Acc:HGNC:13842]	
PCDHGC3	0,53	10,79	0	1 x 50	5 q31	protocadherin gamma subfamily C, 3 [Source:HGNC Symbol;Acc:HGNC:8716]	
ZNF415	0,52	10,75	0	3 x 48	19 q13	zinc finger protein 415 [Source:HGNC Symbol;Acc:HGNC:20636]	
IDO1	0,52	10,70	0	3 x 49	8 p11	indoleamine 2,3-dioxygenase 1 [Source:HGNC Symbol;Acc:HGNC:6059]	
GADD45GIP1	0,52	10,65	0	1 x 47	19 p13	GADD45G interacting protein 1 [Source:HGNC Symbol;Acc:HGNC:29996]	
TRAPPC5	0,52	10,61	0	1 x 46	19 p13	trafficking protein particle complex 5 [Source:HGNC Symbol;Acc:HGNC:23067]	
SERINC5	0,52	10,58	0	1 x 50	5 q14	serine incorporator 5 [Source:HGNC Symbol;Acc:HGNC:18825]	
PFN1	0,52	10,58	0	1 x 44	17 p13	profilin 1 [Source:HGNC Symbol;Acc:HGNC:8881]	
PCDHGA10	0,52	10,57	0	3 x 50	5 q31	protocadherin gamma subfamily A, 10 [Source:HGNC Symbol;Acc:HGNC:8697]	
RHOBTB3	0,52	10,50	0	1 x 50	5 q15	Rho related BTB domain containing 3 [Source:HGNC Symbol;Acc:HGNC:18757]	
FGF12	0,51	10,48	0	4 x 50	3 q29	fibroblast growth factor 12 [Source:HGNC Symbol;Acc:HGNC:3668]	
PINX1	0,51	10,45	0	1 x 50	8 p23	PIN2/TERF1 interacting, telomerase inhibitor 1 [Source:HGNC Symbol;Acc:HGNC:30046]	
PCDH9	0,51	10,44	0	2 x 50	13 q21	protocadherin 9 [Source:HGNC Symbol;Acc:HGNC:8661]	
ATP6V1B2	0,51	10,44	0	1 x 50	8 p21	ATPase H+ transporting V1 subunit B2 [Source:HGNC Symbol;Acc:HGNC:854]	
ARRDC3	0,51	10,40	0	1 x 50	5 q14	arrestin domain containing 3 [Source:HGNC Symbol;Acc:HGNC:29263]	
CHL1	0,51	10,35	0	1 x 50	3 p26	cell adhesion molecule L1 like [Source:HGNC Symbol;Acc:HGNC:1939]	
CELF2	0,51	10,27	0	1 x 49	10 p14	CUGBP, Elav-like family member 2 [Source:HGNC Symbol;Acc:HGNC:2550]	
REXO2	0,51	10,22	0	1 x 50	11 q23	RNA exonuclease 2 [Source:HGNC Symbol;Acc:HGNC:17851]	
NUDT22	0,50	10,22	0	1 x 47	11 q13	nudix hydrolase 22 [Source:HGNC Symbol;Acc:HGNC:28189]	
GMPR	0,50	10,20	0	1 x 49	6 p22	guanosine monophosphate reductase [Source:HGNC Symbol;Acc:HGNC:4376]	
SIGIRR	0,50	10,18	0	1 x 49	11 p15	single immunoglobulin and toll-interleukin 1 receptor (TIR) domain [Source:HGNC Symbol;Acc:HGNC:30575]	
C21orf33	0,50	10,07	0	1 x 44	21 q22	chromosome 21 open reading frame 33 [Source:HGNC Symbol;Acc:HGNC:1273]	
SAT1	0,50	9,98	0	1 x 50	X p22	spermidine/spermine N1-acetyltransferase 1 [Source:HGNC Symbol;Acc:HGNC:10540]	
CTSK	0,49	9,89	0	1 x 50	1 q21	cathepsin K [Source:HGNC Symbol;Acc:HGNC:2536]	
C11orf31	0,49	9,88	0	1 x 46	11 q12	chromosome 11 open reading frame 31 [Source:HGNC Symbol;Acc:HGNC:18251]	


BCHE	0,49	9,84	0	1 x 50	3 q26	butyrylcholinesterase [Source:HGNC Symbol;Acc:HGNC:983]	
DNAJC15	0,49	9,82	0	1 x 47	13 q14	DnaJ heat shock protein family (Hsp40) member C15 [Source:HGNC Symbol;Acc:HGNC:20325]	
SYNM	0,49	9,72	0	3 x 50	15 q26	synemin [Source:HGNC Symbol;Acc:HGNC:24466]	
C7orf50	0,49	9,72	0	1 x 46	7 p22	chromosome 7 open reading frame 50 [Source:HGNC Symbol;Acc:HGNC:22421]	
GSN	0,49	9,69	0	1 x 49	9 q33	gelsolin [Source:HGNC Symbol;Acc:HGNC:4620]	
MFSD12	0,48	9,61	0	1 x 46	19 p13	major facilitator superfamily domain containing 12 [Source:HGNC Symbol;Acc:HGNC:28299]	
SCN1B	0,48	9,61	0	5 x 48	19 q13	sodium voltage-gated channel beta subunit 1 [Source:HGNC Symbol;Acc:HGNC:10586]	
PCDHB11	0,48	9,60	0	4 x 48	5 q31	protocadherin beta 11 [Source:HGNC Symbol;Acc:HGNC:8682]	
MICA	0,48	9,60	0	4 x 50	CHR_HSCHR6_	MHC class I polypeptide-related sequence A [Source:HGNC Symbol;Acc:HGNC:7090]	
MLLT11	0,48	9,51	0	1 x 50	1 q21	myeloid/lymphoid or mixed-lineage leukemia; translocated to, 11 [Source:HGNC Symbol;Acc:HGNC:16997]	
FAM110B	0,48	9,44	0	2 x 50	8 q12	family with sequence similarity 110 member B [Source:HGNC Symbol;Acc:HGNC:28587]	
TRIM51	0,48	9,44	0	4 x 48	11 q12	tripartite motif-containing 51 [Source:HGNC Symbol;Acc:HGNC:19023]	
SLC35F5	0,48	9,43	0	1 x 50	2 q14	solute carrier family 35 member F5 [Source:HGNC Symbol;Acc:HGNC:23617]	
RNASEH2C	0,47	9,42	0	1 x 47	11 q13	ribonuclease H2 subunit C [Source:HGNC Symbol;Acc:HGNC:24116]	
CA8	0,47	9,30	0	3 x 50	8 q12	carbonic anhydrase 8 [Source:HGNC Symbol;Acc:HGNC:1382]	
MTSS1	0,47	9,28	0	1 x 49	8 q24	metastasis suppressor 1 [Source:HGNC Symbol;Acc:HGNC:20443]	
CYC1	0,47	9,24	0	1 x 45	8 q24	cytochrome c1 [Source:HGNC Symbol;Acc:HGNC:2579]	
SRGN	0,47	9,23	0	5 x 49	10 q22	serglycin [Source:HGNC Symbol;Acc:HGNC:9361]	
ZNF521	0,47	9,22	0	5 x 50	18 q11	zinc finger protein 521 [Source:HGNC Symbol;Acc:HGNC:24605]	
CPT1A	0,47	9,18	0	4 x 50	11 q13	carnitine palmitoyltransferase 1A [Source:HGNC Symbol;Acc:HGNC:2328]	
ZNF570	0,47	9,18	0	3 x 49	19 q13	zinc finger protein 570 [Source:HGNC Symbol;Acc:HGNC:26416]	
CD44	0,46	9,17	0	6 x 49	11 p13	CD44 molecule (Indian blood group) [Source:HGNC Symbol;Acc:HGNC:1681]	
LDHA	0,46	9,16	0	2 x 45	11 p15	lactate dehydrogenase A [Source:HGNC Symbol;Acc:HGNC:6535]	
ADA	0,46	9,14	0	1 x 50	20 q13	adenosine deaminase [Source:HGNC Symbol;Acc:HGNC:186]	
IMPDH1	0,46	9,13	0	1 x 47	7 q32	IMP (inosine 5'-monophosphate) dehydrogenase 1 [Source:HGNC Symbol;Acc:HGNC:6052]	
PCDH7	0,46	9,12	0	1 x 50	4 p15	protocadherin 7 [Source:HGNC Symbol;Acc:HGNC:8659]	
MRI1	0,46	9,12	0	7 x 50	19 p13	methylthioribose-1-phosphate isomerase 1 [Source:HGNC Symbol;Acc:HGNC:28469]	
BAD	0,46	9,11	0	1 x 44	11 q13	BCL2 associated agonist of cell death [Source:HGNC Symbol;Acc:HGNC:936]	
PTCHD4	0,46	9,08	0	1 x 50	6 p12	patched domain containing 4 [Source:HGNC Symbol;Acc:HGNC:21345]	
ITM2B	0,46	9,07	0	1 x 48	13 q14	integral membrane protein 2B [Source:HGNC Symbol;Acc:HGNC:6174]	
CYP27A1	0,46	9,04	0	1 x 49	2 q35	cytochrome P450 family 27 subfamily A member 1 [Source:HGNC Symbol;Acc:HGNC:2605]	
AKAP12	0,46	9,04	0	1 x 50	6 q25	A-kinase anchoring protein 12 [Source:HGNC Symbol;Acc:HGNC:370]	
ZNF287	0,46	9,02	0	2 x 49	17 p11	zinc finger protein 287 [Source:HGNC Symbol;Acc:HGNC:13502]	


H2AFJ	0,46	8,99	0	3 x 50	12 p12	H2A histone family member J [Source:HGNC Symbol;Acc:HGNC:14456]	
GSTM3	0,46	8,99	0	1 x 50	1 p13	glutathione S-transferase mu 3 (brain) [Source:HGNC Symbol;Acc:HGNC:4635]	
LRRC8A	0,46	8,98	0	1 x 50	9 q34	leucine rich repeat containing 8 family member A [Source:HGNC Symbol;Acc:HGNC:19027]	
FAM65B	0,46	8,95	0	5 x 50	6 p22	family with sequence similarity 65 member B [Source:HGNC Symbol;Acc:HGNC:13872]	
TMEM205	0,46	8,95	0	1 x 48	19 p13	transmembrane protein 205 [Source:HGNC Symbol;Acc:HGNC:29631]	
ITGA1	0,45	8,90	0	1 x 50	5 q11	integrin subunit alpha 1 [Source:HGNC Symbol;Acc:HGNC:6134]	
BIVM	0,45	8,89	0	5 x 50	13 q33	basic, immunoglobulin-like variable motif containing [Source:HGNC Symbol;Acc:HGNC:16034]	
CALM2	0,45	8,88	0	1 x 50	19 q13	calmodulin 3 (phosphorylase kinase, delta) [Source:HGNC Symbol;Acc:HGNC:1449]	
SERPINI1	0,45	8,87	0	1 x 49	3 q26	serpin family I member 1 [Source:HGNC Symbol;Acc:HGNC:8943]	
TRPM4	0,45	8,83	0	2 x 49	19 q13	transient receptor potential cation channel subfamily M member 4 [Source:HGNC Symbol;Acc:HGNC:17993]	
EI24	0,45	8,76	0	1 x 47	11 q24	EI24, autophagy associated transmembrane protein [Source:HGNC Symbol;Acc:HGNC:13276]	
ARHGAP1	0,45	8,72	1,11E-16	2 x 50	11 p11	Rho GTPase activating protein 1 [Source:HGNC Symbol;Acc:HGNC:673]	
DNPH1	0,45	8,70	1,11E-16	1 x 45	6 p21	2'-deoxynucleoside 5'-phosphate N-hydrolase 1 [Source:HGNC Symbol;Acc:HGNC:21218]	
SLC12A2	0,45	8,70	1,11E-16	3 x 50	5 q23	solute carrier family 12 member 2 [Source:HGNC Symbol;Acc:HGNC:10911]	
SERPINB6	0,44	8,67	1,11E-16	1 x 50	6 p25	serpin family B member 6 [Source:HGNC Symbol;Acc:HGNC:8950]	
TIMM8B	0,44	8,67	1,11E-16	1 x 49	11 q23	translocase of inner mitochondrial membrane 8 homolog B (yeast) [Source:HGNC Symbol;Acc:HGNC:11818]	
GOLM1	0,44	8,66	1,11E-16	1 x 48	9 q21	golgi membrane protein 1 [Source:HGNC Symbol;Acc:HGNC:15451]	
NDUFS3	0,44	8,65	2,22E-16	1 x 46	11 p11	NADH:ubiquinone oxidoreductase core subunit S3 [Source:HGNC Symbol;Acc:HGNC:7710]	
	0,44	8,64	2,22E-16	2 x 50			
TBCB	0,44	8,64	2,22E-16	1 x 46	19 q13	tubulin folding cofactor B [Source:HGNC Symbol;Acc:HGNC:1989]	
SEPW1	0,44	8,62	2,22E-16	2 x 50	19 q13	selenoprotein W, 1 [Source:HGNC Symbol;Acc:HGNC:10752]	
TUBA1A	0,44	8,61	2,22E-16	1 x 45	12 q13	tubulin alpha 1a [Source:HGNC Symbol;Acc:HGNC:20766]	
VCAN	0,44	8,59	2,22E-16	2 x 50	5 q14	versican [Source:HGNC Symbol;Acc:HGNC:2464]	
ICAM1	0,44	8,56	2,22E-16	2 x 48	19 p13	intercellular adhesion molecule 1 [Source:HGNC Symbol;Acc:HGNC:5344]	
FDFT1	0,44	8,51	4,44E-16	1 x 45	8 p23	farnesyl-diphosphate farnesyltransferase 1 [Source:HGNC Symbol;Acc:HGNC:3629]	
SURF4	0,44	8,50	4,44E-16	1 x 45	CHR_HG2030_	surfeit 4 [Source:HGNC Symbol;Acc:HGNC:11476]	
PTRH1	0,43	8,43	6,66E-16	1 x 47	9 q34	peptidyl-tRNA hydrolase 1 homolog [Source:HGNC Symbol;Acc:HGNC:27039]	
ZNF585A	0,43	8,43	6,66E-16	4 x 50	19 q13	zinc finger protein 585A [Source:HGNC Symbol;Acc:HGNC:26305]	
PTPMT1	0,43	8,43	6,66E-16	1 x 46	11 p11	protein tyrosine phosphatase, mitochondrial 1 [Source:HGNC Symbol;Acc:HGNC:26965]	
EPHA3	0,43	8,42	6,66E-16	6 x 48	3 p11	EPH receptor A3 [Source:HGNC Symbol;Acc:HGNC:3387]	
TMEM160	0,43	8,39	8,88E-16	2 x 47	19 q13	transmembrane protein 160 [Source:HGNC Symbol;Acc:HGNC:26042]	
TMEM218	0,43	8,39	8,88E-16	1 x 48	11 q24	transmembrane protein 218 [Source:HGNC Symbol;Acc:HGNC:27344]	
NAA10	0,43	8,39	8,88E-16	2 x 49	X q28	N(alpha)-acetyltransferase 10, NatA catalytic subunit [Source:HGNC Symbol;Acc:HGNC:18704]	


ENTPD4	0,43	8,38	9,99E-16	2 x 49	8 p21	ectonucleoside triphosphate diphosphohydrolase 4 [Source:HGNC Symbol;Acc:HGNC:14573]	
RBM42	0,43	8,30	1,78E-15	2 x 48	19 q13	RNA binding motif protein 42 [Source:HGNC Symbol;Acc:HGNC:28117]	
C11orf71	0,43	8,29	1,89E-15	1 x 50	11 q23	chromosome 11 open reading frame 71 [Source:HGNC Symbol;Acc:HGNC:25937]	
HEXB	0,43	8,28	2,00E-15	1 x 46	5 q13	hexosaminidase subunit beta [Source:HGNC Symbol;Acc:HGNC:4879]	
PLD3	0,43	8,27	2,11E-15	1 x 45	19 q13	phospholipase D family member 3 [Source:HGNC Symbol;Acc:HGNC:17158]	
GPC3	0,43	8,24	2,66E-15	5 x 50	X q26	glypican 3 [Source:HGNC Symbol;Acc:HGNC:4451]	
SYAP1	0,42	8,16	4,44E-15	4 x 50	X p22	synapse associated protein 1 [Source:HGNC Symbol;Acc:HGNC:16273]	
ZNF331	0,42	8,15	4,77E-15	6 x 50	19 q13	zinc finger protein 331 [Source:HGNC Symbol;Acc:HGNC:15489]	
DNASE2	0,42	8,13	5,55E-15	3 x 50	19 p13	deoxyribonuclease II, lysosomal [Source:HGNC Symbol;Acc:HGNC:2960]	
PAFAH1B2	0,42	8,12	5,77E-15	6 x 50	11 q23	platelet activating factor acetylhydrolase 1b catalytic subunit 2 [Source:HGNC Symbol;Acc:HGNC:8575]	
NOL7	0,42	8,11	6,22E-15	1 x 44	6 p23	nucleolar protein 7 [Source:HGNC Symbol;Acc:HGNC:21040]	
STOM	0,42	8,04	1,02E-14	1 x 48	9 q33	stomatin [Source:HGNC Symbol;Acc:HGNC:3383]	
EIF4EBP3	0,42	7,99	1,40E-14	5 x 47	5 q31	eukaryotic translation initiation factor 4E binding protein 3 [Source:HGNC Symbol;Acc:HGNC:3290]	
CCDC50	0,41	7,94	1,90E-14	3 x 50	3 q28	coiled-coil domain containing 50 [Source:HGNC Symbol;Acc:HGNC:18111]	
UQCRFS1	0,41	7,93	2,04E-14	1 x 45	19 q12	ubiquinol-cytochrome c reductase, Rieske iron-sulfur polypeptide 1 [Source:HGNC Symbol;Acc:HGNC:12587]	
SCAND1	0,41	7,91	2,38E-14	1 x 45	20 q11	SCAN domain containing 1 [Source:HGNC Symbol;Acc:HGNC:10566]	
CD99	0,41	7,89	2,80E-14	1 x 44	X p22	CD99 molecule [Source:HGNC Symbol;Acc:HGNC:7082]	
F2R	0,41	7,87	3,03E-14	1 x 50	5 q13	coagulation factor II thrombin receptor [Source:HGNC Symbol;Acc:HGNC:3537]	
TM2D2	0,41	7,84	3,71E-14	1 x 47	8 p11	TM2 domain containing 2 [Source:HGNC Symbol;Acc:HGNC:24127]	
FAM127A	0,41	7,81	4,73E-14	1 x 45	X q26	family with sequence similarity 127 member A [Source:HGNC Symbol;Acc:HGNC:2569]	
NDUFS5	0,41	7,77	5,88E-14	5 x 49	1 p34	NADH:ubiquinone oxidoreductase subunit S5 [Source:HGNC Symbol;Acc:HGNC:7712]	
ALKBH3	0,41	7,77	6,01E-14	1 x 50	11 p11	alkB homolog 3, alpha-ketoglutaratedependent dioxygenase [Source:HGNC Symbol;Acc:HGNC:30141]	
ALG9	0,41	7,76	6,27E-14	4 x 50	11 q23	ALG9, alpha-1,2-mannosyltransferase [Source:HGNC Symbol;Acc:HGNC:15672]	
HOOK3	0,41	7,75	6,91E-14	3 x 50	8 p11	hook microtubule-tethering protein 3 [Source:HGNC Symbol;Acc:HGNC:23576]	
ENG	0,40	7,71	8,92E-14	1 x 47	9 q34	endoglin [Source:HGNC Symbol;Acc:HGNC:3349]	
ERICH1	0,40	7,68	1,11E-13	2 x 50	CHR_HSCHR8_	glutamate rich 1 [Source:HGNC Symbol;Acc:HGNC:27234]	
EED	0,40	7,66	1,21E-13	2 x 50	11 q14	embryonic ectoderm development [Source:HGNC Symbol;Acc:HGNC:3188]	
PRAF2	0,40	7,65	1,33E-13	1 x 46	X p11	PRA1 domain family member 2 [Source:HGNC Symbol;Acc:HGNC:28911]	
AMPH	0,40	7,63	1,48E-13	4 x 50	7 p14	amphiphysin [Source:HGNC Symbol;Acc:HGNC:471]	
STRIP2	0,40	7,60	1,83E-13	5 x 50	7 q32	striatin interacting protein 2 [Source:HGNC Symbol;Acc:HGNC:22209]	
MYBBP1A	0,40	7,57	2,24E-13	4 x 50	17 p13	MYB binding protein 1a [Source:HGNC Symbol;Acc:HGNC:7546]	
SARAF	0,40	7,55	2,46E-13	1 x 47	8 p12	store-operated calcium entry associated regulatory factor [Source:HGNC Symbol;Acc:HGNC:28789]	
TMEM50A	0,40	7,55	2,55E-13	3 x 47	1 p36	transmembrane protein 50A [Source:HGNC Symbol;Acc:HGNC:30590]	


MAP3K1	0,40	7,54	2,68E-13	3 x 50	5 q11	mitogen-activated protein kinase kinase kinase 1 [Source:HGNC Symbol;Acc:HGNC:6848]	
MVB12A	0,40	7,52	2,99E-13	1 x 45	19 p13	multivesicular body subunit 12A [Source:HGNC Symbol;Acc:HGNC:25153]	
TMEM80	0,39	7,51	3,35E-13	1 x 48	11 p15	transmembrane protein 80 [Source:HGNC Symbol;Acc:HGNC:27453]	
SYNJ2	0,39	7,46	4,49E-13	5 x 49	6 q25	synaptojanin 2 [Source:HGNC Symbol;Acc:HGNC:11504]	
LRRC16A	0,39	7,44	5,09E-13	2 x 50	6 p22	leucine rich repeat containing 16A [Source:HGNC Symbol;Acc:HGNC:21581]	
JAM3	0,39	7,43	5,56E-13	4 x 47	11 q25	junctional adhesion molecule 3 [Source:HGNC Symbol;Acc:HGNC:15532]	
PEX16	0,39	7,43	5,57E-13	3 x 49	11 p11	peroxisomal biogenesis factor 16 [Source:HGNC Symbol;Acc:HGNC:8857]	
YIF1A	0,39	7,39	7,29E-13	1 x 44	11 q13	Yip1 interacting factor homolog A, membrane trafficking protein [Source:HGNC Symbol;Acc:HGNC:16688]	
PDK2	0,39	7,36	8,82E-13	5 x 49	17 q21	pyruvate dehydrogenase kinase 2 [Source:HGNC Symbol;Acc:HGNC:8810]	
ASB9	0,39	7,34	9,90E-13	5 x 50	X p22	ankyrin repeat and SOCS box containing 9 [Source:HGNC Symbol;Acc:HGNC:17184]	
ROBO1	0,39	7,34	1,00E-12	4 x 50	3 p12	roundabout guidance receptor 1 [Source:HGNC Symbol;Acc:HGNC:10249]	
LY6E	0,39	7,33	1,07E-12	4 x 50	8 q24	lymphocyte antigen 6 complex, locus E [Source:HGNC Symbol;Acc:HGNC:6727]	
TOR1A	0,39	7,32	1,11E-12	1 x 47	9 q34	torsin family 1 member A [Source:HGNC Symbol;Acc:HGNC:3098]	
ALG3	0,39	7,31	1,17E-12	1 x 44	3 q27	ALG3, alpha-1,3- mannosyltransferase [Source:HGNC Symbol;Acc:HGNC:23056]	
SLC39A13	0,39	7,30	1,23E-12	4 x 50	11 p11	solute carrier family 39 member 13 [Source:HGNC Symbol;Acc:HGNC:20859]	
L3MBTL3	0,38	7,27	1,50E-12	5 x 50	6 q23	l(3)mbt-like 3 (Drosophila) [Source:HGNC Symbol;Acc:HGNC:23035]	
EDA2R	0,38	7,26	1,57E-12	5 x 50	X q12	ectodysplasin A2 receptor [Source:HGNC Symbol;Acc:HGNC:17756]	
POLR2E	0,38	7,25	1,73E-12	1 x 44	19 p13	polymerase (RNA) II subunit E [Source:HGNC Symbol;Acc:HGNC:9192]	
TMEM59	0,38	7,25	1,78E-12	1 x 45	1 p32	transmembrane protein 59 [Source:HGNC Symbol;Acc:HGNC:1239]	
SPP1	0,38	7,23	2,01E-12	1 x 49	4 q22	secreted phosphoprotein 1 [Source:HGNC Symbol;Acc:HGNC:11255]	
SSSCA1	0,38	7,23	2,01E-12	1 x 47	11 q13	Sjogren syndrome/scleroderma autoantigen 1 [Source:HGNC Symbol;Acc:HGNC:11328]	
EFNA5	0,38	7,19	2,46E-12	2 x 50	5 q21	ephrin A5 [Source:HGNC Symbol;Acc:HGNC:3225]	
HMGN5	0,38	7,19	2,50E-12	1 x 50	X q21	high mobility group nucleosome binding domain 5 [Source:HGNC Symbol;Acc:HGNC:8013]	
SCAMP3	0,38	7,17	2,80E-12	1 x 45	1 q22	secretory carrier membrane protein 3 [Source:HGNC Symbol;Acc:HGNC:10565]	
MED19	0,38	7,16	2,99E-12	2 x 49	11 q12	mediator complex subunit 19 [Source:HGNC Symbol;Acc:HGNC:29600]	
CNST	0,38	7,16	3,05E-12	3 x 50	1 q44	consortin, connexin sorting protein [Source:HGNC Symbol;Acc:HGNC:26486]	
ATP6V1F	0,38	7,14	3,41E-12	2 x 45	7 q32	ATPase H+ transporting V1 subunit F [Source:HGNC Symbol;Acc:HGNC:16832]	
ZNF28	0,38	7,12	3,80E-12	2 x 49	19 q13	zinc finger protein 28 [Source:HGNC Symbol;Acc:HGNC:13073]	
CNP	0,38	7,12	4,01E-12	6 x 48	17 q21	2',3'-cyclic nucleotide 3' phosphodiesterase [Source:HGNC Symbol;Acc:HGNC:2158]	
EIF4EBP1	0,38	7,11	4,26E-12	1 x 44	8 p11	eukaryotic translation initiation factor 4E binding protein 1 [Source:HGNC Symbol;Acc:HGNC:3288]	
FARSA	0,38	7,09	4,58E-12	1 x 45	19 p13	phenylalanyl-tRNA synthetase alpha subunit [Source:HGNC Symbol;Acc:HGNC:3592]	
	0,38	7,09	4,73E-12	1 x 45			
CARD8	0,37	7,01	7,71E-12	3 x 50	19 q13	caspase recruitment domain family member 8 [Source:HGNC Symbol;Acc:HGNC:17057]	


DBNL	0,37	7,01	7,77E-12	3 x 50	7 p13	drebrin like [Source:HGNC Symbol;Acc:HGNC:2696]	
PPP2R2B	0,37	7,01	7,86E-12	6 x 48	5 q32	protein phosphatase 2 regulatory subunit Bbeta [Source:HGNC Symbol;Acc:HGNC:9305]	
LMAN2	0,37	6,99	8,49E-12	1 x 44	5 q35	lectin, mannose binding 2 [Source:HGNC Symbol;Acc:HGNC:16986]	
SDCBP	0,37	6,96	1,02E-11	1 x 45	8 q12	syndecan binding protein [Source:HGNC Symbol;Acc:HGNC:10662]	
OVCA2	0,37	6,95	1,12E-11	1 x 46	17 p13	ovarian tumor suppressor candidate 2 [Source:HGNC Symbol;Acc:HGNC:24203]	
SQSTM1	0,37	6,94	1,16E-11	1 x 44	5 q35	sequestosome 1 [Source:HGNC Symbol;Acc:HGNC:11280]	
PRICKLE2	0,37	6,93	1,22E-11	1 x 50	3 p14	prickle planar cell polarity protein 2 [Source:HGNC Symbol;Acc:HGNC:20340]	
ATP6AP1	0,37	6,93	1,29E-11	1 x 45	X q28	ATPase H+ transporting accessory protein 1 [Source:HGNC Symbol;Acc:HGNC:868]	
IQGAP2	0,37	6,90	1,51E-11	1 x 48	5 q13	IQ motif containing GTPase activating protein 2 [Source:HGNC Symbol;Acc:HGNC:6111]	
DPAGT1	0,37	6,89	1,60E-11	1 x 48	11 q23	dolichyl-phosphate N-acetylglucosaminephosphotransferase 1 [Source:HGNC Symbol;Acc:HGNC:2995]	
TBRG1	0,37	6,89	1,64E-11	3 x 50	11 q24	transforming growth factor beta regulator 1 [Source:HGNC Symbol;Acc:HGNC:29551]	
FRMD4A	0,37	6,86	1,88E-11	5 x 50	10 p13	FERM domain containing 4A [Source:HGNC Symbol;Acc:HGNC:25491]	
SMIM19	0,37	6,86	1,88E-11	1 x 49	8 p11	small integral membrane protein 19 [Source:HGNC Symbol;Acc:HGNC:25166]	
MICU2	0,37	6,86	1,95E-11	1 x 47	13 q12	mitochondrial calcium uptake 2 [Source:HGNC Symbol;Acc:HGNC:31830]	
ST8SIA1	0,36	6,83	2,29E-11	6 x 50	12 p12	ST8 alpha-N-acetyl-neuraminide alpha-2,8-sialyltransferase 1 [Source:HGNC Symbol;Acc:HGNC:10869]	
C11orf54	0,36	6,83	2,34E-11	4 x 50	11 q21	chromosome 11 open reading frame 54 [Source:HGNC Symbol;Acc:HGNC:30204]	
ZNF232	0,36	6,82	2,46E-11	5 x 50	17 p13	zinc finger protein 232 [Source:HGNC Symbol;Acc:HGNC:13026]	
	0,36	6,82	2,52E-11	1 x 48			
HTATIP2	0,36	6,80	2,78E-11	5 x 50	11 p15	HIV-1 Tat interactive protein 2 [Source:HGNC Symbol;Acc:HGNC:16637]	
FBXO3	0,36	6,80	2,80E-11	3 x 50	11 p13	F-box protein 3 [Source:HGNC Symbol;Acc:HGNC:13582]	
RBPMS	0,36	6,80	2,82E-11	5 x 47	8 p12	RNA binding protein with multiple splicing [Source:HGNC Symbol;Acc:HGNC:19097]	
CCDC28A	0,36	6,75	3,62E-11	3 x 50	6 q24	coiled-coil domain containing 28A [Source:HGNC Symbol;Acc:HGNC:21098]	
MRPS12	0,36	6,75	3,63E-11	1 x 44	CHR_HG26_P	mitochondrial ribosomal protein S12 [Source:HGNC Symbol;Acc:HGNC:10380]	
C19orf24	0,36	6,74	3,88E-11	3 x 47	19 p13	chromosome 19 open reading frame 24 [Source:HGNC Symbol;Acc:HGNC:26073]	
MRPS26	0,36	6,74	4,02E-11	1 x 44	20 p13	mitochondrial ribosomal protein S26 [Source:HGNC Symbol;Acc:HGNC:14045]	
POLB	0,36	6,73	4,11E-11	1 x 49	8 p11	polymerase (DNA) beta [Source:HGNC Symbol;Acc:HGNC:9174]	
BRD3	0,36	6,73	4,11E-11	1 x 48	9 q34	bromodomain containing 3 [Source:HGNC Symbol;Acc:HGNC:1104]	
ENPP1	0,36	6,71	4,67E-11	1 x 50	6 q23	ectonucleotide pyrophosphatase/phosphodiesterase 1 [Source:HGNC Symbol;Acc:HGNC:3356]	
EBF1	0,36	6,68	5,63E-11	5 x 48	5 q33	early B-cell factor 1 [Source:HGNC Symbol;Acc:HGNC:3126]	
C9orf16	0,36	6,68	5,68E-11	1 x 46	9 q34	chromosome 9 open reading frame 16 [Source:HGNC Symbol;Acc:HGNC:17823]	
MEF2C	0,36	6,67	5,89E-11	1 x 49	5 q14	myocyte enhancer factor 2C [Source:HGNC Symbol;Acc:HGNC:6996]	
RAB11B	0,36	6,64	6,97E-11	2 x 47	19 p13	RAB11B, member RAS oncogene family [Source:HGNC Symbol;Acc:HGNC:9761]	
LDLRAD3	0,36	6,64	7,20E-11	4 x 46	11 p13	low density lipoprotein receptor class A domain containing 3 [Source:HGNC Symbol;Acc:HGNC:27046]	


SRA1	0,35	6,62	7,88E-11	1 x 45	5 q31	steroid receptor RNA activator 1 [Source:HGNC Symbol;Acc:HGNC:11281]	
STX8	0,35	6,61	8,41E-11	1 x 50	17 p13	syntaxin 8 [Source:HGNC Symbol;Acc:HGNC:11443]	
PGM2L1	0,35	6,60	8,86E-11	6 x 50	11 q13	phosphoglucomutase 2-like 1 [Source:HGNC Symbol;Acc:HGNC:20898]	
TMX4	0,35	6,60	8,94E-11	5 x 50	20 p12	thioredoxin related transmembrane protein 4 [Source:HGNC Symbol;Acc:HGNC:25237]	
RABAC1	0,35	6,60	9,09E-11	1 x 44	19 q13	Rab acceptor 1 [Source:HGNC Symbol;Acc:HGNC:9794]	
SIGMAR1	0,35	6,59	9,44E-11	1 x 46	9 p13	sigma non-opioid intracellular receptor 1 [Source:HGNC Symbol;Acc:HGNC:8157]	
WDR18	0,35	6,57	1,10E-10	1 x 44	19 p13	WD repeat domain 18 [Source:HGNC Symbol;Acc:HGNC:17956]	
FLYWCH2	0,35	6,56	1,16E-10	1 x 44	16 p13	FLYWCH family member 2 [Source:HGNC Symbol;Acc:HGNC:25178]	
SLC39A3	0,35	6,56	1,16E-10	4 x 46	19 p13	solute carrier family 39 member 3 [Source:HGNC Symbol;Acc:HGNC:17128]	
HSPBP1	0,35	6,56	1,18E-10	1 x 46	19 q13	HSPA (heat shock 70kDa) binding protein, cytoplasmic cochaperone 1 [Source:HGNC Symbol;Acc:HGNC:24989]	
PHACTR2	0,35	6,54	1,26E-10	3 x 50	6 q24	phosphatase and actin regulator 2 [Source:HGNC Symbol;Acc:HGNC:20956]	
LEPROTL1	0,35	6,54	1,27E-10	3 x 50	8 p12	leptin receptor overlapping transcript-like 1 [Source:HGNC Symbol;Acc:HGNC:6555]	
CD55	0,35	6,54	1,29E-10	3 x 50	1 q32	CD55 molecule (Cromer blood group) [Source:HGNC Symbol;Acc:HGNC:2665]	
HSPB1	0,35	6,53	1,34E-10	1 x 45	7 q11	heat shock protein family B (small) member 1 [Source:HGNC Symbol;Acc:HGNC:5246]	
CEP164	0,35	6,52	1,43E-10	4 x 50	11 q23	centrosomal protein 164 [Source:HGNC Symbol;Acc:HGNC:29182]	
VAMP2	0,35	6,52	1,46E-10	7 x 50	17 p13	vesicle associated membrane protein 2 [Source:HGNC Symbol;Acc:HGNC:12643]	
ZNF490	0,35	6,52	1,49E-10	7 x 50	19 p13	zinc finger protein 490 [Source:HGNC Symbol;Acc:HGNC:23705]	
ZFP36L1	0,35	6,50	1,59E-10	4 x 50	14 q24	ZFP36 ring finger protein-like 1 [Source:HGNC Symbol;Acc:HGNC:1107]	
SDF2L1	0,35	6,50	1,66E-10	2 x 47	22 q11	stromal cell derived factor 2 like 1 [Source:HGNC Symbol;Acc:HGNC:10676]	
FKBP2	0,35	6,49	1,69E-10	1 x 44	11 q13	FK506 binding protein 2 [Source:HGNC Symbol;Acc:HGNC:3718]	
XPA	0,35	6,48	1,86E-10	3 x 47	9 q22	XPA, DNA damage recognition and repair factor [Source:HGNC Symbol;Acc:HGNC:12814]	
GLTSCR2	0,35	6,47	1,91E-10	1 x 46	19 q13	glioma tumor suppressor candidate region gene 2 [Source:HGNC Symbol;Acc:HGNC:4333]	
EXOSC4	0,35	6,47	1,93E-10	1 x 46	8 q24	exosome component 4 [Source:HGNC Symbol;Acc:HGNC:18189]	
KRT10	0,35	6,47	1,99E-10	7 x 50	17 q21	keratin 10 [Source:HGNC Symbol;Acc:HGNC:6413]	
TLE4	0,35	6,46	2,03E-10	5 x 50	9 q21	transducin like enhancer of split 4 [Source:HGNC Symbol;Acc:HGNC:11840]	
LHFPL2	0,35	6,46	2,09E-10	4 x 50	5 q14	lipoma HMGIC fusion partner-like 2 [Source:HGNC Symbol;Acc:HGNC:6588]	
FBXO25	0,35	6,44	2,33E-10	1 x 47	8 p23	F-box protein 25 [Source:HGNC Symbol;Acc:HGNC:13596]	
DCPS	0,35	6,42	2,54E-10	2 x 50	11 q24	decapping enzyme, scavenger [Source:HGNC Symbol;Acc:HGNC:29812]	
TMEM126B	0,34	6,40	2,99E-10	2 x 50	11 q14	transmembrane protein 126B [Source:HGNC Symbol;Acc:HGNC:30883]	
RAB34	0,34	6,39	3,18E-10	1 x 45	17 q11	RAB34, member RAS oncogene family [Source:HGNC Symbol;Acc:HGNC:16519]	
SASH1	0,34	6,38	3,31E-10	6 x 50	6 q24	SAM and SH3 domain containing 1 [Source:HGNC Symbol;Acc:HGNC:19182]	
ALG2	0,34	6,36	3,71E-10	1 x 48	9 q22	ALG2, alpha-1,3/1,6-mannosyltransferase [Source:HGNC Symbol;Acc:HGNC:23159]	
CNOT8	0,34	6,36	3,72E-10	1 x 46	5 q33	CCR4-NOT transcription complex subunit 8 [Source:HGNC Symbol;Acc:HGNC:9207]	


AP3M2	0,34	6,35	3,92E-10	2 x 50	8 p11	adaptor related protein complex 3 mu 2 subunit [Source:HGNC Symbol;Acc:HGNC:570]	
CORO1B	0,34	6,33	4,32E-10	1 x 44	11 q13	coronin 1B [Source:HGNC Symbol;Acc:HGNC:2253]	
ALKBH7	0,34	6,31	4,78E-10	1 x 44	19 p13	alkB homolog 7 [Source:HGNC Symbol;Acc:HGNC:21306]	
KIF3A	0,34	6,30	5,07E-10	1 x 46	5 q31	kinesin family member 3A [Source:HGNC Symbol;Acc:HGNC:6319]	
NRP2	0,34	6,29	5,58E-10	3 x 49	2 q33	neuropilin 2 [Source:HGNC Symbol;Acc:HGNC:8005]	
ZMAT2	0,34	6,26	6,46E-10	3 x 50	5 q31	zinc finger matrin-type 2 [Source:HGNC Symbol;Acc:HGNC:26433]	
VPS37A	0,34	6,26	6,59E-10	5 x 50	8 p22	VPS37A, ESCRT-I subunit [Source:HGNC Symbol;Acc:HGNC:24928]	
CWC15	0,34	6,25	6,78E-10	4 x 50	11 q21	CWC15 spliceosome-associated protein [Source:HGNC Symbol;Acc:HGNC:26939]	
MTUS1	0,34	6,24	7,20E-10	6 x 49	8 p22	microtubule associated tumor suppressor 1 [Source:HGNC Symbol;Acc:HGNC:29789]	
	0,34	6,22	8,23E-10	3 x 50			
KMT2A	0,33	6,20	9,14E-10	5 x 50	11 q23	lysine methyltransferase 2A [Source:HGNC Symbol;Acc:HGNC:7132]	
TXNDC15	0,33	6,20	9,37E-10	3 x 48	5 q31	thioredoxin domain containing 15 [Source:HGNC Symbol;Acc:HGNC:20652]	
HYKK	0,33	6,19	9,77E-10	4 x 50	15 q25	hydroxylysine kinase [Source:HGNC Symbol;Acc:HGNC:34403]	
CHMP2A	0,33	6,19	9,88E-10	1 x 49	19 q13	charged multivesicular body protein 2A [Source:HGNC Symbol;Acc:HGNC:30216]	
MYD88	0,33	6,17	1,10E-09	5 x 49	3 p22	myeloid differentiation primary response 88 [Source:HGNC Symbol;Acc:HGNC:7562]	
NDUFA2	0,33	6,16	1,12E-09	3 x 44	5 q31	NADH:ubiquinone oxidoreductase subunit A2 [Source:HGNC Symbol;Acc:HGNC:7685]	
BNIP3L	0,33	6,16	1,12E-09	2 x 50	8 p21	BCL2/adenovirus E1B 19kDa interacting protein 3-like [Source:HGNC Symbol;Acc:HGNC:1085]	
MTCH2	0,33	6,16	1,16E-09	1 x 45	11 p11	mitochondrial carrier 2 [Source:HGNC Symbol;Acc:HGNC:17587]	
HSPA4	0,33	6,15	1,21E-09	1 x 44	5 q31	heat shock protein family A (Hsp70) member 4 [Source:HGNC Symbol;Acc:HGNC:5237]	
TMEM242	0,33	6,15	1,23E-09	6 x 50	6 q25	transmembrane protein 242 [Source:HGNC Symbol;Acc:HGNC:17206]	
PCM1	0,33	6,15	1,23E-09	4 x 50	8 p22	pericentriolar material 1 [Source:HGNC Symbol;Acc:HGNC:8727]	
	0,33	6,14	1,25E-09	4 x 48			
TMEM70	0,33	6,14	1,26E-09	1 x 45	8 q21	transmembrane protein 70 [Source:HGNC Symbol;Acc:HGNC:26050]	
MCC	0,33	6,13	1,34E-09	1 x 49	5 q22	mutated in colorectal cancers [Source:HGNC Symbol;Acc:HGNC:6935]	
LGALS3BP	0,33	6,12	1,46E-09	1 x 45	17 q25	galectin 3 binding protein [Source:HGNC Symbol;Acc:HGNC:6564]	
DBN1	0,33	6,12	1,47E-09	1 x 46	5 q35	drebrin 1 [Source:HGNC Symbol;Acc:HGNC:2695]	
CLN5	0,33	6,11	1,50E-09	1 x 46	13 q22	ceroid-lipofuscinosis, neuronal 5 [Source:HGNC Symbol;Acc:HGNC:2076]	
GOLGA7	0,33	6,11	1,51E-09	3 x 49	8 p11	golgin A7 [Source:HGNC Symbol;Acc:HGNC:24876]	
FLNA	0,33	6,11	1,55E-09	1 x 44	X q28	filamin A [Source:HGNC Symbol;Acc:HGNC:3754]	
ARMC9	0,33	6,10	1,60E-09	1 x 47	2 q37	armadillo repeat containing 9 [Source:HGNC Symbol;Acc:HGNC:20730]	
PRELID1	0,33	6,09	1,67E-09	1 x 44	5 q35	PRELI domain containing 1 [Source:HGNC Symbol;Acc:HGNC:30255]	
TMEM147	0,33	6,08	1,84E-09	1 x 44	19 q13	transmembrane protein 147 [Source:HGNC Symbol;Acc:HGNC:30414]	
GBP3	0,33	6,06	1,96E-09	6 x 49	1 p22	guanylate binding protein 3 [Source:HGNC Symbol;Acc:HGNC:4184]	


PGLS	0,33	6,05	2,08E-09	1 x 45	19 p13	6-phosphogluconolactonase [Source:HGNC Symbol;Acc:HGNC:8903]	
NUDT16L1	0,33	6,05	2,13E-09	3 x 47	16 p13	nudix hydrolase 16 like 1 [Source:HGNC Symbol;Acc:HGNC:28154]	
CAMLG	0,33	6,04	2,25E-09	1 x 47	5 q31	calcium modulating ligand [Source:HGNC Symbol;Acc:HGNC:1471]	
SSBP1	0,33	6,03	2,40E-09	5 x 47	CHR_HSCHR7_	single stranded DNA binding protein 1 [Source:HGNC Symbol;Acc:HGNC:11317]	
DIMT1	0,33	6,02	2,47E-09	1 x 46	5 q12	DIM1 dimethyladenosine transferase 1 homolog [Source:HGNC Symbol;Acc:HGNC:30217]	
PSMC3	0,33	6,02	2,53E-09	4 x 50	11 p11	proteasome 26S subunit, ATPase 3 [Source:HGNC Symbol;Acc:HGNC:9549]	
TMEM67	0,33	6,02	2,56E-09	4 x 50	8 q22	transmembrane protein 67 [Source:HGNC Symbol;Acc:HGNC:28396]	
THEM4	0,33	6,01	2,68E-09	3 x 50	1 q21	thioesterase superfamily member 4 [Source:HGNC Symbol;Acc:HGNC:17947]	
KBTBD4	0,32	6,00	2,85E-09	5 x 49	11 p11	kelch repeat and BTB domain containing 4 [Source:HGNC Symbol;Acc:HGNC:23761]	
CLTA	0,32	5,99	2,99E-09	1 x 44	9 p13	clathrin light chain A [Source:HGNC Symbol;Acc:HGNC:2090]	
YKT6	0,32	5,98	3,07E-09	5 x 47	7 p13	YKT6 v-SNARE homolog (S. cerevisiae) [Source:HGNC Symbol;Acc:HGNC:16959]	
FUT10	0,32	5,97	3,23E-09	4 x 50	8 p12	fucosyltransferase 10 [Source:HGNC Symbol;Acc:HGNC:19234]	
OTUB1	0,32	5,97	3,24E-09	2 x 44	11 q13	OTU deubiquitinase, ubiquitin aldehyde binding 1 [Source:HGNC Symbol;Acc:HGNC:23077]	
C11orf49	0,32	5,97	3,38E-09	4 x 50	11 p11	chromosome 11 open reading frame 49 [Source:HGNC Symbol;Acc:HGNC:28720]	
ST6GAL1	0,32	5,96	3,50E-09	4 x 50	3 q27	ST6 beta-galactoside alpha-2,6-sialyltransferase 1 [Source:HGNC Symbol;Acc:HGNC:10860]	
PLSCR3	0,32	5,96	3,51E-09	5 x 48	17 p13	TMEM256-PLSCR3 readthrough (NMD candidate) [Source:HGNC Symbol;Acc:HGNC:49186]	
LAGE3	0,32	5,95	3,65E-09	1 x 47	X q28	L antigen family member 3 [Source:HGNC Symbol;Acc:HGNC:26058]	
EGFL7	0,32	5,94	3,95E-09	2 x 45	9 q34	EGF like domain multiple 7 [Source:HGNC Symbol;Acc:HGNC:20594]	
GRWD1	0,32	5,93	4,09E-09	3 x 46	19 q13	glutamate rich WD repeat containing 1 [Source:HGNC Symbol;Acc:HGNC:21270]	
PSMB2	0,32	5,93	4,19E-09	6 x 48	1 p34	proteasome subunit beta 2 [Source:HGNC Symbol;Acc:HGNC:9539]	
TTC14	0,32	5,93	4,19E-09	2 x 50	3 q26	tetratricopeptide repeat domain 14 [Source:HGNC Symbol;Acc:HGNC:24697]	
SSBP2	0,32	5,92	4,23E-09	2 x 50	5 q14	single stranded DNA binding protein 2 [Source:HGNC Symbol;Acc:HGNC:15831]	
SGK3	0,32	5,92	4,36E-09	5 x 50	8 q13	serum/glucocorticoid regulated kinase family member 3 [Source:HGNC Symbol;Acc:HGNC:10812]	
GPR180	0,32	5,91	4,62E-09	7 x 50	13 q32	G protein-coupled receptor 180 [Source:HGNC Symbol;Acc:HGNC:28899]	
OFD1	0,32	5,90	4,71E-09	4 x 50	X p22	OFD1, centriole and centriolar satellite protein [Source:HGNC Symbol;Acc:HGNC:2567]	
POLR3G	0,32	5,90	4,77E-09	5 x 50	5 q14	polymerase (RNA) III subunit G [Source:HGNC Symbol;Acc:HGNC:30075]	
MED18	0,32	5,88	5,46E-09	4 x 50	1 p35	mediator complex subunit 18 [Source:HGNC Symbol;Acc:HGNC:25944]	
FBXL20	0,32	5,87	5,69E-09	5 x 50	17 q12	F-box and leucine rich repeat protein 20 [Source:HGNC Symbol;Acc:HGNC:24679]	
TMEM179B	0,32	5,85	6,19E-09	1 x 46	11 q12	transmembrane protein 179B [Source:HGNC Symbol;Acc:HGNC:33744]	
CREM	0,32	5,84	6,51E-09	3 x 50	10 p11	cAMP responsive element modulator [Source:HGNC Symbol;Acc:HGNC:2352]	
CRYL1	0,32	5,84	6,78E-09	1 x 46	13 q12	crystallin lambda 1 [Source:HGNC Symbol;Acc:HGNC:18246]	
SNAI2	0,32	5,84	6,81E-09	1 x 45	8 q11	snail family transcriptional repressor 2 [Source:HGNC Symbol;Acc:HGNC:11094]	
ZNF814	0,32	5,83	7,10E-09	1 x 46	19 q13	zinc finger protein 814 [Source:HGNC Symbol;Acc:HGNC:33258]	


UBE2D4	0,32	5,83	7,24E-09	3 x 50	7 p13	ubiquitin conjugating enzyme E2 D4 (putative) [Source:HGNC Symbol;Acc:HGNC:21647]	
ECE2	0,32	5,82	7,55E-09	5 x 48	3 q27	endothelin converting enzyme 2 [Source:HGNC Symbol;Acc:HGNC:13275]	
SDHD	0,32	5,81	7,65E-09	3 x 47	11 q23	succinate dehydrogenase complex subunit D [Source:HGNC Symbol;Acc:HGNC:10683]	
C17orf49	0,32	5,81	7,89E-09	3 x 50	17 p13	chromosome 17 open reading frame 49 [Source:HGNC Symbol;Acc:HGNC:28737]	
CPN1	0,32	5,80	8,09E-09	4 x 46	10 q24	carboxypeptidase N subunit 1 [Source:HGNC Symbol;Acc:HGNC:2312]	
MGAT1	0,32	5,80	8,40E-09	2 x 46	5 q35	mannosyl (alpha-1,3-)-glycoprotein beta-1,2-N-acetylglucosaminyltransferase [Source:HGNC Symbol;Acc:HGNC:7044]	
PSMG4	0,31	5,78	9,12E-09	3 x 50	6 p25	proteasome assembly chaperone 4 [Source:HGNC Symbol;Acc:HGNC:21108]	
STX10	0,31	5,78	9,34E-09	1 x 48	19 p13	syntaxin 10 [Source:HGNC Symbol;Acc:HGNC:11428]	
RAD50	0,31	5,78	9,39E-09	4 x 50	5 q31	RAD50 double strand break repair protein [Source:HGNC Symbol;Acc:HGNC:9816]	
NEK6	0,31	5,77	9,88E-09	1 x 45	9 q33	NIMA related kinase 6 [Source:HGNC Symbol;Acc:HGNC:7749]	
GLRX2	0,31	5,76	1,00E-08	2 x 44	1 q31	glutaredoxin 2 [Source:HGNC Symbol;Acc:HGNC:16065]	
PSD3	0,31	5,75	1,10E-08	5 x 47	8 p22	pleckstrin and Sec7 domain containing 3 [Source:HGNC Symbol;Acc:HGNC:19093]	
NOSIP	0,31	5,75	1,10E-08	2 x 46	19 q13	nitric oxide synthase interacting protein [Source:HGNC Symbol;Acc:HGNC:17946]	
MRPL4	0,31	5,74	1,13E-08	1 x 45	19 p13	mitochondrial ribosomal protein L4 [Source:HGNC Symbol;Acc:HGNC:14276]	
LRP12	0,31	5,73	1,22E-08	2 x 50	8 q22	LDL receptor related protein 12 [Source:HGNC Symbol;Acc:HGNC:31708]	
HSDL2	0,31	5,72	1,26E-08	2 x 47	9 q32	hydroxysteroid dehydrogenase like 2 [Source:HGNC Symbol;Acc:HGNC:18572]	
EMP3	0,31	5,72	1,27E-08	5 x 47	19 q13	epithelial membrane protein 3 [Source:HGNC Symbol;Acc:HGNC:3335]	
LPAR6	0,31	5,72	1,30E-08	5 x 47	13 q14	lysophosphatidic acid receptor 6 [Source:HGNC Symbol;Acc:HGNC:15520]	
NDUFAF2	0,31	5,72	1,30E-08	4 x 48	5 q12	NADH:ubiquinone oxidoreductase complex assembly factor 2 [Source:HGNC Symbol;Acc:HGNC:28086]	
F2RL2	0,31	5,71	1,36E-08	6 x 50	5 q13	coagulation factor II thrombin receptor like 2 [Source:HGNC Symbol;Acc:HGNC:3539]	
ARF5	0,31	5,71	1,37E-08	1 x 45	7 q32	ADP ribosylation factor 5 [Source:HGNC Symbol;Acc:HGNC:658]	
C1orf122	0,31	5,69	1,50E-08	7 x 50	1 p34	chromosome 1 open reading frame 122 [Source:HGNC Symbol;Acc:HGNC:24789]	
KBTBD3	0,31	5,68	1,53E-08	6 x 48	11 q22	kelch repeat and BTB domain containing 3 [Source:HGNC Symbol;Acc:HGNC:22934]	
FBXO32	0,31	5,68	1,61E-08	3 x 50	8 q24	F-box protein 32 [Source:HGNC Symbol;Acc:HGNC:16731]	
ATP5D	0,31	5,67	1,65E-08	1 x 47	19 p13	ATP synthase, H+ transporting, mitochondrial F1 complex, delta subunit [Source:HGNC Symbol;Acc:HGNC:837]	
RB1CC1	0,31	5,67	1,66E-08	1 x 47	8 q11	RB1 inducible coiled-coil 1 [Source:HGNC Symbol;Acc:HGNC:15574]	
APC	0,31	5,66	1,76E-08	4 x 50	5 q22	adenomatous polyposis coli [Source:HGNC Symbol;Acc:HGNC:583]	
GSTK1	0,31	5,66	1,79E-08	1 x 48	7 q34	glutathione S-transferase kappa 1 [Source:HGNC Symbol;Acc:HGNC:16906]	
CAPNS1	0,31	5,63	2,00E-08	4 x 48	19 q13	calpain small subunit 1 [Source:HGNC Symbol;Acc:HGNC:1481]	
TANC2	0,31	5,63	2,01E-08	7 x 50	17 q23	tetratricopeptide repeat, ankyrin repeat and coiled-coil containing 2 [Source:HGNC Symbol;Acc:HGNC:30212]	
SNX19	0,31	5,63	2,02E-08	5 x 47	11 q25	sorting nexin 19 [Source:HGNC Symbol;Acc:HGNC:21532]	
NDUFB7	0,31	5,63	2,05E-08	1 x 44	19 p13	NADH:ubiquinone oxidoreductase subunit B7 [Source:HGNC Symbol;Acc:HGNC:7702]	
A1BG	0,31	5,62	2,17E-08	4 x 50	19 q13	alpha-1-B glycoprotein [Source:HGNC Symbol;Acc:HGNC:5]	


ARSK	0,30	5,57	2,74E-08	6 x 50	5 q15	arylsulfatase family member K [Source:HGNC Symbol;Acc:HGNC:25239]	
PCMT1	0,30	5,55	3,05E-08	2 x 45	6 q25	protein-L-isoaspartate (D-aspartate) O-methyltransferase [Source:HGNC Symbol;Acc:HGNC:8728]	
FBL	0,30	5,55	3,06E-08	2 x 48	CHR_HG2021_	fibrillarin [Source:HGNC Symbol;Acc:HGNC:3599]	
CEP41	0,30	5,55	3,15E-08	4 x 50	7 q32	centrosomal protein 41 [Source:HGNC Symbol;Acc:HGNC:12370]	
DPM2	0,30	5,54	3,22E-08	1 x 45	9 q34	dolichyl-phosphate mannosyltransferase polypeptide 2, regulatory subunit [Source:HGNC Symbol;Acc:HGNC:3006]	
C6orf1	0,30	5,54	3,28E-08	1 x 44	6 p21	chromosome 6 open reading frame 1 [Source:HGNC Symbol;Acc:HGNC:1340]	
BHLHE40	0,30	5,53	3,40E-08	1 x 46	3 p26	basic helix-loop-helix family member e40 [Source:HGNC Symbol;Acc:HGNC:1046]	
FUCA1	0,30	5,52	3,63E-08	4 x 49	1 p36	fucosidase, alpha-L- 1, tissue [Source:HGNC Symbol;Acc:HGNC:4006]	
C19orf70	0,30	5,50	4,03E-08	1 x 45	19 p13	chromosome 19 open reading frame 70 [Source:HGNC Symbol;Acc:HGNC:33702]	
NR1H2	0,30	5,50	4,05E-08	1 x 44	19 q13	nuclear receptor subfamily 1 group H member 2 [Source:HGNC Symbol;Acc:HGNC:7965]	
DGCR6L	0,30	5,50	4,06E-08	4 x 48	22 q11	DiGeorge syndrome critical region gene 6-like [Source:HGNC Symbol;Acc:HGNC:18551]	
KLHL7	0,30	5,49	4,20E-08	1 x 48	7 p15	kelch like family member 7 [Source:HGNC Symbol;Acc:HGNC:15646]	
NAA35	0,30	5,48	4,37E-08	4 x 50	9 q21	N(alpha)-acetyltransferase 35, NatC auxiliary subunit [Source:HGNC Symbol;Acc:HGNC:24340]	
CXorf56	0,30	5,47	4,59E-08	5 x 48	X q24	chromosome X open reading frame 56 [Source:HGNC Symbol;Acc:HGNC:26239]	
CBX3	0,30	5,46	4,97E-08	7 x 50	7 p15	chromobox 3 [Source:HGNC Symbol;Acc:HGNC:1553]	
FAM91A1	0,30	5,46	5,03E-08	6 x 50	8 q24	family with sequence similarity 91 member A1 [Source:HGNC Symbol;Acc:HGNC:26306]	
CHORDC1	0,30	5,45	5,14E-08	5 x 50	11 q14	cysteine and histidine rich domain containing 1 [Source:HGNC Symbol;Acc:HGNC:14525]	
VDAC3	0,30	5,45	5,23E-08	5 x 47	8 p11	voltage dependent anion channel 3 [Source:HGNC Symbol;Acc:HGNC:12674]	
MLLT3	0,30	5,45	5,28E-08	5 x 50	9 p21	myeloid/lymphoid or mixed-lineage leukemia; translocated to, 3 [Source:HGNC Symbol;Acc:HGNC:7136]	
MRPL16	0,30	5,43	5,90E-08	1 x 45	11 q12	mitochondrial ribosomal protein L16 [Source:HGNC Symbol;Acc:HGNC:14476]	
NDUFS7	0,30	5,42	5,97E-08	1 x 44	19 p13	NADH:ubiquinone oxidoreductase core subunit S7 [Source:HGNC Symbol;Acc:HGNC:7714]	
GDF15	0,30	5,42	6,10E-08	1 x 45	19 p13	growth differentiation factor 15 [Source:HGNC Symbol;Acc:HGNC:30142]	
STX3	0,30	5,39	6,98E-08	1 x 46	11 q12	syntaxin 3 [Source:HGNC Symbol;Acc:HGNC:11438]	
CTBS	0,29	5,39	7,09E-08	6 x 50	1 p22	chitobiase [Source:HGNC Symbol;Acc:HGNC:2496]	
ZNF226	0,29	5,38	7,50E-08	2 x 49	19 q13	zinc finger protein 226 [Source:HGNC Symbol;Acc:HGNC:13019]	
CST3	0,29	5,38	7,59E-08	1 x 44	20 p11	cystatin C [Source:HGNC Symbol;Acc:HGNC:2475]	
SAT2	0,29	5,38	7,60E-08	3 x 48	17 p13	spermidine/spermine N1-acetyltransferase family member 2 [Source:HGNC Symbol;Acc:HGNC:23160]	
MED29	0,29	5,37	7,69E-08	4 x 50	19 q13	mediator complex subunit 29 [Source:HGNC Symbol;Acc:HGNC:23074]	
SYNGR1	0,29	5,36	8,27E-08	1 x 46	22 q13	synaptogyrin 1 [Source:HGNC Symbol;Acc:HGNC:11498]	
EXO5	0,29	5,33	9,73E-08	7 x 50	1 p34	exonuclease 5 [Source:HGNC Symbol;Acc:HGNC:26115]	
WIPI2	0,29	5,32	9,92E-08	4 x 48	7 p22	WD repeat domain, phosphoinositide interacting 2 [Source:HGNC Symbol;Acc:HGNC:32225]	
STAT3	0,29	5,32	1,02E-07	1 x 45	17 q21	signal transducer and activator of transcription 3 [Source:HGNC Symbol;Acc:HGNC:11364]	
AP3B1	0,29	5,32	1,03E-07	5 x 50	5 q14	adaptor related protein complex 3 beta 1 subunit [Source:HGNC Symbol;Acc:HGNC:566]	


ZCCHC6	0,29	5,30	1,10E-07	5 x 50	9 q21	zinc finger CCHC-type containing 6 [Source:HGNC Symbol;Acc:HGNC:25817]	
KRTCAP2	0,29	5,30	1,13E-07	5 x 48	1 q22	keratinocyte associated protein 2 [Source:HGNC Symbol;Acc:HGNC:28942]	
PAX3	0,29	5,29	1,19E-07	3 x 46	2 q36	paired box 3 [Source:HGNC Symbol;Acc:HGNC:8617]	
BTD	0,29	5,28	1,20E-07	5 x 50	3 p25	biotinidase [Source:HGNC Symbol;Acc:HGNC:1122]	
THYN1	0,29	5,28	1,25E-07	4 x 50	11 q25	thymocyte nuclear protein 1 [Source:HGNC Symbol;Acc:HGNC:29560]	
KIF13A	0,29	5,27	1,27E-07	6 x 50	6 p22	kinesin family member 13A [Source:HGNC Symbol;Acc:HGNC:14566]	
GEMIN8	0,29	5,27	1,29E-07	3 x 48	X p22	gem nuclear organelle associated protein 8 [Source:HGNC Symbol;Acc:HGNC:26044]	
SLU7	0,29	5,27	1,29E-07	3 x 47	5 q33	SLU7 homolog, splicing factor [Source:HGNC Symbol;Acc:HGNC:16939]	
UBE2L6	0,29	5,27	1,29E-07	1 x 47	11 q12	ubiquitin conjugating enzyme E2 L6 [Source:HGNC Symbol;Acc:HGNC:12490]	
COMMD5	0,29	5,26	1,38E-07	3 x 46	8 q24	COMM domain containing 5 [Source:HGNC Symbol;Acc:HGNC:17902]	
CCDC90B	0,29	5,25	1,45E-07	4 x 50	11 q14	coiled-coil domain containing 90B [Source:HGNC Symbol;Acc:HGNC:28108]	
ISCU	0,29	5,22	1,64E-07	1 x 46	12 q23	iron-sulfur cluster assembly enzyme [Source:HGNC Symbol;Acc:HGNC:29882]	
FNBP4	0,29	5,22	1,65E-07	5 x 50	11 p11	formin binding protein 4 [Source:HGNC Symbol;Acc:HGNC:19752]	
ZFPL1	0,29	5,22	1,70E-07	1 x 46	11 q13	zinc finger protein like 1 [Source:HGNC Symbol;Acc:HGNC:12868]	
SMARCA1	0,29	5,19	1,88E-07	4 x 50	X q26	SWI/SNF related, matrix associated, actin dependent regulator of chromatin, subfamily a, member 1	
ZNF587	0,29	5,19	1,89E-07	5 x 50	19 q13	zinc finger protein 587 [Source:HGNC Symbol;Acc:HGNC:30955]	
VCP	0,28	5,16	2,18E-07	1 x 46	9 p13	valosin containing protein [Source:HGNC Symbol;Acc:HGNC:12666]	
ZSWIM7	0,28	5,16	2,27E-07	1 x 44	17 p12	zinc finger SWIM-type containing 7 [Source:HGNC Symbol;Acc:HGNC:26993]	
ACOT13	0,28	5,15	2,28E-07	6 x 48	6 p22	acyl-CoA thioesterase 13 [Source:HGNC Symbol;Acc:HGNC:20999]	
SLC36A4	0,28	5,13	2,56E-07	4 x 47	11 q21	solute carrier family 36 member 4 [Source:HGNC Symbol;Acc:HGNC:19660]	
ELF1	0,28	5,12	2,67E-07	5 x 50	13 q14	E74 like ETS transcription factor 1 [Source:HGNC Symbol;Acc:HGNC:3316]	
ST20	0,28	5,12	2,72E-07	1 x 44	15 q25	suppressor of tumorigenicity 20 [Source:HGNC Symbol;Acc:HGNC:33520]	
PLK2	0,28	5,09	3,07E-07	1 x 45	5 q11	polo like kinase 2 [Source:HGNC Symbol;Acc:HGNC:19699]	
TCTA	0,28	5,08	3,25E-07	3 x 47	3 p21	T-cell leukemia translocation altered [Source:HGNC Symbol;Acc:HGNC:11692]	
ZFAND2A	0,28	5,07	3,42E-07	4 x 50	7 p22	zinc finger AN1-type containing 2A [Source:HGNC Symbol;Acc:HGNC:28073]	
ARL6IP5	0,28	5,07	3,53E-07	5 x 48	3 p14	ADP ribosylation factor like GTPase 6 interacting protein 5 [Source:HGNC Symbol;Acc:HGNC:16937]	
MCPH1	0,28	5,06	3,55E-07	7 x 50	8 p23	microcephalin 1 [Source:HGNC Symbol;Acc:HGNC:6954]	
TMEM120A	0,28	5,05	3,74E-07	1 x 44	7 q11	transmembrane protein 120A [Source:HGNC Symbol;Acc:HGNC:21697]	
DDB2	0,28	5,05	3,77E-07	4 x 50	11 p11	damage specific DNA binding protein 2 [Source:HGNC Symbol;Acc:HGNC:2718]	
RNF170	0,28	5,03	4,22E-07	5 x 49	8 p11	ring finger protein 170 [Source:HGNC Symbol;Acc:HGNC:25358]	
VPS13A	0,28	5,02	4,38E-07	1 x 47	9 q21	vacuolar protein sorting 13 homolog A [Source:HGNC Symbol;Acc:HGNC:1908]	
ASH2L	0,28	5,02	4,44E-07	6 x 50	8 p11	ASH2 like histone lysine methyltransferase complex subunit [Source:HGNC Symbol;Acc:HGNC:744]	
AKR1A1	0,28	5,00	4,91E-07	4 x 50	1 p34	aldo-keto reductase family 1 member A1 [Source:HGNC Symbol;Acc:HGNC:380]	


PPP2R1B	0,27	5,00	4,96E-07	5 x 49	11 q23	protein phosphatase 2 scaffold subunit Abeta [Source:HGNC Symbol;Acc:HGNC:9303]	
ABI1	0,27	4,99	5,14E-07	6 x 50	10 p12	abl interactor 1 [Source:HGNC Symbol;Acc:HGNC:11320]	
SLC20A2	0,27	4,98	5,45E-07	6 x 50	8 p11	solute carrier family 20 member 2 [Source:HGNC Symbol;Acc:HGNC:10947]	
VKORC1	0,27	4,97	5,51E-07	1 x 44	16 p11	vitamin K epoxide reductase complex subunit 1 [Source:HGNC Symbol;Acc:HGNC:23663]	
MOCS2	0,27	4,96	5,84E-07	4 x 48	5 q11	molybdenum cofactor synthesis 2 [Source:HGNC Symbol;Acc:HGNC:7193]	
FUCA2	0,27	4,96	5,97E-07	2 x 46	6 q24	fucosidase, alpha-L- 2, plasma [Source:HGNC Symbol;Acc:HGNC:4008]	
POLR2H	0,27	4,95	6,01E-07	1 x 46	3 q27	polymerase (RNA) II subunit H [Source:HGNC Symbol;Acc:HGNC:9195]	
HMBS	0,27	4,94	6,46E-07	1 x 46	CHR_HG2217_	hydroxymethylbilane synthase [Source:HGNC Symbol;Acc:HGNC:4982]	
FAM172A	0,27	4,92	7,01E-07	4 x 50	5 q15	family with sequence similarity 172 member A [Source:HGNC Symbol;Acc:HGNC:25365]	
WBSCR22	0,27	4,92	7,21E-07	1 x 44	7 q11	Williams-Beuren syndrome chromosome region 22 [Source:HGNC Symbol;Acc:HGNC:16405]	
PSMC2	0,27	4,90	7,64E-07	5 x 47	7 q22	proteasome 26S subunit, ATPase 2 [Source:HGNC Symbol;Acc:HGNC:9548]	
DCTN6	0,27	4,89	8,18E-07	5 x 50	8 p12	dynactin subunit 6 [Source:HGNC Symbol;Acc:HGNC:16964]	
DHX29	0,27	4,88	8,56E-07	7 x 50	5 q11	DEAH-box helicase 29 [Source:HGNC Symbol;Acc:HGNC:15815]	
RTN3	0,27	4,84	1,04E-06	1 x 44	11 q13	reticulon 3 [Source:HGNC Symbol;Acc:HGNC:10469]	
PCCA	0,27	4,84	1,05E-06	6 x 48	13 q32	propionyl-CoA carboxylase alpha subunit [Source:HGNC Symbol;Acc:HGNC:8653]	
MARCKSL1	0,27	4,84	1,05E-06	4 x 46	1 p35	MARCKS like 1 [Source:HGNC Symbol;Acc:HGNC:7142]	
CDK7	0,27	4,83	1,06E-06	6 x 50	CHR_HSCHR5_	cyclin dependent kinase 7 [Source:HGNC Symbol;Acc:HGNC:1778]	
KXD1	0,27	4,83	1,06E-06	4 x 47	19 p13	KxDL motif containing 1 [Source:HGNC Symbol;Acc:HGNC:28420]	
SMARCA2	0,27	4,83	1,09E-06	6 x 49	9 p24	SWI/SNF related, matrix associated, actin dependent regulator of chromatin, subfamily a, member 2 [Source:HGNC Symbol;Acc:HGNC:110	
MED11	0,27	4,83	1,09E-06	2 x 46	17 p13	mediator complex subunit 11 [Source:HGNC Symbol;Acc:HGNC:32687]	
	0,27	4,81	1,17E-06	1 x 44			
ZBTB44	0,26	4,78	1,38E-06	7 x 50	11 q24	zinc finger and BTB domain containing 44 [Source:HGNC Symbol;Acc:HGNC:25001]	
YIF1B	0,26	4,77	1,46E-06	1 x 44	19 q13	Yip1 interacting factor homolog B, membrane trafficking protein [Source:HGNC Symbol;Acc:HGNC:30511]	
DNAJC19	0,26	4,76	1,51E-06	6 x 50	3 q26	DnaJ heat shock protein family (Hsp40) member C19 [Source:HGNC Symbol;Acc:HGNC:30528]	
CBR4	0,26	4,75	1,60E-06	4 x 50	4 q32	carbonyl reductase 4 [Source:HGNC Symbol;Acc:HGNC:25891]	
TRIM44	0,26	4,74	1,64E-06	7 x 50	11 p13	tripartite motif containing 44 [Source:HGNC Symbol;Acc:HGNC:19016]	
FAM127B	0,26	4,74	1,64E-06	1 x 44	X q26	family with sequence similarity 127 member B [Source:HGNC Symbol;Acc:HGNC:24514]	
LSM10	0,26	4,73	1,74E-06	3 x 44	1 p34	LSM10, U7 small nuclear RNA associated [Source:HGNC Symbol;Acc:HGNC:17562]	
GNB4	0,26	4,73	1,75E-06	3 x 46	3 q26	G protein subunit beta 4 [Source:HGNC Symbol;Acc:HGNC:20731]	
	0,26	4,72	1,76E-06	5 x 49			
SGCD	0,26	4,72	1,79E-06	1 x 48	5 q33	sarcoglycan delta [Source:HGNC Symbol;Acc:HGNC:10807]	
NAT1	0,26	4,71	1,85E-06	7 x 50	8 p22	N-acetyltransferase 1 (arylamine N-acetyltransferase) [Source:HGNC Symbol;Acc:HGNC:7645]	
PIK3R4	0,26	4,69	2,06E-06	2 x 46	3 q22	phosphoinositide-3-kinase regulatory subunit 4 [Source:HGNC Symbol;Acc:HGNC:8982]	


ALDH7A1	0,26	4,69	2,07E-06	3 x 48	5 q23	aldehyde dehydrogenase 7 family member A1 [Source:HGNC Symbol;Acc:HGNC:877]	
PPP1CA	0,26	4,67	2,26E-06	1 x 44	11 q13	protein phosphatase 1 catalytic subunit alpha [Source:HGNC Symbol;Acc:HGNC:9281]	
EXT2	0,26	4,67	2,27E-06	4 x 49	11 p11	exostosin glycosyltransferase 2 [Source:HGNC Symbol;Acc:HGNC:3513]	
ITGAE	0,26	4,67	2,28E-06	3 x 44	17 p13	integrin subunit alpha E [Source:HGNC Symbol;Acc:HGNC:6147]	
SACS	0,26	4,64	2,55E-06	6 x 50	13 q12	sacsin molecular chaperone [Source:HGNC Symbol;Acc:HGNC:10519]	
ARID5B	0,26	4,63	2,75E-06	5 x 50	10 q21	AT-rich interaction domain 5B [Source:HGNC Symbol;Acc:HGNC:17362]	
YIPF1	0,26	4,62	2,83E-06	4 x 48	1 p32	Yip1 domain family member 1 [Source:HGNC Symbol;Acc:HGNC:25231]	
EIF4E3	0,25	4,60	3,08E-06	7 x 50	3 p13	eukaryotic translation initiation factor 4E family member 3 [Source:HGNC Symbol;Acc:HGNC:31837]	
SCYL1	0,25	4,59	3,29E-06	1 x 45	11 q13	SCY1 like pseudokinase 1 [Source:HGNC Symbol;Acc:HGNC:14372]	
ARFGAP2	0,25	4,58	3,38E-06	6 x 50	11 p11	ADP ribosylation factor GTPase activating protein 2 [Source:HGNC Symbol;Acc:HGNC:13504]	
MPV17L2	0,25	4,57	3,61E-06	1 x 44	19 p13	MPV17 mitochondrial inner membrane protein like 2 [Source:HGNC Symbol;Acc:HGNC:28177]	
PARP4	0,25	4,56	3,72E-06	6 x 50	13 q12	poly(ADP-ribose) polymerase family member 4 [Source:HGNC Symbol;Acc:HGNC:271]	
TMEM60	0,25	4,55	3,87E-06	4 x 47	7 q11	transmembrane protein 60 [Source:HGNC Symbol;Acc:HGNC:21754]	
PKIG	0,25	4,53	4,31E-06	4 x 48	20 q13	protein kinase (cAMP-dependent, catalytic) inhibitor gamma [Source:HGNC Symbol;Acc:HGNC:9019]	
C1orf21	0,25	4,51	4,54E-06	1 x 47	1 q25	chromosome 1 open reading frame 21 [Source:HGNC Symbol;Acc:HGNC:15494]	
PXMP4	0,25	4,50	4,76E-06	6 x 50	20 q11	peroxisomal membrane protein 4 [Source:HGNC Symbol;Acc:HGNC:15920]	
AHI1	0,25	4,50	4,77E-06	5 x 50	6 q23	Abelson helper integration site 1 [Source:HGNC Symbol;Acc:HGNC:21575]	
FAM162A	0,25	4,50	4,88E-06	3 x 47	3 q21	family with sequence similarity 162 member A [Source:HGNC Symbol;Acc:HGNC:17865]	
GALK1	0,25	4,49	5,13E-06	2 x 44	17 q25	galactokinase 1 [Source:HGNC Symbol;Acc:HGNC:4118]	
DDRGK1	0,25	4,48	5,34E-06	1 x 44	20 p13	DDRGK domain containing 1 [Source:HGNC Symbol;Acc:HGNC:16110]	
MYCBP	0,25	4,48	5,37E-06	6 x 49	1 p34	MYC binding protein [Source:HGNC Symbol;Acc:HGNC:7554]	
MRPL22	0,25	4,47	5,62E-06	3 x 44	5 q33	mitochondrial ribosomal protein L22 [Source:HGNC Symbol;Acc:HGNC:14480]	
RNF24	0,25	4,46	5,75E-06	7 x 50	20 p13	ring finger protein 24 [Source:HGNC Symbol;Acc:HGNC:13779]	
RAB1B	0,25	4,45	5,90E-06	1 x 44	11 q13	RAB1B, member RAS oncogene family [Source:HGNC Symbol;Acc:HGNC:18370]	
POLR3GL	0,25	4,45	6,06E-06	4 x 47	1 q21	polymerase (RNA) III subunit G like [Source:HGNC Symbol;Acc:HGNC:28466]	
SFRP1	0,25	4,44	6,30E-06	6 x 50	8 p11	secreted frizzled related protein 1 [Source:HGNC Symbol;Acc:HGNC:10776]	
UTP23	0,25	4,42	6,85E-06	5 x 49	8 q24	UTP23, small subunit processome component [Source:HGNC Symbol;Acc:HGNC:28224]	
HIBADH	0,25	4,42	6,85E-06	4 x 46	7 p15	3-hydroxyisobutyrate dehydrogenase [Source:HGNC Symbol;Acc:HGNC:4907]	
NTMT1	0,24	4,39	7,87E-06	1 x 44	9 q34	N-terminal Xaa-Pro-Lys N-methyltransferase 1 [Source:HGNC Symbol;Acc:HGNC:23373]	
XRCC4	0,24	4,36	8,72E-06	6 x 50	5 q14	X-ray repair cross complementing 4 [Source:HGNC Symbol;Acc:HGNC:12831]	
PAFAH1B3	0,24	4,36	8,88E-06	1 x 44	19 q13	platelet activating factor acetylhydrolase 1b catalytic subunit 3 [Source:HGNC Symbol;Acc:HGNC:8576]	
TTC37	0,24	4,35	9,41E-06	5 x 47	5 q15	tetratricopeptide repeat domain 37 [Source:HGNC Symbol;Acc:HGNC:23639]	
	0,24	4,34	9,67E-06	1 x 45			


U2AF1L4	0,24	4,33	9,97E-06	4 x 46	19 q13	U2 small nuclear RNA auxiliary factor 1-like 4 [Source:HGNC Symbol;Acc:HGNC:23020]	
CD68	0,24	4,31	1,12E-05	6 x 50	17 p13	CD68 molecule [Source:HGNC Symbol;Acc:HGNC:1693]	
ST5	0,24	4,29	1,20E-05	1 x 45	11 p15	suppression of tumorigenicity 5 [Source:HGNC Symbol;Acc:HGNC:11350]	
DPH6	0,24	4,29	1,21E-05	4 x 50	15 q14	diphthamine biosynthesis 6 [Source:HGNC Symbol;Acc:HGNC:30543]	
ALG6	0,24	4,28	1,27E-05	4 x 47	1 p31	ALG6, alpha-1,3-glucosyltransferase [Source:HGNC Symbol;Acc:HGNC:23157]	
UBXN8	0,24	4,28	1,28E-05	5 x 47	8 p12	UBX domain protein 8 [Source:HGNC Symbol;Acc:HGNC:30307]	
APMAP	0,24	4,26	1,35E-05	4 x 46	20 p11	adipocyte plasma membrane associated protein [Source:HGNC Symbol;Acc:HGNC:13238]	
GLA	0,24	4,23	1,53E-05	4 x 48	X q22	galactosidase alpha [Source:HGNC Symbol;Acc:HGNC:4296]	
SERINC1	0,24	4,23	1,55E-05	6 x 50	6 q22	serine incorporator 1 [Source:HGNC Symbol;Acc:HGNC:13464]	
MKNK1	0,23	4,21	1,70E-05	6 x 49	1 p33	MAP kinase interacting serine/threonine kinase 1 [Source:HGNC Symbol;Acc:HGNC:7110]	
GCAT	0,23	4,20	1,73E-05	1 x 44	22 q13	glycine C-acetyltransferase [Source:HGNC Symbol;Acc:HGNC:4188]	
HARS	0,23	4,18	1,93E-05	1 x 44	5 q31	histidyl-tRNA synthetase [Source:HGNC Symbol;Acc:HGNC:4816]	
FEZ1	0,23	4,17	1,97E-05	6 x 48	11 q24	fasciculation and elongation protein zeta 1 [Source:HGNC Symbol;Acc:HGNC:3659]	
PBDC1	0,23	4,17	1,98E-05	6 x 50	X q13	polysaccharide biosynthesis domain containing 1 [Source:HGNC Symbol;Acc:HGNC:28790]	
ALG5	0,23	4,16	2,10E-05	5 x 47	13 q13	ALG5, dolichyl-phosphate beta-glucosyltransferase [Source:HGNC Symbol;Acc:HGNC:20266]	
PPP1R15A	0,23	4,14	2,22E-05	1 x 44	19 q13	protein phosphatase 1 regulatory subunit 15A [Source:HGNC Symbol;Acc:HGNC:14375]	
SIL1	0,23	4,14	2,24E-05	4 x 47	5 q31	SIL1 nucleotide exchange factor [Source:HGNC Symbol;Acc:HGNC:24624]	
TATDN1	0,23	4,14	2,25E-05	6 x 50	8 q24	TatD DNase domain containing 1 [Source:HGNC Symbol;Acc:HGNC:24220]	
C8orf59	0,23	4,13	2,31E-05	7 x 50	8 q21	chromosome 8 open reading frame 59 [Source:HGNC Symbol;Acc:HGNC:32235]	
CTNNAL1	0,23	4,12	2,42E-05	1 x 44	9 q31	catenin alpha like 1 [Source:HGNC Symbol;Acc:HGNC:2512]	
C6orf203	0,23	4,12	2,42E-05	4 x 49	6 q21	chromosome 6 open reading frame 203 [Source:HGNC Symbol;Acc:HGNC:17971]	
PRCP	0,23	4,09	2,71E-05	2 x 46	11 q14	prolylcarboxypeptidase [Source:HGNC Symbol;Acc:HGNC:9344]	
WDR41	0,23	4,07	3,02E-05	4 x 48	5 q14	WD repeat domain 41 [Source:HGNC Symbol;Acc:HGNC:25601]	
DCTN3	0,23	4,05	3,24E-05	5 x 47	9 p13	dynactin subunit 3 [Source:HGNC Symbol;Acc:HGNC:2713]	
AASS	0,22	4,03	3,56E-05	3 x 50	7 q31	aminoadipate-semialdehyde synthase [Source:HGNC Symbol;Acc:HGNC:17366]	
ATP1A1	0,22	4,00	3,93E-05	1 x 44	1 p13	ATPase Na+/K+ transporting subunit alpha 1 [Source:HGNC Symbol;Acc:HGNC:799]	
NOL8	0,22	4,00	3,99E-05	5 x 50	9 q22	nucleolar protein 8 [Source:HGNC Symbol;Acc:HGNC:23387]	
PSMG3	0,22	4,00	4,01E-05	5 x 47	7 p22	proteasome assembly chaperone 3 [Source:HGNC Symbol;Acc:HGNC:22420]	
MAPRE2	0,22	3,97	4,47E-05	6 x 50	18 q12	microtubule associated protein RP/EB family member 2 [Source:HGNC Symbol;Acc:HGNC:6891]	
MDP1	0,22	3,96	4,62E-05	6 x 50	14 q12	magnesium dependent phosphatase 1 [Source:HGNC Symbol;Acc:HGNC:28781]	
LACTB2	0,22	3,95	4,83E-05	4 x 46	8 q13	lactamase beta 2 [Source:HGNC Symbol;Acc:HGNC:18512]	
MED31	0,22	3,95	4,91E-05	4 x 48	17 p13	mediator complex subunit 31 [Source:HGNC Symbol;Acc:HGNC:24260]	
NRIP3	0,22	3,94	5,07E-05	6 x 50	11 p15	nuclear receptor interacting protein 3 [Source:HGNC Symbol;Acc:HGNC:1167]	


DYNLT1	0,22	3,93	5,16E-05	3 x 44	6 q25	dynein light chain Tctex-type 1 [Source:HGNC Symbol;Acc:HGNC:11697]	
ISY1	0,22	3,92	5,56E-05	5 x 47	3 q21	ISY1 splicing factor homolog [Source:HGNC Symbol;Acc:HGNC:29201]	
TBCC	0,22	3,91	5,74E-05	1 x 44	6 p21	tubulin folding cofactor C [Source:HGNC Symbol;Acc:HGNC:11580]	
MAT2B	0,22	3,90	5,88E-05	1 x 45	5 q34	methionine adenosyltransferase 2B [Source:HGNC Symbol;Acc:HGNC:6905]	
ATG13	0,22	3,90	5,90E-05	5 x 49	11 p11	autophagy related 13 [Source:HGNC Symbol;Acc:HGNC:29091]	
BBS2	0,22	3,89	6,05E-05	6 x 48	16 q13	Bardet-Biedl syndrome 2 [Source:HGNC Symbol;Acc:HGNC:967]	
TIMM44	0,22	3,89	6,06E-05	6 x 49	19 p13	translocase of inner mitochondrial membrane 44 [Source:HGNC Symbol;Acc:HGNC:17316]	
RABEPK	0,22	3,89	6,13E-05	5 x 47	9 q33	Rab9 effector protein with kelch motifs [Source:HGNC Symbol;Acc:HGNC:16896]	
EHMT1	0,22	3,88	6,28E-05	1 x 44	9 q34	euchromatic histone lysine methyltransferase 1 [Source:HGNC Symbol;Acc:HGNC:24650]	
RAB24	0,22	3,88	6,39E-05	1 x 44	5 q35	RAB24, member RAS oncogene family [Source:HGNC Symbol;Acc:HGNC:9765]	
UPP1	0,22	3,88	6,40E-05	5 x 49	7 p12	uridine phosphorylase 1 [Source:HGNC Symbol;Acc:HGNC:12576]	
WDR36	0,22	3,87	6,72E-05	6 x 50	5 q22	WD repeat domain 36 [Source:HGNC Symbol;Acc:HGNC:30696]	
CTSH	0,21	3,84	7,43E-05	5 x 47	15 q25	cathepsin H [Source:HGNC Symbol;Acc:HGNC:2535]	
C2orf74	0,21	3,79	8,92E-05	5 x 47	2 p15	chromosome 2 open reading frame 74 [Source:HGNC Symbol;Acc:HGNC:34439]	
TGDS	0,21	3,78	9,38E-05	6 x 50	13 q32	TDP-glucose 4,6-dehydratase [Source:HGNC Symbol;Acc:HGNC:20324]	
TAOK3	0,21	3,78	9,40E-05	1 x 45	12 q24	TAO kinase 3 [Source:HGNC Symbol;Acc:HGNC:18133]	
RMDN1	0,21	3,74	0,0001084	2 x 44	8 q21	regulator of microtubule dynamics 1 [Source:HGNC Symbol;Acc:HGNC:24285]	
PPIL4	0,21	3,70	0,0001269	6 x 50	6 q25	peptidylprolyl isomerase like 4 [Source:HGNC Symbol;Acc:HGNC:15702]	
PYURF	0,20	3,65	0,000152	1 x 44	4 q22	PIGY upstream reading frame [Source:HGNC Symbol;Acc:HGNC:44317]	
FUNDC2	0,20	3,63	0,0001681	5 x 47	X q28	FUN14 domain containing 2 [Source:HGNC Symbol;Acc:HGNC:24925]	
GEMIN7	0,20	3,61	0,0001764	5 x 47	19 q13	gem nuclear organelle associated protein 7 [Source:HGNC Symbol;Acc:HGNC:20045]	
RALA	0,20	3,61	0,0001784	4 x 46	7 p14	RALA Ras like proto-oncogene A [Source:HGNC Symbol;Acc:HGNC:9839]	
PCYT1A	0,20	3,58	0,0001989	6 x 48	3 q29	phosphate cytidylyltransferase 1, choline, alpha [Source:HGNC Symbol;Acc:HGNC:8754]	
REEP5	0,20	3,51	0,0002578	2 x 44	5 q22	receptor accessory protein 5 [Source:HGNC Symbol;Acc:HGNC:30077]	
TAX1BP3	0,20	3,51	0,0002622	6 x 49	17 p13	Tax1 binding protein 3 [Source:HGNC Symbol;Acc:HGNC:30684]	
NOL12	0,20	3,48	0,0002908	3 x 44	22 q13	nucleolar protein 12 [Source:HGNC Symbol;Acc:HGNC:28585]	
PHYH	0,20	3,48	0,0002924	6 x 48	10 p13	phytanoyl-CoA 2-hydroxylase [Source:HGNC Symbol;Acc:HGNC:8940]	
HIST1H2BK	0,19	3,47	0,0002981	4 x 47	6 p22	histone cluster 1, H2bk [Source:HGNC Symbol;Acc:HGNC:13954]	
NUDCD3	0,19	3,47	0,0003004	7 x 50	7 p13	NudC domain containing 3 [Source:HGNC Symbol;Acc:HGNC:22208]	
OXR1	0,19	3,46	0,0003089	6 x 50	8 q23	oxidation resistance 1 [Source:HGNC Symbol;Acc:HGNC:15822]	
MTMR6	0,19	3,44	0,0003354	7 x 50	13 q12	myotubularin related protein 6 [Source:HGNC Symbol;Acc:HGNC:7453]	
MCCC1	0,19	3,43	0,000344	3 x 44	3 q27	methylcrotonoyl-CoA carboxylase 1 [Source:HGNC Symbol;Acc:HGNC:6936]	
HSD17B7	0,19	3,40	0,0003778	7 x 50	1 q23	hydroxysteroid 17-beta dehydrogenase 7 [Source:HGNC Symbol;Acc:HGNC:5215]	


TTC17	0,19	3,36	0,0004356	7 x 50	11 p12	tetratricopeptide repeat domain 17 [Source:HGNC Symbol;Acc:HGNC:25596]	
IDH3G	0,19	3,32	0,0005048	6 x 50	X q28	isocitrate dehydrogenase 3 (NAD(+)) gamma [Source:HGNC Symbol;Acc:HGNC:5386]	
ZFP91	0,19	3,30	0,0005377	7 x 50	11 q12	ZFP91 zinc finger protein [Source:HGNC Symbol;Acc:HGNC:14983]	
TCEB3	0,18	3,22	0,0007103	7 x 50	1 p36	transcription elongation factor B subunit 3 [Source:HGNC Symbol;Acc:HGNC:11620]	
MFSD1	0,17	3,06	0,0012094	1 x 44	3 q25	major facilitator superfamily domain containing 1 [Source:HGNC Symbol;Acc:HGNC:25874]	
TCEAL8	0,16	2,91	0,0019694	6 x 48	X q22	transcription elongation factor A like 8 [Source:HGNC Symbol;Acc:HGNC:28683]	


Genes upregulated in wt/mut (NRAS_mut)	
Symbol	correlatio	->t.score	->p.value	Metagene	Chromosome	Description	
AXL	0,96	63,59	0	50 x 50	19 q13	AXL receptor tyrosine kinase [Source:HGNC Symbol;Acc:HGNC:905]	
CDH13	0,94	49,76	0	50 x 50	16 q23	cadherin 13 [Source:HGNC Symbol;Acc:HGNC:1753]	
CD74	0,93	43,65	0	50 x 50	5 q33	CD74 molecule [Source:HGNC Symbol;Acc:HGNC:1697]	
HLA-DRA	0,93	42,98	0	50 x 50	CHR_HSCHR6_MHC	major histocompatibility complex, class II, DR alpha [Source:HGNC Symbol;Acc:HGNC:4947]	
CSAG1	0,91	38,15	0	50 x 50	X q28	chondrosarcoma associated gene 1 [Source:HGNC Symbol;Acc:HGNC:24294]	
SERPINB2	0,91	37,29	0	50 x 50	18 q21	serpin family B member 2 [Source:HGNC Symbol;Acc:HGNC:8584]	
TGFBI	0,90	36,69	0	50 x 50	5 q31	transforming growth factor beta induced [Source:HGNC Symbol;Acc:HGNC:11771]	
THBS1	0,89	33,57	0	50 x 50	15 q14	thrombospondin 1 [Source:HGNC Symbol;Acc:HGNC:11785]	
GAGE1	0,88	32,94	0	50 x 49	X p11	G antigen 1 [Source:HGNC Symbol;Acc:HGNC:4098]	
HLA-DRB1	0,88	31,81	0	50 x 50	CHR_HSCHR6_MHC	major histocompatibility complex, class II, DR beta 1 [Source:HGNC Symbol;Acc:HGNC:4948]	
S100A6	0,87	31,56	0	50 x 50	1 q21	S100 calcium binding protein A6 [Source:HGNC Symbol;Acc:HGNC:10496]	
DHRS3	0,87	30,65	0	50 x 50	1 p36	dehydrogenase/reductase 3 [Source:HGNC Symbol;Acc:HGNC:17693]	
LOXL3	0,87	30,56	0	50 x 50	2 p13	lysyl oxidase like 3 [Source:HGNC Symbol;Acc:HGNC:13869]	
PMEPA1	0,87	30,27	0	50 x 50	20 q13	prostate transmembrane protein, androgen induced 1 [Source:HGNC Symbol;Acc:HGNC:14107]	
TIMP3	0,86	29,78	0	50 x 50	22 q12	TIMP metallopeptidase inhibitor 3 [Source:HGNC Symbol;Acc:HGNC:11822]	
GPRC5A	0,86	29,51	0	50 x 50	12 p13	G protein-coupled receptor class C group 5 member A [Source:HGNC Symbol;Acc:HGNC:9836]	
CADM1	0,86	29,13	0	50 x 50	11 q23	cell adhesion molecule 1 [Source:HGNC Symbol;Acc:HGNC:5951]	
HLA-DMA	0,85	28,33	0	50 x 50	CHR_HSCHR6_MHC	major histocompatibility complex, class II, DM alpha [Source:HGNC Symbol;Acc:HGNC:4934]	
TMSB4X	0,85	28,32	0	50 x 50	X p22	thymosin beta 4, X-linked [Source:HGNC Symbol;Acc:HGNC:11881]	
ITGB8	0,85	27,90	0	50 x 50	7 p21	integrin subunit beta 8 [Source:HGNC Symbol;Acc:HGNC:6163]	
PLAUR	0,84	27,56	0	50 x 50	19 q13	plasminogen activator, urokinase receptor [Source:HGNC Symbol;Acc:HGNC:9053]	
S100A16	0,84	27,34	0	50 x 50	1 q21	S100 calcium binding protein A16 [Source:HGNC Symbol;Acc:HGNC:20441]	
MGLL	0,84	26,71	0	50 x 50	3 q21	monoglyceride lipase [Source:HGNC Symbol;Acc:HGNC:17038]	
CD109	0,83	26,35	0	50 x 50	6 q13	CD109 molecule [Source:HGNC Symbol;Acc:HGNC:21685]	
MIA	0,83	26,20	0	50 x 50	19 q13	melanoma inhibitory activity [Source:HGNC Symbol;Acc:HGNC:7076]	
TPM1	0,83	25,83	0	50 x 50	15 q22	tropomyosin 1 (alpha) [Source:HGNC Symbol;Acc:HGNC:12010]	
TMSB10	0,82	25,26	0	49 x 48	2 p11	thymosin beta 10 [Source:HGNC Symbol;Acc:HGNC:11879]	
TNC	0,82	24,97	0	50 x 50	9 q33	tenascin C [Source:HGNC Symbol;Acc:HGNC:5318]	
MAGEA6	0,82	24,85	0	50 x 49	X q28	MAGE family member A6 [Source:HGNC Symbol;Acc:HGNC:6804]	
NTM	0,82	24,63	0	50 x 50	11 q25	neurotrimin [Source:HGNC Symbol;Acc:HGNC:17941]	
S100A10	0,82	24,60	0	50 x 50	1 q21	S100 calcium binding protein A10 [Source:HGNC Symbol;Acc:HGNC:10487]	


FAM114A1	0,81	24,51	0	50 x 50	4 p14	family with sequence similarity 114 member A1 [Source:HGNC Symbol;Acc:HGNC:25087]	
NIPAL3	0,81	24,48	0	50 x 50	1 p36	NIPA like domain containing 3 [Source:HGNC Symbol;Acc:HGNC:25233]	
RND3	0,81	24,30	0	50 x 50	2 q23	Rho family GTPase 3 [Source:HGNC Symbol;Acc:HGNC:671]	
GPX1	0,81	24,11	0	50 x 50	3 p21	glutathione peroxidase 1 [Source:HGNC Symbol;Acc:HGNC:4553]	
DOCK5	0,81	24,05	0	50 x 50	8 p21	dedicator of cytokinesis 5 [Source:HGNC Symbol;Acc:HGNC:23476]	
IGFBP7	0,81	24,00	0	50 x 50	4 q12	insulin like growth factor binding protein 7 [Source:HGNC Symbol;Acc:HGNC:5476]	
	0,81	23,78	0	50 x 50			
IL24	0,80	23,63	0	50 x 50	1 q32	interleukin 24 [Source:HGNC Symbol;Acc:HGNC:11346]	
HLA-DQB1	0,80	23,52	0	50 x 48	CHR_HSCHR6_MHC	major histocompatibility complex, class II, DQ beta 1 [Source:HGNC Symbol;Acc:HGNC:4944]	
SVIL	0,80	23,33	0	50 x 50	10 p11	supervillin [Source:HGNC Symbol;Acc:HGNC:11480]	
ANXA1	0,80	23,18	0	50 x 50	9 q21	annexin A1 [Source:HGNC Symbol;Acc:HGNC:533]	
TM4SF1	0,80	23,17	0	50 x 50	3 q25	transmembrane 4 L six family member 1 [Source:HGNC Symbol;Acc:HGNC:11853]	
FLT1	0,80	23,03	0	50 x 50	13 q12	fms related tyrosine kinase 1 [Source:HGNC Symbol;Acc:HGNC:3763]	
FHL2	0,78	21,70	0	50 x 50	2 q12	four and a half LIM domains 2 [Source:HGNC Symbol;Acc:HGNC:3703]	
MYOF	0,77	21,39	0	50 x 50	10 q23	myoferlin [Source:HGNC Symbol;Acc:HGNC:3656]	
UBB	0,76	20,44	0	50 x 50	17 p11	ubiquitin B [Source:HGNC Symbol;Acc:HGNC:12463]	
C12orf75	0,76	20,41	0	50 x 50	12 q23	chromosome 12 open reading frame 75 [Source:HGNC Symbol;Acc:HGNC:35164]	
HLA-DRB5	0,76	20,40	0	50 x 49	6 p21	major histocompatibility complex, class II, DR beta 5 [Source:HGNC Symbol;Acc:HGNC:4953]	
TPM2	0,76	20,30	0	50 x 48	9 p13	tropomyosin 2 (beta) [Source:HGNC Symbol;Acc:HGNC:12011]	
UBASH3B	0,76	20,11	0	50 x 49	11 q24	ubiquitin associated and SH3 domain containing B [Source:HGNC Symbol;Acc:HGNC:29884]	
TAC1	0,75	19,99	0	50 x 50	7 q21	tachykinin precursor 1 [Source:HGNC Symbol;Acc:HGNC:11517]	
MAGEA12	0,75	19,84	0	50 x 49	X q28	MAGE family member A12 [Source:HGNC Symbol;Acc:HGNC:6799]	
ITGA6	0,75	19,64	0	50 x 50	2 q31	integrin subunit alpha 6 [Source:HGNC Symbol;Acc:HGNC:6142]	
SOX9	0,74	19,38	0	50 x 48	17 q24	SRY-box 9 [Source:HGNC Symbol;Acc:HGNC:11204]	
FN1	0,74	19,37	0	50 x 50	2 q35	fibronectin 1 [Source:HGNC Symbol;Acc:HGNC:3778]	
WNT5A	0,74	19,34	0	50 x 47	3 p14	Wnt family member 5A [Source:HGNC Symbol;Acc:HGNC:12784]	
PRSS23	0,74	19,31	0	50 x 50	11 q14	protease, serine 23 [Source:HGNC Symbol;Acc:HGNC:14370]	
HLA-DQA1	0,74	19,26	0	49 x 47	CHR_HSCHR6_MHC	major histocompatibility complex, class II, DQ alpha 1 [Source:HGNC Symbol;Acc:HGNC:4942]	
S100A4	0,74	19,24	0	50 x 50	1 q21	S100 calcium binding protein A4 [Source:HGNC Symbol;Acc:HGNC:10494]	
ANKRD1	0,74	19,20	0	50 x 49	10 q23	ankyrin repeat domain 1 [Source:HGNC Symbol;Acc:HGNC:15819]	
SLITRK6	0,73	18,87	0	50 x 48	13 q31	SLIT and NTRK like family member 6 [Source:HGNC Symbol;Acc:HGNC:23503]	
GULP1	0,73	18,73	0	50 x 50	2 q32	GULP, engulfment adaptor PTB domain containing 1 [Source:HGNC Symbol;Acc:HGNC:18649]	
S100B	0,73	18,70	0	50 x 50	21 q22	S100 calcium binding protein B [Source:HGNC Symbol;Acc:HGNC:10500]	


COL8A1	0,73	18,64	0	50 x 48	3 q12	collagen type VIII alpha 1 [Source:HGNC Symbol;Acc:HGNC:2215]	
CDK6	0,73	18,54	0	50 x 49	7 q21	cyclin dependent kinase 6 [Source:HGNC Symbol;Acc:HGNC:1777]	
PLAT	0,73	18,44	0	50 x 50	8 p11	plasminogen activator, tissue type [Source:HGNC Symbol;Acc:HGNC:9051]	
CD163L1	0,72	18,38	0	50 x 48	12 p13	CD163 molecule like 1 [Source:HGNC Symbol;Acc:HGNC:30375]	
DEPDC7	0,72	18,32	0	50 x 50	11 p13	DEP domain containing 7 [Source:HGNC Symbol;Acc:HGNC:29899]	
CYTL1	0,72	18,16	0	50 x 49	4 p16	cytokine like 1 [Source:HGNC Symbol;Acc:HGNC:24435]	
BEX1	0,72	18,08	0	50 x 49	X q22	brain expressed X-linked 1 [Source:HGNC Symbol;Acc:HGNC:1036]	
MAGEA3	0,72	18,06	0	50 x 48	X q28	MAGE family member A3 [Source:HGNC Symbol;Acc:HGNC:6801]	
CD96	0,72	18,06	0	50 x 48	3 q13	CD96 molecule [Source:HGNC Symbol;Acc:HGNC:16892]	
SERPINE2	0,72	17,93	0	50 x 50	2 q36	serpin family E member 2 [Source:HGNC Symbol;Acc:HGNC:8951]	
HLA-DPA1	0,71	17,59	0	49 x 50	6 p21	major histocompatibility complex, class II, DP alpha 1 [Source:HGNC Symbol;Acc:HGNC:4938]	
IGFBP5	0,71	17,37	0	50 x 48	2 q35	insulin like growth factor binding protein 5 [Source:HGNC Symbol;Acc:HGNC:5474]	
CRIM1	0,70	17,30	0	50 x 49	CHR_HSCHR2_1_C	cysteine rich transmembrane BMP regulator 1 (chordin-like) [Source:HGNC Symbol;Acc:HGNC:2359]	
CD9	0,70	17,27	0	50 x 50	12 p13	CD9 molecule [Source:HGNC Symbol;Acc:HGNC:1709]	
ADAM19	0,70	17,18	0	49 x 50	5 q33	ADAM metallopeptidase domain 19 [Source:HGNC Symbol;Acc:HGNC:197]	
IL7R	0,70	17,04	0	50 x 49	5 p13	interleukin 7 receptor [Source:HGNC Symbol;Acc:HGNC:6024]	
FRMD6	0,70	16,95	0	50 x 50	14 q22	FERM domain containing 6 [Source:HGNC Symbol;Acc:HGNC:19839]	
APOD	0,70	16,91	0	50 x 50	3 q29	apolipoprotein D [Source:HGNC Symbol;Acc:HGNC:612]	
PRNP	0,70	16,90	0	50 x 50	20 p13	prion protein [Source:HGNC Symbol;Acc:HGNC:9449]	
TIMP1	0,69	16,86	0	50 x 50	X p11	TIMP metallopeptidase inhibitor 1 [Source:HGNC Symbol;Acc:HGNC:11820]	
FOXC2	0,69	16,83	0	49 x 46	16 q24	forkhead box C2 [Source:HGNC Symbol;Acc:HGNC:3801]	
GAPDH	0,69	16,82	0	50 x 44	12 p13	glyceraldehyde-3-phosphate dehydrogenase [Source:HGNC Symbol;Acc:HGNC:4141]	
PPFIBP1	0,69	16,75	0	50 x 50	12 p11	PPFIA binding protein 1 [Source:HGNC Symbol;Acc:HGNC:9249]	
HLA-DMB	0,69	16,63	0	48 x 47	CHR_HSCHR6_MHC	major histocompatibility complex, class II, DM beta [Source:HGNC Symbol;Acc:HGNC:4935]	
CAV1	0,69	16,59	0	50 x 50	7 q31	caveolin 1 [Source:HGNC Symbol;Acc:HGNC:1527]	
CEBPZOS	0,69	16,59	0	50 x 50	2 p22	CEBPZ opposite strand [Source:HGNC Symbol;Acc:HGNC:49288]	
MT1E	0,69	16,56	0	50 x 48	16 q13	metallothionein 1E [Source:HGNC Symbol;Acc:HGNC:7397]	
DCBLD2	0,68	16,18	0	50 x 50	3 q12	discoidin, CUB and LCCL domain containing 2 [Source:HGNC Symbol;Acc:HGNC:24627]	
DLC1	0,68	16,16	0	50 x 50	8 p22	DLC1 Rho GTPase activating protein [Source:HGNC Symbol;Acc:HGNC:2897]	
LYPD1	0,68	16,12	0	49 x 49	2 q21	LY6/PLAUR domain containing 1 [Source:HGNC Symbol;Acc:HGNC:28431]	
LGALS1	0,68	16,03	0	50 x 50	22 q13	galectin 1 [Source:HGNC Symbol;Acc:HGNC:6561]	
DPYD	0,68	15,99	0	50 x 49	1 p21	dihydropyrimidine dehydrogenase [Source:HGNC Symbol;Acc:HGNC:3012]	
YBX3	0,67	15,97	0	50 x 49	12 p13	Y-box binding protein 3 [Source:HGNC Symbol;Acc:HGNC:2428]	


COL9A3	0,67	15,86	0	50 x 47	20 q13	collagen type IX alpha 3 [Source:HGNC Symbol;Acc:HGNC:2219]	
MMP2	0,67	15,81	0	50 x 47	16 q12	matrix metallopeptidase 2 [Source:HGNC Symbol;Acc:HGNC:7166]	
TMEM47	0,67	15,72	0	49 x 50	X p21	transmembrane protein 47 [Source:HGNC Symbol;Acc:HGNC:18515]	
FTL	0,67	15,61	0	47 x 47	19 q13	ferritin, light polypeptide [Source:HGNC Symbol;Acc:HGNC:3999]	
SAMD12	0,67	15,58	0	50 x 48	8 q24	sterile alpha motif domain containing 12 [Source:HGNC Symbol;Acc:HGNC:31750]	
PDE1C	0,67	15,56	0	49 x 49	7 p14	phosphodiesterase 1C [Source:HGNC Symbol;Acc:HGNC:8776]	
	0,66	15,48	0	50 x 50			
NT5E	0,66	15,39	0	50 x 48	6 q14	5'-nucleotidase ecto [Source:HGNC Symbol;Acc:HGNC:8021]	
SNRPB2	0,66	15,38	0	50 x 46	20 p12	small nuclear ribonucleoprotein polypeptide B2 [Source:HGNC Symbol;Acc:HGNC:11155]	
RAB21	0,66	15,32	0	50 x 48	12 q21	RAB21, member RAS oncogene family [Source:HGNC Symbol;Acc:HGNC:18263]	
TLE3	0,66	15,30	0	50 x 48	15 q23	transducin like enhancer of split 3 [Source:HGNC Symbol;Acc:HGNC:11839]	
TSC22D1	0,66	15,25	0	50 x 50	13 q14	TSC22 domain family member 1 [Source:HGNC Symbol;Acc:HGNC:16826]	
SLC1A5	0,66	15,19	0	49 x 50	19 q13	solute carrier family 1 member 5 [Source:HGNC Symbol;Acc:HGNC:10943]	
COL19A1	0,66	15,17	0	47 x 49	6 q13	collagen type XIX alpha 1 chain [Source:HGNC Symbol;Acc:HGNC:2196]	
MRPL40	0,66	15,16	0	50 x 50	22 q11	mitochondrial ribosomal protein L40 [Source:HGNC Symbol;Acc:HGNC:14491]	
YWHAZ	0,65	15,00	0	50 x 44	8 q22	tyrosine 3-monooxygenase/tryptophan 5-monooxygenase activation protein zeta [Source:HGNC Symbol;Acc:HGNC:12	
IGFBP3	0,65	14,99	0	50 x 48	7 p12	insulin like growth factor binding protein 3 [Source:HGNC Symbol;Acc:HGNC:5472]	
HOXA3	0,65	14,95	0	48 x 47	7 p15	homeobox A3 [Source:HGNC Symbol;Acc:HGNC:5104]	
GAS7	0,65	14,94	0	50 x 50	17 p13	growth arrest specific 7 [Source:HGNC Symbol;Acc:HGNC:4169]	
FKBP9	0,65	14,89	0	47 x 50	7 p14	FK506 binding protein 9 [Source:HGNC Symbol;Acc:HGNC:3725]	
CDC42EP3	0,65	14,77	0	50 x 49	2 p22	CDC42 effector protein 3 [Source:HGNC Symbol;Acc:HGNC:16943]	
S100A11	0,64	14,73	0	46 x 50	1 q21	S100 calcium binding protein A11 [Source:HGNC Symbol;Acc:HGNC:10488]	
GNG2	0,64	14,72	0	49 x 50	14 q22	G protein subunit gamma 2 [Source:HGNC Symbol;Acc:HGNC:4404]	
BMP8B	0,64	14,70	0	47 x 47	1 p34	bone morphogenetic protein 8b [Source:HGNC Symbol;Acc:HGNC:1075]	
FGF13	0,64	14,69	0	50 x 49	X q27	fibroblast growth factor 13 [Source:HGNC Symbol;Acc:HGNC:3670]	
ALDH1A3	0,64	14,60	0	48 x 50	15 q26	aldehyde dehydrogenase 1 family member A3 [Source:HGNC Symbol;Acc:HGNC:409]	
SDC4	0,64	14,51	0	50 x 48	20 q13	syndecan 4 [Source:HGNC Symbol;Acc:HGNC:10661]	
RNF128	0,64	14,43	0	50 x 47	X q22	ring finger protein 128, E3 ubiquitin protein ligase [Source:HGNC Symbol;Acc:HGNC:21153]	
TNFAIP6	0,63	14,25	0	50 x 48	2 q23	TNF alpha induced protein 6 [Source:HGNC Symbol;Acc:HGNC:11898]	
COL4A2	0,63	14,00	0	48 x 47	13 q34	collagen type IV alpha 2 [Source:HGNC Symbol;Acc:HGNC:2203]	
EHBP1	0,63	13,99	0	50 x 49	2 p15	EH domain binding protein 1 [Source:HGNC Symbol;Acc:HGNC:29144]	
COL4A1	0,62	13,94	0	48 x 48	13 q34	collagen type IV alpha 1 chain [Source:HGNC Symbol;Acc:HGNC:2202]	
CAMK2D	0,62	13,90	0	50 x 50	4 q26	calcium/calmodulin dependent protein kinase II delta [Source:HGNC Symbol;Acc:HGNC:1462]	


PLP2	0,62	13,74	0	50 x 50	X p11	proteolipid protein 2 [Source:HGNC Symbol;Acc:HGNC:9087]	
DSCR8	0,62	13,69	0	50 x 47	21 q22	Down syndrome critical region 8 [Source:HGNC Symbol;Acc:HGNC:16707]	
ITGA4	0,62	13,68	0	49 x 46	2 q31	integrin subunit alpha 4 [Source:HGNC Symbol;Acc:HGNC:6140]	
TPM4	0,61	13,61	0	48 x 50	19 p13	tropomyosin 4 [Source:HGNC Symbol;Acc:HGNC:12013]	
GDI2	0,61	13,61	0	50 x 48	10 p15	GDP dissociation inhibitor 2 [Source:HGNC Symbol;Acc:HGNC:4227]	
SH3KBP1	0,61	13,58	0	50 x 50	X p22	SH3 domain containing kinase binding protein 1 [Source:HGNC Symbol;Acc:HGNC:13867]	
DKK1	0,61	13,54	0	50 x 49	10 q21	dickkopf WNT signaling pathway inhibitor 1 [Source:HGNC Symbol;Acc:HGNC:2891]	
SOX5	0,61	13,52	0	48 x 50	12 p12	SRY-box 5 [Source:HGNC Symbol;Acc:HGNC:11201]	
CALD1	0,61	13,52	0	50 x 50	7 q33	caldesmon 1 [Source:HGNC Symbol;Acc:HGNC:1441]	
ERRFI1	0,61	13,48	0	50 x 46	1 p36	ERBB receptor feedback inhibitor 1 [Source:HGNC Symbol;Acc:HGNC:18185]	
IL1RAPL1	0,61	13,47	0	48 x 49	X p21	interleukin 1 receptor accessory protein like 1 [Source:HGNC Symbol;Acc:HGNC:5996]	
CTTNBP2NL	0,61	13,42	0	50 x 48	1 p13	CTTNBP2 N-terminal like [Source:HGNC Symbol;Acc:HGNC:25330]	
LPCAT2	0,61	13,42	0	50 x 48	16 q12	lysophosphatidylcholine acyltransferase 2 [Source:HGNC Symbol;Acc:HGNC:26032]	
SPAG16	0,61	13,34	0	50 x 45	2 q34	sperm associated antigen 16 [Source:HGNC Symbol;Acc:HGNC:23225]	
GPC6	0,60	13,27	0	47 x 50	13 q31	glypican 6 [Source:HGNC Symbol;Acc:HGNC:4454]	
VIM	0,60	13,24	0	48 x 50	10 p13	vimentin [Source:HGNC Symbol;Acc:HGNC:12692]	
CXCL8	0,60	13,19	0	50 x 47	4 q13	C-X-C motif chemokine ligand 8 [Source:HGNC Symbol;Acc:HGNC:6025]	
PDGFC	0,60	13,13	0	50 x 47	4 q32	platelet derived growth factor C [Source:HGNC Symbol;Acc:HGNC:8801]	
ARSJ	0,60	13,11	0	47 x 48	4 q26	arylsulfatase family member J [Source:HGNC Symbol;Acc:HGNC:26286]	
HDAC9	0,60	13,10	0	50 x 50	7 p21	histone deacetylase 9 [Source:HGNC Symbol;Acc:HGNC:14065]	
PRKCA	0,60	13,09	0	49 x 50	17 q24	protein kinase C alpha [Source:HGNC Symbol;Acc:HGNC:9393]	
PROCR	0,60	13,07	0	50 x 47	20 q11	protein C receptor [Source:HGNC Symbol;Acc:HGNC:9452]	
MT2A	0,60	12,99	0	50 x 49	16 q13	metallothionein 2A [Source:HGNC Symbol;Acc:HGNC:7406]	
SLX4IP	0,60	12,96	0	47 x 50	20 p12	SLX4 interacting protein [Source:HGNC Symbol;Acc:HGNC:16225]	
CCL2	0,59	12,92	0	50 x 50	17 q12	C-C motif chemokine ligand 2 [Source:HGNC Symbol;Acc:HGNC:10618]	
DPYSL2	0,59	12,86	0	49 x 50	8 p21	dihydropyrimidinase like 2 [Source:HGNC Symbol;Acc:HGNC:3014]	
UGDH	0,59	12,86	0	50 x 48	4 p14	UDP-glucose 6-dehydrogenase [Source:HGNC Symbol;Acc:HGNC:12525]	
RAB8B	0,59	12,65	0	50 x 48	15 q22	RAB8B, member RAS oncogene family [Source:HGNC Symbol;Acc:HGNC:30273]	
C16orf52	0,58	12,47	0	47 x 48	16 p12	chromosome 16 open reading frame 52 [Source:HGNC Symbol;Acc:HGNC:27087]	
HMGA2	0,58	12,46	0	50 x 46	12 q14	high mobility group AT-hook 2 [Source:HGNC Symbol;Acc:HGNC:5009]	
FLRT3	0,58	12,45	0	48 x 50	20 p12	fibronectin leucine rich transmembrane protein 3 [Source:HGNC Symbol;Acc:HGNC:3762]	
SH3BGRL3	0,58	12,43	0	50 x 47	1 p36	SH3 domain binding glutamate rich protein like 3 [Source:HGNC Symbol;Acc:HGNC:15568]	
TRPV2	0,58	12,42	0	50 x 46	17 p11	transient receptor potential cation channel subfamily V member 2 [Source:HGNC Symbol;Acc:HGNC:18082]	


POU3F2	0,58	12,39	0	46 x 48	6 q16	POU class 3 homeobox 2 [Source:HGNC Symbol;Acc:HGNC:9215]	
NCEH1	0,58	12,36	0	50 x 48	3 q26	neutral cholesterol ester hydrolase 1 [Source:HGNC Symbol;Acc:HGNC:29260]	
PRKACB	0,58	12,29	0	50 x 48	1 p31	protein kinase cAMP-activated catalytic subunit beta [Source:HGNC Symbol;Acc:HGNC:9381]	
NRP1	0,58	12,28	0	50 x 49	10 p11	neuropilin 1 [Source:HGNC Symbol;Acc:HGNC:8004]	
C3orf14	0,57	12,18	0	49 x 50	3 p14	chromosome 3 open reading frame 14 [Source:HGNC Symbol;Acc:HGNC:25024]	
ROCK2	0,57	12,17	0	50 x 47	2 p25	Rho associated coiled-coil containing protein kinase 2 [Source:HGNC Symbol;Acc:HGNC:10252]	
MLH1	0,57	12,15	0	48 x 49	3 p22	mutL homolog 1 [Source:HGNC Symbol;Acc:HGNC:7127]	
CALM2	0,57	12,11	0	50 x 45	2 p21	calmodulin 2 (phosphorylase kinase, delta) [Source:HGNC Symbol;Acc:HGNC:1445]	
METTL21B	0,57	12,07	0	50 x 47	12 q14	methyltransferase like 21B [Source:HGNC Symbol;Acc:HGNC:24936]	
ALCAM	0,57	11,99	0	48 x 50	3 q13	activated leukocyte cell adhesion molecule [Source:HGNC Symbol;Acc:HGNC:400]	
SLC43A3	0,57	11,99	0	48 x 50	11 q12	solute carrier family 43 member 3 [Source:HGNC Symbol;Acc:HGNC:17466]	
COL12A1	0,56	11,95	0	48 x 45	6 q14	collagen type XII alpha 1 chain [Source:HGNC Symbol;Acc:HGNC:2188]	
RCN3	0,56	11,93	0	47 x 46	19 q13	reticulocalbin 3 [Source:HGNC Symbol;Acc:HGNC:21145]	
CLIC1	0,56	11,90	0	46 x 50	6 p21	chloride intracellular channel 1 [Source:HGNC Symbol;Acc:HGNC:2062]	
IFNGR2	0,56	11,86	0	48 x 49	CHR_HSCHR21_4_C	interferon gamma receptor 2 (interferon gamma transducer 1) [Source:HGNC Symbol;Acc:HGNC:5440]	
MMP16	0,56	11,86	0	47 x 50	8 q21	matrix metallopeptidase 16 [Source:HGNC Symbol;Acc:HGNC:7162]	
BEX2	0,56	11,84	0	48 x 47	X q22	brain expressed X-linked 2 [Source:HGNC Symbol;Acc:HGNC:30933]	
PTRF	0,56	11,84	0	46 x 50	17 q21	polymerase I and transcript release factor [Source:HGNC Symbol;Acc:HGNC:9688]	
SHC4	0,56	11,82	0	47 x 50	15 q21	SHC adaptor protein 4 [Source:HGNC Symbol;Acc:HGNC:16743]	
CDH2	0,56	11,77	0	47 x 50	18 q12	cadherin 2 [Source:HGNC Symbol;Acc:HGNC:1759]	
LPXN	0,56	11,75	0	50 x 48	11 q12	leupaxin [Source:HGNC Symbol;Acc:HGNC:14061]	
COTL1	0,56	11,73	0	49 x 48	16 q24	coactosin like F-actin binding protein 1 [Source:HGNC Symbol;Acc:HGNC:18304]	
COMT	0,56	11,72	0	50 x 50	22 q11	catechol-O-methyltransferase [Source:HGNC Symbol;Acc:HGNC:2228]	
KCNN4	0,55	11,64	0	49 x 45	19 q13	potassium calcium-activated channel subfamily N member 4 [Source:HGNC Symbol;Acc:HGNC:6293]	
CTGF	0,55	11,62	0	50 x 47	6 q23	connective tissue growth factor [Source:HGNC Symbol;Acc:HGNC:2500]	
S100A2	0,55	11,59	0	50 x 48	1 q21	S100 calcium binding protein A2 [Source:HGNC Symbol;Acc:HGNC:10492]	
MRPL51	0,55	11,59	0	50 x 45	12 p13	mitochondrial ribosomal protein L51 [Source:HGNC Symbol;Acc:HGNC:14044]	
B4GALT1	0,55	11,55	0	46 x 50	9 p21	beta-1,4-galactosyltransferase 1 [Source:HGNC Symbol;Acc:HGNC:924]	
FOSL1	0,55	11,53	0	47 x 49	11 q13	FOS like 1, AP-1 transcription factor subunit [Source:HGNC Symbol;Acc:HGNC:13718]	
FGF5	0,55	11,52	0	48 x 45	4 q21	fibroblast growth factor 5 [Source:HGNC Symbol;Acc:HGNC:3683]	
G6PD	0,55	11,52	0	49 x 48	X q28	glucose-6-phosphate dehydrogenase [Source:HGNC Symbol;Acc:HGNC:4057]	
SLC14A1	0,55	11,50	0	50 x 47	18 q12	solute carrier family 14 member 1 (Kidd blood group) [Source:HGNC Symbol;Acc:HGNC:10918]	
SORBS2	0,55	11,49	0	48 x 50	4 q35	sorbin and SH3 domain containing 2 [Source:HGNC Symbol;Acc:HGNC:24098]	


PEG10	0,55	11,44	0	50 x 46	7 q21	paternally expressed 10 [Source:HGNC Symbol;Acc:HGNC:14005]	
RBMS2	0,55	11,38	0	50 x 45	12 q13	RNA binding motif single stranded interacting protein 2 [Source:HGNC Symbol;Acc:HGNC:9909]	
CPA4	0,55	11,37	0	49 x 46	7 q32	carboxypeptidase A4 [Source:HGNC Symbol;Acc:HGNC:15740]	
TNIK	0,54	11,34	0	47 x 50	3 q26	TRAF2 and NCK interacting kinase [Source:HGNC Symbol;Acc:HGNC:30765]	
SERPINE1	0,54	11,34	0	50 x 45	7 q22	serpin family E member 1 [Source:HGNC Symbol;Acc:HGNC:8583]	
CD59	0,54	11,31	0	47 x 50	11 p13	CD59 molecule [Source:HGNC Symbol;Acc:HGNC:1689]	
ZNF804A	0,54	11,28	0	48 x 45	2 q32	zinc finger protein 804A [Source:HGNC Symbol;Acc:HGNC:21711]	
KLF9	0,54	11,14	0	47 x 50	9 q21	Kruppel like factor 9 [Source:HGNC Symbol;Acc:HGNC:1123]	
CRIP2	0,54	11,10	0	47 x 50	14 q32	cysteine rich protein 2 [Source:HGNC Symbol;Acc:HGNC:2361]	
FGD4	0,54	11,06	0	49 x 50	12 p11	FYVE, RhoGEF and PH domain containing 4 [Source:HGNC Symbol;Acc:HGNC:19125]	
NFE2L3	0,53	11,06	0	49 x 46	7 p15	nuclear factor, erythroid 2 like 3 [Source:HGNC Symbol;Acc:HGNC:7783]	
NAV3	0,53	11,05	0	50 x 47	12 q21	neuron navigator 3 [Source:HGNC Symbol;Acc:HGNC:15998]	
SCG5	0,53	11,03	0	50 x 45	CHR_HSCHR15_6_C	secretogranin V [Source:HGNC Symbol;Acc:HGNC:10816]	
AZGP1	0,53	10,99	0	47 x 47	7 q22	alpha-2-glycoprotein 1, zinc-binding [Source:HGNC Symbol;Acc:HGNC:910]	
DST	0,53	10,96	0	47 x 49	6 p12	dystonin [Source:HGNC Symbol;Acc:HGNC:1090]	
ZFHX3	0,53	10,93	0	48 x 45	16 q22	zinc finger homeobox 3 [Source:HGNC Symbol;Acc:HGNC:777]	
ASAP2	0,53	10,92	0	48 x 48	2 p25	ArfGAP with SH3 domain, ankyrin repeat and PH domain 2 [Source:HGNC Symbol;Acc:HGNC:2721]	
WWTR1	0,53	10,90	0	46 x 50	3 q25	WW domain containing transcription regulator 1 [Source:HGNC Symbol;Acc:HGNC:24042]	
ITGB1	0,53	10,89	0	48 x 48	10 p11	integrin subunit beta 1 [Source:HGNC Symbol;Acc:HGNC:6153]	
CCDC80	0,53	10,87	0	49 x 47	3 q13	coiled-coil domain containing 80 [Source:HGNC Symbol;Acc:HGNC:30649]	
ARHGDIB	0,53	10,81	0	47 x 50	12 p12	Rho GDP dissociation inhibitor beta [Source:HGNC Symbol;Acc:HGNC:679]	
PTX3	0,53	10,80	0	49 x 46	3 q25	pentraxin 3 [Source:HGNC Symbol;Acc:HGNC:9692]	
REEP3	0,52	10,73	0	50 x 47	10 q21	receptor accessory protein 3 [Source:HGNC Symbol;Acc:HGNC:23711]	
FMN2	0,52	10,72	0	50 x 46	1 q43	formin 2 [Source:HGNC Symbol;Acc:HGNC:14074]	
S100A13	0,52	10,71	0	50 x 50	1 q21	S100 calcium binding protein A13 [Source:HGNC Symbol;Acc:HGNC:10490]	
TMEM156	0,52	10,67	0	50 x 45	4 p14	transmembrane protein 156 [Source:HGNC Symbol;Acc:HGNC:26260]	
ITGA2	0,52	10,62	0	46 x 49	5 q11	integrin subunit alpha 2 [Source:HGNC Symbol;Acc:HGNC:6137]	
AMOTL1	0,52	10,60	0	46 x 50	11 q21	angiomotin like 1 [Source:HGNC Symbol;Acc:HGNC:17811]	
ZNF518A	0,52	10,59	0	47 x 46	10 q24	zinc finger protein 518A [Source:HGNC Symbol;Acc:HGNC:29009]	
HSD17B12	0,52	10,57	0	48 x 50	11 p11	hydroxysteroid 17-beta dehydrogenase 12 [Source:HGNC Symbol;Acc:HGNC:18646]	
TMEM171	0,52	10,54	0	47 x 50	5 q13	transmembrane protein 171 [Source:HGNC Symbol;Acc:HGNC:27031]	
TNFAIP2	0,52	10,51	0	48 x 45	14 q32	TNF alpha induced protein 2 [Source:HGNC Symbol;Acc:HGNC:11895]	
CUL4B	0,51	10,31	0	50 x 48	X q24	cullin 4B [Source:HGNC Symbol;Acc:HGNC:2555]	


FAM208B	0,51	10,31	0	50 x 47	10 p15	family with sequence similarity 208 member B [Source:HGNC Symbol;Acc:HGNC:23484]	
RECQL	0,51	10,30	0	50 x 47	12 p12	RecQ like helicase [Source:HGNC Symbol;Acc:HGNC:9948]	
ADAM9	0,51	10,25	0	47 x 50	8 p11	ADAM metallopeptidase domain 9 [Source:HGNC Symbol;Acc:HGNC:216]	
CDH19	0,51	10,24	0	48 x 50	18 q22	cadherin 19 [Source:HGNC Symbol;Acc:HGNC:1758]	
WIPF1	0,51	10,23	0	47 x 47	2 q31	WAS/WASL interacting protein family member 1 [Source:HGNC Symbol;Acc:HGNC:12736]	
RBX1	0,50	10,22	0	50 x 44	22 q13	ring-box 1 [Source:HGNC Symbol;Acc:HGNC:9928]	
MTPAP	0,50	10,21	0	50 x 48	10 p11	mitochondrial poly(A) polymerase [Source:HGNC Symbol;Acc:HGNC:25532]	
QSOX1	0,50	10,18	0	49 x 47	1 q25	quiescin sulfhydryl oxidase 1 [Source:HGNC Symbol;Acc:HGNC:9756]	
IGF2BP2	0,50	10,16	0	46 x 50	3 q27	insulin like growth factor 2 mRNA binding protein 2 [Source:HGNC Symbol;Acc:HGNC:28867]	
SMTN	0,50	10,15	0	50 x 46	22 q12	smoothelin [Source:HGNC Symbol;Acc:HGNC:11126]	
ITGB5	0,50	10,15	0	50 x 47	3 q21	integrin subunit beta 5 [Source:HGNC Symbol;Acc:HGNC:6160]	
MAP1B	0,50	10,11	0	47 x 50	5 q13	microtubule associated protein 1B [Source:HGNC Symbol;Acc:HGNC:6836]	
AK5	0,50	10,11	0	47 x 47	1 p31	adenylate kinase 5 [Source:HGNC Symbol;Acc:HGNC:365]	
GXYLT2	0,50	10,06	0	50 x 45	3 p13	glucoside xylosyltransferase 2 [Source:HGNC Symbol;Acc:HGNC:33383]	
HLA-DPB1	0,50	10,00	0	48 x 46	CHR_HSCHR6_MHC	major histocompatibility complex, class II, DP beta 1 [Source:HGNC Symbol;Acc:HGNC:4940]	
SPATS2L	0,50	9,95	0	50 x 47	2 q33	spermatogenesis associated serine rich 2 like [Source:HGNC Symbol;Acc:HGNC:24574]	
NEGR1	0,50	9,95	0	47 x 46	1 p31	neuronal growth regulator 1 [Source:HGNC Symbol;Acc:HGNC:17302]	
MT1X	0,49	9,92	0	50 x 46	16 q13	metallothionein 1X [Source:HGNC Symbol;Acc:HGNC:7405]	
RHOC	0,49	9,91	0	50 x 46	1 p13	ras homolog family member C [Source:HGNC Symbol;Acc:HGNC:669]	
GRIK2	0,49	9,89	0	47 x 50	6 q16	glutamate ionotropic receptor kainate type subunit 2 [Source:HGNC Symbol;Acc:HGNC:4580]	
RTN4	0,49	9,89	0	50 x 46	2 p16	reticulon 4 [Source:HGNC Symbol;Acc:HGNC:14085]	
CNN2	0,49	9,88	0	48 x 50	19 p13	calponin 2 [Source:HGNC Symbol;Acc:HGNC:2156]	
UBE2L3	0,49	9,84	0	47 x 47	22 q11	ubiquitin conjugating enzyme E2 L3 [Source:HGNC Symbol;Acc:HGNC:12488]	
ZMYND8	0,49	9,78	0	50 x 47	20 q13	zinc finger MYND-type containing 8 [Source:HGNC Symbol;Acc:HGNC:9397]	
BCL2A1	0,49	9,76	0	46 x 49	15 q25	BCL2 related protein A1 [Source:HGNC Symbol;Acc:HGNC:991]	
FAT1	0,49	9,75	0	47 x 50	4 q35	FAT atypical cadherin 1 [Source:HGNC Symbol;Acc:HGNC:3595]	
LMO7	0,49	9,72	0	50 x 49	13 q22	LIM domain 7 [Source:HGNC Symbol;Acc:HGNC:6646]	
SKIL	0,49	9,72	0	49 x 46	3 q26	SKI-like proto-oncogene [Source:HGNC Symbol;Acc:HGNC:10897]	
SLFN12	0,48	9,58	0	48 x 47	17 q12	schlafen family member 12 [Source:HGNC Symbol;Acc:HGNC:25500]	
CCDC25	0,48	9,56	0	46 x 50	8 p21	coiled-coil domain containing 25 [Source:HGNC Symbol;Acc:HGNC:25591]	
ATXN1	0,48	9,55	0	46 x 50	6 p22	ataxin 1 [Source:HGNC Symbol;Acc:HGNC:10548]	
NMI	0,48	9,55	0	48 x 45	2 q23	N-myc and STAT interactor [Source:HGNC Symbol;Acc:HGNC:7854]	
TRAPPC4	0,48	9,48	0	48 x 50	CHR_HG2217_PAT	trafficking protein particle complex 4 [Source:HGNC Symbol;Acc:HGNC:19943]	


TNS3	0,48	9,48	0	47 x 49	7 p12	tensin 3 [Source:HGNC Symbol;Acc:HGNC:21616]	
NRAS	0,48	9,44	0	50 x 46	1 p13	neuroblastoma RAS viral oncogene homolog [Source:HGNC Symbol;Acc:HGNC:7989]	
SERPINB8	0,47	9,42	0	49 x 47	18 q22	serpin family B member 8 [Source:HGNC Symbol;Acc:HGNC:8952]	
LHFP	0,47	9,40	0	47 x 50	13 q14	lipoma HMGIC fusion partner [Source:HGNC Symbol;Acc:HGNC:6586]	
EML1	0,47	9,37	0	50 x 46	14 q32	echinoderm microtubule associated protein like 1 [Source:HGNC Symbol;Acc:HGNC:3330]	
ANKRD36	0,47	9,31	0	46 x 48	2 q11	ankyrin repeat domain 36 [Source:HGNC Symbol;Acc:HGNC:24079]	
UAP1	0,47	9,28	0	50 x 47	1 q23	UDP-N-acetylglucosamine pyrophosphorylase 1 [Source:HGNC Symbol;Acc:HGNC:12457]	
RBMS3	0,47	9,21	0	50 x 50	3 p24	RNA binding motif single stranded interacting protein 3 [Source:HGNC Symbol;Acc:HGNC:13427]	
UFD1L	0,47	9,19	0	49 x 49	22 q11	ubiquitin fusion degradation 1 like (yeast) [Source:HGNC Symbol;Acc:HGNC:12520]	
LAMB1	0,46	9,12	0	50 x 48	7 q31	laminin subunit beta 1 [Source:HGNC Symbol;Acc:HGNC:6486]	
HMGA1	0,46	9,11	0	47 x 46	6 p21	high mobility group AT-hook 1 [Source:HGNC Symbol;Acc:HGNC:5010]	
TNFRSF12A	0,46	9,07	0	48 x 50	16 p13	tumor necrosis factor receptor superfamily member 12A [Source:HGNC Symbol;Acc:HGNC:18152]	
SHC1	0,46	9,05	0	50 x 47	1 q21	SHC adaptor protein 1 [Source:HGNC Symbol;Acc:HGNC:10840]	
FARSB	0,46	9,04	0	50 x 46	2 q36	phenylalanyl-tRNA synthetase beta subunit [Source:HGNC Symbol;Acc:HGNC:17800]	
TNFRSF1A	0,46	9,03	0	49 x 47	12 p13	tumor necrosis factor receptor superfamily member 1A [Source:HGNC Symbol;Acc:HGNC:11916]	
BAG4	0,46	9,00	0	46 x 50	8 p11	BCL2 associated athanogene 4 [Source:HGNC Symbol;Acc:HGNC:940]	
FEZ2	0,46	9,00	0	50 x 47	2 p22	fasciculation and elongation protein zeta 2 [Source:HGNC Symbol;Acc:HGNC:3660]	
ACTN4	0,46	8,99	0	46 x 48	CHR_HG26_PATCH	actinin alpha 4 [Source:HGNC Symbol;Acc:HGNC:166]	
FBXO18	0,46	8,99	0	50 x 47	10 p15	F-box protein, helicase, 18 [Source:HGNC Symbol;Acc:HGNC:13620]	
ENO2	0,46	8,98	0	47 x 46	12 p13	enolase 2 [Source:HGNC Symbol;Acc:HGNC:3353]	
DDR2	0,46	8,96	0	47 x 50	1 q23	discoidin domain receptor tyrosine kinase 2 [Source:HGNC Symbol;Acc:HGNC:2731]	
CYBRD1	0,46	8,96	0	47 x 47	2 q31	cytochrome b reductase 1 [Source:HGNC Symbol;Acc:HGNC:20797]	
PSMD5	0,46	8,95	0	46 x 50	9 q33	proteasome 26S subunit, non-ATPase 5 [Source:HGNC Symbol;Acc:HGNC:9563]	
FNDC3B	0,46	8,94	0	48 x 47	3 q26	fibronectin type III domain containing 3B [Source:HGNC Symbol;Acc:HGNC:24670]	
OSBPL10	0,45	8,92	0	46 x 50	3 p22	oxysterol binding protein like 10 [Source:HGNC Symbol;Acc:HGNC:16395]	
PRR4	0,45	8,89	0	50 x 46	12 p13	proline rich 4 (lacrimal) [Source:HGNC Symbol;Acc:HGNC:18020]	
ANTXR1	0,45	8,89	0	48 x 47	2 p13	anthrax toxin receptor 1 [Source:HGNC Symbol;Acc:HGNC:21014]	
C9orf3	0,45	8,88	0	48 x 46	9 q22	chromosome 9 open reading frame 3 [Source:HGNC Symbol;Acc:HGNC:1361]	
MACF1	0,45	8,86	0	50 x 46	1 p34	microtubule-actin crosslinking factor 1 [Source:HGNC Symbol;Acc:HGNC:13664]	
BCL6	0,45	8,83	0	46 x 50	3 q27	B-cell CLL/lymphoma 6 [Source:HGNC Symbol;Acc:HGNC:1001]	
RAP1GDS1	0,45	8,78	0	46 x 50	4 q23	Rap1 GTPase-GDP dissociation stimulator 1 [Source:HGNC Symbol;Acc:HGNC:9859]	
SNX24	0,45	8,77	0	47 x 50	5 q23	sorting nexin 24 [Source:HGNC Symbol;Acc:HGNC:21533]	
PDLIM2	0,45	8,77	0	47 x 49	8 p21	PDZ and LIM domain 2 [Source:HGNC Symbol;Acc:HGNC:13992]	


MGST1	0,45	8,76	0	50 x 44	12 p12	microsomal glutathione S-transferase 1 [Source:HGNC Symbol;Acc:HGNC:7061]	
ETV6	0,45	8,75	0	47 x 47	12 p13	ETS variant 6 [Source:HGNC Symbol;Acc:HGNC:3495]	
AAK1	0,45	8,73	1,11E-16	47 x 47	2 p13	AP2 associated kinase 1 [Source:HGNC Symbol;Acc:HGNC:19679]	
AHR	0,45	8,70	1,11E-16	46 x 50	7 p21	aryl hydrocarbon receptor [Source:HGNC Symbol;Acc:HGNC:348]	
KHDRBS3	0,45	8,68	1,11E-16	48 x 50	8 q24	KH RNA binding domain containing, signal transduction associated 3 [Source:HGNC Symbol;Acc:HGNC:18117]	
	0,44	8,68	1,11E-16	50 x 45			
ACTR2	0,44	8,67	1,11E-16	50 x 44	2 p14	ARP2 actin-related protein 2 homolog (yeast) [Source:HGNC Symbol;Acc:HGNC:169]	
DNAJC16	0,44	8,66	2,22E-16	48 x 50	1 p36	DnaJ heat shock protein family (Hsp40) member C16 [Source:HGNC Symbol;Acc:HGNC:29157]	
LAMA4	0,44	8,65	2,22E-16	46 x 50	6 q21	laminin subunit alpha 4 [Source:HGNC Symbol;Acc:HGNC:6484]	
CAPRIN2	0,44	8,59	2,22E-16	50 x 46	12 p11	caprin family member 2 [Source:HGNC Symbol;Acc:HGNC:21259]	
SNAPC1	0,44	8,57	2,22E-16	48 x 46	14 q23	small nuclear RNA activating complex polypeptide 1 [Source:HGNC Symbol;Acc:HGNC:11134]	
RCAN1	0,44	8,51	4,44E-16	50 x 46	21 q22	regulator of calcineurin 1 [Source:HGNC Symbol;Acc:HGNC:3040]	
TUBE1	0,44	8,48	4,44E-16	49 x 47	6 q21	tubulin epsilon 1 [Source:HGNC Symbol;Acc:HGNC:20775]	
RSU1	0,44	8,46	5,55E-16	48 x 50	10 p13	Ras suppressor protein 1 [Source:HGNC Symbol;Acc:HGNC:10464]	
SPRY2	0,43	8,43	6,66E-16	47 x 50	13 q31	sprouty RTK signaling antagonist 2 [Source:HGNC Symbol;Acc:HGNC:11270]	
TMEM45A	0,43	8,39	8,88E-16	50 x 47	3 q12	transmembrane protein 45A [Source:HGNC Symbol;Acc:HGNC:25480]	
CRKL	0,43	8,37	1,11E-15	47 x 47	22 q11	v-crk avian sarcoma virus CT10 oncogene homolog-like [Source:HGNC Symbol;Acc:HGNC:2363]	
ECM1	0,43	8,37	1,11E-15	46 x 50	1 q21	extracellular matrix protein 1 [Source:HGNC Symbol;Acc:HGNC:3153]	
NEK7	0,43	8,34	1,33E-15	50 x 45	1 q31	NIMA related kinase 7 [Source:HGNC Symbol;Acc:HGNC:13386]	
TPST1	0,43	8,31	1,67E-15	48 x 47	7 q11	tyrosylprotein sulfotransferase 1 [Source:HGNC Symbol;Acc:HGNC:12020]	
TSPYL1	0,43	8,22	2,89E-15	50 x 44	6 q22	TSPY-like 1 [Source:HGNC Symbol;Acc:HGNC:12382]	
ASNS	0,43	8,20	3,33E-15	50 x 47	7 q21	asparagine synthetase (glutamine-hydrolyzing) [Source:HGNC Symbol;Acc:HGNC:753]	
TMEM209	0,43	8,20	3,33E-15	46 x 50	7 q32	transmembrane protein 209 [Source:HGNC Symbol;Acc:HGNC:21898]	
STARD13	0,42	8,19	3,77E-15	50 x 45	13 q13	StAR related lipid transfer domain containing 13 [Source:HGNC Symbol;Acc:HGNC:19164]	
PRPS2	0,42	8,18	3,89E-15	48 x 47	X p22	phosphoribosyl pyrophosphate synthetase 2 [Source:HGNC Symbol;Acc:HGNC:9465]	
TCF7L2	0,42	8,16	4,44E-15	50 x 46	10 q25	transcription factor 7 like 2 [Source:HGNC Symbol;Acc:HGNC:11641]	
LEF1	0,42	8,15	4,66E-15	46 x 48	4 q25	lymphoid enhancer binding factor 1 [Source:HGNC Symbol;Acc:HGNC:6551]	
FGFBP2	0,42	8,15	4,88E-15	48 x 45	4 p15	fibroblast growth factor binding protein 2 [Source:HGNC Symbol;Acc:HGNC:29451]	
GPX4	0,42	8,14	5,11E-15	47 x 50	19 p13	glutathione peroxidase 4 [Source:HGNC Symbol;Acc:HGNC:4556]	
PORCN	0,42	8,11	6,00E-15	47 x 47	X p11	porcupine homolog (Drosophila) [Source:HGNC Symbol;Acc:HGNC:17652]	
GRIP1	0,42	8,10	6,66E-15	50 x 45	12 q14	glutamate receptor interacting protein 1 [Source:HGNC Symbol;Acc:HGNC:18708]	
GOLT1B	0,42	8,10	6,66E-15	50 x 46	12 p12	golgi transport 1B [Source:HGNC Symbol;Acc:HGNC:20175]	
TFPI	0,42	8,08	7,55E-15	50 x 48	2 q32	tissue factor pathway inhibitor [Source:HGNC Symbol;Acc:HGNC:11760]	


SLC20A1	0,42	8,05	9,55E-15	50 x 45	2 q14	solute carrier family 20 member 1 [Source:HGNC Symbol;Acc:HGNC:10946]	
ETV1	0,42	8,05	9,55E-15	46 x 50	7 p21	ETS variant 1 [Source:HGNC Symbol;Acc:HGNC:3490]	
PDGFA	0,42	8,01	1,27E-14	50 x 46	7 p22	platelet derived growth factor subunit A [Source:HGNC Symbol;Acc:HGNC:8799]	
SLC25A37	0,41	7,97	1,64E-14	46 x 50	8 p21	solute carrier family 25 member 37 [Source:HGNC Symbol;Acc:HGNC:29786]	
EMG1	0,41	7,90	2,51E-14	50 x 45	12 p13	EMG1, N1-specific pseudouridine methyltransferase [Source:HGNC Symbol;Acc:HGNC:16912]	
KRCC1	0,41	7,85	3,60E-14	50 x 45	2 p11	lysine rich coiled-coil 1 [Source:HGNC Symbol;Acc:HGNC:28039]	
FAM171B	0,41	7,84	3,82E-14	46 x 50	2 q32	family with sequence similarity 171 member B [Source:HGNC Symbol;Acc:HGNC:29412]	
MDM2	0,41	7,84	3,85E-14	49 x 45	12 q15	MDM2 proto-oncogene [Source:HGNC Symbol;Acc:HGNC:6973]	
RDH10	0,41	7,83	4,02E-14	50 x 46	8 q21	retinol dehydrogenase 10 (all-trans) [Source:HGNC Symbol;Acc:HGNC:19975]	
MRPL33	0,41	7,81	4,49E-14	50 x 44	2 p23	mitochondrial ribosomal protein L33 [Source:HGNC Symbol;Acc:HGNC:14487]	
TBC1D19	0,41	7,81	4,73E-14	46 x 50	4 p15	TBC1 domain family member 19 [Source:HGNC Symbol;Acc:HGNC:25624]	
PAM	0,41	7,79	5,32E-14	49 x 47	5 q21	peptidylglycine alpha-amidating monooxygenase [Source:HGNC Symbol;Acc:HGNC:8596]	
ADAMTS1	0,41	7,79	5,42E-14	47 x 48	21 q21	ADAM metallopeptidase with thrombospondin type 1 motif 1 [Source:HGNC Symbol;Acc:HGNC:217]	
SLC35F1	0,40	7,73	7,69E-14	46 x 50	6 q22	solute carrier family 35 member F1 [Source:HGNC Symbol;Acc:HGNC:21483]	
NEIL2	0,40	7,64	1,44E-13	46 x 50	8 p23	nei like DNA glycosylase 2 [Source:HGNC Symbol;Acc:HGNC:18956]	
STEAP1B	0,40	7,63	1,52E-13	50 x 45	7 p15	STEAP family member 1B [Source:HGNC Symbol;Acc:HGNC:41907]	
CCND1	0,40	7,63	1,53E-13	47 x 50	11 q13	cyclin D1 [Source:HGNC Symbol;Acc:HGNC:1582]	
BICC1	0,40	7,63	1,54E-13	46 x 50	10 q21	BicC family RNA binding protein 1 [Source:HGNC Symbol;Acc:HGNC:19351]	
LRP6	0,40	7,60	1,78E-13	49 x 45	CHR_HG1362_PAT	LDL receptor related protein 6 [Source:HGNC Symbol;Acc:HGNC:6698]	
ASF1A	0,40	7,58	2,07E-13	47 x 47	6 q22	anti-silencing function 1A histone chaperone [Source:HGNC Symbol;Acc:HGNC:20995]	
TIMM17B	0,39	7,48	3,92E-13	46 x 50	X p11	translocase of inner mitochondrial membrane 17 homolog B (yeast) [Source:HGNC Symbol;Acc:HGNC:17310]	
MYO1E	0,39	7,45	4,85E-13	48 x 47	15 q22	myosin IE [Source:HGNC Symbol;Acc:HGNC:7599]	
COMMD1	0,39	7,44	5,05E-13	48 x 47	2 p15	copper metabolism domain containing 1 [Source:HGNC Symbol;Acc:HGNC:23024]	
BAZ2A	0,39	7,44	5,14E-13	50 x 45	12 q13	bromodomain adjacent to zinc finger domain 2A [Source:HGNC Symbol;Acc:HGNC:962]	
ANKRD28	0,39	7,43	5,55E-13	47 x 47	3 p25	ankyrin repeat domain 28 [Source:HGNC Symbol;Acc:HGNC:29024]	
KDM5A	0,39	7,42	5,76E-13	50 x 45	12 p13	lysine demethylase 5A [Source:HGNC Symbol;Acc:HGNC:9886]	
RAPH1	0,39	7,39	6,99E-13	50 x 45	2 q33	Ras association (RalGDS/AF-6) and pleckstrin homology domains 1 [Source:HGNC Symbol;Acc:HGNC:14436]	
GORAB	0,39	7,39	7,13E-13	50 x 46	1 q24	golgin, RAB6 interacting [Source:HGNC Symbol;Acc:HGNC:25676]	
RAP2C	0,39	7,36	8,80E-13	47 x 48	X q26	RAP2C, member of RAS oncogene family [Source:HGNC Symbol;Acc:HGNC:21165]	
TSPAN31	0,39	7,33	1,02E-12	50 x 46	12 q14	tetraspanin 31 [Source:HGNC Symbol;Acc:HGNC:10539]	
DYNLT3	0,39	7,31	1,18E-12	50 x 44	X p11	dynein light chain Tctex-type 3 [Source:HGNC Symbol;Acc:HGNC:11694]	
UBE2G1	0,39	7,31	1,19E-12	48 x 46	17 p13	ubiquitin conjugating enzyme E2 G1 [Source:HGNC Symbol;Acc:HGNC:12482]	
P4HA1	0,38	7,27	1,49E-12	50 x 45	10 q22	prolyl 4-hydroxylase subunit alpha 1 [Source:HGNC Symbol;Acc:HGNC:8546]	


TLK1	0,38	7,26	1,61E-12	49 x 45	2 q31	tousled like kinase 1 [Source:HGNC Symbol;Acc:HGNC:11841]	
ETHE1	0,38	7,23	2,00E-12	46 x 50	19 q13	ETHE1, persulfide dioxygenase [Source:HGNC Symbol;Acc:HGNC:23287]	
LSM1	0,38	7,19	2,49E-12	46 x 50	8 p11	LSM1 homolog, mRNA degradation associated [Source:HGNC Symbol;Acc:HGNC:20472]	
IGF2BP3	0,38	7,19	2,49E-12	50 x 44	7 p15	insulin like growth factor 2 mRNA binding protein 3 [Source:HGNC Symbol;Acc:HGNC:28868]	
CHMP5	0,38	7,19	2,50E-12	46 x 49	9 p13	charged multivesicular body protein 5 [Source:HGNC Symbol;Acc:HGNC:26942]	
DDX6	0,38	7,19	2,55E-12	46 x 50	11 q23	DEAD-box helicase 6 [Source:HGNC Symbol;Acc:HGNC:2747]	
FRS2	0,38	7,17	2,79E-12	50 x 44	12 q15	fibroblast growth factor receptor substrate 2 [Source:HGNC Symbol;Acc:HGNC:16971]	
DTWD1	0,38	7,15	3,25E-12	50 x 46	15 q21	DTW domain containing 1 [Source:HGNC Symbol;Acc:HGNC:30926]	
TGOLN2	0,38	7,14	3,49E-12	50 x 44	2 p11	trans-golgi network protein 2 [Source:HGNC Symbol;Acc:HGNC:15450]	
DHDDS	0,38	7,10	4,28E-12	50 x 45	1 p36	dehydrodolichyl diphosphate synthase subunit [Source:HGNC Symbol;Acc:HGNC:20603]	
IRF2BP2	0,38	7,08	5,02E-12	47 x 46	1 q42	interferon regulatory factor 2 binding protein 2 [Source:HGNC Symbol;Acc:HGNC:21729]	
TTR	0,37	7,06	5,60E-12	47 x 46	18 q12	transthyretin [Source:HGNC Symbol;Acc:HGNC:12405]	
BCAS2	0,37	7,05	5,93E-12	50 x 46	1 p13	breast carcinoma amplified sequence 2 [Source:HGNC Symbol;Acc:HGNC:975]	
USP53	0,37	7,03	6,67E-12	50 x 44	4 q26	ubiquitin specific peptidase 53 [Source:HGNC Symbol;Acc:HGNC:29255]	
GPX8	0,37	7,02	7,21E-12	46 x 50	5 q11	glutathione peroxidase 8 (putative) [Source:HGNC Symbol;Acc:HGNC:33100]	
BPGM	0,37	6,99	8,55E-12	50 x 46	7 q33	bisphosphoglycerate mutase [Source:HGNC Symbol;Acc:HGNC:1093]	
C22orf29	0,37	6,98	9,19E-12	46 x 48	22 q11	chromosome 22 open reading frame 29 [Source:HGNC Symbol;Acc:HGNC:26112]	
GLS	0,37	6,97	9,54E-12	49 x 45	2 q32	glutaminase [Source:HGNC Symbol;Acc:HGNC:4331]	
ATP2B4	0,37	6,96	1,06E-11	48 x 45	1 q32	ATPase plasma membrane Ca2+ transporting 4 [Source:HGNC Symbol;Acc:HGNC:817]	
ASUN	0,37	6,93	1,25E-11	50 x 47	12 p11	asunder, spermatogenesis regulator [Source:HGNC Symbol;Acc:HGNC:20174]	
MPHOSPH10	0,37	6,93	1,27E-11	50 x 44	2 p13	M-phase phosphoprotein 10 [Source:HGNC Symbol;Acc:HGNC:7213]	
	0,37	6,92	1,34E-11	50 x 45			
ADK	0,37	6,86	1,91E-11	50 x 46	10 q22	adenosine kinase [Source:HGNC Symbol;Acc:HGNC:257]	
PFKP	0,36	6,82	2,42E-11	47 x 47	10 p15	phosphofructokinase, platelet [Source:HGNC Symbol;Acc:HGNC:8878]	
TES	0,36	6,79	2,93E-11	46 x 50	7 q31	testin LIM domain protein [Source:HGNC Symbol;Acc:HGNC:14620]	
PYGB	0,36	6,76	3,53E-11	50 x 45	20 p11	phosphorylase, glycogen; brain [Source:HGNC Symbol;Acc:HGNC:9723]	
IGF1R	0,36	6,76	3,60E-11	47 x 50	15 q26	insulin like growth factor 1 receptor [Source:HGNC Symbol;Acc:HGNC:5465]	
CPM	0,36	6,72	4,45E-11	46 x 50	12 q15	carboxypeptidase M [Source:HGNC Symbol;Acc:HGNC:2311]	
NOC3L	0,36	6,68	5,72E-11	50 x 44	10 q23	NOC3 like DNA replication regulator [Source:HGNC Symbol;Acc:HGNC:24034]	
PIGF	0,36	6,64	7,33E-11	50 x 45	2 p21	phosphatidylinositol glycan anchor biosynthesis class F [Source:HGNC Symbol;Acc:HGNC:8962]	
PHGDH	0,35	6,61	8,51E-11	50 x 45	1 p12	phosphoglycerate dehydrogenase [Source:HGNC Symbol;Acc:HGNC:8923]	
STYXL1	0,35	6,61	8,79E-11	50 x 46	7 q11	serine/threonine/tyrosine interacting-like 1 [Source:HGNC Symbol;Acc:HGNC:18165]	
SH2B3	0,35	6,59	9,41E-11	48 x 45	12 q24	SH2B adaptor protein 3 [Source:HGNC Symbol;Acc:HGNC:29605]	


ACVR2A	0,35	6,58	1,02E-10	50 x 45	2 q22	activin A receptor type 2A [Source:HGNC Symbol;Acc:HGNC:173]	
COPS7A	0,35	6,57	1,11E-10	49 x 46	12 p13	COP9 signalosome subunit 7A [Source:HGNC Symbol;Acc:HGNC:16758]	
MAGOHB	0,35	6,55	1,22E-10	50 x 45	12 p13	mago homolog B, exon junction complex core component [Source:HGNC Symbol;Acc:HGNC:25504]	
GFPT1	0,35	6,54	1,33E-10	50 x 44	2 p13	glutamine--fructose-6-phosphate transaminase 1 [Source:HGNC Symbol;Acc:HGNC:4241]	
EMP1	0,35	6,47	1,90E-10	50 x 45	12 p13	epithelial membrane protein 1 [Source:HGNC Symbol;Acc:HGNC:3333]	
COQ3	0,35	6,42	2,61E-10	50 x 45	6 q16	coenzyme Q3, methyltransferase [Source:HGNC Symbol;Acc:HGNC:18175]	
ZBTB8OS	0,34	6,42	2,62E-10	49 x 45	1 p35	zinc finger and BTB domain containing 8 opposite strand [Source:HGNC Symbol;Acc:HGNC:24094]	
HMGCL	0,34	6,41	2,80E-10	46 x 48	1 p36	3-hydroxymethyl-3-methylglutaryl-CoA lyase [Source:HGNC Symbol;Acc:HGNC:5005]	
FRMD5	0,34	6,36	3,71E-10	48 x 46	15 q15	FERM domain containing 5 [Source:HGNC Symbol;Acc:HGNC:28214]	
TIPARP	0,34	6,33	4,44E-10	46 x 50	3 q25	TCDD inducible poly(ADP-ribose) polymerase [Source:HGNC Symbol;Acc:HGNC:23696]	
FGFR1OP2	0,34	6,31	4,78E-10	50 x 44	12 p11	FGFR1 oncogene partner 2 [Source:HGNC Symbol;Acc:HGNC:23098]	
WDR3	0,34	6,28	5,68E-10	46 x 50	1 p12	WD repeat domain 3 [Source:HGNC Symbol;Acc:HGNC:12755]	
YPEL5	0,34	6,28	5,90E-10	48 x 46	2 p23	yippee like 5 [Source:HGNC Symbol;Acc:HGNC:18329]	
RASSF8	0,34	6,24	7,18E-10	50 x 44	12 p12	Ras association domain family member 8 [Source:HGNC Symbol;Acc:HGNC:13232]	
ANAPC13	0,34	6,24	7,46E-10	50 x 44	3 q22	anaphase promoting complex subunit 13 [Source:HGNC Symbol;Acc:HGNC:24540]	
MID1	0,33	6,20	9,15E-10	50 x 46	X p22	midline 1 [Source:HGNC Symbol;Acc:HGNC:7095]	
WDR75	0,33	6,18	1,04E-09	50 x 44	2 q32	WD repeat domain 75 [Source:HGNC Symbol;Acc:HGNC:25725]	
PCID2	0,33	6,18	1,05E-09	50 x 45	13 q34	PCI domain containing 2 [Source:HGNC Symbol;Acc:HGNC:25653]	
ACTN1	0,33	6,07	1,87E-09	47 x 46	14 q24	actinin alpha 1 [Source:HGNC Symbol;Acc:HGNC:163]	
CNEP1R1	0,33	6,06	2,06E-09	48 x 46	16 q12	CTD nuclear envelope phosphatase 1 regulatory subunit 1 [Source:HGNC Symbol;Acc:HGNC:26759]	
PPIL3	0,33	6,05	2,07E-09	50 x 45	2 q33	peptidylprolyl isomerase like 3 [Source:HGNC Symbol;Acc:HGNC:9262]	
TWISTNB	0,33	6,01	2,59E-09	48 x 46	7 p21	TWIST neighbor [Source:HGNC Symbol;Acc:HGNC:18027]	
C15orf38-AP3S2	0,32	5,99	2,98E-09	50 x 44	15 q26	C15orf38-AP3S2 readthrough [Source:HGNC Symbol;Acc:HGNC:38824]	
FAM200B	0,32	5,95	3,65E-09	50 x 45	4 p15	family with sequence similarity 200 member B [Source:HGNC Symbol;Acc:HGNC:27740]	
EMP2	0,32	5,94	3,88E-09	50 x 45	16 p13	epithelial membrane protein 2 [Source:HGNC Symbol;Acc:HGNC:3334]	
SEMA3A	0,32	5,92	4,43E-09	50 x 45	7 q21	semaphorin 3A [Source:HGNC Symbol;Acc:HGNC:10723]	
INO80D	0,32	5,91	4,50E-09	50 x 44	CHR_HSCHR2_6_C	INO80 complex subunit D [Source:HGNC Symbol;Acc:HGNC:25997]	
SP100	0,32	5,88	5,44E-09	47 x 46	2 q37	SP100 nuclear antigen [Source:HGNC Symbol;Acc:HGNC:11206]	
PAPOLG	0,31	5,70	1,39E-08	50 x 44	2 p16	poly(A) polymerase gamma [Source:HGNC Symbol;Acc:HGNC:14982]	
DFFA	0,31	5,70	1,39E-08	50 x 44	1 p36	DNA fragmentation factor subunit alpha [Source:HGNC Symbol;Acc:HGNC:2772]	
DCUN1D4	0,31	5,70	1,44E-08	50 x 45	4 q12	defective in cullin neddylation 1 domain containing 4 [Source:HGNC Symbol;Acc:HGNC:28998]	
NOL10	0,31	5,69	1,46E-08	48 x 45	2 p25	nucleolar protein 10 [Source:HGNC Symbol;Acc:HGNC:25862]	
DDAH1	0,31	5,68	1,55E-08	50 x 46	1 p22	dimethylarginine dimethylaminohydrolase 1 [Source:HGNC Symbol;Acc:HGNC:2715]	


EML4	0,31	5,68	1,60E-08	50 x 44	2 p21	echinoderm microtubule associated protein like 4 [Source:HGNC Symbol;Acc:HGNC:1316]	
CAB39	0,31	5,68	1,61E-08	50 x 44	2 q37	calcium binding protein 39 [Source:HGNC Symbol;Acc:HGNC:20292]	
CWC22	0,31	5,64	1,92E-08	50 x 44	2 q31	CWC22 homolog, spliceosome-associated protein [Source:HGNC Symbol;Acc:HGNC:29322]	
JMJD1C	0,31	5,59	2,47E-08	48 x 45	10 q21	jumonji domain containing 1C [Source:HGNC Symbol;Acc:HGNC:12313]	
SLC4A1AP	0,30	5,56	2,92E-08	50 x 44	2 p23	solute carrier family 4 member 1 adaptor protein [Source:HGNC Symbol;Acc:HGNC:13813]	
HERC4	0,28	5,15	2,31E-07	50 x 44	10 q21	HECT and RLD domain containing E3 ubiquitin protein ligase 4 [Source:HGNC Symbol;Acc:HGNC:24521]	
LUZP1	0,28	5,12	2,76E-07	48 x 45	1 p36	leucine zipper protein 1 [Source:HGNC Symbol;Acc:HGNC:14985]	
MOB4	0,27	4,96	5,82E-07	50 x 44	2 q33	MOB family member 4, phocein [Source:HGNC Symbol;Acc:HGNC:17261]	
METTL5	0,27	4,95	6,23E-07	50 x 44	2 q31	methyltransferase like 5 [Source:HGNC Symbol;Acc:HGNC:25006]	
AP3M1	0,27	4,82	1,13E-06	50 x 44	10 q22	adaptor related protein complex 3 mu 1 subunit [Source:HGNC Symbol;Acc:HGNC:569]	
NEDD4L	0,27	4,82	1,13E-06	50 x 44	18 q21	neural precursor cell expressed, developmentally down-regulated 4-like, E3 ubiquitin protein ligase [Source:HGNC Sym	
HSPE1-MOB4	0,26	4,78	1,34E-06	50 x 44	2 q33	HSPE1-MOB4 readthrough [Source:HGNC Symbol;Acc:HGNC:49184]	
THUMPD1	0,26	4,72	1,84E-06	50 x 44	16 p12	THUMP domain containing 1 [Source:HGNC Symbol;Acc:HGNC:23807]	


Genes upregulated in group3	
Symbol	correlation	->t.score	->p.value	Metagene	Chromosome	Description	
VTN	0,60	13,12	0	3 x 21	17 q11	vitronectin [Source:HGNC Symbol;Acc:HGNC:12724]	
PZP	0,59	12,92	0	4 x 22	12 p13	PZP, alpha-2-macroglobulin like [Source:HGNC Symbol;Acc:HGNC:9750]	
HIST2H4B	0,58	12,57	0	3 x 20	1 q21	histone cluster 2, H4b [Source:HGNC Symbol;Acc:HGNC:29607]	
POTEI	0,57	12,26	0	6 x 19	2 q21	POTE ankyrin domain family member I [Source:HGNC Symbol;Acc:HGNC:37093]	
HIST2H4A	0,57	12,18	0	3 x 20	1 q21	histone cluster 2, H4a [Source:HGNC Symbol;Acc:HGNC:4794]	
HIST2H2AA4	0,57	12,05	0	4 x 20	1 q21	histone cluster 2, H2aa4 [Source:HGNC Symbol;Acc:HGNC:29668]	
SPTA1	0,54	11,27	0	3 x 21	1 q23	spectrin alpha, erythrocytic 1 [Source:HGNC Symbol;Acc:HGNC:11272]	
A2M	0,54	11,27	0	2 x 21	12 p13	alpha-2-macroglobulin [Source:HGNC Symbol;Acc:HGNC:7]	
	0,54	11,07	0	3 x 21	11 q23	HSPB2-C11orf52 readthrough (NMD candidate) [Source:HGNC Symbol;Acc:HGNC:41996]	
LOC102724334	0,52	10,63	0	4 x 19	21 q22	H2B histone family member S (pseudogene) [Source:HGNC Symbol;Acc:HGNC:4762]	
PCDH20	0,50	10,08	0	3 x 21	13 q21	protocadherin 20 [Source:HGNC Symbol;Acc:HGNC:14257]	
HSPB2	0,50	10,06	0	3 x 22	11 q23	heat shock protein family B (small) member 2 [Source:HGNC Symbol;Acc:HGNC:5247]	
EPAS1	0,49	9,90	0	3 x 22	2 p21	endothelial PAS domain protein 1 [Source:HGNC Symbol;Acc:HGNC:3374]	
RAB6C	0,49	9,79	0	4 x 18	2 q21	RAB6C, member RAS oncogene family [Source:HGNC Symbol;Acc:HGNC:16525]	
DNAJC6	0,49	9,75	0	3 x 22	1 p31	DnaJ heat shock protein family (Hsp40) member C6 [Source:HGNC Symbol;Acc:HGNC:15469]	
NPIPA7	0,48	9,66	0	5 x 18	16 p13	nuclear pore complex interacting protein family member A7 [Source:HGNC Symbol;Acc:HGNC:41982]	
BOLA2	0,47	9,18	0	1 x 18	16 p11	bolA family member 2 [Source:HGNC Symbol;Acc:HGNC:29488]	
HSPB7	0,46	9,03	0	4 x 22	1 p36	heat shock protein family B (small) member 7 [Source:HGNC Symbol;Acc:HGNC:5249]	
POLR2J2	0,44	8,63	2,22E-16	1 x 21	7 q22	polymerase (RNA) II subunit J2 [Source:HGNC Symbol;Acc:HGNC:23208]	
EDIL3	0,44	8,52	3,33E-16	3 x 22	5 q14	EGF like repeats and discoidin domains 3 [Source:HGNC Symbol;Acc:HGNC:3173]	
KRTAP7-1	0,44	8,49	4,44E-16	3 x 20	21 q22	keratin associated protein 7-1 (gene/pseudogene) [Source:HGNC Symbol;Acc:HGNC:18934]	
PALMD	0,44	8,49	4,44E-16	2 x 20	1 p21	palmdelphin [Source:HGNC Symbol;Acc:HGNC:15846]	
PTPRM	0,43	8,27	2,22E-15	2 x 20	18 p11	protein tyrosine phosphatase, receptor type M [Source:HGNC Symbol;Acc:HGNC:9675]	
CBLB	0,42	8,17	4,22E-15	2 x 21	3 q13	Cbl proto-oncogene B [Source:HGNC Symbol;Acc:HGNC:1542]	
STAT2	0,41	7,89	2,69E-14	2 x 22	12 q13	signal transducer and activator of transcription 2 [Source:HGNC Symbol;Acc:HGNC:11363]	
MATN2	0,41	7,88	2,98E-14	2 x 21	8 q22	matrilin 2 [Source:HGNC Symbol;Acc:HGNC:6908]	
OLR1	0,40	7,61	1,69E-13	4 x 23	12 p13	oxidized low density lipoprotein receptor 1 [Source:HGNC Symbol;Acc:HGNC:8133]	
HIST1H2AC	0,39	7,47	4,19E-13	3 x 21	6 p22	histone cluster 1, H2ac [Source:HGNC Symbol;Acc:HGNC:4733]	
GPR37	0,39	7,46	4,52E-13	4 x 22	7 q31	G protein-coupled receptor 37 [Source:HGNC Symbol;Acc:HGNC:4494]	
LSP1	0,39	7,46	4,67E-13	6 x 20	11 p15	lymphocyte-specific protein 1 [Source:HGNC Symbol;Acc:HGNC:6707]	
HIST2H2BE	0,39	7,36	8,83E-13	2 x 22	1 q21	histone cluster 2, H2be [Source:HGNC Symbol;Acc:HGNC:4760]	


PKD2	0,39	7,35	9,44E-13	3 x 22	4 q22	polycystin 2, transient receptor potential cation channel [Source:HGNC Symbol;Acc:HGNC:9009]	
UBE2E2	0,38	7,15	3,21E-12	3 x 20	3 p24	ubiquitin conjugating enzyme E2 E2 [Source:HGNC Symbol;Acc:HGNC:12478]	
CDKN2B	0,38	7,09	4,72E-12	3 x 23	9 p21	cyclin dependent kinase inhibitor 2B [Source:HGNC Symbol;Acc:HGNC:1788]	
GFRA1	0,38	7,08	5,09E-12	5 x 24	10 q25	GDNF family receptor alpha 1 [Source:HGNC Symbol;Acc:HGNC:4243]	
MYL4	0,38	7,07	5,34E-12	4 x 23	17 q21	myosin light chain 4 [Source:HGNC Symbol;Acc:HGNC:7585]	
SLC40A1	0,37	7,04	6,43E-12	3 x 21	2 q32	solute carrier family 40 member 1 [Source:HGNC Symbol;Acc:HGNC:10909]	
ALDH3A2	0,37	6,99	8,93E-12	2 x 20	17 p11	aldehyde dehydrogenase 3 family member A2 [Source:HGNC Symbol;Acc:HGNC:403]	
MYOZ2	0,37	6,89	1,63E-11	4 x 22	4 q26	myozenin 2 [Source:HGNC Symbol;Acc:HGNC:1330]	
SAMD4A	0,37	6,86	1,86E-11	3 x 22	14 q22	sterile alpha motif domain containing 4A [Source:HGNC Symbol;Acc:HGNC:23023]	
CCDC144A	0,37	6,86	1,90E-11	2 x 22	17 p11	coiled-coil domain containing 144A [Source:HGNC Symbol;Acc:HGNC:29072]	
SSX4	0,36	6,83	2,24E-11	6 x 18	X p11	SSX family member 4 [Source:HGNC Symbol;Acc:HGNC:11338]	
DAPK1	0,36	6,83	2,29E-11	2 x 23	9 q21	death associated protein kinase 1 [Source:HGNC Symbol;Acc:HGNC:2674]	
SSX4B	0,36	6,82	2,46E-11	6 x 18	X p11	SSX family member 4B [Source:HGNC Symbol;Acc:HGNC:16880]	
ADRA2A	0,36	6,81	2,65E-11	1 x 21	10 q25	adrenoceptor alpha 2A [Source:HGNC Symbol;Acc:HGNC:281]	
CTHRC1	0,36	6,76	3,42E-11	4 x 22	8 q22	collagen triple helix repeat containing 1 [Source:HGNC Symbol;Acc:HGNC:18831]	
GAB1	0,36	6,75	3,70E-11	4 x 21	4 q31	GRB2 associated binding protein 1 [Source:HGNC Symbol;Acc:HGNC:4066]	
CBWD5	0,36	6,68	5,69E-11	1 x 18	9 q21	COBW domain containing 3 [Source:HGNC Symbol;Acc:HGNC:18519]	
RGS4	0,36	6,64	7,06E-11	3 x 21	1 q23	regulator of G-protein signaling 4 [Source:HGNC Symbol;Acc:HGNC:10000]	
IGFBP2	0,36	6,64	7,12E-11	4 x 22	2 q35	insulin like growth factor binding protein 2 [Source:HGNC Symbol;Acc:HGNC:5471]	
LAMB2	0,35	6,55	1,25E-10	3 x 20	3 p21	laminin subunit beta 2 [Source:HGNC Symbol;Acc:HGNC:6487]	
TUBB2B	0,35	6,50	1,67E-10	3 x 18	6 p25	tubulin beta 2B class IIb [Source:HGNC Symbol;Acc:HGNC:30829]	
ZNF839	0,35	6,49	1,69E-10	3 x 22	14 q32	zinc finger protein 839 [Source:HGNC Symbol;Acc:HGNC:20345]	
PBX1	0,35	6,42	2,54E-10	5 x 22	1 q23	PBX homeobox 1 [Source:HGNC Symbol;Acc:HGNC:8632]	
KITLG	0,34	6,41	2,82E-10	3 x 20	12 q21	KIT ligand [Source:HGNC Symbol;Acc:HGNC:6343]	
HIST1H2BF	0,34	6,31	4,81E-10	4 x 21	6 p22	histone cluster 1, H2bf [Source:HGNC Symbol;Acc:HGNC:4752]	
NPEPPS	0,34	6,31	4,96E-10	1 x 18	17 q21	aminopeptidase puromycin sensitive [Source:HGNC Symbol;Acc:HGNC:7900]	
RECK	0,33	6,21	8,83E-10	4 x 22	9 p13	reversion inducing cysteine rich protein with kazal motifs [Source:HGNC Symbol;Acc:HGNC:11345]	
SLIT2	0,33	6,20	9,18E-10	3 x 22	4 p15	slit guidance ligand 2 [Source:HGNC Symbol;Acc:HGNC:11086]	
LIN7A	0,33	6,20	9,36E-10	3 x 22	12 q21	lin-7 homolog A, crumbs cell polarity complex component [Source:HGNC Symbol;Acc:HGNC:17787]	
	0,33	6,19	9,68E-10	3 x 19			
FRY	0,33	6,13	1,40E-09	4 x 23	13 q13	FRY microtubule binding protein [Source:HGNC Symbol;Acc:HGNC:20367]	
LAMC1	0,33	6,10	1,59E-09	3 x 20	1 q25	laminin subunit gamma 1 [Source:HGNC Symbol;Acc:HGNC:6492]	
	0,33	6,09	1,72E-09	2 x 20			


HHIP	0,33	6,06	1,96E-09	4 x 22	4 q31	hedgehog interacting protein [Source:HGNC Symbol;Acc:HGNC:14866]	
DRAM1	0,33	6,02	2,44E-09	4 x 22	12 q23	DNA damage regulated autophagy modulator 1 [Source:HGNC Symbol;Acc:HGNC:25645]	
KIAA0922	0,32	5,99	2,93E-09	2 x 20	4 q31	KIAA0922 [Source:HGNC Symbol;Acc:HGNC:29146]	
HECTD4	0,32	5,99	2,98E-09	3 x 21	12 q24	HECT domain E3 ubiquitin protein ligase 4 [Source:HGNC Symbol;Acc:HGNC:26611]	
DCAF8	0,32	5,93	4,21E-09	1 x 22	1 q23	DDB1 and CUL4 associated factor 8 [Source:HGNC Symbol;Acc:HGNC:24891]	
TGIF1	0,32	5,91	4,67E-09	3 x 21	18 p11	TGFB induced factor homeobox 1 [Source:HGNC Symbol;Acc:HGNC:11776]	
MEGF9	0,32	5,86	6,03E-09	1 x 20	9 q33	multiple EGF like domains 9 [Source:HGNC Symbol;Acc:HGNC:3234]	
HIST1H2BC	0,32	5,82	7,28E-09	3 x 22	6 p22	histone cluster 1, H2bc [Source:HGNC Symbol;Acc:HGNC:4757]	
FGF7	0,32	5,81	7,77E-09	4 x 22	15 q21	fibroblast growth factor 7 [Source:HGNC Symbol;Acc:HGNC:3685]	
VGLL4	0,31	5,78	9,13E-09	2 x 21	3 p25	vestigial like family member 4 [Source:HGNC Symbol;Acc:HGNC:28966]	
	0,31	5,77	9,66E-09	1 x 18			
PRRC2B	0,31	5,74	1,15E-08	1 x 20	9 q34	proline rich coiled-coil 2B [Source:HGNC Symbol;Acc:HGNC:28121]	
CLTC	0,31	5,69	1,50E-08	1 x 21	17 q23	clathrin heavy chain [Source:HGNC Symbol;Acc:HGNC:2092]	
HDAC5	0,31	5,69	1,51E-08	2 x 22	17 q21	histone deacetylase 5 [Source:HGNC Symbol;Acc:HGNC:14068]	
SRPX	0,31	5,68	1,58E-08	4 x 21	X p11	sushi repeat containing protein, X-linked [Source:HGNC Symbol;Acc:HGNC:11309]	
CYTH3	0,31	5,67	1,66E-08	4 x 22	7 p22	cytohesin 3 [Source:HGNC Symbol;Acc:HGNC:9504]	
ATG9A	0,31	5,64	1,93E-08	1 x 18	2 q35	autophagy related 9A [Source:HGNC Symbol;Acc:HGNC:22408]	
HIST1H3D	0,31	5,60	2,39E-08	3 x 21	6 p22	histone cluster 1, H3d [Source:HGNC Symbol;Acc:HGNC:4767]	
FADS3	0,30	5,56	2,90E-08	3 x 22	11 q12	fatty acid desaturase 3 [Source:HGNC Symbol;Acc:HGNC:3576]	
OXTR	0,30	5,52	3,59E-08	5 x 23	3 p25	oxytocin receptor [Source:HGNC Symbol;Acc:HGNC:8529]	
CCDC51	0,30	5,46	4,82E-08	2 x 20	3 p21	coiled-coil domain containing 51 [Source:HGNC Symbol;Acc:HGNC:25714]	
RFTN1	0,30	5,46	4,94E-08	5 x 20	3 p24	raftlin, lipid raft linker 1 [Source:HGNC Symbol;Acc:HGNC:30278]	
FNDC1	0,30	5,46	4,96E-08	6 x 22	6 q25	fibronectin type III domain containing 1 [Source:HGNC Symbol;Acc:HGNC:21184]	
PLLP	0,30	5,46	4,97E-08	4 x 19	16 q13	plasmolipin [Source:HGNC Symbol;Acc:HGNC:18553]	
PDLIM3	0,30	5,45	5,29E-08	3 x 20	4 q35	PDZ and LIM domain 3 [Source:HGNC Symbol;Acc:HGNC:20767]	
PLCD3	0,30	5,44	5,51E-08	4 x 23	17 q21	phospholipase C delta 3 [Source:HGNC Symbol;Acc:HGNC:9061]	
	0,29	5,39	7,13E-08	6 x 23	1 p11	neuroblastoma breakpoint family member 8 [Source:HGNC Symbol;Acc:HGNC:31990]	
GZF1	0,29	5,35	8,74E-08	4 x 21	20 p11	GDNF inducible zinc finger protein 1 [Source:HGNC Symbol;Acc:HGNC:15808]	
DPYSL4	0,29	5,35	8,83E-08	1 x 24	10 q26	dihydropyrimidinase like 4 [Source:HGNC Symbol;Acc:HGNC:3016]	
NDRG1	0,29	5,34	9,15E-08	4 x 23	8 q24	N-myc downstream regulated 1 [Source:HGNC Symbol;Acc:HGNC:7679]	
EFNA1	0,29	5,34	9,25E-08	3 x 22	1 q22	ephrin A1 [Source:HGNC Symbol;Acc:HGNC:3221]	
CELSR2	0,29	5,29	1,14E-07	2 x 24	1 p13	cadherin EGF LAG seven-pass G-type receptor 2 [Source:HGNC Symbol;Acc:HGNC:3231]	
PTPRG	0,29	5,27	1,31E-07	2 x 22	3 p14	protein tyrosine phosphatase, receptor type G [Source:HGNC Symbol;Acc:HGNC:9671]	


KDM5B	0,29	5,26	1,34E-07	1 x 22	1 q32	lysine demethylase 5B [Source:HGNC Symbol;Acc:HGNC:18039]	
TXNIP	0,29	5,22	1,64E-07	4 x 22	1 q21	thioredoxin interacting protein [Source:HGNC Symbol;Acc:HGNC:16952]	
CUL5	0,29	5,21	1,71E-07	1 x 18	11 q22	cullin 5 [Source:HGNC Symbol;Acc:HGNC:2556]	
ABHD4	0,29	5,21	1,78E-07	4 x 20	14 q11	abhydrolase domain containing 4 [Source:HGNC Symbol;Acc:HGNC:20154]	
INPPL1	0,28	5,14	2,51E-07	1 x 19	11 q13	inositol polyphosphate phosphatase like 1 [Source:HGNC Symbol;Acc:HGNC:6080]	
AFF4	0,28	5,13	2,53E-07	1 x 20	5 q31	AF4/FMR2 family member 4 [Source:HGNC Symbol;Acc:HGNC:17869]	
PLXNB1	0,28	5,09	3,12E-07	3 x 22	3 p21	plexin B1 [Source:HGNC Symbol;Acc:HGNC:9103]	
NRIP1	0,28	5,09	3,16E-07	1 x 23	21 q21	nuclear receptor interacting protein 1 [Source:HGNC Symbol;Acc:HGNC:8001]	
SLC6A6	0,28	5,06	3,72E-07	5 x 22	3 p25	solute carrier family 6 member 6 [Source:HGNC Symbol;Acc:HGNC:11052]	
ACSS2	0,28	5,04	4,07E-07	1 x 19	20 q11	acyl-CoA synthetase short-chain family member 2 [Source:HGNC Symbol;Acc:HGNC:15814]	
B9D1	0,28	5,02	4,49E-07	1 x 21	17 p11	B9 domain containing 1 [Source:HGNC Symbol;Acc:HGNC:24123]	
ID4	0,28	5,01	4,59E-07	4 x 21	6 p22	inhibitor of DNA binding 4, HLH protein [Source:HGNC Symbol;Acc:HGNC:5363]	
SNURF	0,27	4,98	5,28E-07	2 x 20	15 q11	SNRPN upstream reading frame [Source:HGNC Symbol;Acc:HGNC:11171]	
DHRSX	0,27	4,97	5,72E-07	1 x 18	X p22	dehydrogenase/reductase X-linked [Source:HGNC Symbol;Acc:HGNC:18399]	
CDC26	0,27	4,94	6,49E-07	1 x 19	9 q32	cell division cycle 26 [Source:HGNC Symbol;Acc:HGNC:17839]	
NEDD9	0,27	4,94	6,52E-07	3 x 21	6 p24	neural precursor cell expressed, developmentally down-regulated 9 [Source:HGNC Symbol;Acc:HGNC:7733]	
SATB1	0,27	4,92	7,15E-07	3 x 22	3 p24	SATB homeobox 1 [Source:HGNC Symbol;Acc:HGNC:10541]	
NCOA5	0,27	4,90	7,76E-07	4 x 23	20 q13	nuclear receptor coactivator 5 [Source:HGNC Symbol;Acc:HGNC:15909]	
NAP1L5	0,27	4,89	8,28E-07	5 x 21	4 q22	nucleosome assembly protein 1 like 5 [Source:HGNC Symbol;Acc:HGNC:19968]	
HEG1	0,27	4,86	9,57E-07	3 x 23	3 q21	heart development protein with EGF like domains 1 [Source:HGNC Symbol;Acc:HGNC:29227]	
FOXO1	0,27	4,83	1,06E-06	3 x 23	13 q14	forkhead box O1 [Source:HGNC Symbol;Acc:HGNC:3819]	
ISYNA1	0,27	4,82	1,11E-06	1 x 17	19 p13	inositol-3-phosphate synthase 1 [Source:HGNC Symbol;Acc:HGNC:29821]	
SLC4A2	0,26	4,76	1,48E-06	1 x 18	7 q36	solute carrier family 4 member 2 [Source:HGNC Symbol;Acc:HGNC:11028]	
KLF13	0,26	4,76	1,53E-06	3 x 22	CHR_HSCHR1	Kruppel like factor 13 [Source:HGNC Symbol;Acc:HGNC:13672]	
SAV1	0,26	4,75	1,59E-06	4 x 22	14 q22	salvador family WW domain containing protein 1 [Source:HGNC Symbol;Acc:HGNC:17795]	
IGF2R	0,26	4,73	1,74E-06	2 x 19	6 q25	insulin like growth factor 2 receptor [Source:HGNC Symbol;Acc:HGNC:5467]	
TTC3	0,26	4,72	1,78E-06	3 x 21	21 q22	tetratricopeptide repeat domain 3 [Source:HGNC Symbol;Acc:HGNC:12393]	
GPR21	0,26	4,69	2,05E-06	2 x 21	9 q33	RAB GTPase activating protein 1 [Source:HGNC Symbol;Acc:HGNC:17155]	
ZNF106	0,26	4,68	2,21E-06	1 x 17	15 q15	zinc finger protein 106 [Source:HGNC Symbol;Acc:HGNC:12886]	
PIEZO2	0,26	4,67	2,22E-06	3 x 22	18 p11	piezo type mechanosensitive ion channel component 2 [Source:HGNC Symbol;Acc:HGNC:26270]	
TCEA2	0,26	4,67	2,28E-06	4 x 21	20 q13	transcription elongation factor A2 [Source:HGNC Symbol;Acc:HGNC:11614]	
MBOAT2	0,25	4,60	3,15E-06	4 x 21	2 p25	membrane bound O-acyltransferase domain containing 2 [Source:HGNC Symbol;Acc:HGNC:25193]	
CDK18	0,25	4,60	3,15E-06	5 x 24	1 q32	cyclin dependent kinase 18 [Source:HGNC Symbol;Acc:HGNC:8751]	


SMG5	0,25	4,56	3,68E-06	2 x 23	1 q22	SMG5, nonsense mediated mRNA decay factor [Source:HGNC Symbol;Acc:HGNC:24644]	
TUBB2A	0,25	4,55	3,92E-06	3 x 19	6 p25	tubulin beta 2A class IIa [Source:HGNC Symbol;Acc:HGNC:12412]	
CBWD5	0,25	4,52	4,46E-06	1 x 18	9 p11	COBW domain containing 7 [Source:HGNC Symbol;Acc:HGNC:31977]	
GOLPH3	0,25	4,50	4,77E-06	4 x 22	5 p13	golgi phosphoprotein 3 [Source:HGNC Symbol;Acc:HGNC:15452]	
TCP11L2	0,25	4,50	4,78E-06	6 x 23	12 q23	t-complex 11 like 2 [Source:HGNC Symbol;Acc:HGNC:28627]	
CTR9	0,25	4,50	4,83E-06	1 x 19	11 p15	CTR9 homolog, Paf1/RNA polymerase II complex component [Source:HGNC Symbol;Acc:HGNC:16850]	
SLC23A2	0,25	4,49	5,14E-06	3 x 22	20 p13	solute carrier family 23 member 2 [Source:HGNC Symbol;Acc:HGNC:10973]	
TUT1	0,25	4,48	5,29E-06	4 x 20	11 q12	terminal uridylyl transferase 1, U6 snRNA-specific [Source:HGNC Symbol;Acc:HGNC:26184]	
PRRG1	0,25	4,46	5,68E-06	3 x 21	X p21	proline rich and Gla domain 1 [Source:HGNC Symbol;Acc:HGNC:9469]	
CRTAC1	0,25	4,44	6,22E-06	5 x 19	10 q24	cartilage acidic protein 1 [Source:HGNC Symbol;Acc:HGNC:14882]	
CSAD	0,25	4,44	6,38E-06	3 x 23	12 q13	cysteine sulfinic acid decarboxylase [Source:HGNC Symbol;Acc:HGNC:18966]	
TCTN1	0,25	4,42	6,76E-06	1 x 18	12 q24	tectonic family member 1 [Source:HGNC Symbol;Acc:HGNC:26113]	
CTDSPL	0,24	4,39	7,84E-06	3 x 21	3 p22	CTD small phosphatase like [Source:HGNC Symbol;Acc:HGNC:16890]	
AXIN1	0,24	4,38	8,06E-06	1 x 23	16 p13	axin 1 [Source:HGNC Symbol;Acc:HGNC:903]	
CD276	0,24	4,37	8,45E-06	4 x 19	15 q24	CD276 molecule [Source:HGNC Symbol;Acc:HGNC:19137]	
L3MBTL4	0,24	4,37	8,62E-06	1 x 17	18 p11	l(3)mbt-like 4 (Drosophila) [Source:HGNC Symbol;Acc:HGNC:26677]	
MYCBP2	0,24	4,35	9,47E-06	3 x 19	13 q22	MYC binding protein 2, E3 ubiquitin protein ligase [Source:HGNC Symbol;Acc:HGNC:23386]	
EPN2	0,24	4,29	1,19E-05	5 x 19	17 p11	epsin 2 [Source:HGNC Symbol;Acc:HGNC:18639]	
SLC38A10	0,24	4,29	1,20E-05	1 x 22	17 q25	solute carrier family 38 member 10 [Source:HGNC Symbol;Acc:HGNC:28237]	
KCNJ2	0,24	4,27	1,30E-05	4 x 22	17 q24	potassium voltage-gated channel subfamily J member 2 [Source:HGNC Symbol;Acc:HGNC:6263]	
LGMN	0,24	4,26	1,35E-05	2 x 18	14 q32	legumain [Source:HGNC Symbol;Acc:HGNC:9472]	
MBTPS1	0,24	4,26	1,39E-05	3 x 22	16 q24	membrane bound transcription factor peptidase, site 1 [Source:HGNC Symbol;Acc:HGNC:15456]	
REV3L	0,24	4,25	1,44E-05	3 x 22	6 q21	REV3 like, DNA directed polymerase zeta catalytic subunit [Source:HGNC Symbol;Acc:HGNC:9968]	
CAPS2	0,24	4,24	1,46E-05	3 x 21	12 q21	calcyphosine 2 [Source:HGNC Symbol;Acc:HGNC:16471]	
CSGALNACT2	0,24	4,23	1,52E-05	3 x 21	10 q11	chondroitin sulfate N-acetylgalactosaminyltransferase 2 [Source:HGNC Symbol;Acc:HGNC:24292]	
L3HYPDH	0,24	4,23	1,53E-05	2 x 20	14 q23	trans-L-3-hydroxyproline dehydratase [Source:HGNC Symbol;Acc:HGNC:20488]	
CHMP1B	0,24	4,23	1,55E-05	1 x 21	18 p11	charged multivesicular body protein 1B [Source:HGNC Symbol;Acc:HGNC:24287]	
PIGS	0,24	4,22	1,59E-05	1 x 18	17 q11	phosphatidylinositol glycan anchor biosynthesis class S [Source:HGNC Symbol;Acc:HGNC:14937]	
ARHGEF1	0,23	4,22	1,62E-05	1 x 22	19 q13	Rho guanine nucleotide exchange factor 1 [Source:HGNC Symbol;Acc:HGNC:681]	
PDIA5	0,23	4,21	1,66E-05	3 x 21	3 q21	protein disulfide isomerase family A member 5 [Source:HGNC Symbol;Acc:HGNC:24811]	
WDR25	0,23	4,19	1,82E-05	4 x 22	14 q32	WD repeat domain 25 [Source:HGNC Symbol;Acc:HGNC:21064]	
ARFIP2	0,23	4,17	2,00E-05	1 x 18	11 p15	ADP ribosylation factor interacting protein 2 [Source:HGNC Symbol;Acc:HGNC:17160]	
RTN2	0,23	4,16	2,04E-05	6 x 23	19 q13	reticulon 2 [Source:HGNC Symbol;Acc:HGNC:10468]	


CLOCK	0,23	4,16	2,07E-05	3 x 22	4 q12	clock circadian regulator [Source:HGNC Symbol;Acc:HGNC:2082]	
CHD7	0,23	4,15	2,13E-05	2 x 22	8 q12	chromodomain helicase DNA binding protein 7 [Source:HGNC Symbol;Acc:HGNC:20626]	
TRIP6	0,23	4,11	2,51E-05	4 x 21	7 q22	thyroid hormone receptor interactor 6 [Source:HGNC Symbol;Acc:HGNC:12311]	
RARG	0,23	4,11	2,57E-05	5 x 22	12 q13	retinoic acid receptor gamma [Source:HGNC Symbol;Acc:HGNC:9866]	
SDF4	0,23	4,10	2,67E-05	6 x 22	1 p36	stromal cell derived factor 4 [Source:HGNC Symbol;Acc:HGNC:24188]	
ZNF350	0,23	4,08	2,82E-05	1 x 23	19 q13	zinc finger protein 350 [Source:HGNC Symbol;Acc:HGNC:16656]	
ADPGK	0,23	4,08	2,87E-05	2 x 21	15 q24	ADP dependent glucokinase [Source:HGNC Symbol;Acc:HGNC:25250]	
PCLO	0,22	4,03	3,58E-05	2 x 22	7 q21	piccolo presynaptic cytomatrix protein [Source:HGNC Symbol;Acc:HGNC:13406]	
RERE	0,22	4,02	3,72E-05	5 x 21	1 p36	arginine-glutamic acid dipeptide repeats [Source:HGNC Symbol;Acc:HGNC:9965]	
ABCB6	0,22	4,02	3,74E-05	1 x 19	2 q35	ATP binding cassette subfamily B member 6 (Langereis blood group) [Source:HGNC Symbol;Acc:HGNC:47]	
GPATCH3	0,22	4,00	3,93E-05	5 x 22	1 p36	G-patch domain containing 3 [Source:HGNC Symbol;Acc:HGNC:25720]	
	0,22	3,99	4,11E-05	2 x 19			
CTNND1	0,22	3,98	4,23E-05	2 x 22	11 q12	catenin delta 1 [Source:HGNC Symbol;Acc:HGNC:2515]	
UBE4B	0,22	3,98	4,34E-05	3 x 22	1 p36	ubiquitination factor E4B [Source:HGNC Symbol;Acc:HGNC:12500]	
RAI14	0,22	3,95	4,81E-05	2 x 23	5 p13	retinoic acid induced 14 [Source:HGNC Symbol;Acc:HGNC:14873]	
EXT1	0,22	3,95	4,85E-05	4 x 22	8 q24	exostosin glycosyltransferase 1 [Source:HGNC Symbol;Acc:HGNC:3512]	
	0,22	3,95	4,93E-05	1 x 19			
TNFSF9	0,22	3,94	4,99E-05	5 x 23	19 p13	tumor necrosis factor superfamily member 9 [Source:HGNC Symbol;Acc:HGNC:11939]	
TNFAIP1	0,22	3,93	5,31E-05	2 x 18	17 q11	TNF alpha induced protein 1 [Source:HGNC Symbol;Acc:HGNC:11894]	
CCL18	0,22	3,91	5,68E-05	1 x 19	CHR_HSCHR1	C-C motif chemokine ligand 18 [Source:HGNC Symbol;Acc:HGNC:10616]	
MIEF2	0,22	3,91	5,73E-05	6 x 18	17 p11	mitochondrial elongation factor 2 [Source:HGNC Symbol;Acc:HGNC:17920]	
TSPYL4	0,22	3,91	5,80E-05	5 x 24	6 q22	TSPY-like 4 [Source:HGNC Symbol;Acc:HGNC:21559]	
INF2	0,22	3,89	6,05E-05	5 x 19	14 q32	inverted formin, FH2 and WH2 domain containing [Source:HGNC Symbol;Acc:HGNC:23791]	
CCDC107	0,22	3,89	6,11E-05	1 x 17	9 p13	coiled-coil domain containing 107 [Source:HGNC Symbol;Acc:HGNC:28465]	
PRPF8	0,22	3,88	6,34E-05	1 x 18	17 p13	pre-mRNA processing factor 8 [Source:HGNC Symbol;Acc:HGNC:17340]	
MPRIP	0,22	3,87	6,61E-05	2 x 23	17 p11	myosin phosphatase Rho interacting protein [Source:HGNC Symbol;Acc:HGNC:30321]	
USP48	0,21	3,84	7,62E-05	3 x 20	1 p36	ubiquitin specific peptidase 48 [Source:HGNC Symbol;Acc:HGNC:18533]	
PARVA	0,21	3,83	7,89E-05	1 x 18	11 p15	parvin alpha [Source:HGNC Symbol;Acc:HGNC:14652]	
PCDH18	0,21	3,82	7,95E-05	4 x 24	4 q28	protocadherin 18 [Source:HGNC Symbol;Acc:HGNC:14268]	
BSDC1	0,21	3,80	8,77E-05	3 x 21	1 p35	BSD domain containing 1 [Source:HGNC Symbol;Acc:HGNC:25501]	
NPRL3	0,21	3,78	9,51E-05	5 x 18	16 p13	NPR3 like, GATOR1 complex subunit [Source:HGNC Symbol;Acc:HGNC:14124]	
ARG2	0,21	3,78	9,56E-05	1 x 21	14 q24	arginase 2 [Source:HGNC Symbol;Acc:HGNC:664]	
CLIP1	0,21	3,77	9,80E-05	4 x 22	12 q24	CAP-Gly domain containing linker protein 1 [Source:HGNC Symbol;Acc:HGNC:10461]	


DYNC2LI1	0,21	3,76	0,00010141	3 x 21	2 p21	dynein cytoplasmic 2 light intermediate chain 1 [Source:HGNC Symbol;Acc:HGNC:24595]	
LRP10	0,21	3,74	0,00011189	4 x 19	14 q11	LDL receptor related protein 10 [Source:HGNC Symbol;Acc:HGNC:14553]	
ATP2B1	0,21	3,72	0,00011771	1 x 19	12 q21	ATPase plasma membrane Ca2+ transporting 1 [Source:HGNC Symbol;Acc:HGNC:814]	
WDR26	0,21	3,71	0,00012112	1 x 21	1 q42	WD repeat domain 26 [Source:HGNC Symbol;Acc:HGNC:21208]	
KLHDC8B	0,21	3,70	0,00012918	6 x 18	3 p21	kelch domain containing 8B [Source:HGNC Symbol;Acc:HGNC:28557]	
SBF2	0,21	3,67	0,00014577	1 x 24	11 p15	SET binding factor 2 [Source:HGNC Symbol;Acc:HGNC:2135]	
AREL1	0,20	3,66	0,00015065	1 x 24	14 q24	apoptosis resistant E3 ubiquitin protein ligase 1 [Source:HGNC Symbol;Acc:HGNC:20363]	
HPS5	0,20	3,64	0,00015861	3 x 21	11 p15	HPS5, biogenesis of lysosomal organelles complex 2 subunit 2 [Source:HGNC Symbol;Acc:HGNC:17022]	
C3orf62	0,20	3,64	0,00016143	1 x 24	3 p21	chromosome 3 open reading frame 62 [Source:HGNC Symbol;Acc:HGNC:24771]	
EARS2	0,20	3,64	0,00016155	1 x 22	16 p12	glutamyl-tRNA synthetase 2, mitochondrial [Source:HGNC Symbol;Acc:HGNC:29419]	
ERBB2	0,20	3,63	0,00016388	5 x 24	17 q12	erb-b2 receptor tyrosine kinase 2 [Source:HGNC Symbol;Acc:HGNC:3430]	
C6orf226	0,20	3,63	0,00016394	1 x 18	6 p21	chromosome 6 open reading frame 226 [Source:HGNC Symbol;Acc:HGNC:34431]	
TMEM255A	0,20	3,63	0,00016457	6 x 23	X q24	transmembrane protein 255A [Source:HGNC Symbol;Acc:HGNC:26086]	
XPC	0,20	3,58	0,00020014	3 x 22	3 p25	XPC complex subunit, DNA damage recognition and repair factor [Source:HGNC Symbol;Acc:HGNC:12816]	
RAF1	0,20	3,57	0,0002087	1 x 18	3 p25	Raf-1 proto-oncogene, serine/threonine kinase [Source:HGNC Symbol;Acc:HGNC:9829]	
DUSP6	0,20	3,55	0,00022454	3 x 19	12 q21	dual specificity phosphatase 6 [Source:HGNC Symbol;Acc:HGNC:3072]	
DALRD3	0,20	3,54	0,0002279	1 x 17	3 p21	DALR anticodon binding domain containing 3 [Source:HGNC Symbol;Acc:HGNC:25536]	
SMIM14	0,20	3,54	0,00023092	2 x 21	4 p14	small integral membrane protein 14 [Source:HGNC Symbol;Acc:HGNC:27321]	
CYHR1	0,20	3,53	0,00023589	1 x 23	8 q24	cysteine and histidine rich 1 [Source:HGNC Symbol;Acc:HGNC:17806]	
RMND5B	0,20	3,53	0,00023806	1 x 19	5 q35	required for meiotic nuclear division 5 homolog B [Source:HGNC Symbol;Acc:HGNC:26181]	
COL4A3BP	0,20	3,53	0,00024033	1 x 21	5 q13	collagen type IV alpha 3 binding protein [Source:HGNC Symbol;Acc:HGNC:2205]	
MTMR12	0,20	3,51	0,00025954	1 x 18	5 p13	myotubularin related protein 12 [Source:HGNC Symbol;Acc:HGNC:18191]	
ZNF260	0,20	3,50	0,00026924	1 x 23	19 q13	zinc finger protein 260 [Source:HGNC Symbol;Acc:HGNC:13499]	
THOC5	0,19	3,46	0,00030663	4 x 21	22 q12	THO complex 5 [Source:HGNC Symbol;Acc:HGNC:19074]	
C6orf89	0,19	3,43	0,00034441	4 x 18	6 p21	chromosome 6 open reading frame 89 [Source:HGNC Symbol;Acc:HGNC:21114]	
FOXN3	0,19	3,41	0,00037324	1 x 24	14 q32	forkhead box N3 [Source:HGNC Symbol;Acc:HGNC:1928]	
B4GALT3	0,19	3,40	0,0003752	2 x 17	1 q23	beta-1,4-galactosyltransferase 3 [Source:HGNC Symbol;Acc:HGNC:926]	
MAP1S	0,19	3,40	0,00037782	1 x 20	19 p13	microtubule associated protein 1S [Source:HGNC Symbol;Acc:HGNC:15715]	
SNX29	0,19	3,40	0,00037955	5 x 21	16 p13	sorting nexin 29 [Source:HGNC Symbol;Acc:HGNC:30542]	
GGT7	0,19	3,37	0,00041732	6 x 18	20 q11	gamma-glutamyltransferase 7 [Source:HGNC Symbol;Acc:HGNC:4259]	
CDK9	0,19	3,34	0,00047831	1 x 17	9 q34	cyclin dependent kinase 9 [Source:HGNC Symbol;Acc:HGNC:1780]	
WDR46	0,19	3,32	0,00050471	1 x 22	CHR_HSCHR6	WD repeat domain 46 [Source:HGNC Symbol;Acc:HGNC:13923]	
IP6K1	0,19	3,30	0,00054617	6 x 21	3 p21	inositol hexakisphosphate kinase 1 [Source:HGNC Symbol;Acc:HGNC:18360]	


CYFIP1	0,18	3,29	0,0005669	2 x 18	15 q11	cytoplasmic FMR1 interacting protein 1 [Source:HGNC Symbol;Acc:HGNC:13759]	
RHOB	0,18	3,28	0,00058744	4 x 23	2 p24	ras homolog family member B [Source:HGNC Symbol;Acc:HGNC:668]	
FCHSD2	0,18	3,26	0,0006208	1 x 24	11 q13	FCH and double SH3 domains 2 [Source:HGNC Symbol;Acc:HGNC:29114]	
PINK1	0,18	3,24	0,00066716	6 x 22	1 p36	PTEN induced putative kinase 1 [Source:HGNC Symbol;Acc:HGNC:14581]	
WDR13	0,18	3,21	0,00074112	1 x 20	X p11	WD repeat domain 13 [Source:HGNC Symbol;Acc:HGNC:14352]	
AKT1	0,18	3,20	0,00075783	1 x 19	14 q32	AKT serine/threonine kinase 1 [Source:HGNC Symbol;Acc:HGNC:391]	
FBXL2	0,18	3,20	0,0007595	6 x 21	3 p22	F-box and leucine rich repeat protein 2 [Source:HGNC Symbol;Acc:HGNC:13598]	
CASC3	0,18	3,18	0,00080723	1 x 18	17 q21	cancer susceptibility candidate 3 [Source:HGNC Symbol;Acc:HGNC:17040]	
RBL2	0,18	3,18	0,00081285	5 x 20	16 q12	RB transcriptional corepressor like 2 [Source:HGNC Symbol;Acc:HGNC:9894]	
P2RX7	0,18	3,17	0,00083921	1 x 22	12 q24	purinergic receptor P2X 7 [Source:HGNC Symbol;Acc:HGNC:8537]	
GBE1	0,18	3,16	0,00085876	1 x 24	3 p12	glucan (1,4-alpha-), branching enzyme 1 [Source:HGNC Symbol;Acc:HGNC:4180]	
FBXO38	0,18	3,15	0,00090277	2 x 19	5 q32	F-box protein 38 [Source:HGNC Symbol;Acc:HGNC:28844]	
GNPTG	0,18	3,12	0,00100506	5 x 22	16 p13	N-acetylglucosamine-1-phosphate transferase gamma subunit [Source:HGNC Symbol;Acc:HGNC:23026]	
DNAJC2	0,17	3,05	0,00122791	1 x 24	7 q22	DnaJ heat shock protein family (Hsp40) member C2 [Source:HGNC Symbol;Acc:HGNC:13192]	
CA5B	0,17	3,03	0,00132312	1 x 24	X p22	carbonic anhydrase 5B [Source:HGNC Symbol;Acc:HGNC:1378]	
WDR7	0,17	3,03	0,00133139	2 x 17	18 q21	WD repeat domain 7 [Source:HGNC Symbol;Acc:HGNC:13490]	
ZNF227	0,17	3,03	0,0013394	4 x 20	19 q13	zinc finger protein 227 [Source:HGNC Symbol;Acc:HGNC:13020]	
MAF1	0,17	3,01	0,00140128	6 x 23	8 q24	MAF1 homolog, negative regulator of RNA polymerase III [Source:HGNC Symbol;Acc:HGNC:24966]	
TMEM104	0,17	3,00	0,00146628	4 x 24	17 q25	transmembrane protein 104 [Source:HGNC Symbol;Acc:HGNC:25984]	
TMOD3	0,17	2,99	0,00150625	2 x 23	15 q21	tropomodulin 3 [Source:HGNC Symbol;Acc:HGNC:11873]	
ACAA1	0,17	2,98	0,00153616	1 x 18	3 p22	acetyl-CoA acyltransferase 1 [Source:HGNC Symbol;Acc:HGNC:82]	
SEC24C	0,17	2,96	0,00164287	6 x 21	10 q22	SEC24 homolog C, COPII coat complex component [Source:HGNC Symbol;Acc:HGNC:10705]	
NFS1	0,17	2,95	0,00169049	4 x 19	20 q11	NFS1 cysteine desulfurase [Source:HGNC Symbol;Acc:HGNC:15910]	
IFT20	0,17	2,93	0,00181949	1 x 24	17 q11	intraflagellar transport 20 [Source:HGNC Symbol;Acc:HGNC:30989]	
TMBIM1	0,17	2,92	0,0018559	5 x 21	2 q35	transmembrane BAX inhibitor motif containing 1 [Source:HGNC Symbol;Acc:HGNC:23410]	
SLCO3A1	0,16	2,92	0,00188513	2 x 23	15 q26	solute carrier organic anion transporter family member 3A1 [Source:HGNC Symbol;Acc:HGNC:10952]	
PTK2	0,16	2,89	0,00203612	5 x 19	8 q24	protein tyrosine kinase 2 [Source:HGNC Symbol;Acc:HGNC:9611]	
YTHDF3	0,16	2,89	0,00208178	1 x 23	8 q12	YTH N6-methyladenosine RNA binding protein 3 [Source:HGNC Symbol;Acc:HGNC:26465]	
GTF3C1	0,16	2,83	0,00249631	3 x 24	16 p12	general transcription factor IIIC subunit 1 [Source:HGNC Symbol;Acc:HGNC:4664]	
DDX41	0,16	2,81	0,00264181	2 x 17	5 q35	DEAD-box helicase 41 [Source:HGNC Symbol;Acc:HGNC:18674]	
FNBP1L	0,16	2,81	0,00267432	5 x 23	1 p22	formin binding protein 1 like [Source:HGNC Symbol;Acc:HGNC:20851]	
KCTD3	0,16	2,80	0,00268412	2 x 24	1 q41	potassium channel tetramerization domain containing 3 [Source:HGNC Symbol;Acc:HGNC:21305]	
ACLY	0,16	2,80	0,0026913	1 x 18	17 q21	ATP citrate lyase [Source:HGNC Symbol;Acc:HGNC:115]	


FAF2	0,16	2,79	0,00278896	3 x 17	5 q35	Fas associated factor family member 2 [Source:HGNC Symbol;Acc:HGNC:24666]	
KDM5C	0,16	2,79	0,00282871	4 x 17	X p11	lysine demethylase 5C [Source:HGNC Symbol;Acc:HGNC:11114]	
SNAP47	0,16	2,78	0,00290328	1 x 17	1 q42	synaptosome associated protein 47 [Source:HGNC Symbol;Acc:HGNC:30669]	
IPO4	0,16	2,77	0,00296949	1 x 18	14 q12	importin 4 [Source:HGNC Symbol;Acc:HGNC:19426]	
HUWE1	0,16	2,75	0,00312435	2 x 19	X p11	HECT, UBA and WWE domain containing 1, E3 ubiquitin protein ligase [Source:HGNC Symbol;Acc:HGNC:30892]	
PACS2	0,15	2,73	0,00331455	1 x 24	14 q32	phosphofurin acidic cluster sorting protein 2 [Source:HGNC Symbol;Acc:HGNC:23794]	
ANXA11	0,15	2,72	0,00343885	5 x 19	10 q22	annexin A11 [Source:HGNC Symbol;Acc:HGNC:535]	
MAU2	0,15	2,71	0,00358685	1 x 24	19 p13	MAU2 sister chromatid cohesion factor [Source:HGNC Symbol;Acc:HGNC:29140]	
NR3C1	0,15	2,65	0,0042543	4 x 24	5 q31	nuclear receptor subfamily 3 group C member 1 [Source:HGNC Symbol;Acc:HGNC:7978]	
DUS4L	0,15	2,63	0,00454236	6 x 23	7 q22	dihydrouridine synthase 4 like [Source:HGNC Symbol;Acc:HGNC:21517]	
RCE1	0,15	2,61	0,00475276	6 x 19	11 q13	Ras converting CAAX endopeptidase 1 [Source:HGNC Symbol;Acc:HGNC:13721]	
POLR3E	0,15	2,61	0,00476133	1 x 22	16 p12	polymerase (RNA) III subunit E [Source:HGNC Symbol;Acc:HGNC:30347]	
IARS2	0,15	2,59	0,0049622	4 x 18	1 q41	isoleucyl-tRNA synthetase 2, mitochondrial [Source:HGNC Symbol;Acc:HGNC:29685]	
IFT27	0,15	2,59	0,00506818	1 x 17	22 q12	intraflagellar transport 27 [Source:HGNC Symbol;Acc:HGNC:18626]	
TMEM185A	0,14	2,55	0,00570277	5 x 19	X q28	transmembrane protein 185A [Source:HGNC Symbol;Acc:HGNC:17125]	
RAPGEF6	0,14	2,52	0,00605159	1 x 23	5 q31	Rap guanine nucleotide exchange factor 6 [Source:HGNC Symbol;Acc:HGNC:20655]	
MYO9B	0,14	2,50	0,00641381	6 x 22	19 p13	myosin IXB [Source:HGNC Symbol;Acc:HGNC:7609]	
MAPK14	0,14	2,48	0,00690233	1 x 24	6 p21	mitogen-activated protein kinase 14 [Source:HGNC Symbol;Acc:HGNC:6876]	
KDM6A	0,14	2,47	0,00699429	6 x 22	X p11	lysine demethylase 6A [Source:HGNC Symbol;Acc:HGNC:12637]	
PLA2G12A	0,14	2,44	0,00766518	1 x 17	4 q25	phospholipase A2 group XIIA [Source:HGNC Symbol;Acc:HGNC:18554]	
C18orf8	0,13	2,36	0,00934979	1 x 17	18 q11	chromosome 18 open reading frame 8 [Source:HGNC Symbol;Acc:HGNC:24326]	
ARF3	0,13	2,35	0,00967058	1 x 24	12 q13	ADP ribosylation factor 3 [Source:HGNC Symbol;Acc:HGNC:654]	
MUL1	0,13	2,34	0,00995168	1 x 19	1 p36	mitochondrial E3 ubiquitin protein ligase 1 [Source:HGNC Symbol;Acc:HGNC:25762]	
ZNF692	0,13	2,34	0,01004777	6 x 22	1 q44	zinc finger protein 692 [Source:HGNC Symbol;Acc:HGNC:26049]	
	0,13	2,33	0,01030624	5 x 22			
PRKCI	0,13	2,32	0,01054189	6 x 22	3 q26	protein kinase C iota [Source:HGNC Symbol;Acc:HGNC:9404]	
NUP133	0,13	2,30	0,0110398	1 x 24	1 q42	nucleoporin 133 [Source:HGNC Symbol;Acc:HGNC:18016]	
ZNF616	0,12	2,19	0,01448012	1 x 24	19 q13	zinc finger protein 616 [Source:HGNC Symbol;Acc:HGNC:28062]	
APH1A	0,12	2,05	0,02047934	1 x 17	1 q21	aph-1 homolog A, gamma-secretase subunit [Source:HGNC Symbol;Acc:HGNC:29509]	
CYB5R1	0,11	1,98	0,02403005	1 x 24	1 q32	cytochrome b5 reductase 1 [Source:HGNC Symbol;Acc:HGNC:13397]	
SMG7	0,11	1,96	0,02547392	5 x 18	1 q25	SMG7, nonsense mediated mRNA decay factor [Source:HGNC Symbol;Acc:HGNC:16792]	
CCZ1	0,11	1,93	0,02727402	3 x 18	7 p22	CCZ1 homolog, vacuolar protein trafficking and biogenesis associated [Source:HGNC Symbol;Acc:HGNC:21691]	
ZNF511	0,09	1,59	0,05682701	1 x 17	10 q26	zinc finger protein 511 [Source:HGNC Symbol;Acc:HGNC:28445]	


FAM102A	0,07	1,18	0,12035396	1 x 17	9 q34	family with sequence similarity 102 member A [Source:HGNC Symbol;Acc:HGNC:31419]	
CNPY3	0,06	1,09	0,13788315	1 x 17	6 p21	canopy FGF signaling regulator 3 [Source:HGNC Symbol;Acc:HGNC:11968]	
GINM1	0,04	0,65	0,25893033	1 x 17	6 q25	glycoprotein integral membrane 1 [Source:HGNC Symbol;Acc:HGNC:21074]	


Genes upregulated in proliferative groups	
Symbol	correlation	->t.score	->p.value	Metagene	Chromosome	Description	
HMGB2	0,86	28,99	0	50 x 1	4 q34	high mobility group box 2 [Source:HGNC Symbol;Acc:HGNC:5000]	
CDK1	0,83	25,66	0	50 x 1	10 q21	cyclin dependent kinase 1 [Source:HGNC Symbol;Acc:HGNC:1722]	
TOP2A	0,81	24,28	0	50 x 1	17 q21	topoisomerase (DNA) II alpha [Source:HGNC Symbol;Acc:HGNC:11989]	
NUSAP1	0,81	24,00	0	50 x 1	15 q15	nucleolar and spindle associated protein 1 [Source:HGNC Symbol;Acc:HGNC:18538]	
MAD2L1	0,79	22,15	0	50 x 1	4 q27	MAD2 mitotic arrest deficient-like 1 (yeast) [Source:HGNC Symbol;Acc:HGNC:6763]	
NCAPG	0,77	20,96	0	50 x 1	4 p15	non-SMC condensin I complex subunit G [Source:HGNC Symbol;Acc:HGNC:24304]	
RRM2	0,77	20,75	0	50 x 1	2 p25	ribonucleotide reductase regulatory subunit M2 [Source:HGNC Symbol;Acc:HGNC:10452]	
UBE2C	0,76	20,74	0	50 x 1	20 q13	ubiquitin conjugating enzyme E2 C [Source:HGNC Symbol;Acc:HGNC:15937]	
PRC1	0,76	20,38	0	50 x 1	15 q26	protein regulator of cytokinesis 1 [Source:HGNC Symbol;Acc:HGNC:9341]	
SKA1	0,75	20,04	0	50 x 1	18 q21	spindle and kinetochore associated complex subunit 1 [Source:HGNC Symbol;Acc:HGNC:28109]	
SPC25	0,75	20,00	0	50 x 1	2 q31	SPC25, NDC80 kinetochore complex component [Source:HGNC Symbol;Acc:HGNC:24031]	
RAD51AP1	0,75	19,66	0	50 x 1	12 p13	RAD51 associated protein 1 [Source:HGNC Symbol;Acc:HGNC:16956]	
KIAA0101	0,75	19,66	0	50 x 1	15 q22	KIAA0101 [Source:HGNC Symbol;Acc:HGNC:28961]	
MKI67	0,75	19,52	0	50 x 1	10 q26	marker of proliferation Ki-67 [Source:HGNC Symbol;Acc:HGNC:7107]	
FANCD2	0,74	19,15	0	50 x 1	3 p25	Fanconi anemia complementation group D2 [Source:HGNC Symbol;Acc:HGNC:3585]	
BIRC5	0,73	18,91	0	50 x 2	17 q25	baculoviral IAP repeat containing 5 [Source:HGNC Symbol;Acc:HGNC:593]	
NDC80	0,73	18,81	0	50 x 2	18 p11	NDC80, kinetochore complex component [Source:HGNC Symbol;Acc:HGNC:16909]	
UBE2T	0,73	18,81	0	50 x 1	1 q32	ubiquitin conjugating enzyme E2 T [Source:HGNC Symbol;Acc:HGNC:25009]	
SPC24	0,73	18,72	0	50 x 1	19 p13	SPC24, NDC80 kinetochore complex component [Source:HGNC Symbol;Acc:HGNC:26913]	
CENPF	0,73	18,43	0	50 x 2	1 q41	centromere protein F [Source:HGNC Symbol;Acc:HGNC:1857]	
AURKB	0,72	18,05	0	50 x 1	17 p13	aurora kinase B [Source:HGNC Symbol;Acc:HGNC:11390]	
HIST1H4C	0,72	18,03	0	50 x 1	6 p22	histone cluster 1, H4c [Source:HGNC Symbol;Acc:HGNC:4787]	
KIF2C	0,72	17,96	0	50 x 2	1 p34	kinesin family member 2C [Source:HGNC Symbol;Acc:HGNC:6393]	
TPX2	0,72	17,94	0	50 x 2	20 q11	TPX2, microtubule nucleation factor [Source:HGNC Symbol;Acc:HGNC:1249]	
TMPO	0,72	17,90	0	50 x 1	12 q23	thymopoietin [Source:HGNC Symbol;Acc:HGNC:11875]	
CKAP2L	0,72	17,90	0	50 x 1	2 q14	cytoskeleton associated protein 2 like [Source:HGNC Symbol;Acc:HGNC:26877]	
CDCA5	0,71	17,78	0	50 x 1	11 q13	cell division cycle associated 5 [Source:HGNC Symbol;Acc:HGNC:14626]	
BUB1B	0,71	17,68	0	50 x 1	15 q15	BUB1 mitotic checkpoint serine/threonine kinase B [Source:HGNC Symbol;Acc:HGNC:1149]	
KIF23	0,71	17,47	0	50 x 1	15 q23	kinesin family member 23 [Source:HGNC Symbol;Acc:HGNC:6392]	
TK1	0,71	17,41	0	50 x 1	17 q25	thymidine kinase 1 [Source:HGNC Symbol;Acc:HGNC:11830]	
ATAD2	0,71	17,41	0	49 x 1	8 q24	ATPase family, AAA domain containing 2 [Source:HGNC Symbol;Acc:HGNC:30123]	


SMC4	0,70	17,31	0	50 x 1	3 q25	structural maintenance of chromosomes 4 [Source:HGNC Symbol;Acc:HGNC:14013]	
ANLN	0,70	17,26	0	50 x 1	7 p14	anillin actin binding protein [Source:HGNC Symbol;Acc:HGNC:14082]	
CKS1B	0,70	17,23	0	50 x 1	1 q21	CDC28 protein kinase regulatory subunit 1B [Source:HGNC Symbol;Acc:HGNC:19083]	
SPAG5	0,70	17,13	0	50 x 1	17 q11	sperm associated antigen 5 [Source:HGNC Symbol;Acc:HGNC:13452]	
ASPM	0,69	16,77	0	50 x 2	1 q31	abnormal spindle microtubule assembly [Source:HGNC Symbol;Acc:HGNC:19048]	
TYMS	0,69	16,68	0	50 x 1	18 p11	thymidylate synthetase [Source:HGNC Symbol;Acc:HGNC:12441]	
PBK	0,69	16,67	0	50 x 2	8 p21	PDZ binding kinase [Source:HGNC Symbol;Acc:HGNC:18282]	
NUF2	0,69	16,60	0	50 x 2	1 q23	NUF2, NDC80 kinetochore complex component [Source:HGNC Symbol;Acc:HGNC:14621]	
CASC5	0,69	16,54	0	50 x 2	15 q15	cancer susceptibility candidate 5 [Source:HGNC Symbol;Acc:HGNC:24054]	
CENPK	0,69	16,52	0	50 x 1	5 q12	centromere protein K [Source:HGNC Symbol;Acc:HGNC:29479]	
FANCI	0,69	16,47	0	50 x 1	15 q26	Fanconi anemia complementation group I [Source:HGNC Symbol;Acc:HGNC:25568]	
BUB1	0,69	16,46	0	50 x 2	2 q13	BUB1 mitotic checkpoint serine/threonine kinase [Source:HGNC Symbol;Acc:HGNC:1148]	
ZWINT	0,69	16,42	0	50 x 1	10 q21	ZW10 interacting kinetochore protein [Source:HGNC Symbol;Acc:HGNC:13195]	
ESCO2	0,68	16,33	0	50 x 1	8 p21	establishment of sister chromatid cohesion N-acetyltransferase 2 [Source:HGNC Symbol;Acc:HGNC:27230]	
HJURP	0,68	16,23	0	50 x 1	2 q37	Holliday junction recognition protein [Source:HGNC Symbol;Acc:HGNC:25444]	
KIF20B	0,68	16,21	0	50 x 2	10 q23	kinesin family member 20B [Source:HGNC Symbol;Acc:HGNC:7212]	
SKA3	0,68	16,10	0	50 x 1	13 q12	spindle and kinetochore associated complex subunit 3 [Source:HGNC Symbol;Acc:HGNC:20262]	
BARD1	0,67	15,94	0	49 x 1	2 q35	BRCA1 associated RING domain 1 [Source:HGNC Symbol;Acc:HGNC:952]	
KIF15	0,67	15,72	0	50 x 1	CHR_HG2066_P	kinesin family member 15 [Source:HGNC Symbol;Acc:HGNC:17273]	
DEPDC1	0,67	15,67	0	50 x 2	1 p31	DEP domain containing 1 [Source:HGNC Symbol;Acc:HGNC:22949]	
MELK	0,67	15,60	0	50 x 1	9 p13	maternal embryonic leucine zipper kinase [Source:HGNC Symbol;Acc:HGNC:16870]	
CDCA3	0,66	15,22	0	50 x 2	12 p13	cell division cycle associated 3 [Source:HGNC Symbol;Acc:HGNC:14624]	
FAM111A	0,65	14,98	0	50 x 1	11 q12	family with sequence similarity 111 member A [Source:HGNC Symbol;Acc:HGNC:24725]	
H2AFZ	0,65	14,94	0	50 x 3	4 q23	H2A histone family member Z [Source:HGNC Symbol;Acc:HGNC:4741]	
SHCBP1	0,65	14,80	0	50 x 1	16 q11	SHC binding and spindle associated 1 [Source:HGNC Symbol;Acc:HGNC:29547]	
CENPN	0,64	14,72	0	50 x 1	16 q23	centromere protein N [Source:HGNC Symbol;Acc:HGNC:30873]	
CCNB2	0,64	14,41	0	50 x 2	15 q22	cyclin B2 [Source:HGNC Symbol;Acc:HGNC:1580]	
GTSE1	0,64	14,39	0	50 x 3	22 q13	G2 and S-phase expressed 1 [Source:HGNC Symbol;Acc:HGNC:13698]	
ASF1B	0,63	14,19	0	50 x 1	19 p13	anti-silencing function 1B histone chaperone [Source:HGNC Symbol;Acc:HGNC:20996]	
CKAP2	0,63	14,18	0	50 x 2	13 q14	cytoskeleton associated protein 2 [Source:HGNC Symbol;Acc:HGNC:1990]	
CDCA8	0,63	14,17	0	50 x 2	1 p34	cell division cycle associated 8 [Source:HGNC Symbol;Acc:HGNC:14629]	
CENPE	0,63	14,16	0	50 x 2	4 q24	centromere protein E [Source:HGNC Symbol;Acc:HGNC:1856]	
CLSPN	0,63	14,07	0	48 x 1	1 p34	claspin [Source:HGNC Symbol;Acc:HGNC:19715]	


DIAPH3	0,63	14,04	0	50 x 3	13 q21	diaphanous related formin 3 [Source:HGNC Symbol;Acc:HGNC:15480]	
DLGAP5	0,63	14,03	0	50 x 2	14 q22	DLG associated protein 5 [Source:HGNC Symbol;Acc:HGNC:16864]	
KIF22	0,62	13,94	0	50 x 1	16 p11	kinesin family member 22 [Source:HGNC Symbol;Acc:HGNC:6391]	
SMC2	0,62	13,88	0	50 x 1	9 q31	structural maintenance of chromosomes 2 [Source:HGNC Symbol;Acc:HGNC:14011]	
EXO1	0,62	13,72	0	48 x 1	1 q43	exonuclease 1 [Source:HGNC Symbol;Acc:HGNC:3511]	
	0,62	13,64	0	50 x 1			
CCNA2	0,61	13,48	0	50 x 2	4 q27	cyclin A2 [Source:HGNC Symbol;Acc:HGNC:1578]	
GMNN	0,61	13,40	0	49 x 1	6 p22	geminin, DNA replication inhibitor [Source:HGNC Symbol;Acc:HGNC:17493]	
PARPBP	0,61	13,36	0	50 x 1	12 q23	PARP1 binding protein [Source:HGNC Symbol;Acc:HGNC:26074]	
ANP32E	0,61	13,29	0	50 x 4	1 q21	acidic nuclear phosphoprotein 32 family member E [Source:HGNC Symbol;Acc:HGNC:16673]	
FEN1	0,61	13,28	0	49 x 1	11 q12	flap structure-specific endonuclease 1 [Source:HGNC Symbol;Acc:HGNC:3650]	
KPNA2	0,60	13,21	0	50 x 2	17 q24	karyopherin subunit alpha 2 [Source:HGNC Symbol;Acc:HGNC:6395]	
HMGB1	0,60	13,21	0	50 x 5	13 q12	high mobility group box 1 [Source:HGNC Symbol;Acc:HGNC:4983]	
RFC3	0,60	13,18	0	49 x 1	13 q13	replication factor C subunit 3 [Source:HGNC Symbol;Acc:HGNC:9971]	
TTK	0,60	13,18	0	50 x 2	6 q14	TTK protein kinase [Source:HGNC Symbol;Acc:HGNC:12401]	
DTL	0,60	13,15	0	48 x 1	1 q32	denticleless E3 ubiquitin protein ligase homolog [Source:HGNC Symbol;Acc:HGNC:30288]	
CDKN3	0,60	13,13	0	50 x 2	14 q22	cyclin dependent kinase inhibitor 3 [Source:HGNC Symbol;Acc:HGNC:1791]	
STMN1	0,60	13,07	0	50 x 2	1 p36	stathmin 1 [Source:HGNC Symbol;Acc:HGNC:6510]	
CCNF	0,60	13,06	0	50 x 4	16 p13	cyclin F [Source:HGNC Symbol;Acc:HGNC:1591]	
RRM1	0,60	13,04	0	50 x 1	11 p15	ribonucleotide reductase catalytic subunit M1 [Source:HGNC Symbol;Acc:HGNC:10451]	
UHRF1	0,60	13,03	0	47 x 1	19 p13	ubiquitin like with PHD and ring finger domains 1 [Source:HGNC Symbol;Acc:HGNC:12556]	
DEK	0,60	13,02	0	49 x 1	6 p22	DEK proto-oncogene [Source:HGNC Symbol;Acc:HGNC:2768]	
ORC6	0,60	12,97	0	48 x 1	16 q11	origin recognition complex subunit 6 [Source:HGNC Symbol;Acc:HGNC:17151]	
KIF14	0,59	12,92	0	50 x 3	1 q32	kinesin family member 14 [Source:HGNC Symbol;Acc:HGNC:19181]	
PLK4	0,59	12,86	0	49 x 1	4 q28	polo like kinase 4 [Source:HGNC Symbol;Acc:HGNC:11397]	
TRIP13	0,59	12,83	0	50 x 1	5 p15	thyroid hormone receptor interactor 13 [Source:HGNC Symbol;Acc:HGNC:12307]	
CENPU	0,59	12,81	0	49 x 1	4 q35	centromere protein U [Source:HGNC Symbol;Acc:HGNC:21348]	
KIF11	0,59	12,79	0	50 x 3	10 q23	kinesin family member 11 [Source:HGNC Symbol;Acc:HGNC:6388]	
NCAPH	0,59	12,66	0	50 x 1	2 q11	non-SMC condensin I complex subunit H [Source:HGNC Symbol;Acc:HGNC:1112]	
CENPW	0,59	12,63	0	50 x 2	6 q22	centromere protein W [Source:HGNC Symbol;Acc:HGNC:21488]	
GINS2	0,58	12,52	0	48 x 1	16 q24	GINS complex subunit 2 [Source:HGNC Symbol;Acc:HGNC:24575]	
CKS2	0,58	12,48	0	50 x 2	9 q22	CDC28 protein kinase regulatory subunit 2 [Source:HGNC Symbol;Acc:HGNC:2000]	
HELLS	0,58	12,31	0	48 x 1	10 q23	helicase, lymphoid-specific [Source:HGNC Symbol;Acc:HGNC:4861]	


POLQ	0,57	12,07	0	47 x 2	3 q13	polymerase (DNA) theta [Source:HGNC Symbol;Acc:HGNC:9186]	
WDR76	0,57	11,98	0	47 x 1	15 q15	WD repeat domain 76 [Source:HGNC Symbol;Acc:HGNC:25773]	
RHNO1	0,56	11,94	0	50 x 1	12 p13	RAD9-HUS1-RAD1 interacting nuclear orphan 1 [Source:HGNC Symbol;Acc:HGNC:28206]	
	0,56	11,89	0	50 x 3			
HMGN2	0,56	11,84	0	50 x 4	1 p36	high mobility group nucleosomal binding domain 2 [Source:HGNC Symbol;Acc:HGNC:4986]	
RACGAP1	0,56	11,80	0	50 x 2	12 q13	Rac GTPase activating protein 1 [Source:HGNC Symbol;Acc:HGNC:9804]	
KIF4A	0,56	11,79	0	50 x 3	X q13	kinesin family member 4A [Source:HGNC Symbol;Acc:HGNC:13339]	
MMS22L	0,56	11,79	0	48 x 1	6 q16	MMS22 like, DNA repair protein [Source:HGNC Symbol;Acc:HGNC:21475]	
CEP55	0,56	11,72	0	50 x 3	10 q23	centrosomal protein 55 [Source:HGNC Symbol;Acc:HGNC:1161]	
SKA2	0,56	11,69	0	50 x 4	17 q22	spindle and kinetochore associated complex subunit 2 [Source:HGNC Symbol;Acc:HGNC:28006]	
BUB3	0,56	11,68	0	49 x 1	10 q26	BUB3, mitotic checkpoint protein [Source:HGNC Symbol;Acc:HGNC:1151]	
KIF18B	0,56	11,68	0	49 x 5	17 q21	kinesin family member 18B [Source:HGNC Symbol;Acc:HGNC:27102]	
CENPL	0,55	11,63	0	50 x 1	1 q25	centromere protein L [Source:HGNC Symbol;Acc:HGNC:17879]	
HIST1H3B	0,55	11,56	0	49 x 4	6 p22	histone cluster 1, H3b [Source:HGNC Symbol;Acc:HGNC:4776]	
FAM64A	0,55	11,50	0	50 x 2	17 p13	family with sequence similarity 64 member A [Source:HGNC Symbol;Acc:HGNC:25483]	
KIAA1524	0,55	11,49	0	50 x 2	3 q13	KIAA1524 [Source:HGNC Symbol;Acc:HGNC:29302]	
EZH2	0,55	11,48	0	48 x 1	7 q36	enhancer of zeste 2 polycomb repressive complex 2 subunit [Source:HGNC Symbol;Acc:HGNC:3527]	
CDC6	0,55	11,44	0	48 x 1	17 q21	cell division cycle 6 [Source:HGNC Symbol;Acc:HGNC:1744]	
IQGAP3	0,55	11,37	0	50 x 4	1 q22	IQ motif containing GTPase activating protein 3 [Source:HGNC Symbol;Acc:HGNC:20669]	
BLM	0,54	11,27	0	49 x 1	15 q26	Bloom syndrome RecQ like helicase [Source:HGNC Symbol;Acc:HGNC:1058]	
MCM7	0,54	11,22	0	48 x 1	7 q22	minichromosome maintenance complex component 7 [Source:HGNC Symbol;Acc:HGNC:6950]	
ECT2	0,54	11,16	0	50 x 2	3 q26	epithelial cell transforming 2 [Source:HGNC Symbol;Acc:HGNC:3155]	
TUBB	0,54	11,14	0	50 x 5	CHR_HSCHR6_M	tubulin beta class I [Source:HGNC Symbol;Acc:HGNC:20778]	
CCNB1	0,53	11,03	0	50 x 2	5 q13	cyclin B1 [Source:HGNC Symbol;Acc:HGNC:1579]	
USP1	0,53	11,00	0	48 x 1	1 p31	ubiquitin specific peptidase 1 [Source:HGNC Symbol;Acc:HGNC:12607]	
CCDC150	0,53	10,95	0	48 x 1	2 q33	coiled-coil domain containing 150 [Source:HGNC Symbol;Acc:HGNC:26834]	
NEIL3	0,53	10,88	0	49 x 4	4 q34	nei like DNA glycosylase 3 [Source:HGNC Symbol;Acc:HGNC:24573]	
C1orf112	0,53	10,83	0	50 x 2	1 q24	chromosome 1 open reading frame 112 [Source:HGNC Symbol;Acc:HGNC:25565]	
ATAD5	0,53	10,83	0	47 x 1	17 q11	ATPase family, AAA domain containing 5 [Source:HGNC Symbol;Acc:HGNC:25752]	
FBXO5	0,53	10,80	0	49 x 1	6 q25	F-box protein 5 [Source:HGNC Symbol;Acc:HGNC:13584]	
TROAP	0,52	10,71	0	50 x 2	12 q13	trophinin associated protein [Source:HGNC Symbol;Acc:HGNC:12327]	
CCNE2	0,52	10,70	0	48 x 1	8 q22	cyclin E2 [Source:HGNC Symbol;Acc:HGNC:1590]	
MIS18BP1	0,52	10,70	0	50 x 1	14 q21	MIS18 binding protein 1 [Source:HGNC Symbol;Acc:HGNC:20190]	


CENPI	0,52	10,60	0	50 x 4	X q22	centromere protein I [Source:HGNC Symbol;Acc:HGNC:3968]	
KIF20A	0,52	10,59	0	50 x 3	5 q31	kinesin family member 20A [Source:HGNC Symbol;Acc:HGNC:9787]	
AURKA	0,52	10,56	0	50 x 3	20 q13	aurora kinase A [Source:HGNC Symbol;Acc:HGNC:11393]	
SPDL1	0,52	10,53	0	50 x 2	5 q35	spindle apparatus coiled-coil protein 1 [Source:HGNC Symbol;Acc:HGNC:26010]	
MCM10	0,52	10,50	0	47 x 1	10 p13	minichromosome maintenance 10 replication initiation factor [Source:HGNC Symbol;Acc:HGNC:18043]	
KIF18A	0,51	10,45	0	50 x 3	11 p14	kinesin family member 18A [Source:HGNC Symbol;Acc:HGNC:29441]	
TUBA1B	0,51	10,44	0	50 x 4	12 q13	tubulin alpha 1b [Source:HGNC Symbol;Acc:HGNC:18809]	
MYBL2	0,51	10,42	0	48 x 1	20 q13	MYB proto-oncogene like 2 [Source:HGNC Symbol;Acc:HGNC:7548]	
SMC3	0,51	10,42	0	47 x 1	10 q25	structural maintenance of chromosomes 3 [Source:HGNC Symbol;Acc:HGNC:2468]	
NCAPG2	0,51	10,40	0	50 x 3	7 q36	non-SMC condensin II complex subunit G2 [Source:HGNC Symbol;Acc:HGNC:21904]	
HIST1H1A	0,51	10,33	0	48 x 4	6 p22	histone cluster 1, H1a [Source:HGNC Symbol;Acc:HGNC:4715]	
C18orf54	0,51	10,29	0	50 x 4	18 q21	chromosome 18 open reading frame 54 [Source:HGNC Symbol;Acc:HGNC:13796]	
BRCA2	0,51	10,28	0	48 x 1	13 q13	BRCA2, DNA repair associated [Source:HGNC Symbol;Acc:HGNC:1101]	
FOXM1	0,51	10,28	0	50 x 3	12 p13	forkhead box M1 [Source:HGNC Symbol;Acc:HGNC:3818]	
CDCA2	0,51	10,25	0	50 x 4	8 p21	cell division cycle associated 2 [Source:HGNC Symbol;Acc:HGNC:14623]	
GINS1	0,51	10,23	0	47 x 1	20 p11	GINS complex subunit 1 [Source:HGNC Symbol;Acc:HGNC:28980]	
POLD3	0,50	10,17	0	48 x 1	11 q13	polymerase (DNA) delta 3, accessory subunit [Source:HGNC Symbol;Acc:HGNC:20932]	
LBR	0,50	10,16	0	50 x 2	1 q42	lamin B receptor [Source:HGNC Symbol;Acc:HGNC:6518]	
PRR11	0,50	10,11	0	50 x 3	17 q22	proline rich 11 [Source:HGNC Symbol;Acc:HGNC:25619]	
VRK1	0,50	10,11	0	48 x 1	14 q32	vaccinia related kinase 1 [Source:HGNC Symbol;Acc:HGNC:12718]	
CIT	0,50	10,09	0	50 x 4	12 q24	citron rho-interacting serine/threonine kinase [Source:HGNC Symbol;Acc:HGNC:1985]	
KNTC1	0,50	10,09	0	48 x 1	12 q24	kinetochore associated 1 [Source:HGNC Symbol;Acc:HGNC:17255]	
PLK1	0,50	10,08	0	50 x 3	16 p12	polo like kinase 1 [Source:HGNC Symbol;Acc:HGNC:9077]	
E2F8	0,50	10,07	0	47 x 4	11 p15	E2F transcription factor 8 [Source:HGNC Symbol;Acc:HGNC:24727]	
C21orf58	0,50	10,07	0	48 x 5	21 q22	chromosome 21 open reading frame 58 [Source:HGNC Symbol;Acc:HGNC:1300]	
PSMC3IP	0,50	10,05	0	48 x 1	17 q21	PSMC3 interacting protein [Source:HGNC Symbol;Acc:HGNC:17928]	
TMSB15A	0,50	10,02	0	50 x 1	X q22	thymosin beta 15a [Source:HGNC Symbol;Acc:HGNC:30744]	
HMMR	0,50	9,96	0	50 x 3	5 q34	hyaluronan mediated motility receptor [Source:HGNC Symbol;Acc:HGNC:5012]	
MCM4	0,49	9,91	0	48 x 1	8 q11	minichromosome maintenance complex component 4 [Source:HGNC Symbol;Acc:HGNC:6947]	
NEK2	0,49	9,89	0	50 x 3	1 q32	NIMA related kinase 2 [Source:HGNC Symbol;Acc:HGNC:7745]	
MNS1	0,49	9,88	0	49 x 3	15 q21	meiosis specific nuclear structural 1 [Source:HGNC Symbol;Acc:HGNC:29636]	
CSE1L	0,49	9,88	0	48 x 1	20 q13	chromosome segregation 1 like [Source:HGNC Symbol;Acc:HGNC:2431]	
WEE1	0,49	9,87	0	48 x 1	11 p15	WEE1 G2 checkpoint kinase [Source:HGNC Symbol;Acc:HGNC:12761]	


ARHGAP11A	0,49	9,83	0	50 x 5	CHR_HSCHR15_6	Rho GTPase activating protein 11A [Source:HGNC Symbol;Acc:HGNC:15783]	
TUBB4B	0,49	9,81	0	50 x 3	9 q34	tubulin beta 4B class IVb [Source:HGNC Symbol;Acc:HGNC:20771]	
CENPM	0,49	9,76	0	50 x 1	22 q13	centromere protein M [Source:HGNC Symbol;Acc:HGNC:18352]	
DTYMK	0,49	9,75	0	50 x 1	2 q37	deoxythymidylate kinase [Source:HGNC Symbol;Acc:HGNC:3061]	
HAUS8	0,49	9,73	0	48 x 1	19 p13	HAUS augmin like complex subunit 8 [Source:HGNC Symbol;Acc:HGNC:30532]	
CDC20	0,48	9,66	0	50 x 3	1 p34	cell division cycle 20 [Source:HGNC Symbol;Acc:HGNC:1723]	
MCM3	0,48	9,61	0	48 x 1	6 p12	minichromosome maintenance complex component 3 [Source:HGNC Symbol;Acc:HGNC:6945]	
FANCB	0,48	9,58	0	48 x 1	X p22	Fanconi anemia complementation group B [Source:HGNC Symbol;Acc:HGNC:3583]	
HIST1H3C	0,48	9,57	0	48 x 5	6 p22	histone cluster 1, H3c [Source:HGNC Symbol;Acc:HGNC:4768]	
DNAJC9	0,48	9,56	0	48 x 1	10 q22	DnaJ heat shock protein family (Hsp40) member C9 [Source:HGNC Symbol;Acc:HGNC:19123]	
RAD21	0,48	9,56	0	50 x 3	8 q24	RAD21 cohesin complex component [Source:HGNC Symbol;Acc:HGNC:9811]	
PCNA	0,48	9,52	0	48 x 1	20 p12	proliferating cell nuclear antigen [Source:HGNC Symbol;Acc:HGNC:8729]	
WHSC1	0,48	9,52	0	48 x 1	4 p16	Wolf-Hirschhorn syndrome candidate 1 [Source:HGNC Symbol;Acc:HGNC:12766]	
APOBEC3B	0,48	9,50	0	50 x 4	22 q13	apolipoprotein B mRNA editing enzyme catalytic subunit 3B [Source:HGNC Symbol;Acc:HGNC:17352]	
E2F7	0,48	9,47	0	48 x 5	12 q21	E2F transcription factor 7 [Source:HGNC Symbol;Acc:HGNC:23820]	
STIL	0,47	9,38	0	49 x 4	1 p33	SCL/TAL1 interrupting locus [Source:HGNC Symbol;Acc:HGNC:10879]	
RTKN2	0,47	9,37	0	48 x 5	10 q21	rhotekin 2 [Source:HGNC Symbol;Acc:HGNC:19364]	
FAM83D	0,47	9,29	0	50 x 5	20 q11	family with sequence similarity 83 member D [Source:HGNC Symbol;Acc:HGNC:16122]	
RFC4	0,47	9,28	0	48 x 1	3 q27	replication factor C subunit 4 [Source:HGNC Symbol;Acc:HGNC:9972]	
ARL6IP1	0,47	9,27	0	50 x 3	16 p12	ADP ribosylation factor like GTPase 6 interacting protein 1 [Source:HGNC Symbol;Acc:HGNC:697]	
TMEM97	0,47	9,27	0	48 x 1	17 q11	transmembrane protein 97 [Source:HGNC Symbol;Acc:HGNC:28106]	
DEPDC1B	0,47	9,25	0	50 x 3	5 q12	DEP domain containing 1B [Source:HGNC Symbol;Acc:HGNC:24902]	
DNMT1	0,47	9,21	0	48 x 1	19 p13	DNA (cytosine-5-)-methyltransferase 1 [Source:HGNC Symbol;Acc:HGNC:2976]	
C5orf34	0,46	9,14	0	49 x 3	5 p12	chromosome 5 open reading frame 34 [Source:HGNC Symbol;Acc:HGNC:24738]	
CBX5	0,46	9,09	0	48 x 4	12 q13	chromobox 5 [Source:HGNC Symbol;Acc:HGNC:1555]	
CDC45	0,46	9,07	0	47 x 1	22 q11	cell division cycle 45 [Source:HGNC Symbol;Acc:HGNC:1739]	
DNA2	0,46	9,05	0	48 x 1	10 q21	DNA replication helicase/nuclease 2 [Source:HGNC Symbol;Acc:HGNC:2939]	
RFC2	0,46	9,04	0	48 x 1	7 q11	replication factor C subunit 2 [Source:HGNC Symbol;Acc:HGNC:9970]	
NUP155	0,46	9,03	0	48 x 4	5 p13	nucleoporin 155 [Source:HGNC Symbol;Acc:HGNC:8063]	
	0,46	9,01	0	47 x 1			
TACC3	0,46	8,98	0	50 x 4	4 p16	transforming acidic coiled-coil containing protein 3 [Source:HGNC Symbol;Acc:HGNC:11524]	
CDC25C	0,46	8,95	0	50 x 4	5 q31	cell division cycle 25C [Source:HGNC Symbol;Acc:HGNC:1727]	
MZT1	0,46	8,95	0	50 x 4	13 q21	mitotic spindle organizing protein 1 [Source:HGNC Symbol;Acc:HGNC:33830]	


CEP152	0,46	8,92	0	48 x 1	15 q21	centrosomal protein 152 [Source:HGNC Symbol;Acc:HGNC:29298]	
OIP5	0,45	8,83	0	50 x 4	15 q15	Opa interacting protein 5 [Source:HGNC Symbol;Acc:HGNC:20300]	
CHAF1A	0,45	8,80	0	47 x 1	19 p13	chromatin assembly factor 1 subunit A [Source:HGNC Symbol;Acc:HGNC:1910]	
DBF4	0,45	8,73	1,11E-16	50 x 3	7 q21	DBF4 zinc finger [Source:HGNC Symbol;Acc:HGNC:17364]	
CENPO	0,45	8,70	1,11E-16	50 x 3	2 p23	centromere protein O [Source:HGNC Symbol;Acc:HGNC:28152]	
DCK	0,44	8,62	2,22E-16	48 x 1	4 q13	deoxycytidine kinase [Source:HGNC Symbol;Acc:HGNC:2704]	
XRCC2	0,44	8,59	2,22E-16	47 x 3	7 q36	X-ray repair cross complementing 2 [Source:HGNC Symbol;Acc:HGNC:12829]	
CENPQ	0,44	8,59	2,22E-16	48 x 1	6 p12	centromere protein Q [Source:HGNC Symbol;Acc:HGNC:21347]	
RNASEH2A	0,44	8,58	2,22E-16	50 x 1	19 p13	ribonuclease H2 subunit A [Source:HGNC Symbol;Acc:HGNC:18518]	
E2F2	0,44	8,55	2,22E-16	46 x 1	CHR_HSCHR1_4_	E2F transcription factor 2 [Source:HGNC Symbol;Acc:HGNC:3114]	
DHFR	0,44	8,52	4,44E-16	48 x 5	5 q14	dihydrofolate reductase [Source:HGNC Symbol;Acc:HGNC:2861]	
CHAF1B	0,44	8,49	4,44E-16	47 x 1	21 q22	chromatin assembly factor 1 subunit B [Source:HGNC Symbol;Acc:HGNC:1911]	
CENPJ	0,44	8,44	6,66E-16	50 x 5	13 q12	centromere protein J [Source:HGNC Symbol;Acc:HGNC:17272]	
MTFR2	0,43	8,44	6,66E-16	50 x 4	6 q23	mitochondrial fission regulator 2 [Source:HGNC Symbol;Acc:HGNC:21115]	
SMC1A	0,43	8,38	9,99E-16	48 x 1	X p11	structural maintenance of chromosomes 1A [Source:HGNC Symbol;Acc:HGNC:11111]	
TOPBP1	0,43	8,38	9,99E-16	47 x 1	3 q22	topoisomerase (DNA) II binding protein 1 [Source:HGNC Symbol;Acc:HGNC:17008]	
PTTG1	0,43	8,36	1,11E-15	50 x 3	5 q33	pituitary tumor-transforming 1 [Source:HGNC Symbol;Acc:HGNC:9690]	
POC1A	0,43	8,35	1,11E-15	49 x 4	3 p21	POC1 centriolar protein A [Source:HGNC Symbol;Acc:HGNC:24488]	
LRR1	0,43	8,34	1,33E-15	49 x 1	14 q21	leucine rich repeat protein 1 [Source:HGNC Symbol;Acc:HGNC:19742]	
NCAPD3	0,43	8,33	1,33E-15	49 x 4	11 q25	non-SMC condensin II complex subunit D3 [Source:HGNC Symbol;Acc:HGNC:28952]	
KNSTRN	0,43	8,27	2,11E-15	50 x 2	15 q15	kinetochore-localized astrin/SPAG5 binding protein [Source:HGNC Symbol;Acc:HGNC:30767]	
ZWILCH	0,43	8,25	2,44E-15	50 x 1	15 q22	zwilch kinetochore protein [Source:HGNC Symbol;Acc:HGNC:25468]	
DSN1	0,43	8,22	2,89E-15	48 x 1	20 q11	DSN1 homolog, MIS12 kinetochore complex component [Source:HGNC Symbol;Acc:HGNC:16165]	
MSH2	0,42	8,19	3,55E-15	47 x 1	2 p21	mutS homolog 2 [Source:HGNC Symbol;Acc:HGNC:7325]	
RBBP8	0,42	8,11	6,11E-15	47 x 1	18 q11	RB binding protein 8, endonuclease [Source:HGNC Symbol;Acc:HGNC:9891]	
MPHOSPH9	0,42	8,08	7,55E-15	49 x 1	12 q24	M-phase phosphoprotein 9 [Source:HGNC Symbol;Acc:HGNC:7215]	
NCAPD2	0,42	8,07	8,44E-15	50 x 3	12 p13	non-SMC condensin I complex subunit D2 [Source:HGNC Symbol;Acc:HGNC:24305]	
SYNE2	0,42	7,99	1,38E-14	48 x 5	14 q23	spectrin repeat containing nuclear envelope protein 2 [Source:HGNC Symbol;Acc:HGNC:17084]	
RNF168	0,41	7,91	2,46E-14	48 x 4	3 q29	ring finger protein 168 [Source:HGNC Symbol;Acc:HGNC:26661]	
CCP110	0,41	7,90	2,58E-14	47 x 2	16 p12	centriolar coiled-coil protein 110 [Source:HGNC Symbol;Acc:HGNC:24342]	
DDX39A	0,41	7,86	3,35E-14	50 x 2	19 p13	DEAD-box helicase 39A [Source:HGNC Symbol;Acc:HGNC:17821]	
DDIAS	0,41	7,84	3,77E-14	48 x 4	11 q14	DNA damage induced apoptosis suppressor [Source:HGNC Symbol;Acc:HGNC:26351]	
GGCT	0,41	7,84	3,91E-14	47 x 1	7 p14	gamma-glutamylcyclotransferase [Source:HGNC Symbol;Acc:HGNC:21705]	


CCDC18	0,41	7,80	4,93E-14	50 x 4	1 p22	coiled-coil domain containing 18 [Source:HGNC Symbol;Acc:HGNC:30370]	
GAS2L3	0,41	7,78	5,58E-14	50 x 3	12 q23	growth arrest specific 2 like 3 [Source:HGNC Symbol;Acc:HGNC:27475]	
GEN1	0,41	7,74	7,43E-14	48 x 1	2 p24	GEN1, Holliday junction 5' flap endonuclease [Source:HGNC Symbol;Acc:HGNC:26881]	
WDHD1	0,40	7,68	1,10E-13	47 x 1	14 q22	WD repeat and HMG-box DNA binding protein 1 [Source:HGNC Symbol;Acc:HGNC:23170]	
HIST1H1E	0,40	7,66	1,20E-13	47 x 1	6 p22	histone cluster 1, H1e [Source:HGNC Symbol;Acc:HGNC:4718]	
ITGB3BP	0,40	7,64	1,43E-13	49 x 1	1 p31	integrin subunit beta 3 binding protein [Source:HGNC Symbol;Acc:HGNC:6157]	
CDCA7L	0,40	7,61	1,68E-13	47 x 1	7 p15	cell division cycle associated 7 like [Source:HGNC Symbol;Acc:HGNC:30777]	
CHEK2	0,40	7,57	2,20E-13	47 x 1	22 q12	checkpoint kinase 2 [Source:HGNC Symbol;Acc:HGNC:16627]	
EZR	0,40	7,56	2,39E-13	48 x 4	6 q25	ezrin [Source:HGNC Symbol;Acc:HGNC:12691]	
NASP	0,39	7,50	3,45E-13	48 x 1	1 p34	nuclear autoantigenic sperm protein [Source:HGNC Symbol;Acc:HGNC:7644]	
CHEK1	0,39	7,49	3,63E-13	47 x 1	11 q24	checkpoint kinase 1 [Source:HGNC Symbol;Acc:HGNC:1925]	
CMC2	0,39	7,48	3,97E-13	47 x 1	16 q23	C-X9-C motif containing 2 [Source:HGNC Symbol;Acc:HGNC:24447]	
UBR7	0,39	7,44	5,24E-13	47 x 1	CHR_HSCHR14_7	ubiquitin protein ligase E3 component n-recognin 7 (putative) [Source:HGNC Symbol;Acc:HGNC:20344]	
PCNT	0,39	7,43	5,66E-13	48 x 2	21 q22	pericentrin [Source:HGNC Symbol;Acc:HGNC:16068]	
HLTF	0,39	7,35	9,00E-13	47 x 3	3 q24	helicase like transcription factor [Source:HGNC Symbol;Acc:HGNC:11099]	
MND1	0,39	7,34	9,84E-13	47 x 4	4 q31	meiotic nuclear divisions 1 [Source:HGNC Symbol;Acc:HGNC:24839]	
SAE1	0,38	7,28	1,39E-12	48 x 3	19 q13	SUMO1 activating enzyme subunit 1 [Source:HGNC Symbol;Acc:HGNC:30660]	
INCENP	0,38	7,27	1,55E-12	50 x 5	11 q12	inner centromere protein [Source:HGNC Symbol;Acc:HGNC:6058]	
PKMYT1	0,38	7,23	1,93E-12	47 x 1	16 p13	protein kinase, membrane associated tyrosine/threonine 1 [Source:HGNC Symbol;Acc:HGNC:29650]	
HAUS1	0,38	7,22	2,07E-12	48 x 1	18 q21	HAUS augmin like complex subunit 1 [Source:HGNC Symbol;Acc:HGNC:25174]	
CCDC34	0,38	7,21	2,21E-12	50 x 5	11 p14	coiled-coil domain containing 34 [Source:HGNC Symbol;Acc:HGNC:25079]	
LIG1	0,38	7,17	2,87E-12	47 x 1	19 q13	DNA ligase 1 [Source:HGNC Symbol;Acc:HGNC:6598]	
HAT1	0,38	7,16	3,10E-12	46 x 1	2 q31	histone acetyltransferase 1 [Source:HGNC Symbol;Acc:HGNC:4821]	
BRCA1	0,38	7,15	3,17E-12	48 x 2	17 q21	BRCA1, DNA repair associated [Source:HGNC Symbol;Acc:HGNC:1100]	
FANCA	0,38	7,11	4,08E-12	47 x 1	16 q24	Fanconi anemia complementation group A [Source:HGNC Symbol;Acc:HGNC:3582]	
NFYB	0,38	7,08	4,95E-12	47 x 1	12 q23	nuclear transcription factor Y subunit beta [Source:HGNC Symbol;Acc:HGNC:7805]	
RAD51C	0,38	7,08	5,02E-12	48 x 1	17 q22	RAD51 paralog C [Source:HGNC Symbol;Acc:HGNC:9820]	
CEP78	0,38	7,08	5,10E-12	49 x 5	9 q21	centrosomal protein 78 [Source:HGNC Symbol;Acc:HGNC:25740]	
C19orf48	0,37	7,06	5,62E-12	47 x 1	19 q13	chromosome 19 open reading frame 48 [Source:HGNC Symbol;Acc:HGNC:29667]	
ZGRF1	0,37	7,02	7,37E-12	47 x 1	4 q25	zinc finger GRF-type containing 1 [Source:HGNC Symbol;Acc:HGNC:25654]	
RAD54L	0,37	7,01	7,53E-12	46 x 2	1 p34	RAD54-like (S. cerevisiae) [Source:HGNC Symbol;Acc:HGNC:9826]	
SKP2	0,37	6,98	9,11E-12	46 x 1	5 p13	S-phase kinase-associated protein 2, E3 ubiquitin protein ligase [Source:HGNC Symbol;Acc:HGNC:10901]	
NUCKS1	0,37	6,94	1,15E-11	50 x 5	1 q32	nuclear casein kinase and cyclin dependent kinase substrate 1 [Source:HGNC Symbol;Acc:HGNC:29923]	


MCM6	0,37	6,94	1,21E-11	46 x 1	2 q21	minichromosome maintenance complex component 6 [Source:HGNC Symbol;Acc:HGNC:6949]	
RAD51	0,37	6,92	1,31E-11	47 x 1	15 q15	RAD51 recombinase [Source:HGNC Symbol;Acc:HGNC:9817]	
TMEM106C	0,37	6,92	1,34E-11	46 x 1	12 q13	transmembrane protein 106C [Source:HGNC Symbol;Acc:HGNC:28775]	
LSM5	0,37	6,89	1,62E-11	50 x 5	7 p14	LSM5 homolog, U6 small nuclear RNA and mRNA degradation associated [Source:HGNC Symbol;Acc:HGNC:17162]	
TUBG1	0,37	6,87	1,80E-11	48 x 4	17 q21	tubulin gamma 1 [Source:HGNC Symbol;Acc:HGNC:12417]	
MCM5	0,37	6,86	1,94E-11	46 x 1	22 q12	minichromosome maintenance complex component 5 [Source:HGNC Symbol;Acc:HGNC:6948]	
HMGB3	0,36	6,84	2,18E-11	50 x 4	X q28	high mobility group box 3 [Source:HGNC Symbol;Acc:HGNC:5004]	
DUT	0,36	6,83	2,25E-11	47 x 1	15 q21	deoxyuridine triphosphatase [Source:HGNC Symbol;Acc:HGNC:3078]	
PHTF2	0,36	6,83	2,25E-11	50 x 4	7 q11	putative homeodomain transcription factor 2 [Source:HGNC Symbol;Acc:HGNC:13411]	
PDS5B	0,36	6,83	2,35E-11	48 x 5	13 q13	PDS5 cohesin associated factor B [Source:HGNC Symbol;Acc:HGNC:20418]	
H2AFV	0,36	6,81	2,59E-11	50 x 5	7 p13	H2A histone family member V [Source:HGNC Symbol;Acc:HGNC:20664]	
ZNF367	0,36	6,78	3,15E-11	46 x 2	9 q22	zinc finger protein 367 [Source:HGNC Symbol;Acc:HGNC:18320]	
PRIM2	0,36	6,75	3,76E-11	47 x 1	6 p11	primase (DNA) subunit 2 [Source:HGNC Symbol;Acc:HGNC:9370]	
DCLRE1C	0,36	6,73	4,19E-11	48 x 2	10 p13	DNA cross-link repair 1C [Source:HGNC Symbol;Acc:HGNC:17642]	
MCM8	0,36	6,73	4,32E-11	47 x 4	20 p12	minichromosome maintenance 8 homologous recombination repair factor [Source:HGNC Symbol;Acc:HGNC:16147]	
CEP57L1	0,36	6,70	5,03E-11	50 x 5	6 q21	centrosomal protein 57 like 1 [Source:HGNC Symbol;Acc:HGNC:21561]	
RBMX	0,36	6,68	5,65E-11	50 x 5	X q26	RNA binding motif protein, X-linked [Source:HGNC Symbol;Acc:HGNC:9910]	
RAD1	0,35	6,63	7,75E-11	47 x 1	5 p13	RAD1 checkpoint DNA exonuclease [Source:HGNC Symbol;Acc:HGNC:9806]	
G2E3	0,35	6,61	8,56E-11	50 x 4	14 q12	G2/M-phase specific E3 ubiquitin protein ligase [Source:HGNC Symbol;Acc:HGNC:20338]	
CCDC138	0,35	6,59	9,43E-11	47 x 3	2 q13	coiled-coil domain containing 138 [Source:HGNC Symbol;Acc:HGNC:26531]	
UACA	0,35	6,58	1,01E-10	50 x 4	15 q23	uveal autoantigen with coiled-coil domains and ankyrin repeats [Source:HGNC Symbol;Acc:HGNC:15947]	
RANBP1	0,35	6,57	1,08E-10	46 x 1	22 q11	RAN binding protein 1 [Source:HGNC Symbol;Acc:HGNC:9847]	
	0,35	6,52	1,47E-10	47 x 2			
RFWD3	0,35	6,48	1,84E-10	46 x 1	16 q23	ring finger and WD repeat domain 3 [Source:HGNC Symbol;Acc:HGNC:25539]	
TEX30	0,34	6,42	2,67E-10	48 x 1	13 q33	testis expressed 30 [Source:HGNC Symbol;Acc:HGNC:25188]	
C4orf46	0,34	6,38	3,36E-10	47 x 4	4 q32	chromosome 4 open reading frame 46 [Source:HGNC Symbol;Acc:HGNC:27320]	
RMI1	0,34	6,37	3,52E-10	47 x 4	9 q21	RecQ mediated genome instability 1 [Source:HGNC Symbol;Acc:HGNC:25764]	
KPNB1	0,34	6,28	5,97E-10	46 x 2	17 q21	karyopherin subunit beta 1 [Source:HGNC Symbol;Acc:HGNC:6400]	
FIGNL1	0,33	6,17	1,07E-09	46 x 1	7 p12	fidgetin like 1 [Source:HGNC Symbol;Acc:HGNC:13286]	
PRIM1	0,33	6,13	1,37E-09	46 x 1	12 q13	primase (DNA) subunit 1 [Source:HGNC Symbol;Acc:HGNC:9369]	
RBL1	0,33	6,09	1,66E-09	46 x 1	20 q11	RB transcriptional corepressor like 1 [Source:HGNC Symbol;Acc:HGNC:9893]	
TTF2	0,33	6,07	1,85E-09	49 x 4	1 p13	transcription termination factor 2 [Source:HGNC Symbol;Acc:HGNC:12398]	
EXOSC9	0,33	6,01	2,60E-09	46 x 1	4 q27	exosome component 9 [Source:HGNC Symbol;Acc:HGNC:9137]	


CKLF	0,32	5,99	2,97E-09	47 x 2	16 q21	chemokine-like factor [Source:HGNC Symbol;Acc:HGNC:13253]	
COMMD4	0,32	5,97	3,21E-09	47 x 1	15 q24	COMM domain containing 4 [Source:HGNC Symbol;Acc:HGNC:26027]	
EXOSC8	0,32	5,97	3,34E-09	50 x 5	13 q13	exosome component 8 [Source:HGNC Symbol;Acc:HGNC:17035]	
DCP2	0,32	5,96	3,56E-09	48 x 4	5 q22	decapping mRNA 2 [Source:HGNC Symbol;Acc:HGNC:24452]	
LIN54	0,32	5,94	3,79E-09	47 x 4	4 q21	lin-54 DREAM MuvB core complex component [Source:HGNC Symbol;Acc:HGNC:25397]	
ORC1	0,32	5,90	4,78E-09	46 x 2	1 p32	origin recognition complex subunit 1 [Source:HGNC Symbol;Acc:HGNC:8487]	
TMEM237	0,32	5,87	5,65E-09	47 x 3	2 q33	transmembrane protein 237 [Source:HGNC Symbol;Acc:HGNC:14432]	
PRIMPOL	0,32	5,86	6,08E-09	46 x 1	4 q35	primase and DNA directed polymerase [Source:HGNC Symbol;Acc:HGNC:26575]	
NUP35	0,32	5,82	7,57E-09	46 x 1	2 q32	nucleoporin 35 [Source:HGNC Symbol;Acc:HGNC:29797]	
YWHAH	0,31	5,79	8,57E-09	46 x 1	22 q12	tyrosine 3-monooxygenase/tryptophan 5-monooxygenase activation protein eta [Source:HGNC Symbol;Acc:HGNC:12853]	
MAPRE1	0,31	5,78	9,44E-09	46 x 2	20 q11	microtubule associated protein RP/EB family member 1 [Source:HGNC Symbol;Acc:HGNC:6890]	
CASP8AP2	0,31	5,76	1,00E-08	46 x 1	6 q15	caspase 8 associated protein 2 [Source:HGNC Symbol;Acc:HGNC:1510]	
NCAPH2	0,31	5,69	1,51E-08	46 x 1	22 q13	non-SMC condensin II complex subunit H2 [Source:HGNC Symbol;Acc:HGNC:25071]	
RAD54B	0,31	5,66	1,76E-08	47 x 3	8 q22	RAD54 homolog B (S. cerevisiae) [Source:HGNC Symbol;Acc:HGNC:17228]	
POLA1	0,31	5,65	1,81E-08	46 x 1	X p22	polymerase (DNA) alpha 1, catalytic subunit [Source:HGNC Symbol;Acc:HGNC:9173]	
NEDD1	0,31	5,65	1,82E-08	47 x 1	12 q23	neural precursor cell expressed, developmentally down-regulated 1 [Source:HGNC Symbol;Acc:HGNC:7723]	
HIRIP3	0,31	5,65	1,86E-08	46 x 2	16 p11	HIRA interacting protein 3 [Source:HGNC Symbol;Acc:HGNC:4917]	
	0,30	5,56	2,96E-08	46 x 1			
PHF19	0,30	5,53	3,36E-08	50 x 5	9 q33	PHD finger protein 19 [Source:HGNC Symbol;Acc:HGNC:24566]	
NUP85	0,30	5,53	3,37E-08	47 x 1	17 q25	nucleoporin 85 [Source:HGNC Symbol;Acc:HGNC:8734]	
NUP37	0,30	5,50	3,97E-08	47 x 3	12 q23	nucleoporin 37 [Source:HGNC Symbol;Acc:HGNC:29929]	
NDC1	0,30	5,47	4,63E-08	50 x 5	1 p32	NDC1 transmembrane nucleoporin [Source:HGNC Symbol;Acc:HGNC:25525]	
POLE	0,30	5,43	5,64E-08	46 x 1	12 q24	polymerase (DNA) epsilon, catalytic subunit [Source:HGNC Symbol;Acc:HGNC:9177]	
PARP2	0,29	5,37	7,72E-08	46 x 1	14 q11	poly(ADP-ribose) polymerase 2 [Source:HGNC Symbol;Acc:HGNC:272]	
TIMM21	0,29	5,33	9,49E-08	46 x 1	18 q22	translocase of inner mitochondrial membrane 21 [Source:HGNC Symbol;Acc:HGNC:25010]	
H2AFY	0,29	5,33	9,68E-08	49 x 5	5 q31	H2A histone family member Y [Source:HGNC Symbol;Acc:HGNC:4740]	
ZDHHC13	0,29	5,28	1,24E-07	47 x 4	11 p15	zinc finger DHHC-type containing 13 [Source:HGNC Symbol;Acc:HGNC:18413]	
MAD2L2	0,29	5,26	1,33E-07	46 x 1	1 p36	MAD2 mitotic arrest deficient-like 2 (yeast) [Source:HGNC Symbol;Acc:HGNC:6764]	
RAD18	0,29	5,24	1,49E-07	46 x 1	3 p25	RAD18, E3 ubiquitin protein ligase [Source:HGNC Symbol;Acc:HGNC:18278]	
FDPS	0,29	5,23	1,56E-07	46 x 1	1 q22	farnesyl diphosphate synthase [Source:HGNC Symbol;Acc:HGNC:3631]	
HIST1H1C	0,29	5,22	1,62E-07	47 x 3	6 p22	histone cluster 1, H1c [Source:HGNC Symbol;Acc:HGNC:4716]	
ACTL6A	0,28	5,15	2,31E-07	46 x 1	3 q26	actin like 6A [Source:HGNC Symbol;Acc:HGNC:24124]	
GMPS	0,28	5,13	2,64E-07	46 x 1	3 q25	guanine monophosphate synthase [Source:HGNC Symbol;Acc:HGNC:4378]	


RNASEH2B	0,28	5,05	3,80E-07	47 x 3	13 q14	ribonuclease H2 subunit B [Source:HGNC Symbol;Acc:HGNC:25671]	
POLE3	0,27	4,99	5,12E-07	46 x 1	9 q32	polymerase (DNA) epsilon 3, accessory subunit [Source:HGNC Symbol;Acc:HGNC:13546]	
ZDHHC6	0,27	4,89	8,29E-07	46 x 1	10 q25	zinc finger DHHC-type containing 6 [Source:HGNC Symbol;Acc:HGNC:19160]	
ARL6IP6	0,26	4,79	1,31E-06	46 x 1	2 q23	ADP ribosylation factor like GTPase 6 interacting protein 6 [Source:HGNC Symbol;Acc:HGNC:24048]	
CASP3	0,26	4,78	1,34E-06	48 x 5	4 q35	caspase 3 [Source:HGNC Symbol;Acc:HGNC:1504]	
MGME1	0,26	4,67	2,24E-06	46 x 1	20 p11	mitochondrial genome maintenance exonuclease 1 [Source:HGNC Symbol;Acc:HGNC:16205]	
SMCHD1	0,26	4,61	2,93E-06	46 x 1	18 p11	structural maintenance of chromosomes flexible hinge domain containing 1 [Source:HGNC Symbol;Acc:HGNC:29090]	
CDCA7	0,25	4,56	3,63E-06	46 x 1	2 q31	cell division cycle associated 7 [Source:HGNC Symbol;Acc:HGNC:14628]	
ING3	0,23	4,19	1,85E-05	47 x 3	7 q31	inhibitor of growth family member 3 [Source:HGNC Symbol;Acc:HGNC:14587]	
ZNF143	0,23	4,13	2,34E-05	46 x 1	11 p15	zinc finger protein 143 [Source:HGNC Symbol;Acc:HGNC:12928]	
SLBP	0,23	4,07	2,97E-05	46 x 1	4 p16	stem-loop binding protein [Source:HGNC Symbol;Acc:HGNC:10904]	
MYO19	0,22	3,99	4,10E-05	46 x 1	17 q12	myosin XIX [Source:HGNC Symbol;Acc:HGNC:26234]	
HPRT1	0,21	3,81	8,32E-05	46 x 1	X q26	hypoxanthine phosphoribosyltransferase 1 [Source:HGNC Symbol;Acc:HGNC:5157]	
	0,21	3,76	0,0001022	46 x 1			
CEP97	0,20	3,64	0,0001623	46 x 1	3 q12	centrosomal protein 97 [Source:HGNC Symbol;Acc:HGNC:26244]	
INTS7	0,18	3,17	0,0008286	46 x 1	1 q32	integrator complex subunit 7 [Source:HGNC Symbol;Acc:HGNC:24484]	
